# Supplementary material for: Pace of passive margin tectonism revealed by U-Pb dating of fracture-filling calcite
Source: Nat Commun. 2022 Apr 12;13:1953. doi: 10.1038/s41467-022-29680-z (PMC9005700; doi:10.1038/s41467-022-29680-z)
Supplement: Supplementary file 1 — Supplementary Information [file 41467_2022_29680_MOESM1_ESM.pdf]

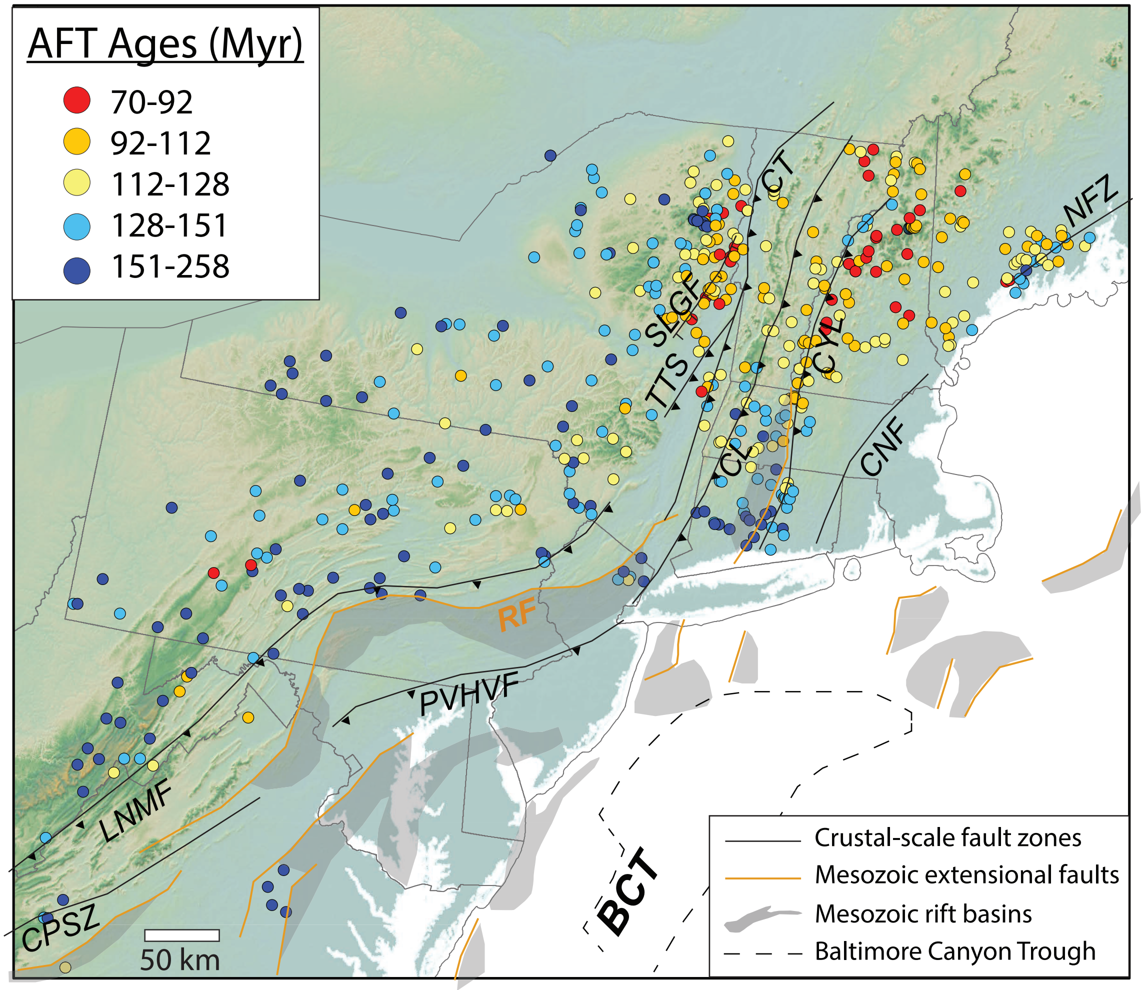

Figure S1) Published apatite fission-track (AFT) ages in the northeastern United States. The concentration of Late Cretaceous ages (orange and red dots) in northern New England records a significant amount of post-rift exhumation and/or a more recent thermal disturbance, presumably due to a tectonic disturbance. The large number of Late Cretaceous cooling ages broadly overlaps the timing of fracturing observed in this study from ~ 115 to 78 Ma. Rift basins from Withjack, 2012 and major fault zones from Hibbard et al., 2006. Ages compiled from: Blackmer et al., 1994; Duddy et al., 1986; Roden-Tice et al., 2012; Roden-Tice and Tice, 2005; Roden-Tice et al., 2000; Roden-Tice et al., 2009; Roden-Tice and Wintsch, 2002; Roden, 1991; Roden et al., 1989;

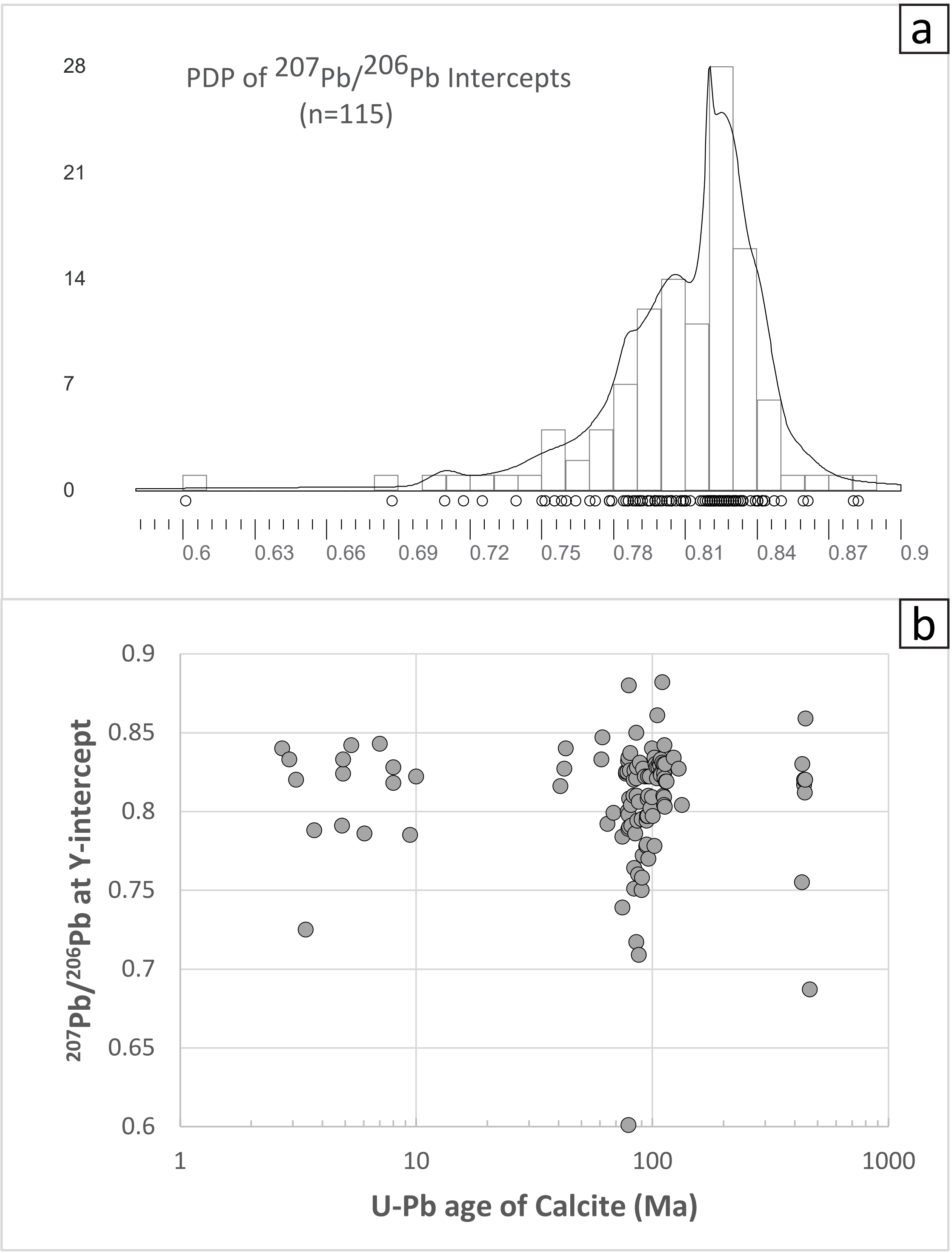

Figure S2) Summary of $^{207}Pb/^{206}Pb$ common lead ratios. a, Probability density plot (PDP) and histogram showing the distribution of y-intercept values for each calcite U-Pb age. These values are obtained by fitting an isochron line in $^{238}U/^{206}Pb$ vs. $^{207}Pb/^{206}Pb$ space and taking the upper Y-intercept. They are interpreted as the common lead ratio present in the crystal prior to radiogenic Pb ingrowth. b, Plot of calcite U-Pb age vs. y-intercept values. The lack of correlation implies isochron ages are not influenced by the common lead ratios and that most isochron ages record primary crystallization.
