# Supplementary material for: Pace of passive margin tectonism revealed by U-Pb dating of fracture-filling calcite
Source: Nat Commun. 2022 Apr 12;13:1953. doi: 10.1038/s41467-022-29680-z (PMC9005700; doi:10.1038/s41467-022-29680-z)
Supplement: Supplementary file 3 — Description of Additional Supplementary Files [file 41467_2022_29680_MOESM3_ESM.pdf]

Description of Additional Supplementary Files

File Name: Supplementary Data 1
Description: Summary table for calcite vein samples

File Name: Supplementary Data 2
Description: Stable isotope results
