# Supplementary material for: Pace of passive margin tectonism revealed by U-Pb dating of fracture-filling calcite
Source: Nat Commun. 2022 Apr 12;13:1953. doi: 10.1038/s41467-022-29680-z (PMC9005700; doi:10.1038/s41467-022-29680-z)
Supplement: Supplementary file 6 — Source Data [file 41467_2022_29680_MOESM6_ESM.zip › 1.zip!/appendix2_site_summaries_1to20/1_richville_reservoir_south_final.pdf]

1: Richville Reservoir South (43.87308, -73.27188)

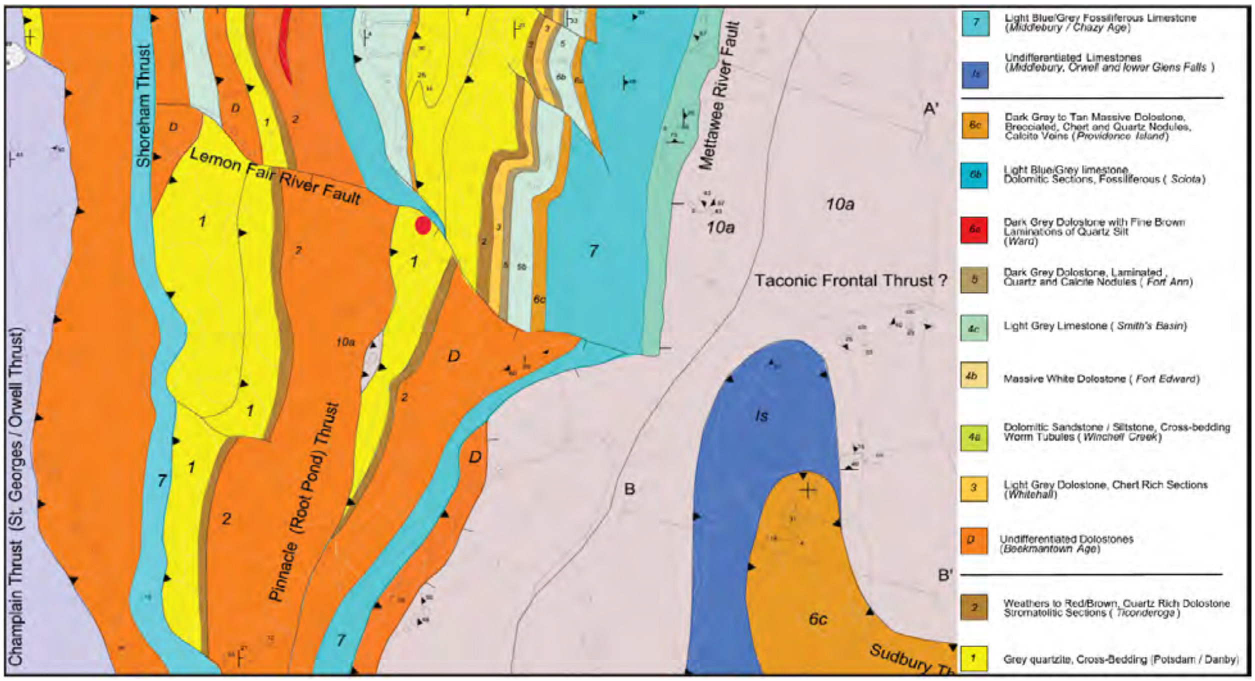

Figure 1: Sample located on geologic map of Hayman and Kidd, 1996.

Failed samples from same site: C002D, SG1A,C,

Bedrock Unit: Potsdam Quartzites (Haymann + Kidd, 2002) or Danby Formation (Cd)

Ages: ***Gen1*:** **C002**: 113 ± 12; **C002B**: 104 ± 13; **C002C**: 104.6 ± 7.4; **SG1B**: 106 ± 13;

***Gen2*:** **C002E**: 81.1 ±3.4; **C002A**: 79 ± 7;

Summary: This site is located on the south side of Richville Road in Shoreham, VT, roughly 100 m west of the bridge over the Lemon Fair River. It is stop 4a in a 2002 NEIGC guidebook chapter by Haymann and Kidd. It sits in the hanging wall of the Lemon Fair Fault, immediately south of the fault (Figure 1). The primary geologic feature is a roughly N-S oriented fold axis, presumably Taconic in age, defined by beds of the Danby/Potsdam quartzite, with a trend of 156 and a plunge of 14 (Fig. 2).

This site is one of the best studied in this paper because it was the subject of a Middlebury College senior thesis project by Sam Graf in 2019. This thesis provides the outcrop-scale measurements (Fig. 2) as well as the regional fracture mapping shown below (Figures 3-5). These two datasets agree well, pointing to two dominant, orthogonal, fracture sets, one at roughly 160/340° and another at roughly 60/250°. Interestingly, none of these fractures have offset along them. Interestingly, only the first N-S set is mineralized at Site 1, whereas and the other E-W set is defined by 'dry' fractures.,

The outcrop at site 1 contains two main types of fractures that cross cut or exploit specific rigid beds within the older fold. The first is a set of thick (1-3 cm), sparry, discontinuous (~5-20 cm in length) calcite veins which are entirely confined within one bed and appear to have been formed as a result of high fluid pressures within the bed (C002,C002B,C). In places they have chaotic, angular geometry with

chunks of host rock incorporated in the calcite, suggestive of implosion breccias opening under high fluid pressure (see photos below for C002 and C002C). These veins are oriented NE-SW, sub-parallel to each other, and generally parallel to the fold axis, dating to between ~105 and 112 Ma, with low $^{238}U/^{206}Pb$ ratios leaving uncertainty on the ages. Lack of evidence for slip, sigmoidal shape, and en-echelon geometries leads us to interpret these as mode-1 veins formed under a NE-SW $\sigma 3$ stress at ~104 Ma, and possibly in an earlier event at ~113 Ma. Interestingly, these two ages overlap the timing of volcanism in the Taconic lobe near Rutland Vermont, where dikes have been dated to 107-104 Ma and the Cuttingsville Pluton was emplaced near 103 Ma.

A second set of veins occurs to the west of the fold axis and consists of more continuous (meter scale) and highly planar fractures filled with sparry, transluscent calcite with no slickenfibres or offset (C002A, E). These two fractures are interpreted as mode 1 fractures formed under an ENE-WSW $\sigma 3$ stress regime at ~79 and 81 Ma, respectively. This inferred stress regime agrees very well with what is observed at site 5 immediately to the north and may record a period of extension along the Lemon Fair Fault. Similar to site 5, we acknowledge that some of these N-S fractures may have originally formed as conjugates with the 'dry' E-W set shown in figure 2 during Taconic time. However, the fact that only the NW-SE oriented fractures contain calcite veins is evidence that they were reopened by a roughly NE-SW stress regime that did not favor opening of the 'dry' conjugate set.

Field Observations:

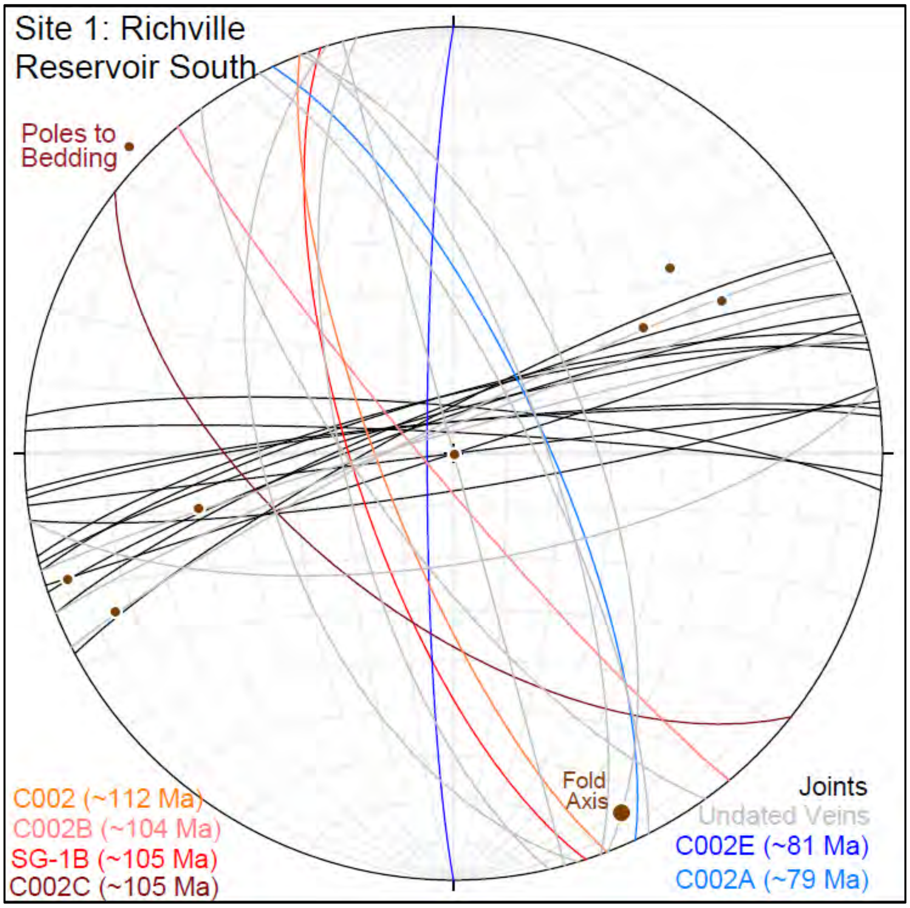

Figure 2: Stereonet showing the 6 dated veins as well as a set of E-W oriented undated ‘dry’ fractures and a set of roughly orthogonal undated veins at a similar orientation to the dated samples. Poles to bedding are shown as red dots and define a best-fit fold axis denoted by the larger red dot.

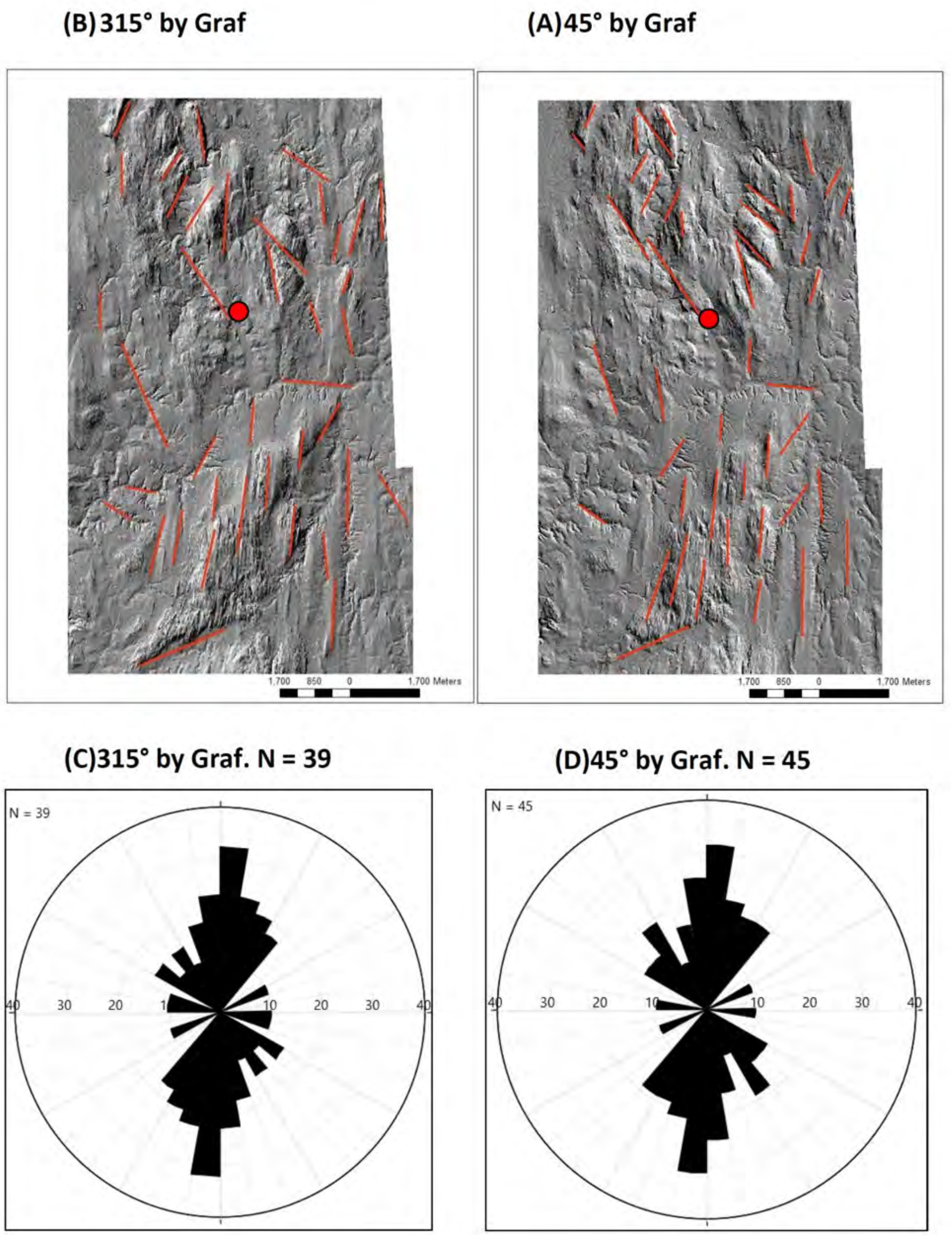

Figure 3: Lineaments mapped by the Sam Graf (thesis student) around the Richville Reservoir study site using 8x14 km hillshade images with azimuth angles 315° (left) and 45° (right). Study site is shown by red dot. Rose diagrams depict a general north-south trend of lineaments.

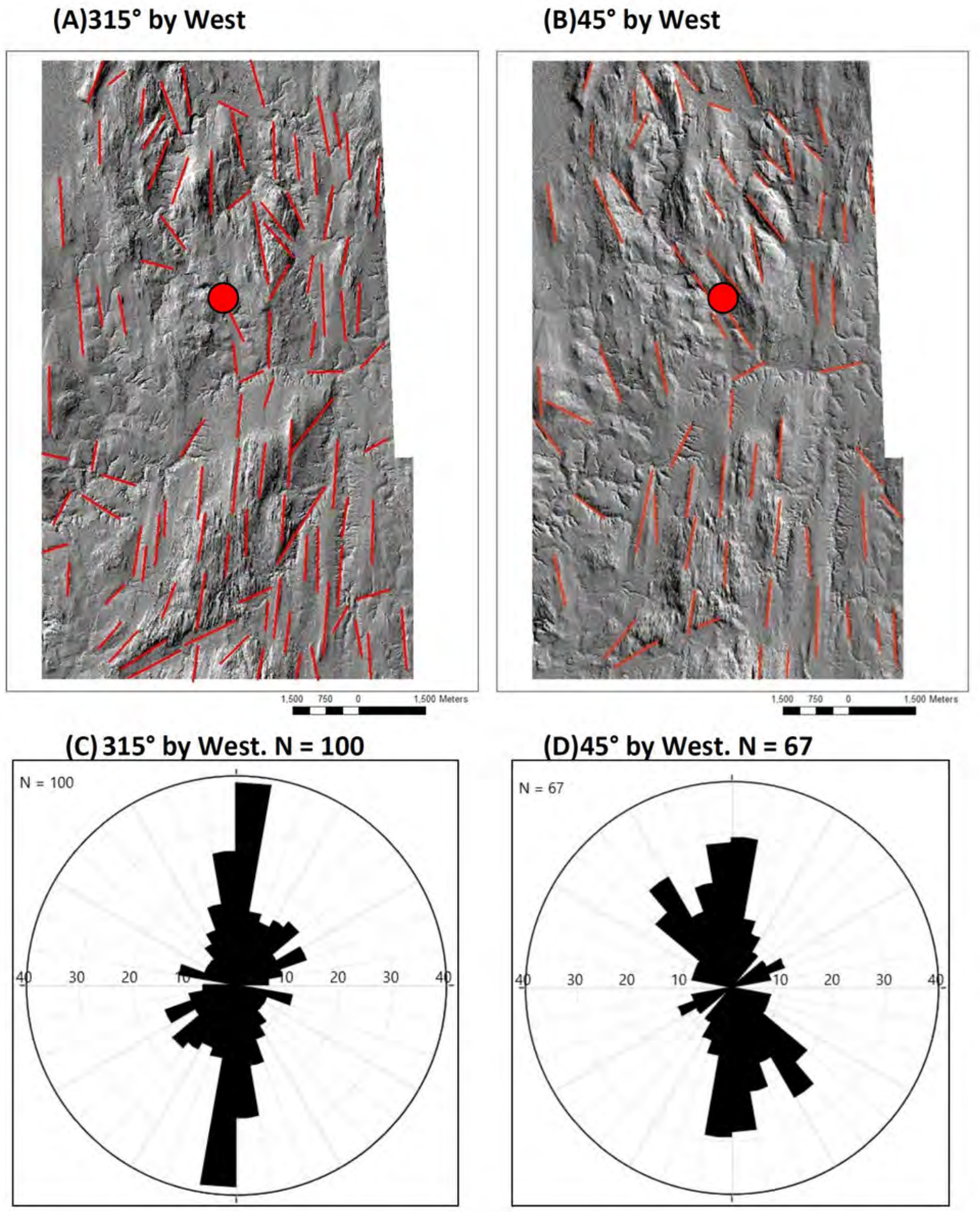

Figure 4: Lineaments mapped by Professor David West around the Richville Reservoir study site using 8x14 km hillshade images with azimuth angles 315° (left) and 45° (right). Study site is shown by red dot. Rose diagrams depict a general north-south trend of lineaments.

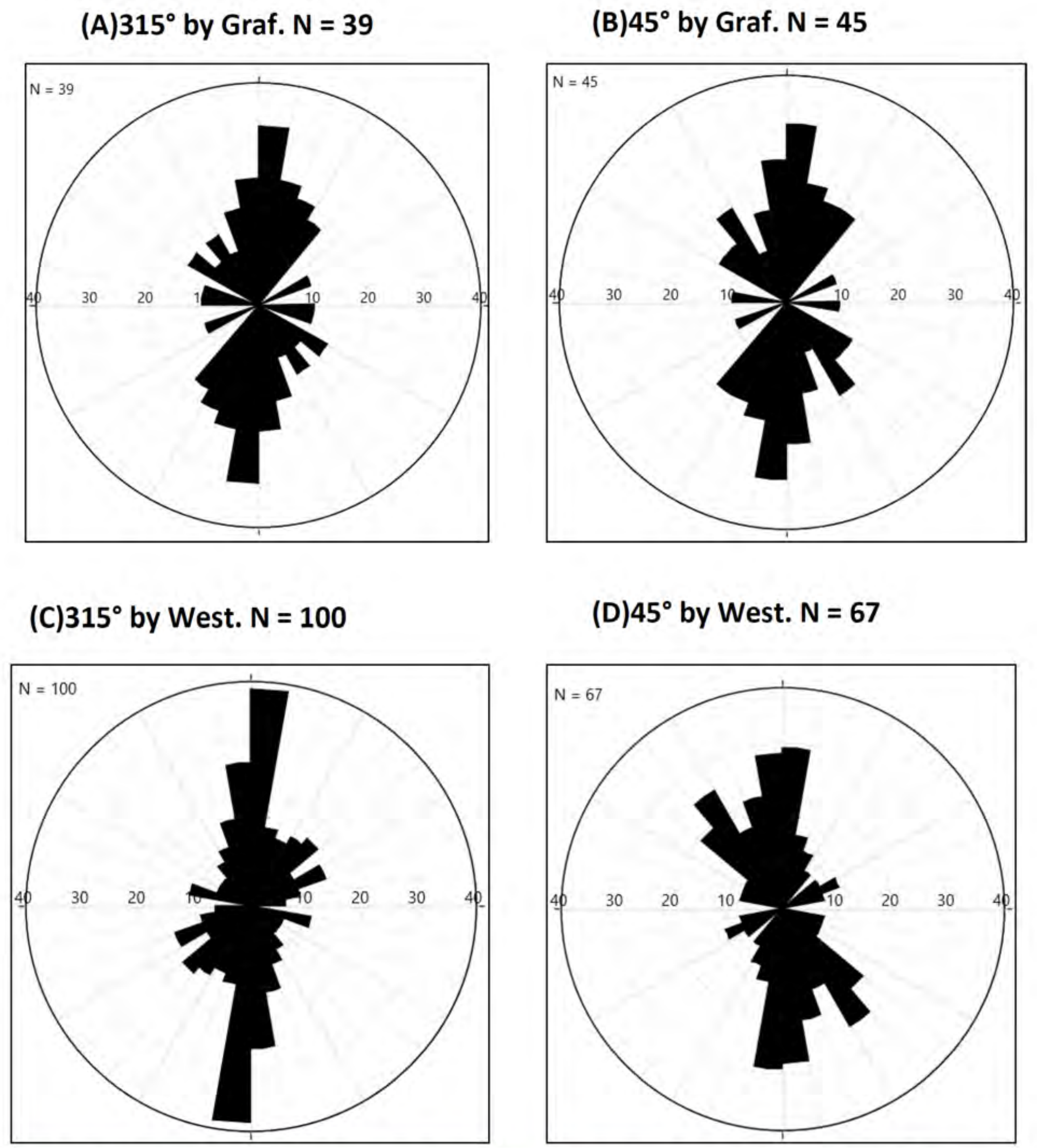

Figure 5: Side-by-side comparison of rose diagrams of lineaments mapped by Sam Graf (thesis student) and Professor Dave West using hillshade images from azimuth angles 315° (left) and 45° (right).

Overview Photos

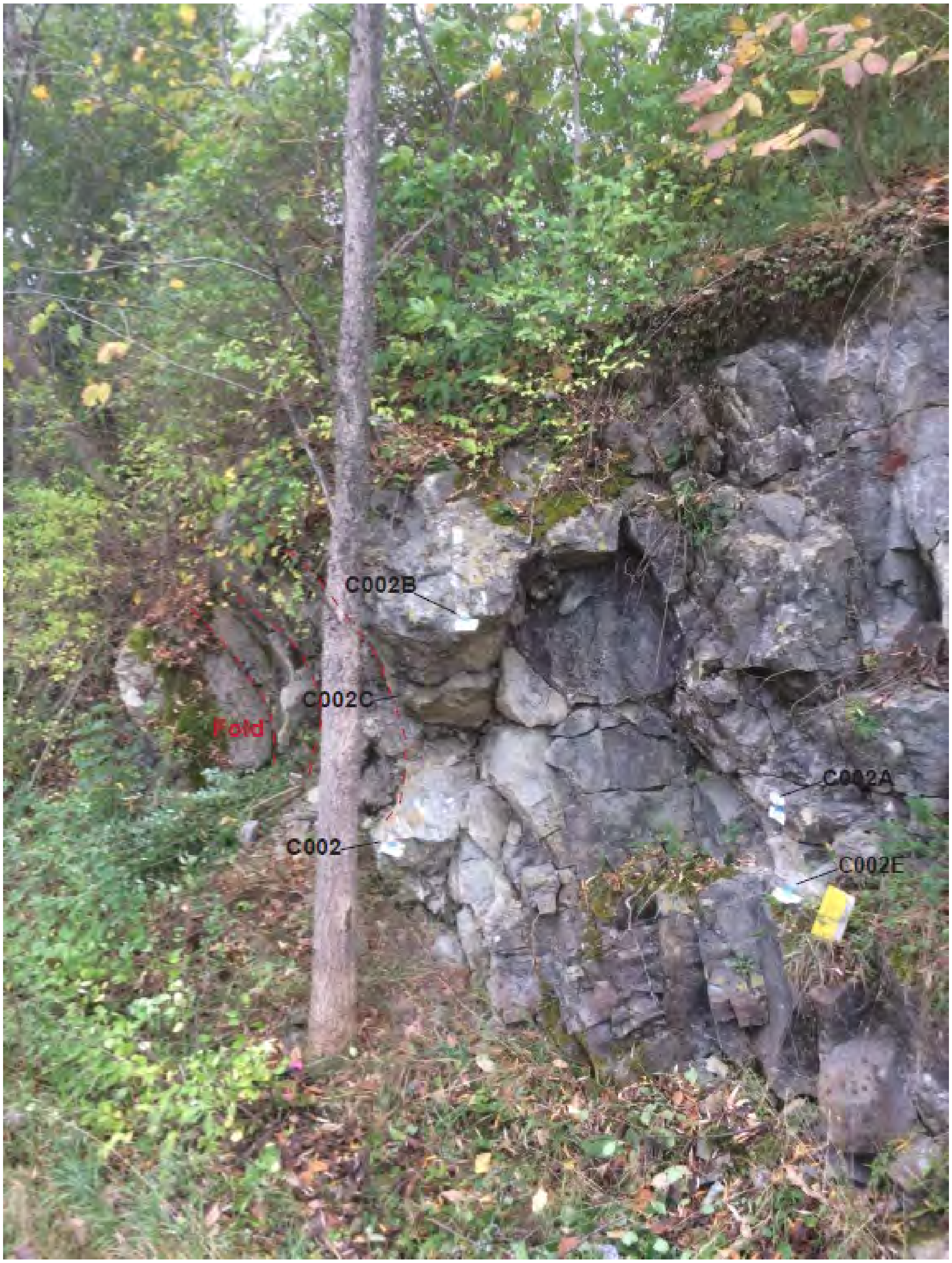

C002:

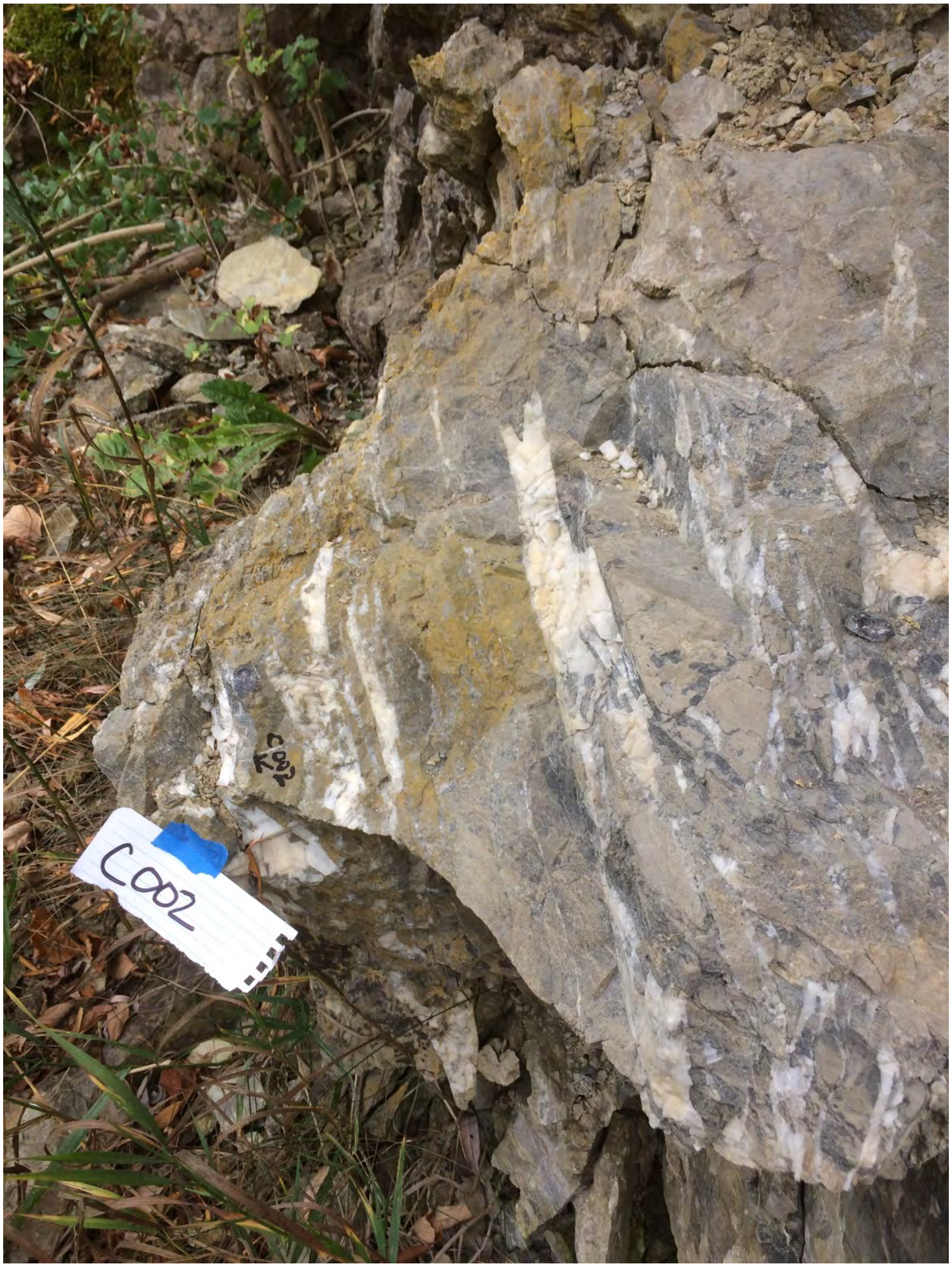

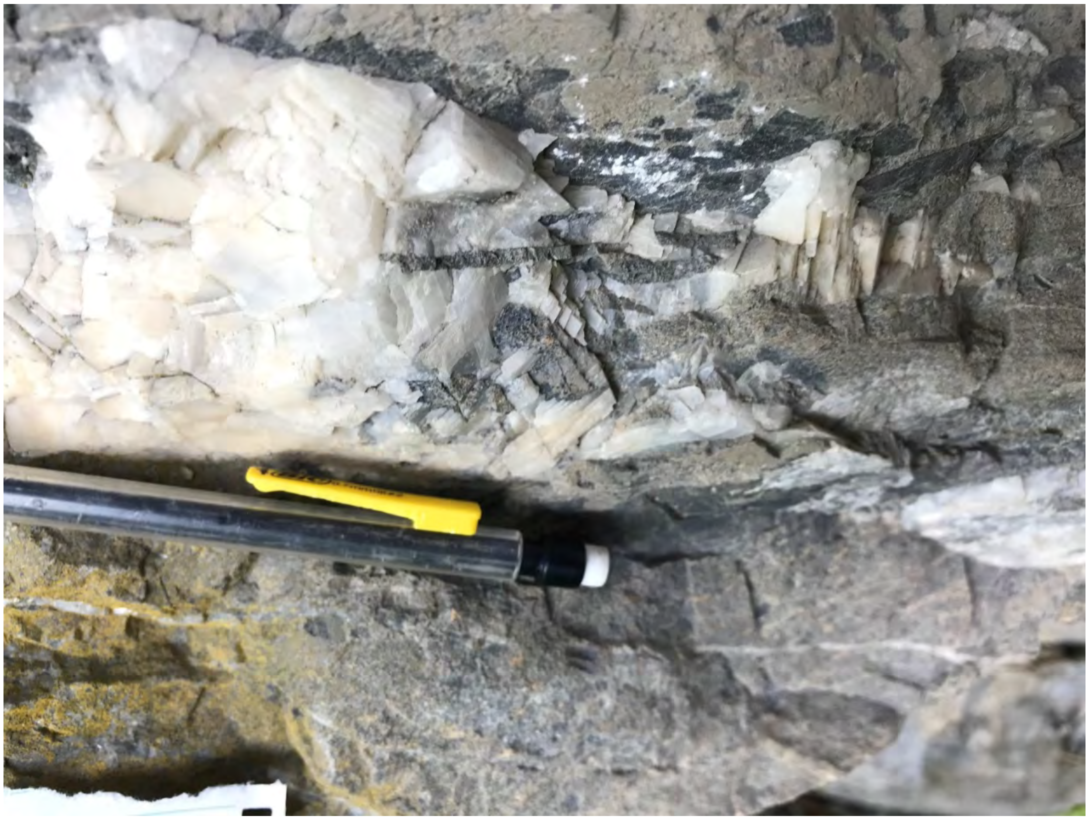

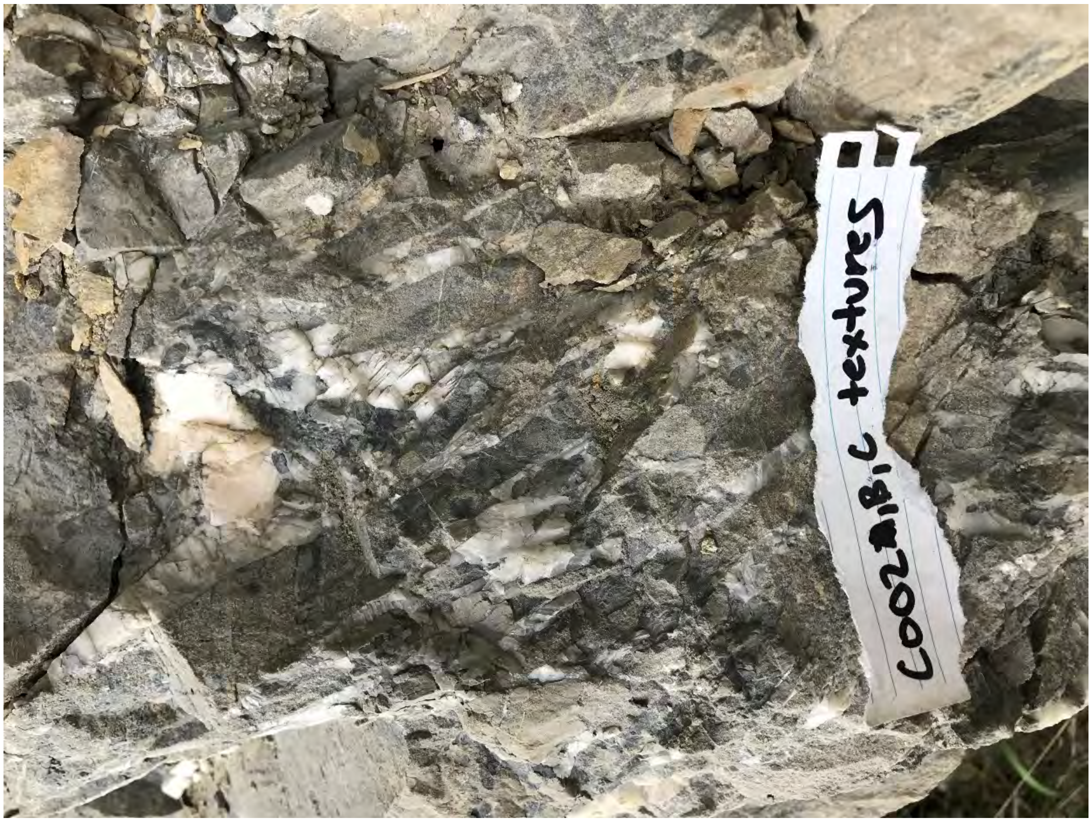
C002B,C textures

C002A + SG-1B:

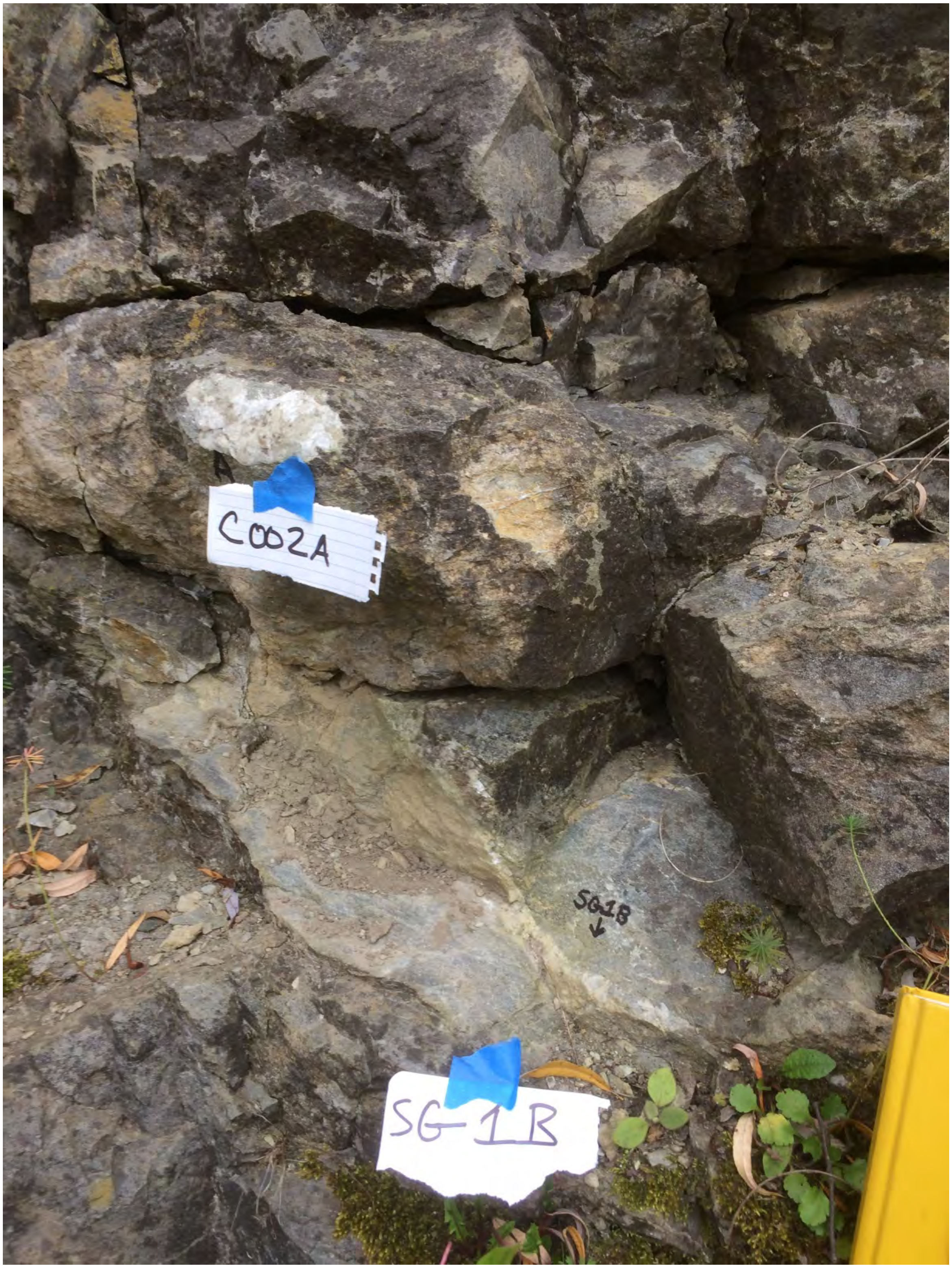

C002B:

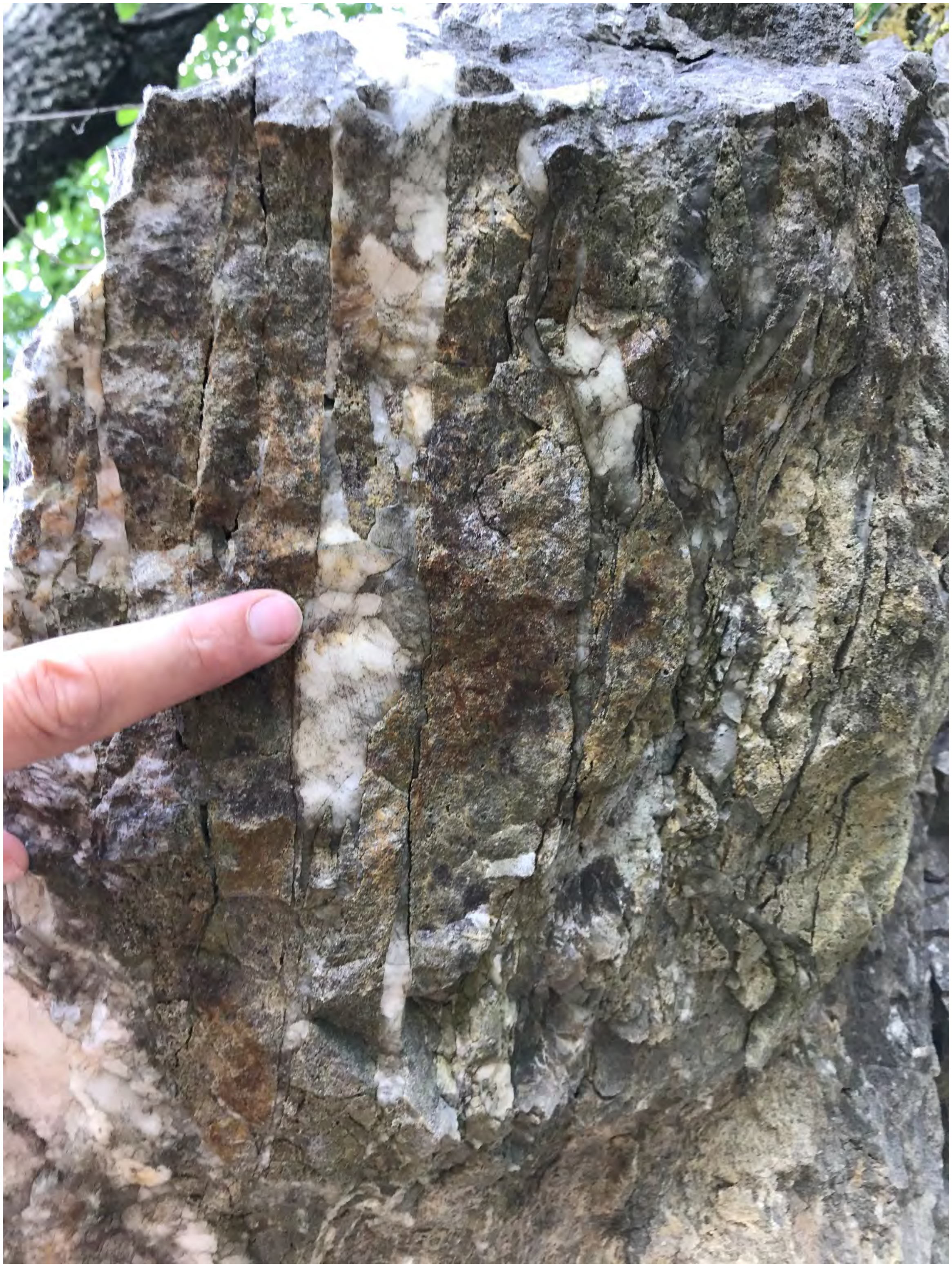

C002C:

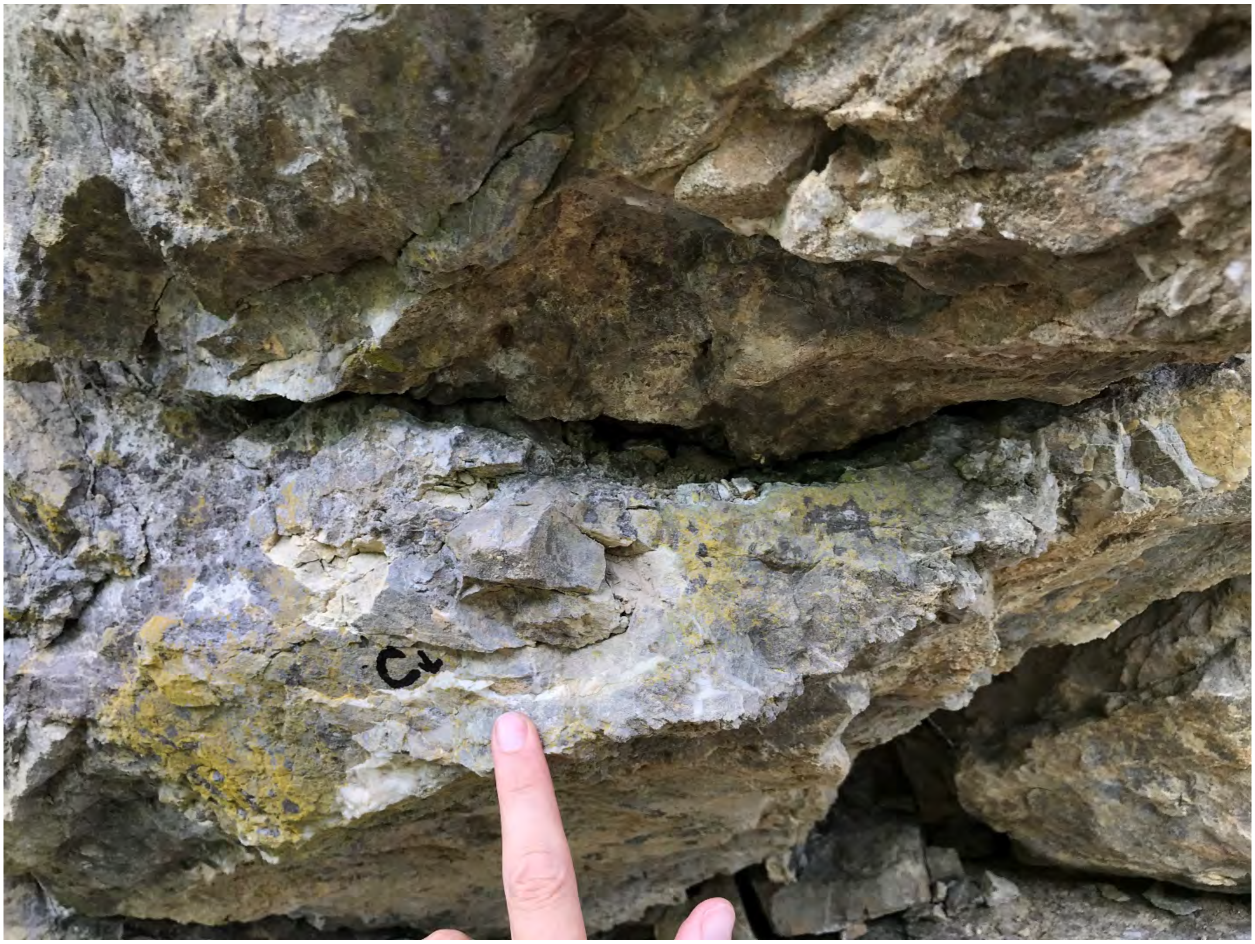

C002E:

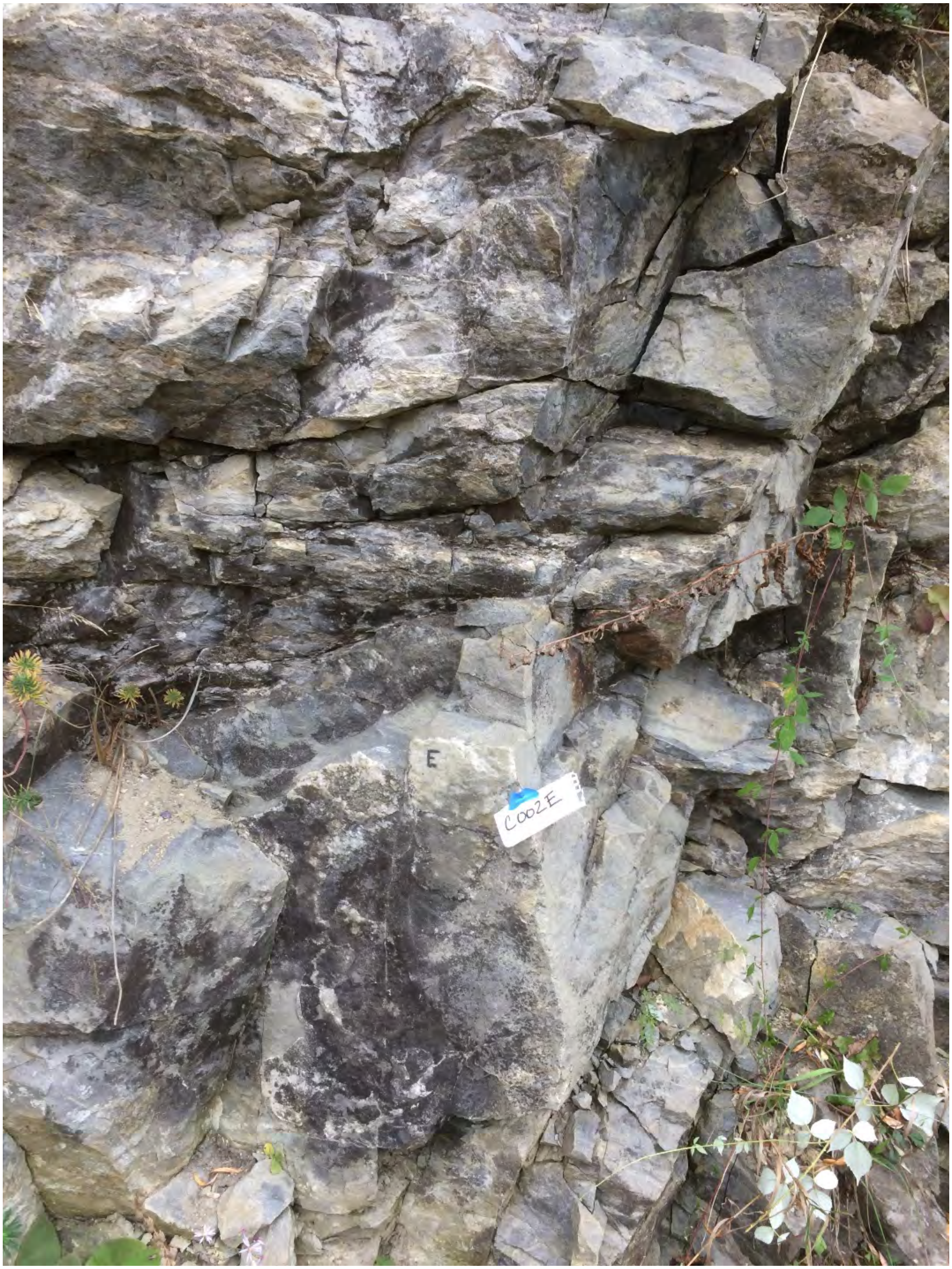

Lab Observations

C002: no optical image, analyzed at UCSB during first visit in fall, 2016.

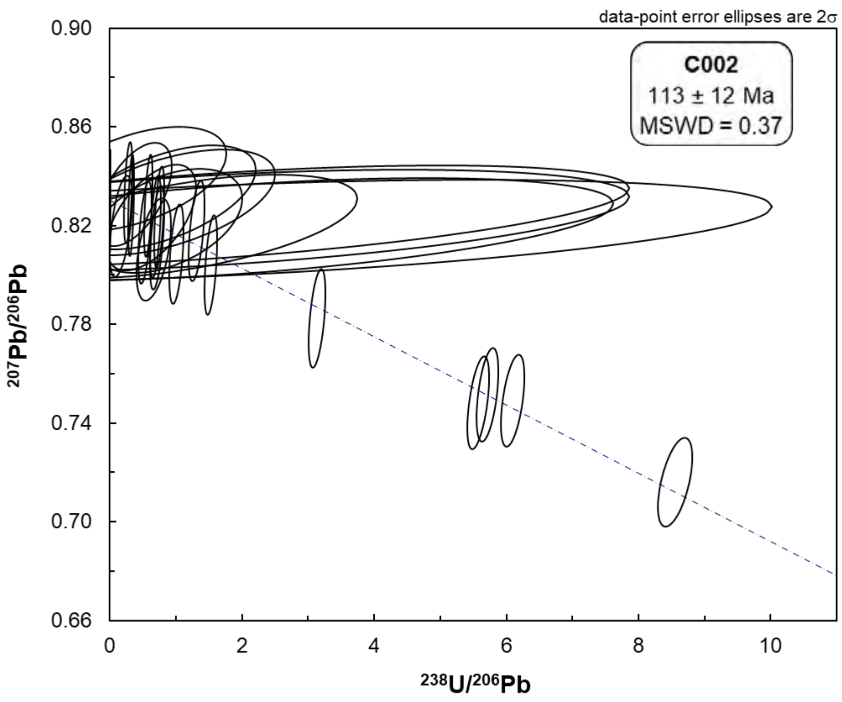

C002A: Circles in microscope image show areas targeted for dating (not laser shots)

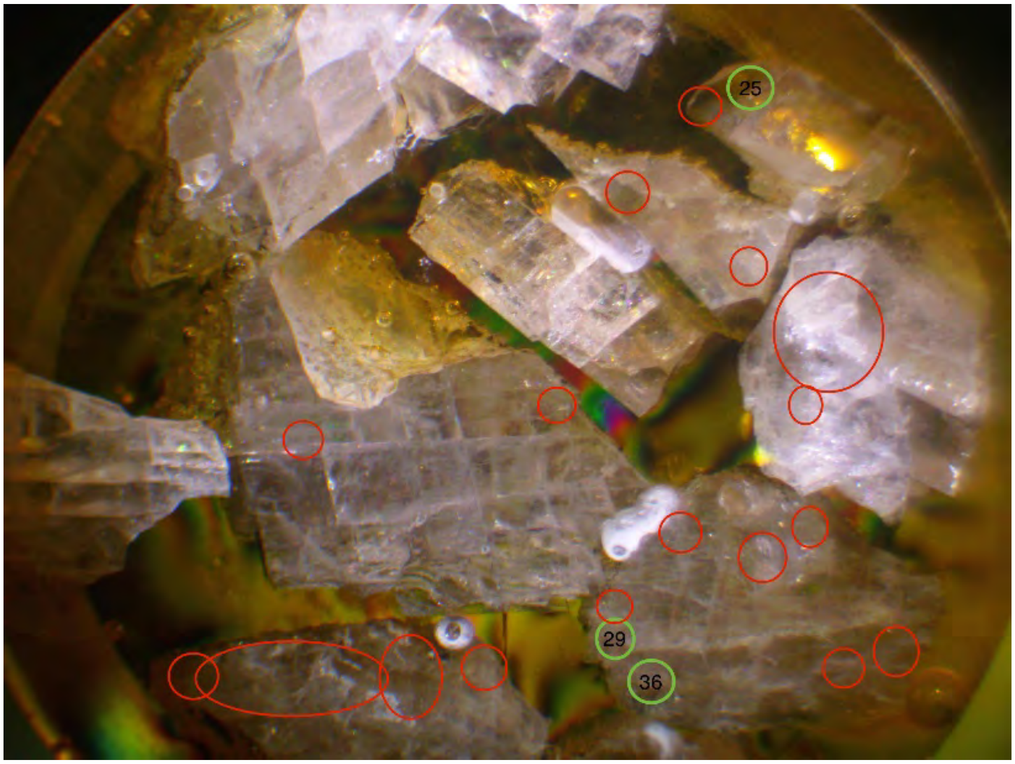

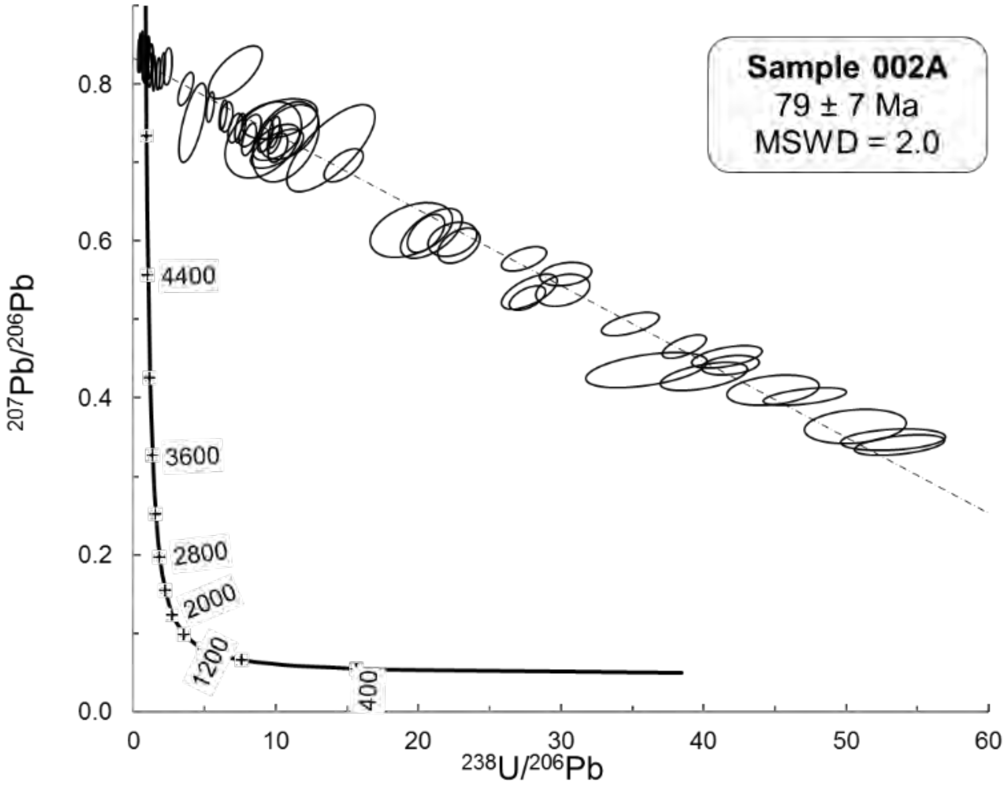

C002B

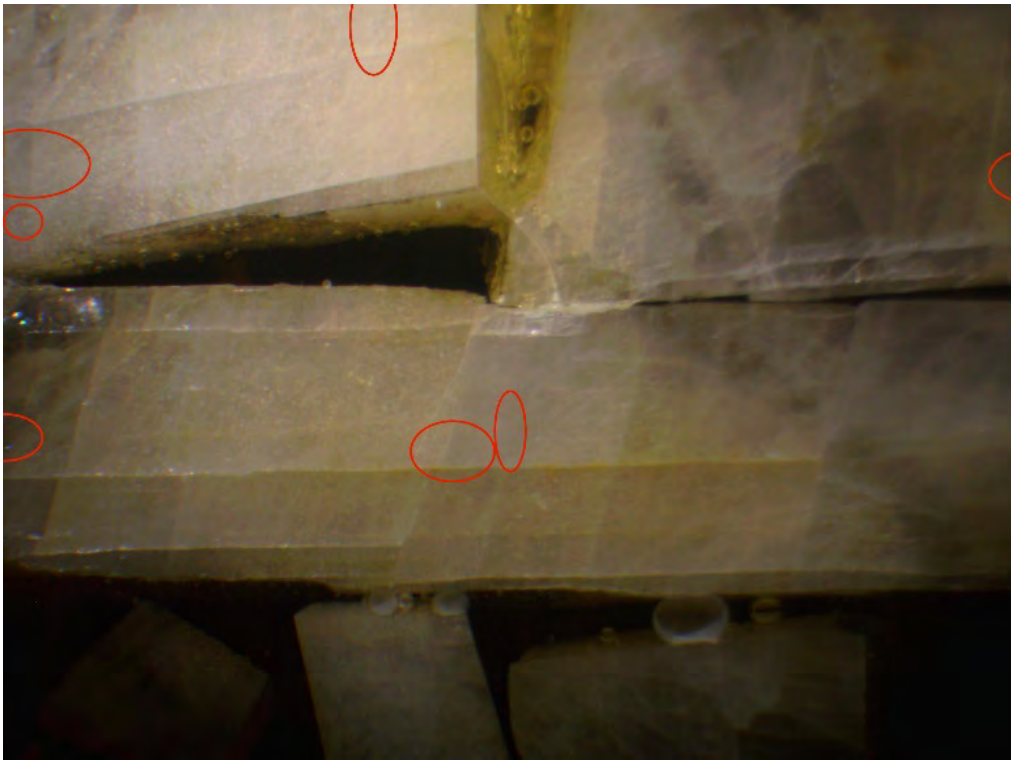

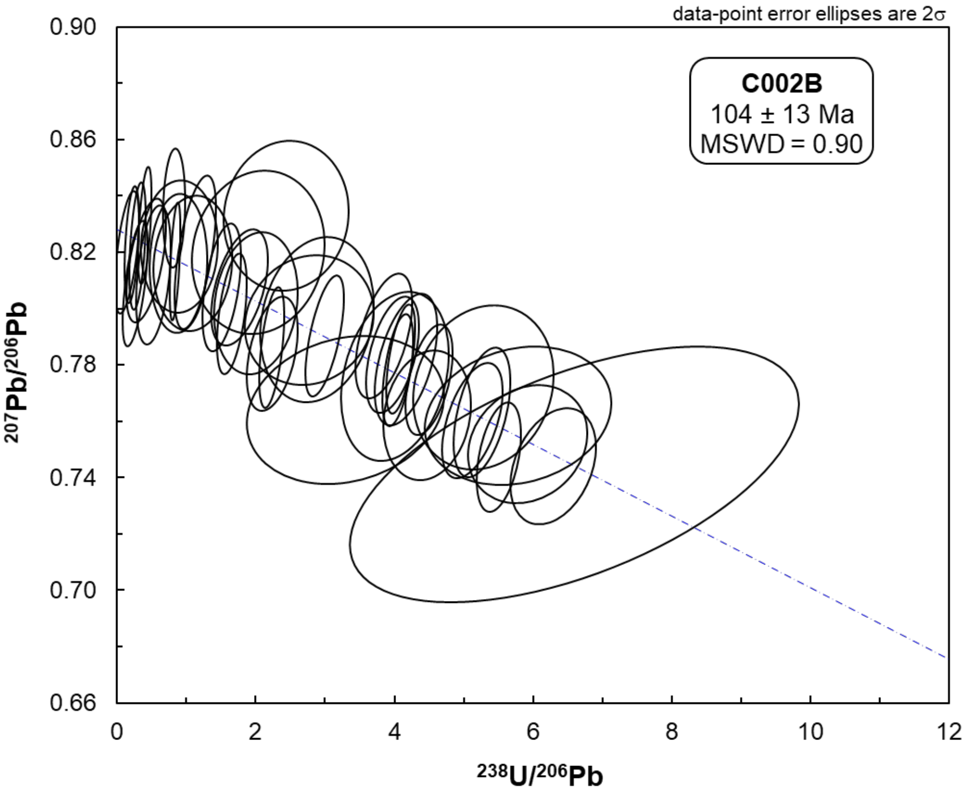

data-point error ellipses are 2σ
C002B
104 ± 13 Ma
MSWD = 0.90
0.90
0.86
0.82
0.78
0.74
0.70
0.66
0
2
4
6
8
10
12
$^{207}Pb/^{206}Pb$
$^{238}U/^{206}Pb$

C002C

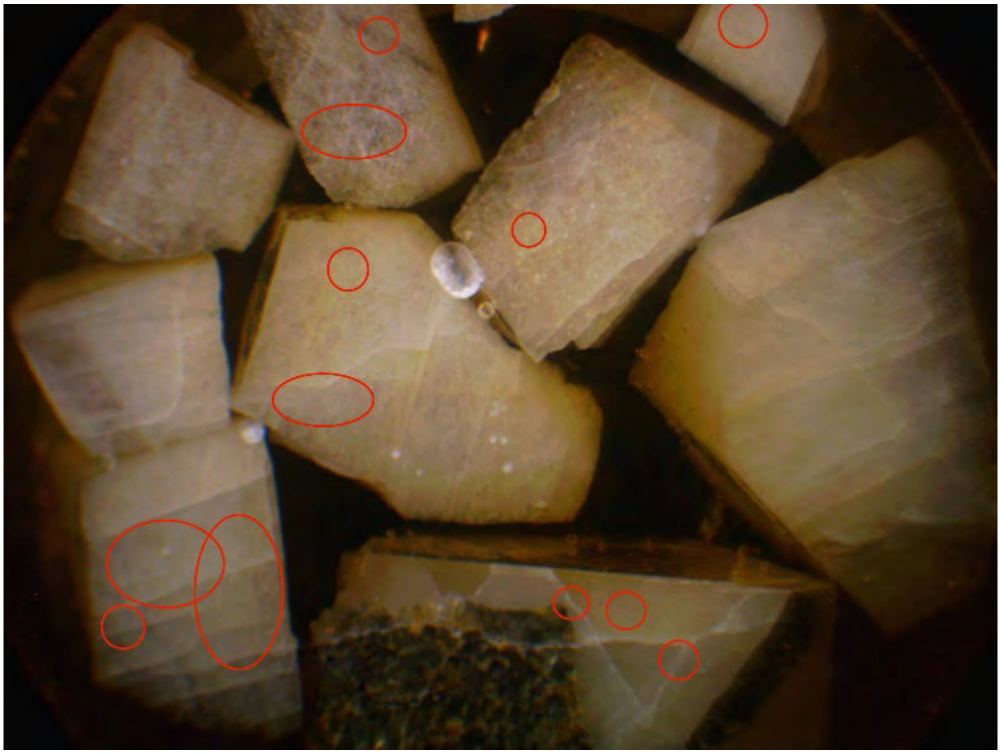

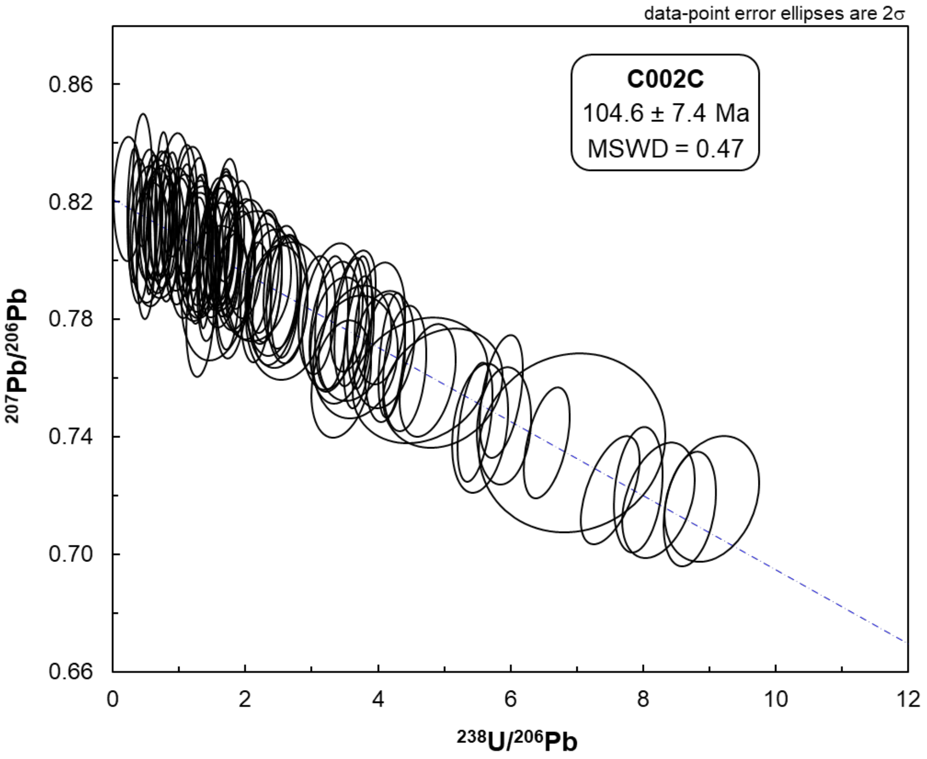
data-point error ellipses are 2σ
C002C
104.6 ± 7.4 Ma
MSWD = 0.47
0.86
0.82
0.78
0.74
0.70
0.66
0
2
4
6
8
10
12
207Pb/206Pb
238U/206Pb

C002E

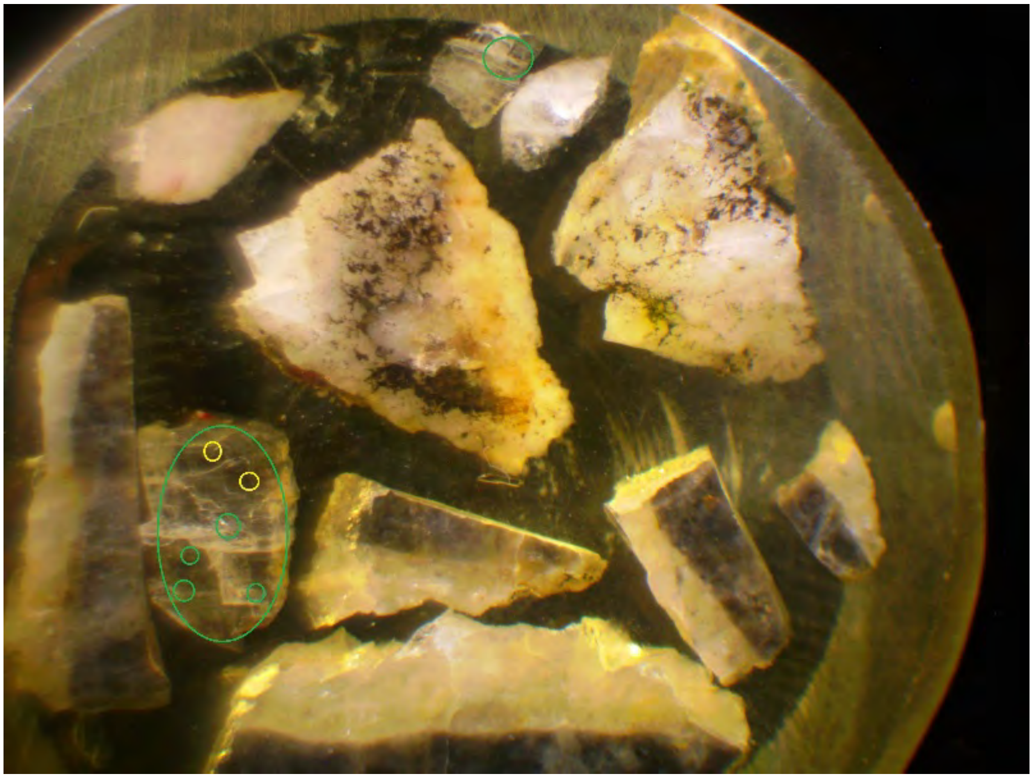

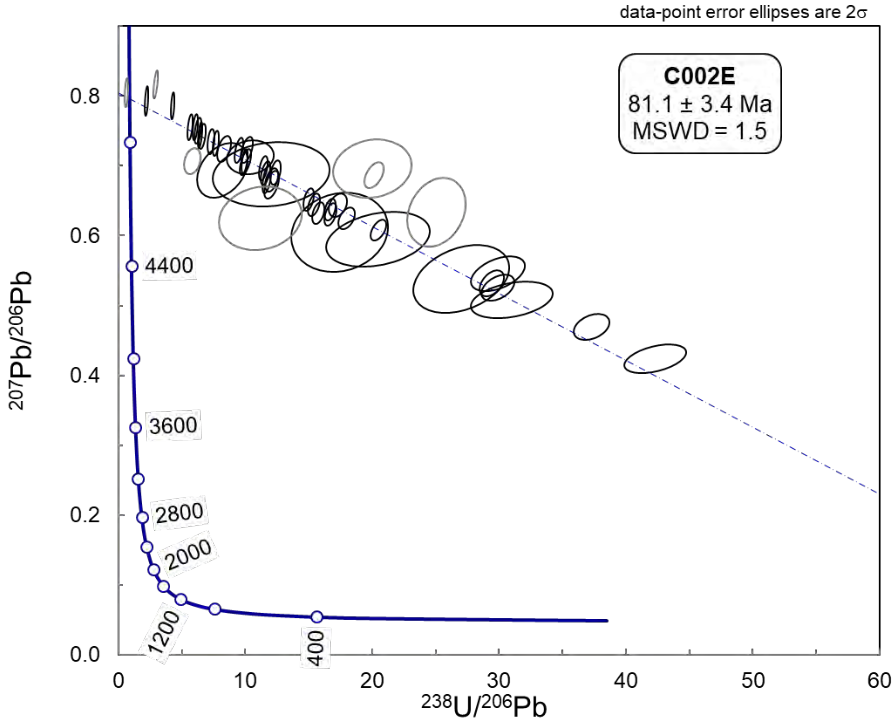

data-point error ellipses are 2σ
C002E
81.1 ± 3.4 Ma
MSWD = 1.5
0.8
0.6
0.4
0.2
0.0
4400
3600
2800
2000
1200
400
0
10
20
30
40
50
60
207Pb/206Pb
238U/206Pb

SG1-B:

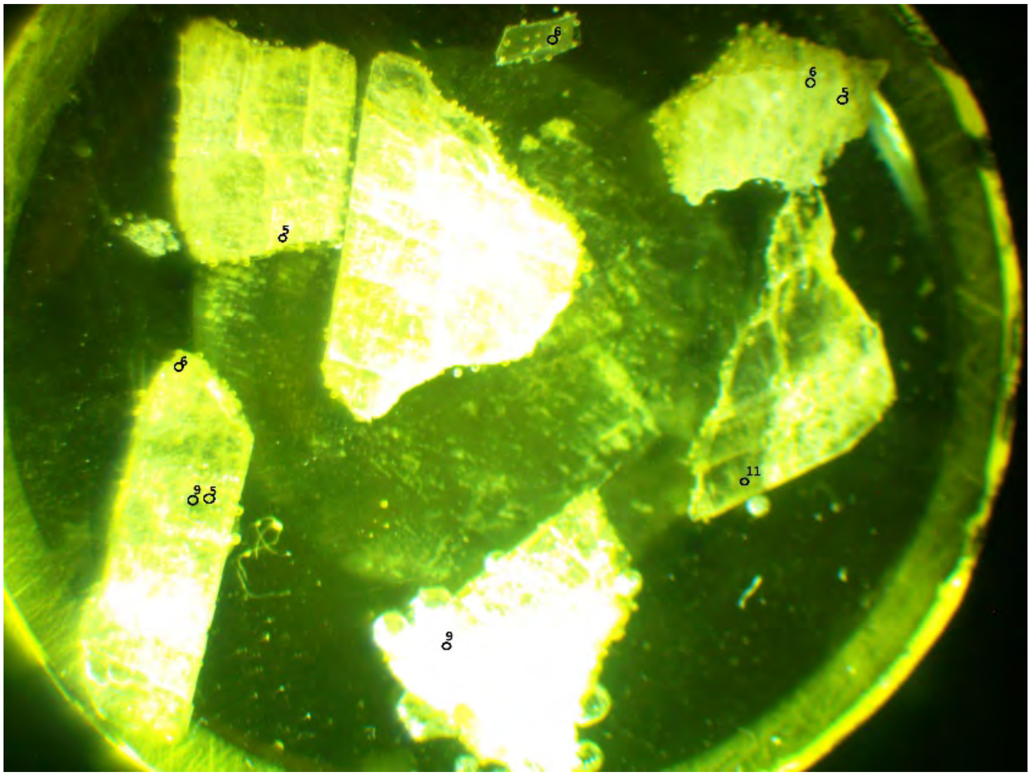
6
6
5
5
6
9
5
11
9

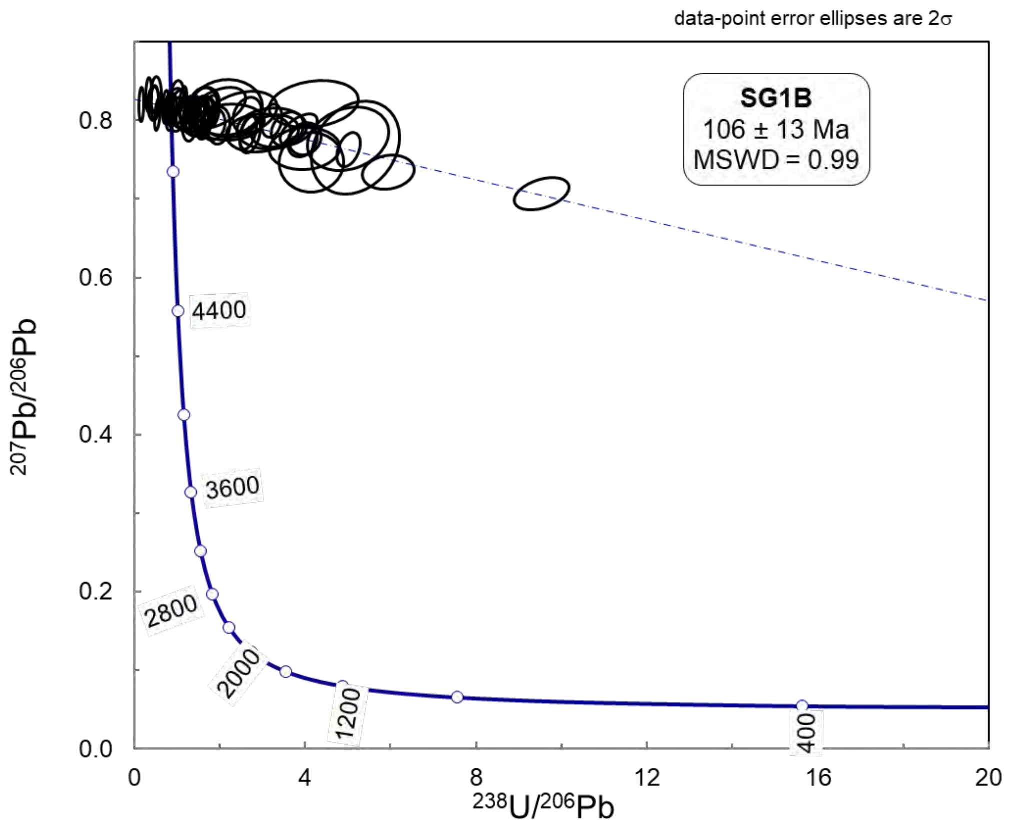
data-point error ellipses are 2σ
SG1B
106 ± 13 Ma
MSWD = 0.99
4400
3600
2800
2000
1200
400
0.0
0.2
0.4
0.6
0.8
0
4
8
12
16
20
207Pb/206Pb
238U/206Pb
