# Supplementary material for: Pace of passive margin tectonism revealed by U-Pb dating of fracture-filling calcite
Source: Nat Commun. 2022 Apr 12;13:1953. doi: 10.1038/s41467-022-29680-z (PMC9005700; doi:10.1038/s41467-022-29680-z)

10: Middlebury College Campus (44.009654, -73.178541)

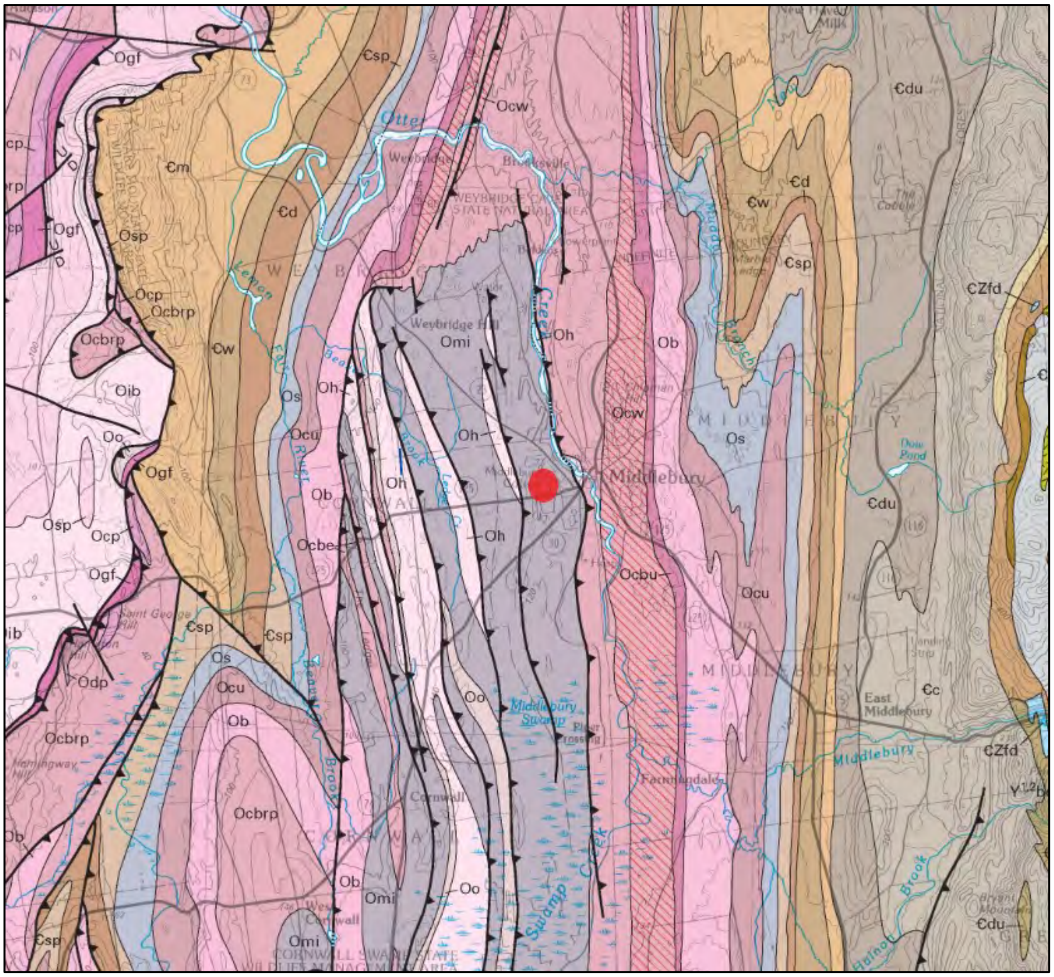

Figure 1: Sample location on state geologic map of Ratcliffe at al. (2011) (red dot slightly mislocated to north)

<u>Number of failed samples from same site</u>: C181b,c,d,e

<u>Bedrock Unit:</u> Middlebury Limestone (Omi)

<u>Ages</u>: **C181**: 112.4 ± 7.2 Ma

<u>Summary</u>: Site is located near base of a large rock exposure near the center of Middlebury College Campus. The Middlebury limestone at this site contains two well-developed cleavage sets, one of which has well-preserved down-dip tiling and slickenfibres recording E-W extension. Sample C181 yields a single domain U-Pb isochron suggesting the observed growth striae can be reliably linked to the age of ~112 Ma. This site is interpreted as a mode 2 shear fracture recording E-W extension. Unfortunately four additional samples from the same site proved undateable.

Field Observations:

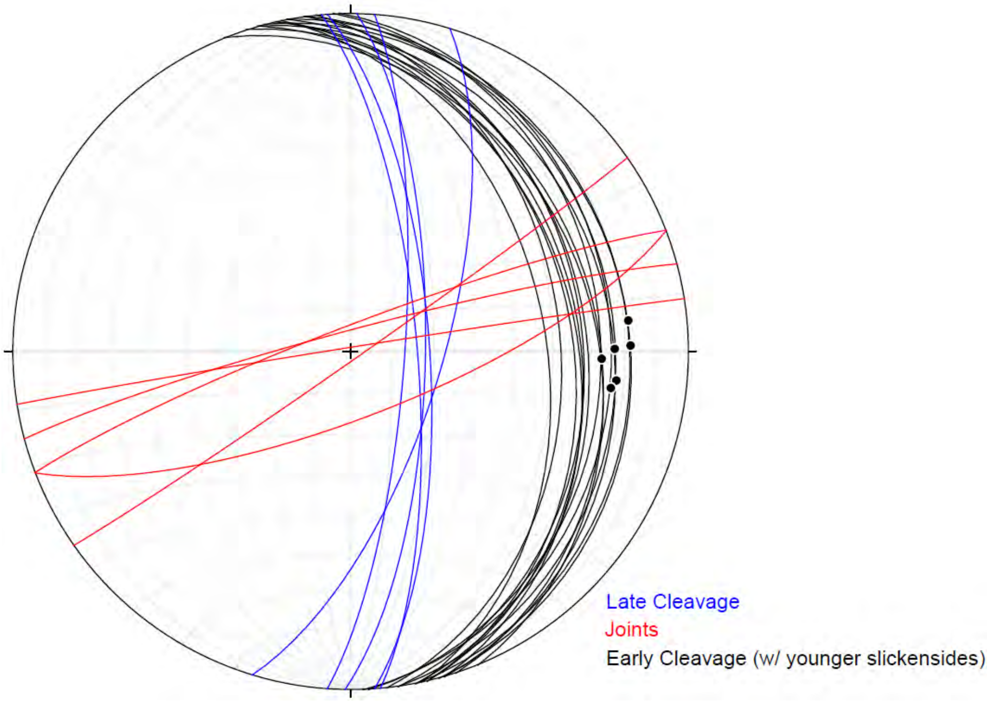

Figure 2: Stereonet plot showing late and early cleavage generations. The latter has been reactivated with E-W extensional slip at ca. 112 Ma.

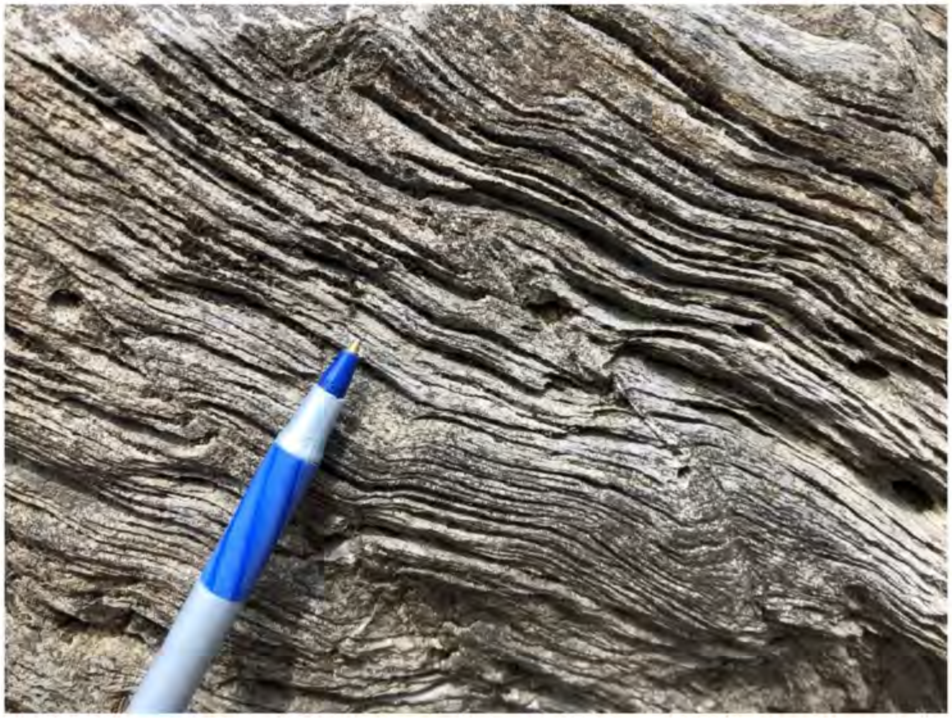

Figure 3: Early cleavage generation.

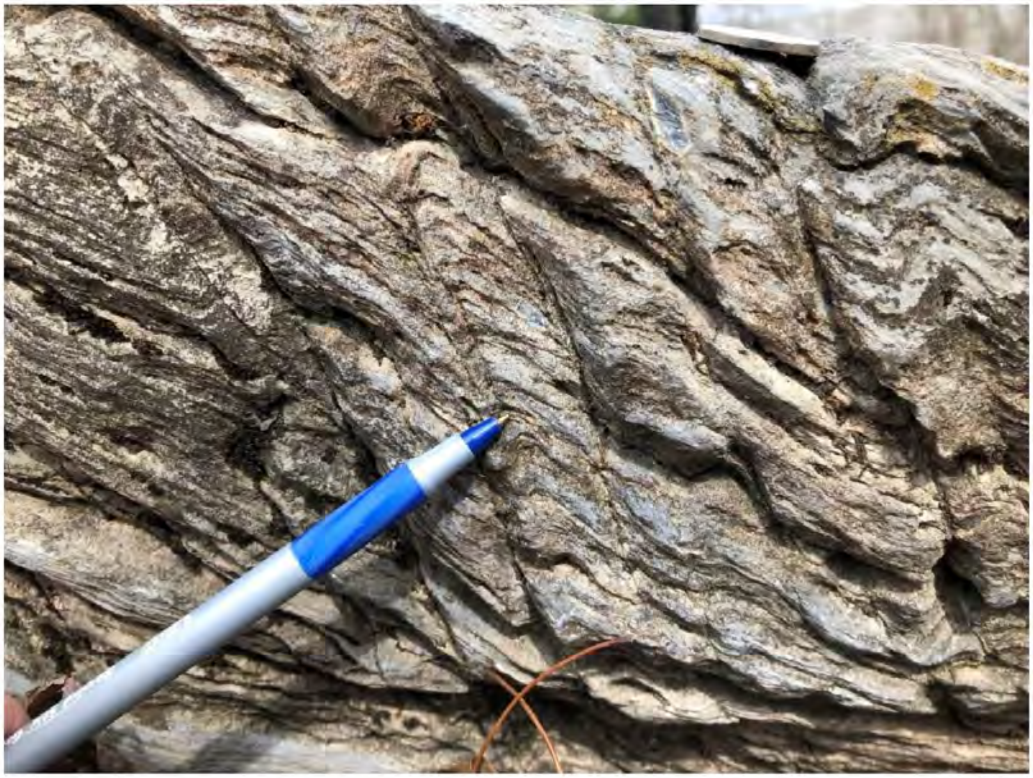

Figure 4: Early cleavage generation cross-cut by higher angle late cleavage.

Regional Lineament Analysis:

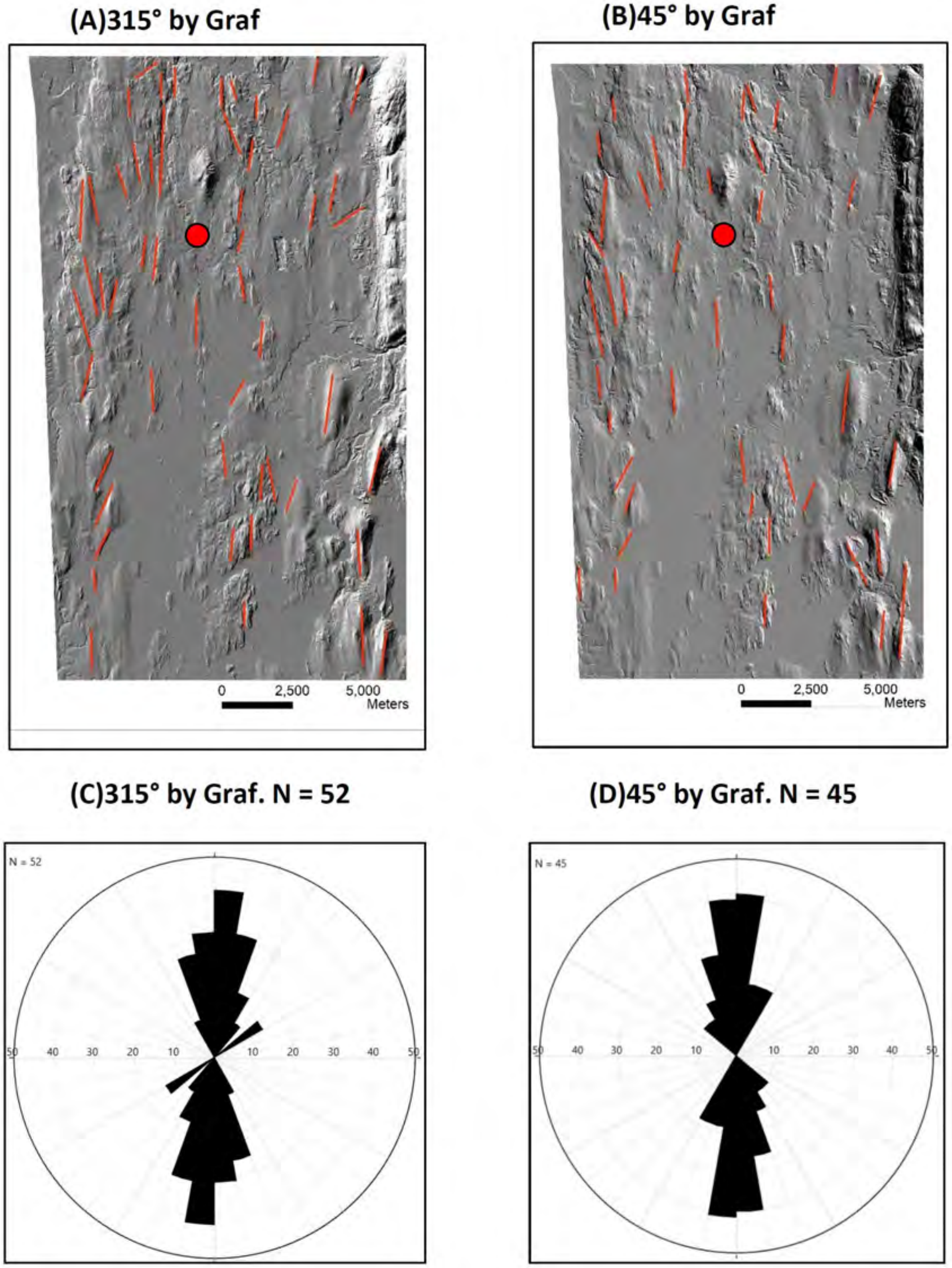

Figure 4: Lineaments mapped by student Sam Graf around the Middlebury College study site using hillshade images with azimuth angles 315° (left) and 45° (right). Study site is shown by red dot. Rose diagrams depict a general north to south trend of plotted lineaments, in good agreement with cleavage orientation at the outcrop.

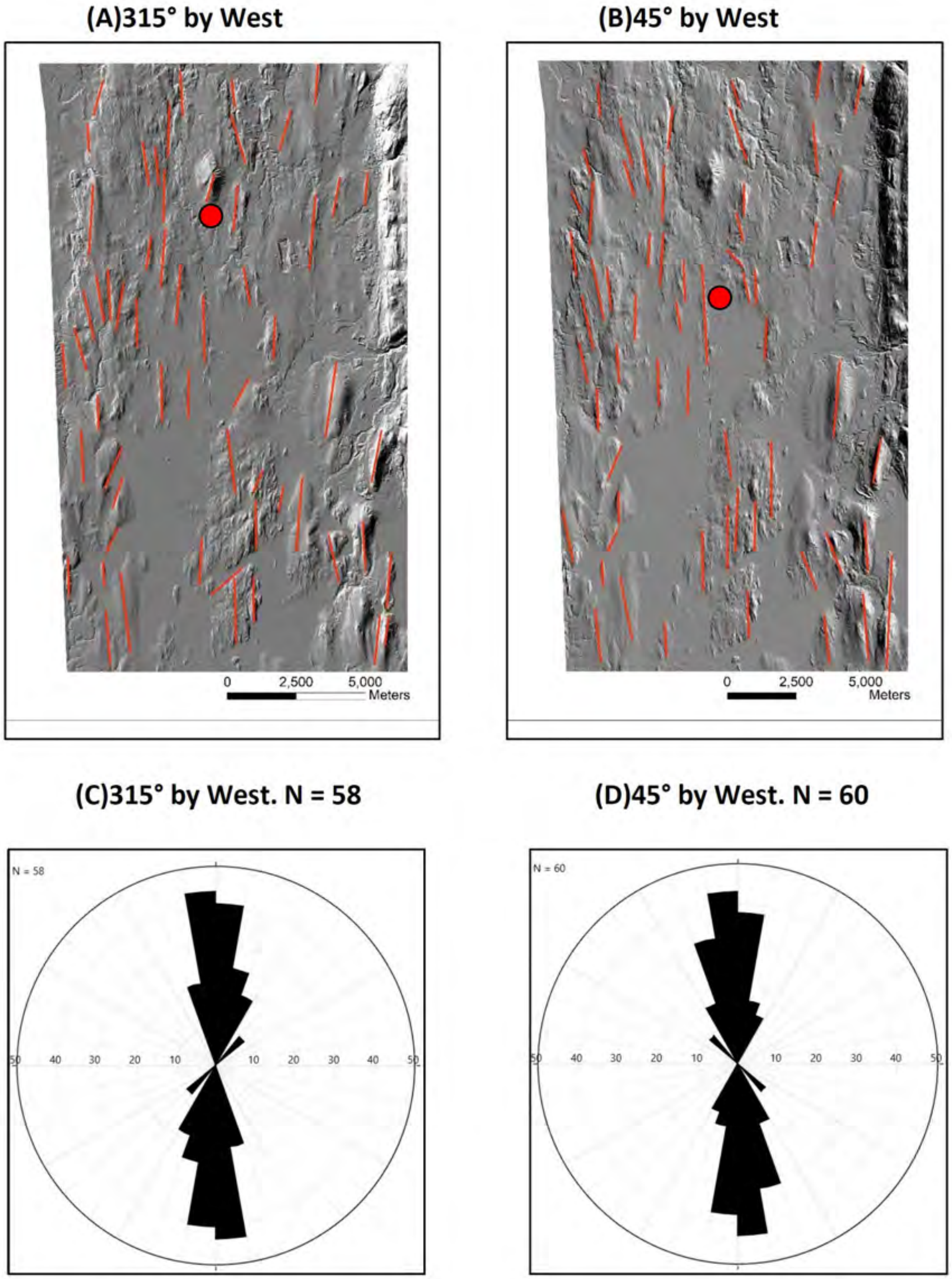

Figure 5: Lineaments mapped by Professor Dave West around the Middlebury College study site using hillshade images with azimuth angles 315° (left) and 45° (right). Study site is shown by red dot. Rose diagrams depict a general north to south trend of plotted lineaments, in good agreement with cleavage orientation at the outcrop.

Field Sample Photos:

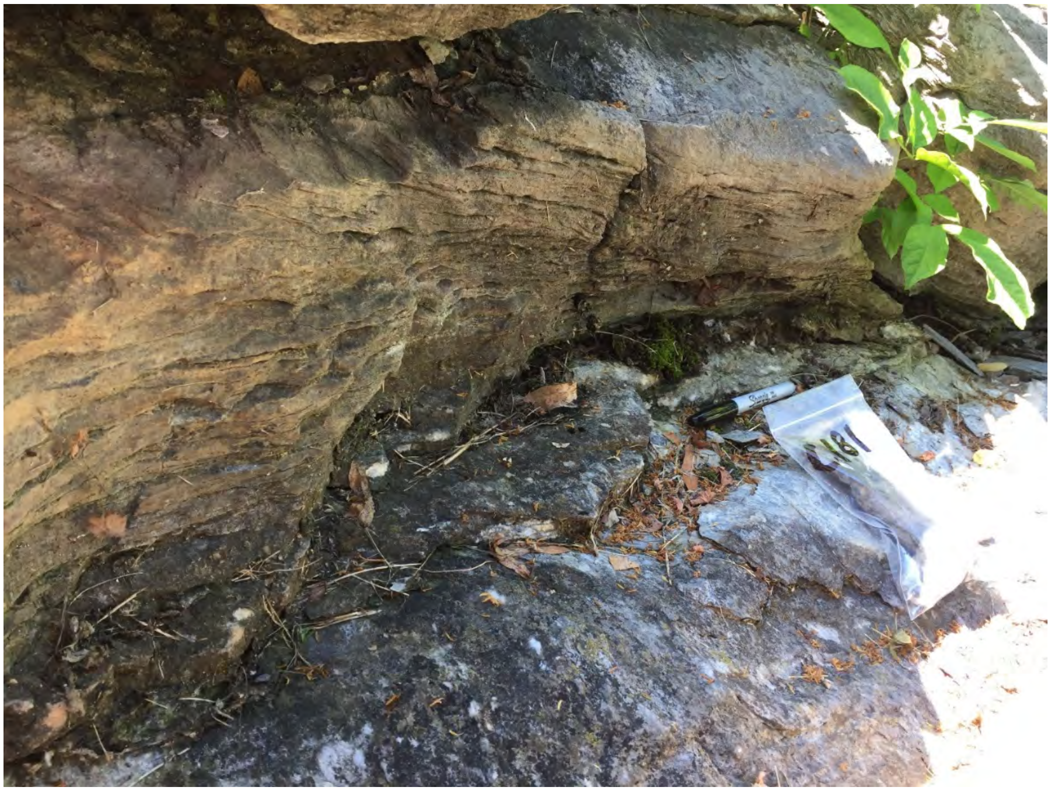

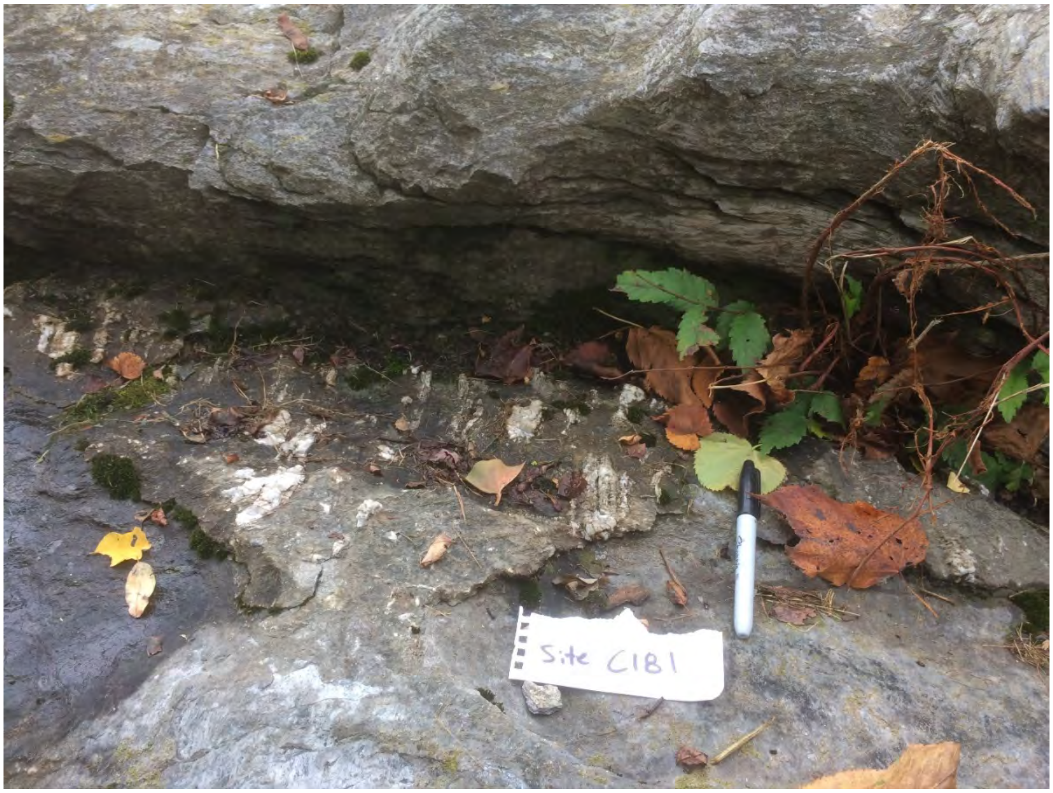

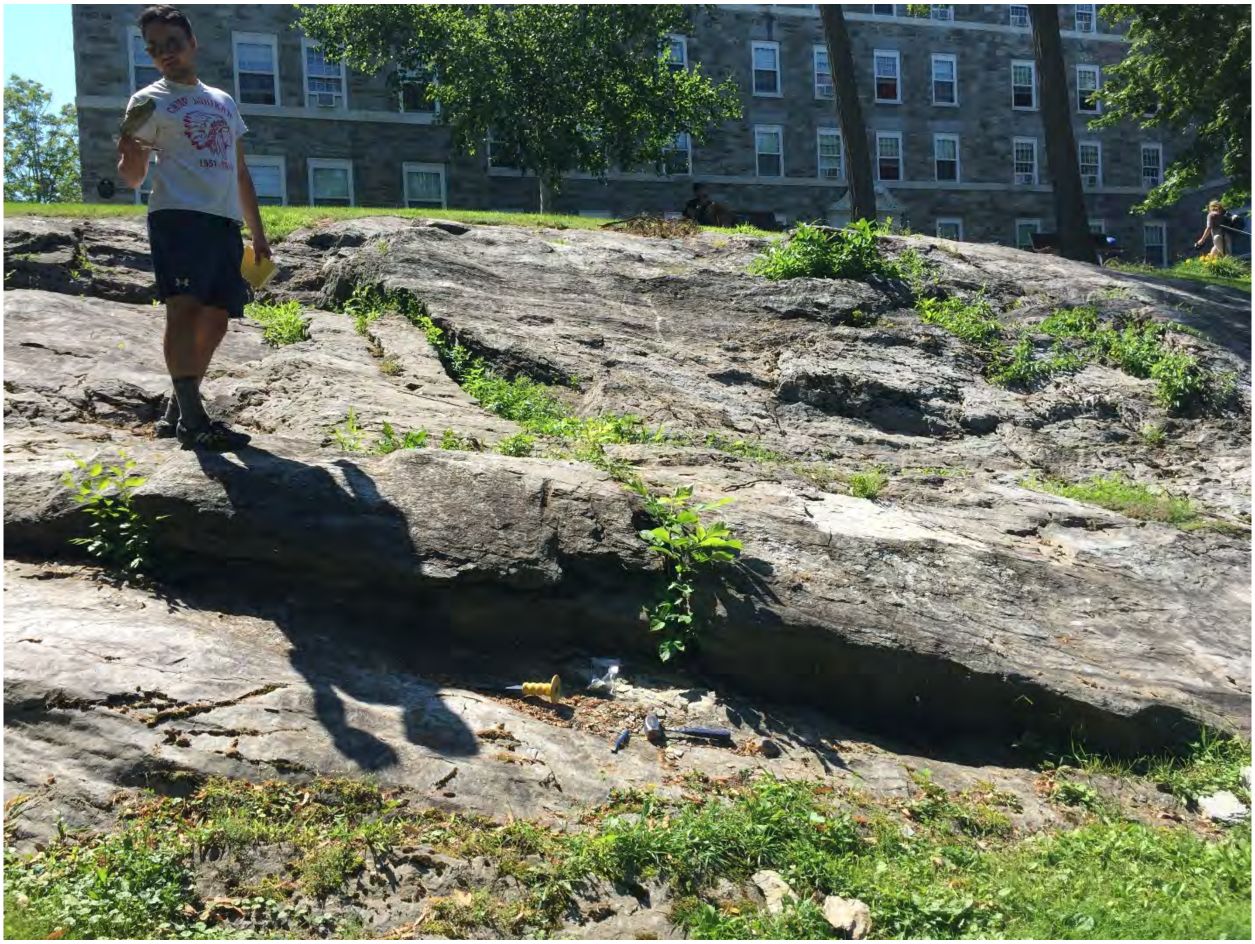

Lab Description: Circles denote areas targeted for dating, not actual laser shots.

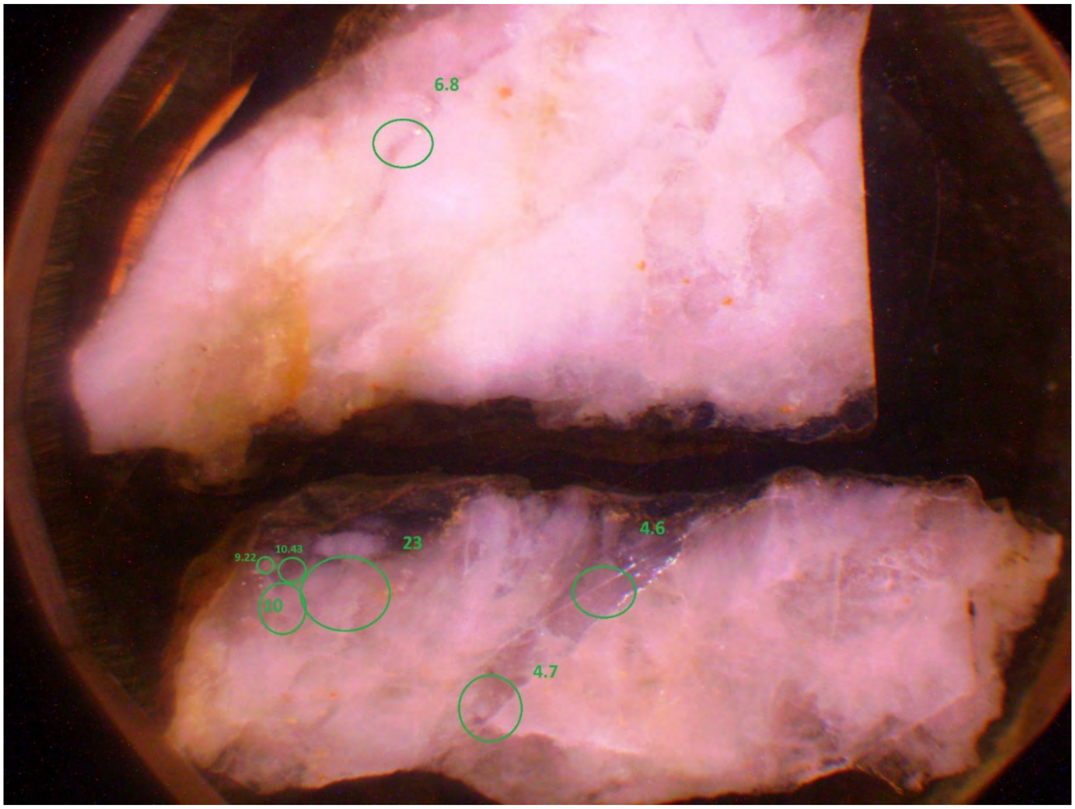

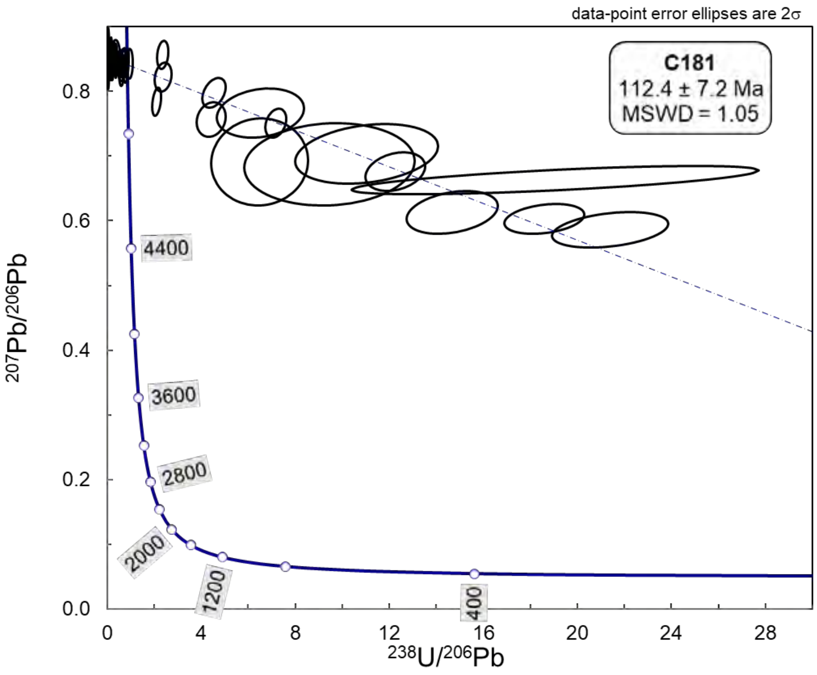

Supplement: Supplementary file 6 — Source Data [file 41467_2022_29680_MOESM6_ESM.zip › 1.zip!/appendix2_site_summaries_1to20/10_middlebury_campus_final.pdf]
