# Supplementary material for: Pace of passive margin tectonism revealed by U-Pb dating of fracture-filling calcite
Source: Nat Commun. 2022 Apr 12;13:1953. doi: 10.1038/s41467-022-29680-z (PMC9005700; doi:10.1038/s41467-022-29680-z)

11: Kingsland Bay East (44.243956, -73.299266)

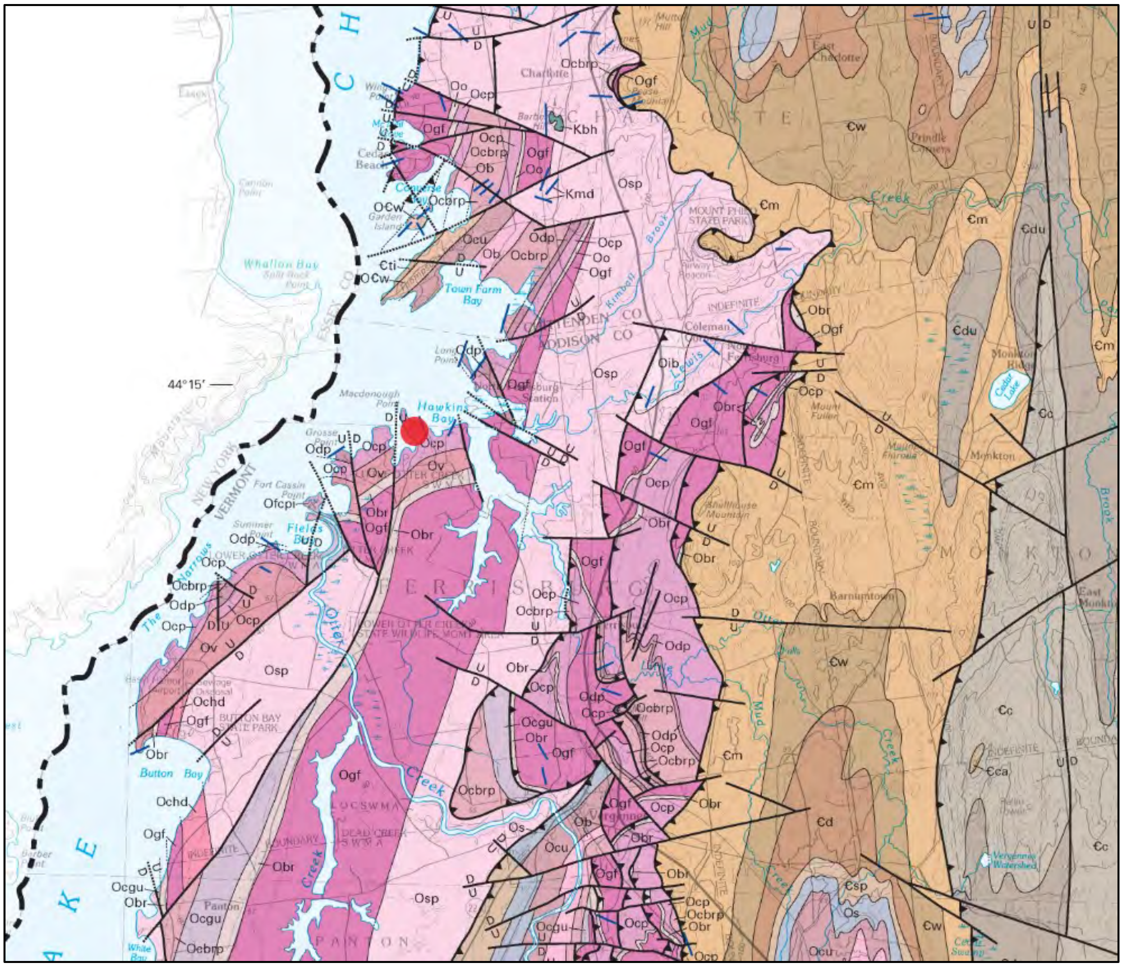

Figure 1: Sample location on state geologic map of Ratcliffe at al. (2011)

Number of failed samples from same site: none

Bedrock Unit: Crown Point Formation (Ocp)

Ages: **C198**: 112.2 ± 1.7

Summary: Site is located on the northeast side of Kingsland Bay on a west-facing cliff that plunges vertically into water. Best access by canoe from Ferrisburgh Town Beach. A thin Mode 1 vein with strike/dip 70°/82° S. Vein cuts bedding plane in a large fold, showing no evidence for slip or trans-tension along fracture. Interpreted to represent N-S extension at ca. 112 Ma.

<u>Field Observations</u>:

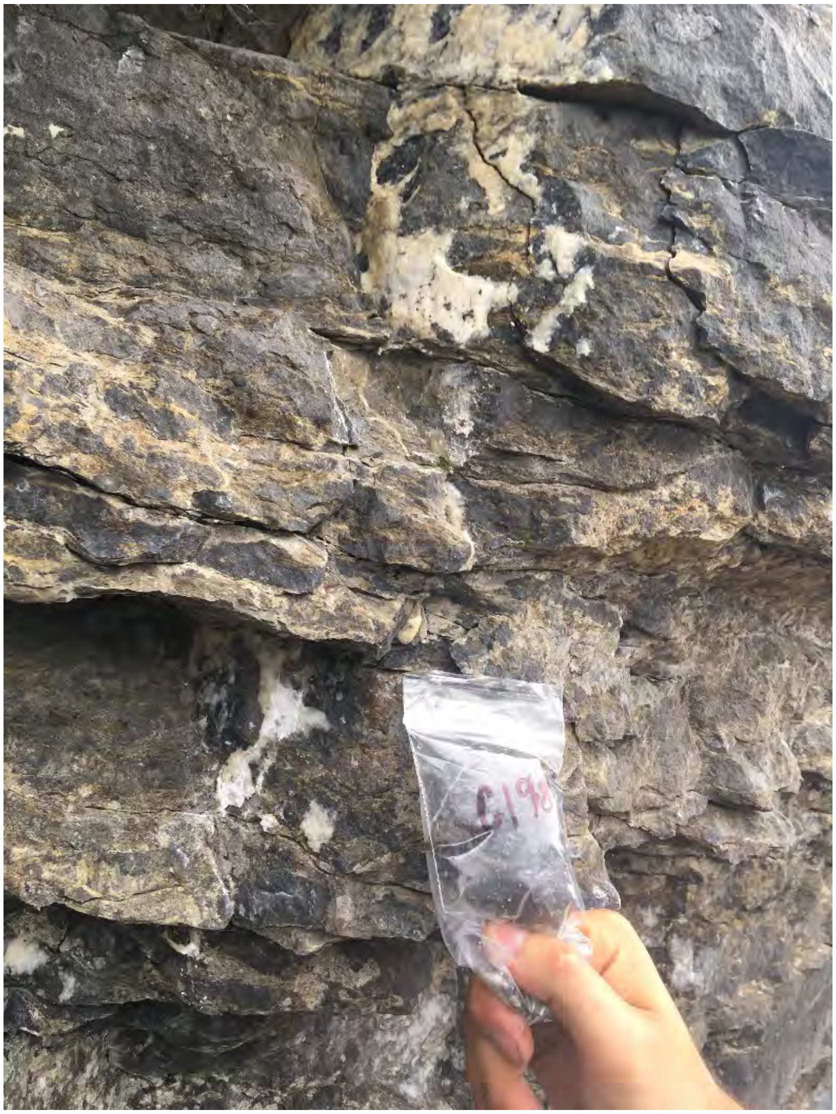

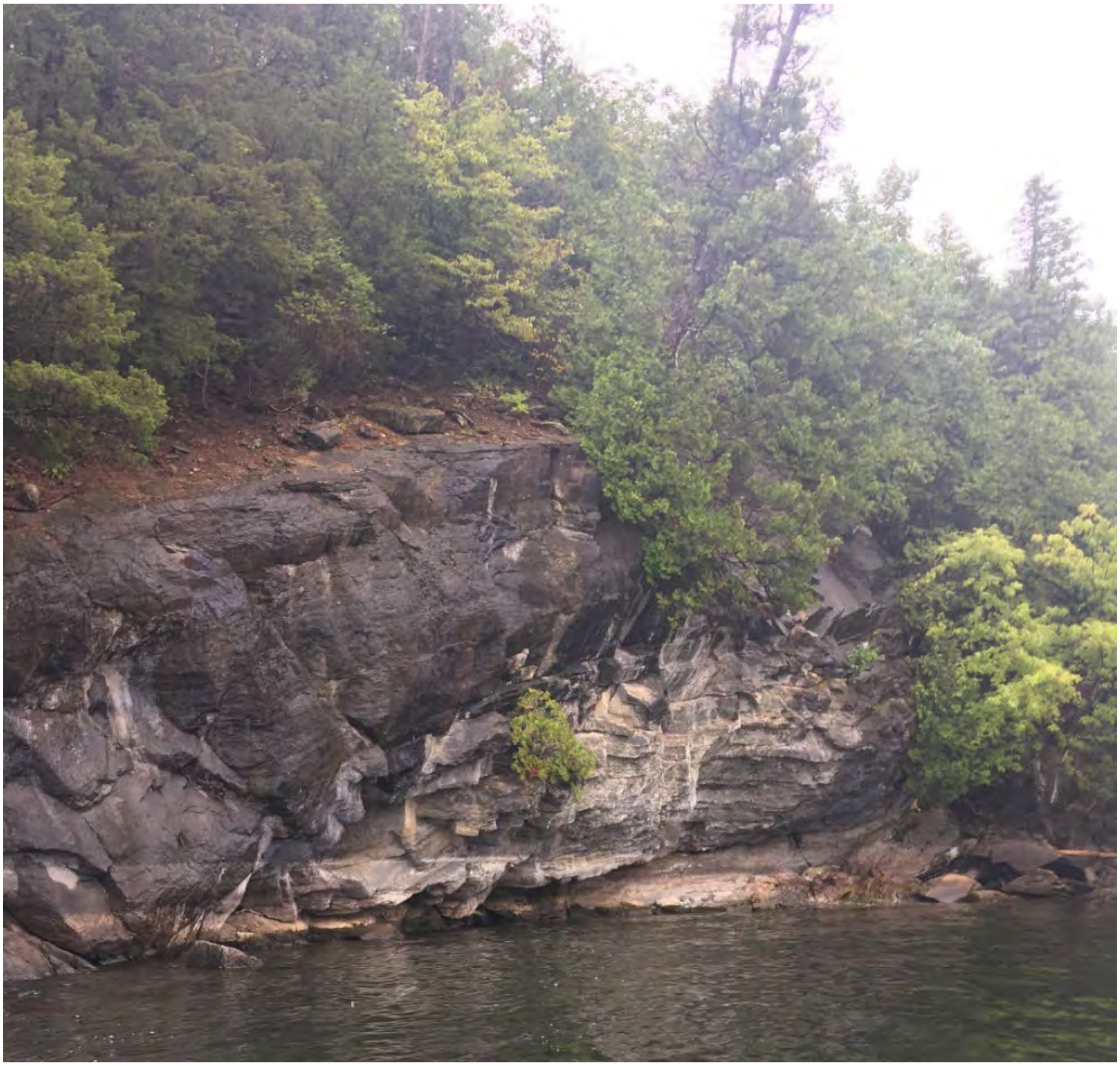

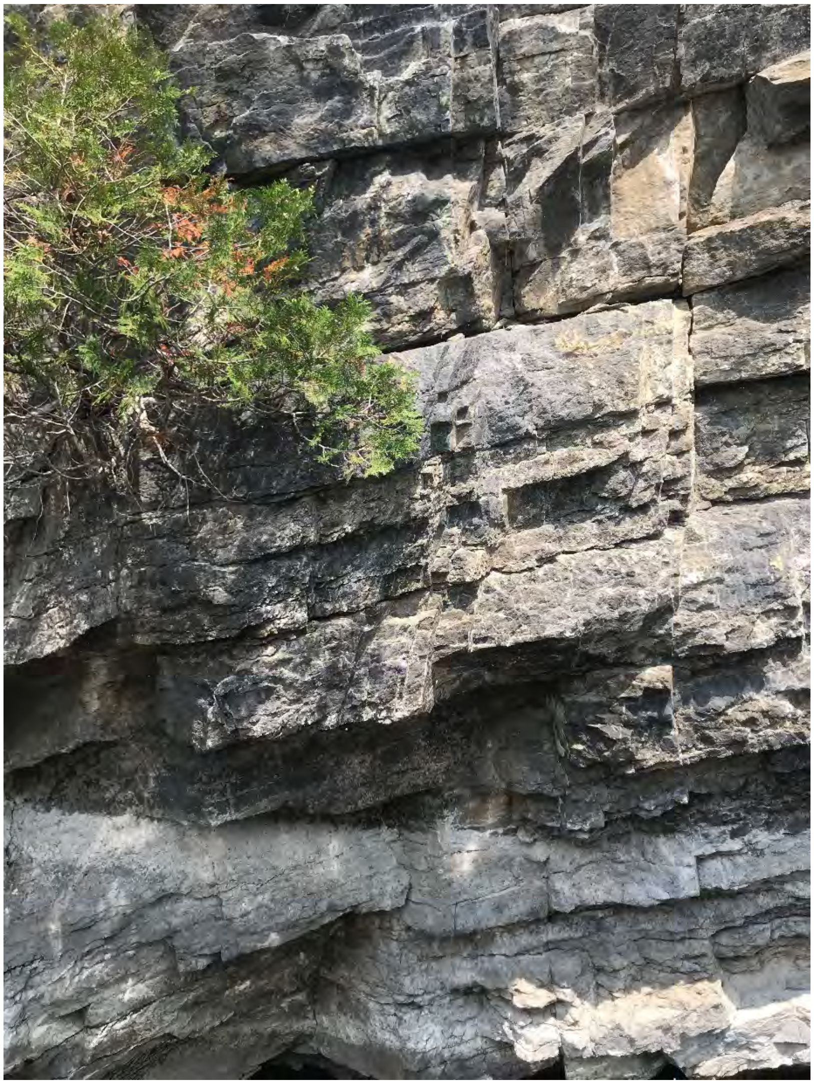

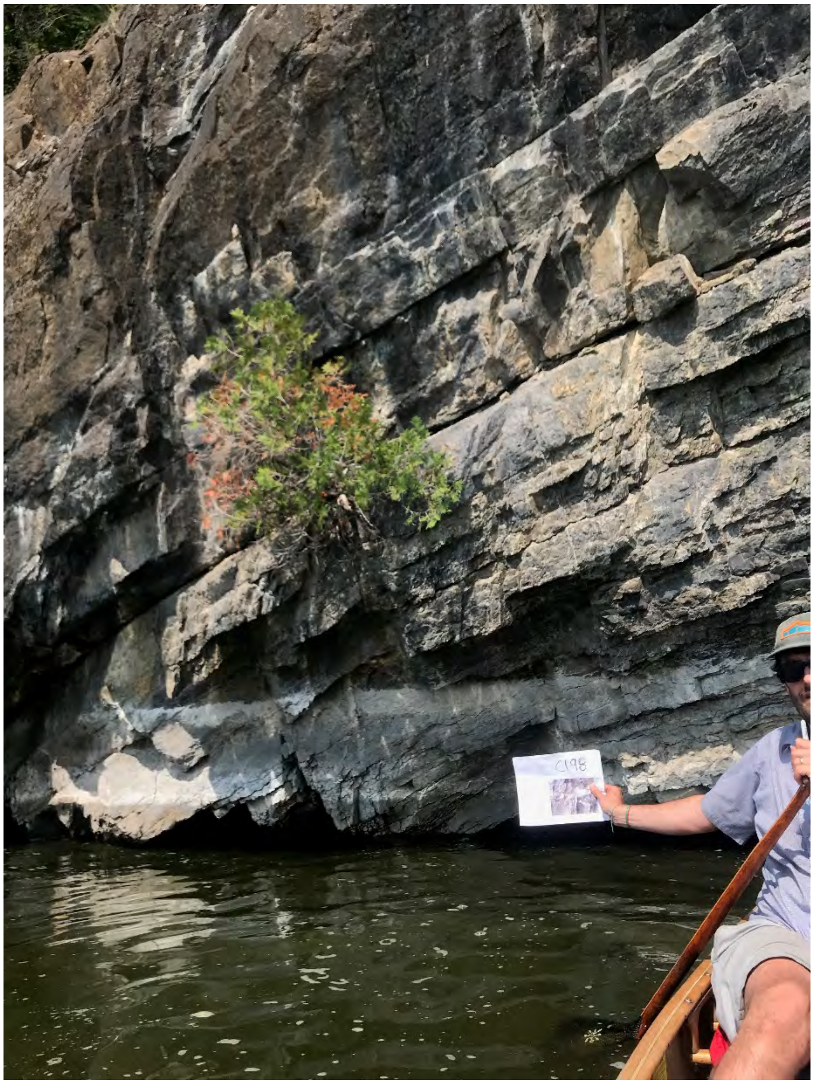
C198

Lab Description:

C198: Clearish, blocky calcite. Good ratios throughout and very well defined, single age domain across multiple fragments. Vein age appears to be representative of the vein.

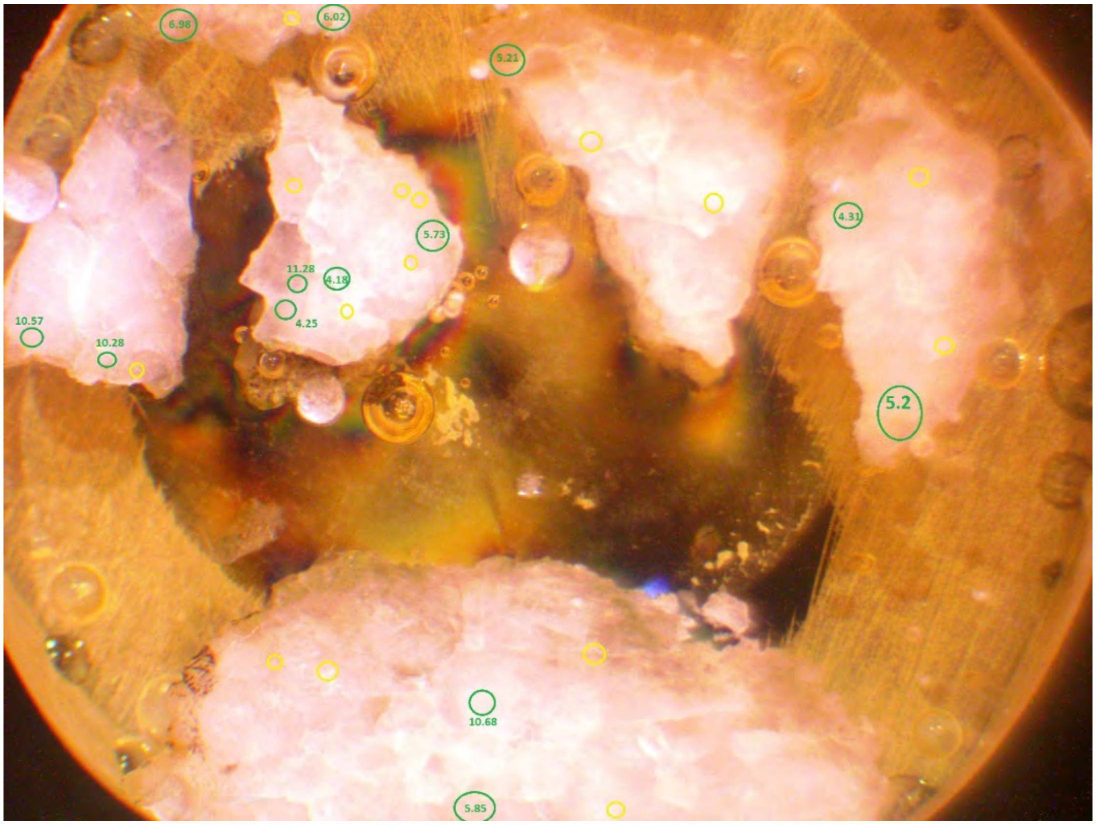

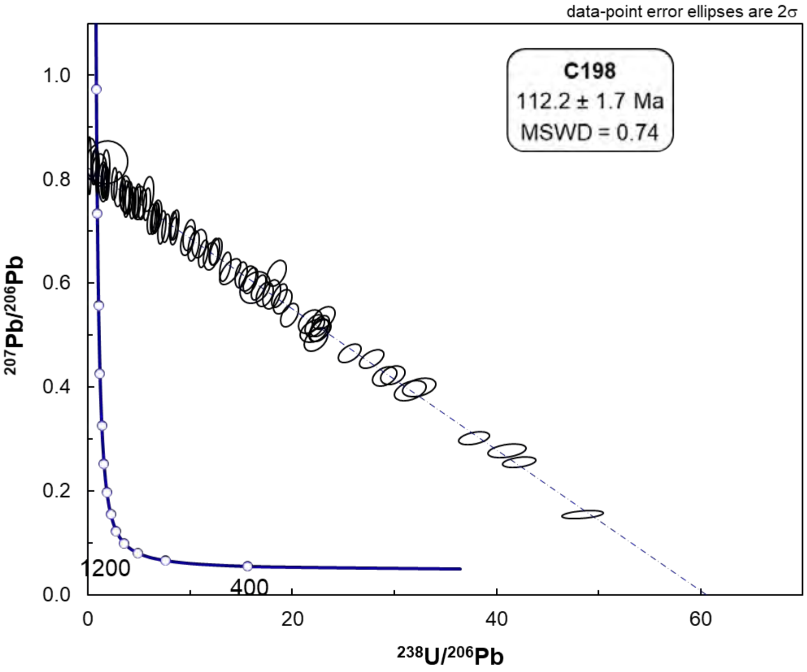

Supplement: Supplementary file 6 — Source Data [file 41467_2022_29680_MOESM6_ESM.zip › 1.zip!/appendix2_site_summaries_1to20/11_kingsland_bay_east_final.pdf]
