# Supplementary material for: Pace of passive margin tectonism revealed by U-Pb dating of fracture-filling calcite
Source: Nat Commun. 2022 Apr 12;13:1953. doi: 10.1038/s41467-022-29680-z (PMC9005700; doi:10.1038/s41467-022-29680-z)
Supplement: Supplementary file 6 — Source Data [file 41467_2022_29680_MOESM6_ESM.zip › 1.zip!/appendix2_site_summaries_1to20/12_double_dike_final.pdf]

12: Double Dikes; Charlotte, VT (44.318368, -73.295103)

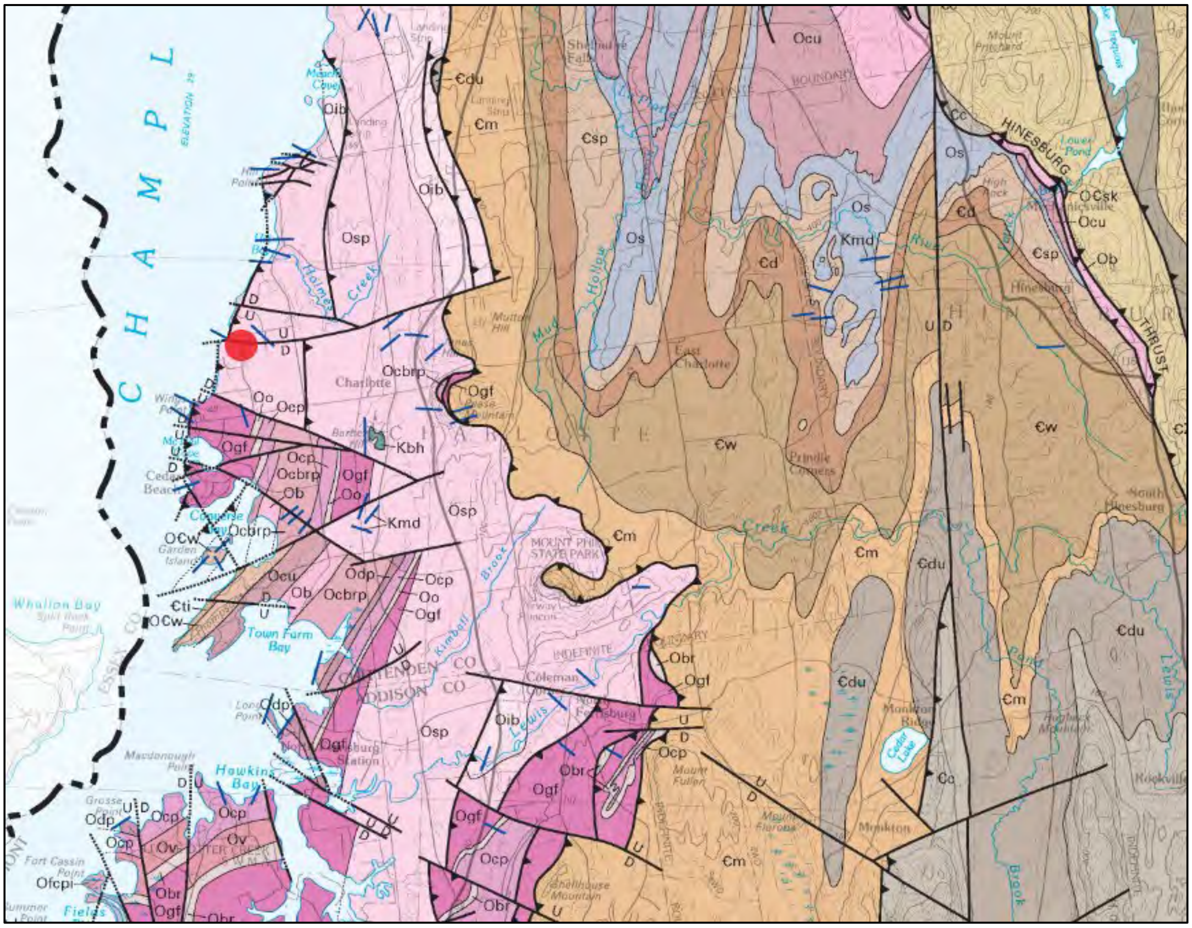

Figure 1: Sample location on state geologic map of Ratcliffe at al. (2011)

Failed samples from same site: C221, 220B,C,D,F,G

Bedrock Unit: Stony Point Formation (Osp) + Lamprophyre dike

Vein Ages: **C220**: 81.4 ± 2.6 Ma; **C220B**: 114 ± 10 **C220H**: 133 ± 12 and 3.4 ± 4.7 Ma

Dike Ages: **D61A (lamprophyre)**: 130.3 ± 0.6 Ma

Summary: Minor faults associated with a pair of large (> 2m wide) dikes exposed along the lakeshore roughly 2 miles south of Charlotte Beach. Access is possible by boat, or by permission of landowner Alex Kroll (alexkroll@gmavt.net). One lamprophyre dike has a strike/dip of 255°/60° N whereas an adjacent trachyte dike is oriented 104°/90°N. Several events are recorded at this site by both the dikes and associated faults. First, vein C220H is a mode 1 vein that is orthogonal to the lamprophyre dike and entirely confined within it. It likely records thermal contraction of the dike shortly after emplacement at ~130 Ma. Second, sample 220B is a mode 1 fracture striking roughly E-W interpreted to record N-S σ_3 at ~114 Ma. Finally, C220 is top-to-the-north extensional fault that records N-S extension along a small normal fault zone between the two dikes at ~81 Ma. The timing and kinematics of the 81 Ma event agree very well with the same event observed at Hills Point (sites 13, 14, and 41) roughly 2 miles to the

north. Finally, a secondary crystallization event is recorded by sample C220H at ~3.4 Ma.   It is thought to be secondary because young U-Pb shots came from a discolored lateral margin of the vein visible in outcrop and thick section the U-Pb isochron shows distinct age domains.

Field Observations:

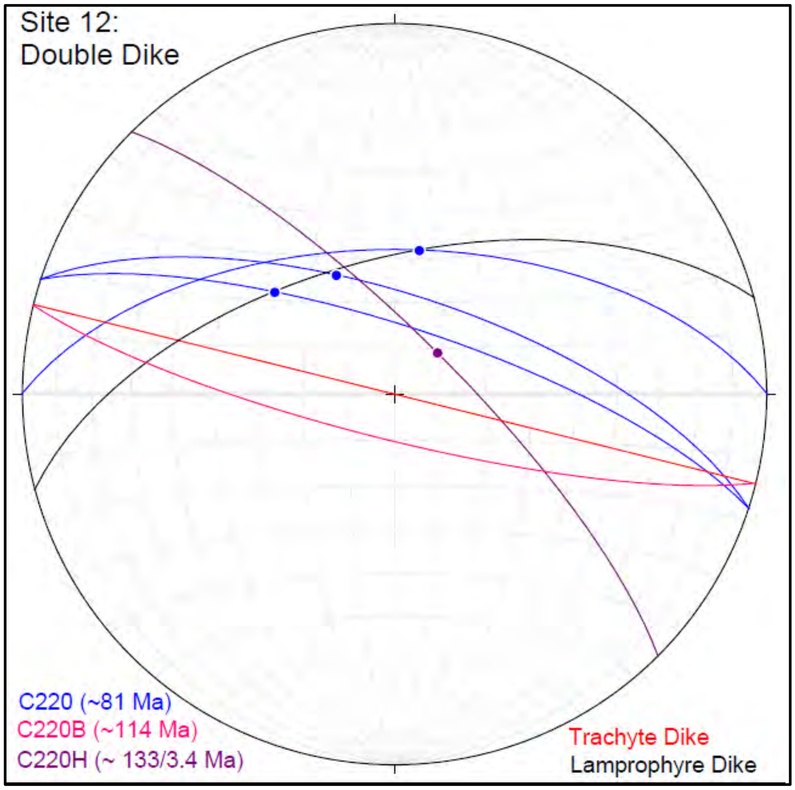

C220:

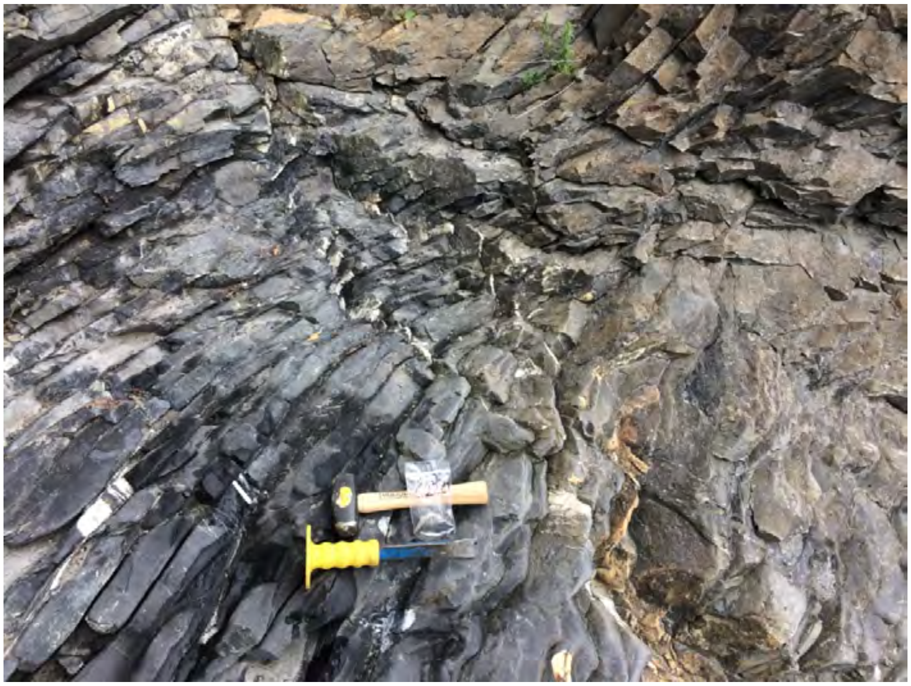

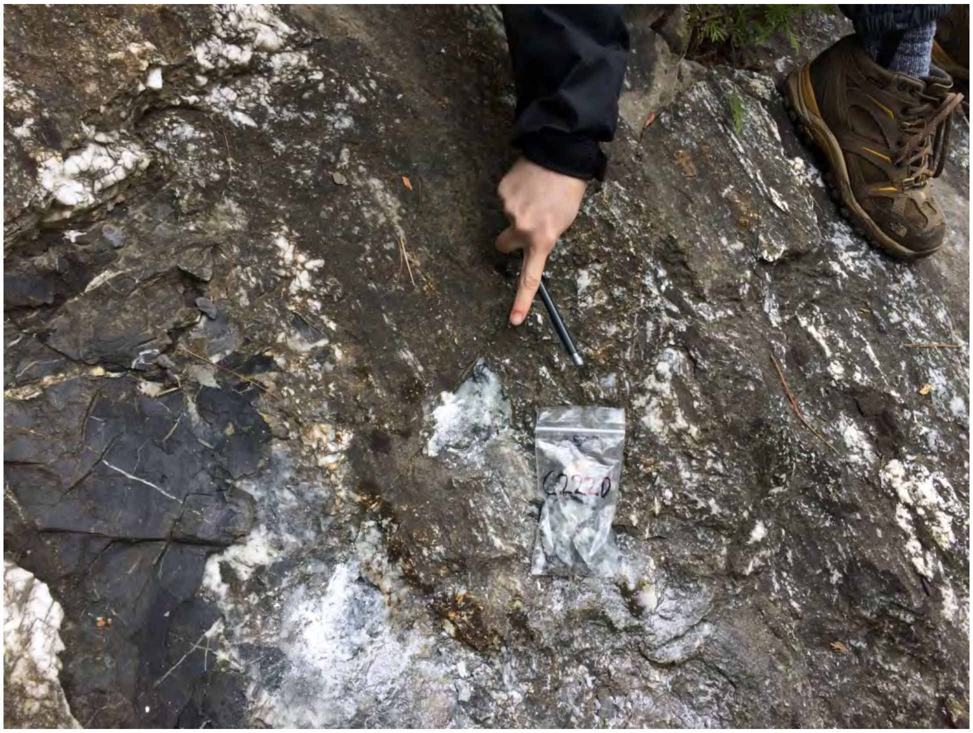

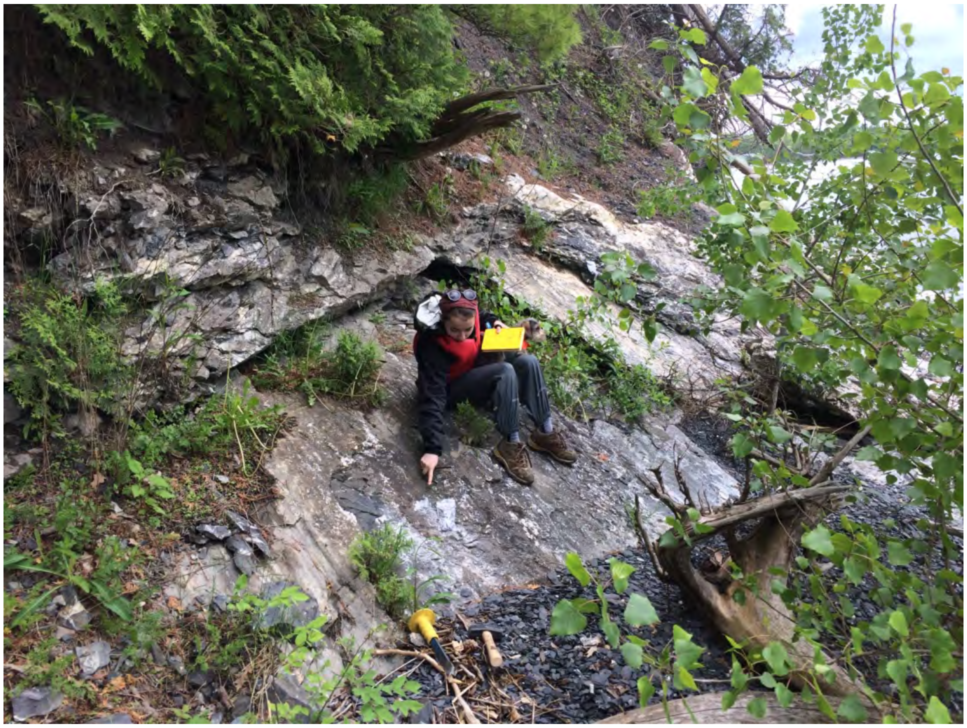

C220B:

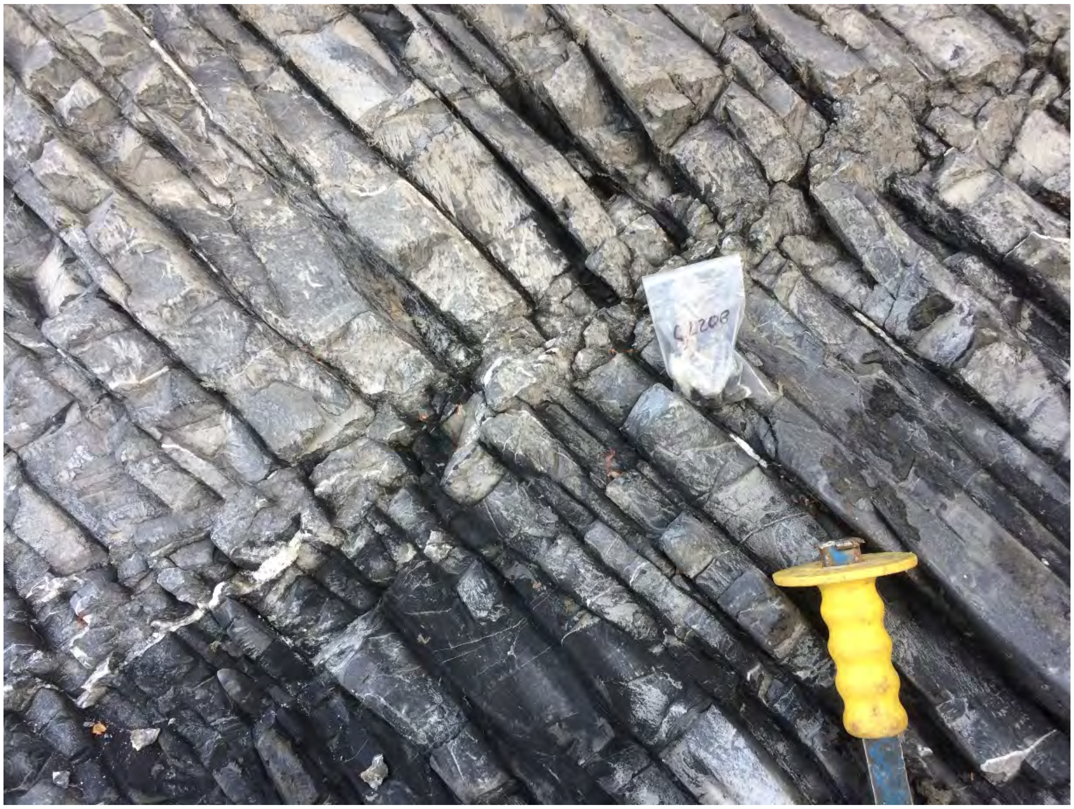

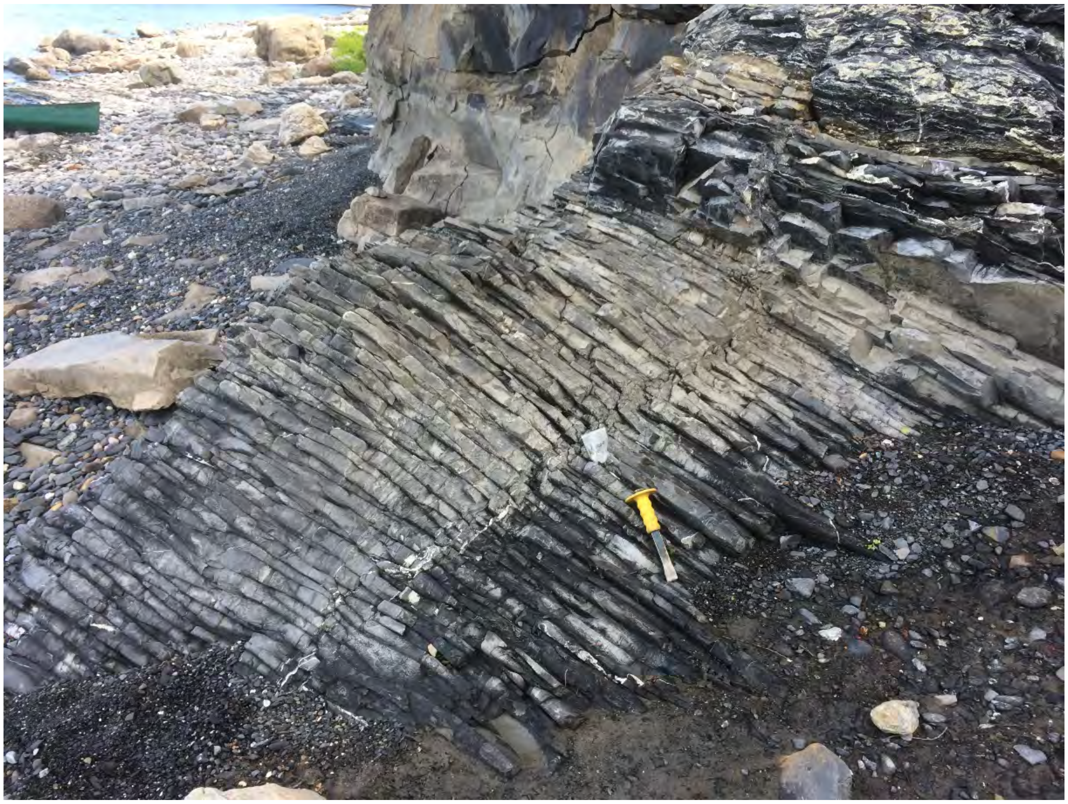

C220H:

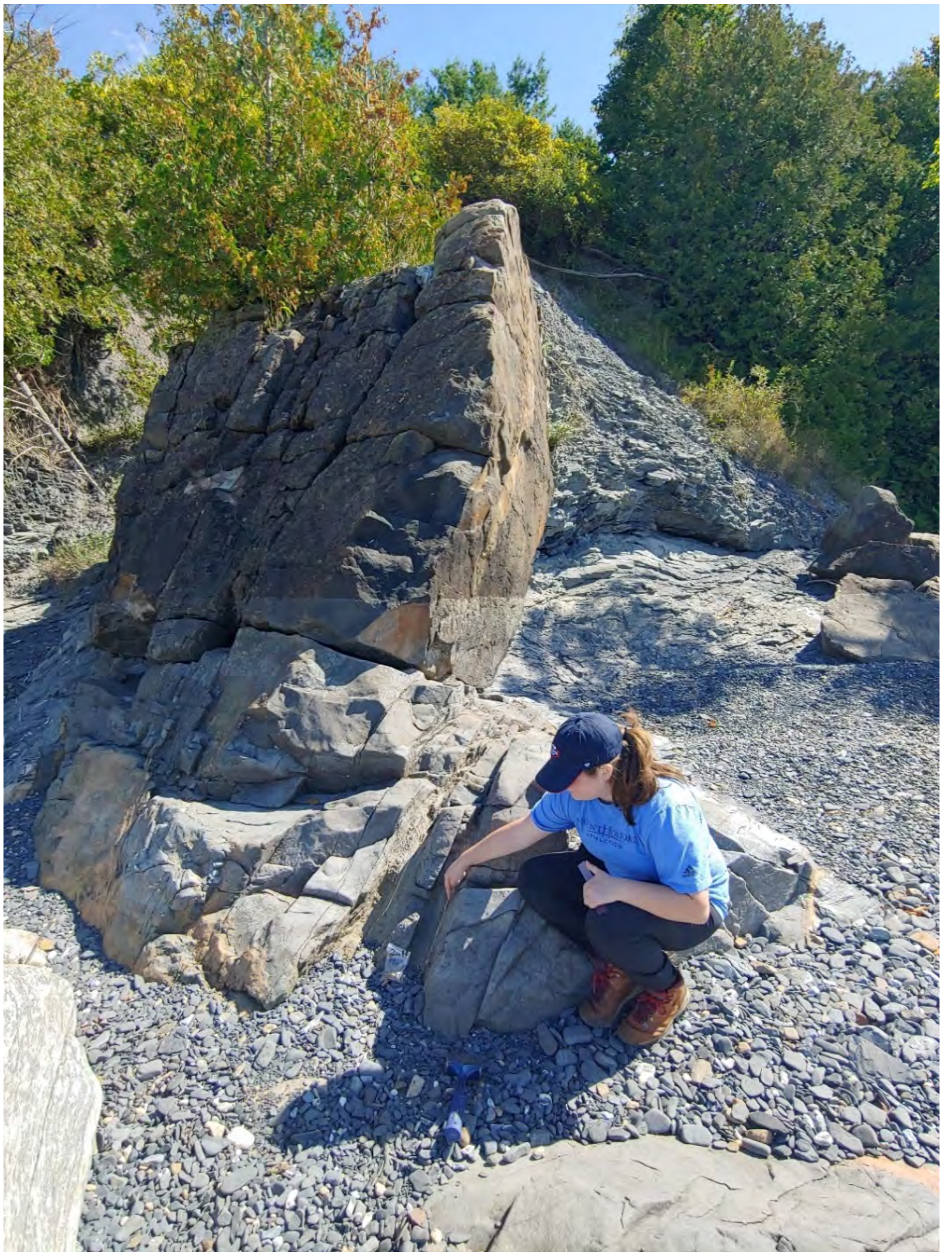

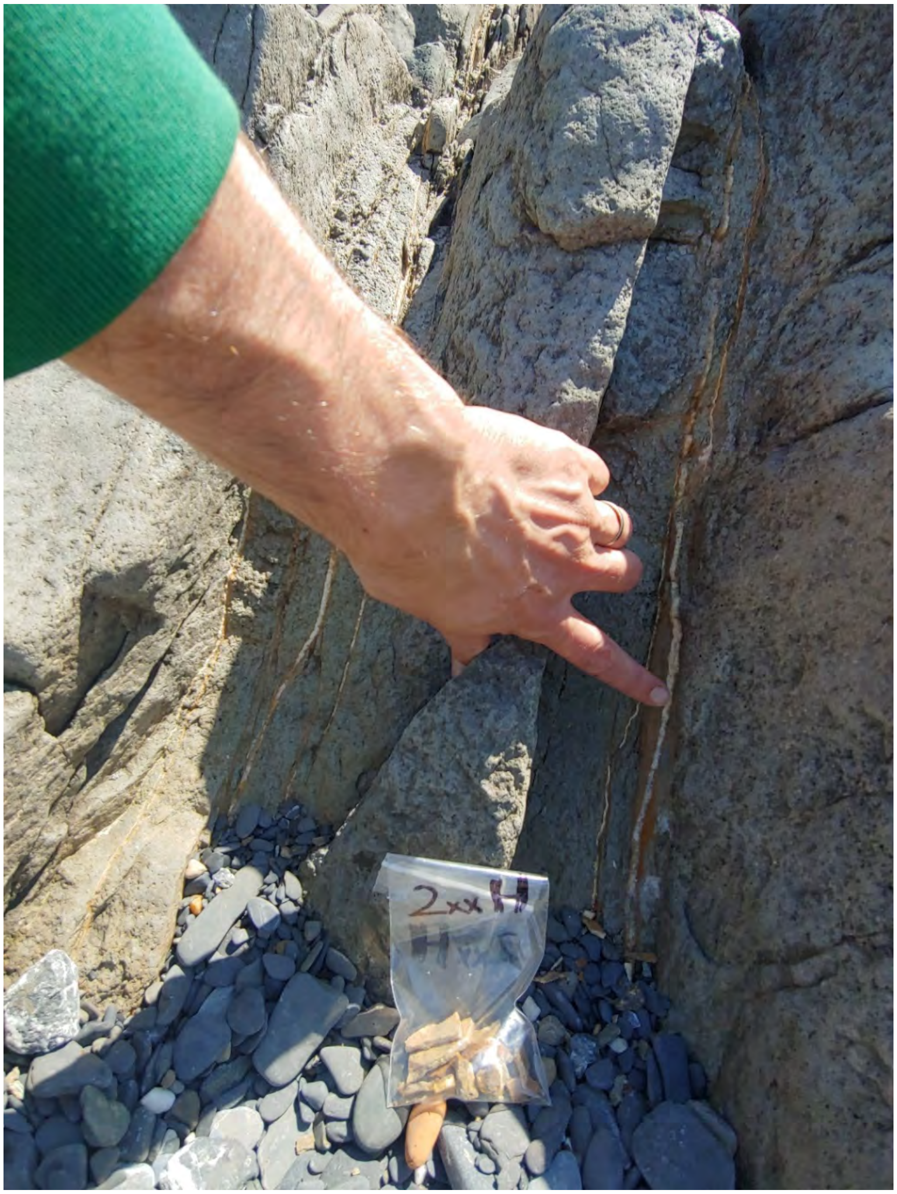
Note the red staining on edges of vein 220H, a recrystallization even that dates to roughly 3.4 Ma

Lab Description:

C220: analyzed at UCSB in June 2018.

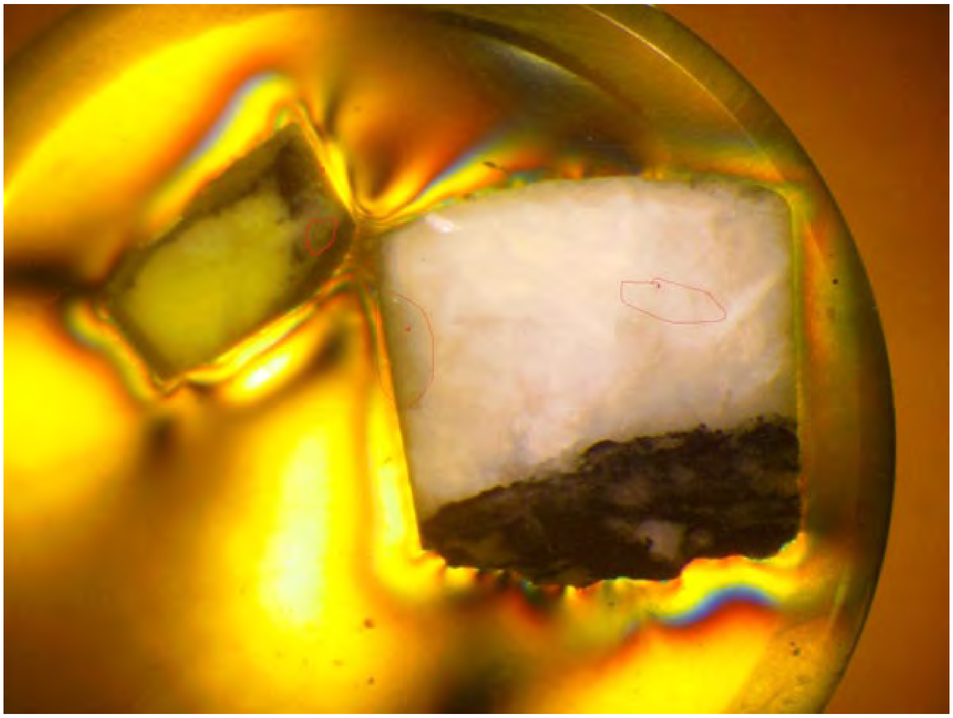

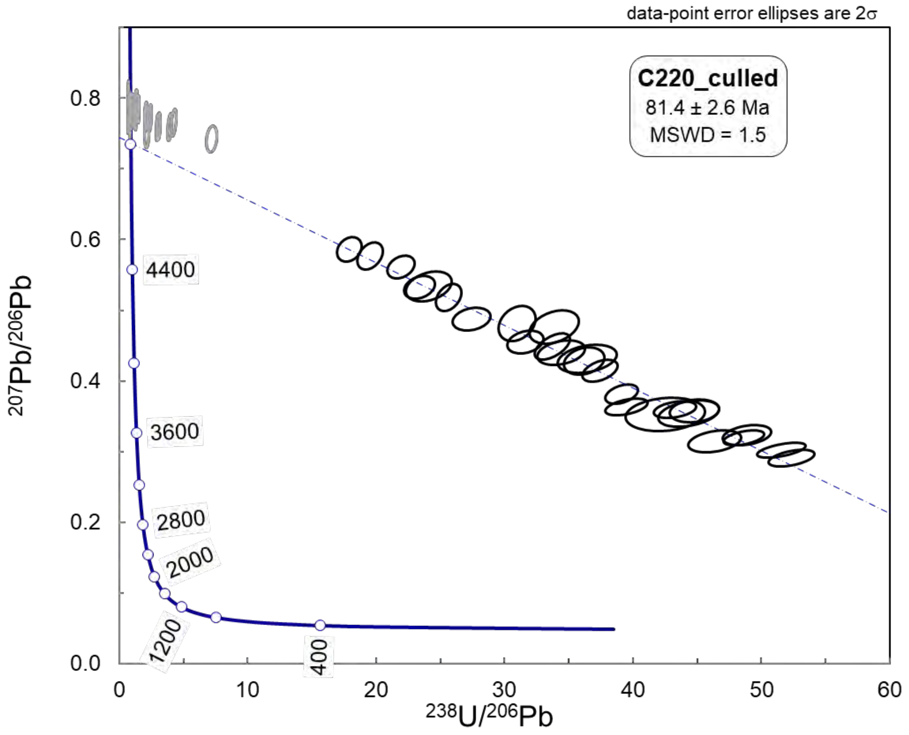

C220B: analyzed at UCSB in January, 2021.

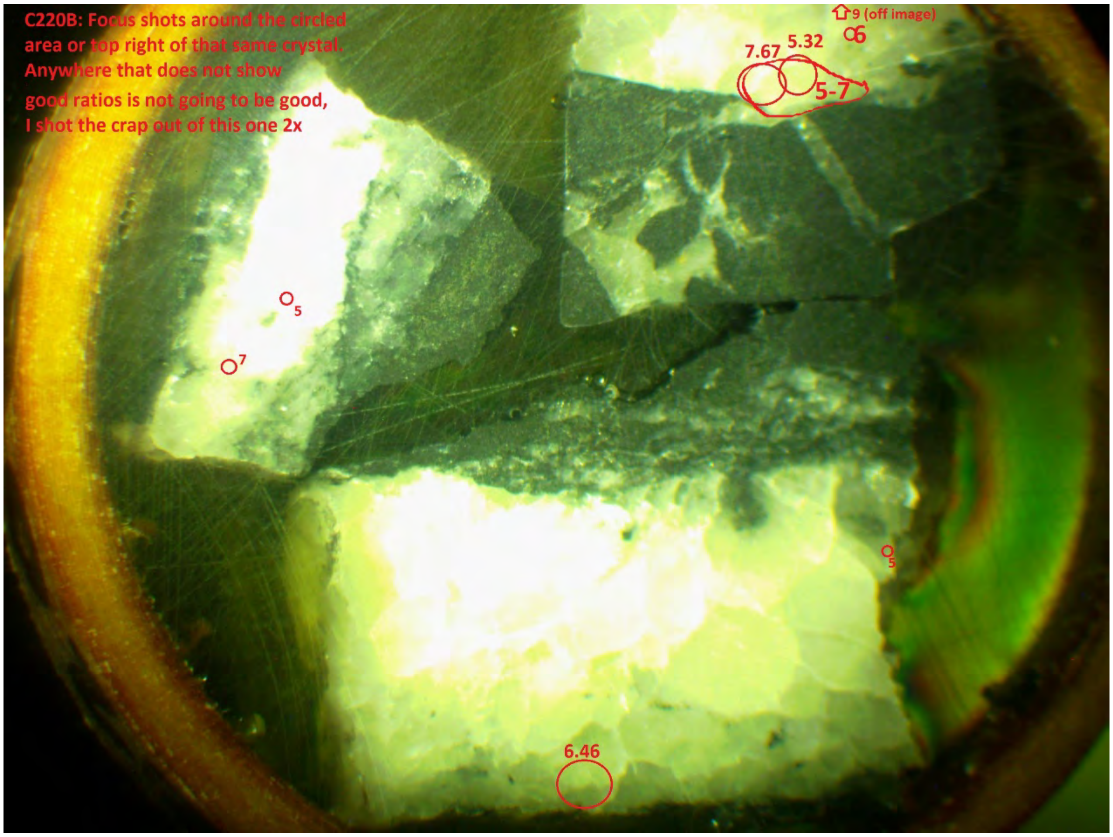

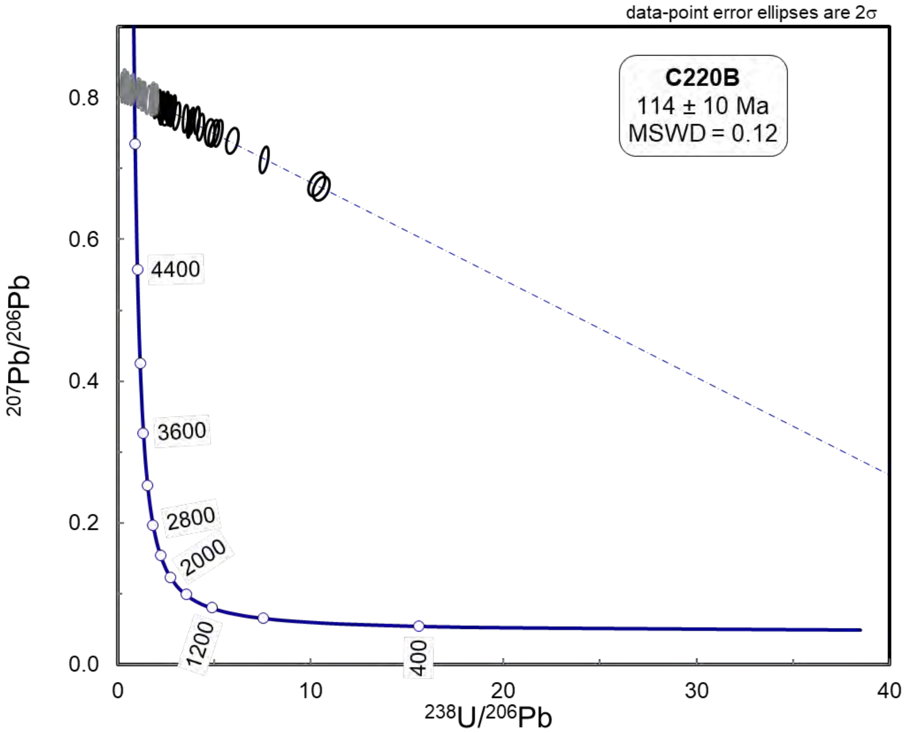

C220H: Analyzed at UCSB April, 2019. Note discolored edges of the vein representing a Pliocene recrystallization event

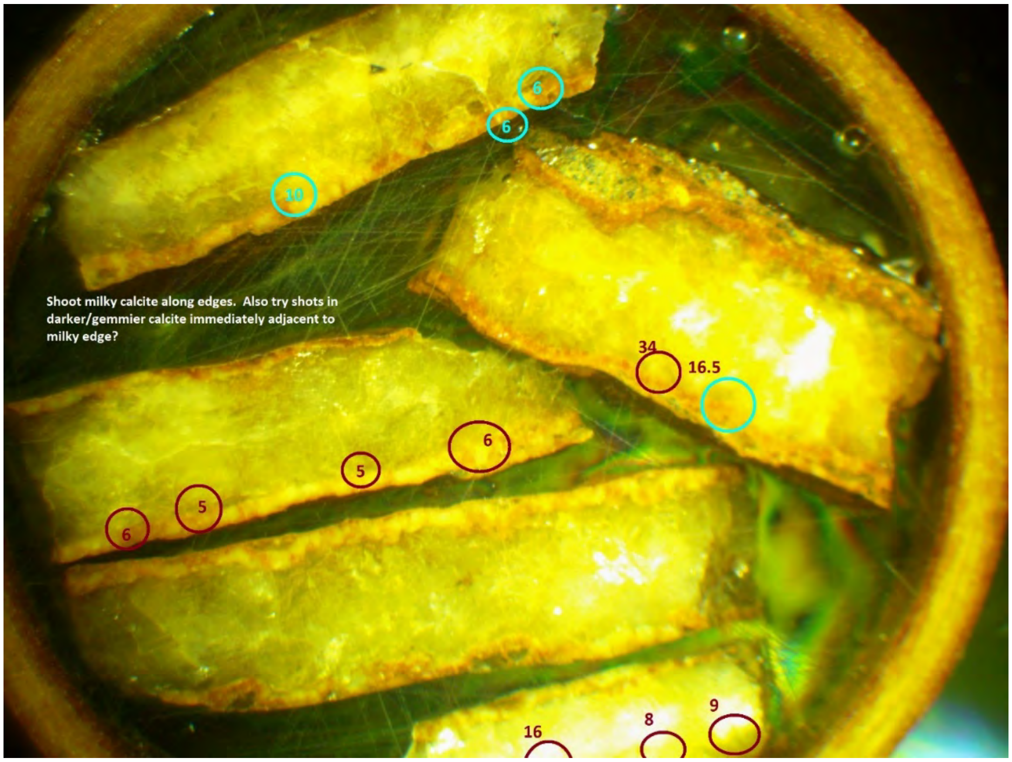

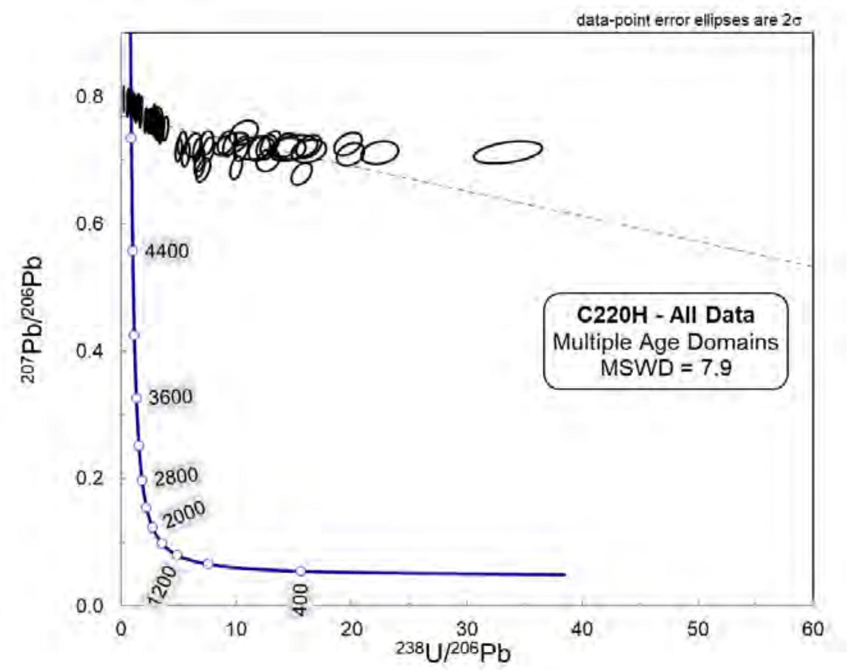

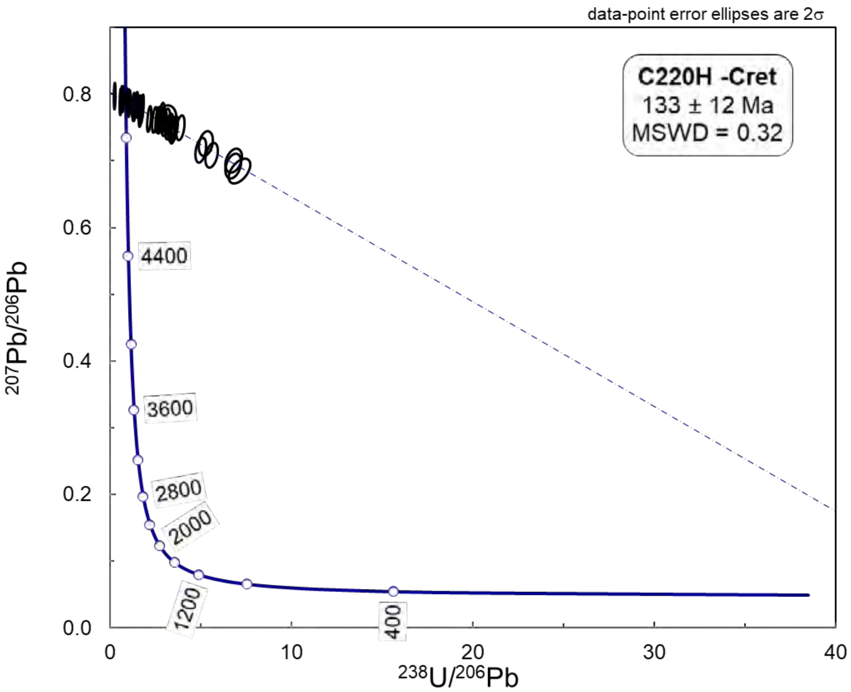
data-point error ellipses are 2σ
C220H -Cret
133 ± 12 Ma
MSWD = 0.32
0.8
0.6
0.4
0.2
0.0
4400
3600
2800
2000
1200
400
0
10
20
30
40
207Pb/206Pb
238U/206Pb

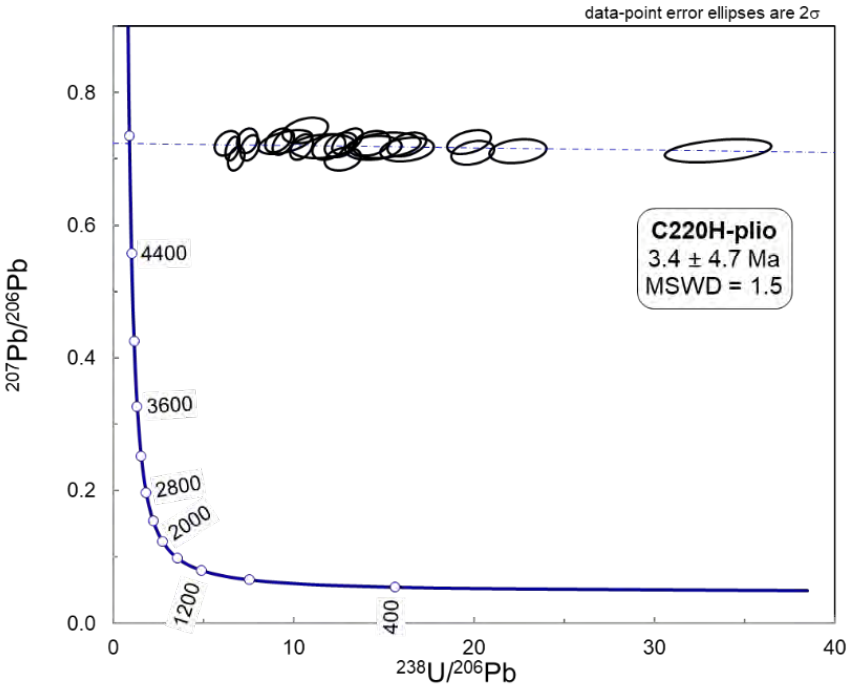
data-point error ellipses are 2σ
C220H-plio
3.4 ± 4.7 Ma
MSWD = 1.5
0.8
0.6
0.4
0.2
0.0
4400
3600
2800
2000
1200
400
0
10
20
30
40
207Pb/206Pb
238U/206Pb
