# Supplementary material for: Pace of passive margin tectonism revealed by U-Pb dating of fracture-filling calcite
Source: Nat Commun. 2022 Apr 12;13:1953. doi: 10.1038/s41467-022-29680-z (PMC9005700; doi:10.1038/s41467-022-29680-z)
Supplement: Supplementary file 6 — Source Data [file 41467_2022_29680_MOESM6_ESM.zip › 1.zip!/appendix2_site_summaries_1to20/13_Hills Point_south_final.pdf]

13: Hills Point South (44.34574, -73.28471)

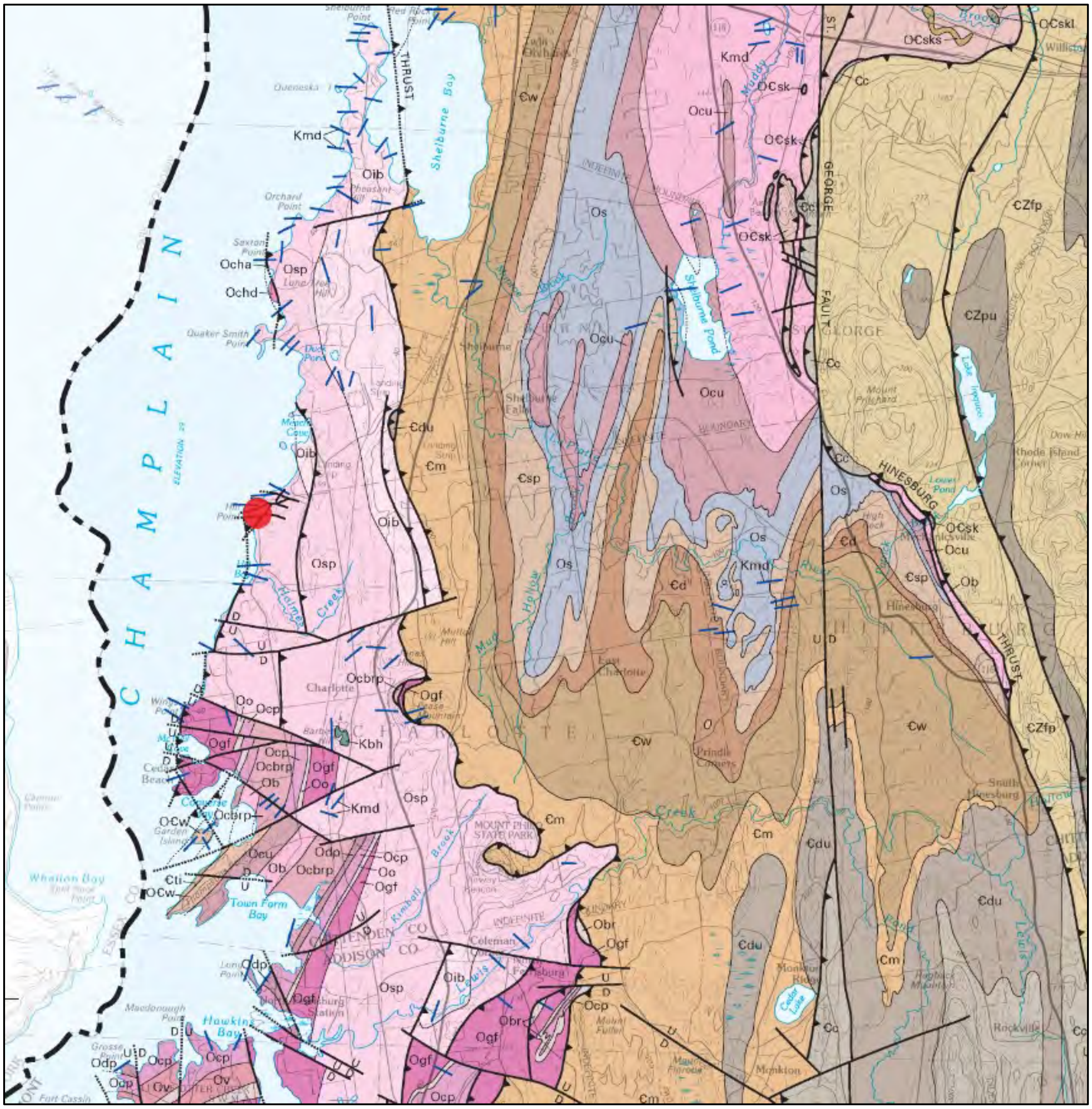

Figure 1: Sample location on state geologic map of Ratcliffe at al. (2011)

<u>Failed samples from nearby sites:</u> C229E, C230F, C231E,F,G; C232, C233, C234

<u>Bedrock Unit:</u> Stony Point Formation (Osp)

<u>Ages</u>: **C229**: 95.9 ± 0.9; **C229B**: 86.4 ± 0.8; **C229C**: 90.3 ± 2.0 **&** 79.0 ± 0.55; **C229D**: 90.8 ± 0.8; **C229F**: 114.7 ± 2.6 Ma

Summary: Fault zone exposed along shores of Lake Champlain on Hills Point in Charlotte. Roughly 30 m south of site 41 and 100 m south of site 14. Best access by canoe from Charlotte Beach or via land owner stairs for beach access. Complex multi-generational fault zone strikes ~60°, dipping ~45° SE. Fault zone cross-cuts an older (~115 Ma) set of vertical mode 1 veins (C229F). Both the ages and field evidence demonstrate that the fault has experienced multiple generations of slip, for example offsetting older 'tabs' of calcite against one another within the fault zone. Likewise, this site and the other nearby sites (41 and 14) returned a large number of different U-Pb ages, suggesting the fault zone was actively accommodating stress in a variety of different tectonic regimes.

The ~114 Ma event (C230F) is a network of vertical veins striking 90 degrees and cross-cut by the fault zone. This vein is interpreted to record a N-S sigma-3 stress.

The 96 Ma event (C229) is from the main pocket of calcite spar within the fault zone and seems to record the opening of significant space within the fault zone by extension and filling with fluids (see photos below). Although no fabrics could be directly linked to this pocket of spar, opening of a large void space suggests a TTS direction of extension and N-S sigma-3.

The ~90 Ma event (C229C and D) is seen in two blocks of tabular calcite from the upper part of the fault zone. Slickensides and growth striae suggest these calcite tabs formed under NW-SE extension (see figure 2 below). Although we were not able to infer sense of slip from these fabrics, TTSE extension is inferred from the observation that significant void space was required to crystallize calcite.

A later 86 Ma event (C229B) affected the fault zone although we were unable to observe growth striae or slickensides associated with this sample. Given the thin laminar nature of this calcite deposit we are not confident it can be assigned to extension and thus classify it as 'fault with unknown slip sense.'

Field Observations:

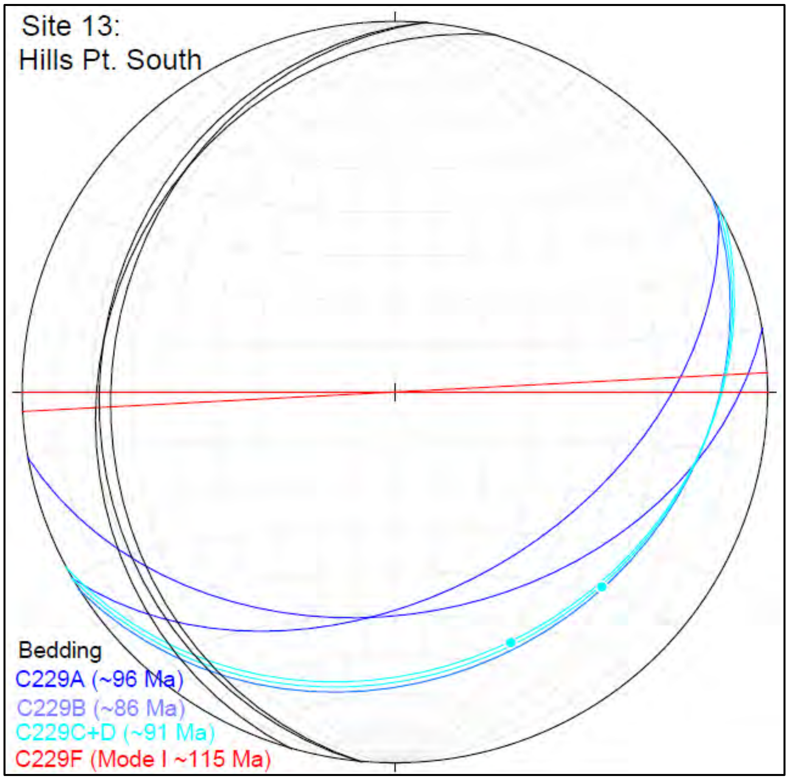

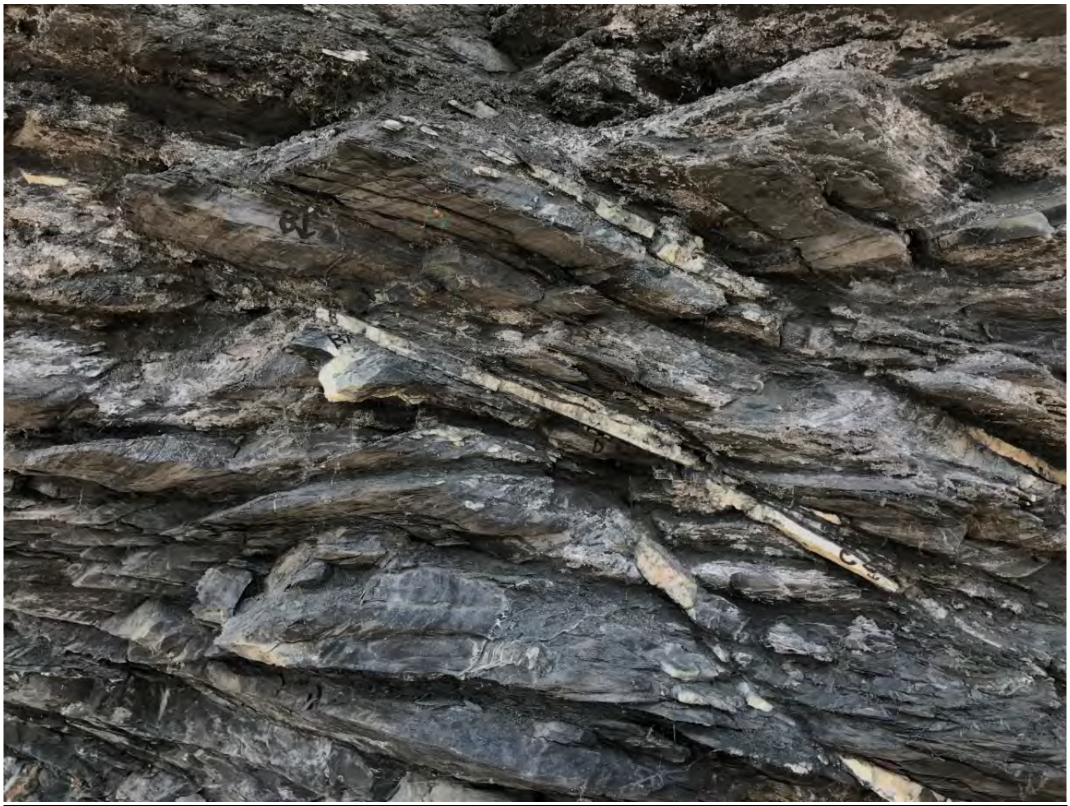

C229A:

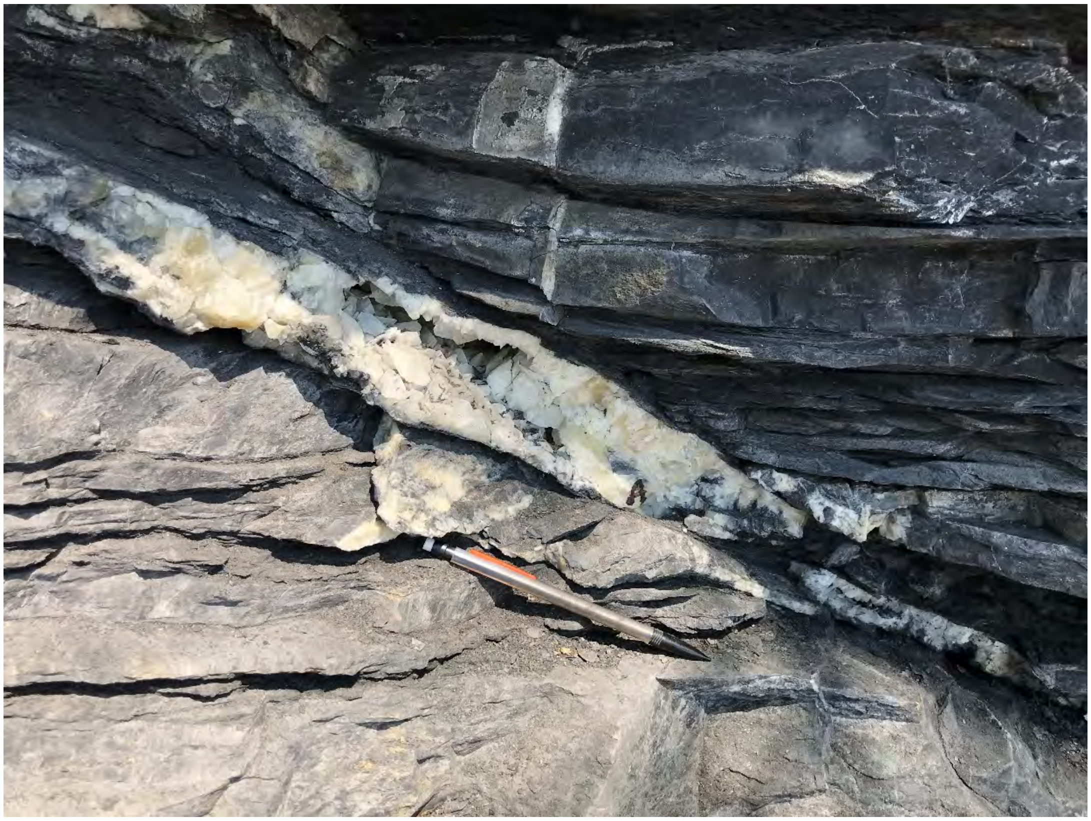

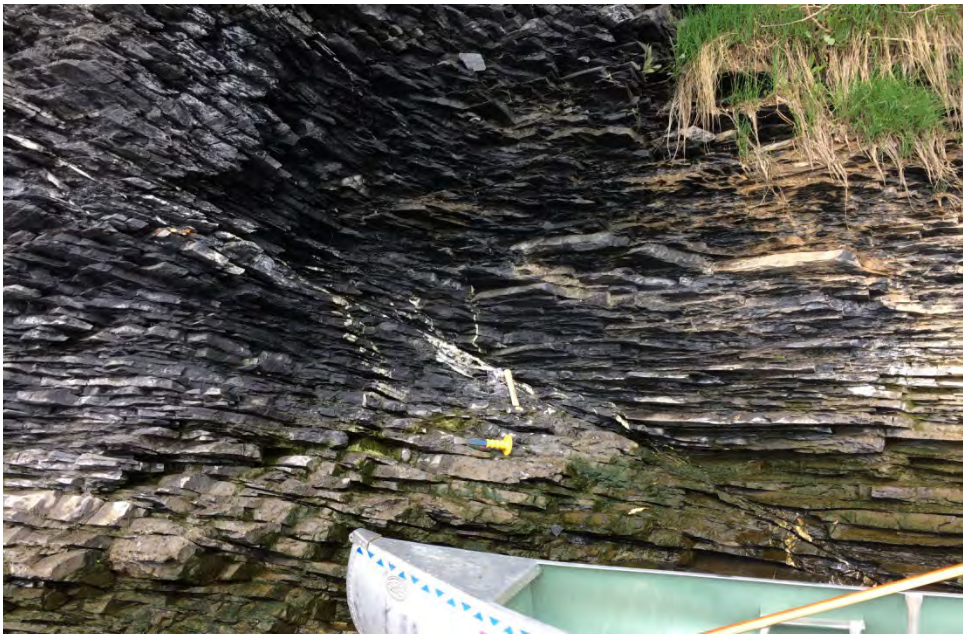

C229B:

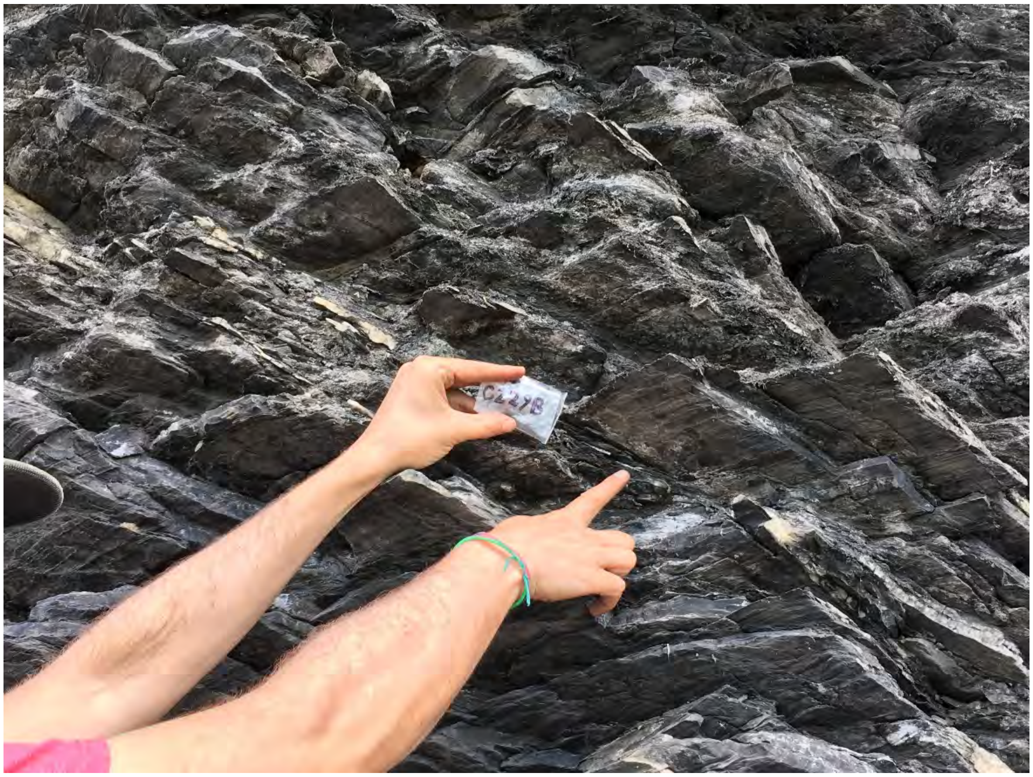

C229C:

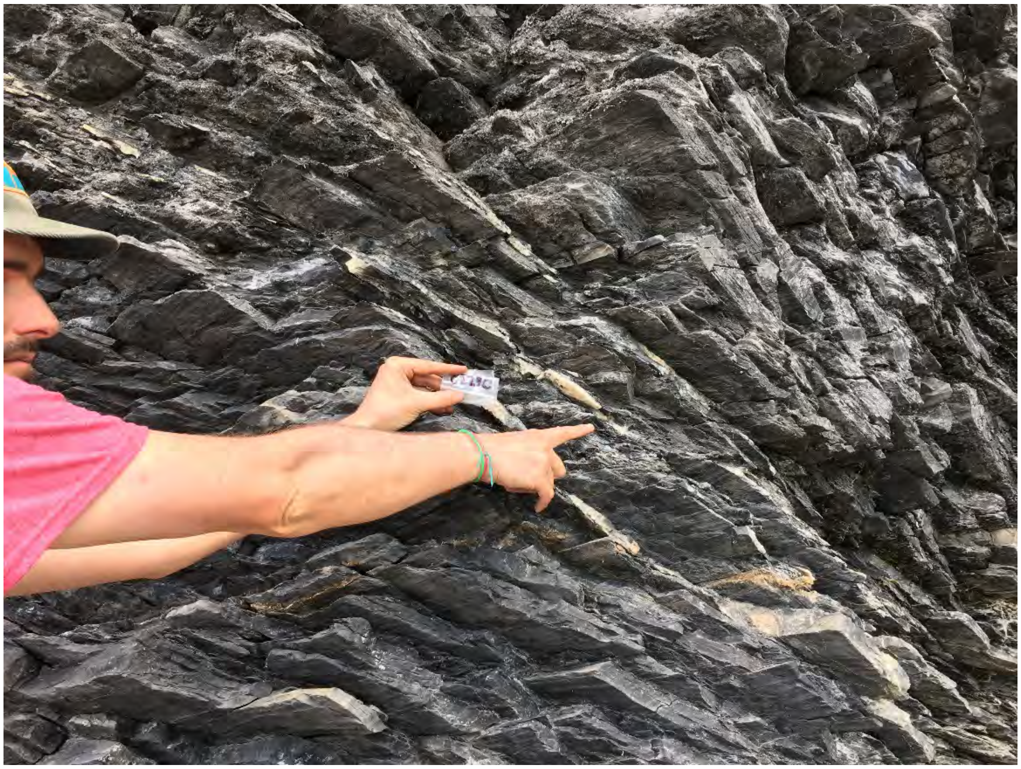

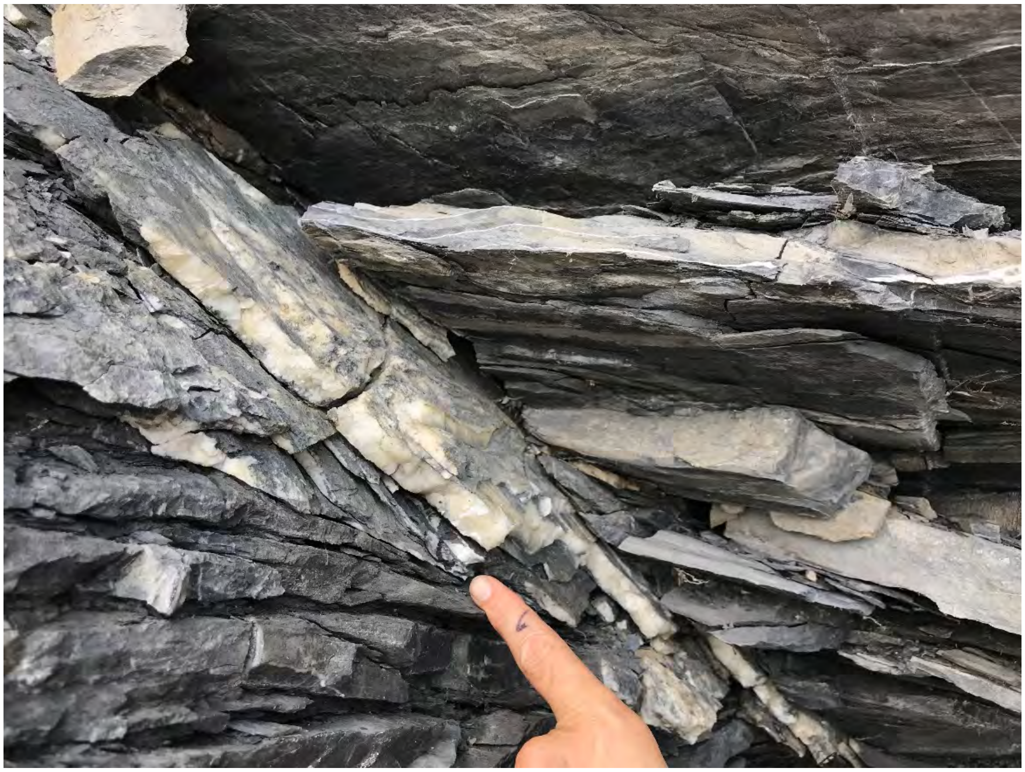

C229D:

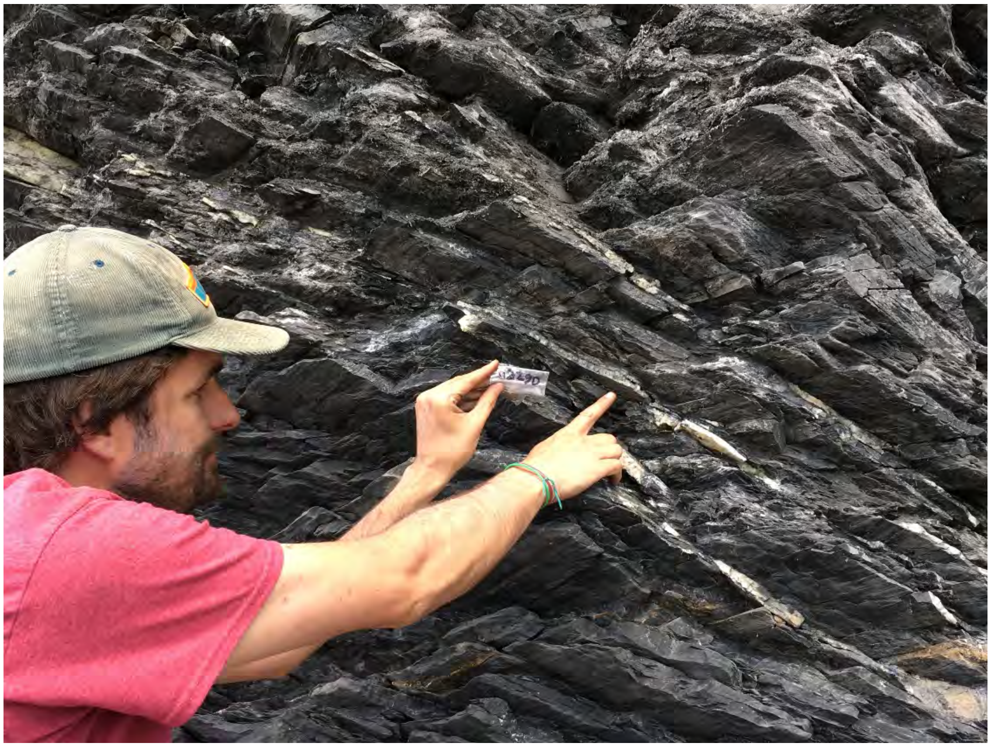

C229F:

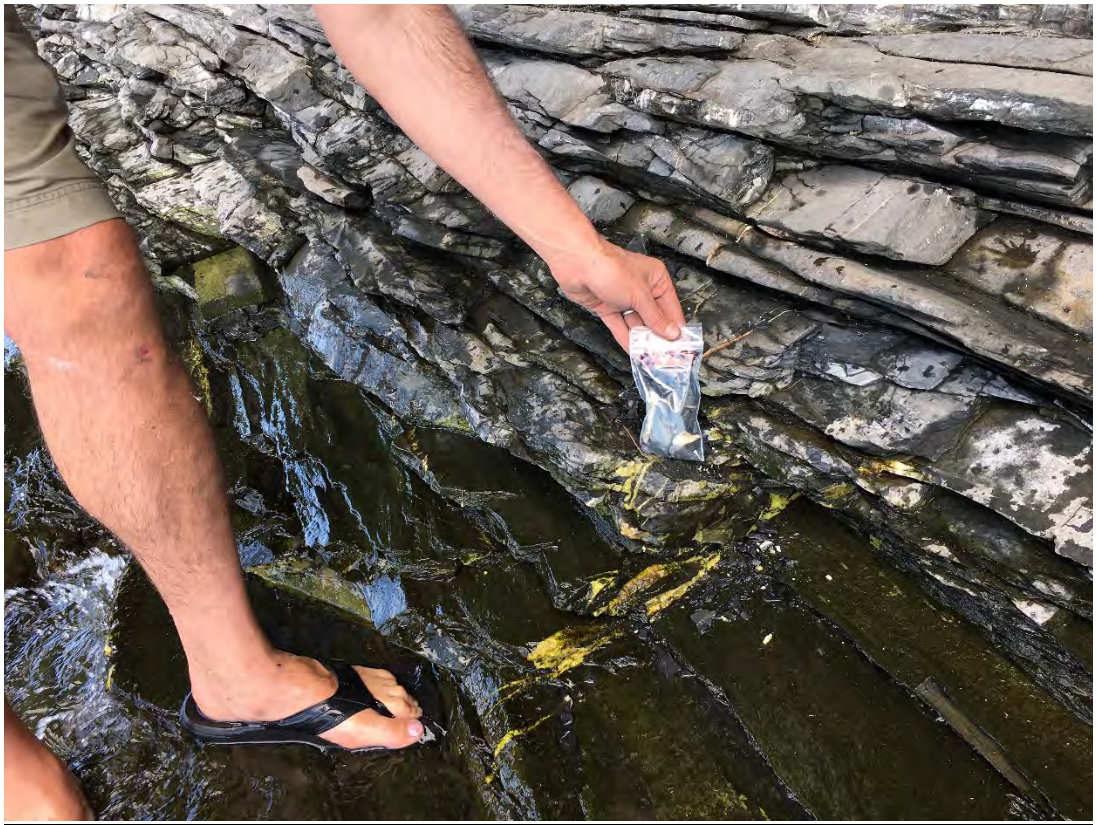

<u>Lab Description</u>:

C229A:

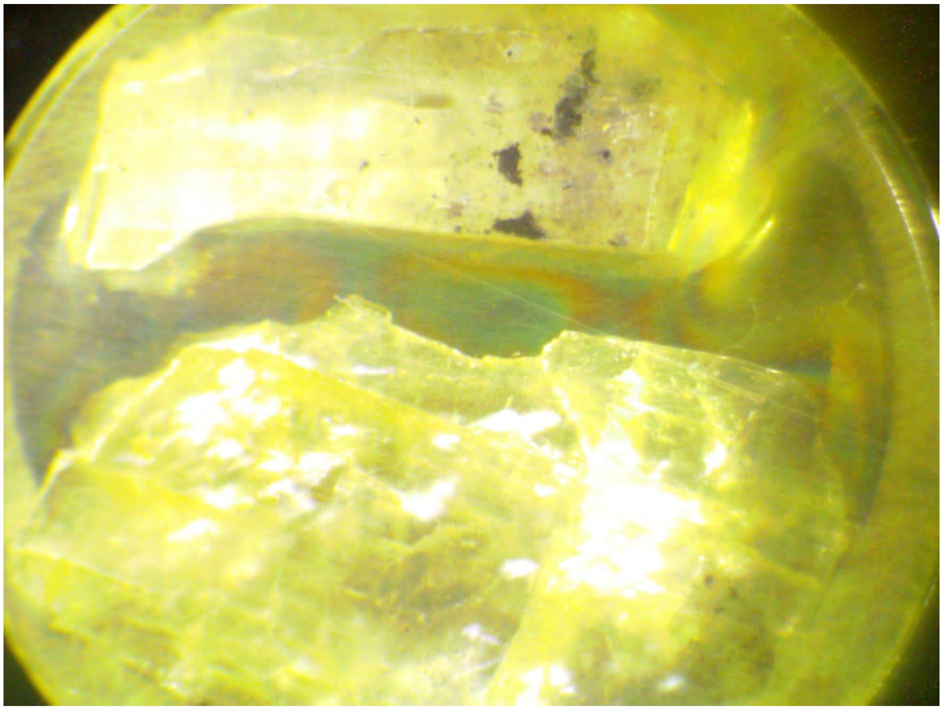

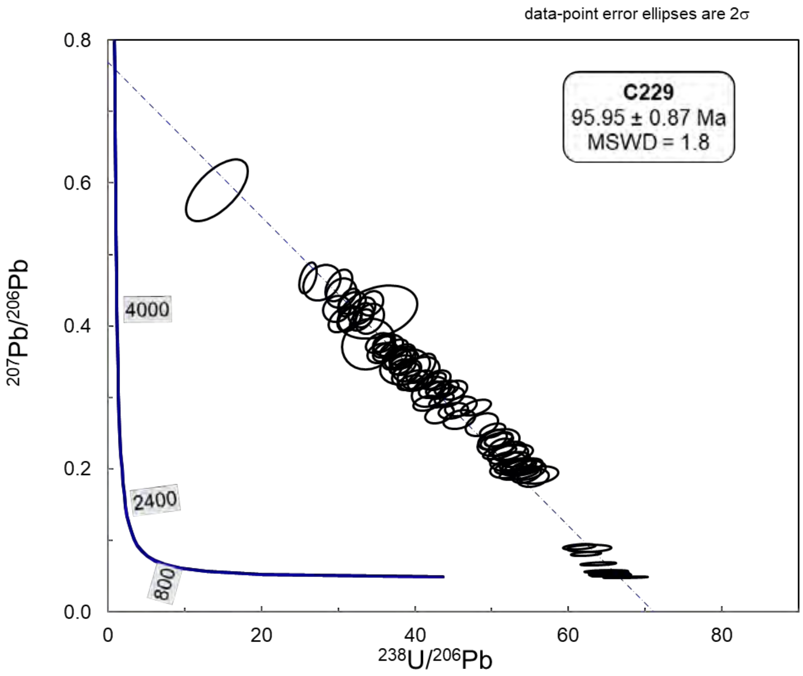

C229B:

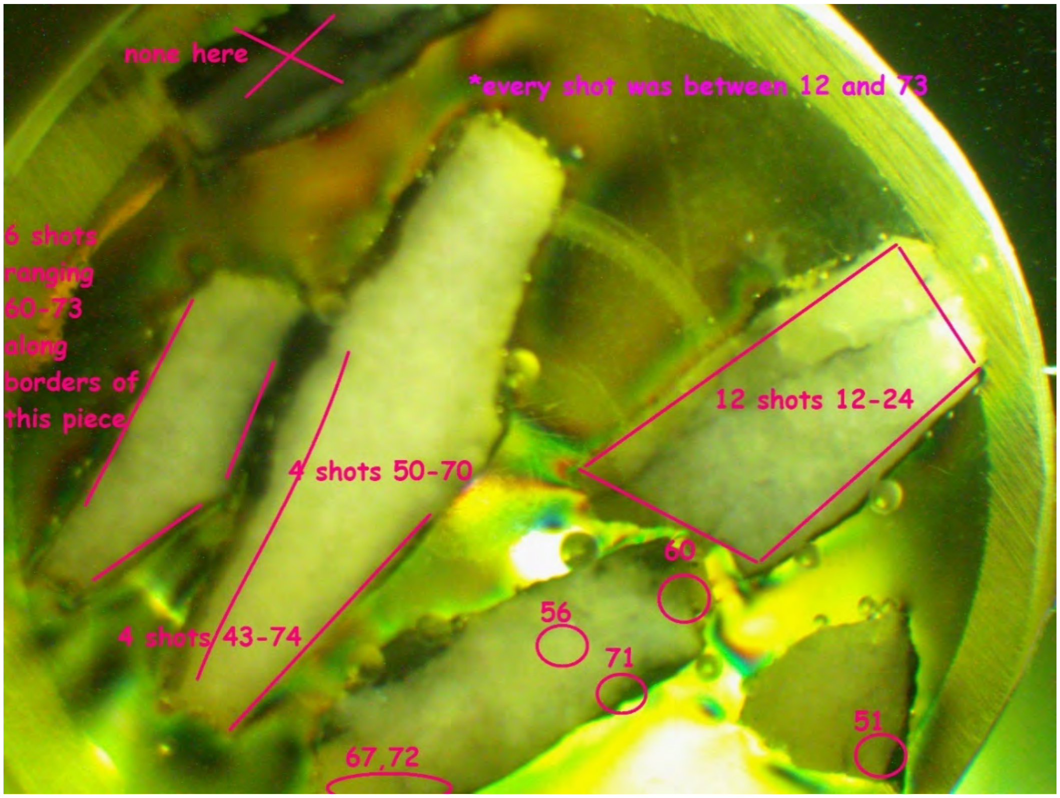
none here
*every shot was between 12 and 73
6 shots ranging 60-73 along borders of this piece
12 shots 12-24
4 shots 50-70
4 shots 43-74
60
56
71
51
67,72

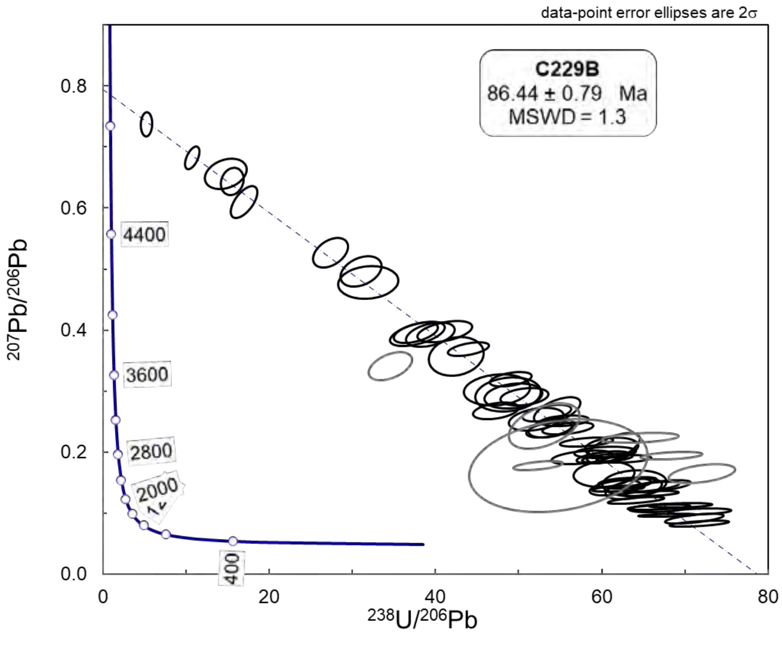
data-point error ellipses are 2σ
C229B
86.44 ± 0.79 Ma
MSWD = 1.3
4400
3600
2800
2000
400
0.0
0.2
0.4
0.6
0.8
0
20
40
60
80
207Pb/206Pb
238U/206Pb

C229C:

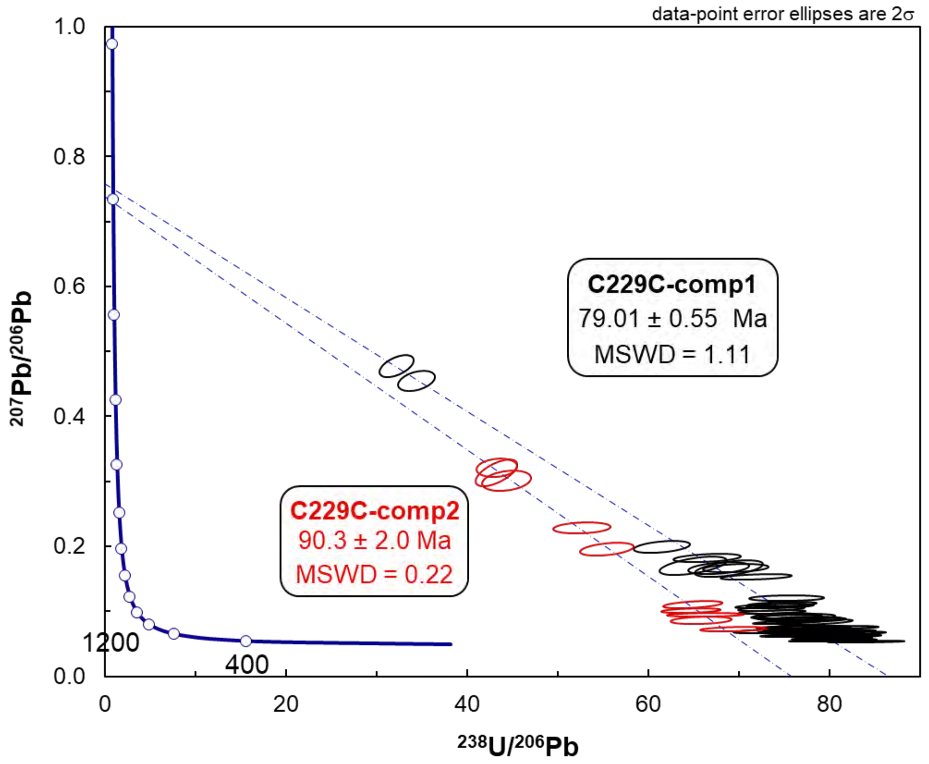

data-point error ellipses are 2σ
1.0
0.8
0.6
0.4
0.2
0.0
207Pb/206Pb
C229C-comp1
79.01 ± 0.55 Ma
MSWD = 1.11
C229C-comp2
90.3 ± 2.0 Ma
MSWD = 0.22
1200
400
0
20
40
60
80
238U/206Pb

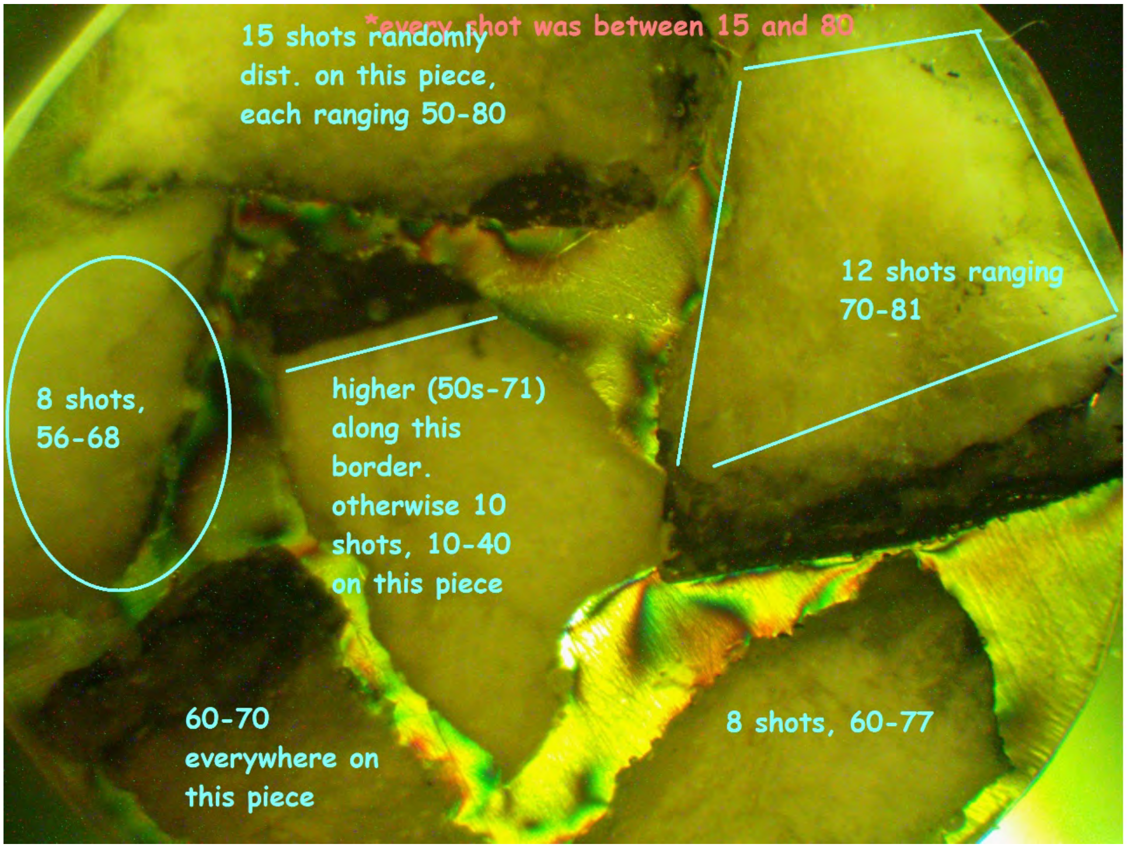

15 shots randomly dist. on this piece, each ranging 50-80
*every shot was between 15 and 80
12 shots ranging 70-81
8 shots, 56-68
higher (50s-71) along this border. otherwise 10 shots, 10-40 on this piece
60-70 everywhere on this piece
8 shots, 60-77

C229D:

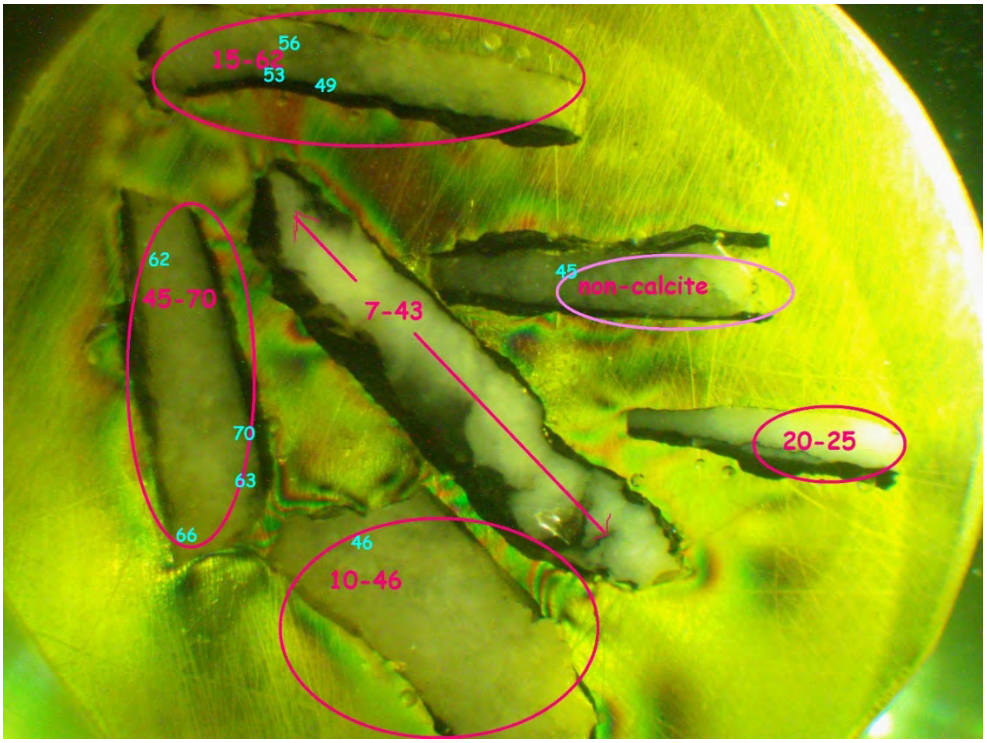

15-62
56
53
49
62
45-70
7-43
45
non-calcite
70
63
66
20-25
46
10-46

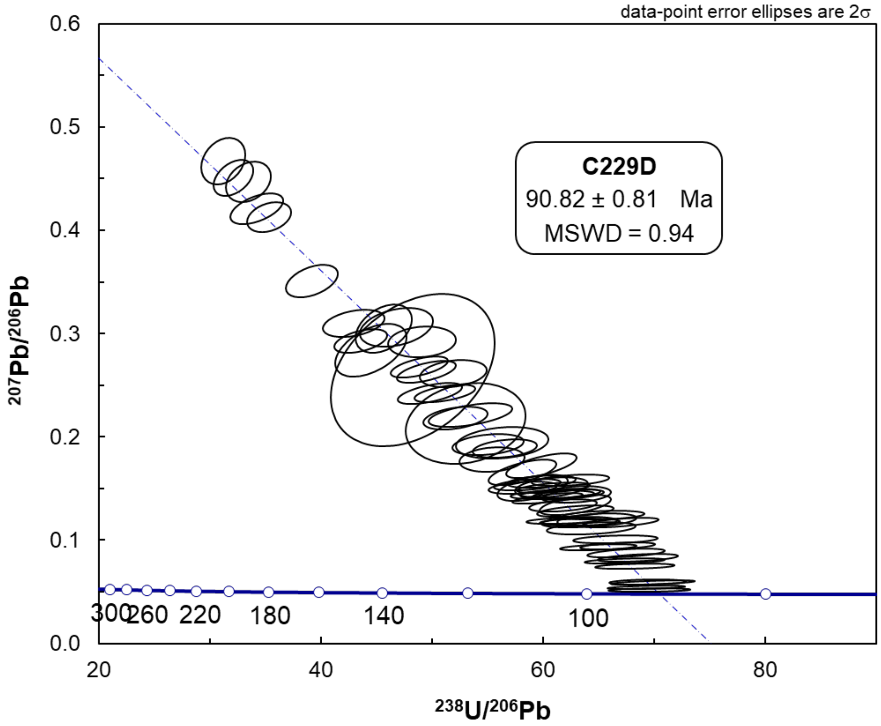

data-point error ellipses are 2σ
C229D
90.82 ± 0.81 Ma
MSWD = 0.94
0.6
0.5
0.4
0.3
0.2
0.1
0.0
300
260
220
180
140
100
20
40
60
80
207Pb/206Pb
238U/206Pb

C229F:

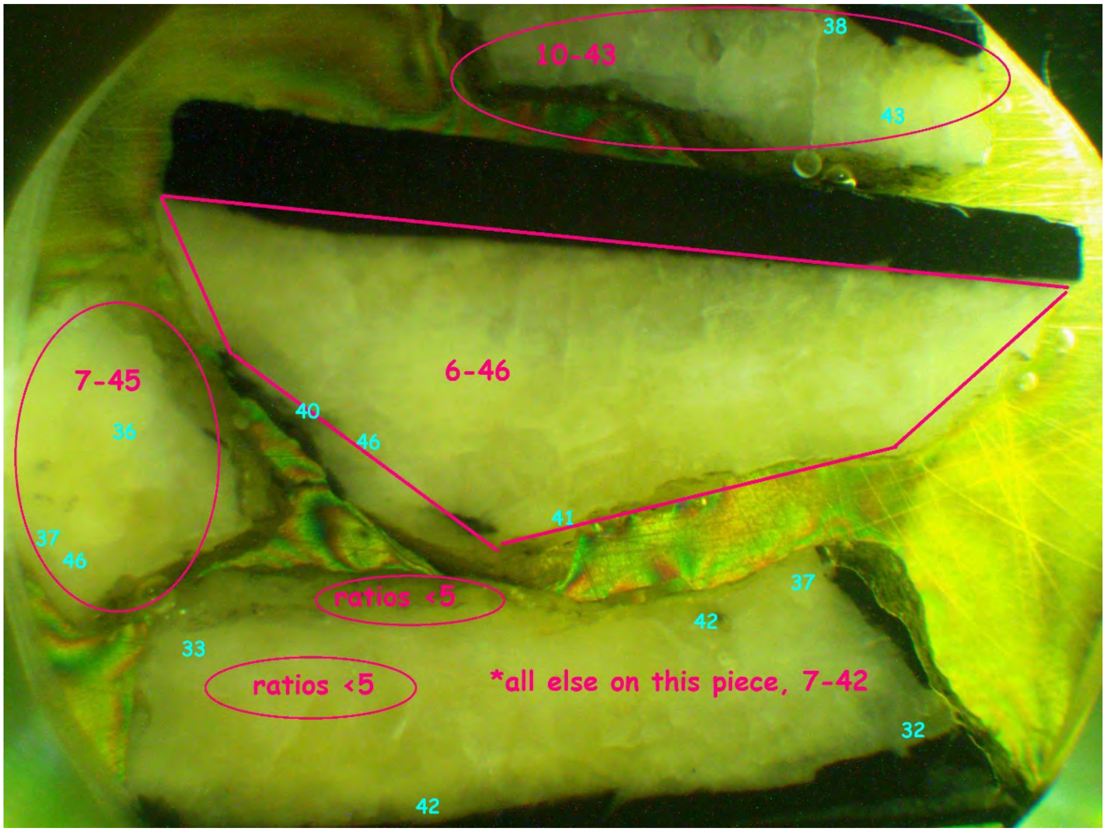

10-43
38
43
7-45
36
37
46
6-46
40
46
41
ratios <5
ratios <5
37
42
33
*all else on this piece, 7-42
32
42

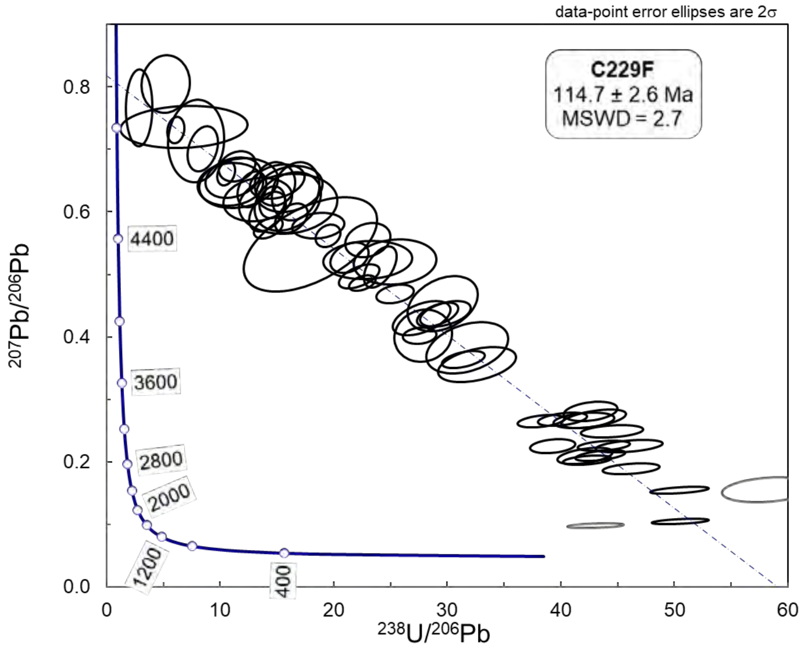

data-point error ellipses are 2σ
C229F
114.7 ± 2.6 Ma
MSWD = 2.7
4400
3600
2800
2000
1200
400
0.0
0.2
0.4
0.6
0.8
0
10
20
30
40
50
60
207Pb/206Pb
238U/206Pb
