# Supplementary material for: Pace of passive margin tectonism revealed by U-Pb dating of fracture-filling calcite
Source: Nat Commun. 2022 Apr 12;13:1953. doi: 10.1038/s41467-022-29680-z (PMC9005700; doi:10.1038/s41467-022-29680-z)

14: Hills Point North, Charlotte (44.34690, -73.28474)

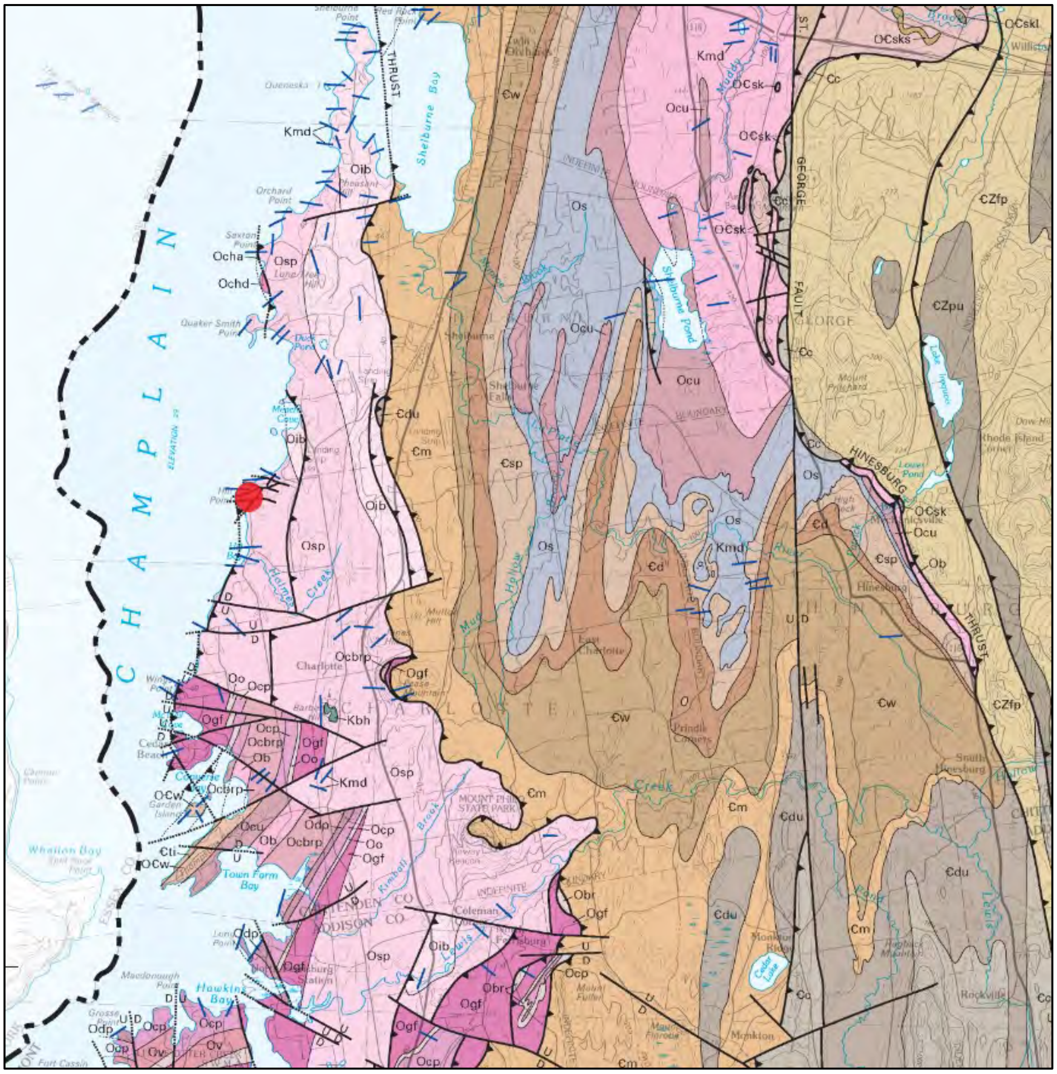

Figure 1: Sample location on geologic map (Ratcliffe et al., 2011).

Dated samples: C231B,C,D

Failed samples from nearby: C229E, C230F, C231E,F,G; C232, C233, C234

Ages: **C231B**: 79.2 ± 1.2; **C231C**: 99.5 ± 2.4 **C231D**: 78.5 ± 0.7

Bedrock Unit: Stony Point Formation (Osp)

Summary: A high angle, E-W oriented extensional fault zone exposed along a private beach on Hills Point, Charlotte, VT. Most easily accessed by canoe, also via landowner Mary Tang who lives on Hills Point Rd. Fault zone strikes ~255° dipping 78° N, with well-developed slickensides showing TTN extensional slip at a trend of roughly 306°. The extensional slickensides date precisely to ~ 79 Ma (C231B + D), interpreted to record NW-SE extension at that time. A sub-parallel accessory fracture

(C231C) contains a 2-4 cm thick calcite vein, which lacks slickensides or growth strata, and is interpreted as a mode 1 fracture. It appears to record N-S oriented $\sigma 3$ stress based on the orientation if the fault and thick continuous vein within it.

U-Pb ages from this site are high quality single-domain isochron ages. This combined with replication of the ca. 79 Ma age between two samples lends high confidence that the U-Pb ages represent the timing of fracture formation and slip at this site.

Field Descriptions:

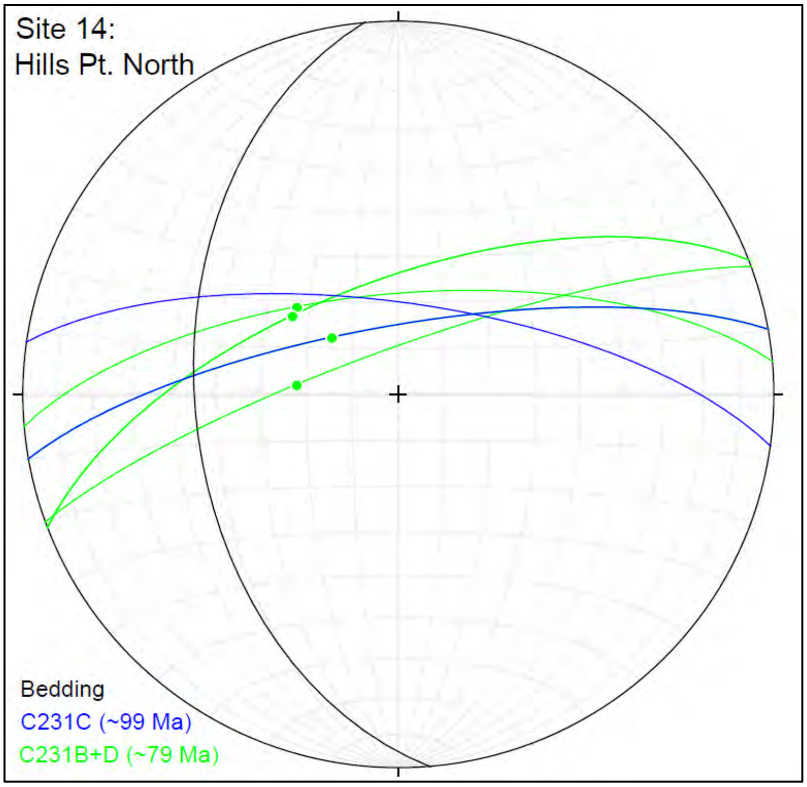

Figure 2: Stereonet showing orientations of main fault zone (C231B+D) and accessory fracture (C231C), which lacks slickensides.

General Photos:

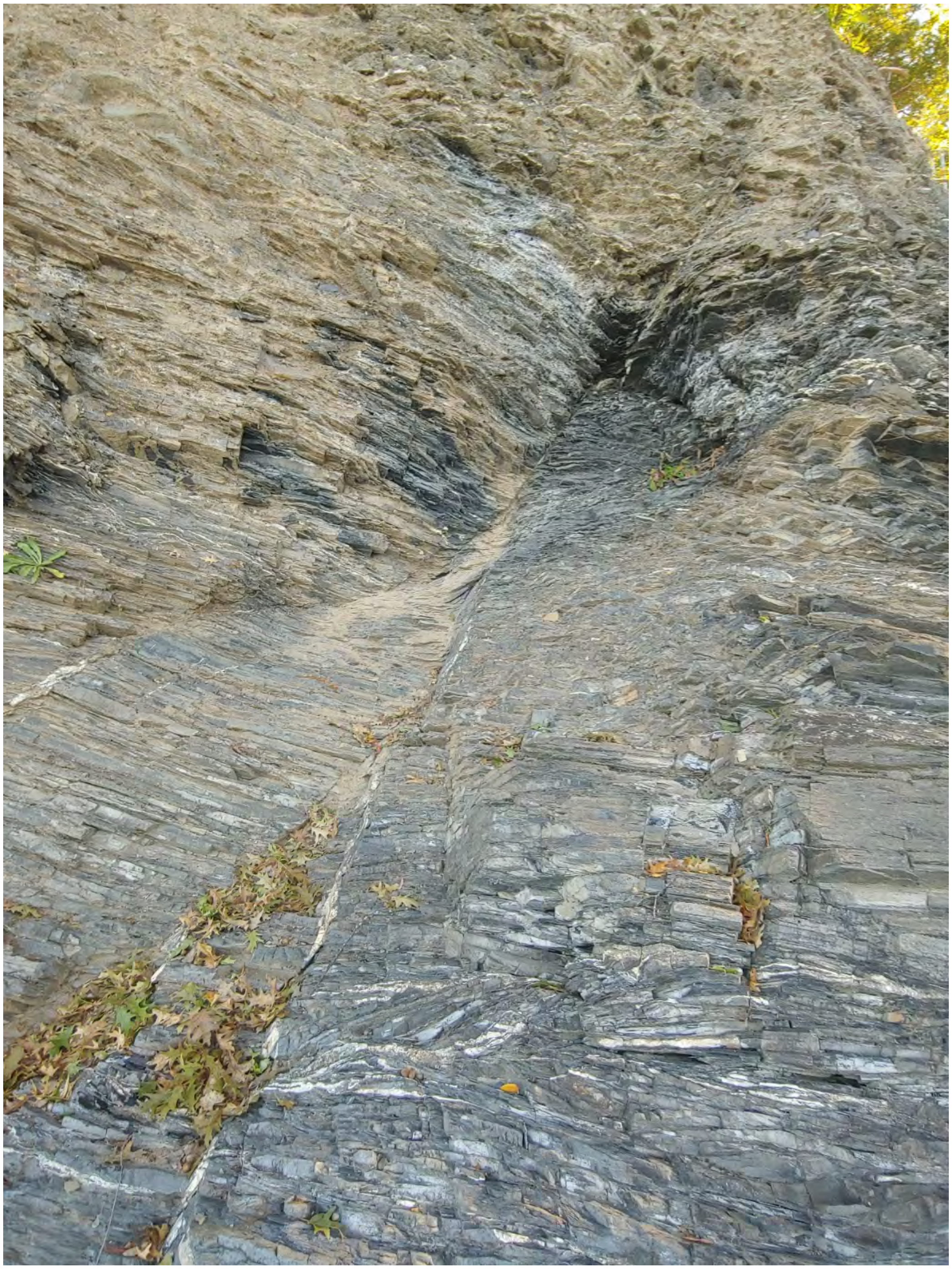

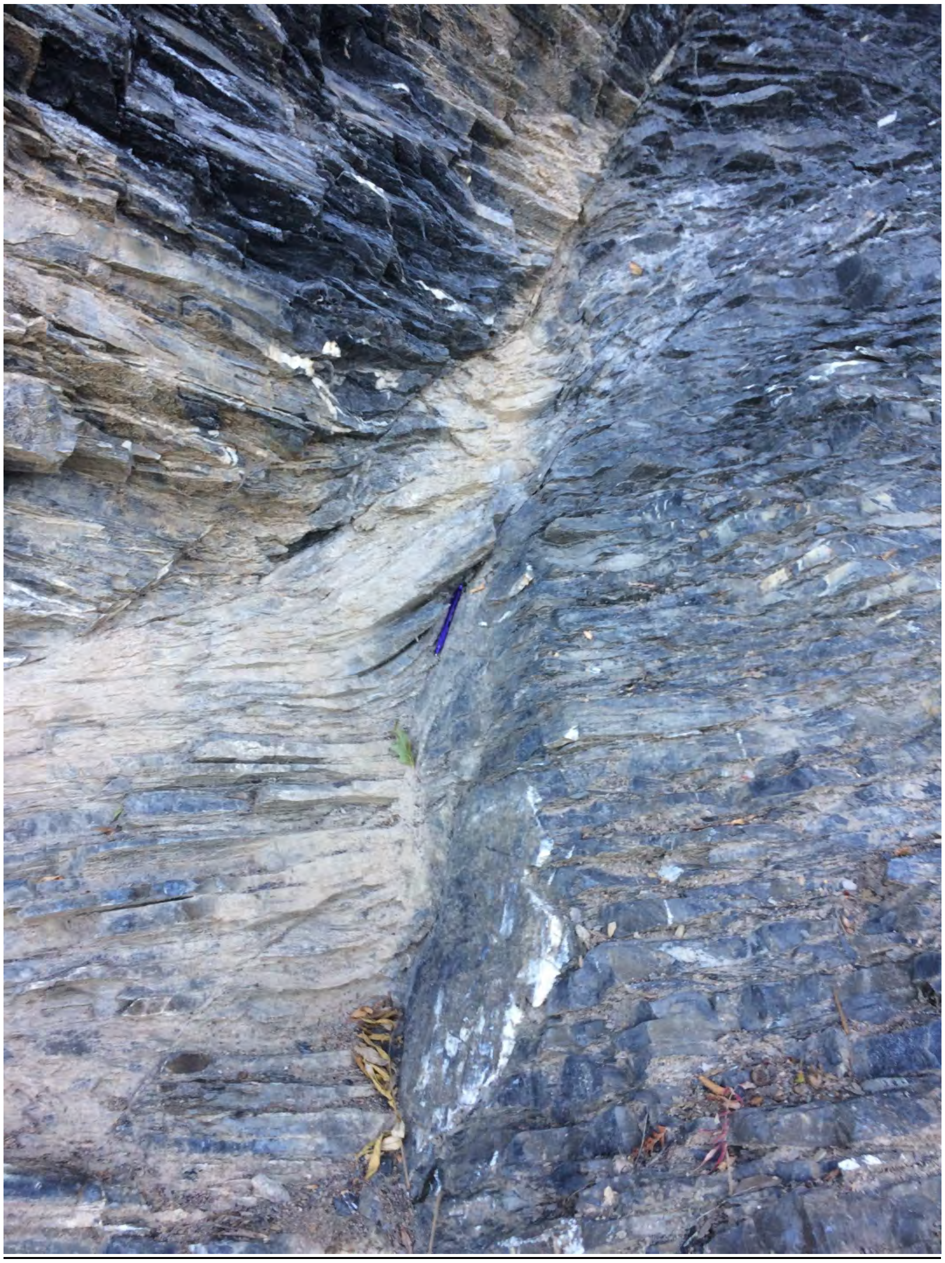

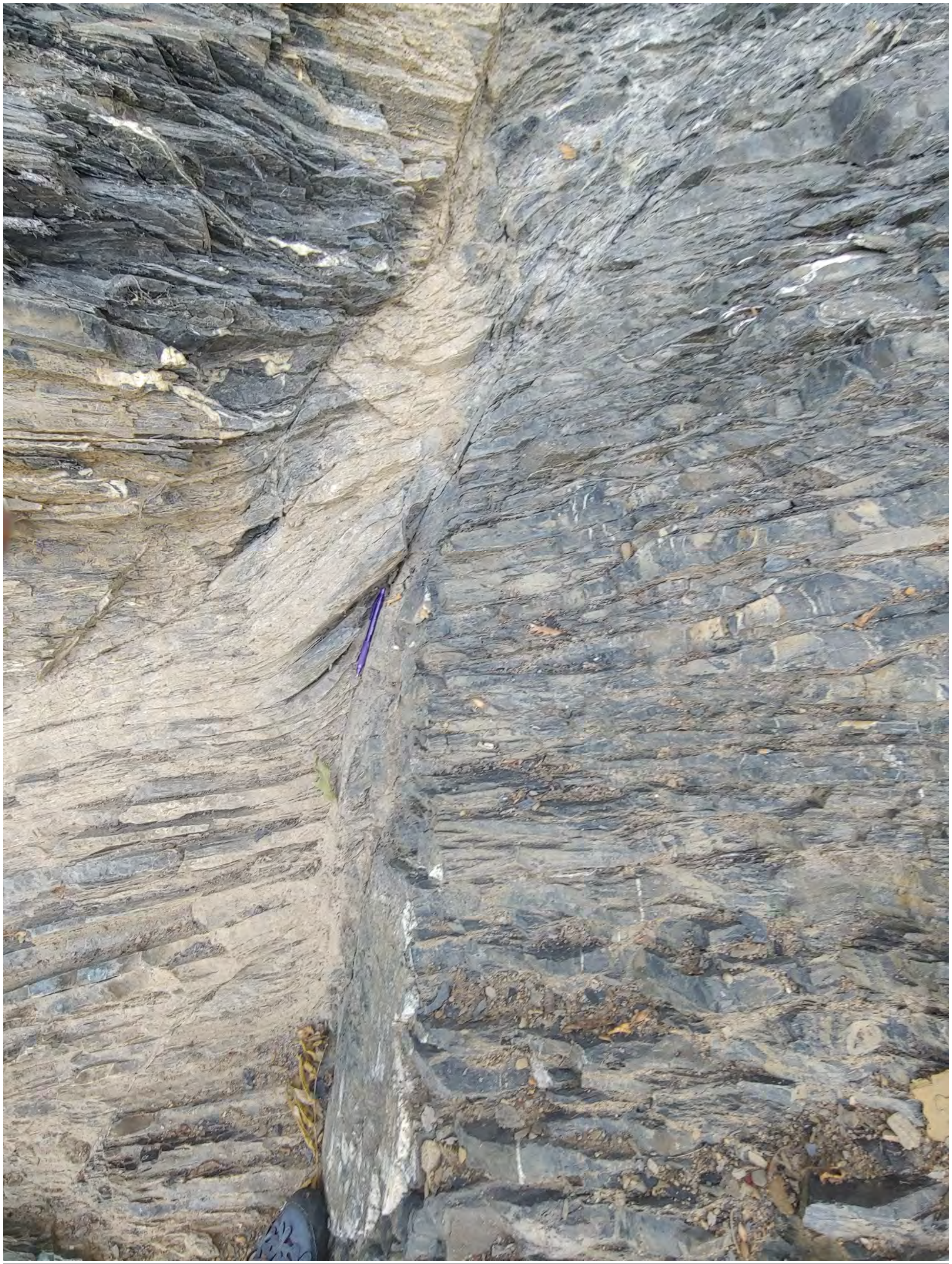

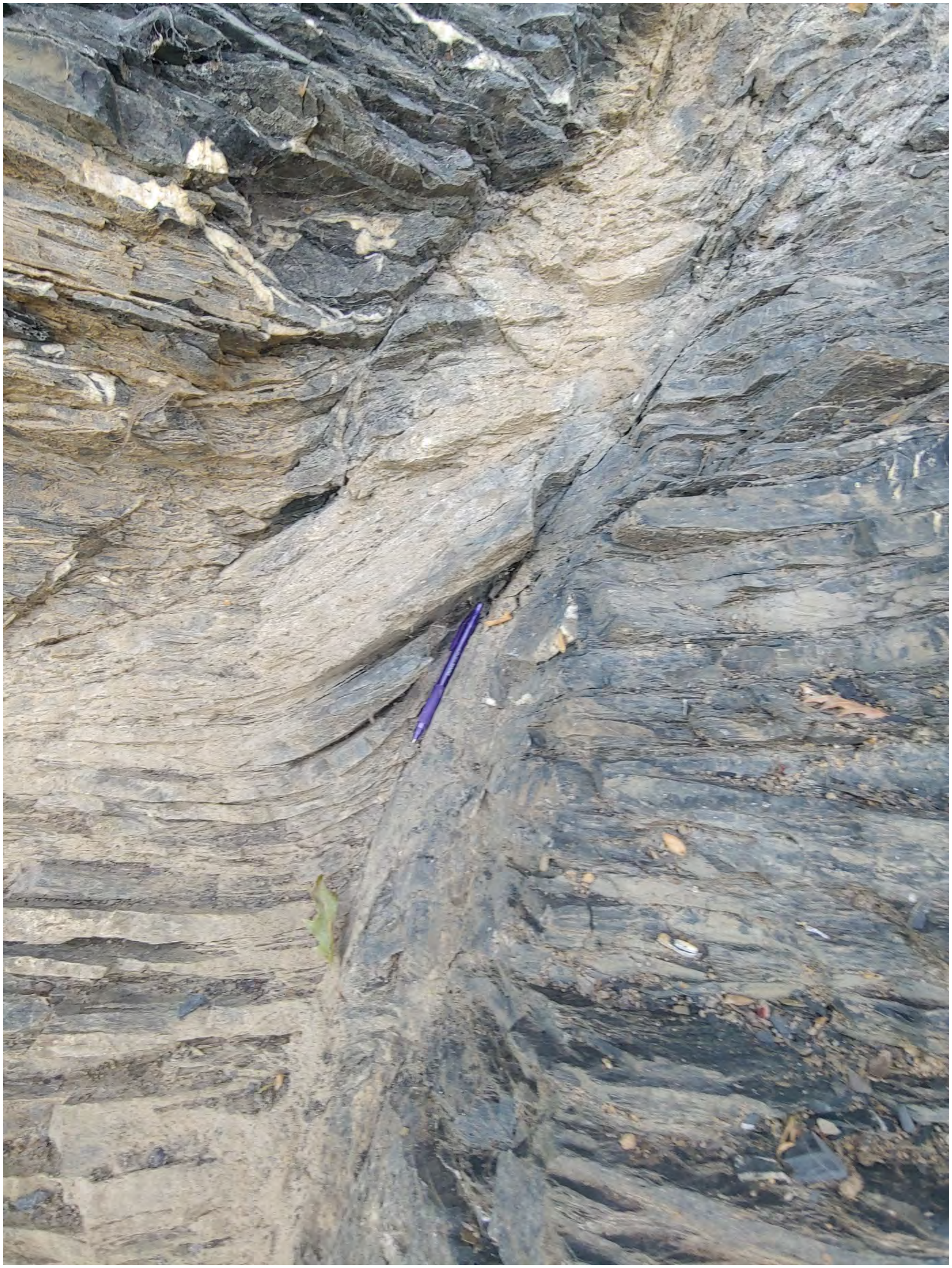

C231B

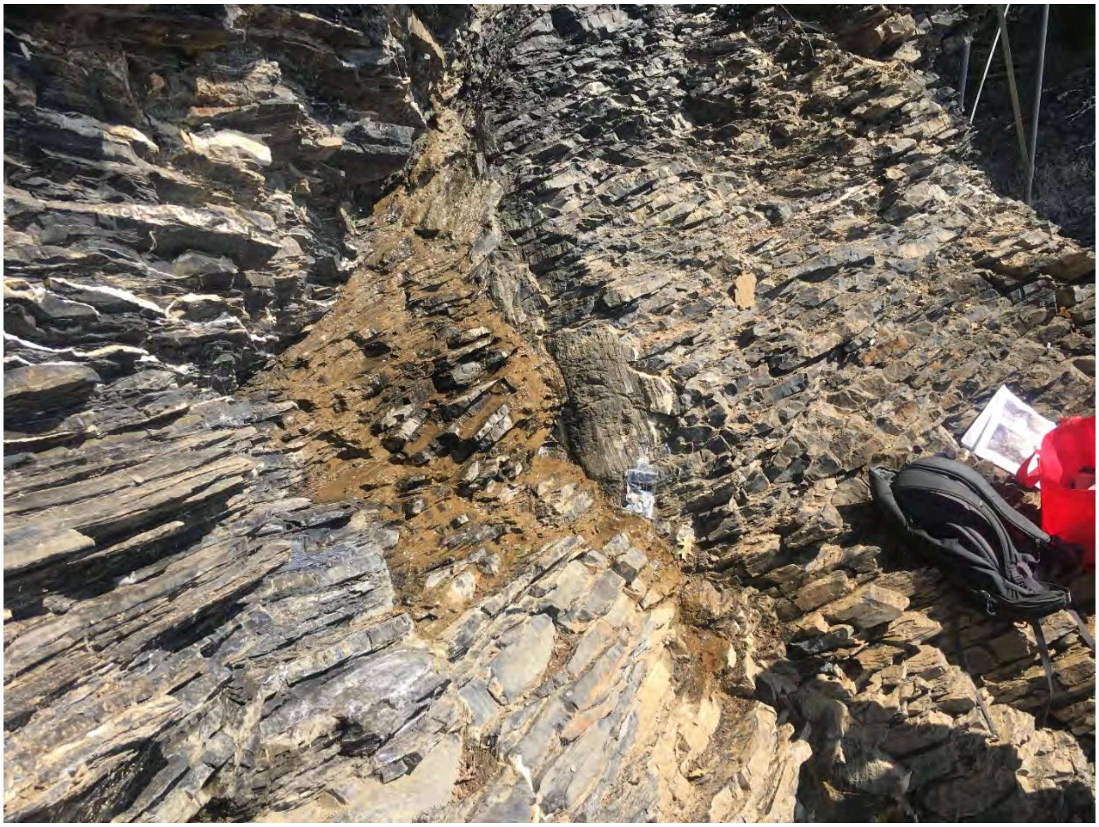

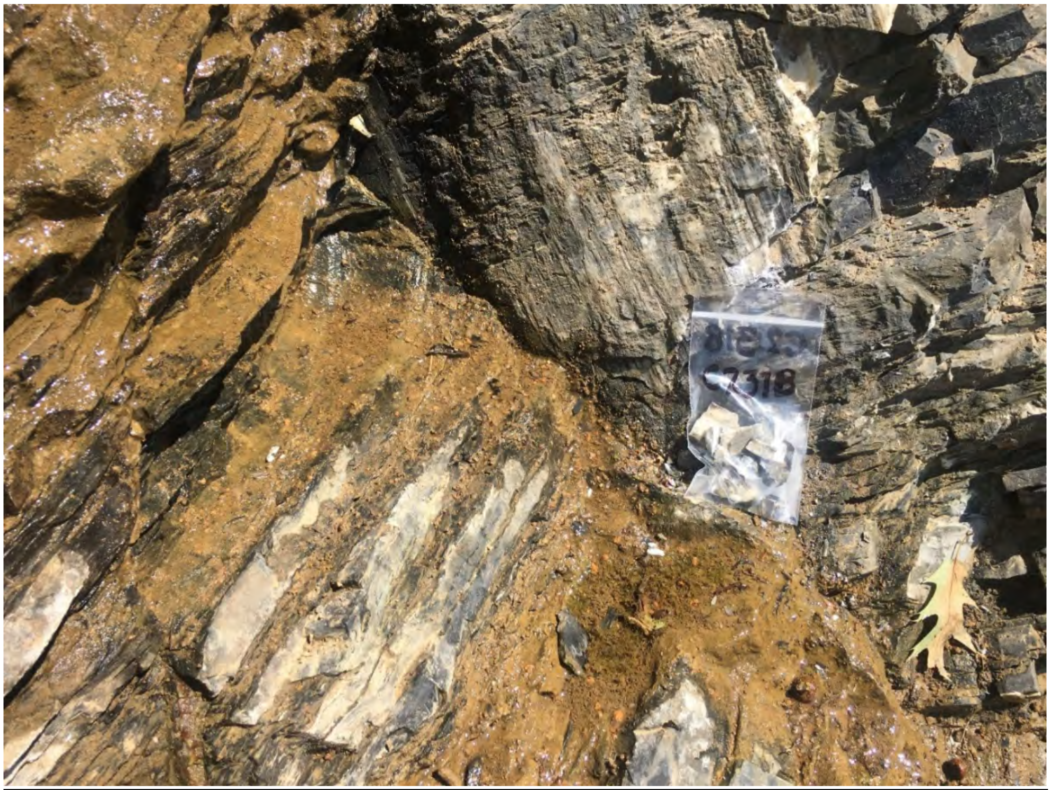

C231C

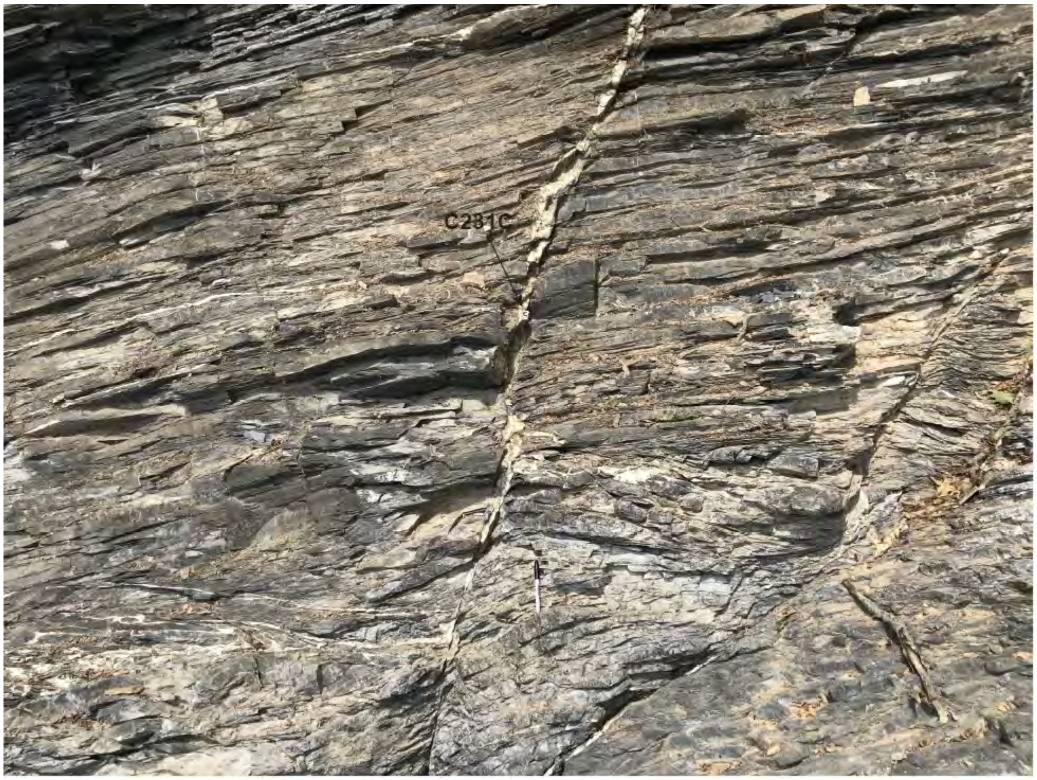

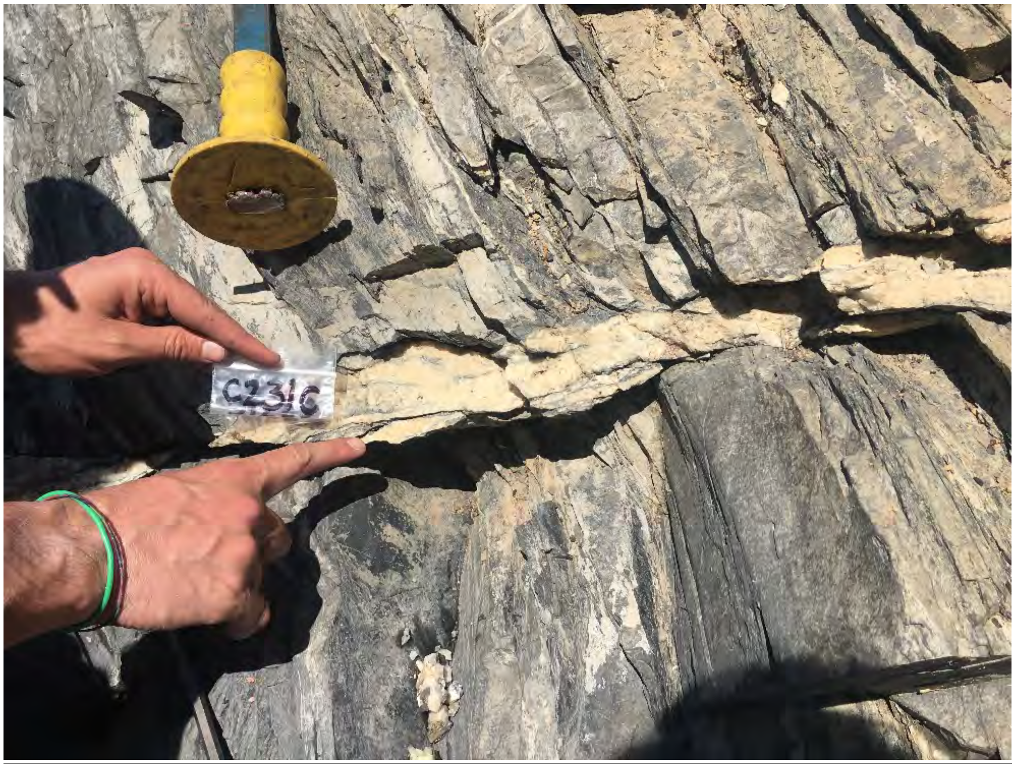

C231D

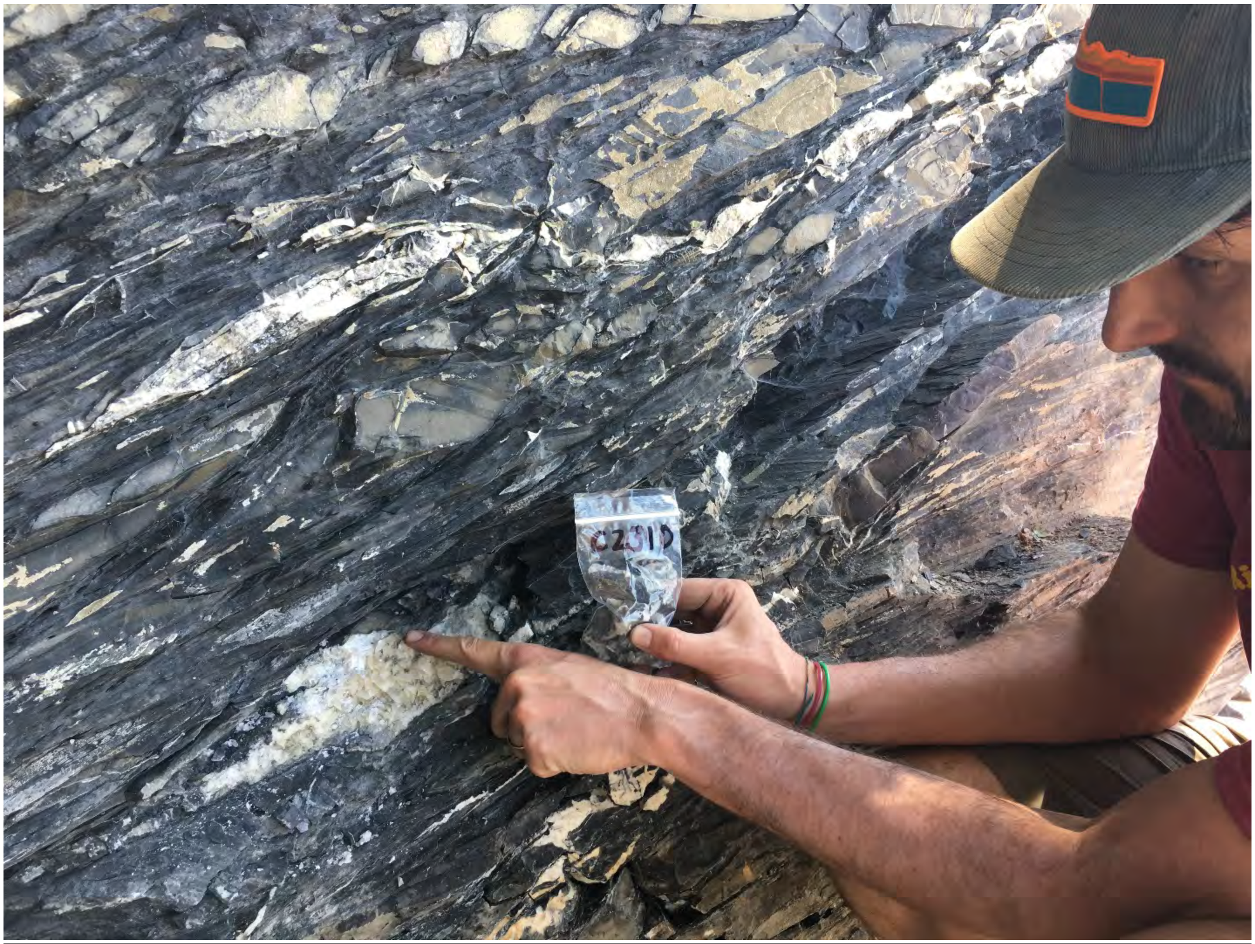

Lab Description:

C231B: circles denote areas target for dating, not laser shots

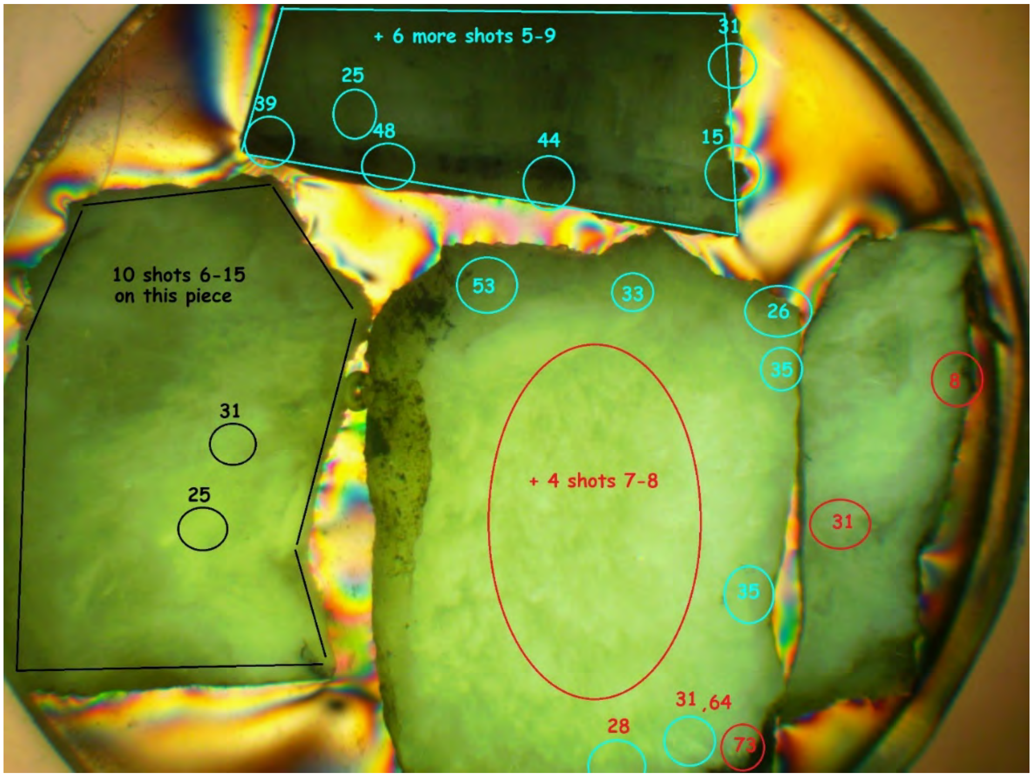

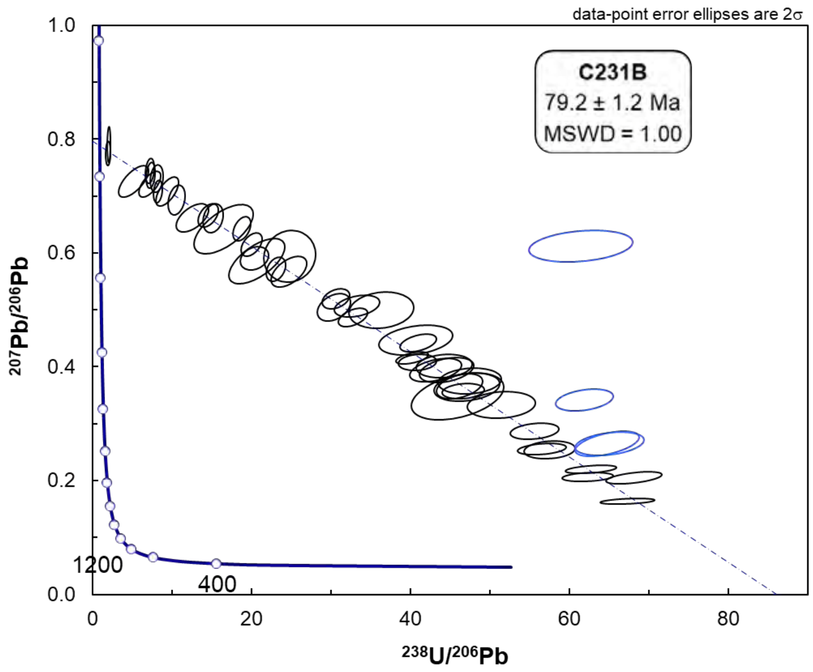

C231C: circles denote areas target for dating, not laser shots

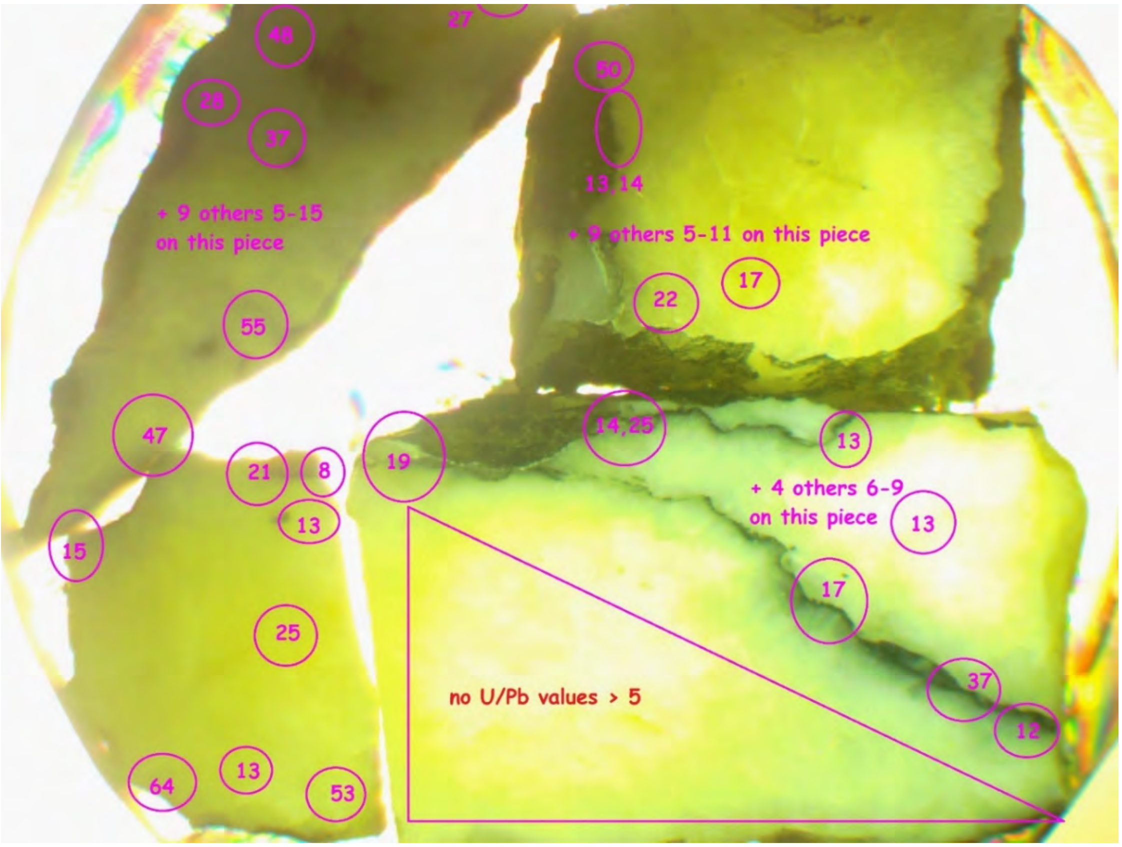

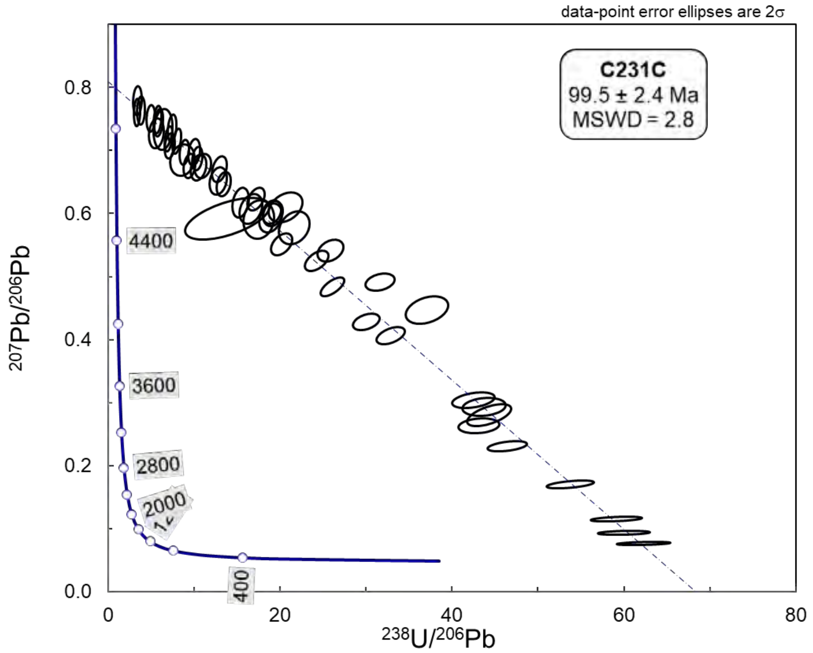

C231D: circles denote areas target for dating, not laser shots

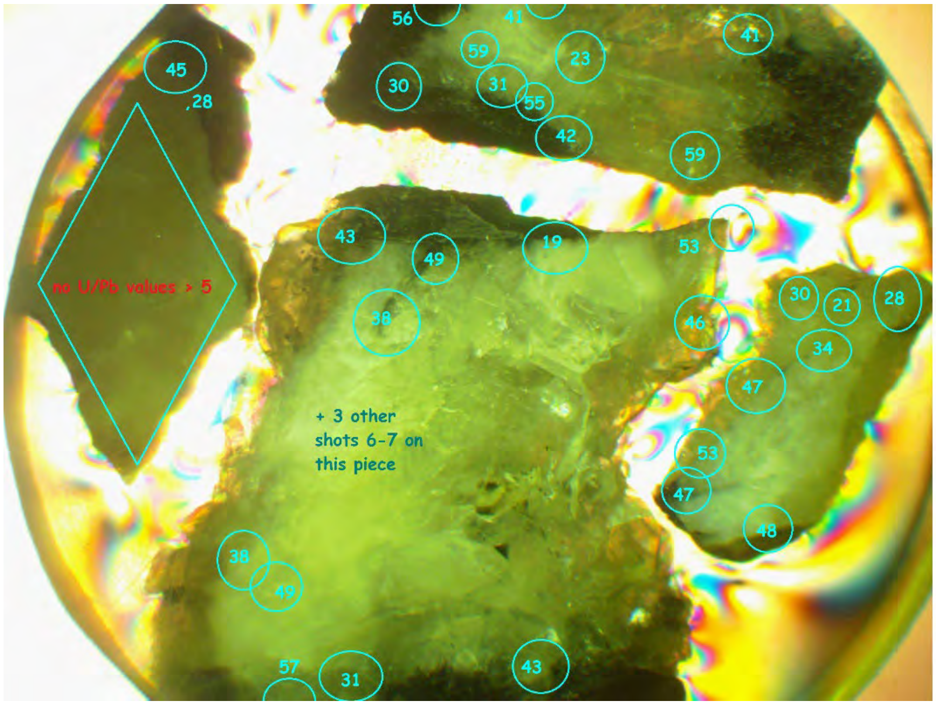

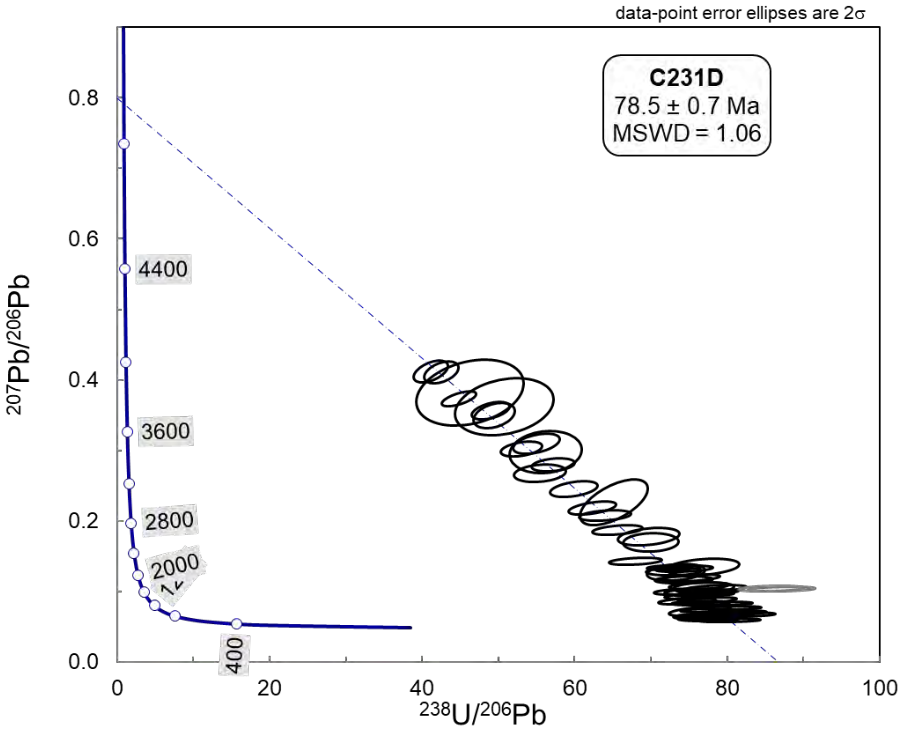

Supplement: Supplementary file 6 — Source Data [file 41467_2022_29680_MOESM6_ESM.zip › 1.zip!/appendix2_site_summaries_1to20/14_Hills Point_north_final.pdf]
