# Supplementary material for: Pace of passive margin tectonism revealed by U-Pb dating of fracture-filling calcite
Source: Nat Commun. 2022 Apr 12;13:1953. doi: 10.1038/s41467-022-29680-z (PMC9005700; doi:10.1038/s41467-022-29680-z)

15: Larson Beach (44.34881, -73.2801)

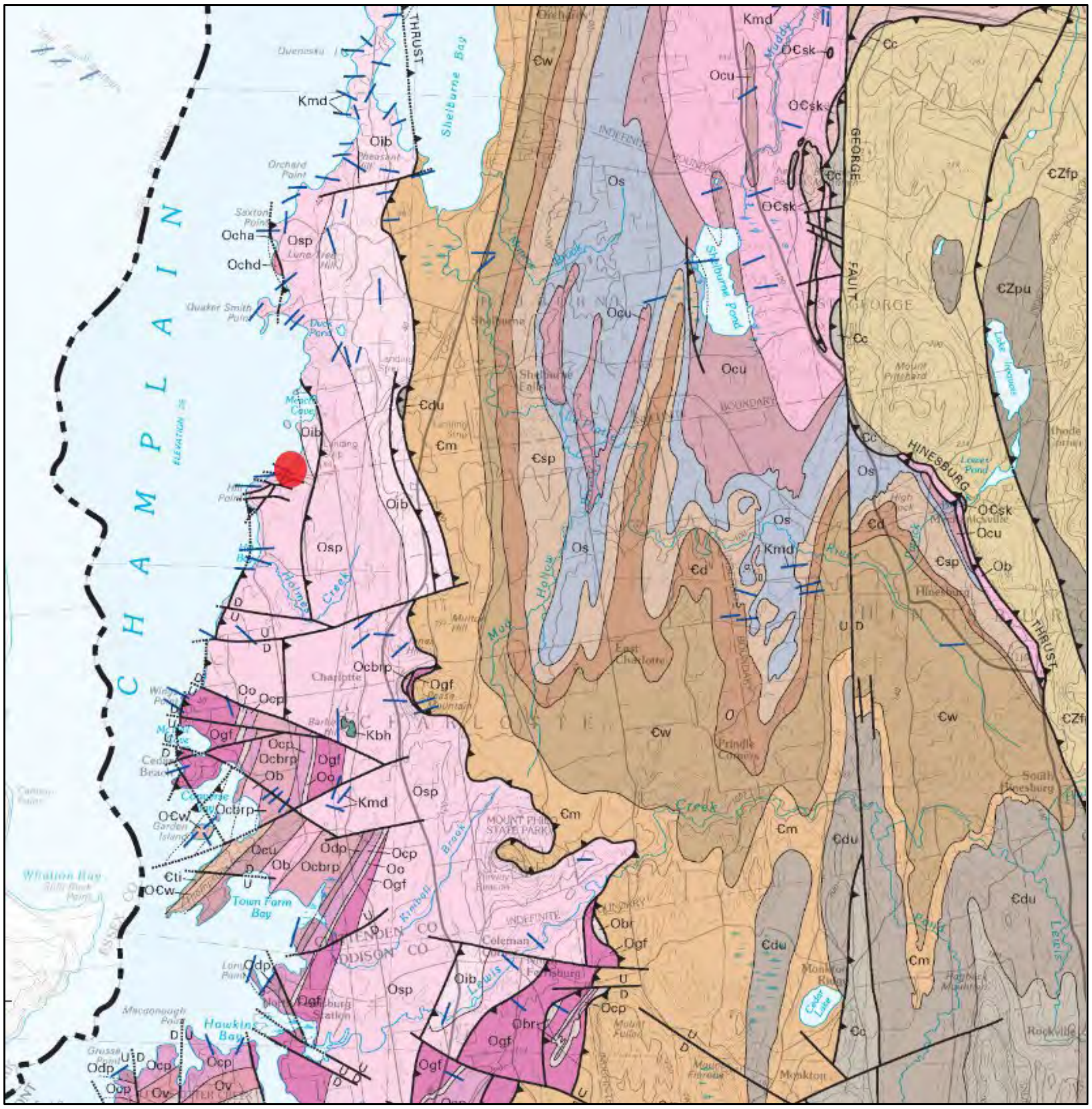

Figure 1: Sample location on state geologic map of Ratcliffe at al. (2011)

__Failed samples from same site__: C235A

__Bedrock Unit:__ Stony Point Formation (Osp)

__Ages__: **C235B**: 122.8 ± 3.4 Ma

Summary: Sampled from a distorted Trachytic dike on the north end of a private beach just north of Hills Point, VT. Contact for site access is Davin Larson on Lake Road in Charlotte (klarson@ottercreekawnings.com or 802-598-1525). This was initially sampled because we thought the vein might be associated with a small cross-cutting fault. However, closer inspection suggests the dike is actually just stepping over along a cleavage plane during intrusion. Thus, sample C235B (~123 Ma) is now interpreted as forming co-eval with dike emplacement or during very minor later offset. The sample location is characterized by a variety of chaotic calcite veins and we are unable to make any

kinematic interpretations. The U-Pb age isochron is single domain suggesting the age is primary. However, it does contain a few outlying shots suggesting the vein has experienced some alteration since its emplacement and the age has fairly significant uncertainty.

<u>Field Observations</u>:

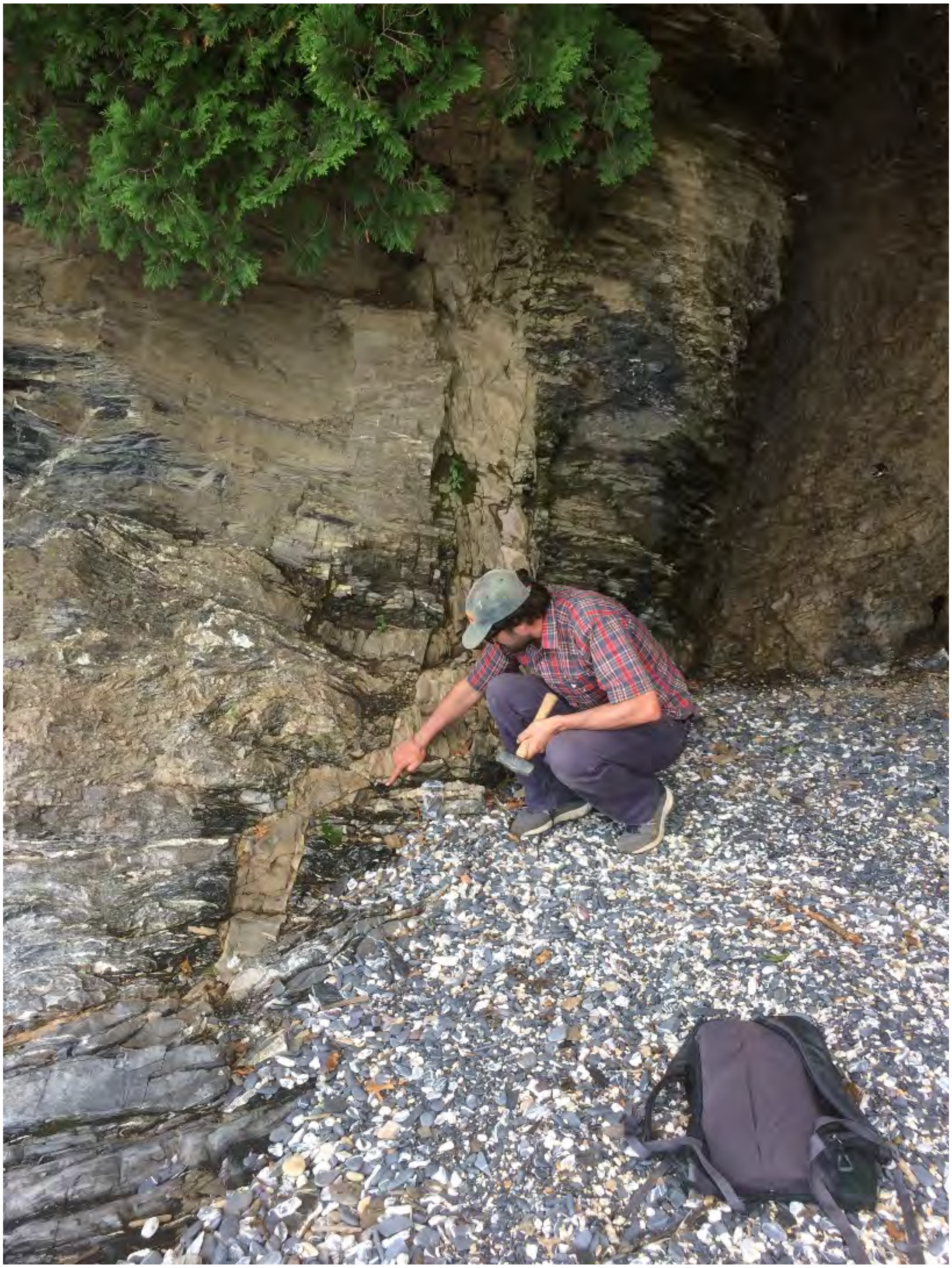

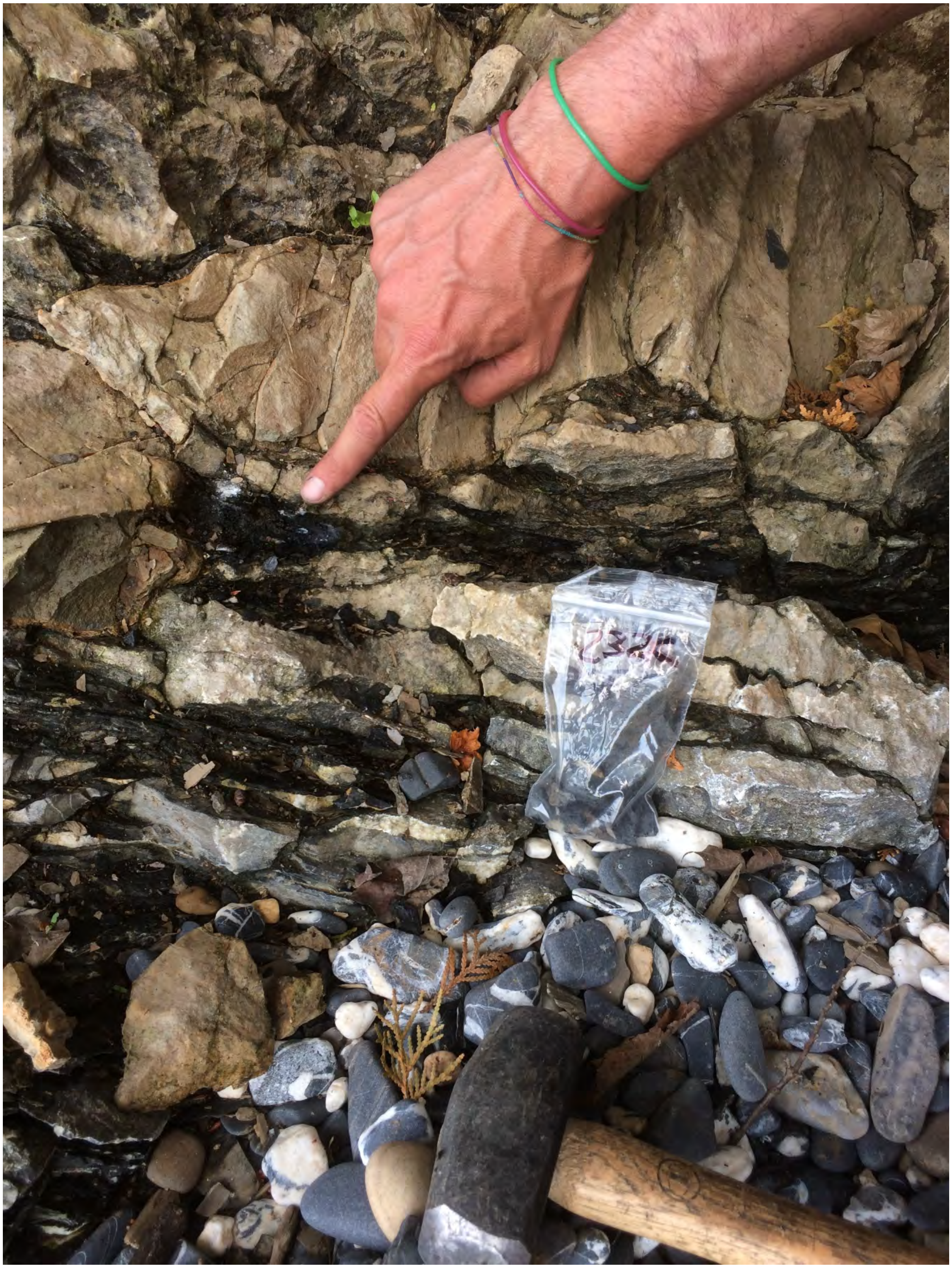

Lab Description:

C235B:

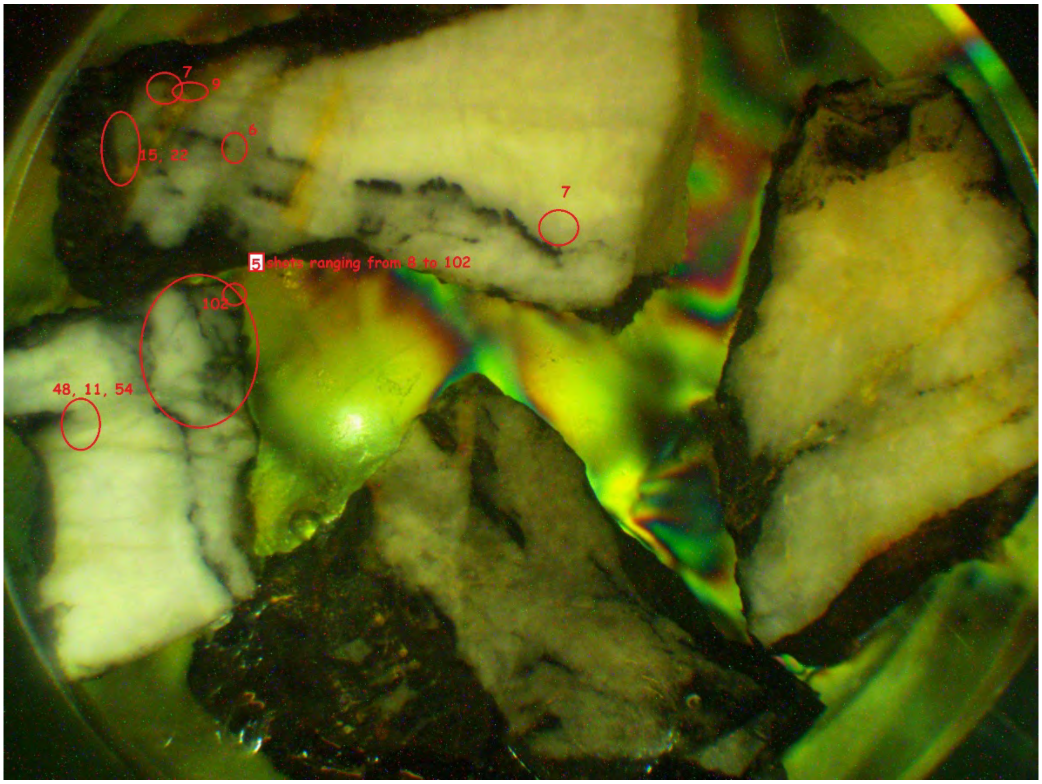

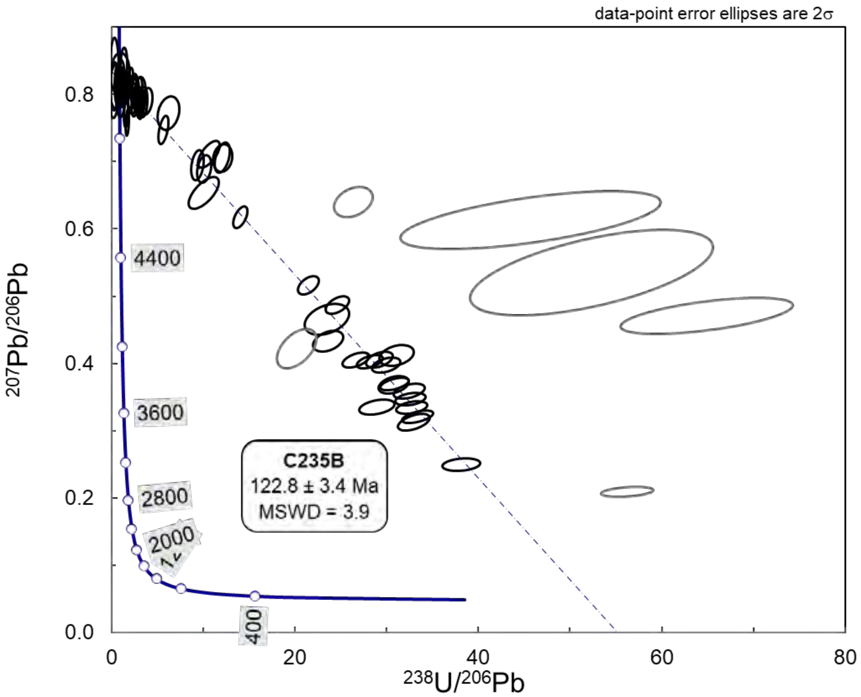

Supplement: Supplementary file 6 — Source Data [file 41467_2022_29680_MOESM6_ESM.zip › 1.zip!/appendix2_site_summaries_1to20/15_larson_beach_final.pdf]
