# Supplementary material for: Pace of passive margin tectonism revealed by U-Pb dating of fracture-filling calcite
Source: Nat Commun. 2022 Apr 12;13:1953. doi: 10.1038/s41467-022-29680-z (PMC9005700; doi:10.1038/s41467-022-29680-z)
Supplement: Supplementary file 6 — Source Data [file 41467_2022_29680_MOESM6_ESM.zip › 1.zip!/appendix2_site_summaries_1to20/16_beadles_cove_final.pdf]

16: Beadles Cove, Lake Champlain (43.84846, -73.3699)

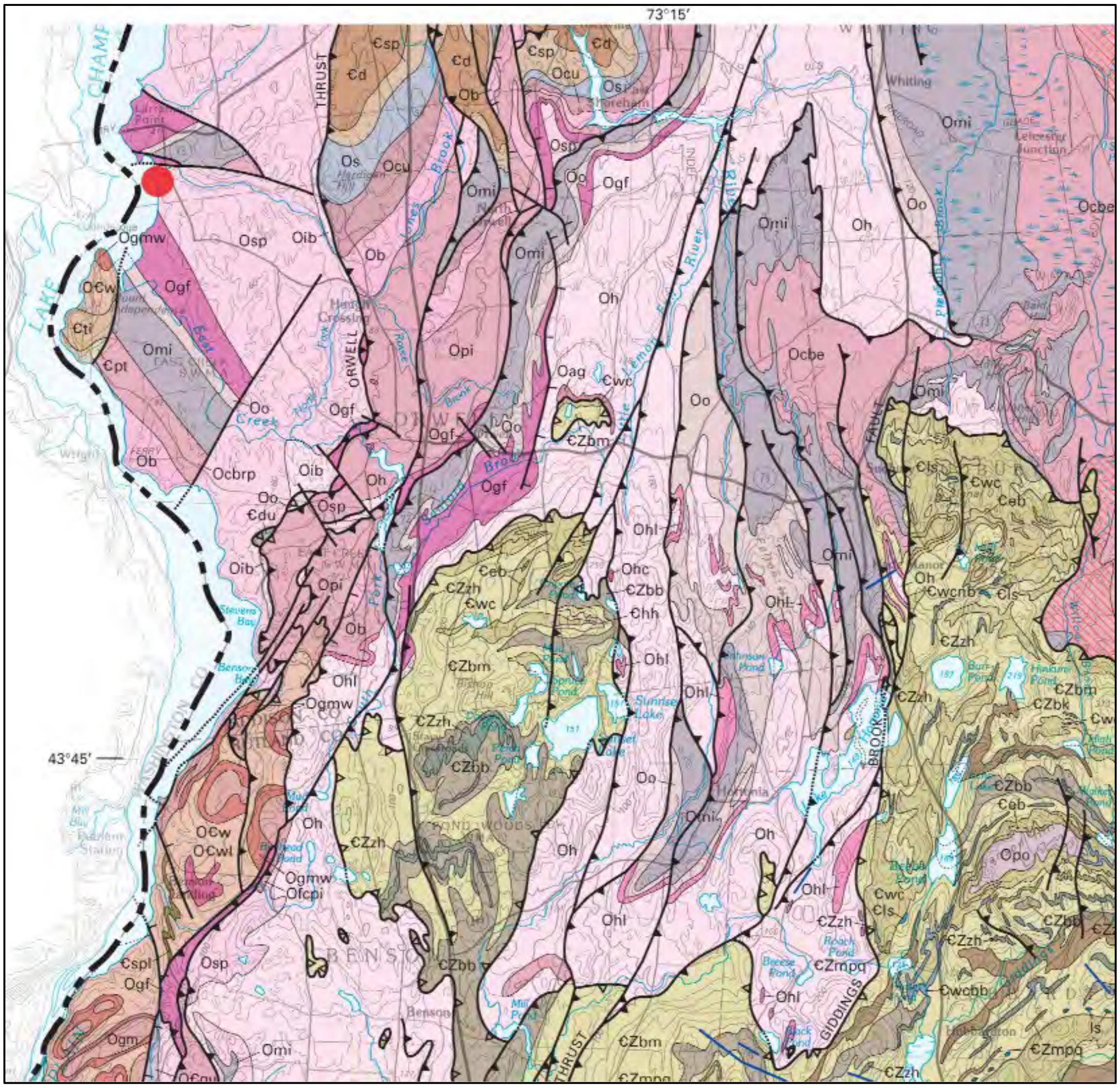

Failed samples from same site: none

Bedrock Unit: Stony Point Formation (Osp)

Ages: **C242**: -0.07±0.05 (analytical)

Summary: One dated sample from thin white veins cutting across foliation and bedding in the Iberville shale. This sample appears to be primary crystallization based on the preserved syntaxial fabrics and U-Pb data that define a single isochron age using laser shots from throughout the sample. Likewise there is no evidence for fluid flow or staining at the exposure. Nonetheless, the extremely young age and high U/Pb ratios suggest raise questions about whether the calcite is primary. Given the uncertainty on the validity of the age and the cryptic nature of the structure itself, we avoid kinematic or tectonic interpretations for this vein.

Field Descriptions:

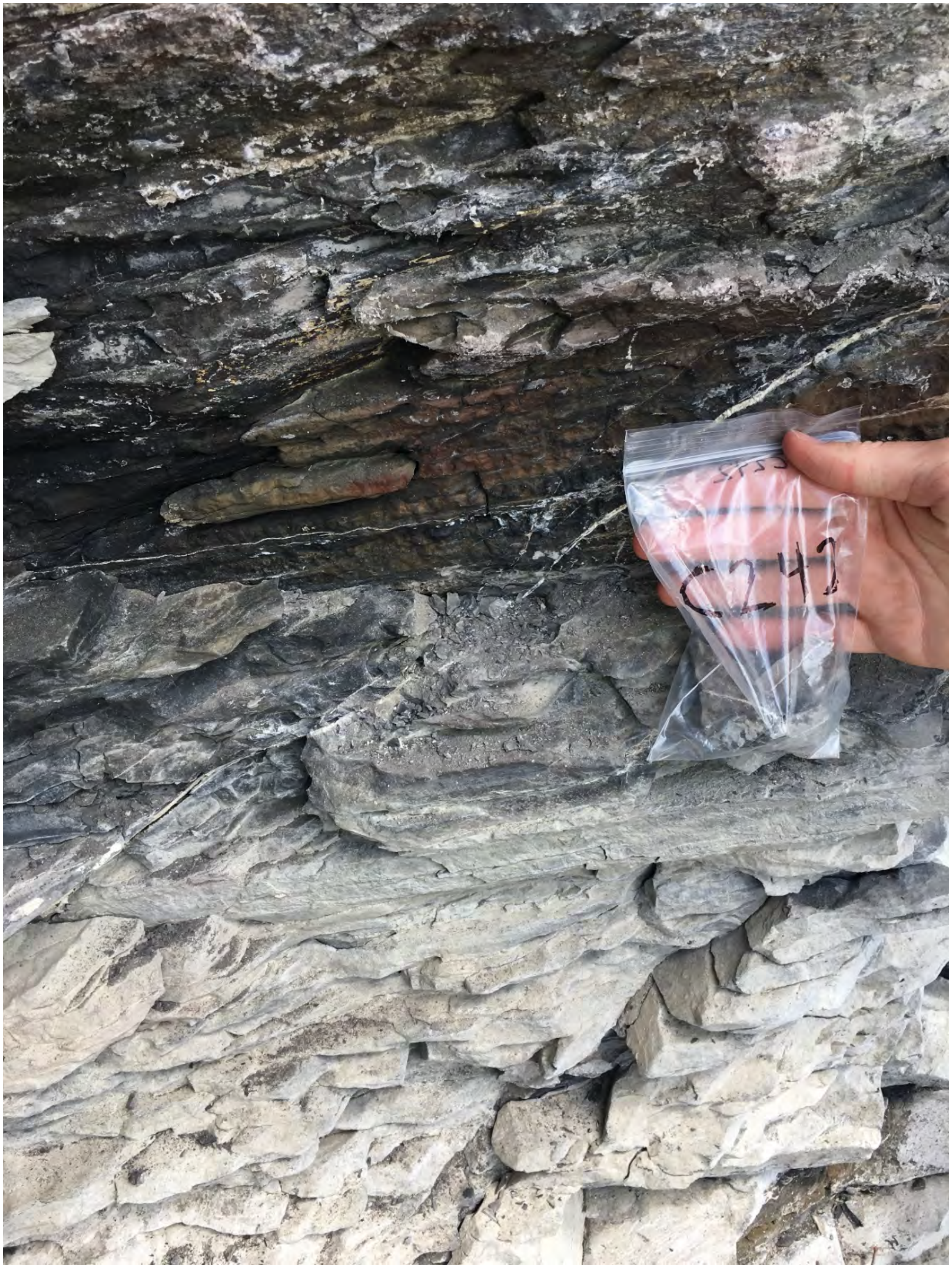

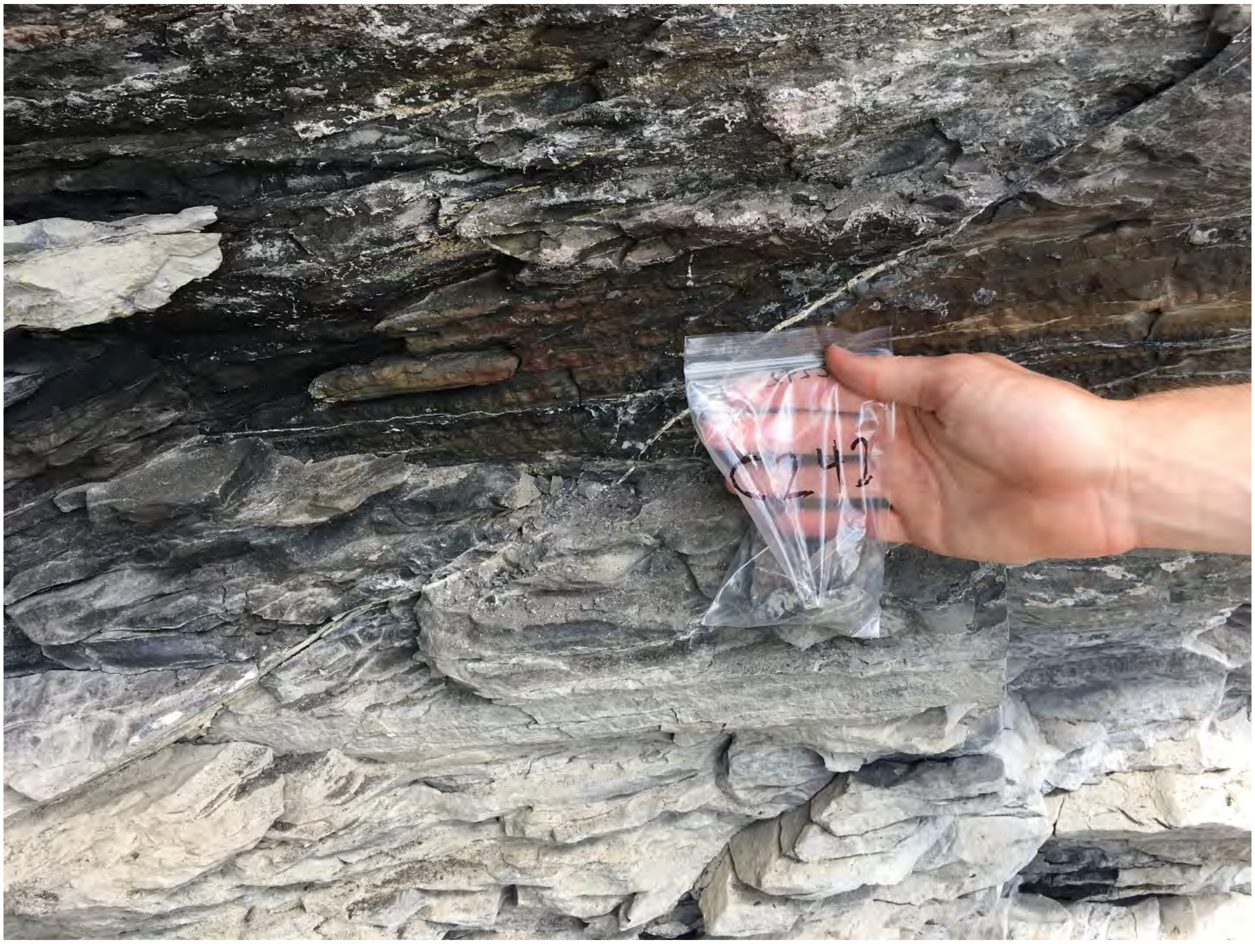
C242

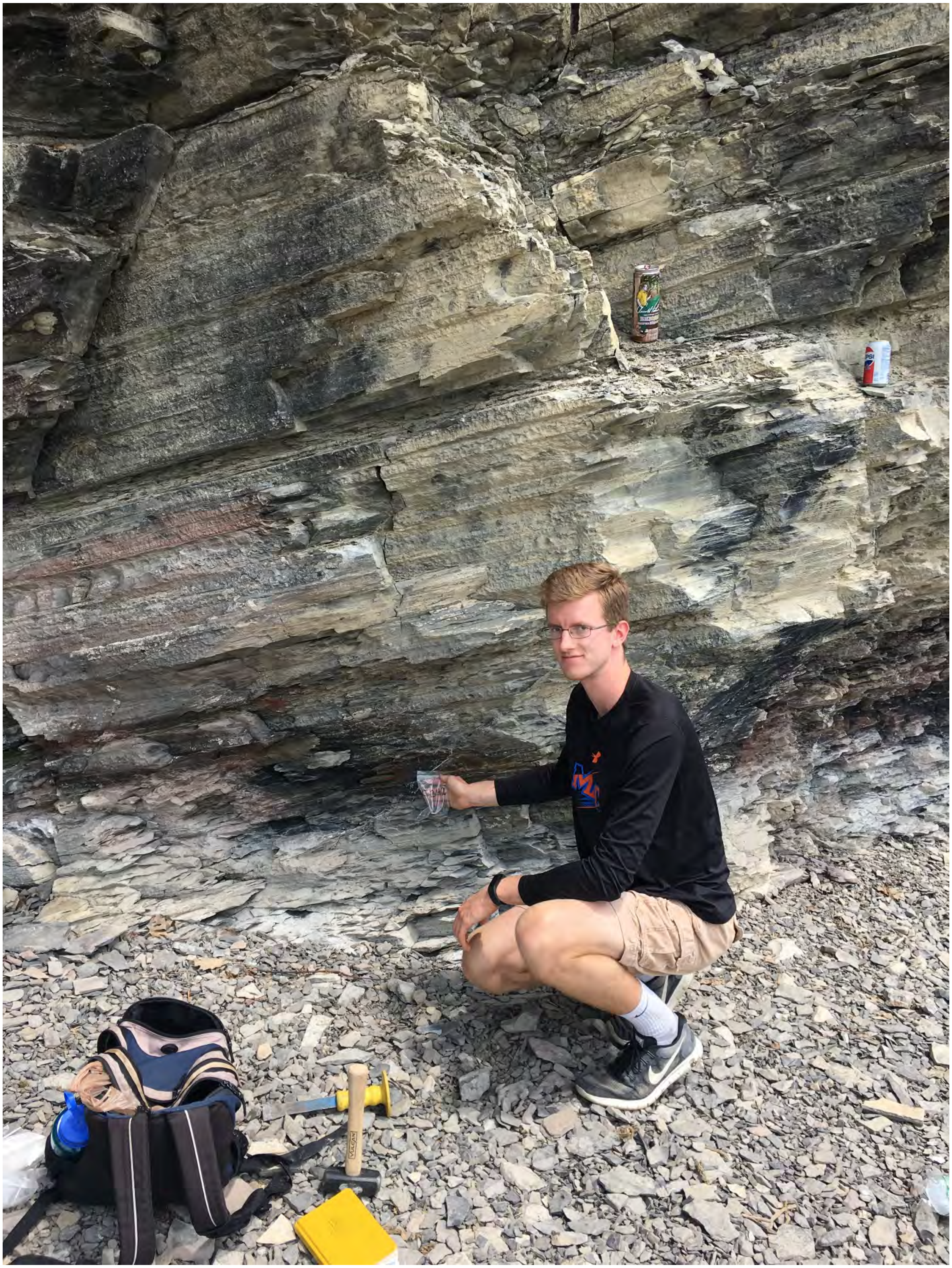

Lab Description:

C242: Circles show recon laser shots

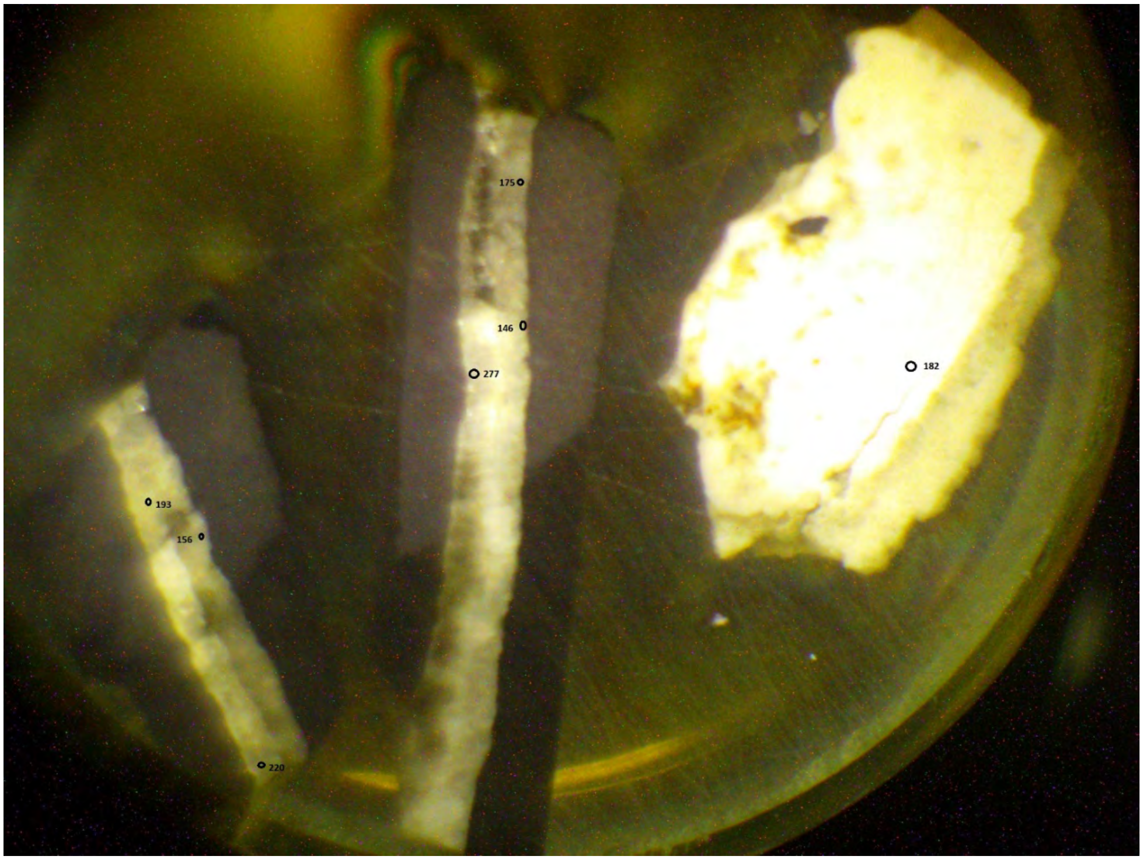

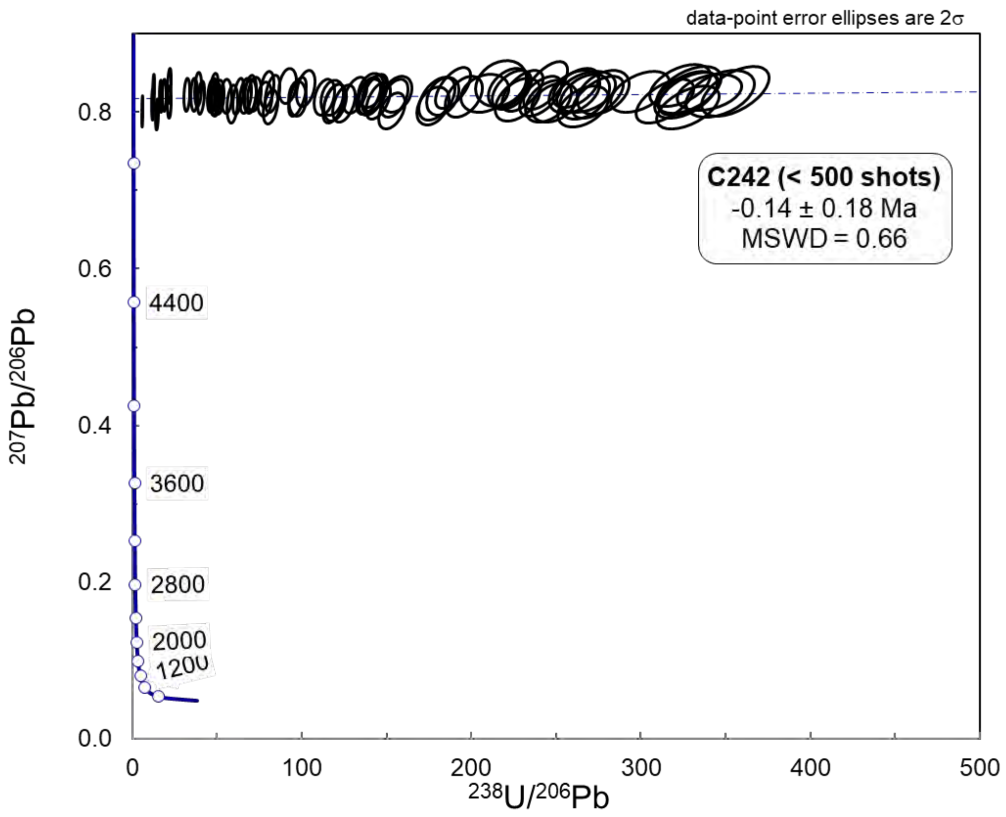
data-point error ellipses are 2σ
C242 (< 500 shots)
-0.14 ± 0.18 Ma
MSWD = 0.66
4400
3600
2800
2000
1200
0.0
0.2
0.4
0.6
0.8
0
100
200
300
400
500
207Pb/206Pb
238U/206Pb

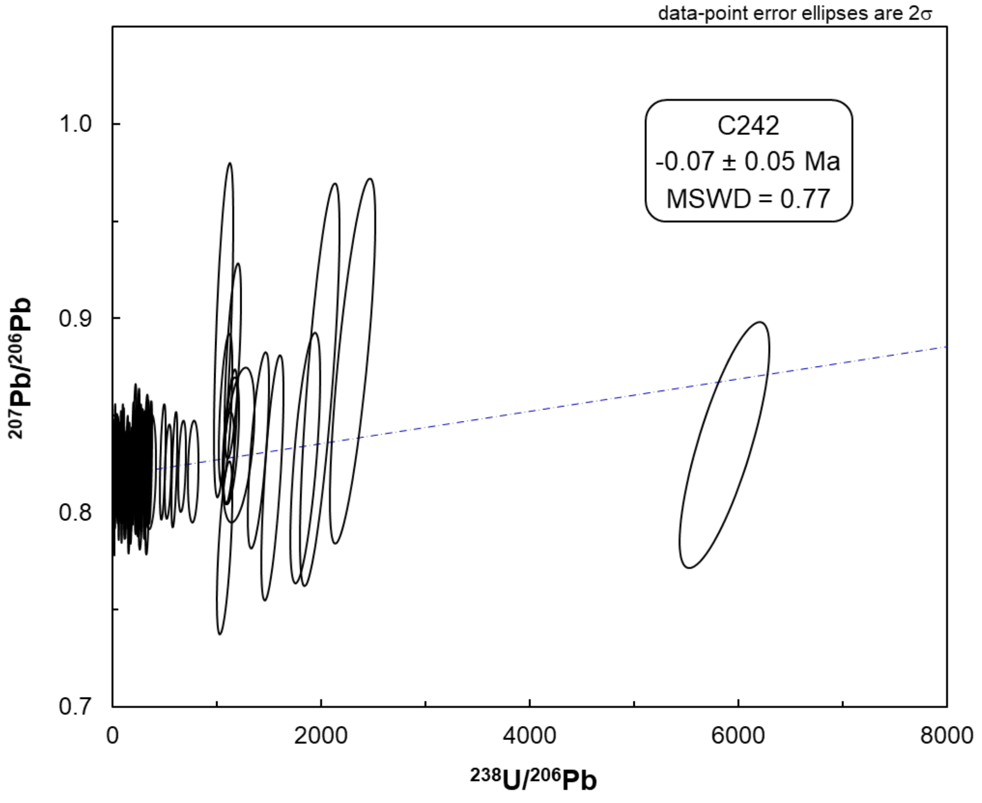
data-point error ellipses are 2σ
C242
-0.07 ± 0.05 Ma
MSWD = 0.77
0.7
0.8
0.9
1.0
0
2000
4000
6000
8000
207Pb/206Pb
238U/206Pb
