# Supplementary material for: Pace of passive margin tectonism revealed by U-Pb dating of fracture-filling calcite
Source: Nat Commun. 2022 Apr 12;13:1953. doi: 10.1038/s41467-022-29680-z (PMC9005700; doi:10.1038/s41467-022-29680-z)

17: Willsboro Quarry, New York (44.360009, -73.378998)

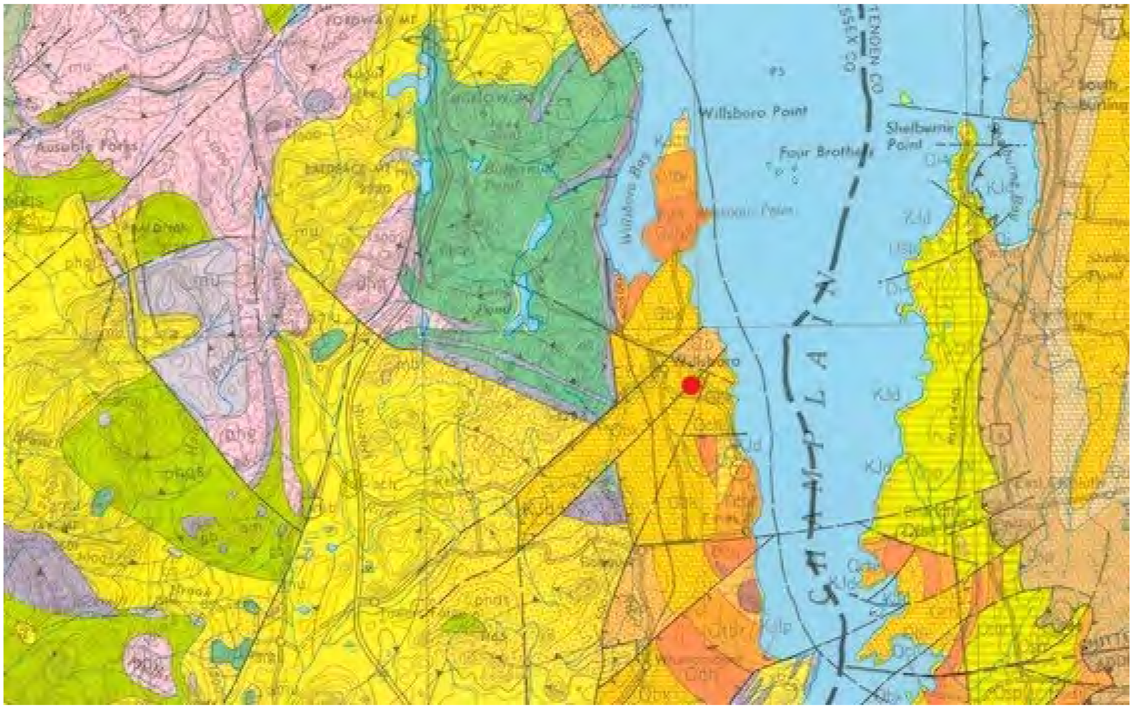

Figure 1: Sample located on Adirondack geologic map of Isachsen and Fisher, 1970.

<u>Failed samples from same site:</u> C246B,C,D,E,F

<u>Bedrock Unit</u>: Beekmantown Group

<u>Age</u>: **C246A**: 91.3 ± 1.5 Ma

Summary: This sample is located inside a stone quarry to which access was never granted. It was collected by students and never successfully relocated. It thus lacks a reliable strike and dip, and is not assigned a tectonic interpretation. Is taken from thin platey sub-vertical vein. Age appears to be high quality and single domain.

<u>Field Descriptions</u>:

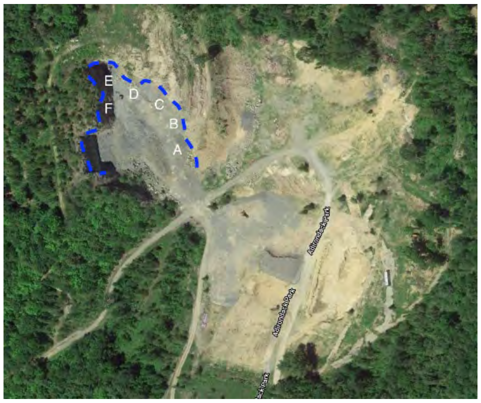

Figure 2: approximate locations of samples C246A-F within the stone quarry

C246A:

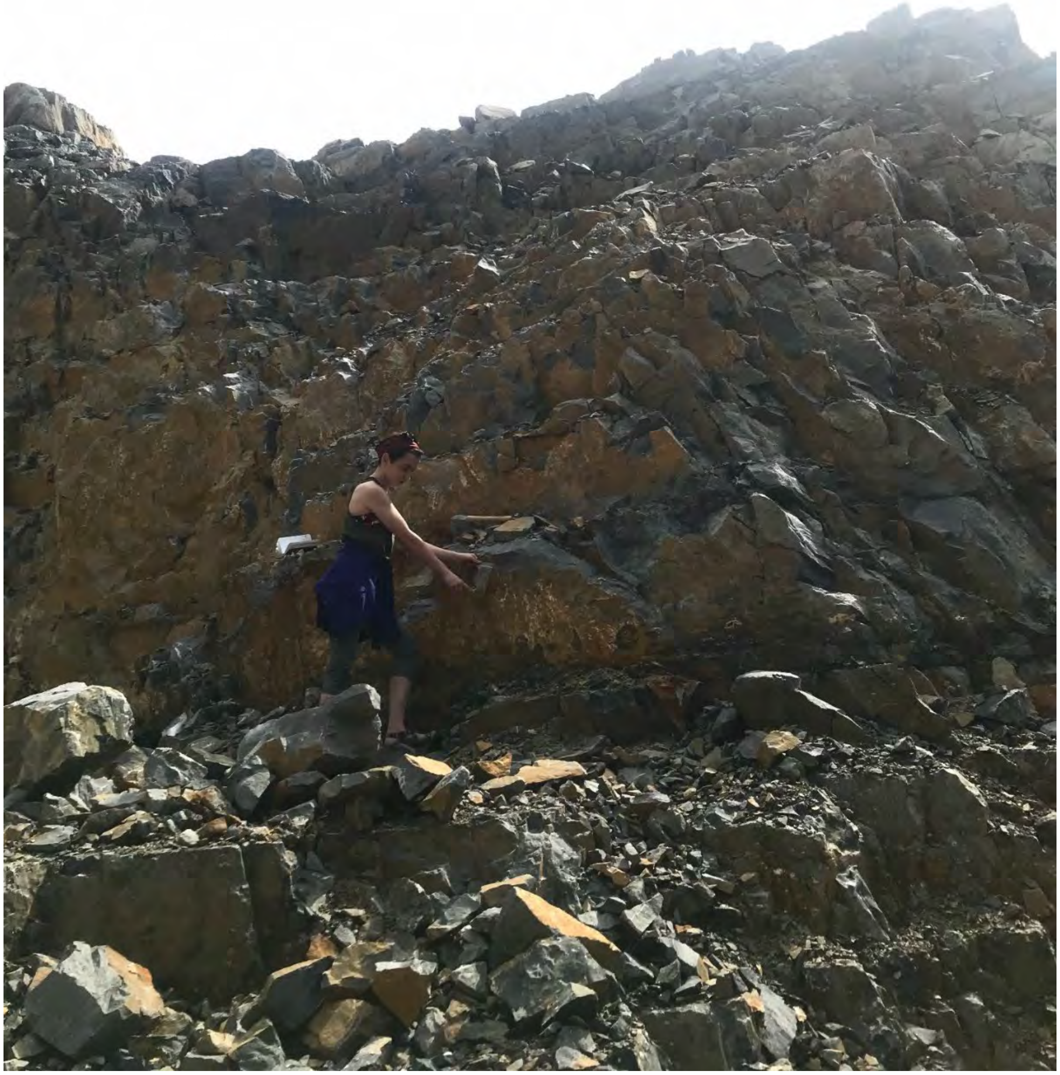

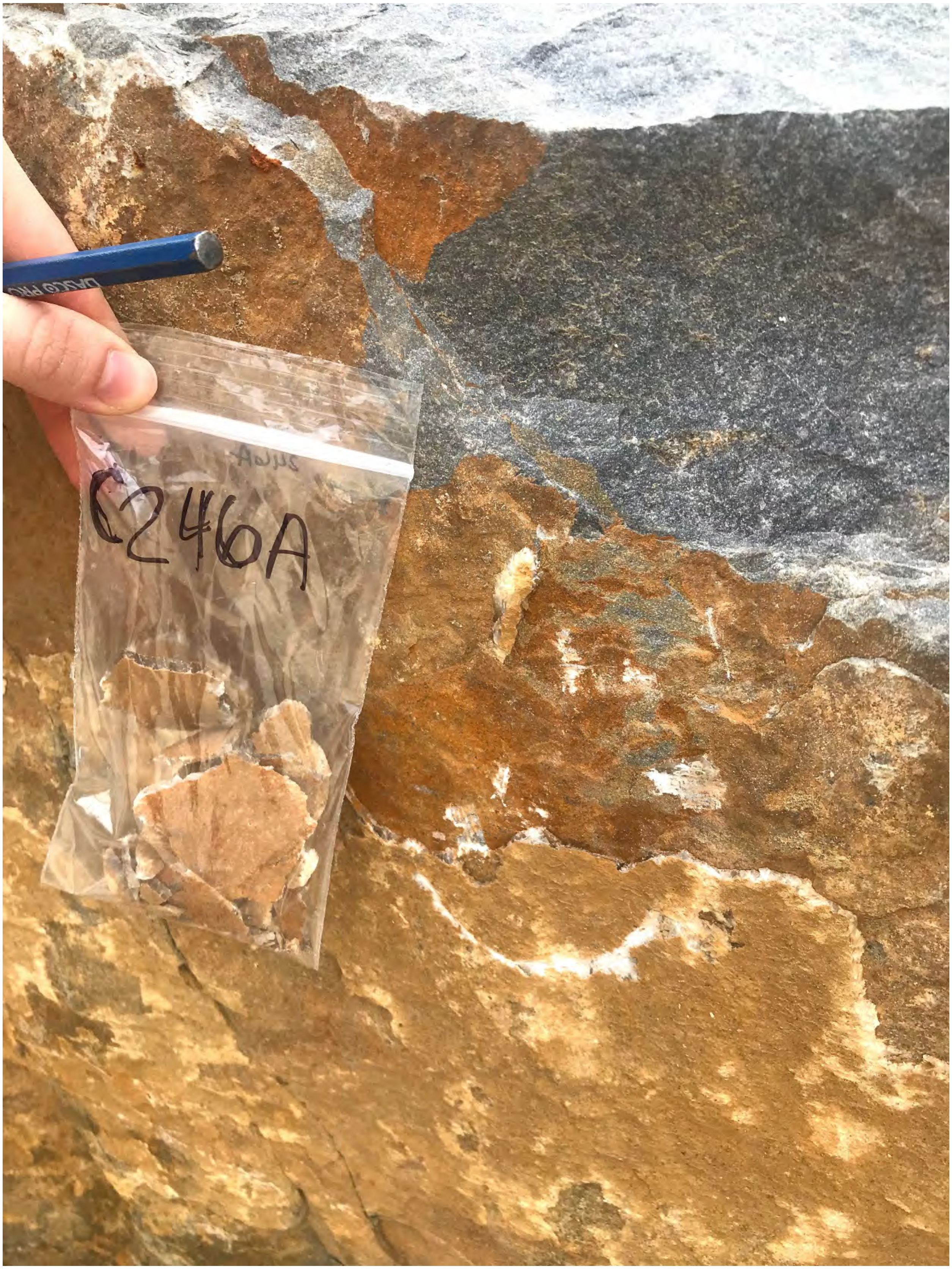
C246A

Lab Description:

C246A: Circles denote areas of interest for dating, not laser shots

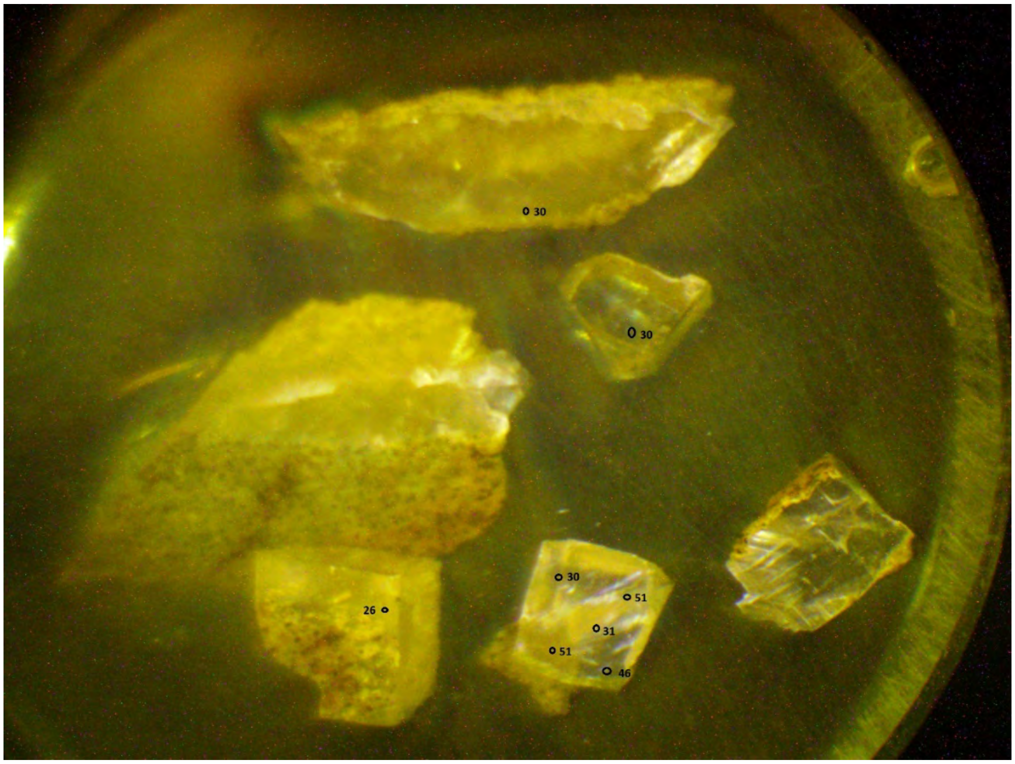

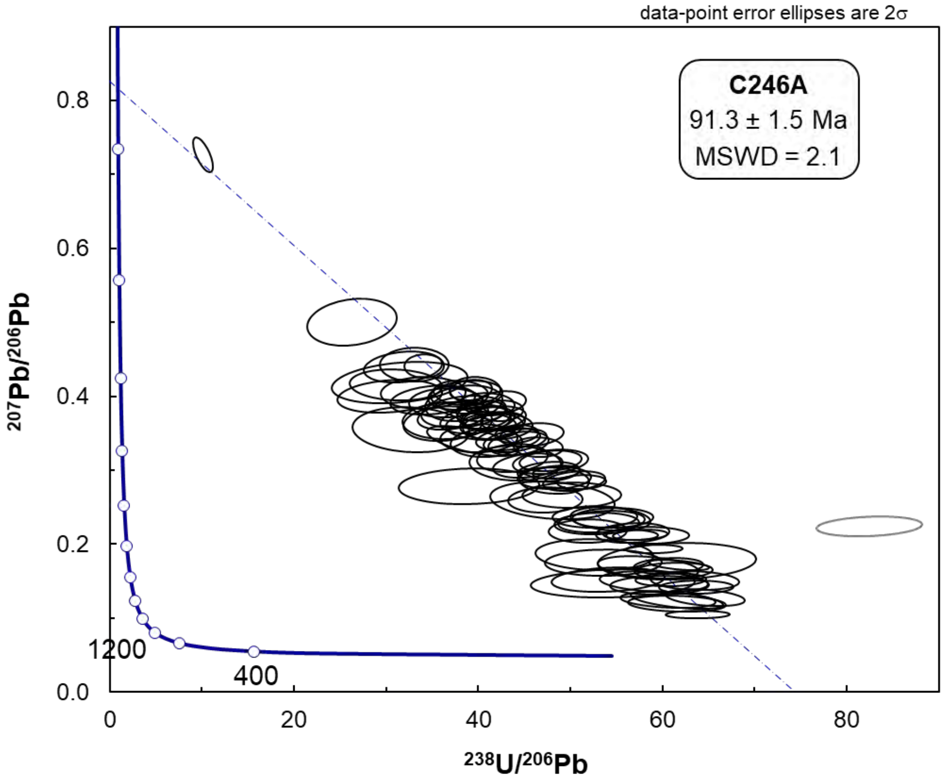

Supplement: Supplementary file 6 — Source Data [file 41467_2022_29680_MOESM6_ESM.zip › 1.zip!/appendix2_site_summaries_1to20/17_willsboro_quarry_final.pdf]
