# Supplementary material for: Pace of passive margin tectonism revealed by U-Pb dating of fracture-filling calcite
Source: Nat Commun. 2022 Apr 12;13:1953. doi: 10.1038/s41467-022-29680-z (PMC9005700; doi:10.1038/s41467-022-29680-z)
Supplement: Supplementary file 6 — Source Data [file 41467_2022_29680_MOESM6_ESM.zip › 1.zip!/appendix2_site_summaries_1to20/18_port_henry_campground_final.pdf]

18: Port Henry NY @ Crag Harbor Property (44.05656, -73.4509)

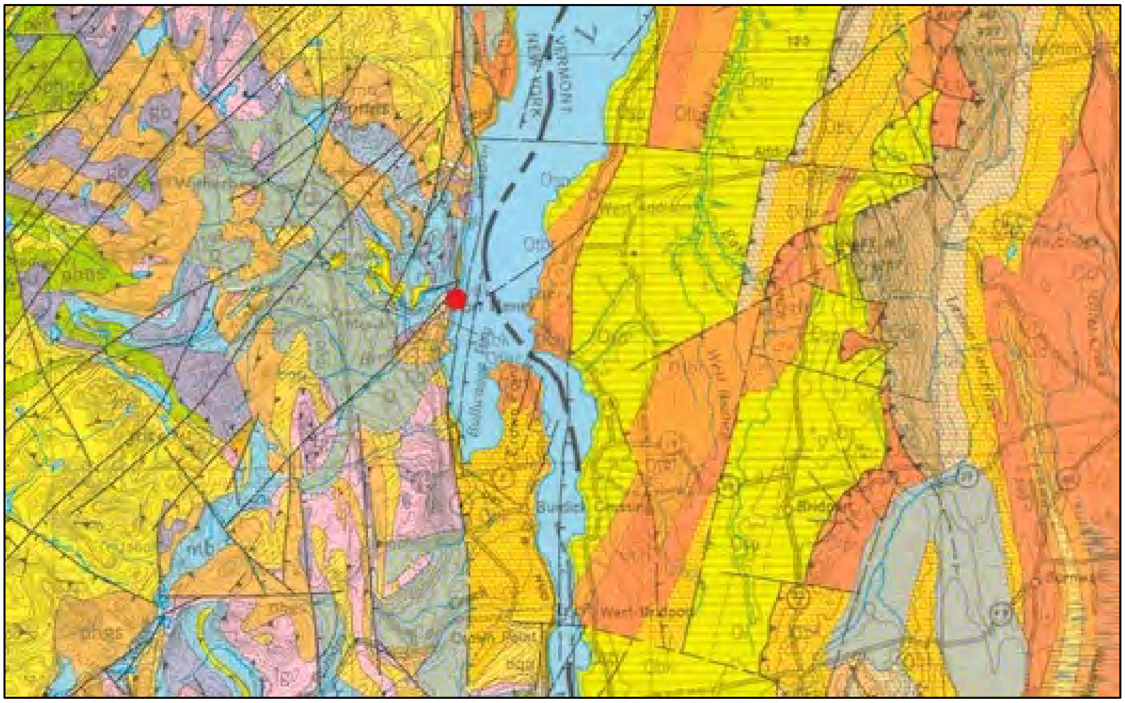

Figure 1: Sample located on Adirondack geologic map of Isachsen and Fisher, 1970.

Failed samples from same site: C254C(?), C255, C260, C261, C262

Bedrock Unit: Ticonderoga Formation/Dolostone (CAti)

Ages: **C254A**: 103 ± 1.9 Ma & 521 ± 26 Ma **C254B**: 90.1 ± 6.2 Ma **C254D**: 438 ± 12 Ma
C256: 42.3 +/-6.0 **C257**: 40.7 ± 2 Ma **C258**: 443 +/- 16 Ma **C259**: 75 ± 0.9 & 46.8 ± 4.7

Summary: This site is located at Crag Harbor and is described in detail in the McHone, 1987 DNAG article. Access is by foot by permission of land owners Andre and Denise Palmer (curly317@yahoo.com), residents of New Haven, VT. This site sits in the hanging wall of a large, southeast-dipping, extensional fault that strikes ~50° and bounds the north side of Crag Harbor. This site has been enigmatic because it produced a suite of low quality ages that are not commonly observed elsewhere in the Champlain Valley. For example, three samples at this cite record evidence for an Eocene crystallization event. Many of the veins show multiple U-Pb age components suggesting the site has experienced multiple episodes of tectonism and calcite crystallization. One explanation is that the unique NE-SW striking geometry of the fault zone may have controlled the local stress field and thus the geometry of the minor hanging wall fractures dated in this study. Most of the successfully dated samples are from the Ticonderoga Dolostone along the railroad tracks south of the harbor. All of the Cenozoic ages are of low quality, with several samples that show multiple age domains whereas others (C256B +C257) show only a few shots with measurable ratios, which define unusual (but reproducible!) ages of ~42 Ma. None of the dated samples at this site contain evidence for slip, so all are interpreted as mode 1 fractures assisted by varying degrees of fluid overpressure.

Samples C258 and C254D record events during the Taconic Orogeny (~440 Ma), which appears to be associated with a set of discontinuous N-S trending veins. Vein C254D is sub-vertical with a distinct 'lenticular' shape; wide in the middle and tapering at the top and bottom, suggestive of a 'mobile hydrofracture' as described by Bons et al., 2012. This suggests a role of fluid overpressure during this Taconic-aged event. This event is likely widespread across the Champlain Valley and is comparable in timing and orientation to the event record on Carleton's Prize Island (Sites 7b, 23, and 43).

Next, sample C254A is a sub-vertical, ultra-planar, ~ 50 cm long dilational vein formed interpreted as a mode 1 fracture formed under a N-S $\sigma 3$ stress at ~ 103 Ma. The U-Pb data defines multiple age isochrones, including a ~521 Ma age arising from accidental shots on the adjacent host rock. Similarly, sample C254B is also a sub-vertical, planar, dilational vein interpreted as a mode 1 fracture formed under a roughly N-S $\sigma 3$ stress regime at ~ 90 Ma. The age is a multi-domain isochron in which an older isochron can be dientifed and several younger shots are also excluded from the 90.1 Ma age estimate.

Sample 259 is a thin (~1-2mm), planar, fracture-plating vein with a primary age of ~75 Ma and a second episode of crystallization at ~47 Ma. Recognizing the isochron plot shows a wide spread of measured ratios, the 75 Ma age is defined as the linear array of shots defining the oldest age. We interpret this as a mode 1 vein formed under an ENE-WSW $\sigma 3$ stress at 75 Ma and classify the 47 Ma event as secondary crystallization with unknown kinematics.

Samples C256 and C257 record a crystallization event around 42 Ma. However, in each sample this age component is defined by very few shots leaving uncertainty on the extent to which the ~42 Ma age is representative of the entire calcite vein. The texture of the calcite shows interlocking zones of transluscent and clear calcite suggestive of recrystallization. We are thus unable to reliably link the vein age to formation of the structures and leave open the possibility that these ages record a secondary recrystallization event. If the U-Pb age of C257 Ma is interpreted as crystallizing during a mode-1 dilation event it would record NNE-SSW extension, but such an inference is highly speculative. However, a nearly identical stress regime at ~43 Ma is recorded at site 24 (Providence Island East), suggesting this Eocene event may warrant further study.

Field Observations:

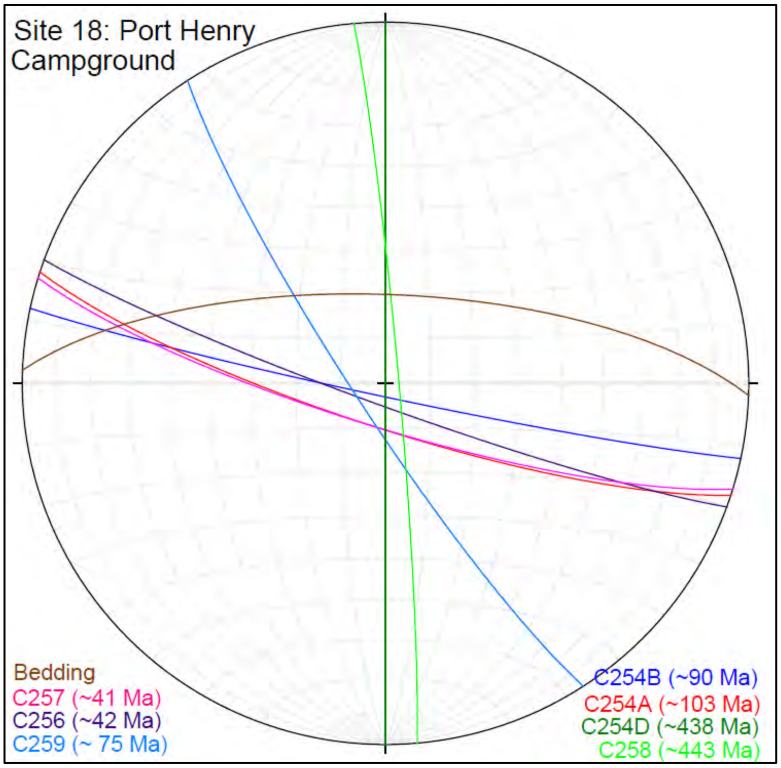

Figure 2: Stereonet plot showing orientations of dated structures at the Port Henry site

C254A:

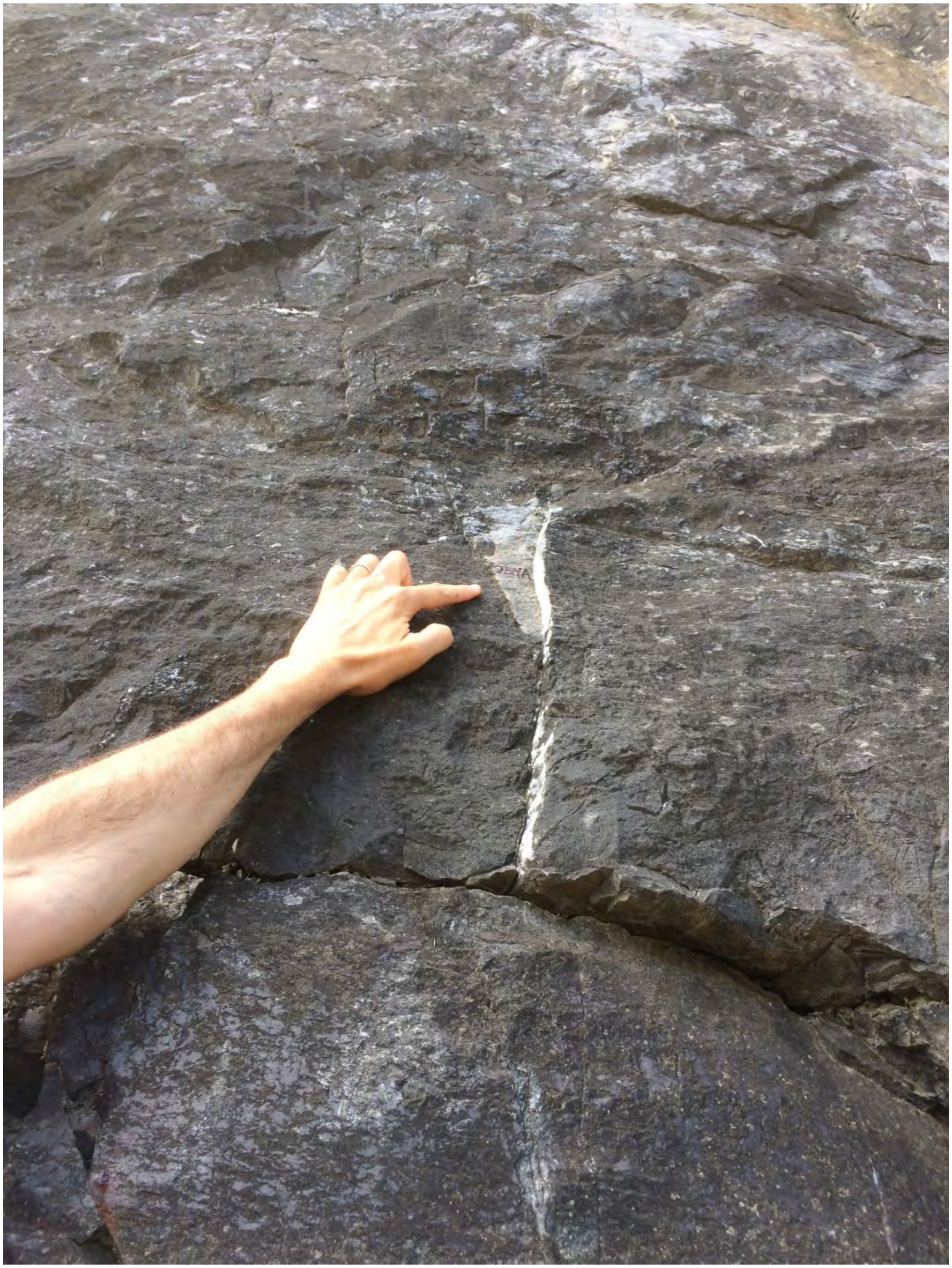

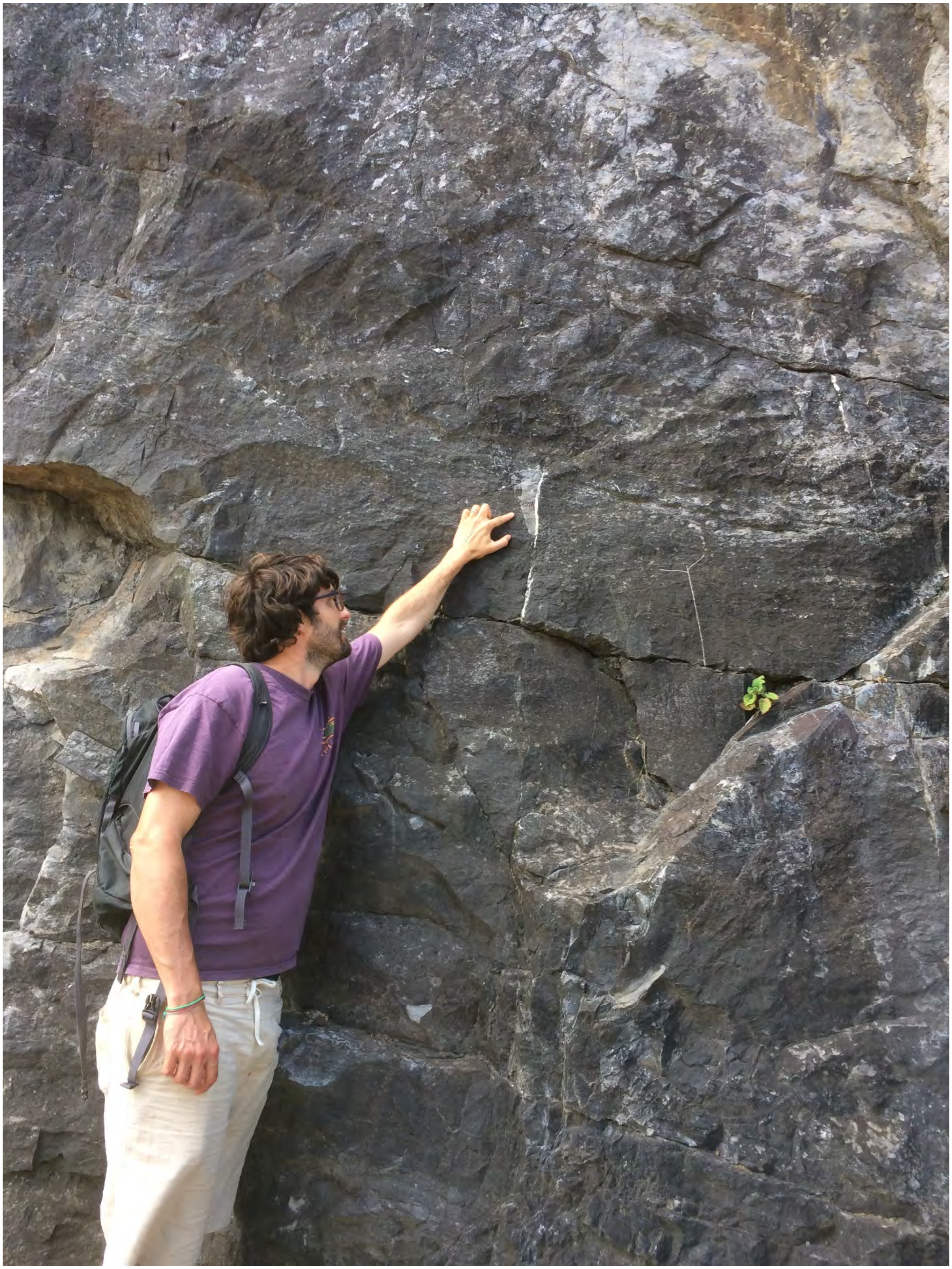

C254B+D:

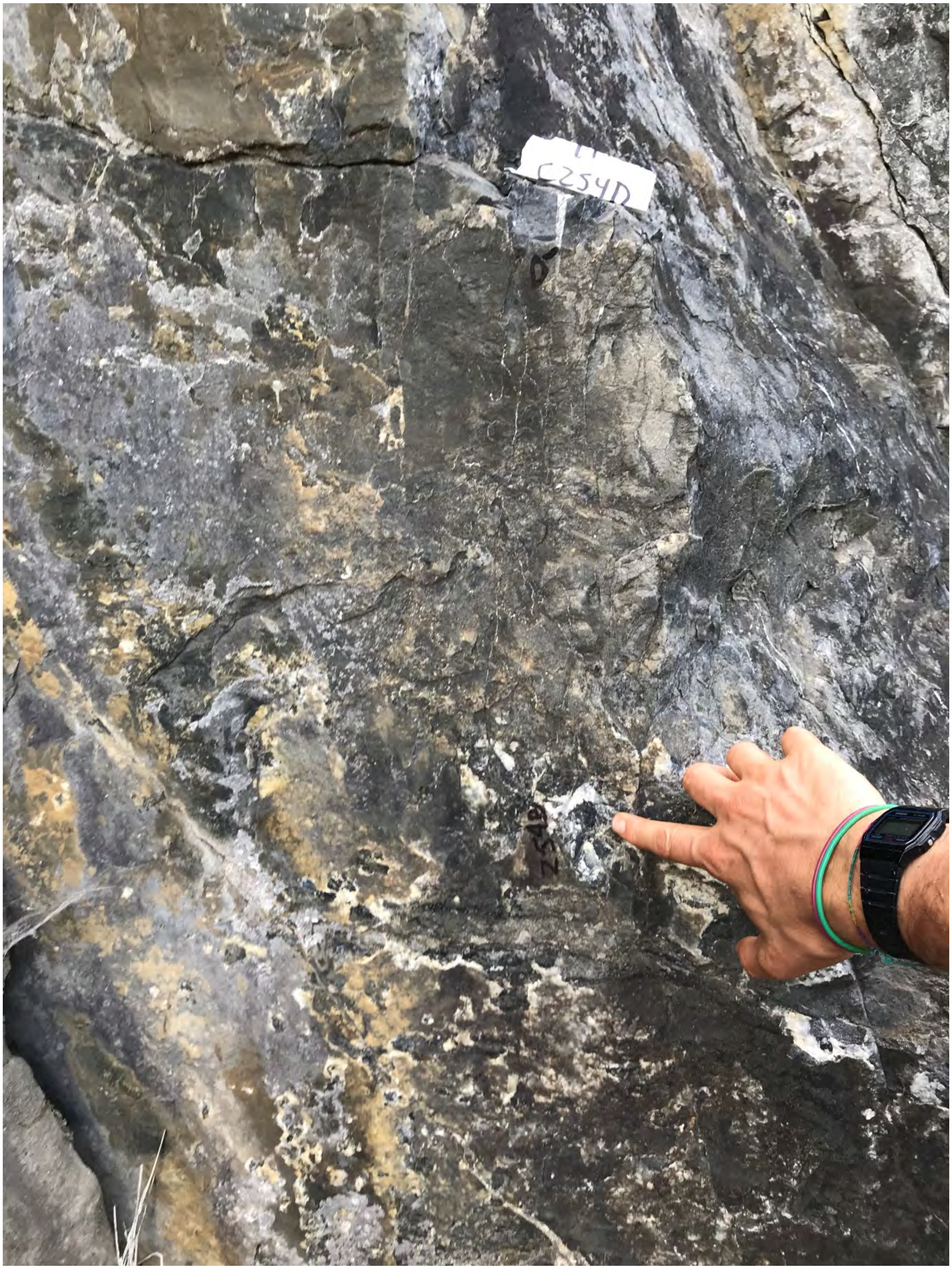

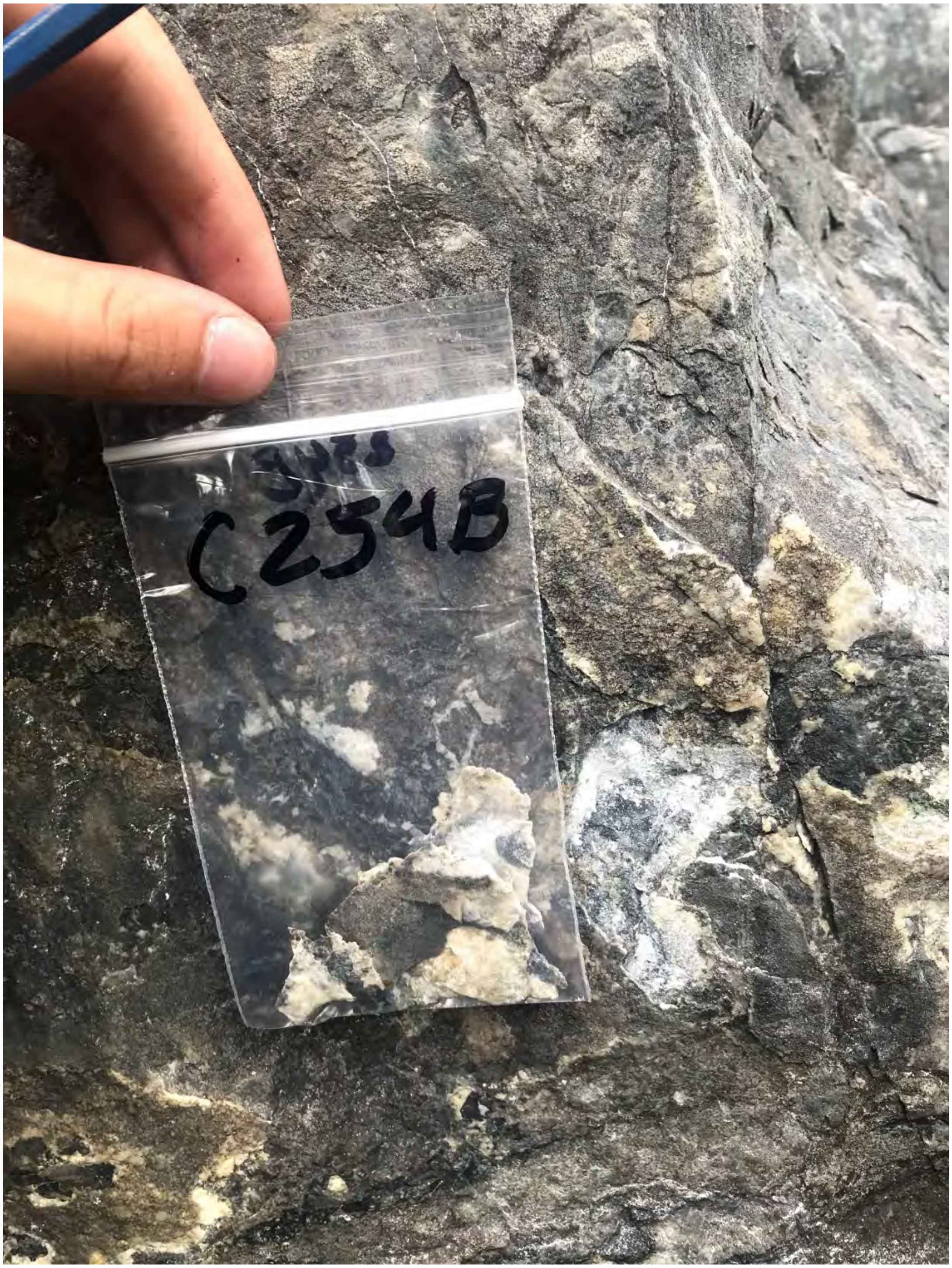
C254B

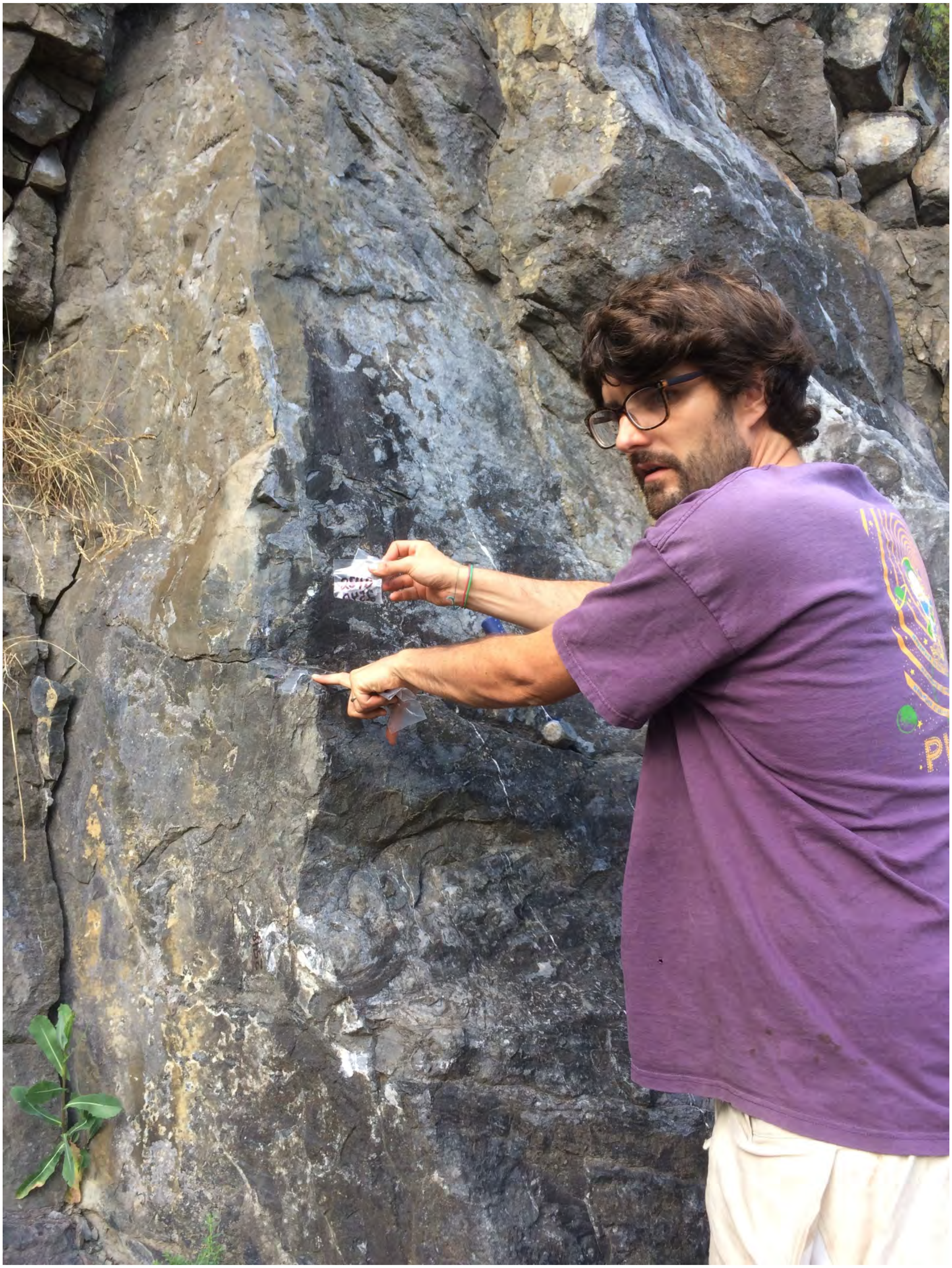

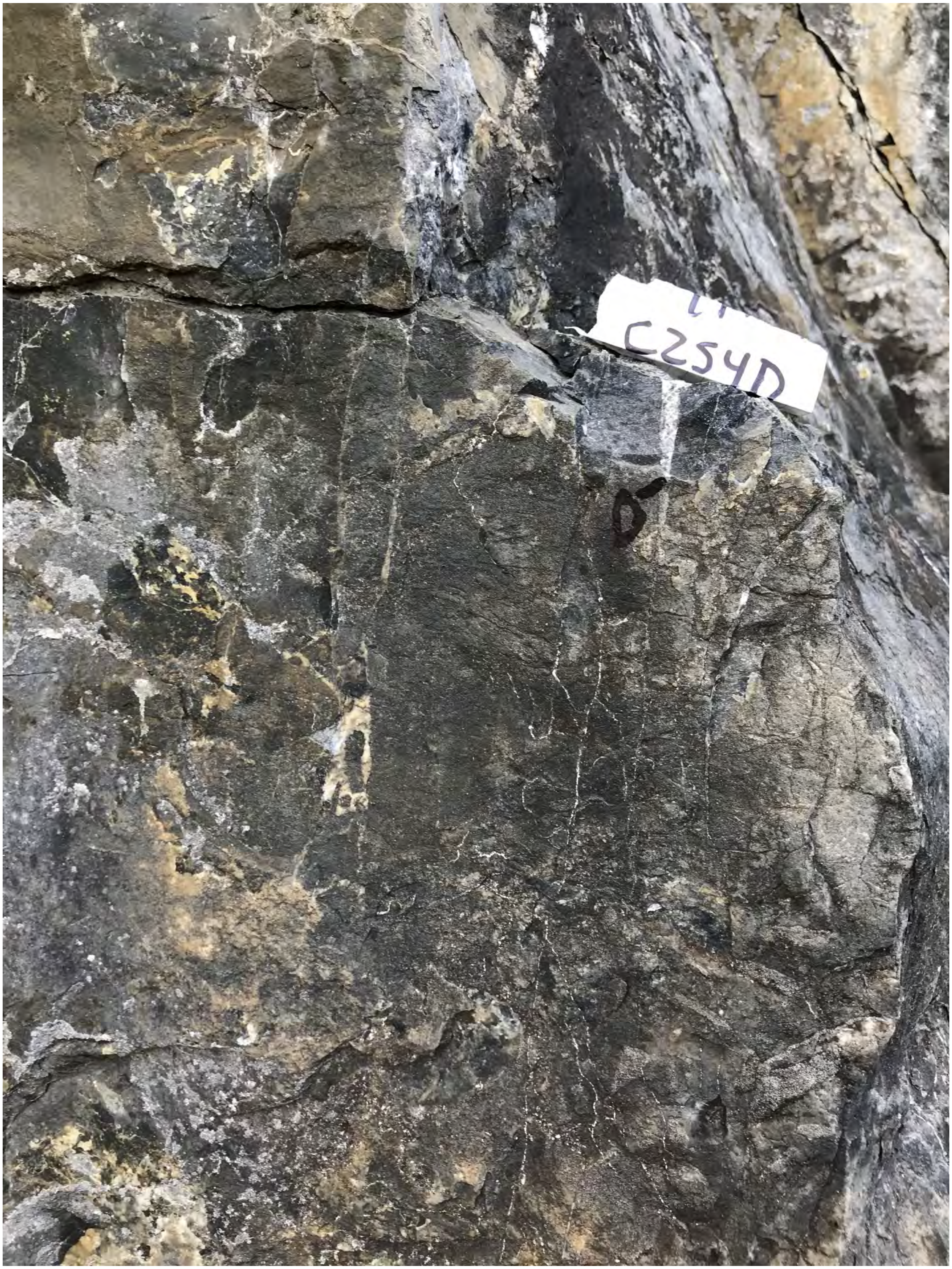
C254D

C256:

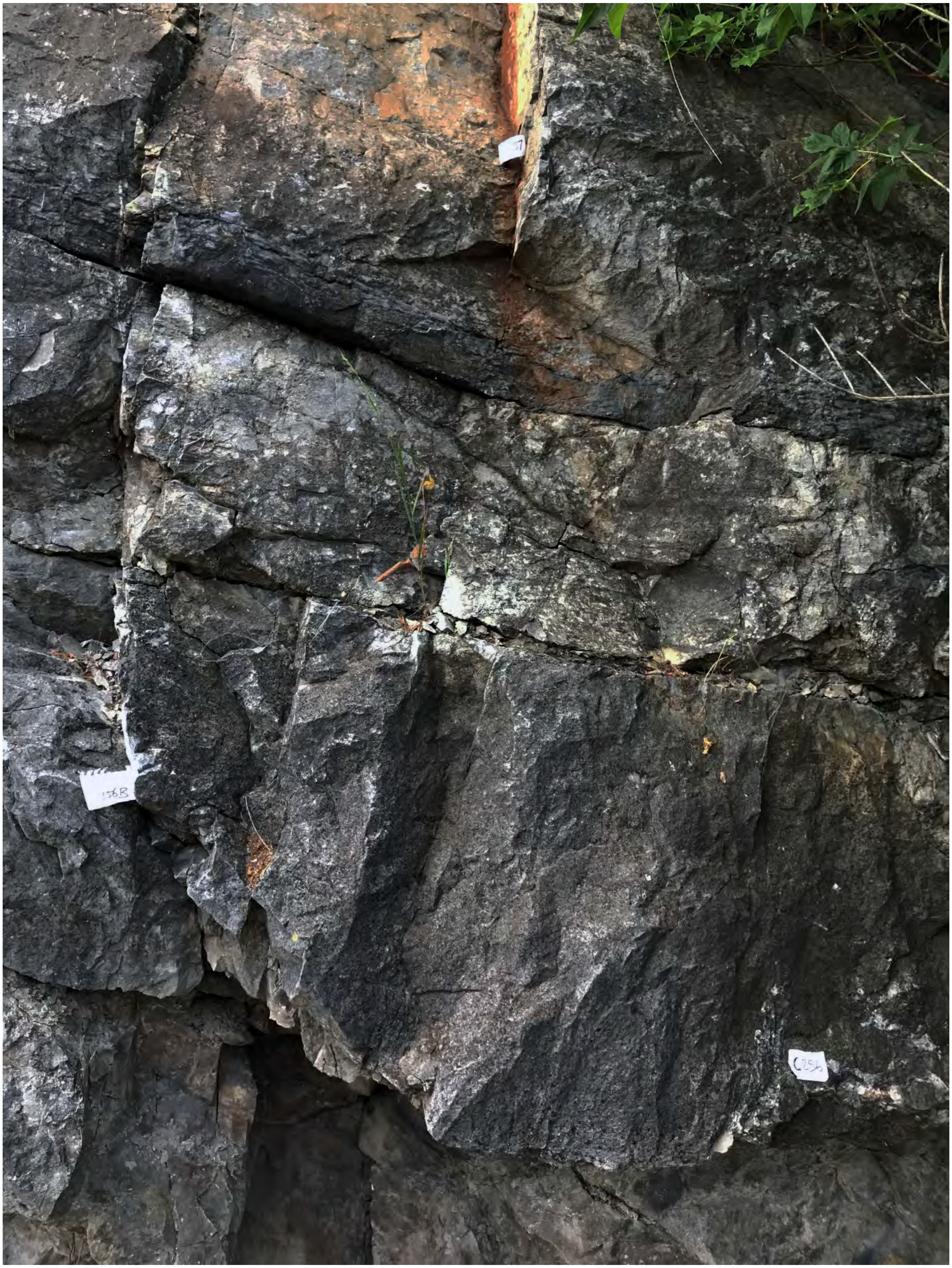

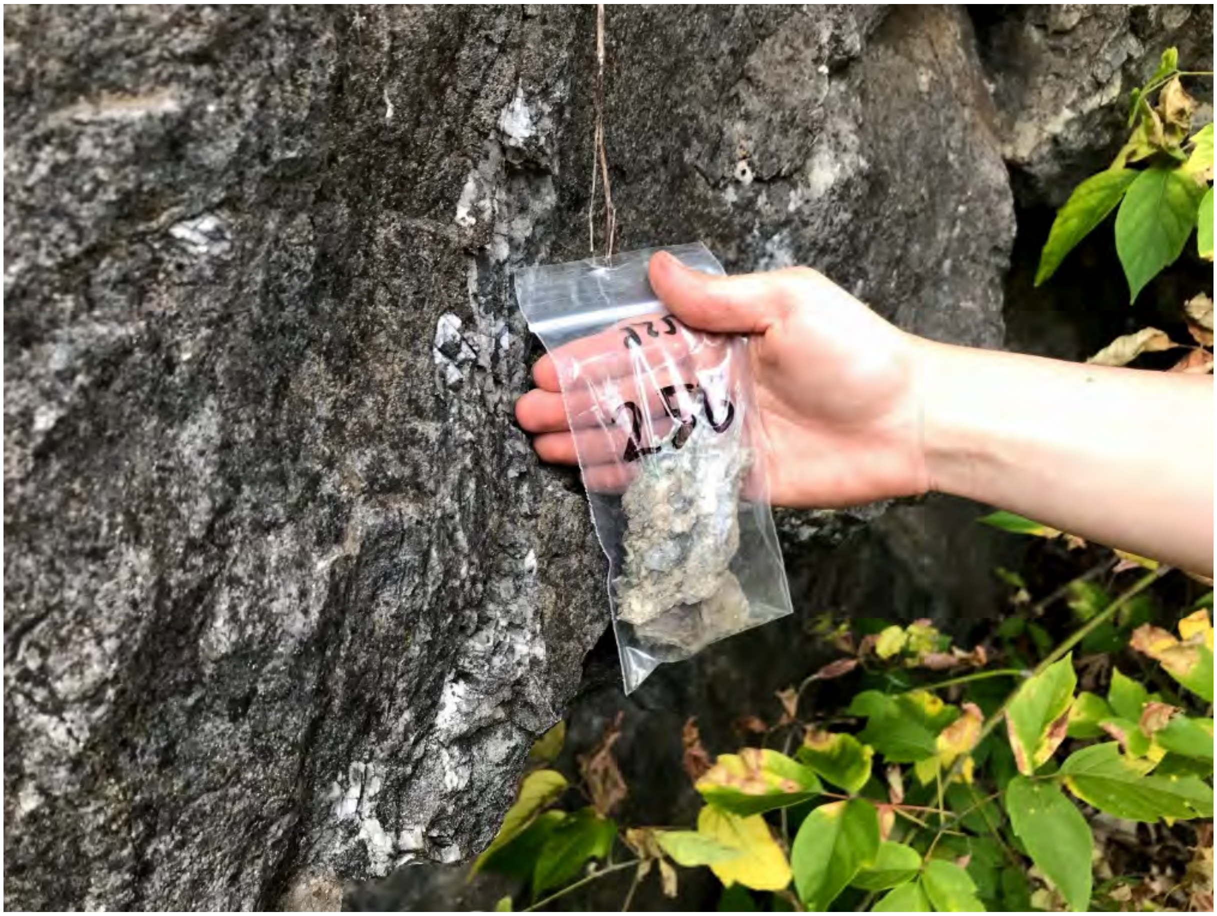

256:

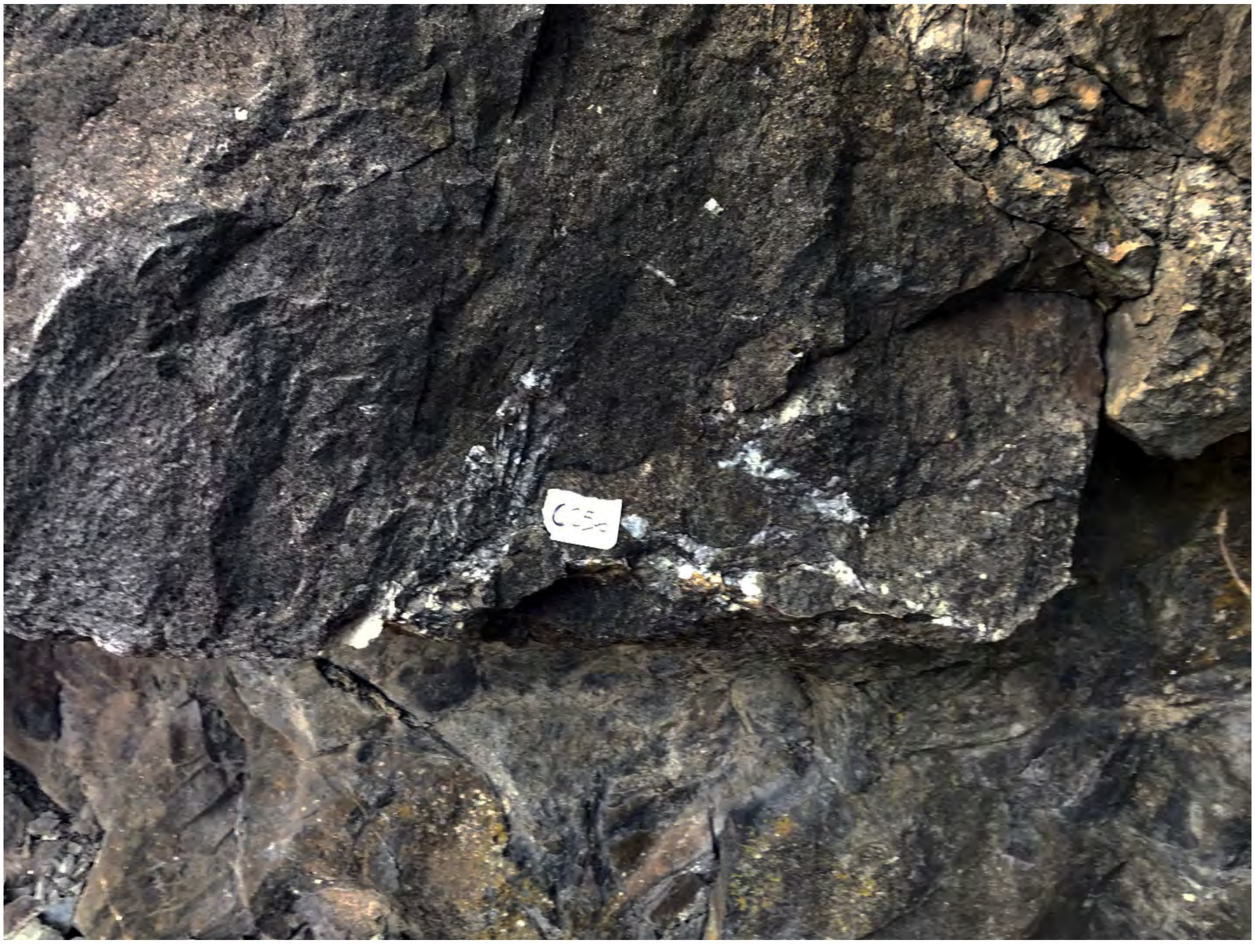

C257:

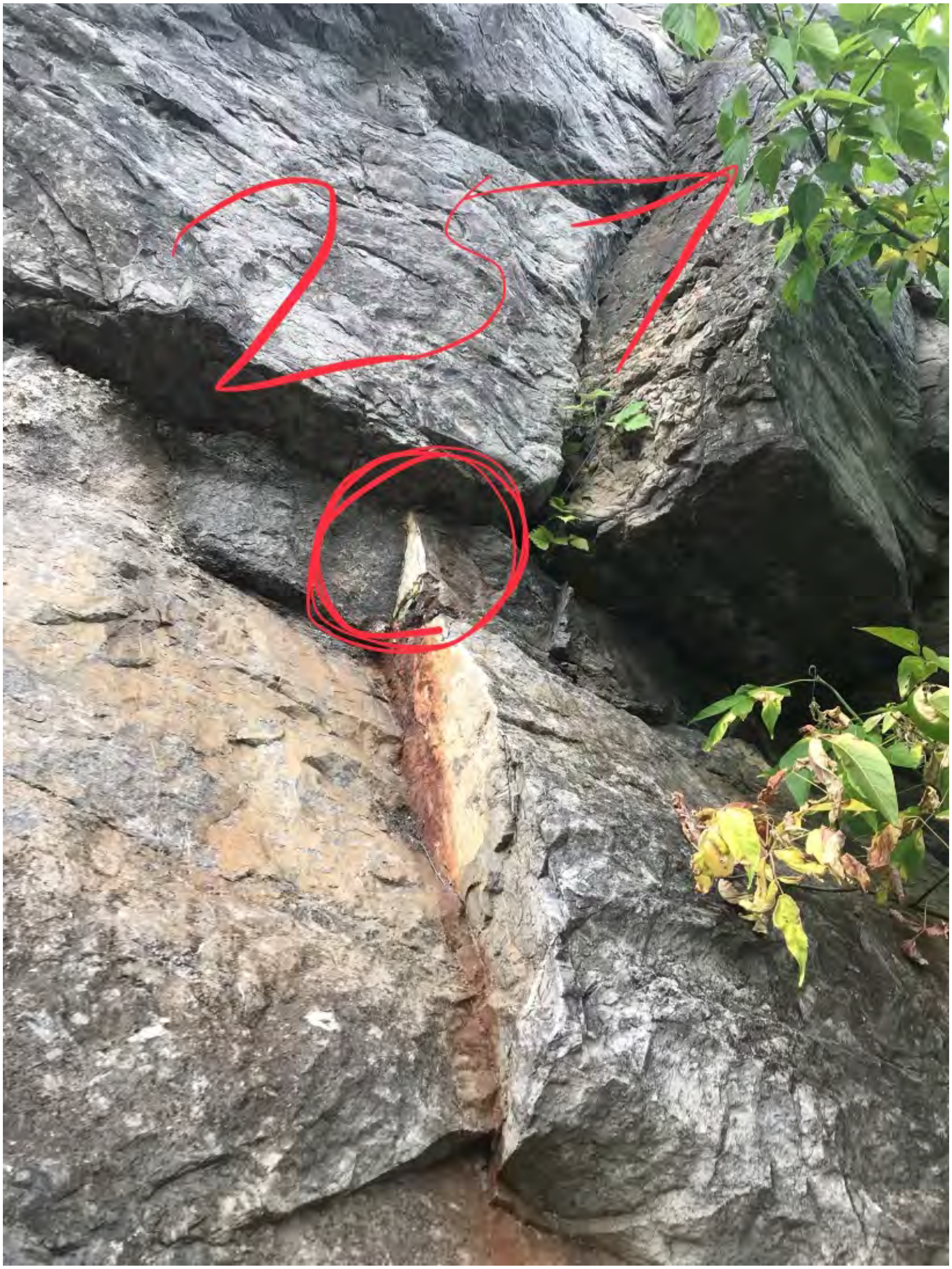

C258:

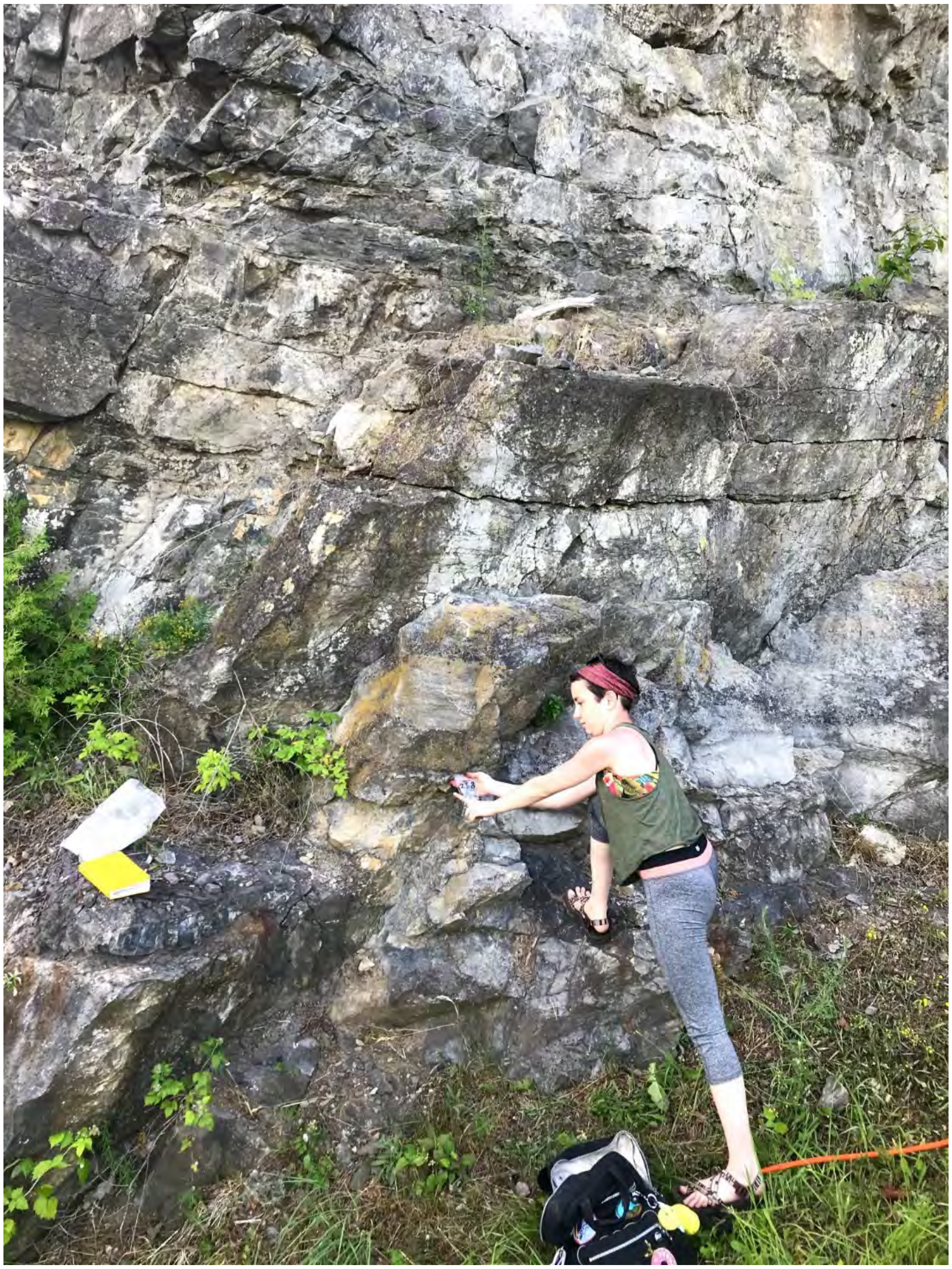

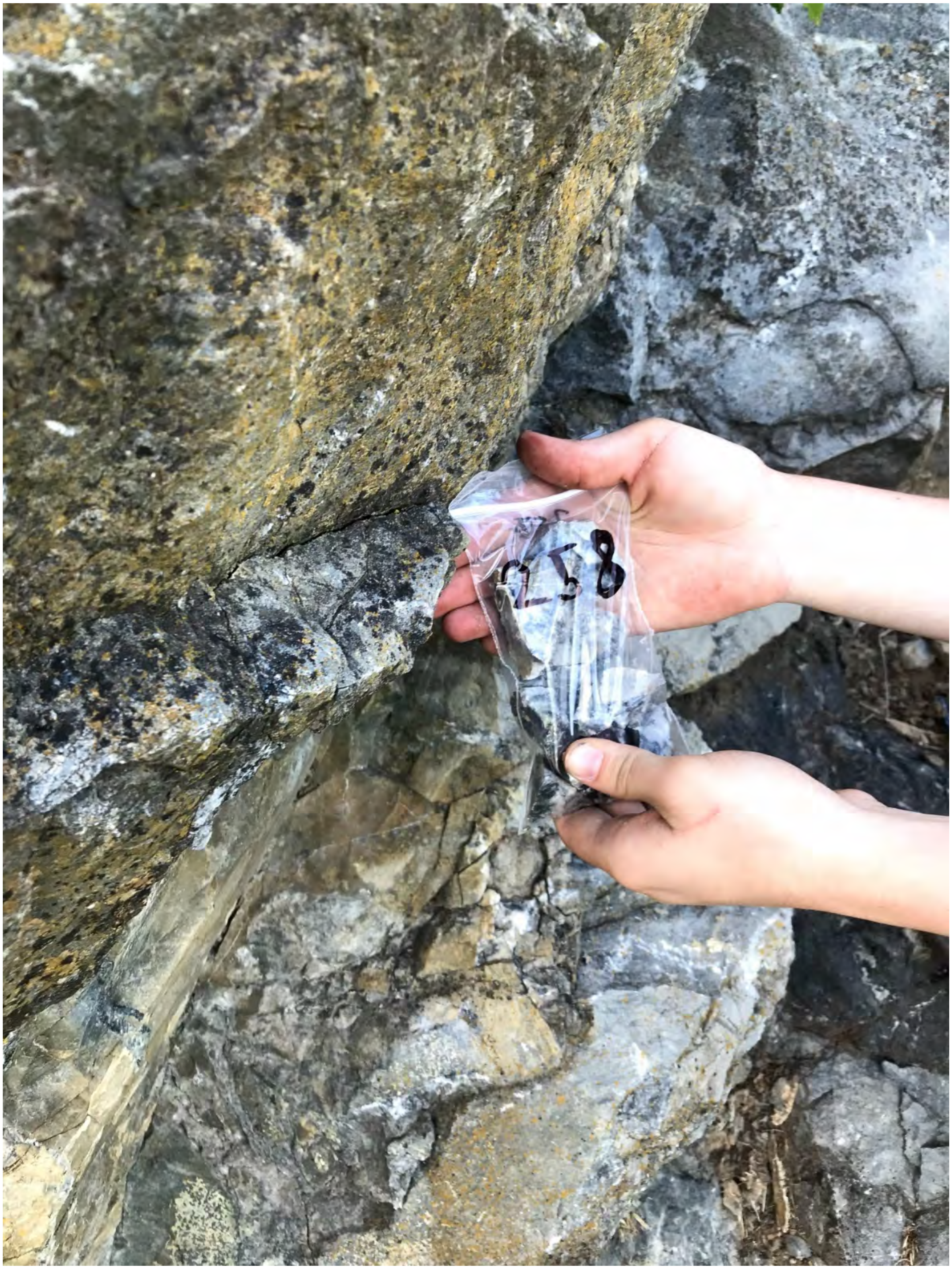

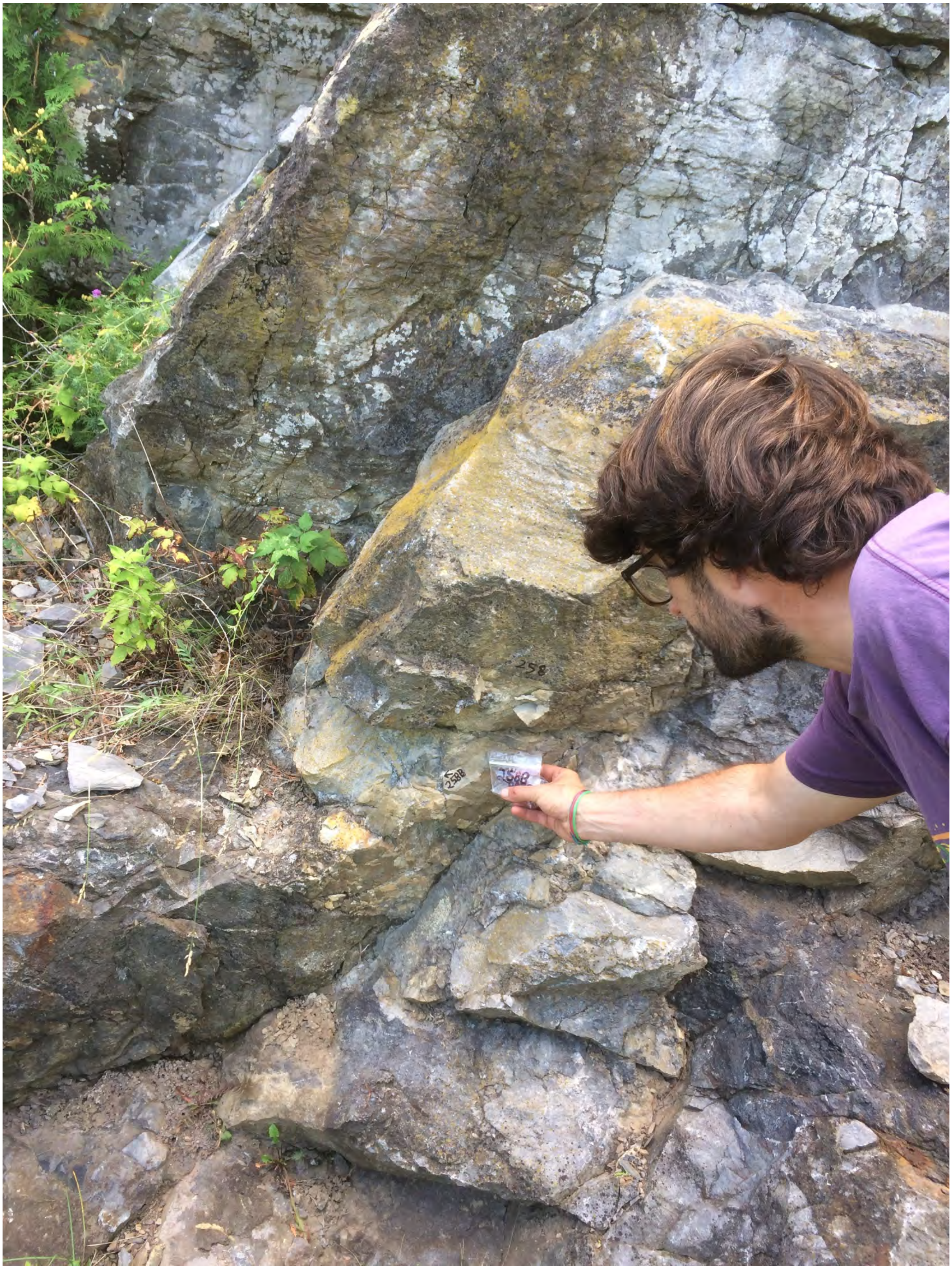
258
258B

C259:

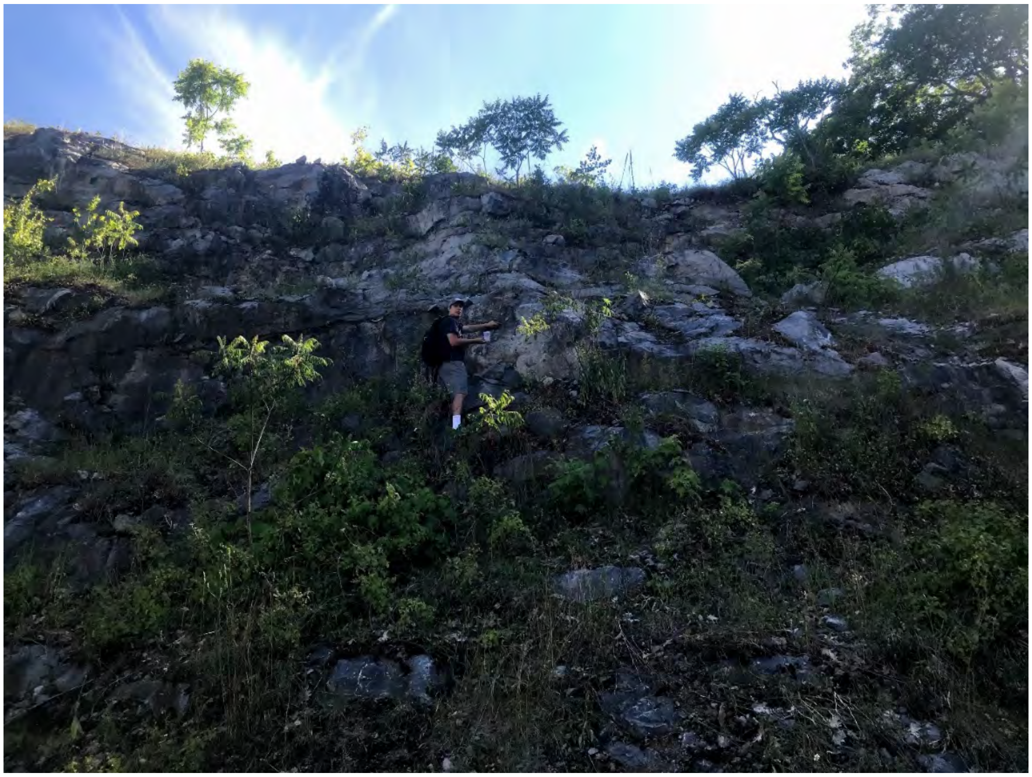

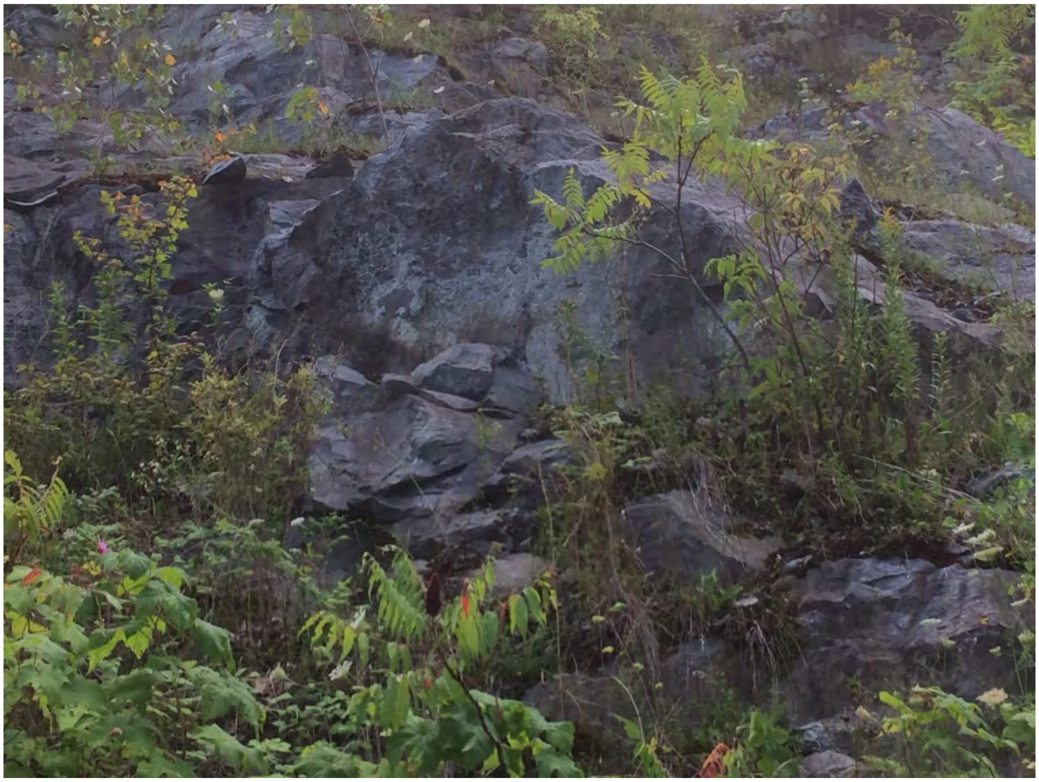

Lab Observations:

C254A: Circles show areas targeted for dating, not laser shots

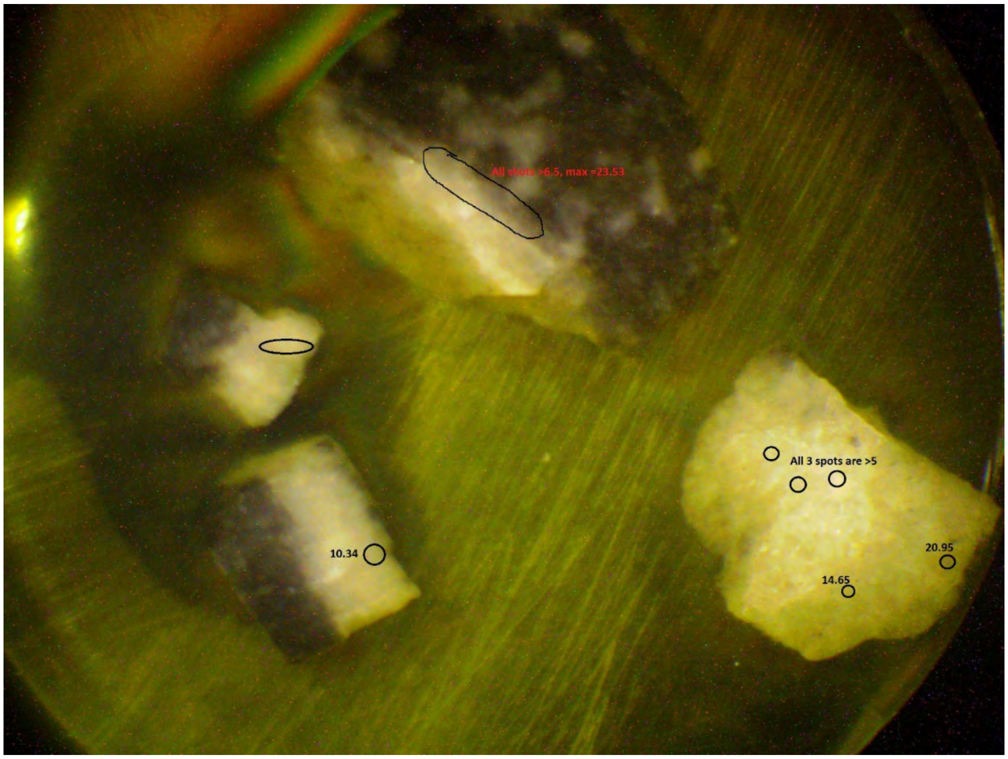

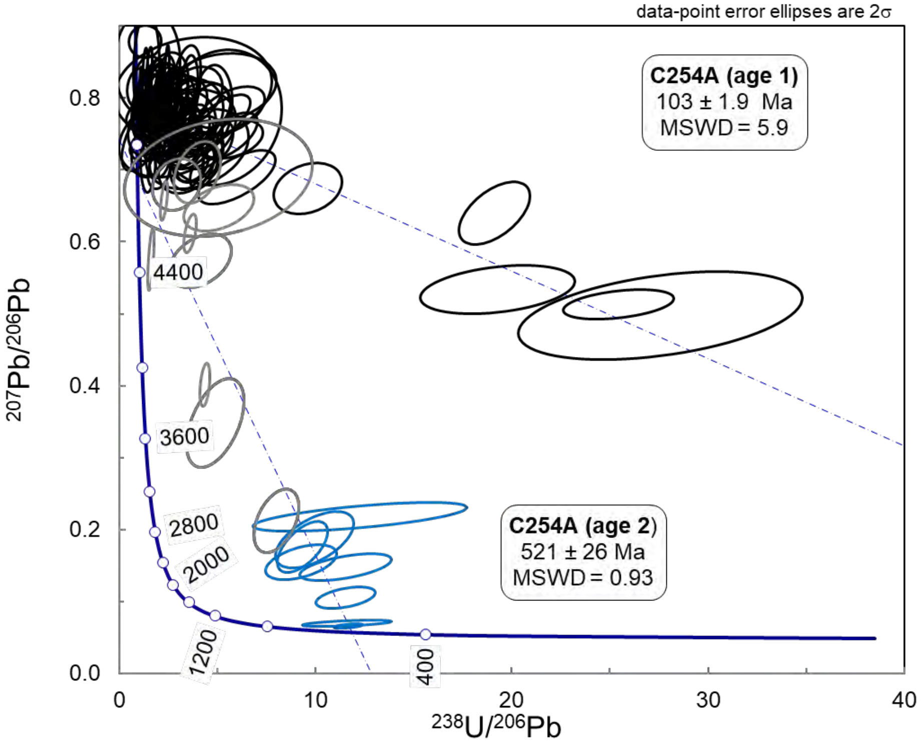

C254B: Circles show areas targeted for dating, not laser shots

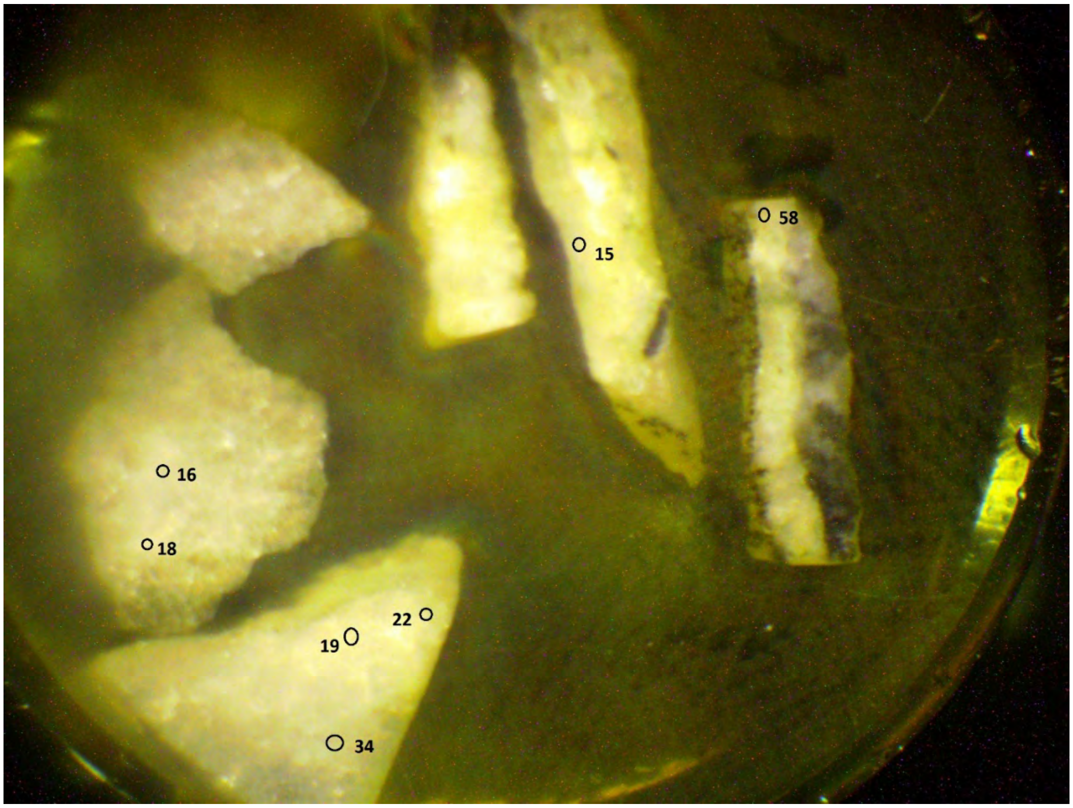

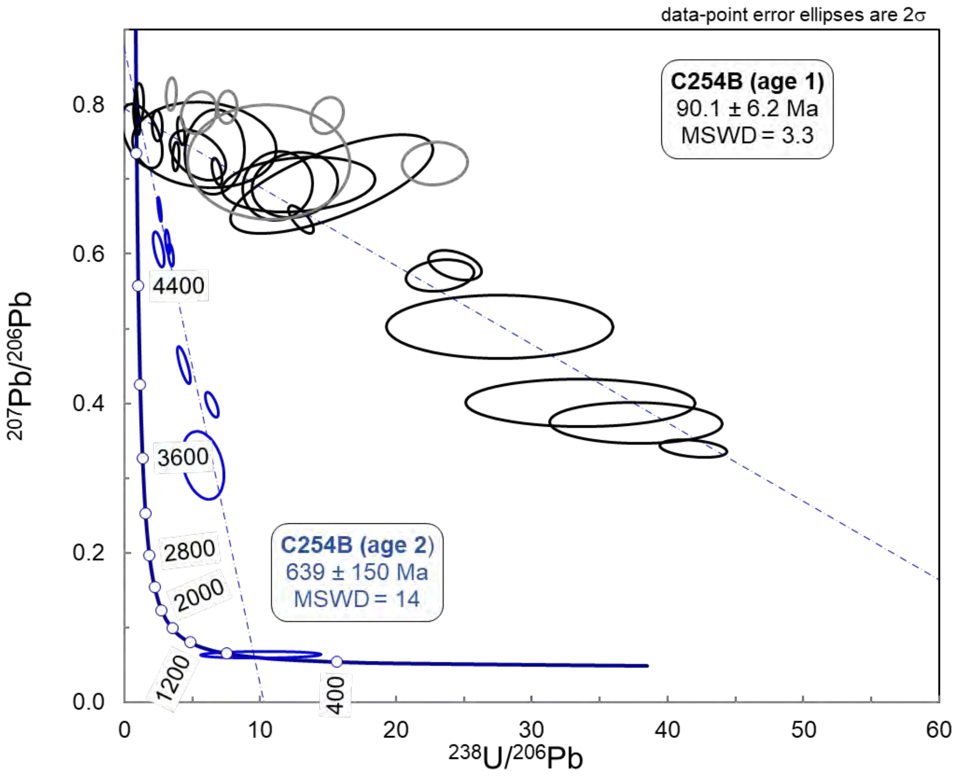

C254D: Circles show areas targeted for dating, not laser shots

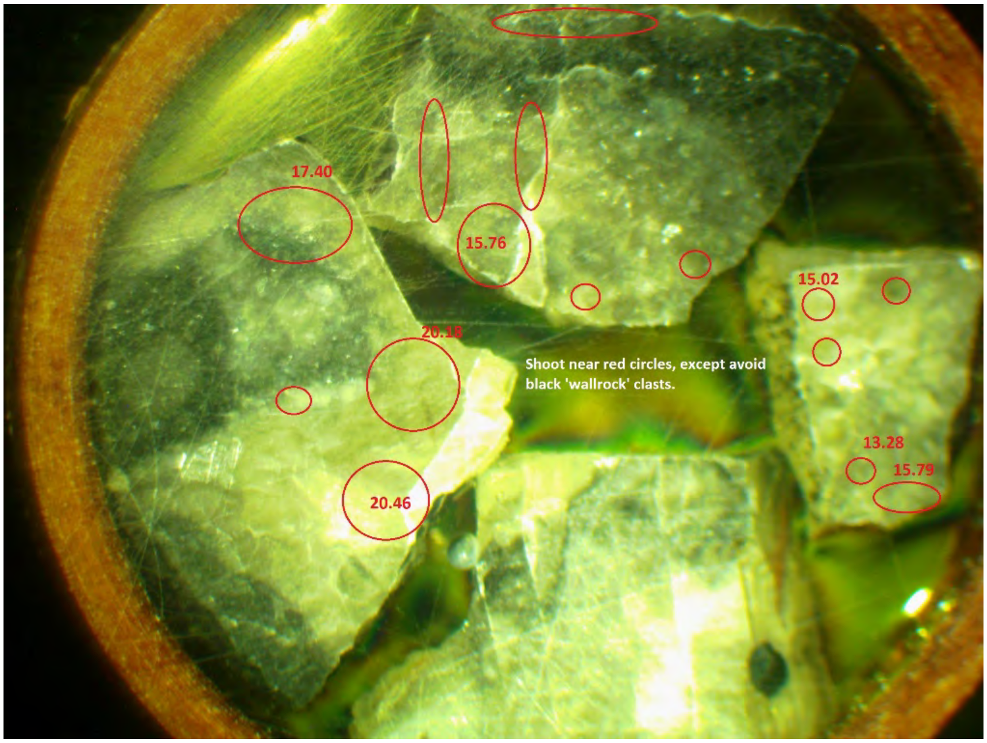

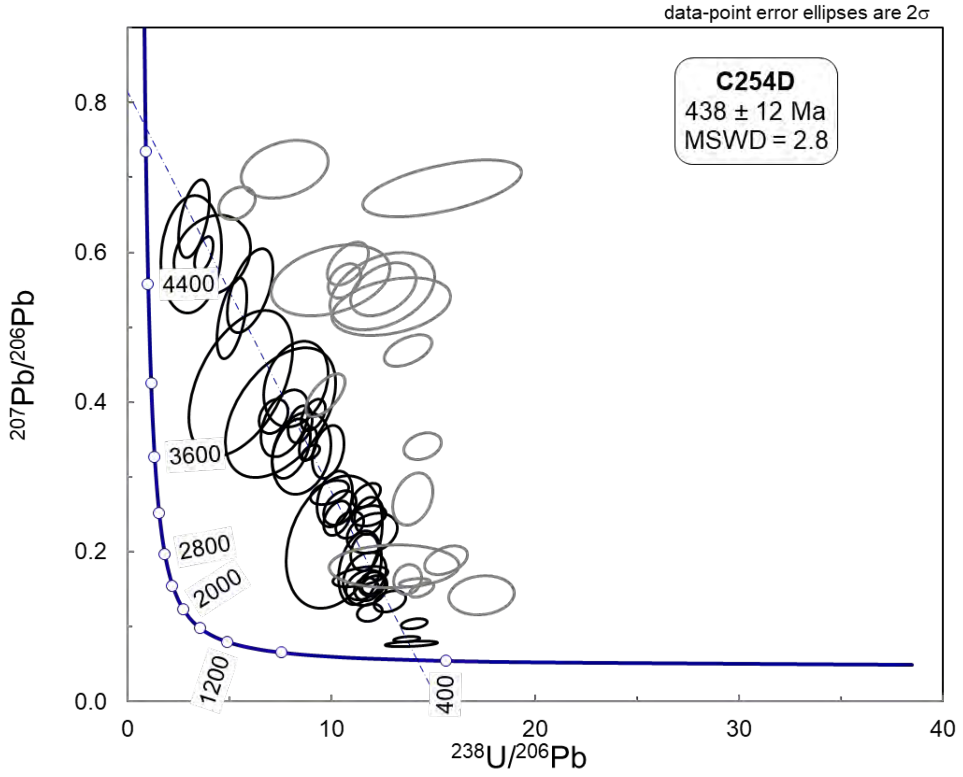

C256: Circles show areas targeted for dating, not laser shots

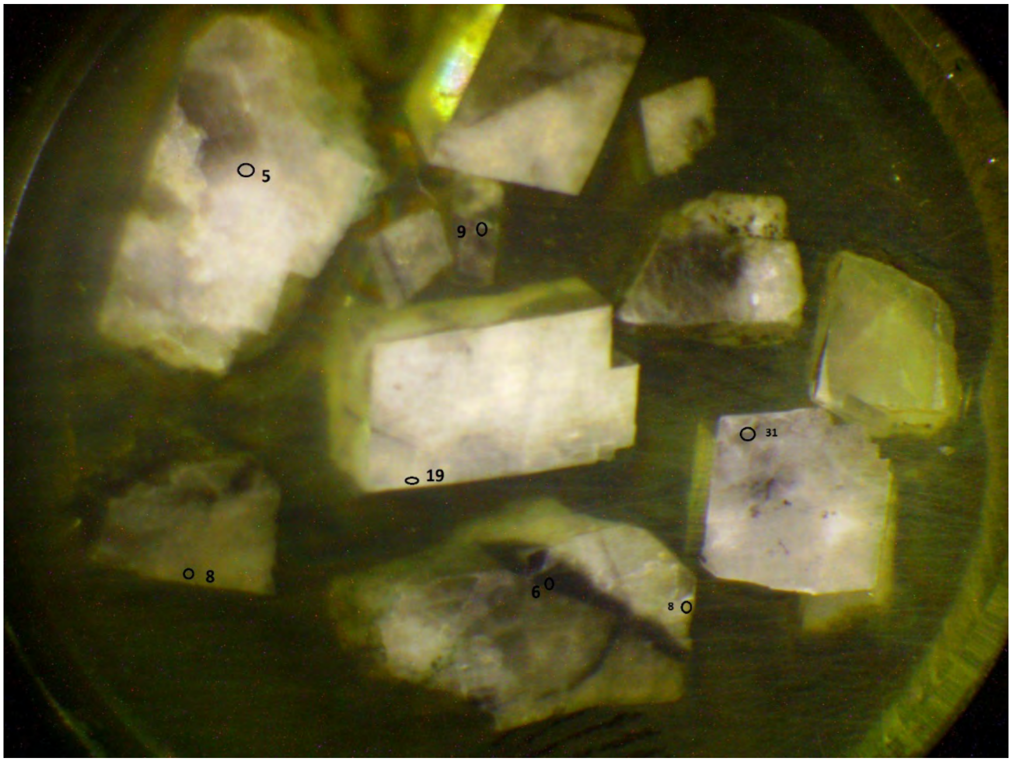

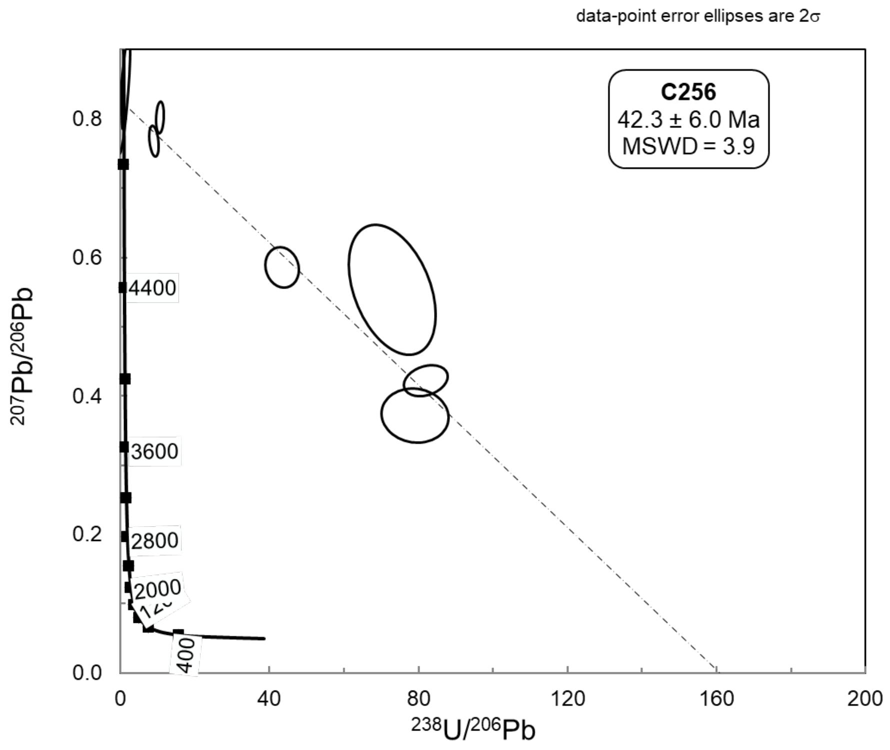

C257: Circles show areas targeted for dating, not laser shots

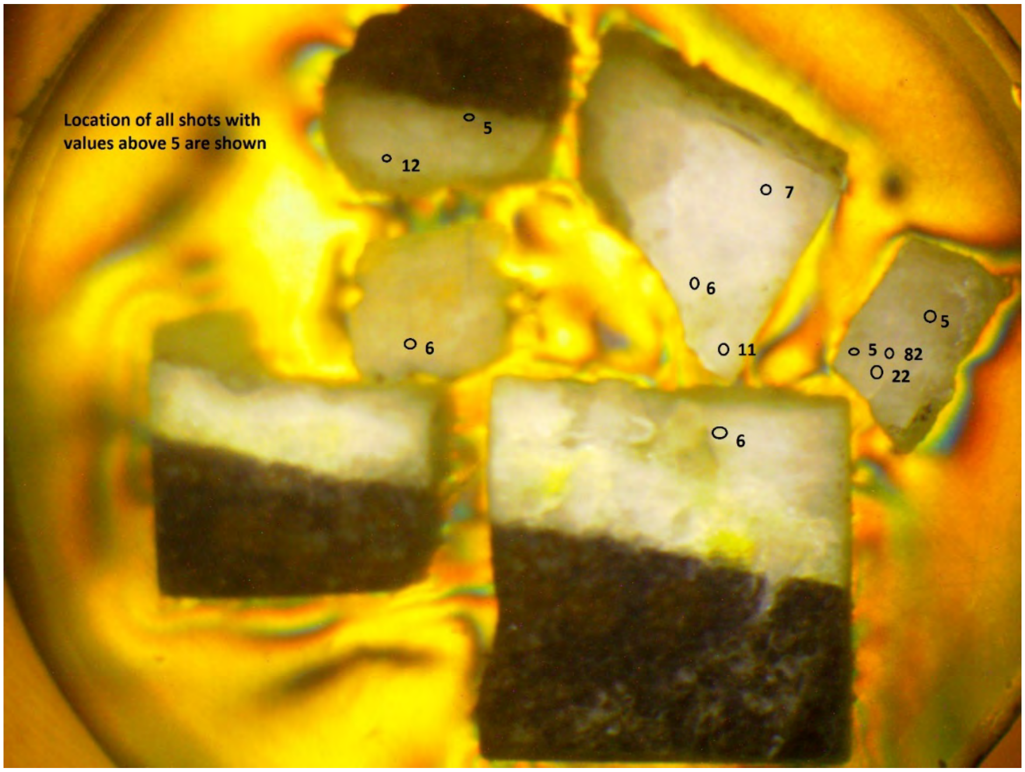

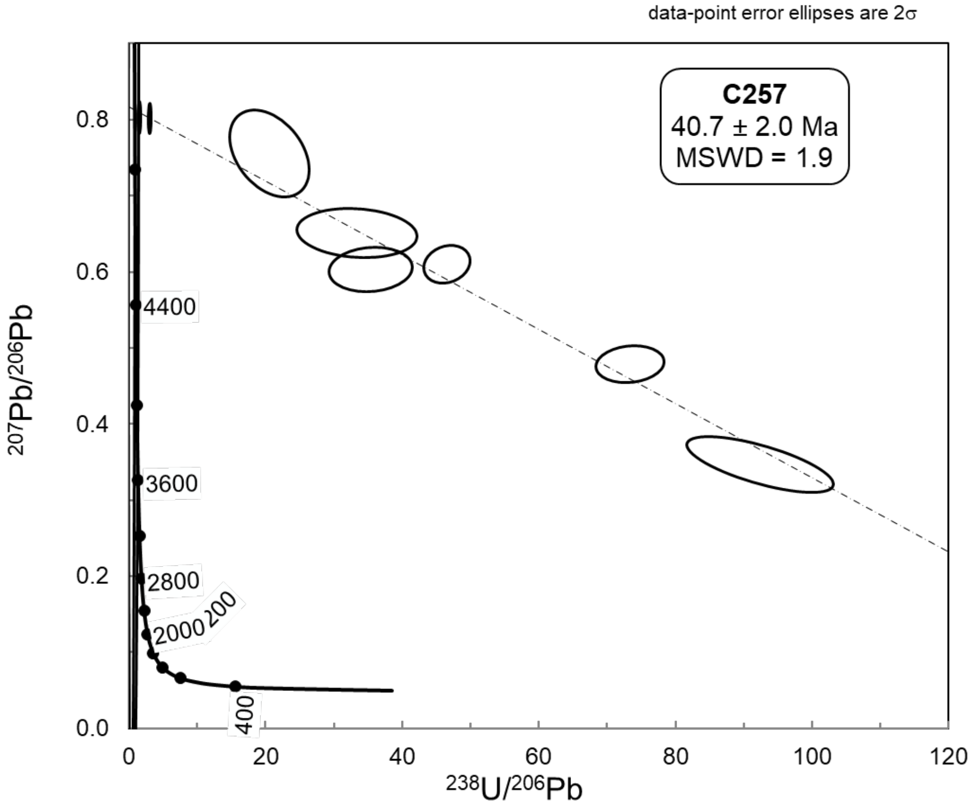

C258:

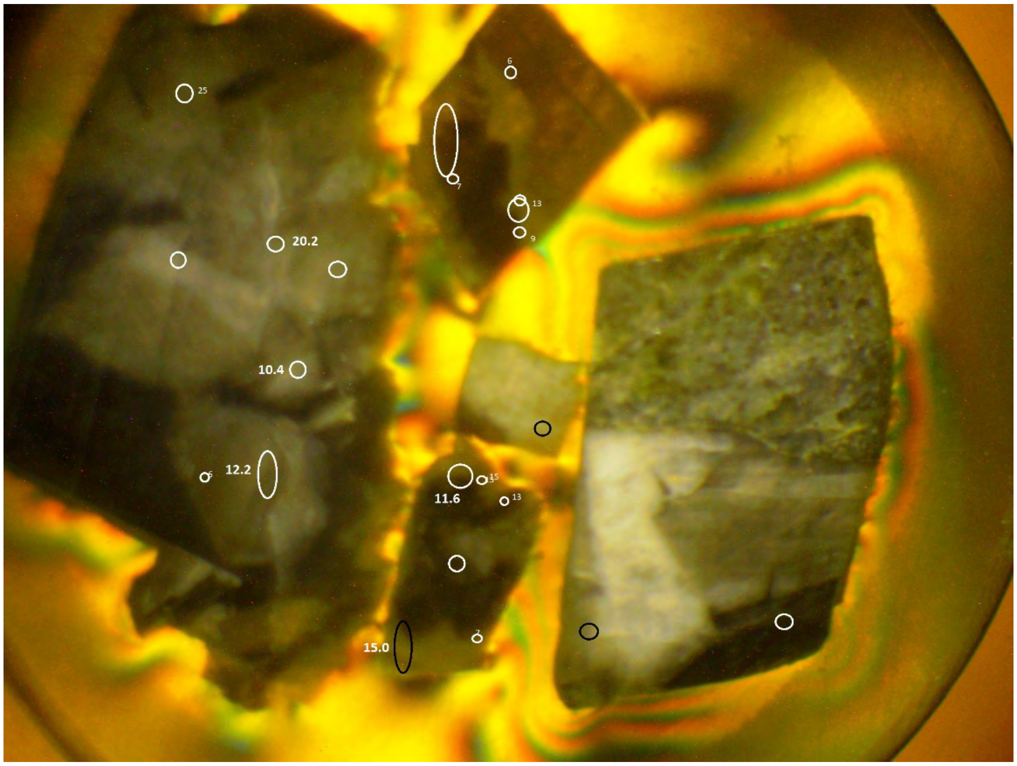

20.2
10.4
12.2
11.6
15.0

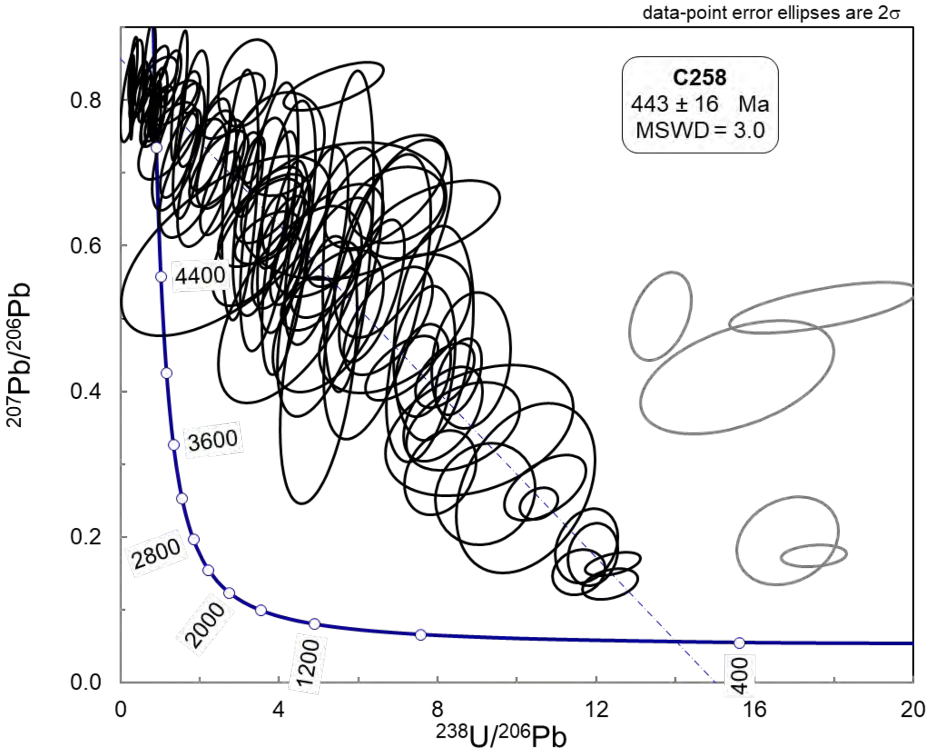

data-point error ellipses are 2σ
C258
443 ± 16 Ma
MSWD = 3.0
0.8
0.6
0.4
0.2
0.0
4400
3600
2800
2000
1200
400
0
4
8
12
16
20
207Pb/206Pb
238U/206Pb

C259:

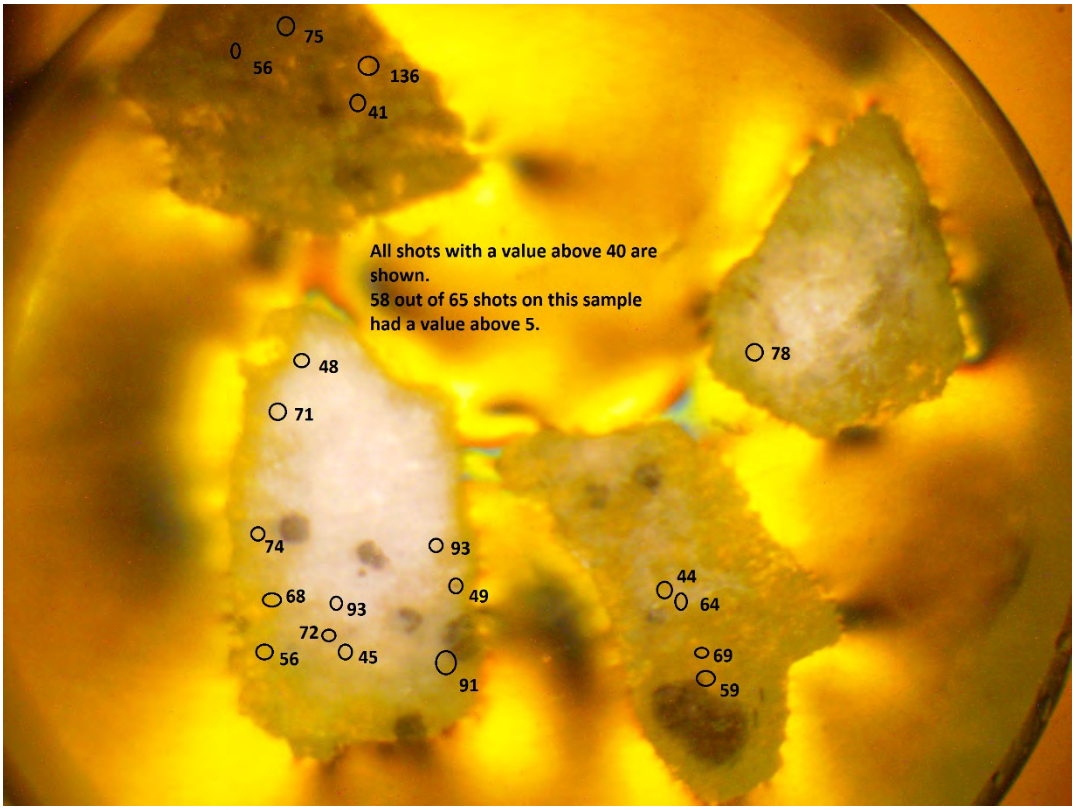
75
56
136
41
All shots with a value above 40 are shown.
58 out of 65 shots on this sample had a value above 5.
78
48
71
74
93
49
68
93
72
45
56
91
44
64
69
59

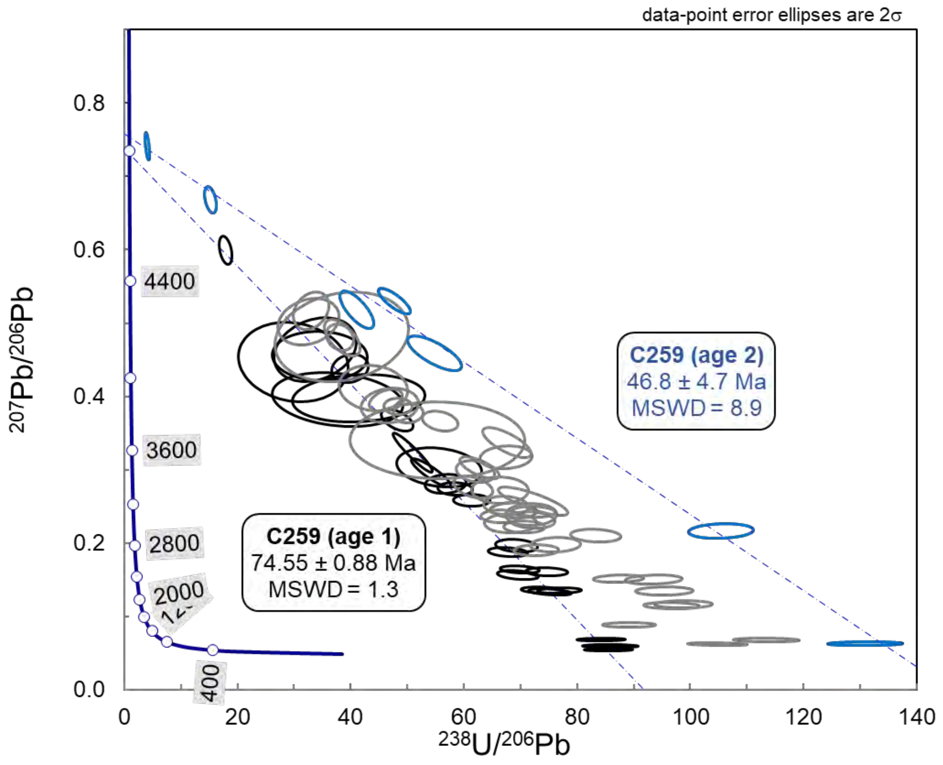
data-point error ellipses are 2σ
C259 (age 2)
46.8 ± 4.7 Ma
MSWD = 8.9
C259 (age 1)
74.55 ± 0.88 Ma
MSWD = 1.3
4400
3600
2800
2000
400
0.0
0.2
0.4
0.6
0.8
0
20
40
60
80
100
120
140
207Pb/206Pb
238U/206Pb
