# Supplementary material for: Pace of passive margin tectonism revealed by U-Pb dating of fracture-filling calcite
Source: Nat Commun. 2022 Apr 12;13:1953. doi: 10.1038/s41467-022-29680-z (PMC9005700; doi:10.1038/s41467-022-29680-z)

19: Kingsland Bay North (44.23987, -73.30760)

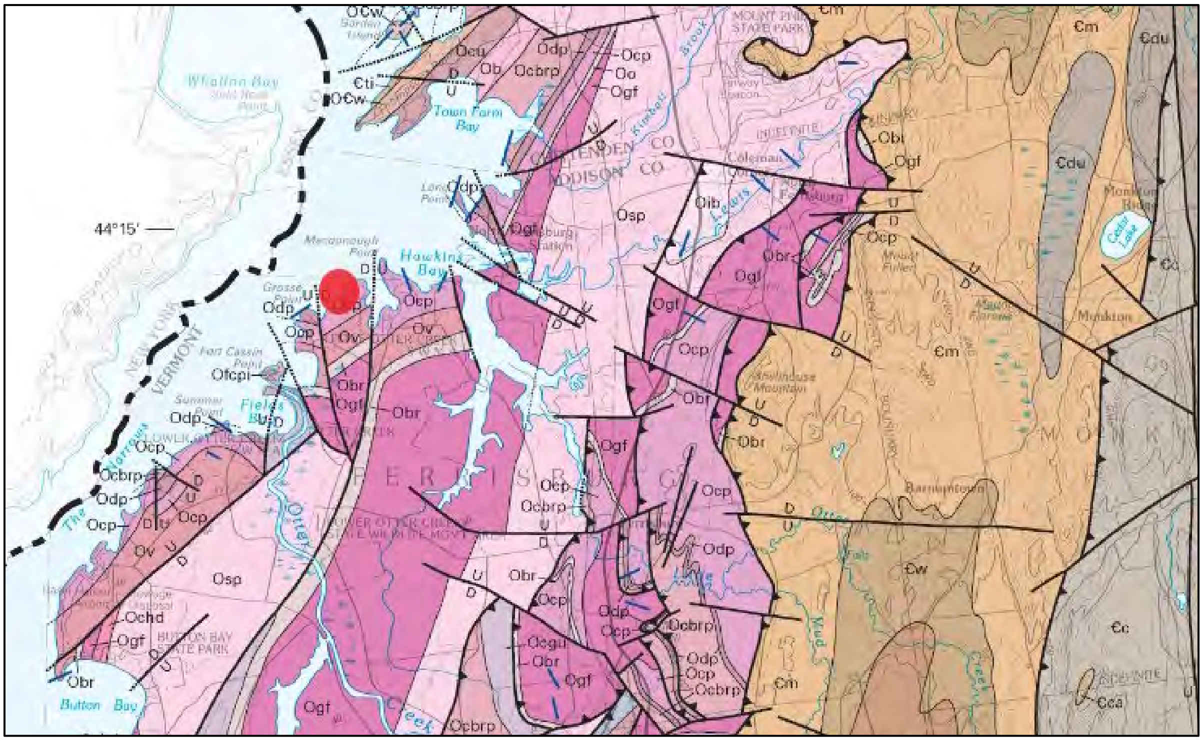

Figure 1: Sample location on state geologic map of Ratcliffe at al. (2011).

<u>Undated samples from this site</u>: none

<u>Bedrock Unit:</u> Crown Point Formation (Ocp)

<u>Ages</u>: C268A: 83.7 ±1.5; **C268B**: 94.6 ±2.1; **C268C**: 99.6±7.3; **C268D**: 79.8±1.2; **C268E**: 79.2±2.9

<u>Summary</u>: This site is the northernmost of the sites on along the west shore of the Kinsgland Bay peninsula and can only be reached by canoe during calm water when it is possible to step from the boat onto the steep shoreline. Difficult access has left this site understudied although the ages agree well with sites immediately to the south (20, 21a,b,c). The limestone is characterized by two sub-orthogonal planar fracture sets, one at roughly E-W and one at roughly N-S (Fig. 2). Although these may have formed as conjugate fractures, only the N-S fractures are mineralized suggesting that these were at least reactivated as mode 1 dilational fractures. This pattern is similar to the fracture pattern observed at sites 1 and 20. With the exception of sample 268C, which was never relocated, we interpret all structures as mode 1 veins and infer stresses assuming σ_3 is perpendicular to the vein, which yields E-W oriented σ_3 stresses for all of the veins.

The ages are of moderate quality, with all samples exhibiting some outlying laser shots. Outlying shots falling off the obvious isochron line were culled regardless of whether they fell above or below the isochron line. This is justified by the idea that some shots may be erroneously young due to secondary crystallization events whereas others may represent mixtures of vein and host rock. In particular, sample 268B contains a handful of shots defining an Ordovician-aged isochron, which we interpret as the age of the host limestone.

Field Observations:

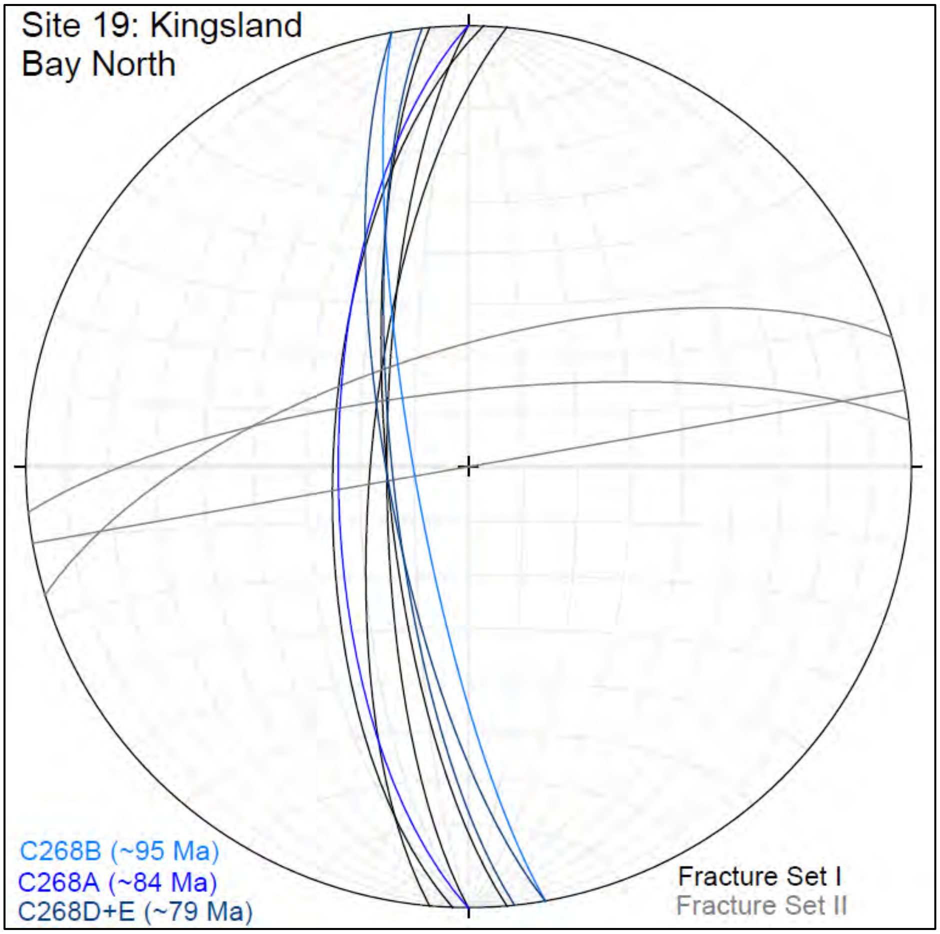

Figure 2: Stereonet showing orientation of structures at Kingsland Bay North

C268A:

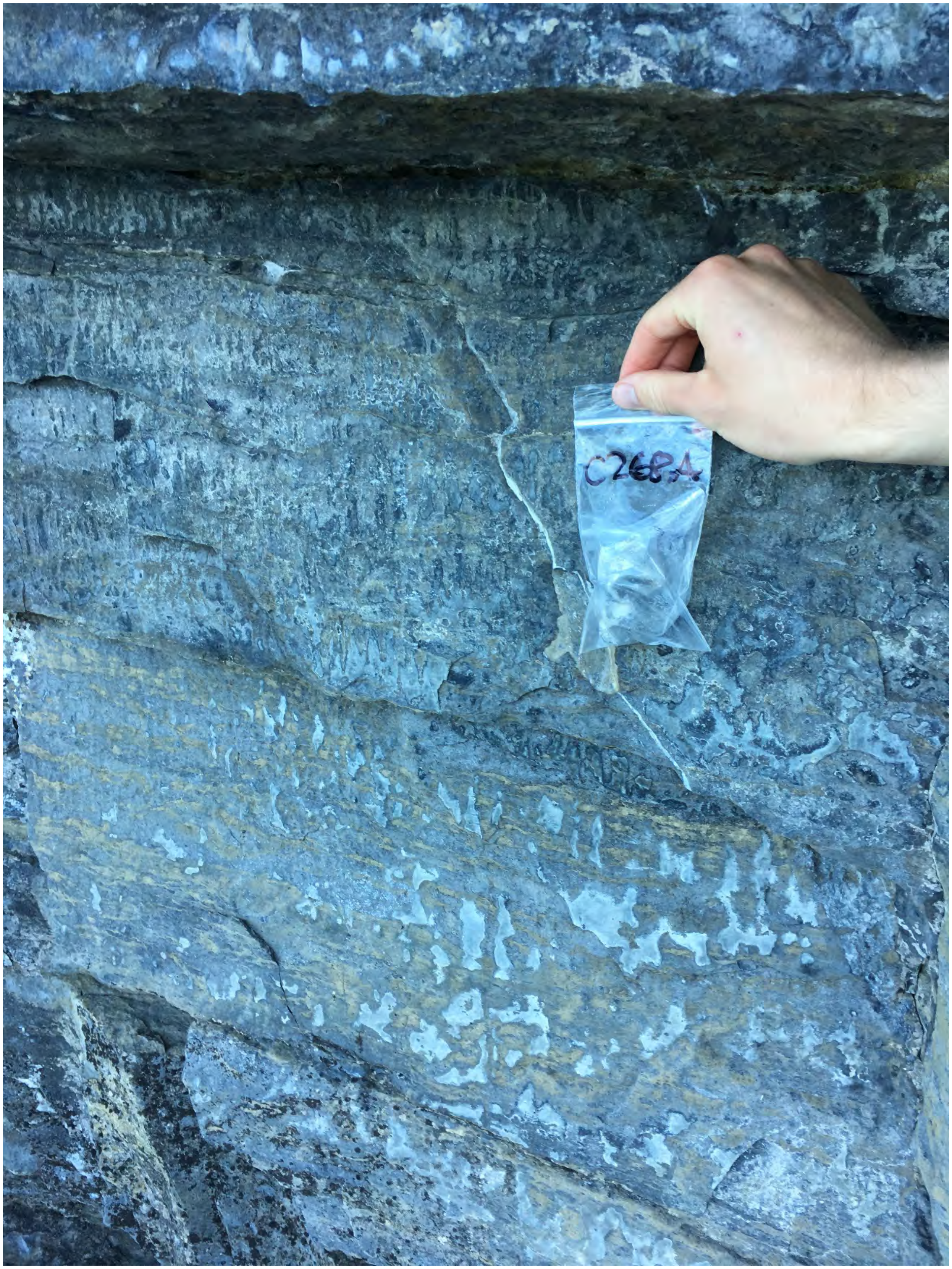

C268B:

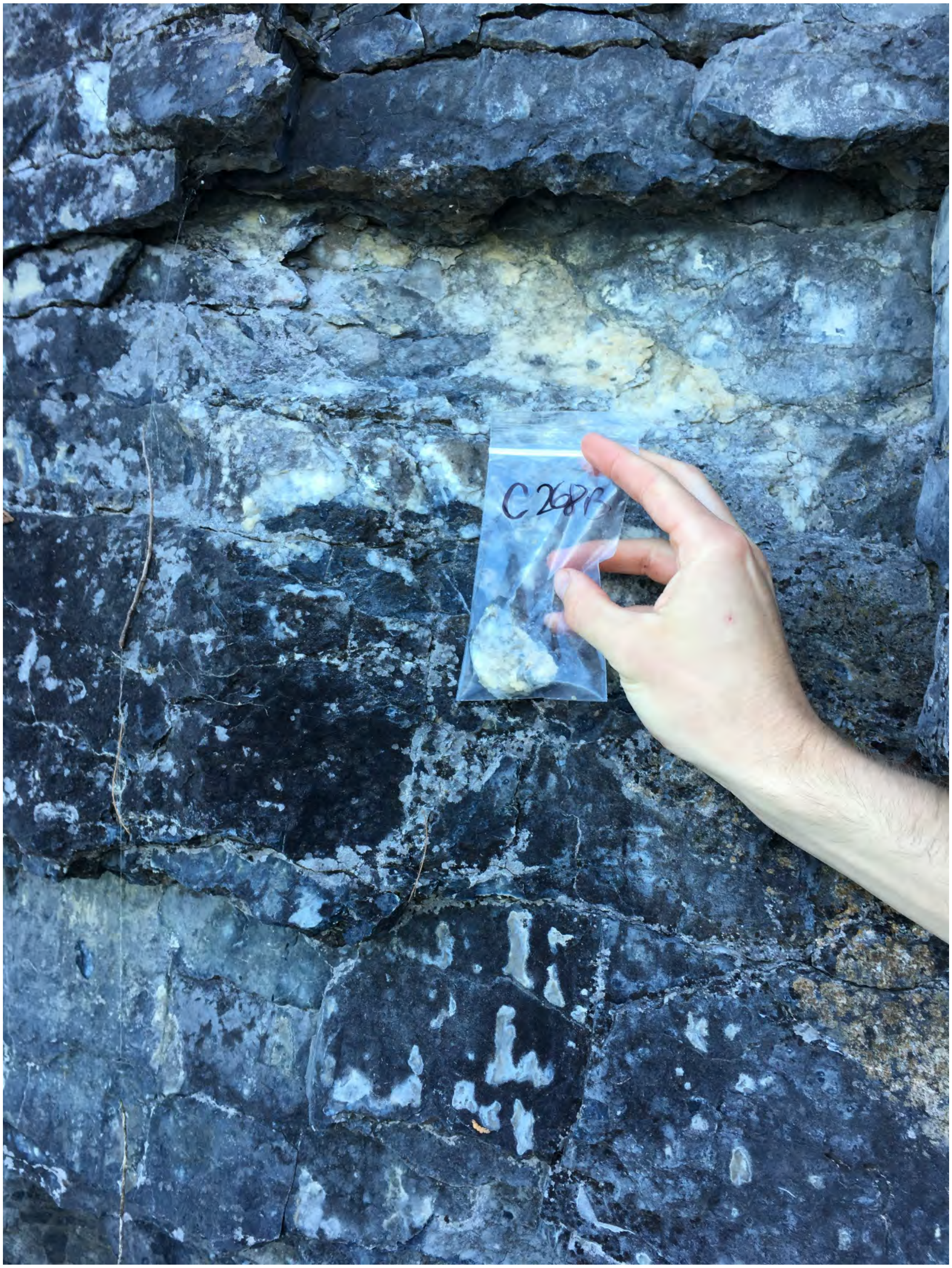

C268C:

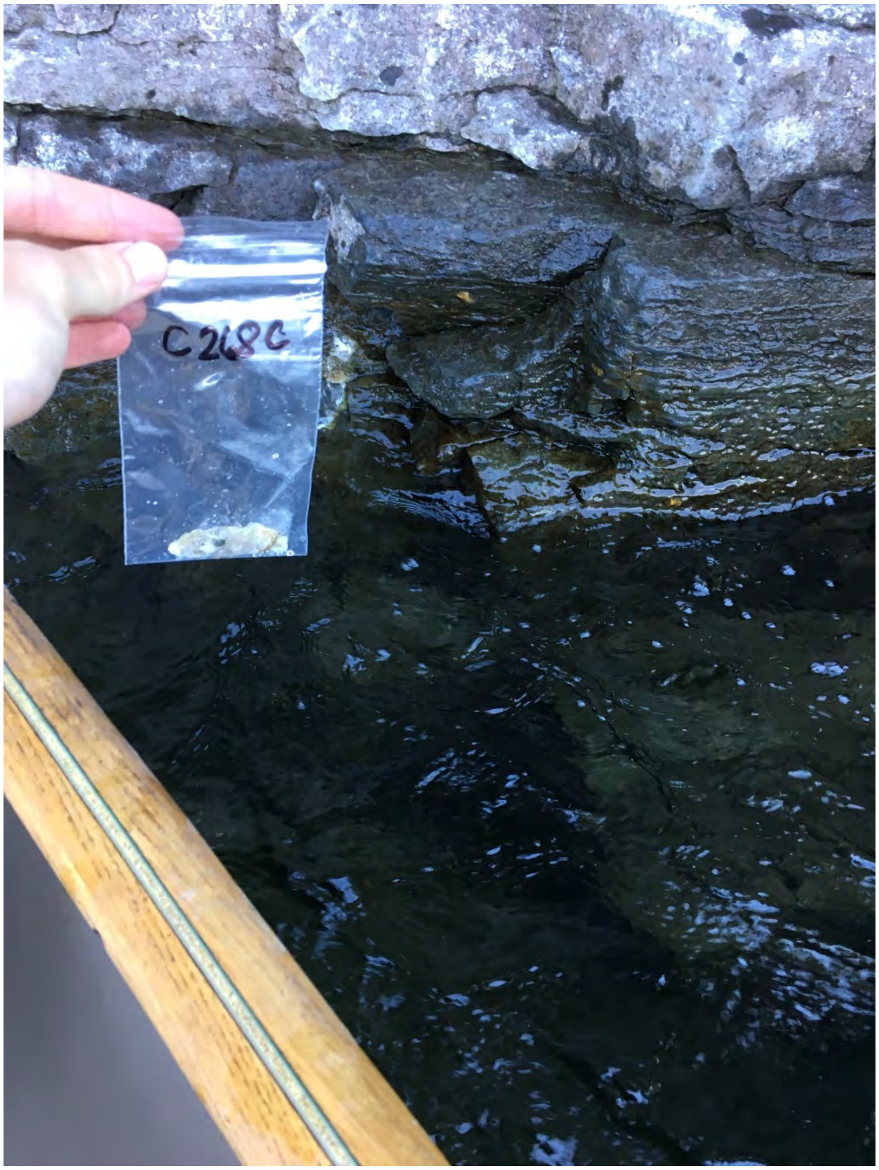

C268D

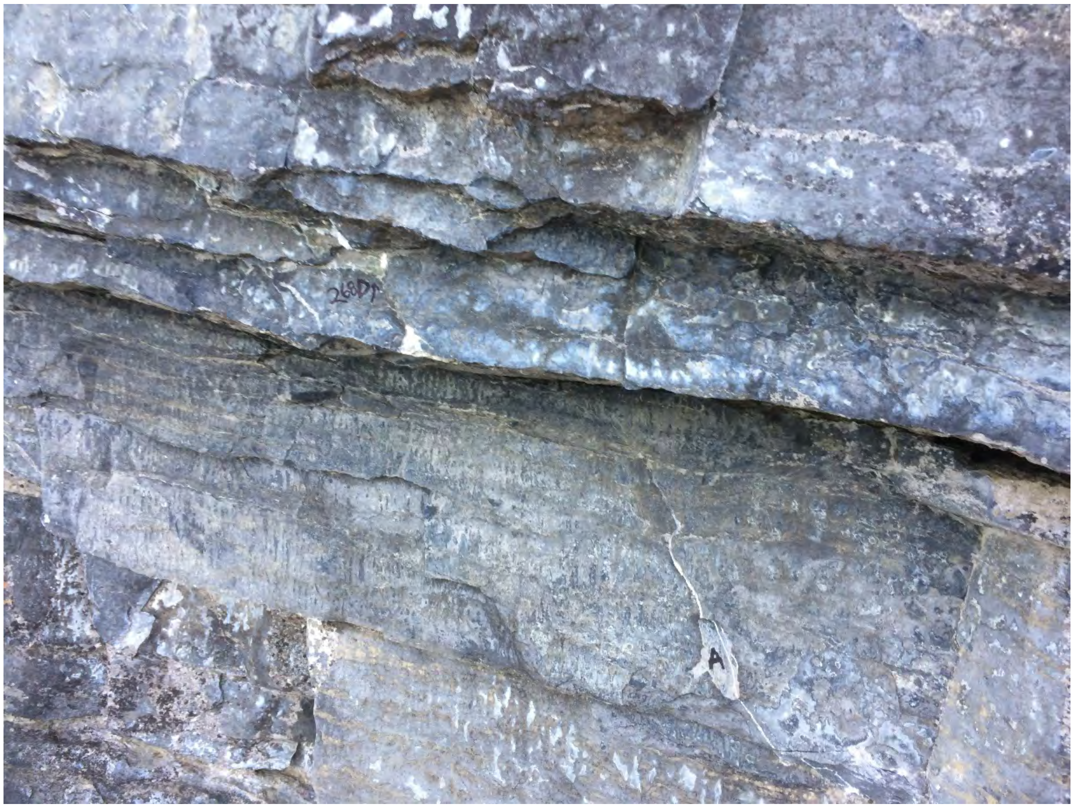

C268E

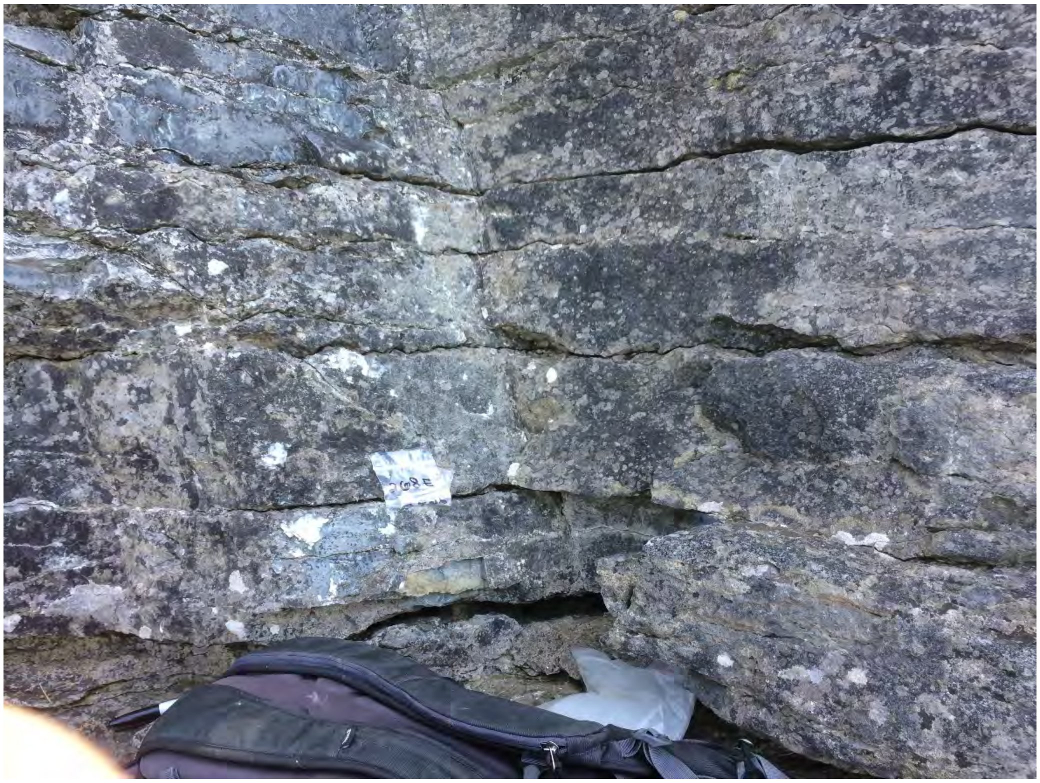

Lab Description:

C268A: Circles denote areas target for U-Pb analysis

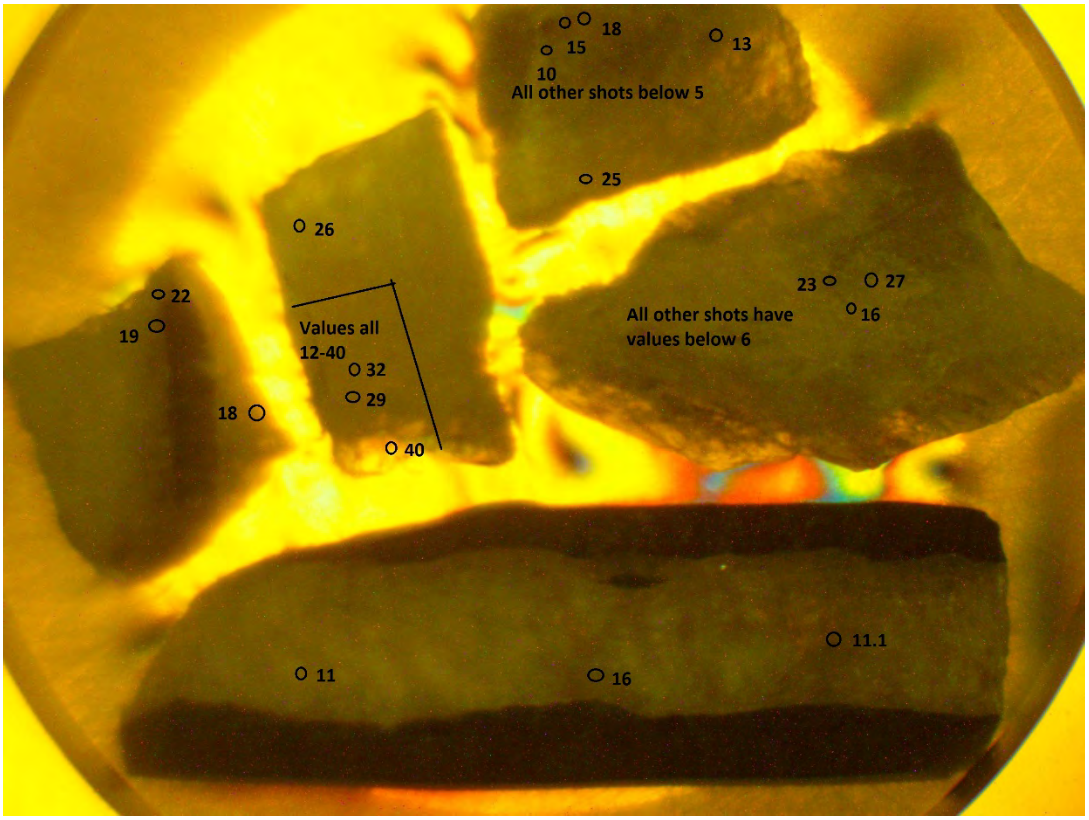

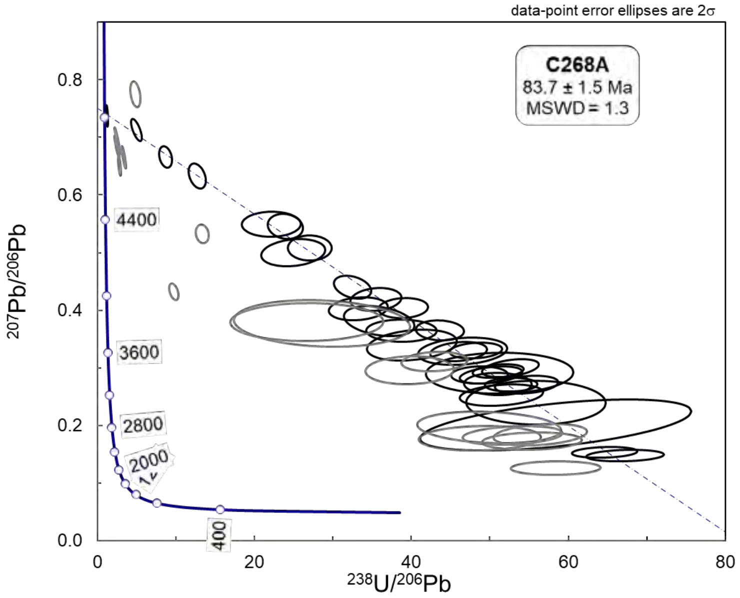

C268B: Circles denote areas target for U-Pb analysis

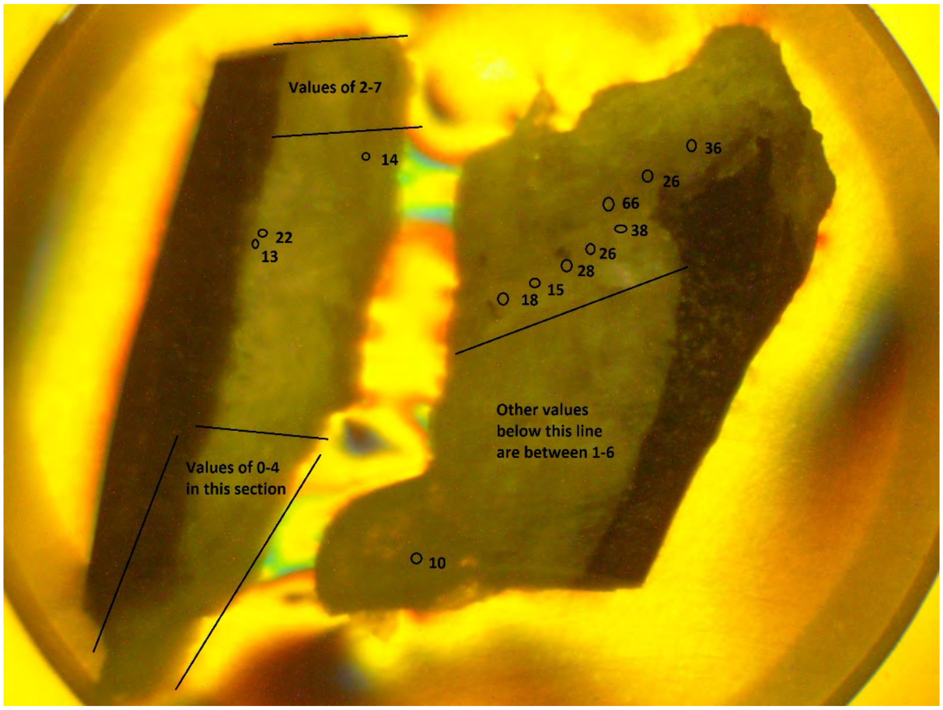

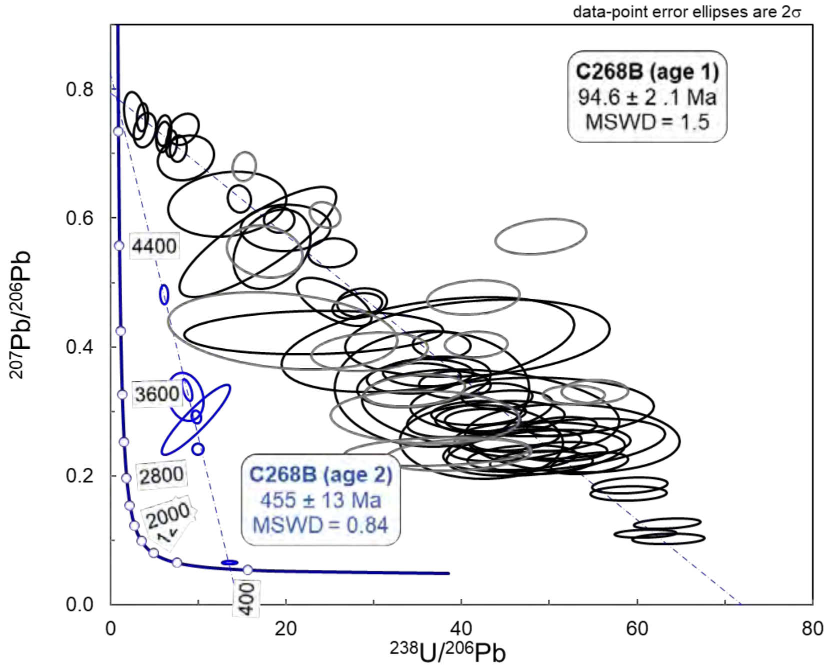

C268C: Circles denote areas target for U-Pb analysis

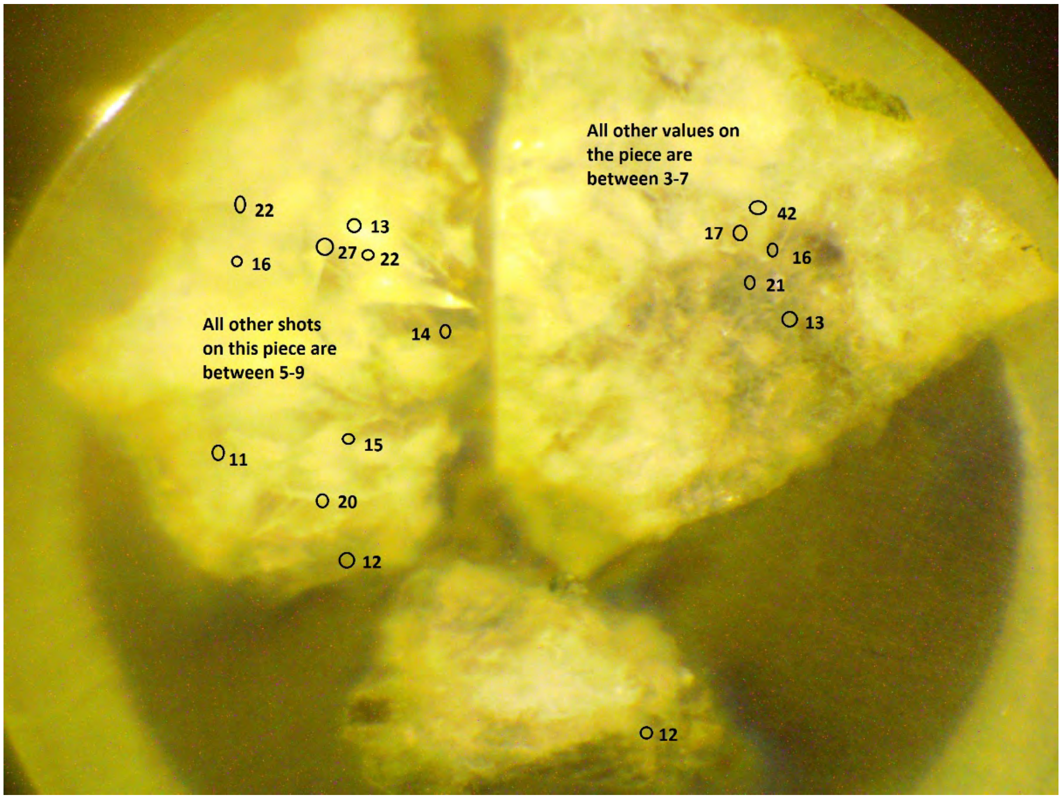

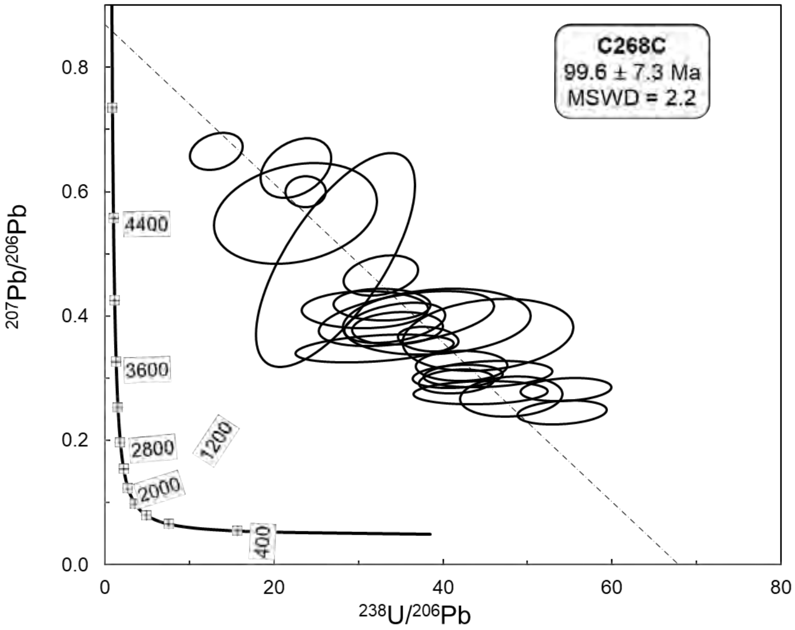

C268D: Circles denote areas target for U-Pb analysis

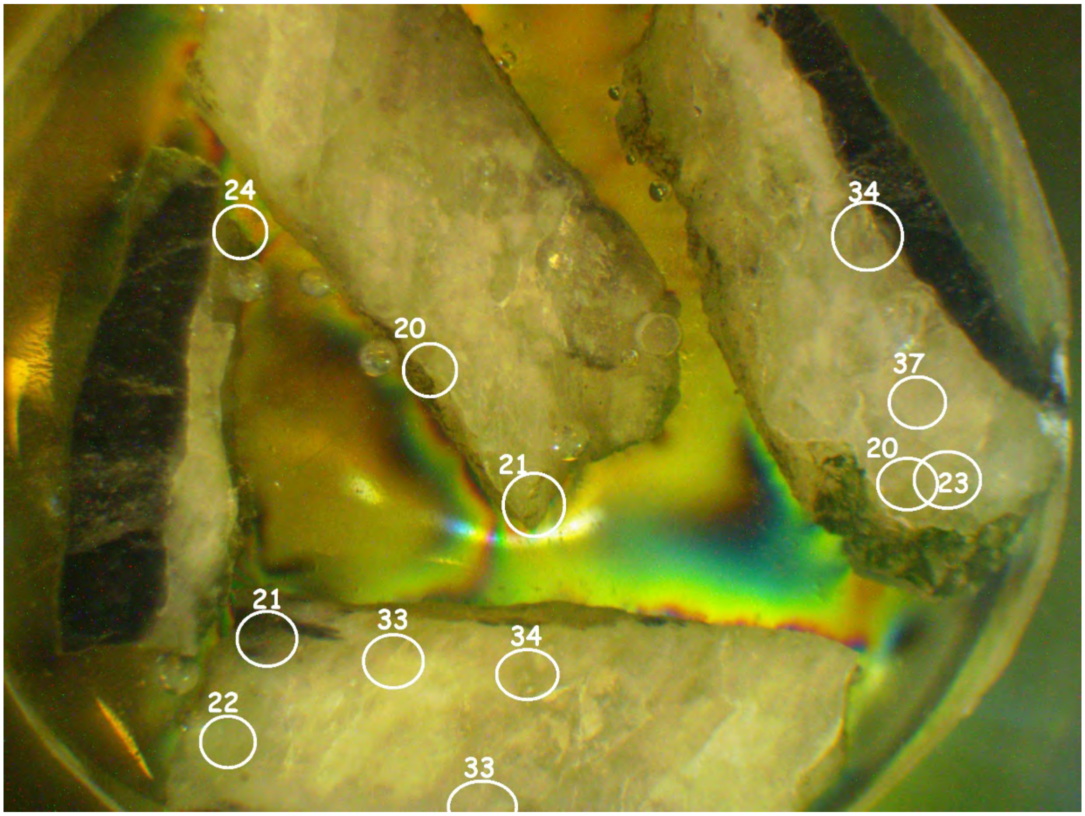

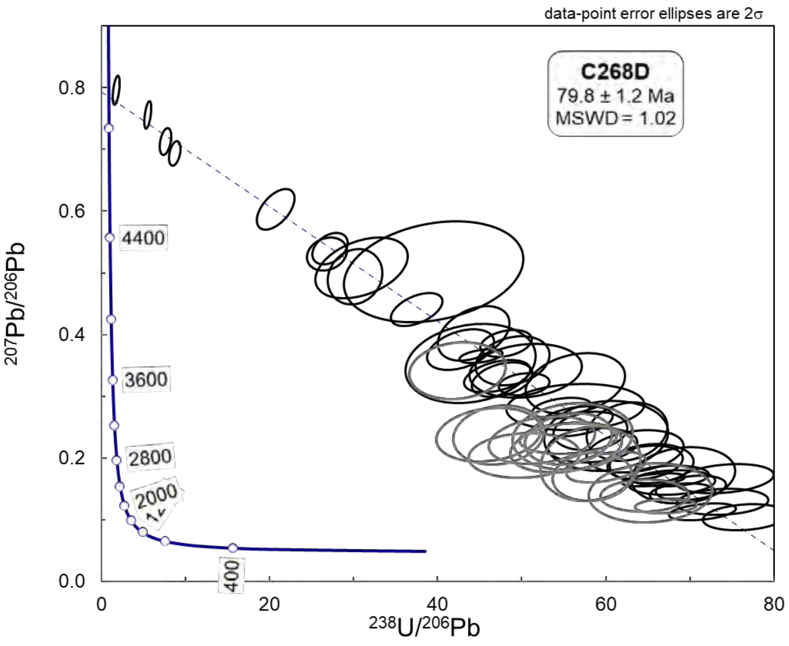

C268E: Circles denote areas target for U-Pb analysis

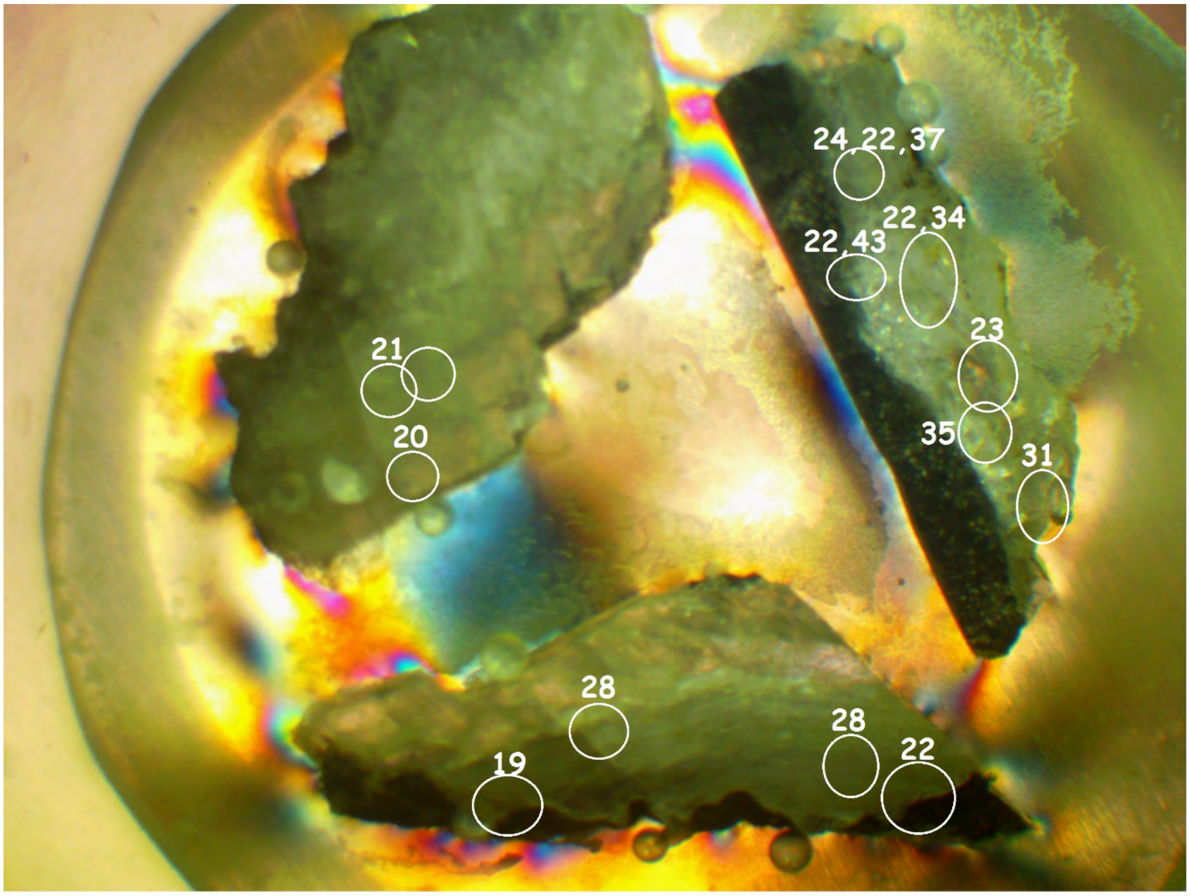

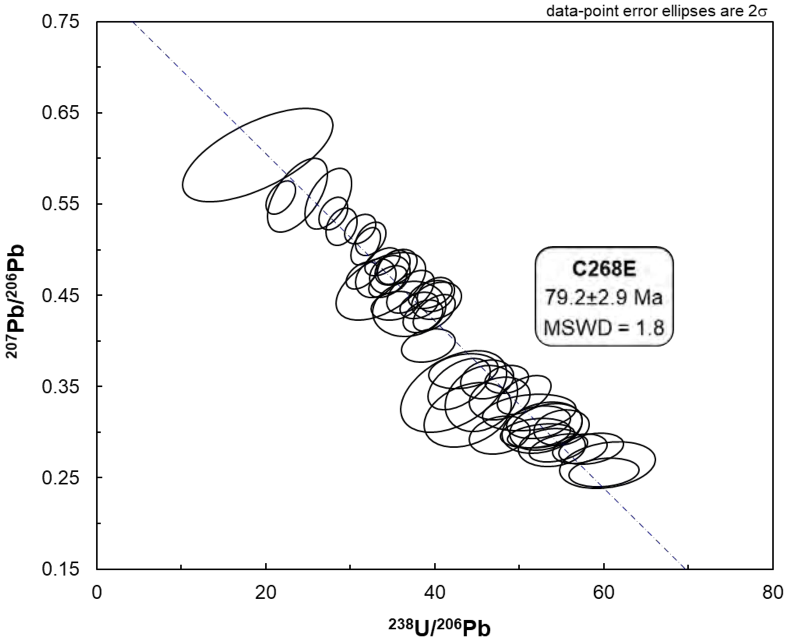

Supplement: Supplementary file 6 — Source Data [file 41467_2022_29680_MOESM6_ESM.zip › 1.zip!/appendix2_site_summaries_1to20/19_kingsland_bay_north_final.pdf]
