# Supplementary material for: Pace of passive margin tectonism revealed by U-Pb dating of fracture-filling calcite
Source: Nat Commun. 2022 Apr 12;13:1953. doi: 10.1038/s41467-022-29680-z (PMC9005700; doi:10.1038/s41467-022-29680-z)

2: West Haven, VT   (43.653241, -73.325777)

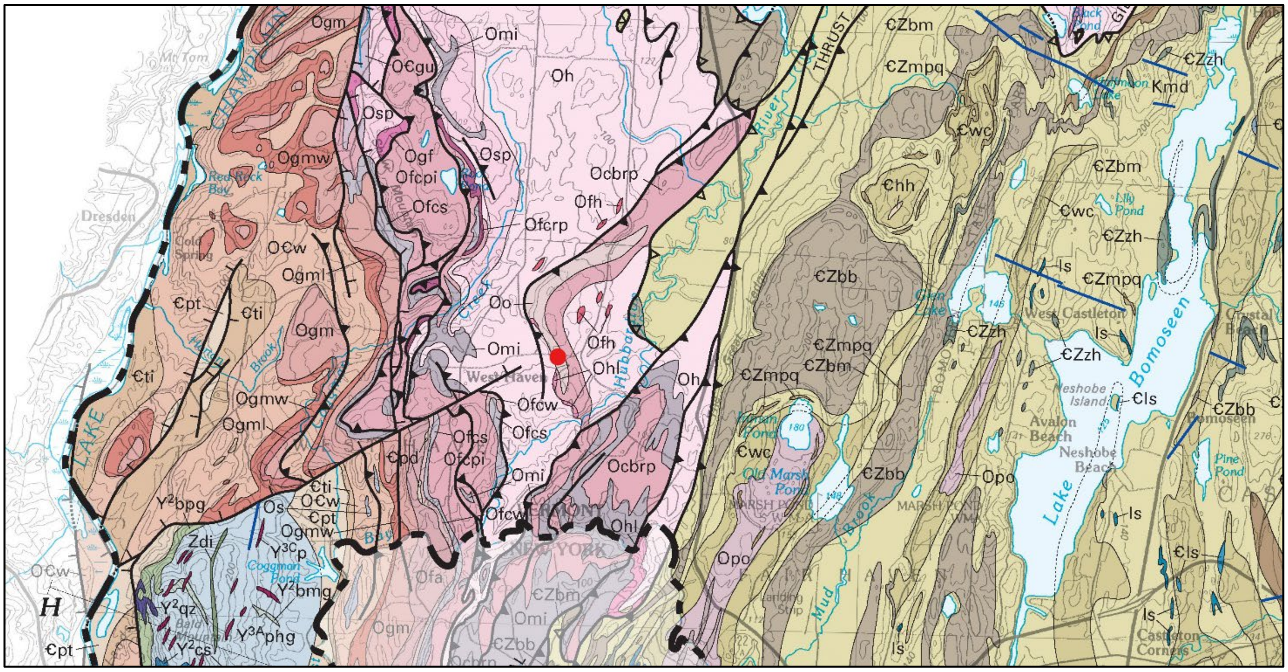

Figure 1: Sample located on VT state geologic map of Ratcliffe et al. (2011).

<u>Failed samples from same site:</u> C017B,C,D,E

<u>Bedrock unit</u>: Orwell Limestone

<u>Ages</u>: C017/17a: 82.2 ± 4.2 Ma (combined two replicate samples collected from same vein)

<u>Summary</u>: This site is located in West Haven, VT, on a low roadcut on the north side of Main road roughly 1000 ft east of the intersection with Best Rd. The outcrop is set back a few feet from the road, partially obscured by bushes. Sample C017 and 17a were collected from the same vein on different dates and the analytical results were combined. The vein is 1-2 mm thick, sparry calcite plating a very planar, continuous (>2 m) mode 1 fracture. The planar nature and lack of offset or slickenfibres leads to an interpretation of a mode 1 vein formed under a N-S oriented $\sigma 3$ stress at ~82 Ma. Interestingly this inferred $\sigma 3$ stress differs from the E-Q or NE-SW $\sigma 3$ stress inferred for this time period from sites further north in the Champlain Valley. Poor exposure at this site makes the tectonic interpretation tentative.

Field Observations:

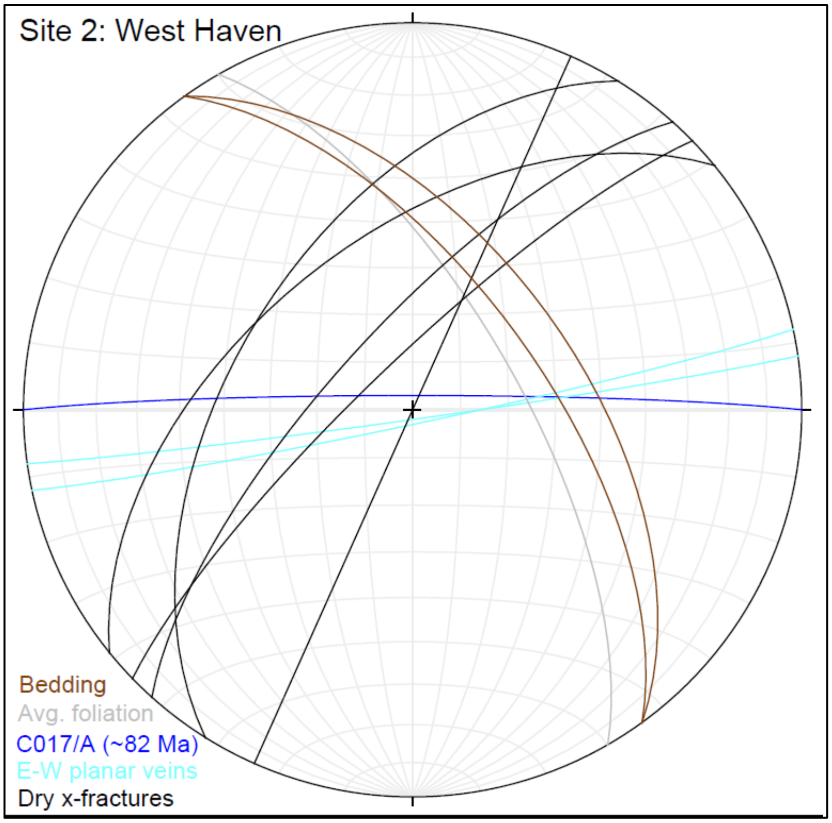

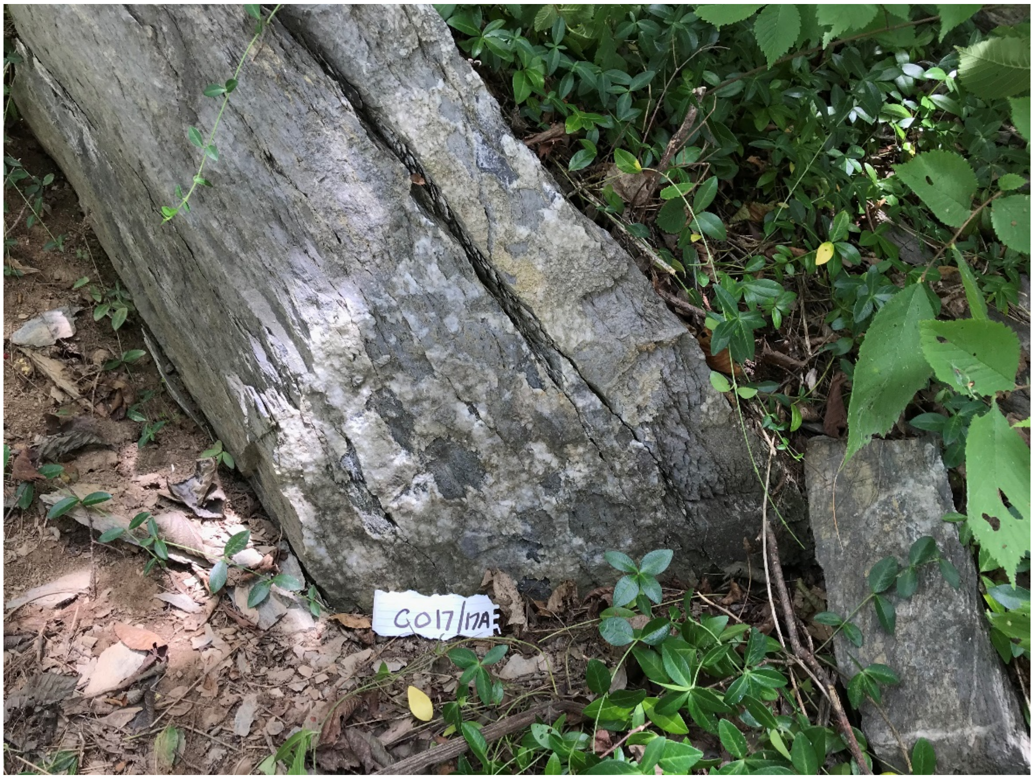
CO17/17A

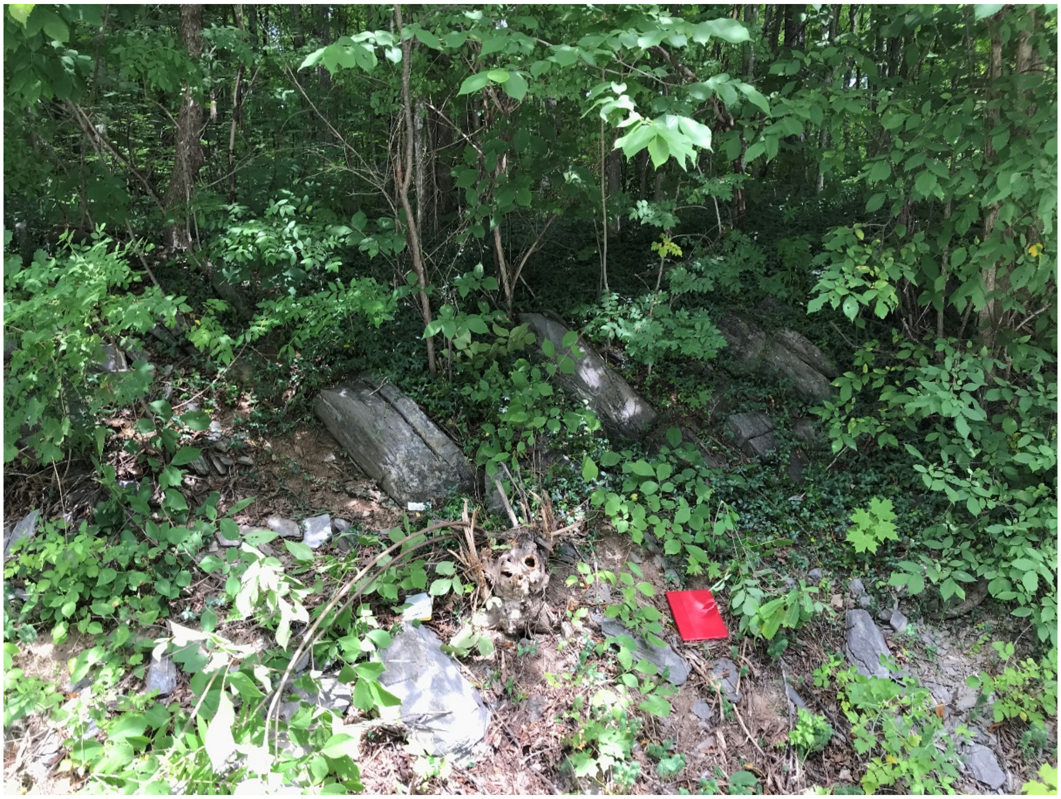

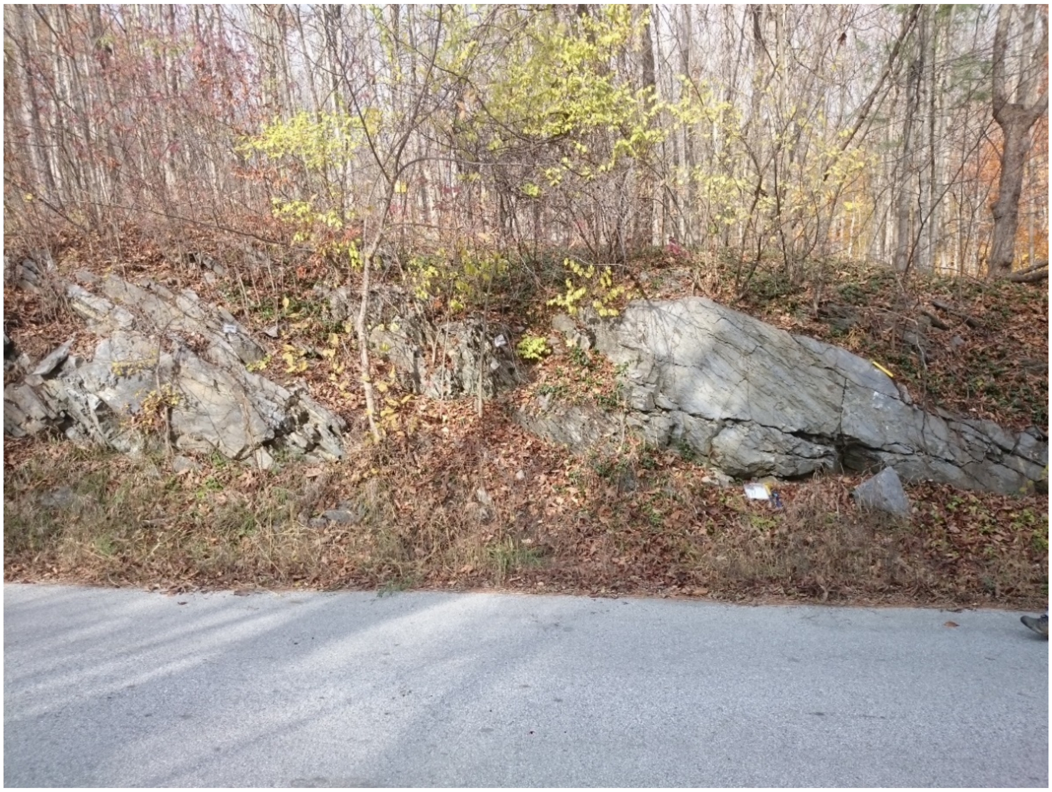

Sample C017B,C,D taken from minor lstrike-slip cross-faults in these outcrops and failed to date. Sample C017/A is located about 10 m to left of this photo.

Lab observations:

C017: No sample photo available. Data combined with replicate sample C017A below

C017A: Circles denote areas target for dating, not actual laser shots

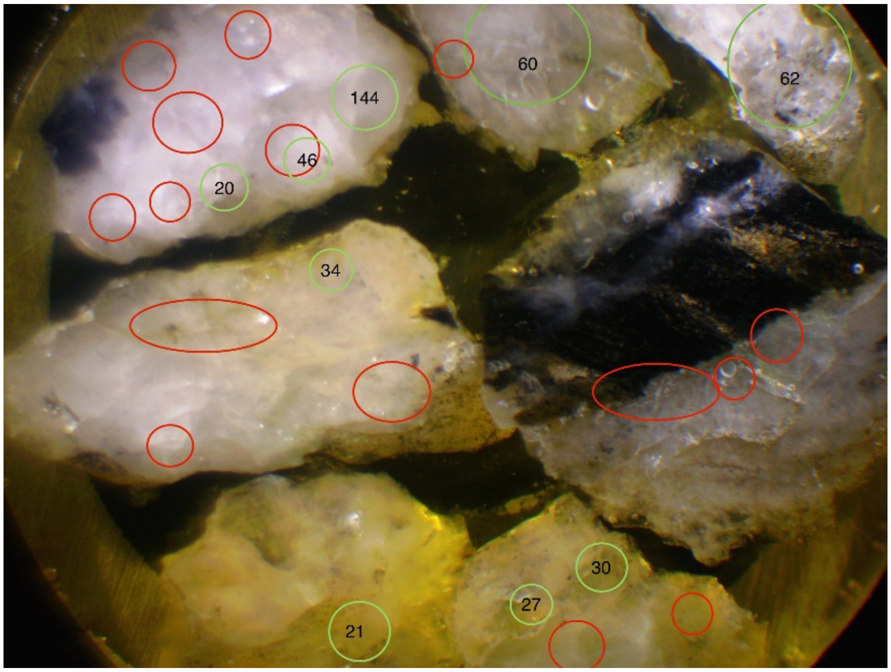

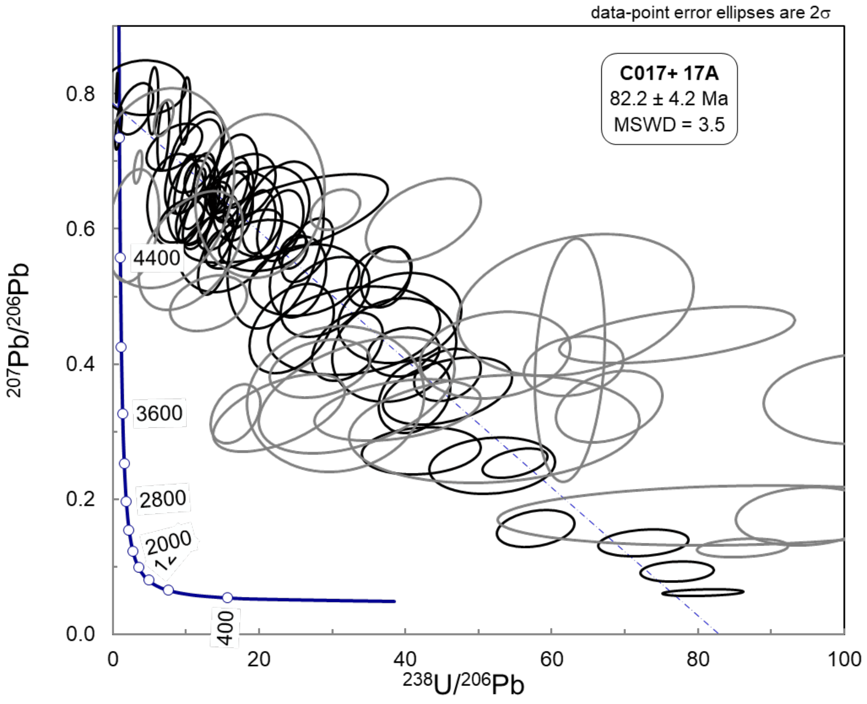

Supplement: Supplementary file 6 — Source Data [file 41467_2022_29680_MOESM6_ESM.zip › 1.zip!/appendix2_site_summaries_1to20/2_west_haven_final.pdf]
