# Supplementary material for: Pace of passive margin tectonism revealed by U-Pb dating of fracture-filling calcite
Source: Nat Commun. 2022 Apr 12;13:1953. doi: 10.1038/s41467-022-29680-z (PMC9005700; doi:10.1038/s41467-022-29680-z)
Supplement: Supplementary file 6 — Source Data [file 41467_2022_29680_MOESM6_ESM.zip › 1.zip!/appendix2_site_summaries_1to20/20_kingsland_bay_central_final.pdf]

20: Kingsland Bay Central (44.23962, -73.3076)

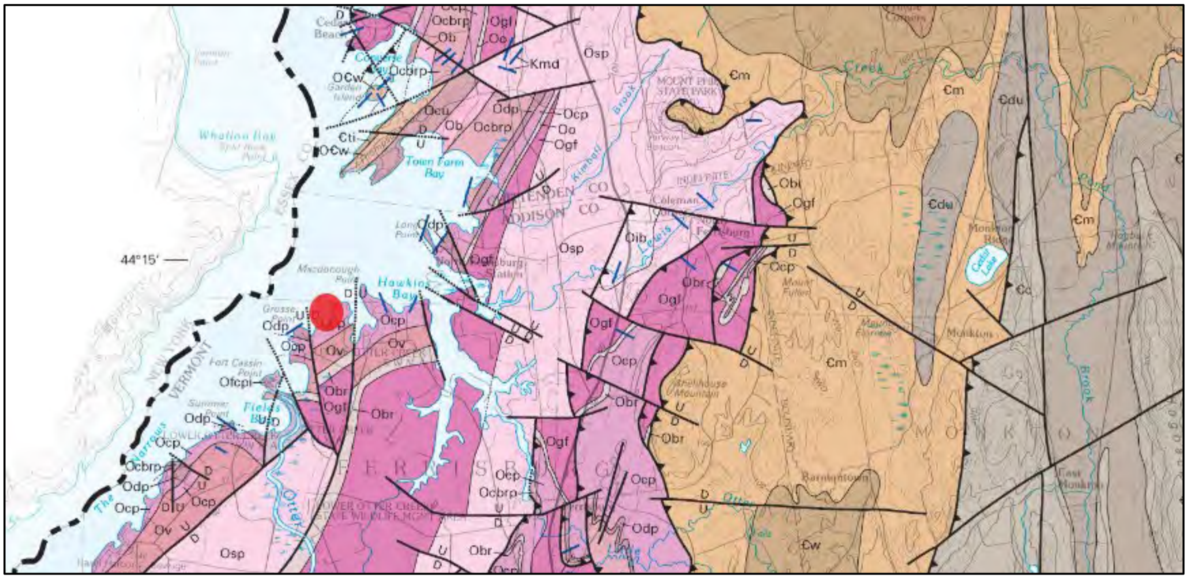

Figure 1: Sample location on state geologic map of Ratcliffe at al. (2011).

<u>Failed samples from same site:</u> C270F

<u>Bedrock Unit:</u> Crown Point Formation (Ocp)

<u>Ages</u>: C270A: 94.9 ± 3.9; **C270B**: 87±12*; **C270C**: 94.2 ± 3.7; **C270D**: 105 ± 9^; **C270E**: 92.5±1.7 & 78.4±2.7 Ma **C270G**: 77.3 ±2.5; **only 9 shots taken* *^only 20 shots taken, need more data*

Summary: Site on western side of Kingsland Bay Peninsula located about 100 m north of the major fault zone observed at site 21a. The location of this site along the continuation of an extensional fault, which surely influenced the local stress regime. This site is best accessed by parking at the Ferrisburgh Town Beach, hiking across the peninsula and scrambling down or around a 6’ cliff.

This site is dominated by two groups of structures. First are three sets of ultra-planar brittle fractures (1, 2, and 3) shown in in figure 2 below. The brittle fractures lack slickenfibres or slip tiling and show evidence for only very small offsets, if any. Although there is potential for some of the fractures to be conjugate sets we were unable to demonstrate this using field or chronological relationships and thus interpret each one as a mode-1 dilational structure in which σ_3 is perpendicular to the plane. Interestingly, only the more N-S oriented fracture sets (1 and 3) contain calcite mineralization, whereas the more E-W oriented fracture set (2) is dry. This is similar to observations from sites 19 and 1. Samples 270B,D,E, and G are collected from these fracture sets. The second group is an anastomosing and often discontinuous network of non-planar veins associated with fluids penetrating through complex pathways within the rock. Samples 270A and 270C were collected from these veins.

In the context of these structures, we infer the following sequence of events. The first event is a mode 1 vein (C270D) intruded at ~ 105 Ma, striking ~325° interpreted to record a NW-SE σ_3 stress. This vein is slightly offset by the younger sets, supporting its older age. The second event is associated with hydrothermal fluid flow event at ~95 Ma in which veins in the second group (C270A and C) were

emplaced by a mechanism that did not require planar fracturing (see photos of C270A and C below). This undulatory vein structure is interpreted to arise from migratory fluids penetrating chaotic pathways through the rock under considerable temperature and/or pressure. We are thus unable to infer stress orientations for samples C270A and C. Third, creation of a roughly N-S set of fractures at ~87 +/- 12 Ma including C270B and fracture set 1, interpreted to record a roughly E-W σ_3 stress. Finally, another extensional event around 78 Ma with a NE-SW σ_3 stress recorded by samples C270E and G.

The quality of U-Pb ages is moderate for these samples, with most of them including outlying laser shots that were not included in the age estimate. In particular, the isochron age for sample C270G was constructed by taking the laser shots defining the youngest isochron, using the rationale that younger shots may represent mixtures of vein and older bedrock material.

<u>Field Observations</u>:

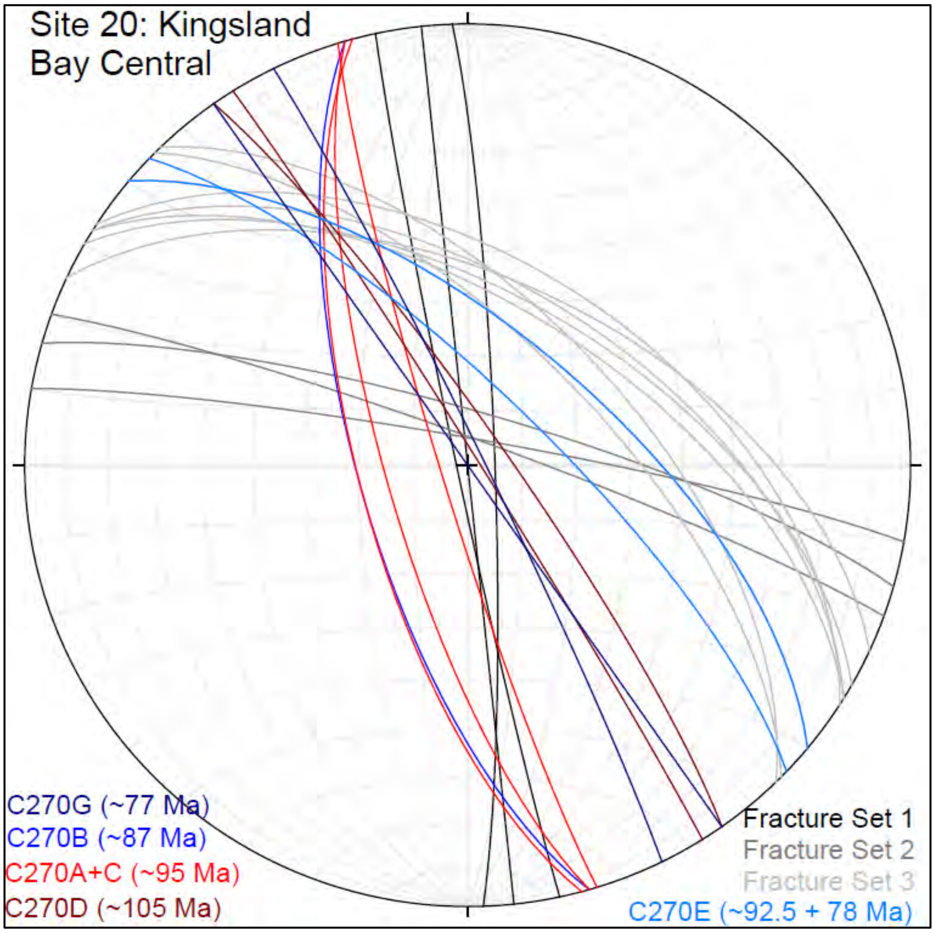

Figure 2: Stereonet showing measured structures at the Kingsland Bay Central site.

Photos of C270A:

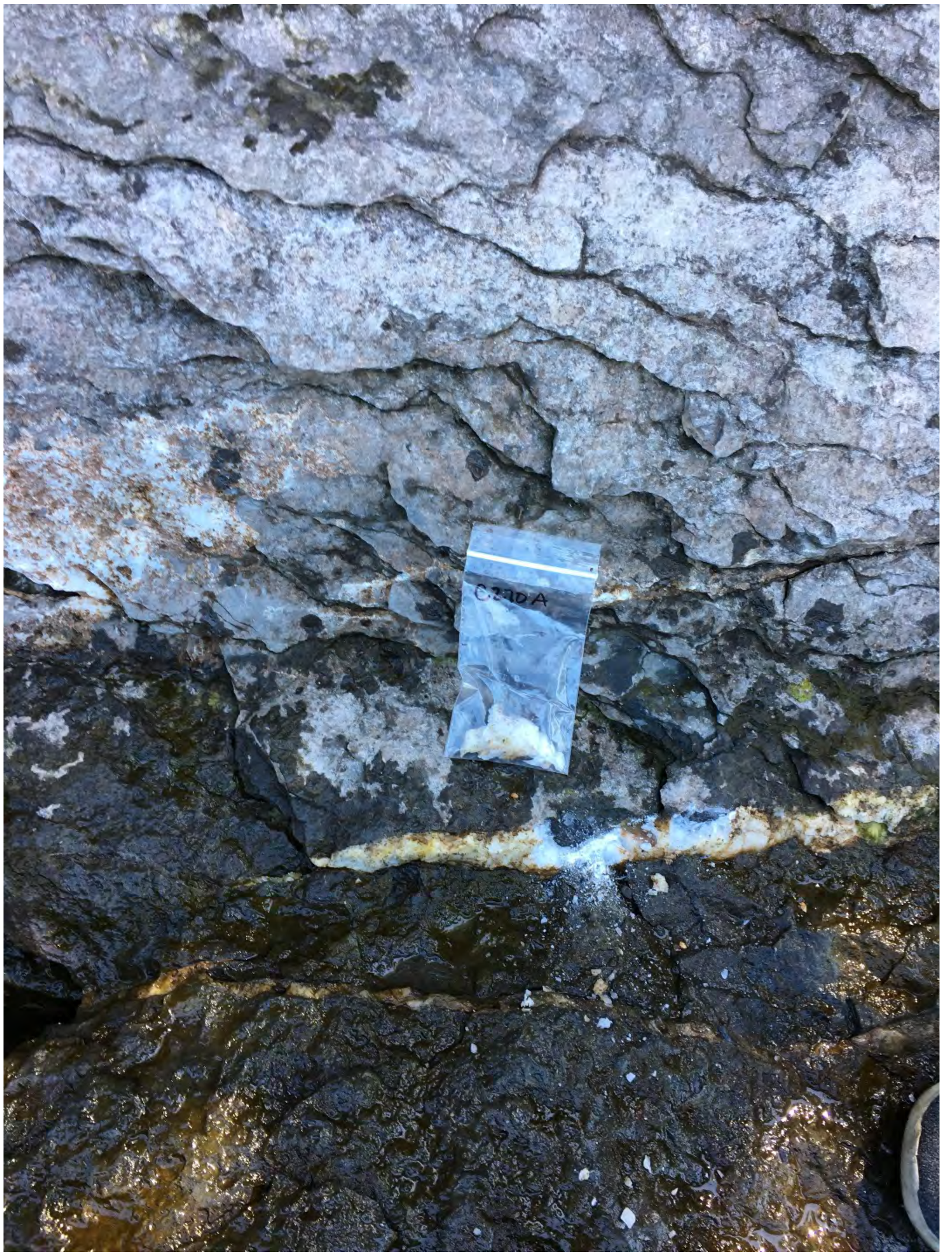

Photos of C270B:

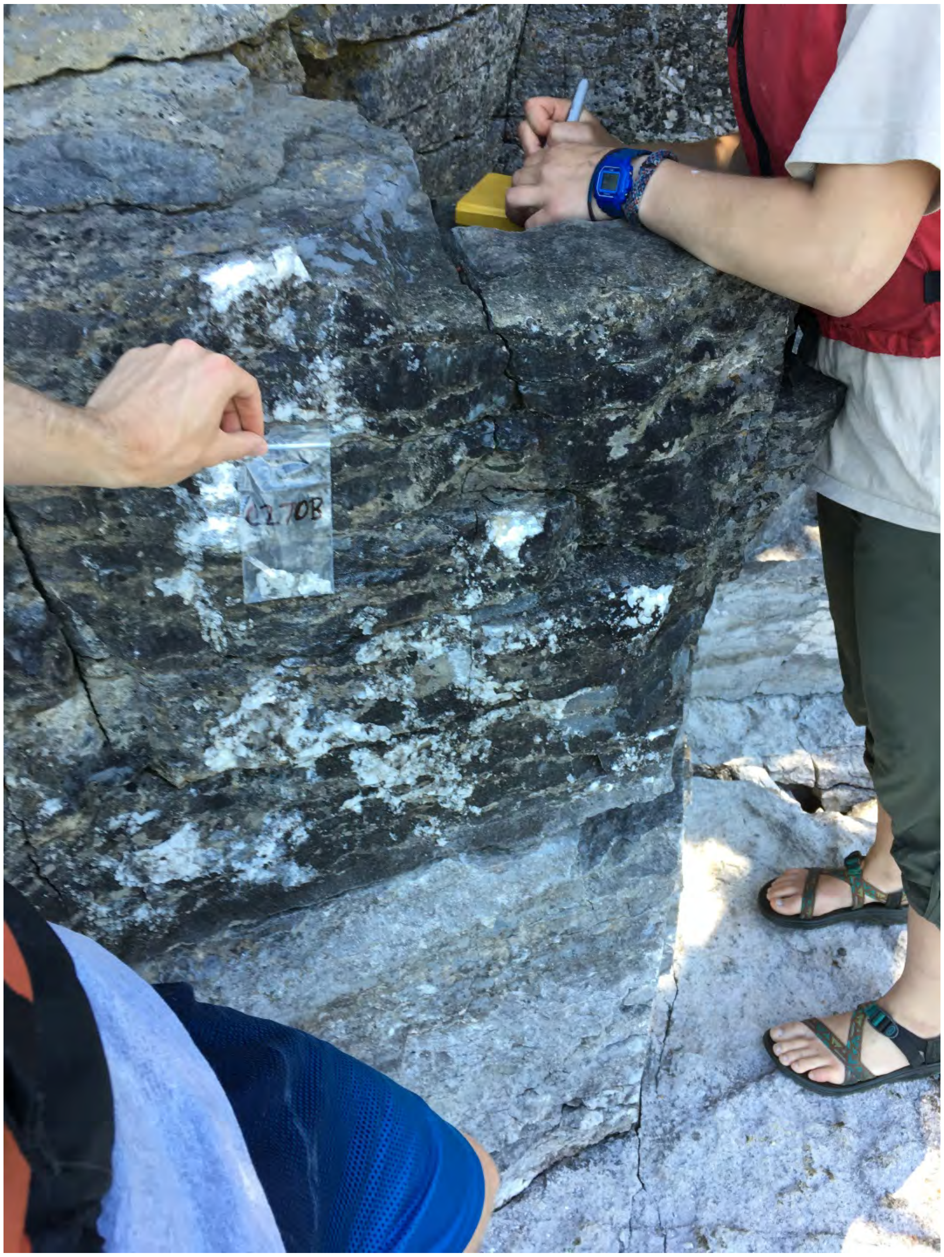

C270C:

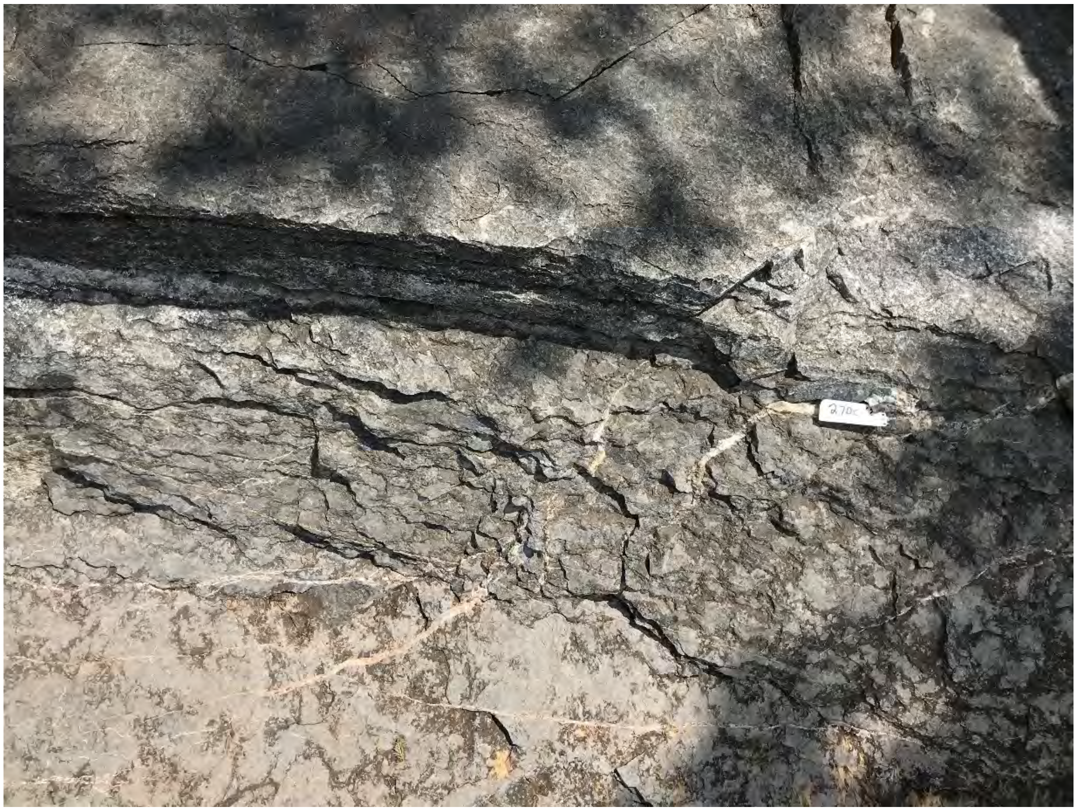

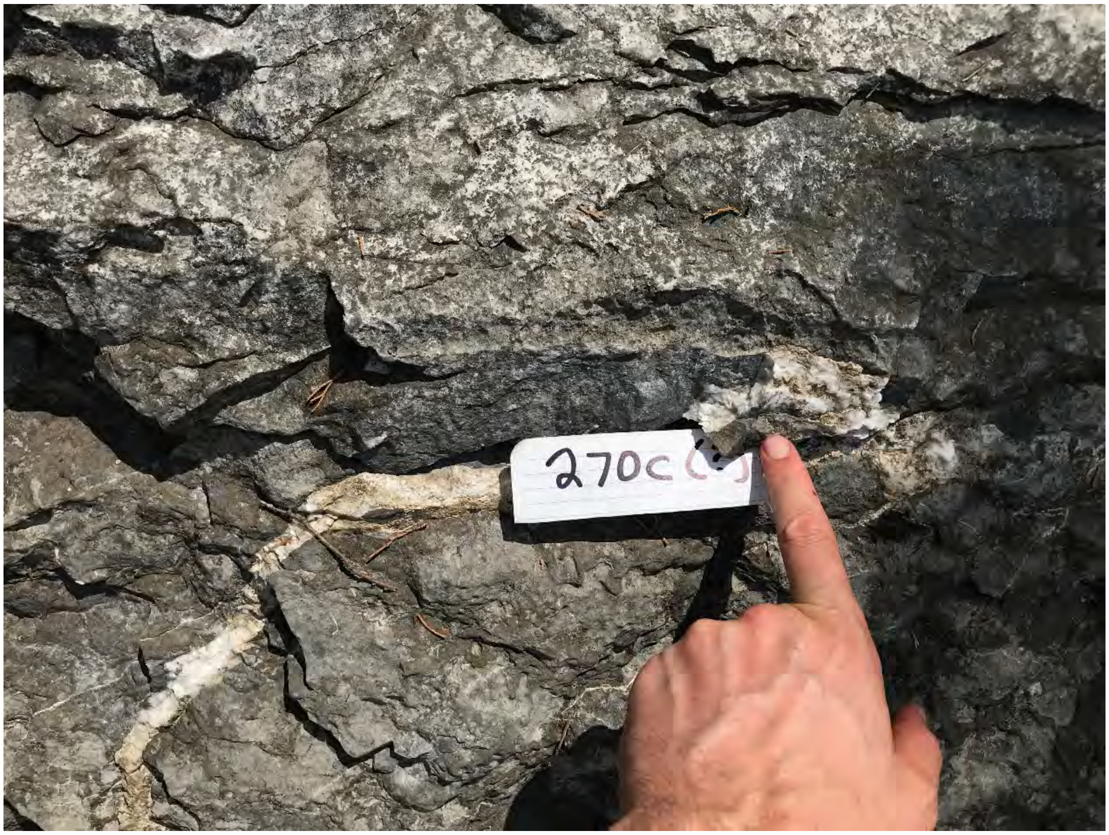

C270D: No slip. Roughly NW-SE striking (strike 325), interpreted as co-magmatic, cut by younger

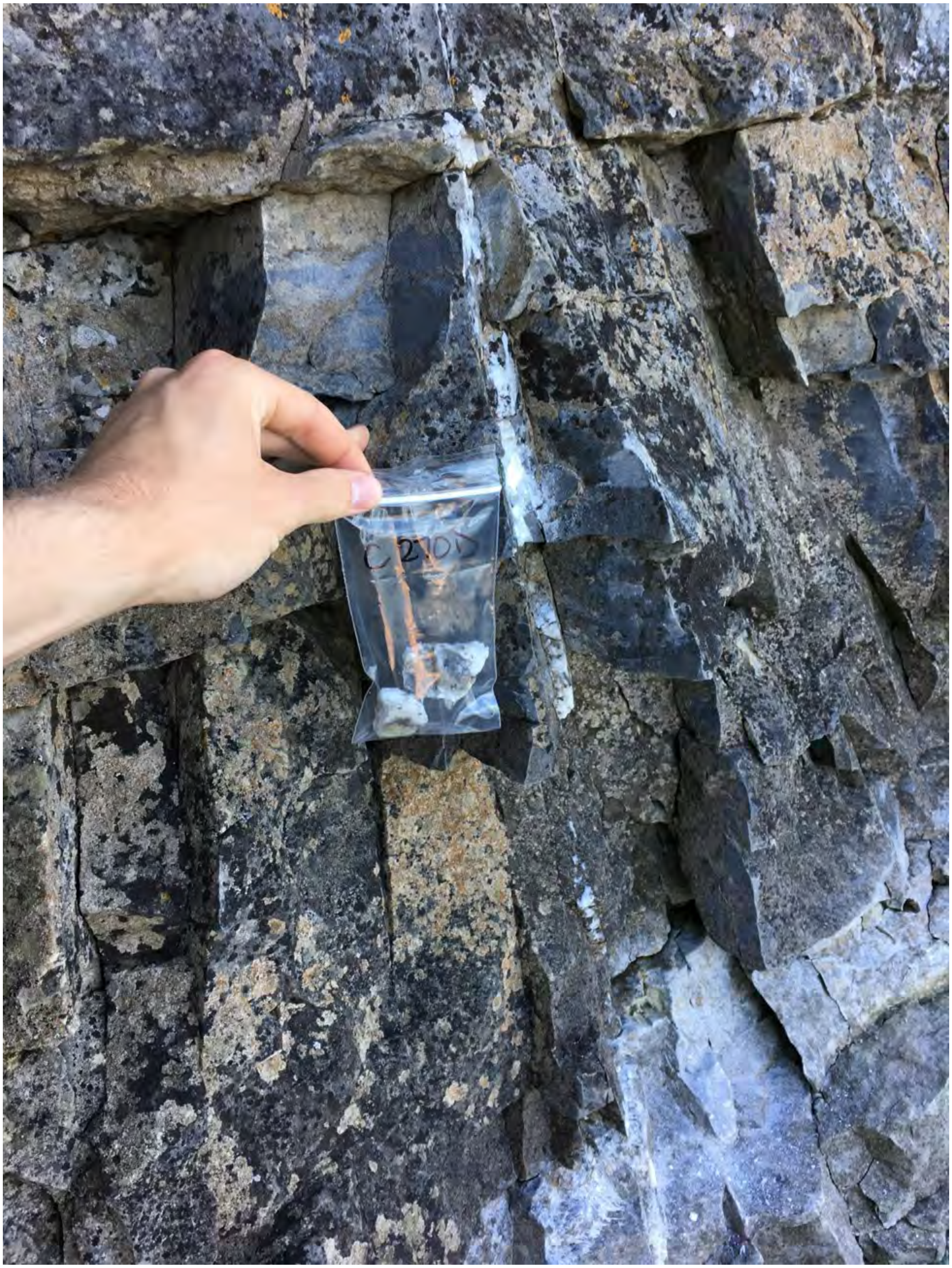
C 270D

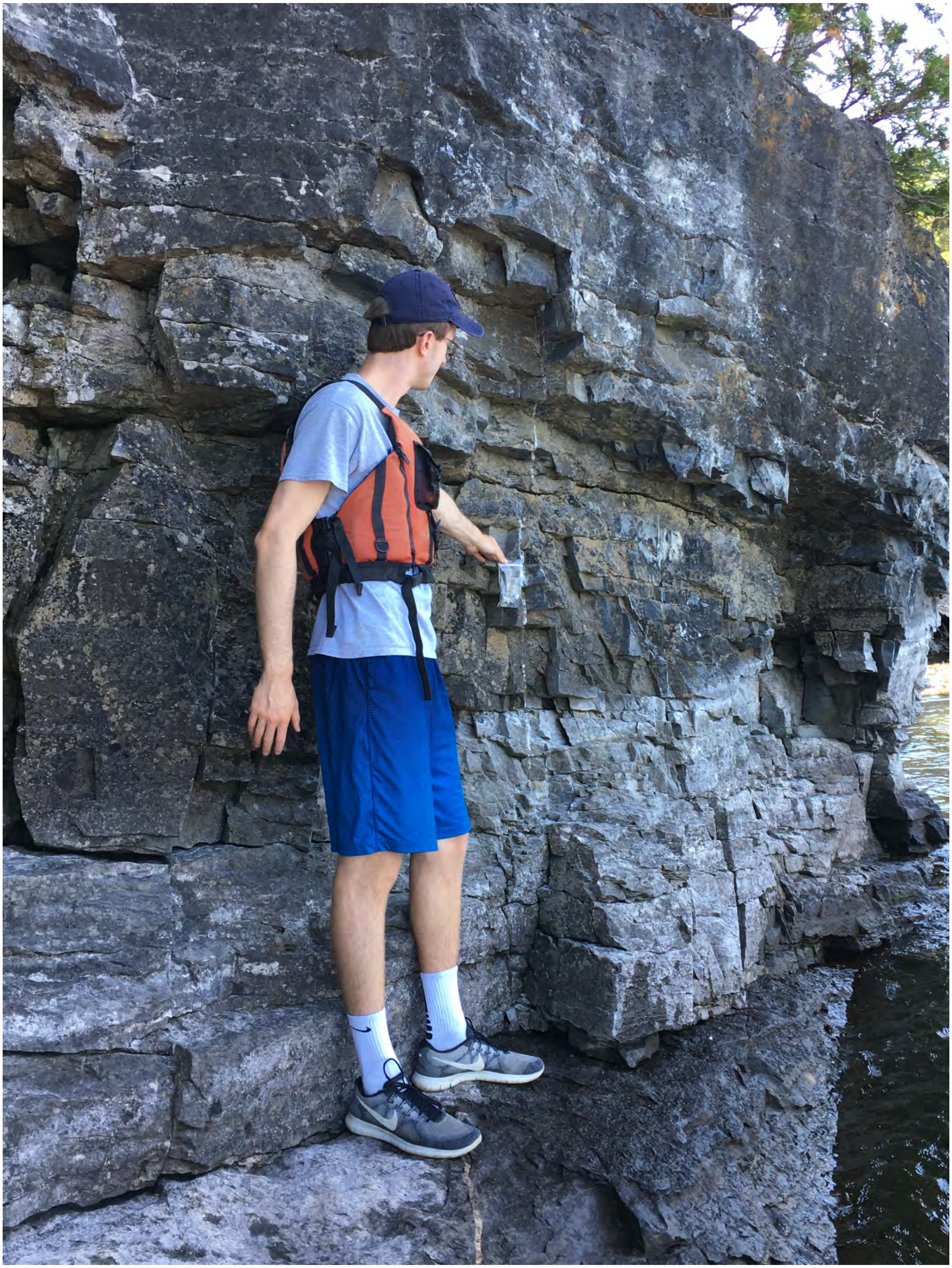

C270E: part of fracture set 2

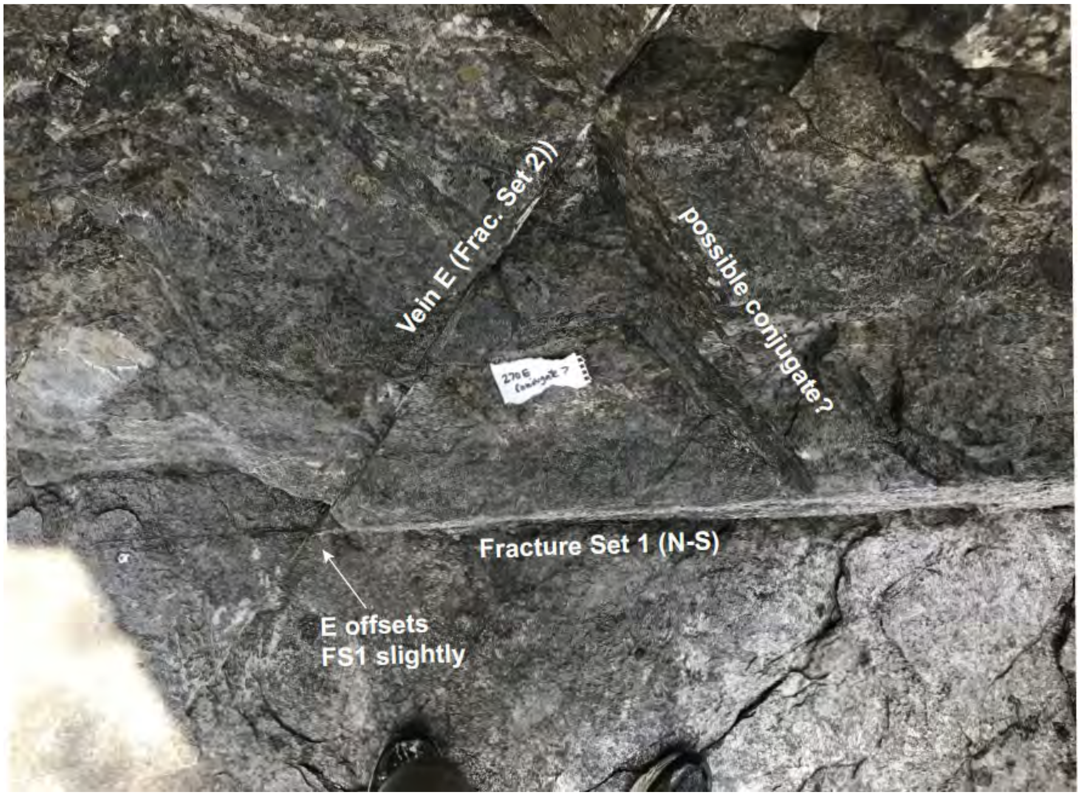

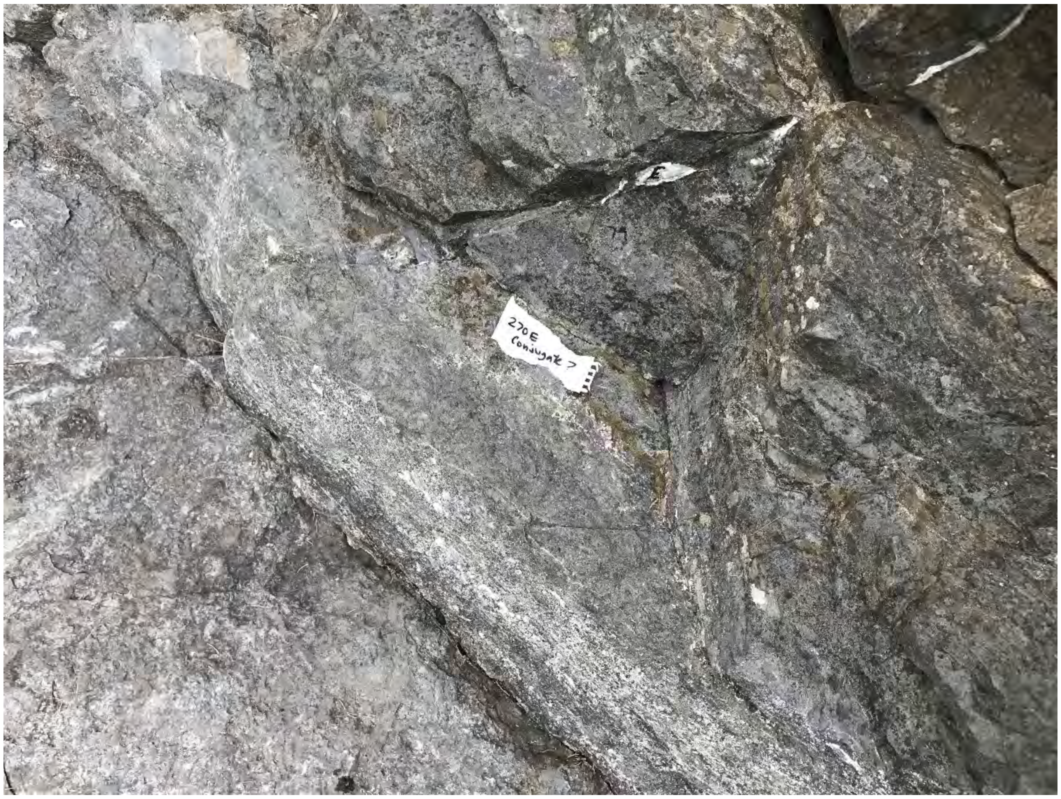

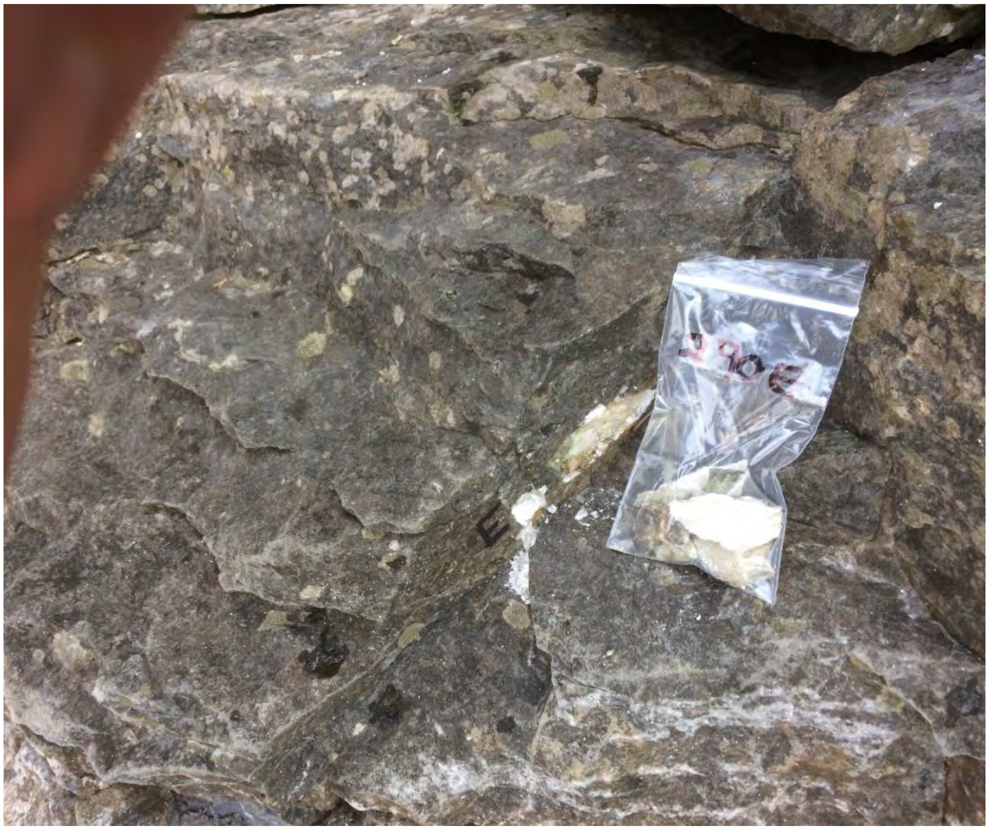

C270G:

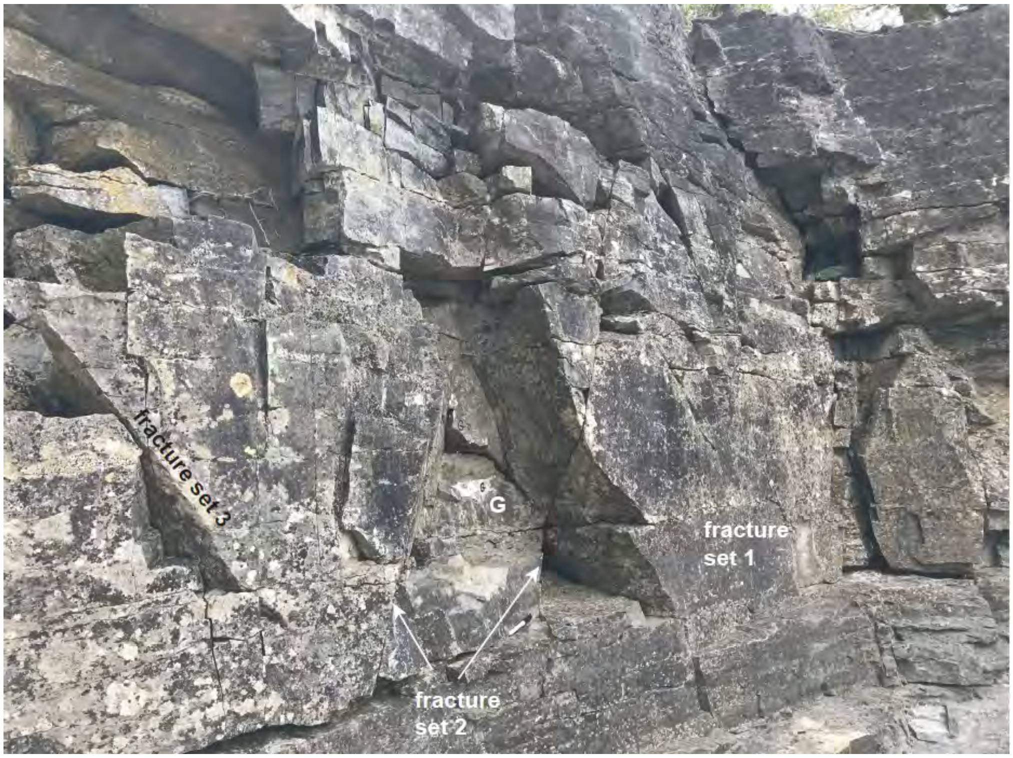

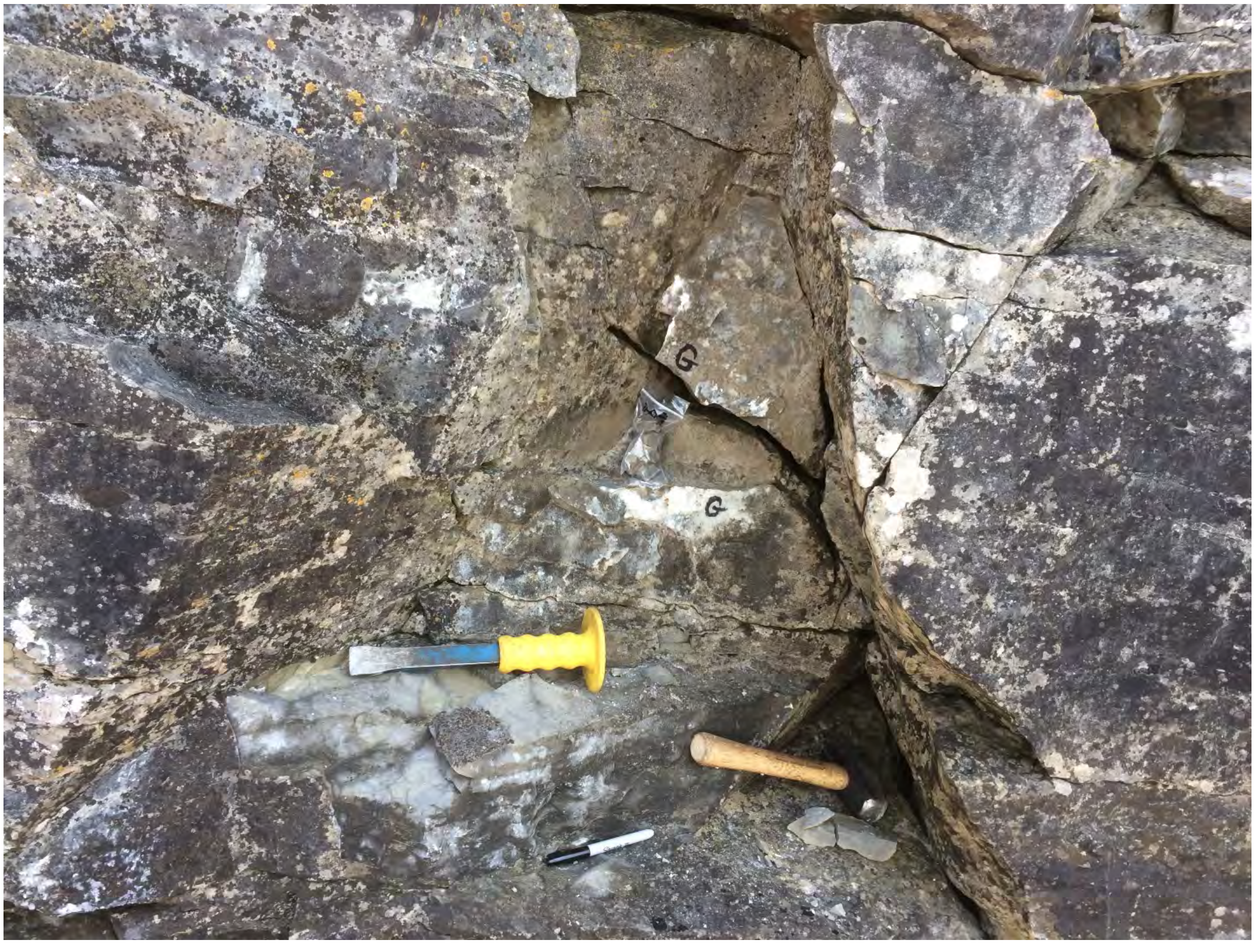
G
G

Lab Description:

C270A: Circles denote target areas for U-Pb dating, not laser shots

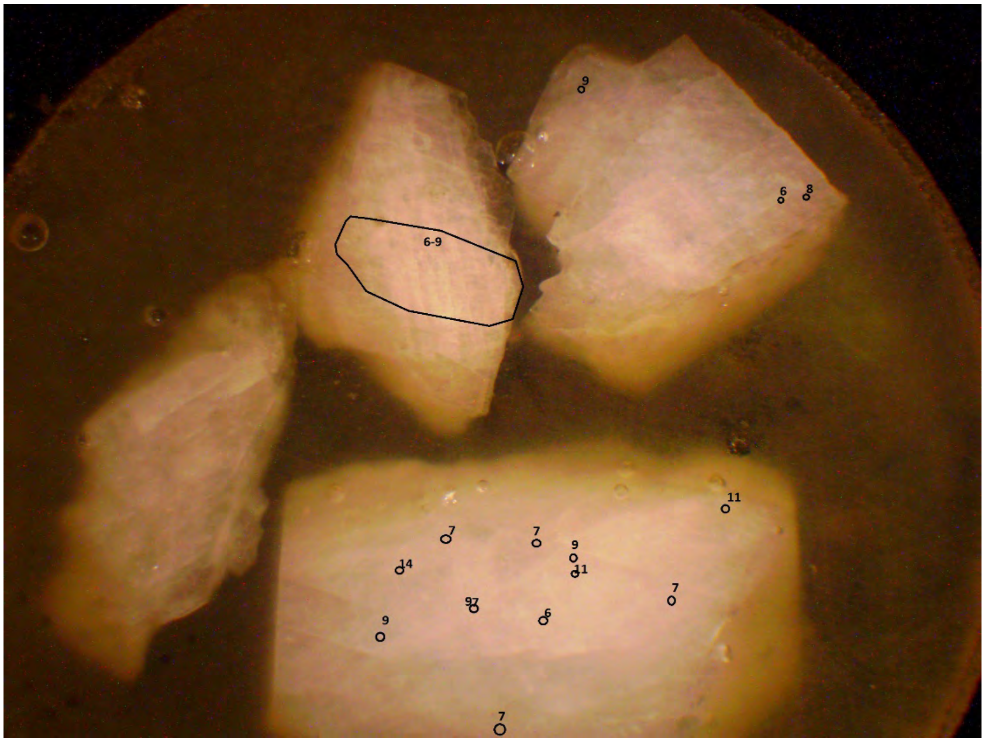

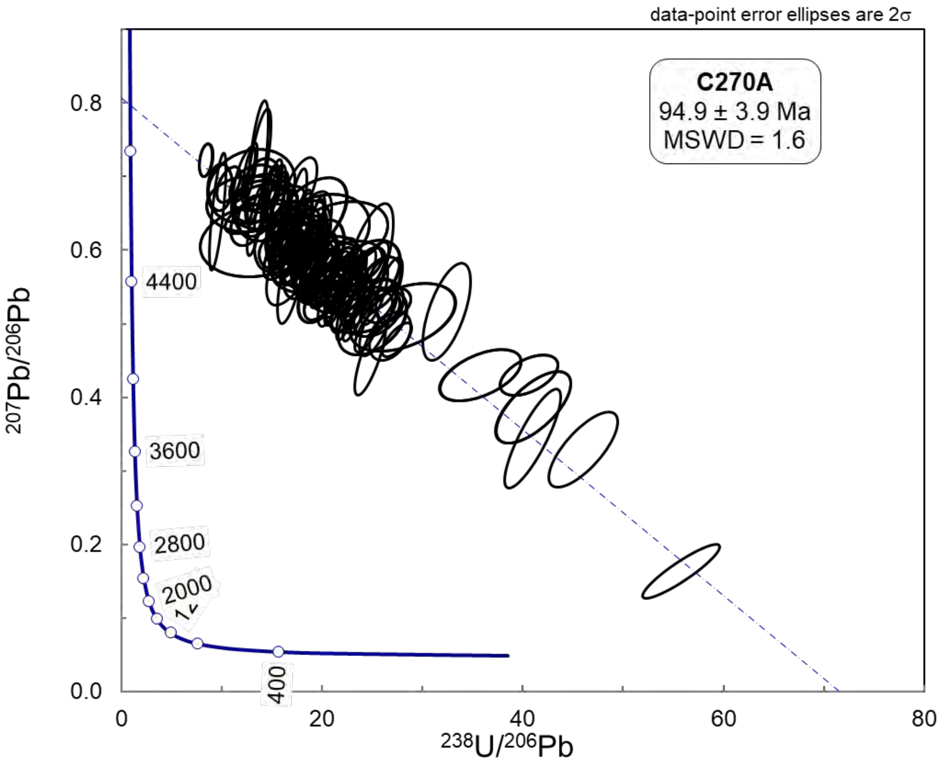

C270B: Circles denote target areas for U-Pb dating, not laser shots

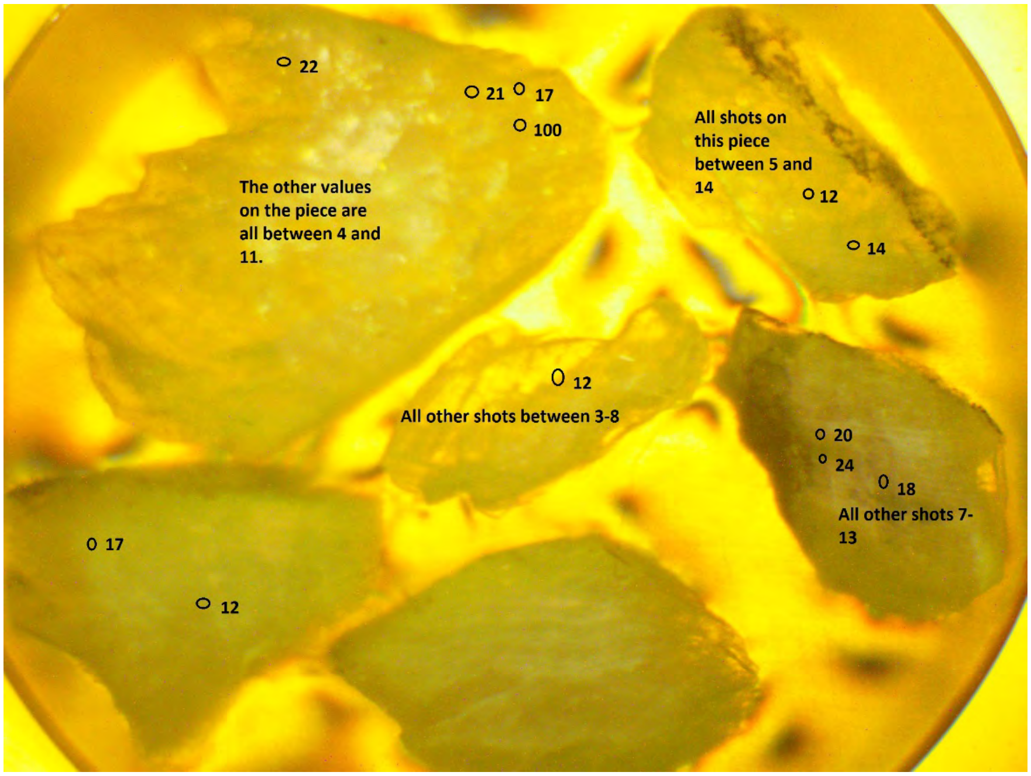

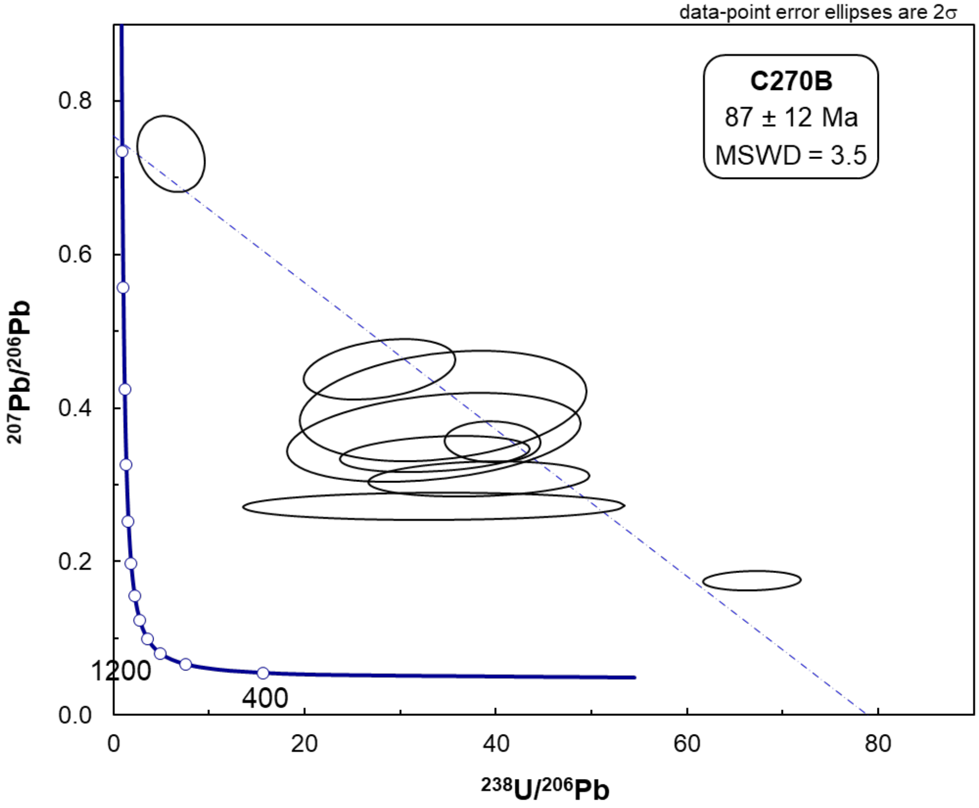

C270C: Circles denote target areas for U-Pb dating, not laser shots

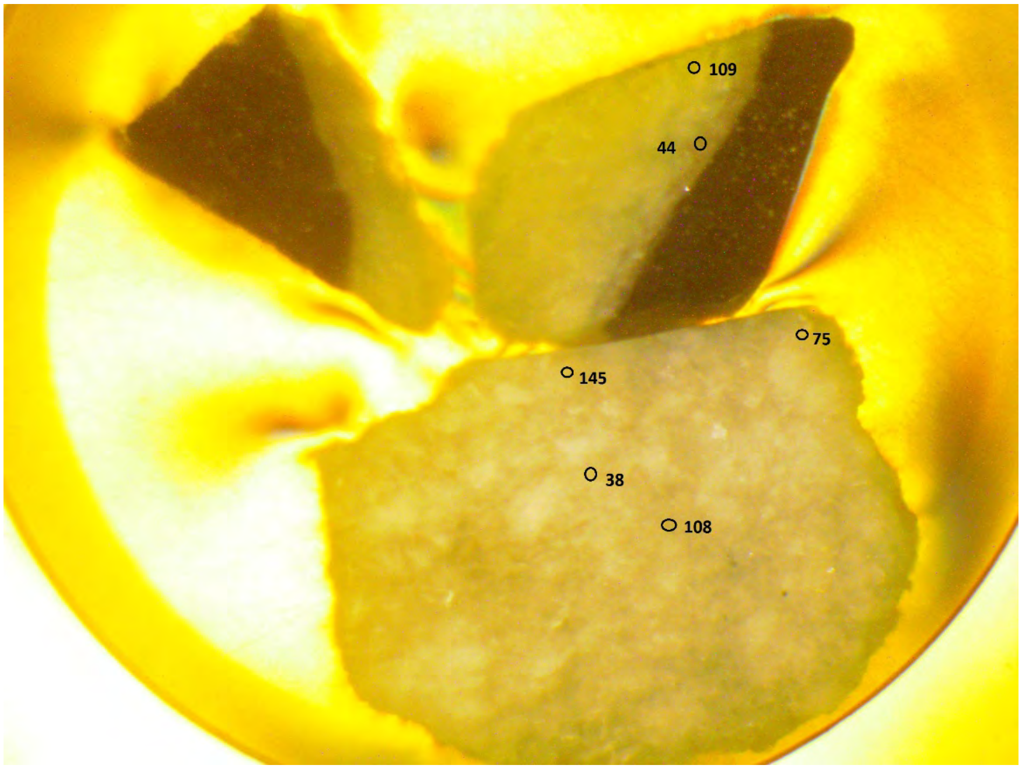

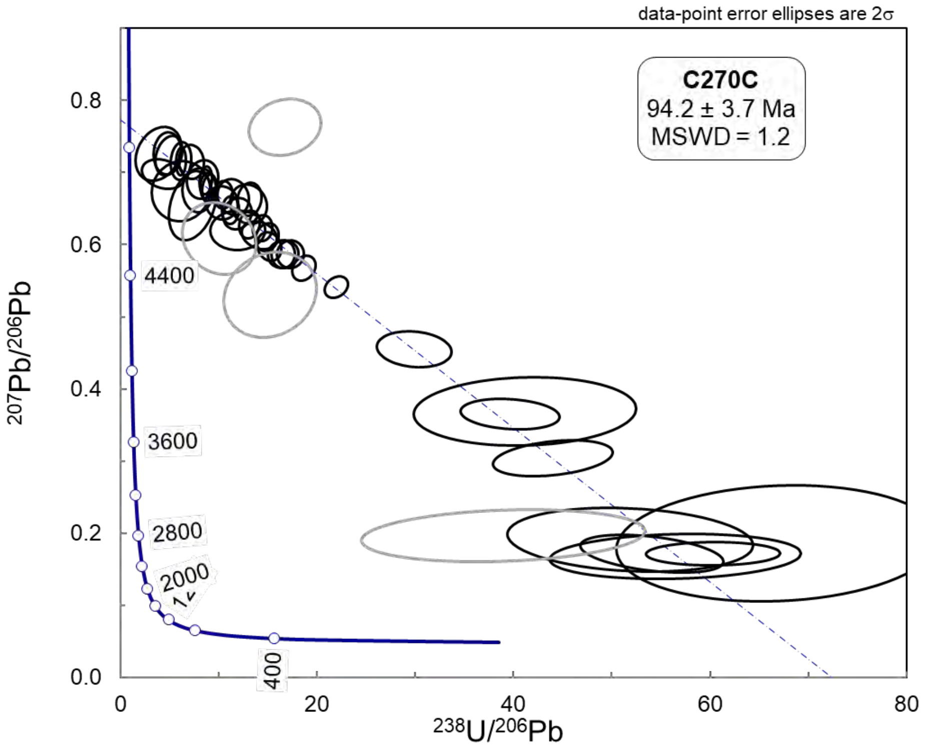

C270D: Circles denote target areas for U-Pb dating, not laser shots

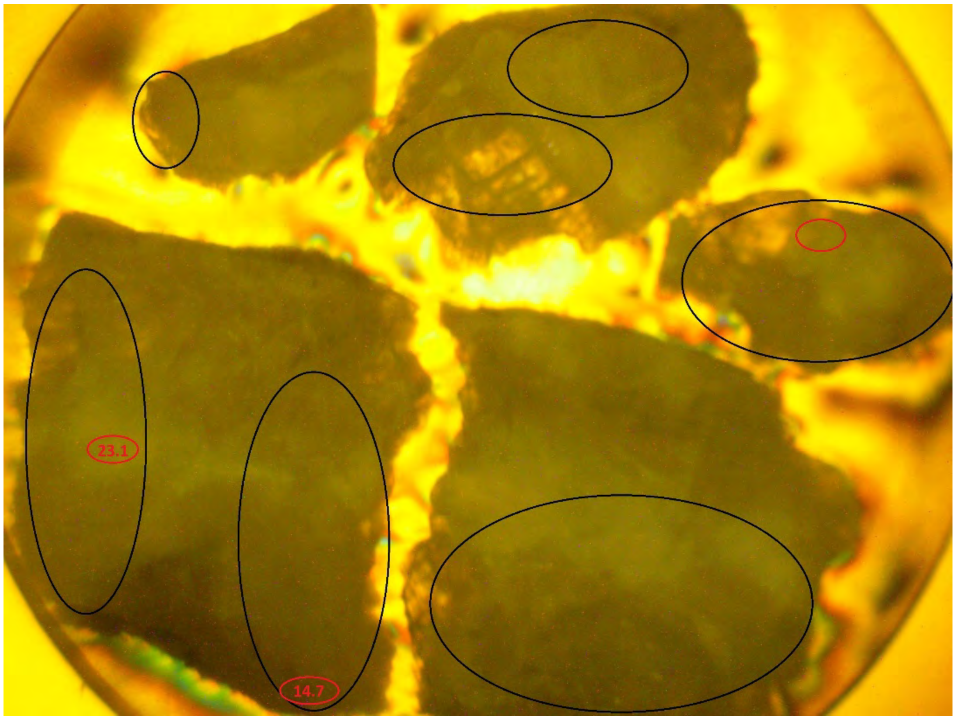

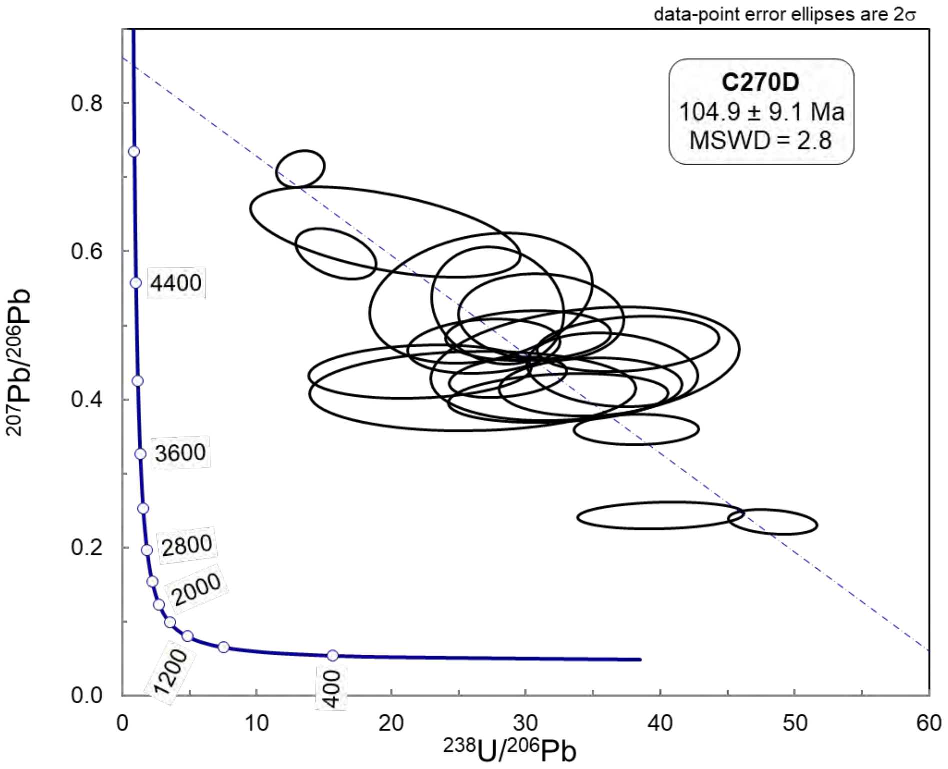

C270E: Circles denote target areas for U-Pb dating, not laser shots

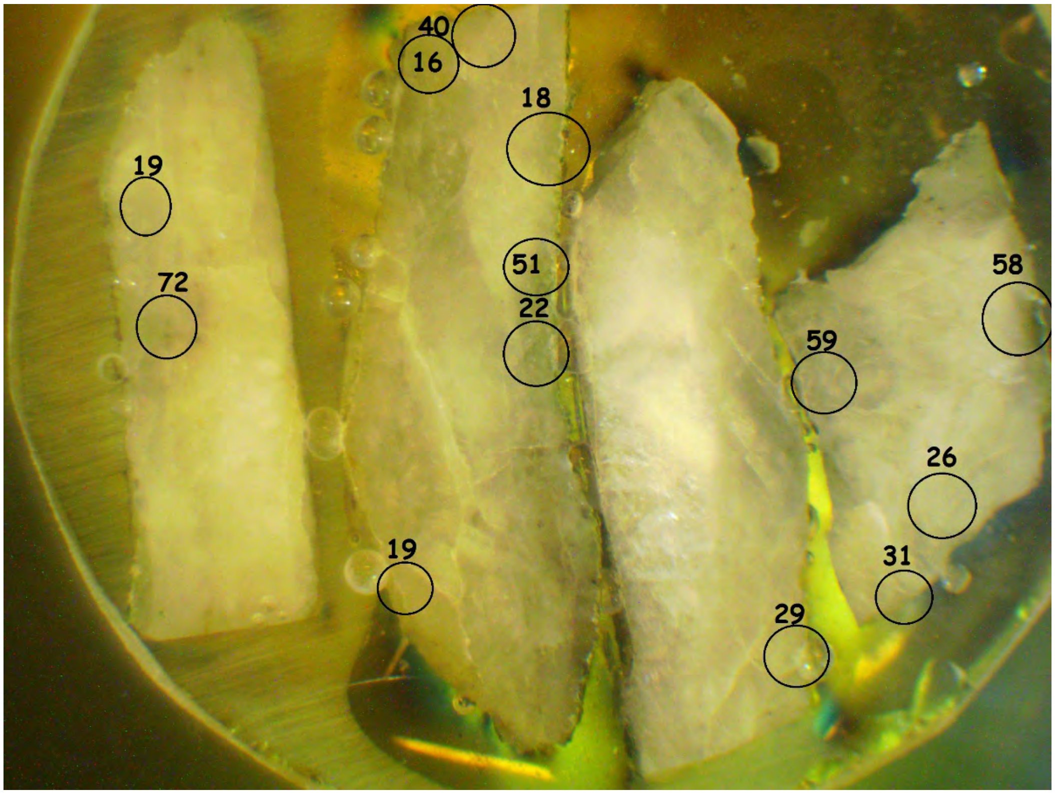

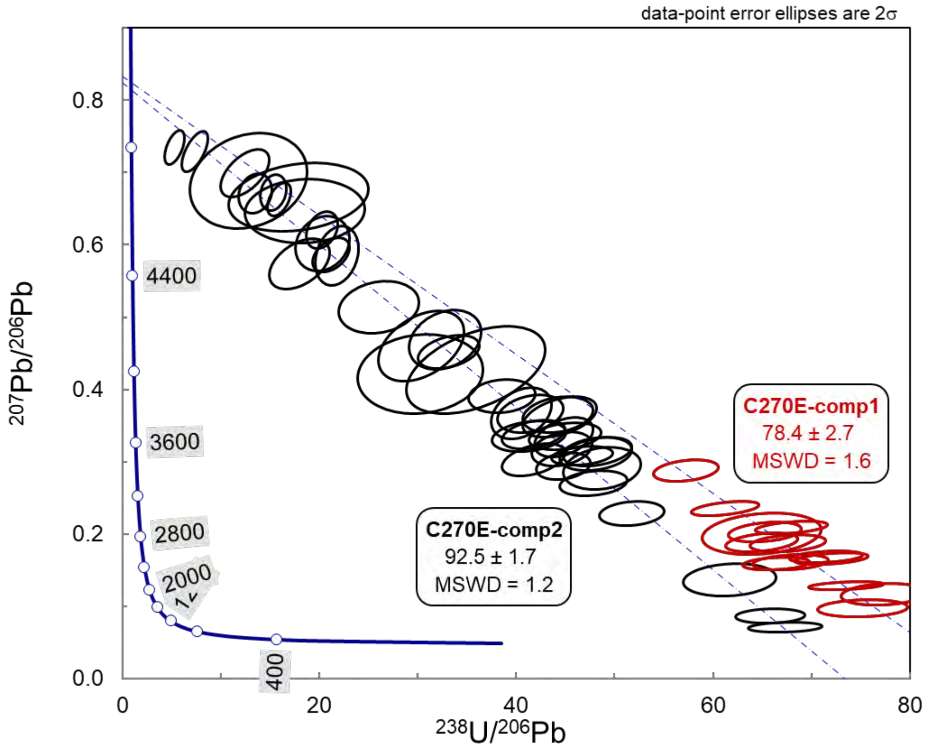

C270G:

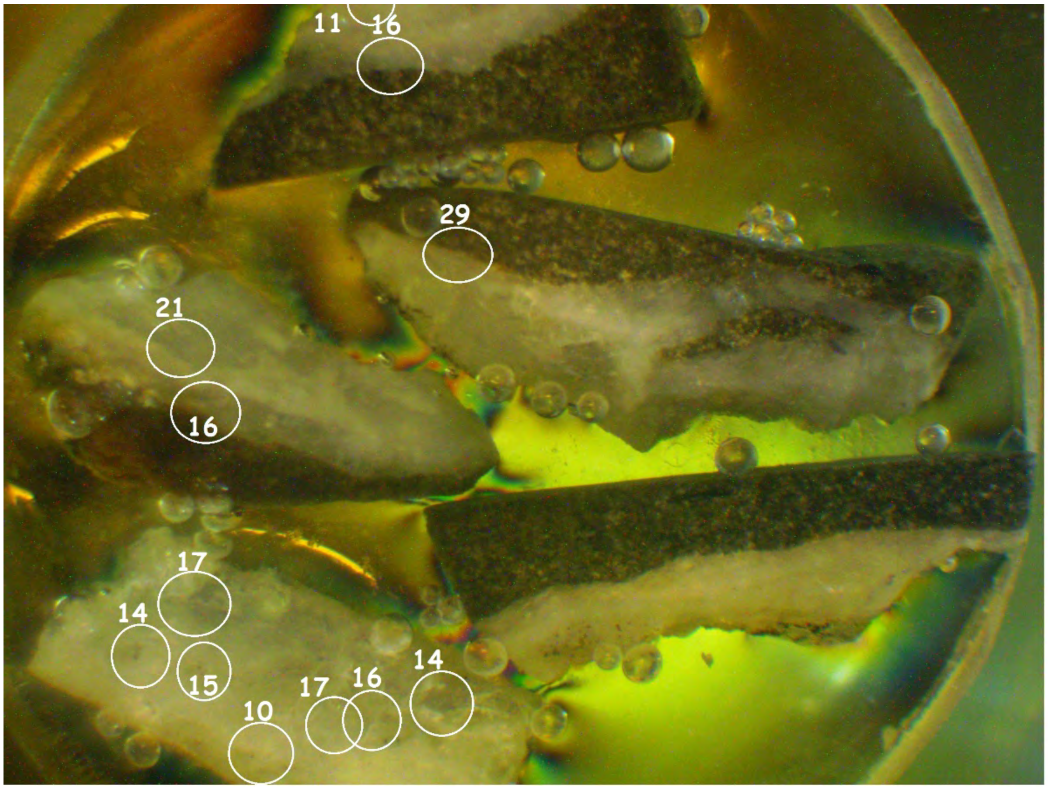
11
16
29
21
16
17
14
15
10
17
16
14

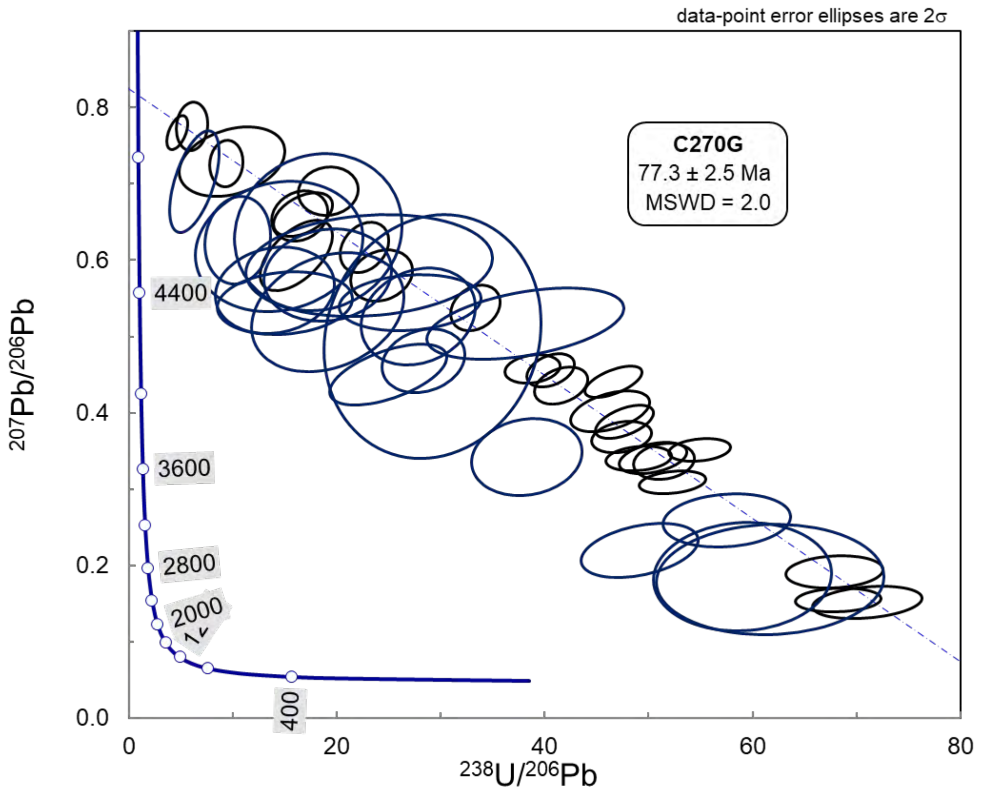
data-point error ellipses are 2σ
C270G
77.3 ± 2.5 Ma
MSWD = 2.0
4400
3600
2800
2000
400
0.0
0.2
0.4
0.6
0.8
0
20
40
60
80
207Pb/206Pb
238U/206Pb
