# Supplementary material for: Pace of passive margin tectonism revealed by U-Pb dating of fracture-filling calcite
Source: Nat Commun. 2022 Apr 12;13:1953. doi: 10.1038/s41467-022-29680-z (PMC9005700; doi:10.1038/s41467-022-29680-z)
Supplement: Supplementary file 6 — Source Data [file 41467_2022_29680_MOESM6_ESM.zip › 1.zip!/appendix2_site_summaries_1to20/3_route7_ferrisburgh_final.pdf]

3: Route 7, Ferrisburgh, VT (44.19292,-73.24796)

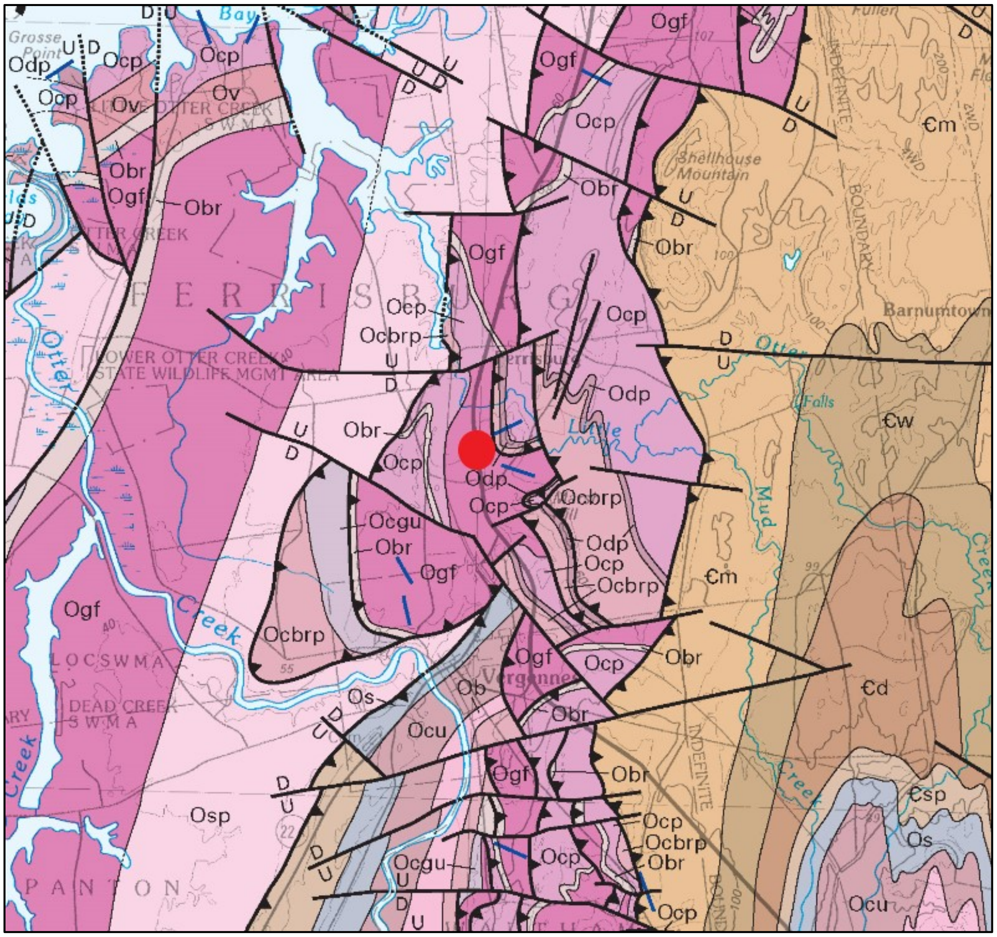

Number of failed samples from same site: none

Bedrock Unit: Glens Fall Limestone (Ogf)

Ages: **C22**: 8.0 ± 5.2 Ma (combination of two separately prepared samples)

Summary: This roadcut is located along the east side of route 7, roughly 1 mile south of the stoplight in Ferrisburgh. Unfortunately the exact location of this sample within the outcrop was not recorded and we are thus unable to draw detailed conclusions from it. However, the outcrop is composed of shattered Glens Falls Limestone cross-cut by numerous small faults. Given the close location to Satterly Quarry (site 6), it is probable that this sample records calcite crystallization during the same Miocene event that affected that site. The age itself is based on separate analytical sessions on two separately prepared samples and yields a very high quality isochron age without culling of any data points. Texture of dated portion of crystal suggests it could be a 2nd crystallization event. Additional elemental mapping of the sample is needed to determine if it is primary or secondary, although the result would not change the interpretation because we lack a detailed field location for this sample.

Field Descriptions:

None available beyond what is summarized above

<u>Lab Description</u>:

C22: Analyzed twice on two separate mounts. Photo below is 2nd mount, no photo of 1st. Texture suggest date is likely a recrystallization event.

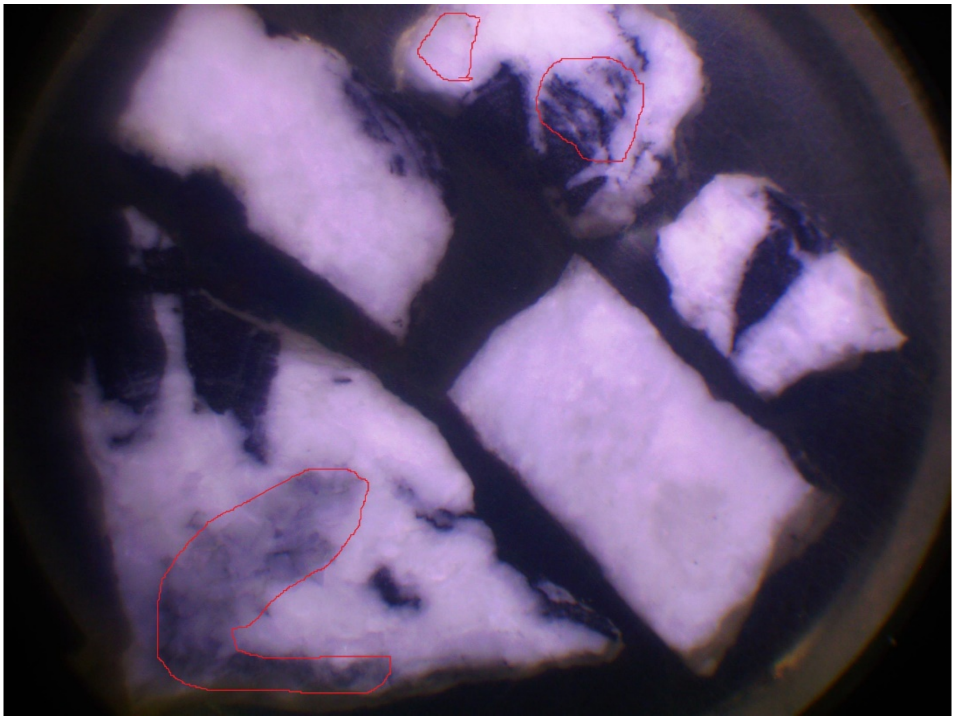

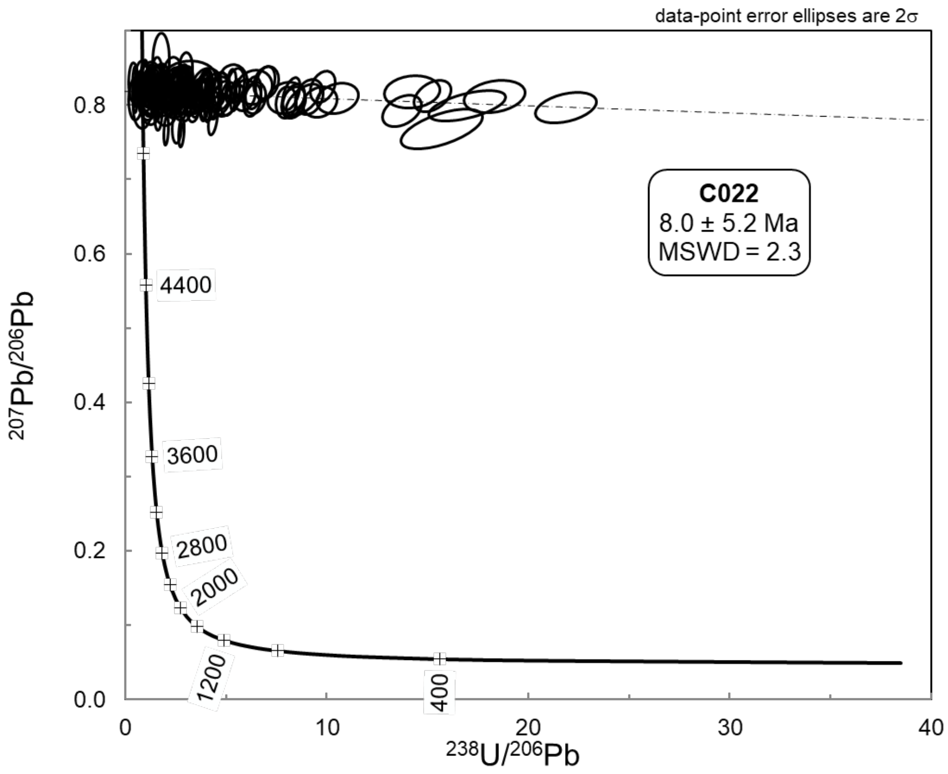

data-point error ellipses are 2σ
C022
8.0 ± 5.2 Ma
MSWD = 2.3
4400
3600
2800
2000
1200
400
0.8
0.6
0.4
0.2
0.0
0
10
20
30
40
207Pb/206Pb
238U/206Pb
