# Supplementary material for: Pace of passive margin tectonism revealed by U-Pb dating of fracture-filling calcite
Source: Nat Commun. 2022 Apr 12;13:1953. doi: 10.1038/s41467-022-29680-z (PMC9005700; doi:10.1038/s41467-022-29680-z)

4: Highway 125, Cornwall, VT (44.00338, -73.22088)

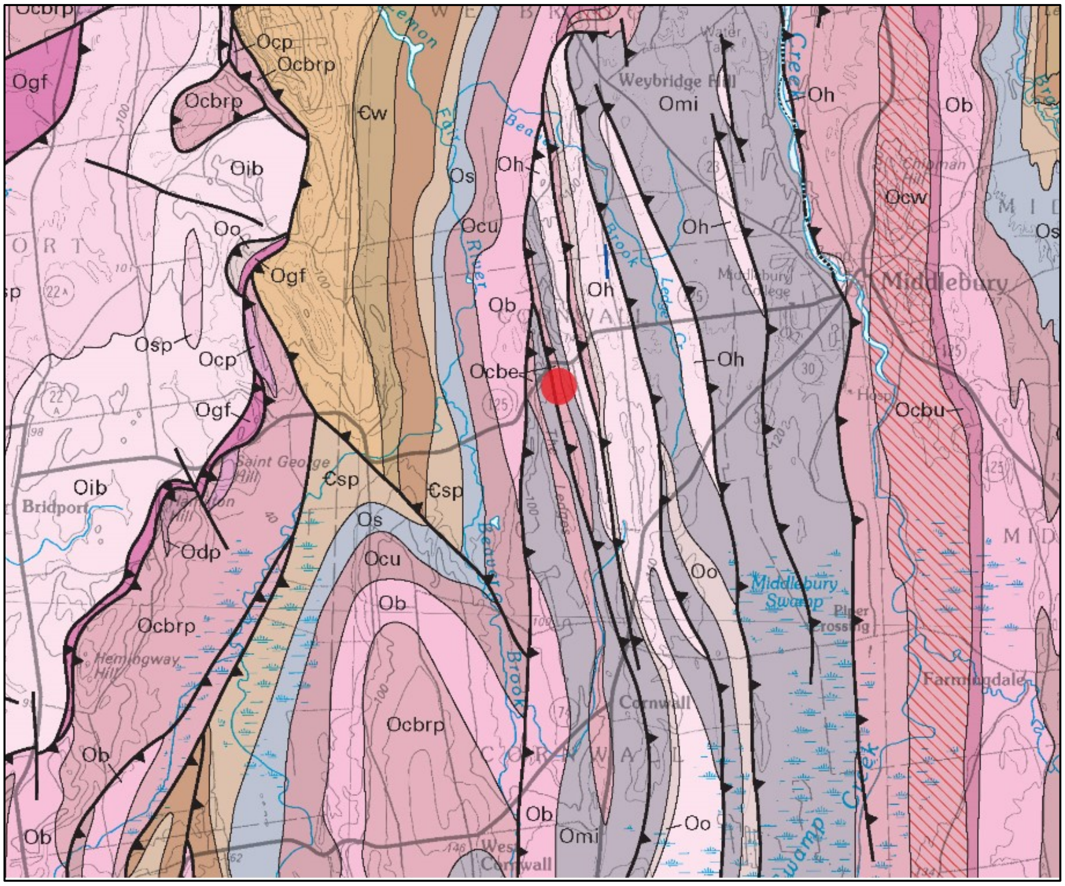

Figure 1: Sample location on state geologic map of Ratcliffe at al. (2011)

<u>Bedrock Unit:</u> Midldebury Limestone (Omi)

<u>Failed samples from same site:</u> C106B,C,D,E,F; 138A,B; 139; 140A,B,C,D; 141A,B (13 samples)

<u>Ages</u>: C106: 8 ± 39 Ma; **C106A**: 3.1 ± 4.2 Ma **C106G**: 4.9 ± 3.8 Ma

<u>Summary</u>: This is a roadcut along the north side of Highway 125 about 2 miles west of Middlebury College (red dot on map is slightly misaligned). Although this outcrop is structurally complex, we obtained dates only on one subset of features, a set of 1-4 cm thick calcite veins striking roughly N-S and dipping roughly 27° west. Several lines of evidence suggest these formed as dilational features under high fluid pressures, including: 1) abundant thick, sparry calcite, 2) sharp linear contacts with host rock, 3) lack of slip indicators, and 4) confinement within a mechanically rigid dolostone layer (see photos).

In each of the dated samples, the high $^{238}U/^{206}Pb$ shots comprising the preferred isochron age come from a limited subset of the sample, whereas most parts of the sample are characterized by low $^{238}U/^{206}Pb$ ratios that could potentially originate from an earlier undateable crystallization event. In particular, closer examination of sample C106A shows that most high ratio shots come from a younger generation of optically clear calcite intruding the more widespread milky-white coarse-calcite veins. It is unclear whether both generations of calcite are Mio-Pliocene in age, or whether one is much older.

Thus, this sample is categorized as a secondary crystallization event and we are **unable to document kinematics associated with the Pliocene event at this site** and it should be noted that stable isotope results from this vein are also unlikely to be indicative of the Pliocene event.

The age of sample 106 is highly uncertain but was retained in the dataset because it is consistent with the Miocene ages observed in sample 106A and 106G. The age of sample 106A is estimated from two analytical sessions on two separately mounted specimens of calcite, which yielded a relatively tight isochron age without discarding any data points. Although the high $^{238}U/^{206}Pb$ shots and the resultant Mio-Pliocene age may record a later fluid-flow or recrystallization event, the N-S orientation of these veins suggests they are vulnerable to reactivation by E-W extensional stresses, similar to what has been inferred for other Mio-Pliocene ages in the Champlain Valley.

Structural Summary:

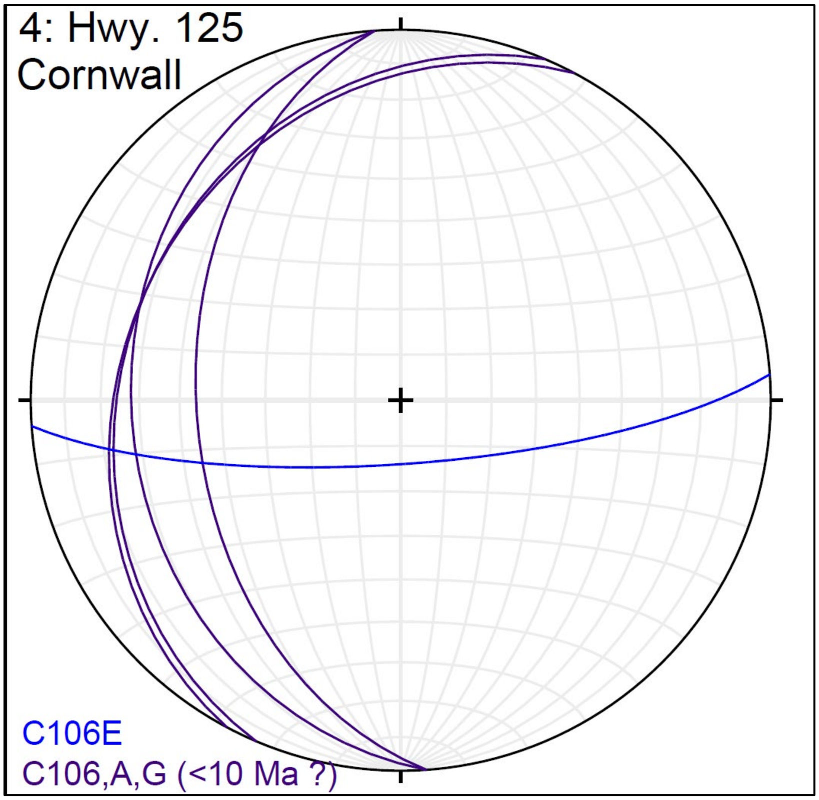

Overview field photos:

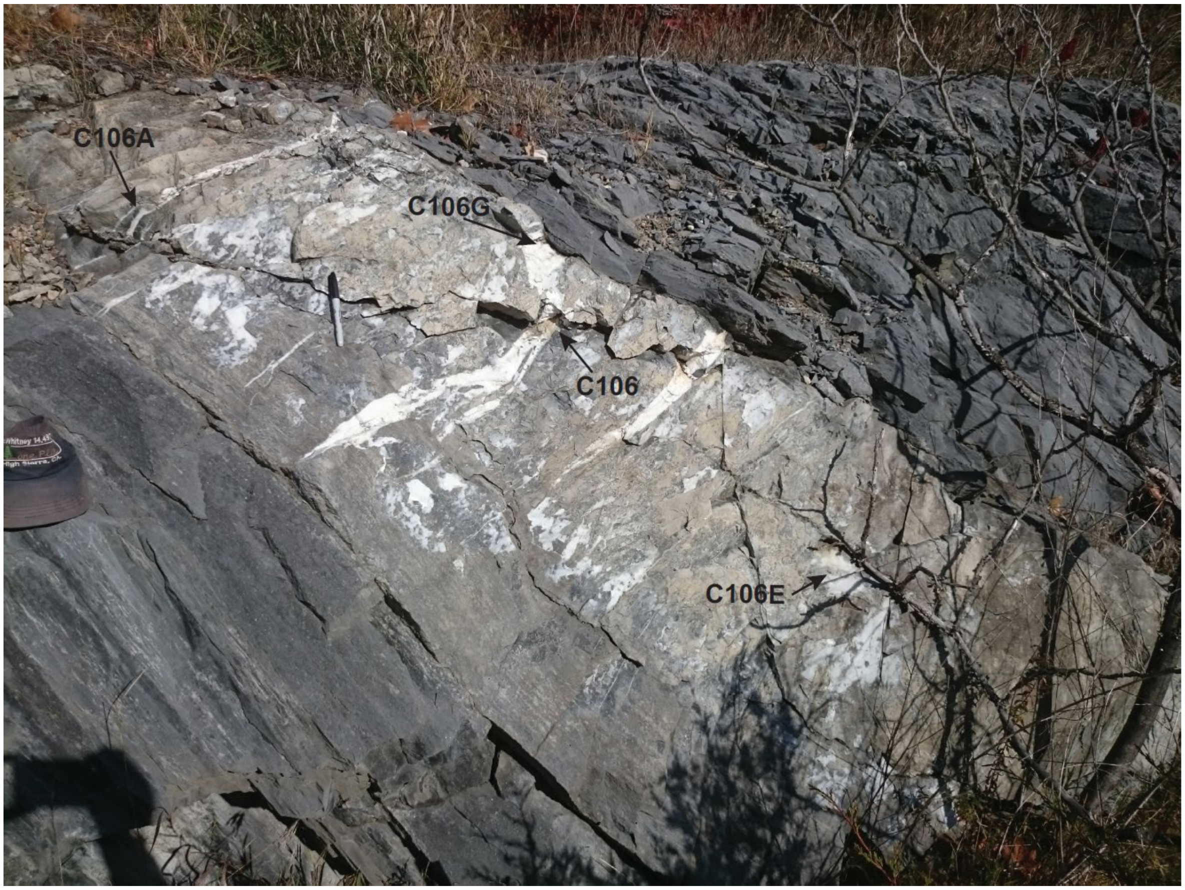

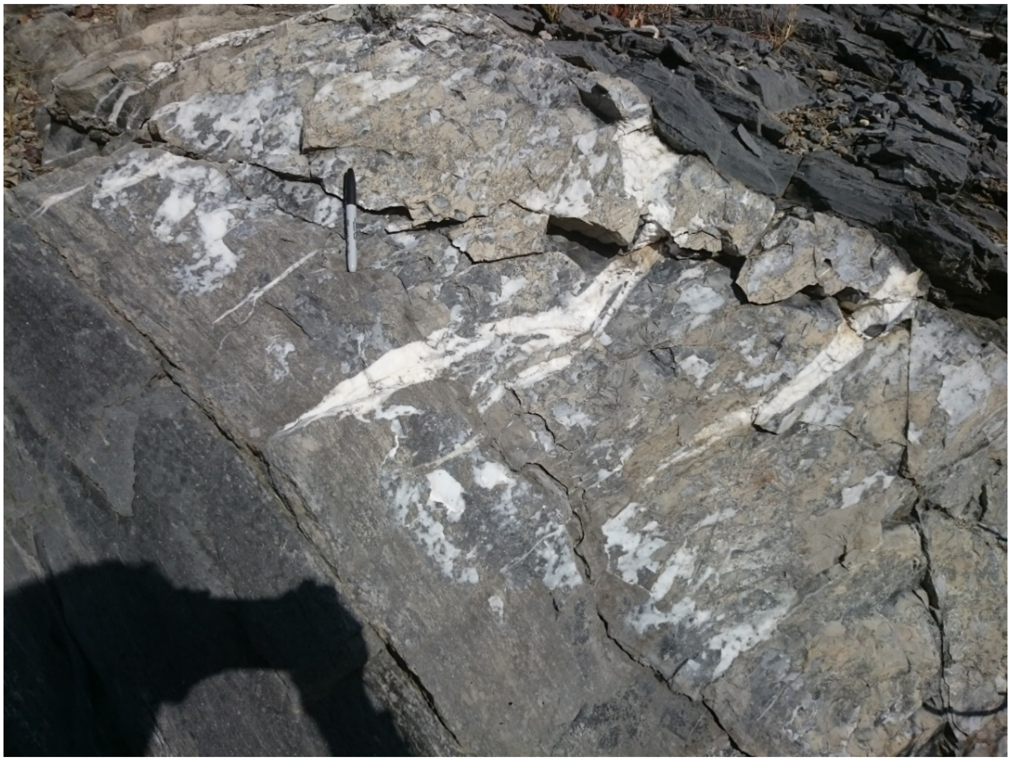

Lab Description:

C106: Analyzed during first visit to UCSB, so no optical image available. Only about 20 total shots taken, very low quality age, but consistent with a Miocene event at this site.

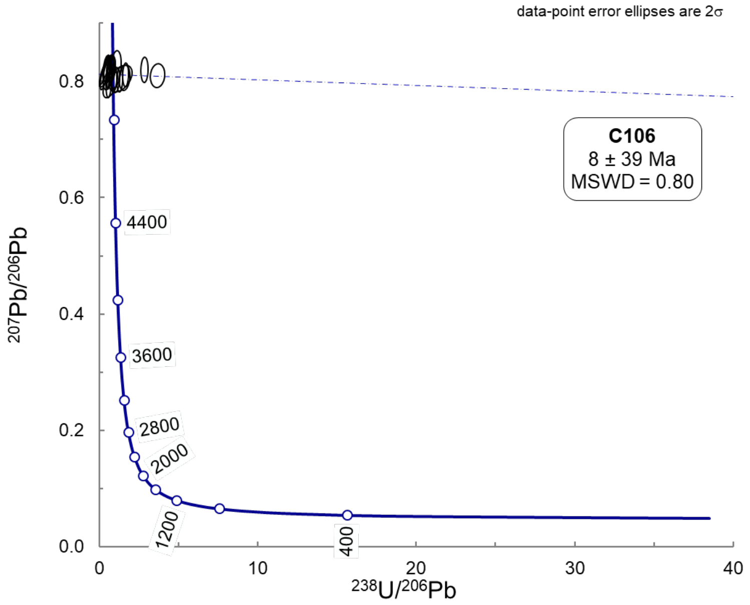

C106A: This sample was mounted and analyzed twice to generate more robust age estimate. Quoted age is a combination of shots from two different sample mounts. Note texture of milky white calcite blocks that appear to be separated by darker 'clear' veins of calcite. Many dateable shots likely came from the 'clear' veins casuing this age to be categorized as secondary crystallization.

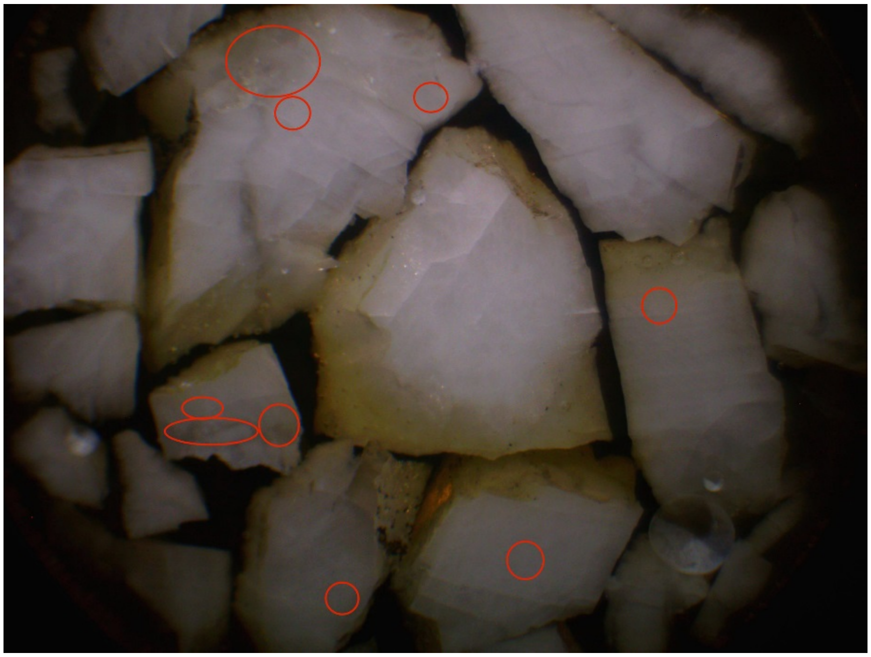

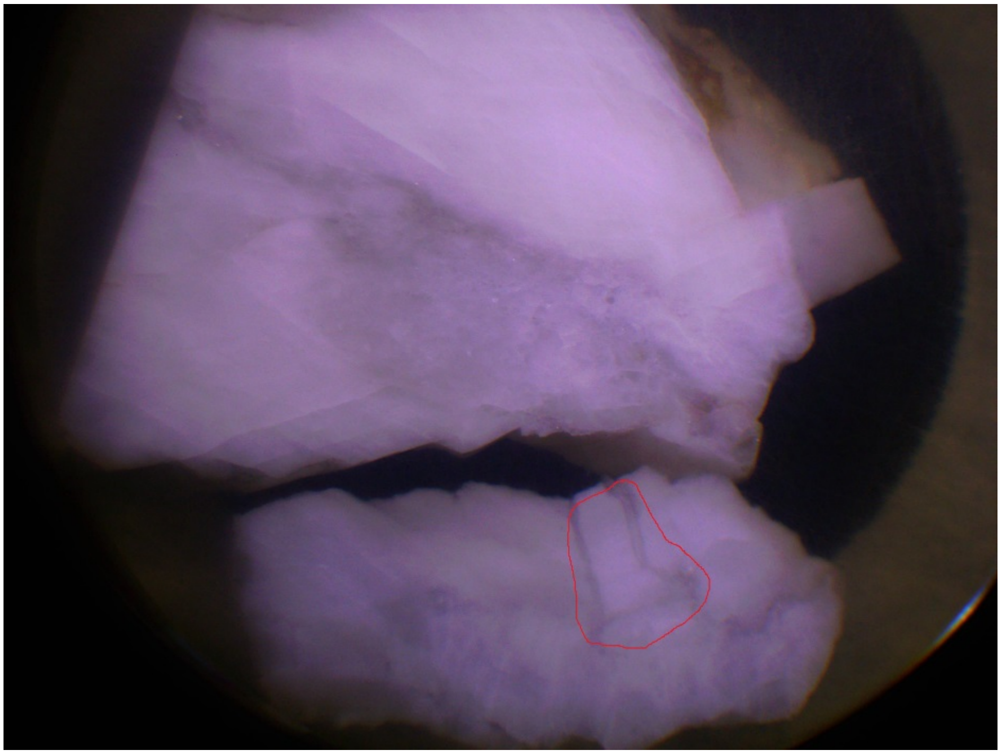

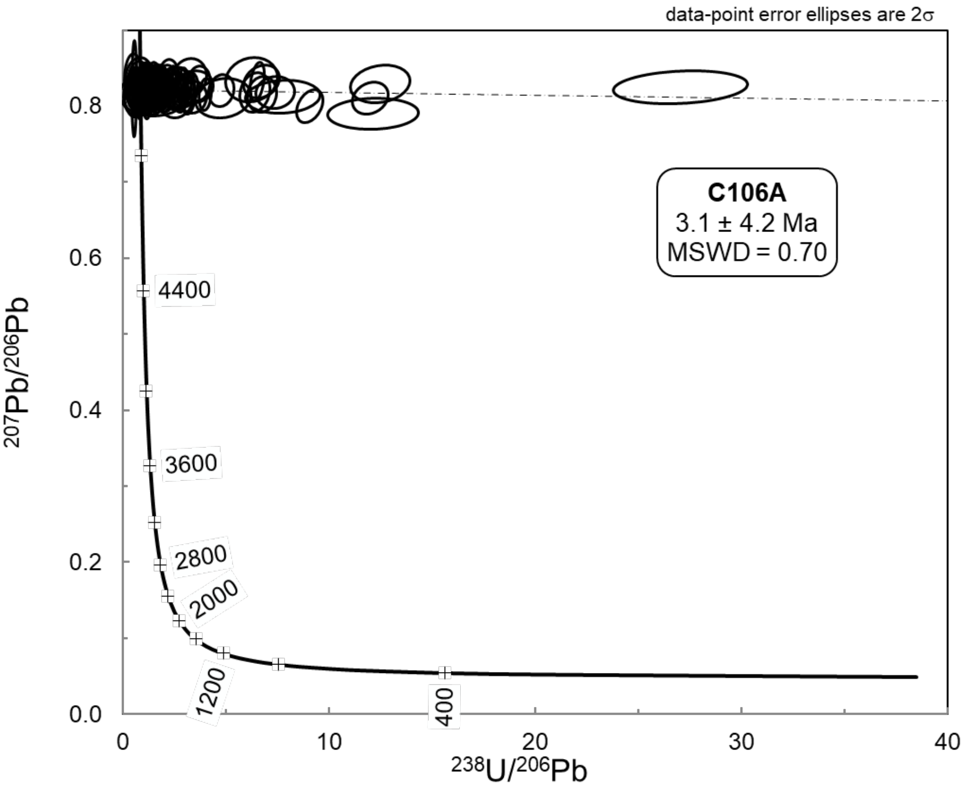
data-point error ellipses are 2σ
C106A
3.1 ± 4.2 Ma
MSWD = 0.70
4400
3600
2800
2000
1200
400
$^{207}Pb/^{206}Pb$
$^{238}U/^{206}Pb$
0.0
0.2
0.4
0.6
0.8
0
10
20
30
40

C106G: Note texture of milky white calcite blocks that appear to be separated by darker 'clear' veins of calcite. Many dateable shots likely came from the 'clear' veins casuing this age to be categorized as secondary crystallization.

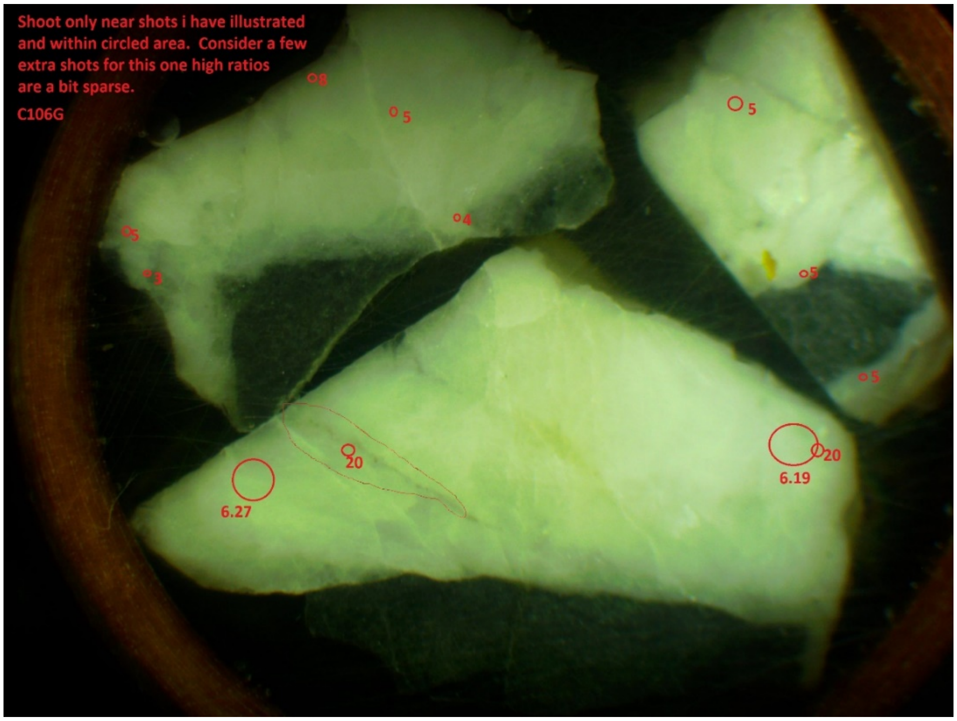

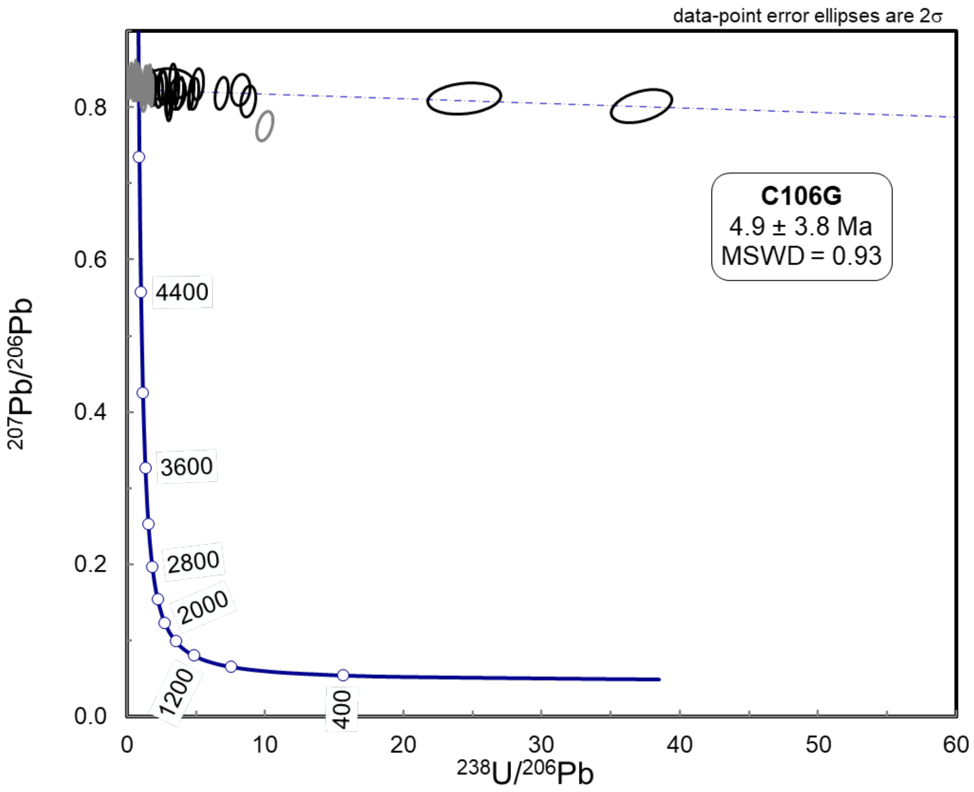

Supplement: Supplementary file 6 — Source Data [file 41467_2022_29680_MOESM6_ESM.zip › 1.zip!/appendix2_site_summaries_1to20/4_Hwy125_cornwall_final.pdf]
