# Supplementary material for: Pace of passive margin tectonism revealed by U-Pb dating of fracture-filling calcite
Source: Nat Commun. 2022 Apr 12;13:1953. doi: 10.1038/s41467-022-29680-z (PMC9005700; doi:10.1038/s41467-022-29680-z)
Supplement: Supplementary file 6 — Source Data [file 41467_2022_29680_MOESM6_ESM.zip › 1.zip!/appendix2_site_summaries_1to20/5_richville_reservoir_north_final.pdf]

5: Richville Reservoir North (43.87347, -73.27134)

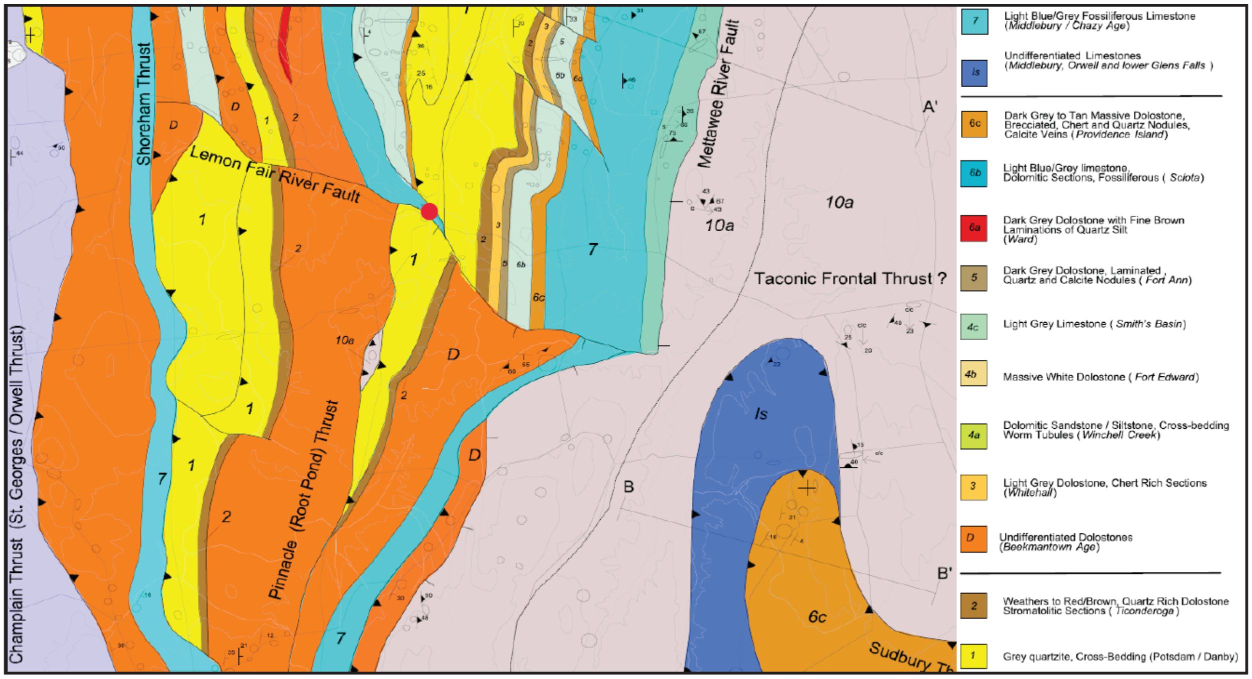

Figure 1: Sample located on geologic map of Hayman and Kidd, 1996.

<u>Failed samples from same site:</u> C127A,B, & D-2, C243B, D

<u>Bedrock Unit:</u> Middlebury limestone (?)

<u>Ages</u>: **C127C**: 85 ± 4 ; **C127D-1**: 80 ± 1.6; **C243A**: 79 ± 1; **C441A**: 97 ± 11

<u>Summary</u>: This site is located along the east side of Shacksboro Rd, about 150 m north of the intersection of Richville Road. This area has been studied in relative detail by Haymann and Kidd, 2002 (see figure 8 of GSA Bulletin Article), and is stop 4b of the 2002 NEIGC guidebook chapter by the same authors. At the macro-scale this site is located where the E-W trending Lemon Fair River Fault intersects the N-S trending Pinnacle Thrust and offsets the N-S trending stratigraphy with an oblique top-to-the south sense (Fig. 1). Hayman and Kidd, 2002 propose that the Lemon Fair Fault is a long-lived structural boundary that existed prior to Taconic thrusting and was active well after Taconic emplacement. At site 5, the Lemon Fair Fault trends NW-SE, sub-parallel to most of the dated structures (see figure 2 below).

The outcrop reveals a pair of inter-related minor fault zones cutting through massively bedded limestone, making exact kinematic interpretations challenging despite four high quality ages. The northern fault zone is a high-angle strike slip fault oriented ~310° containing abundant right-lateral slickenfibres that experienced a phase of deformation at 79 Ma (sample C243A). Although these slickenfibres show a mean trend/plunge of 300°/40°, nearly parallel to the strike of the Lemon Fair Fault, the lack of clear conjugate structures precludes a reliable stress inference.

The southern fault zone contains an upper fault plane oriented at 285°/34° and a lower, possibly conjugate, plane oriented at 328°/54° (see overview photos). The lower plane shows very well developed down-dip slickenfibres recording NE-SW directed extension at ~85 Ma (C127C). A minor slip

surface nearby contains slickenfibres recording NE-SW extension at ~ 80 Ma (C127D-1). NE-SW oriented extension recorded in these minor faults is perpendicular to the orientation of the nearby Lemon Fair Fault (Haymann and Kidd, 2002), suggesting that slip along the larger fault may have influenced the local stress regime and then orientation of slip on these minor surfaces (C127C and C127D-1) nearby.

In addition to the two minor faults described above, the site exhibits a pervasive vertical fracture set with a mean strike/dip of 320°/83° (Fig. 2). These surfaces are routinely coated in a 1-2 mm thick sparry calcite vein, which is interpreted as a mode 1 fracture based on the lack of slickenfibres or tiling, and has been dated to ~97 Ma (C441A). These veins intersect a second set of sub-vertical fractures with a mean strike/dip of 233°/84°, which lack calcite veins (Fig. 2). The dense spacing and near perpendicular nature of these fracture sets suggests they may be conjugates formed at an unknown time. However, the fact that only the NW-SE set is mineralized leads to the interpretation that it acted as a mode-1 dilational fracture under a NE-SW $\sigma 3$ stress at ~97 Ma.

The consistent mineralogical texture and single-domain isochrons suggest all samples at this site date single episodes of primary calcite crystallization.

Field Observations:

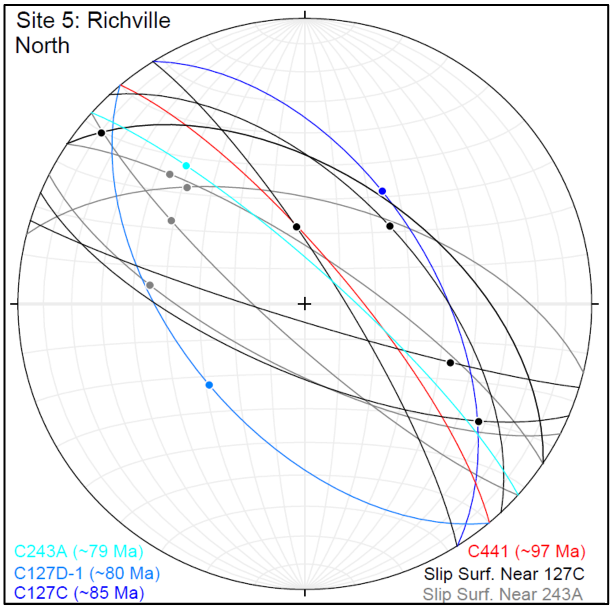

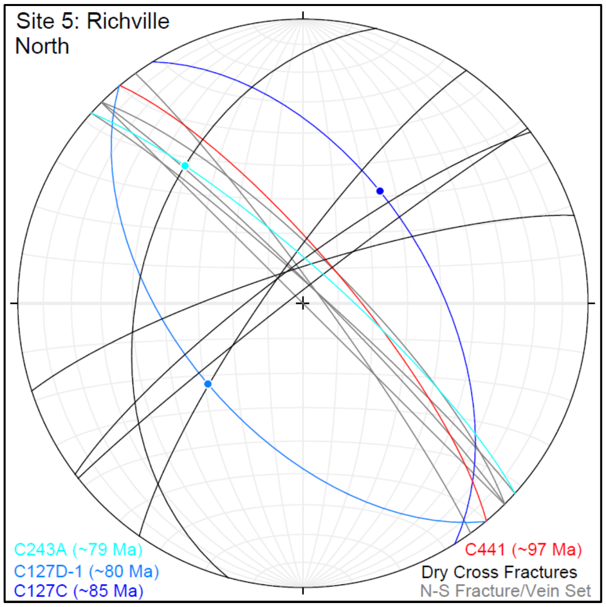

Figure 2: Stereonets showing structural data from site 5. **Left figure** shows all surfaces with measured slickenfibres or striae. Grey are surfaces associated with the near-vertical strike slip fault near sample C243A, black are surfaces associated with the fault zone near sample C127C. Unfortunately, due to the range of orientations and ages it was difficult to identify any definitive conjugate surfaces. **Right figure** shows the dated slip surfaces along with two major fracture sets at the site that lack slip indicators. The N-S oriented set is consistently plated with a 1-2 mm thick sparry calcite vein, the same one sampled as C441.

Overview Photos:

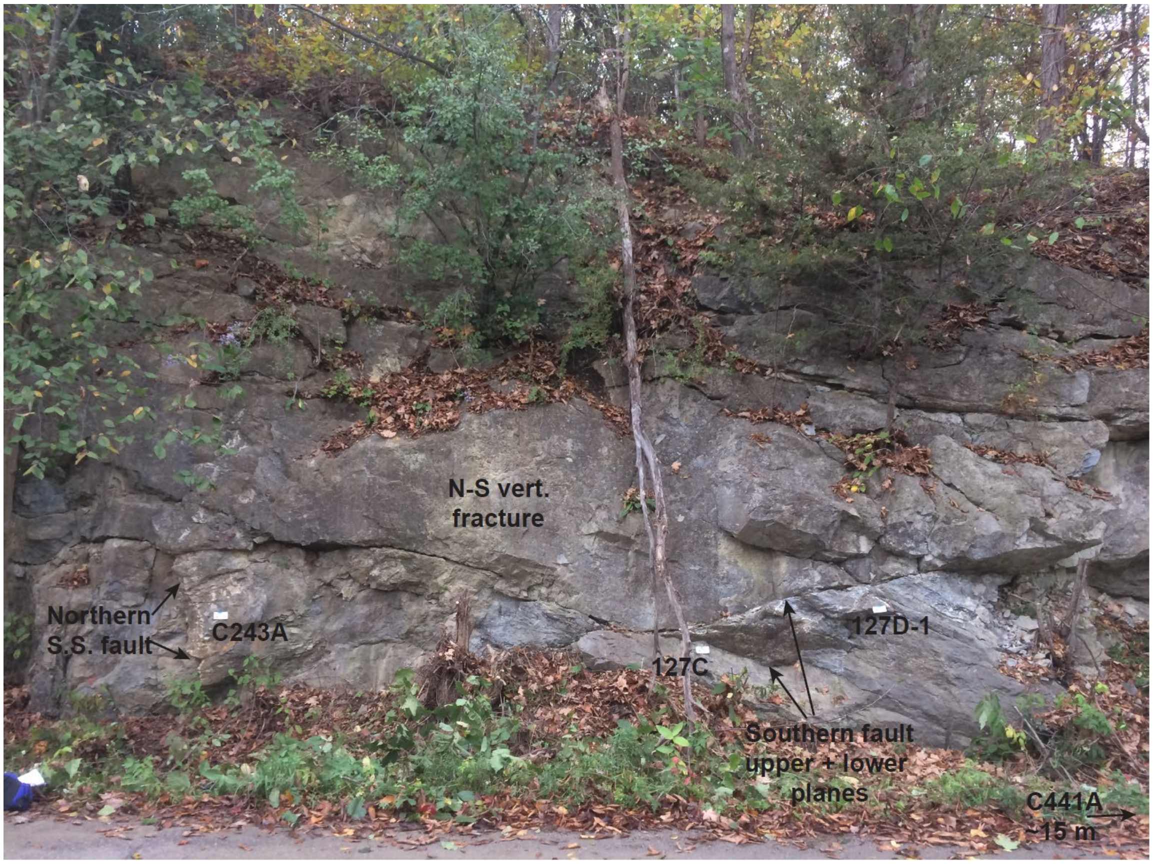

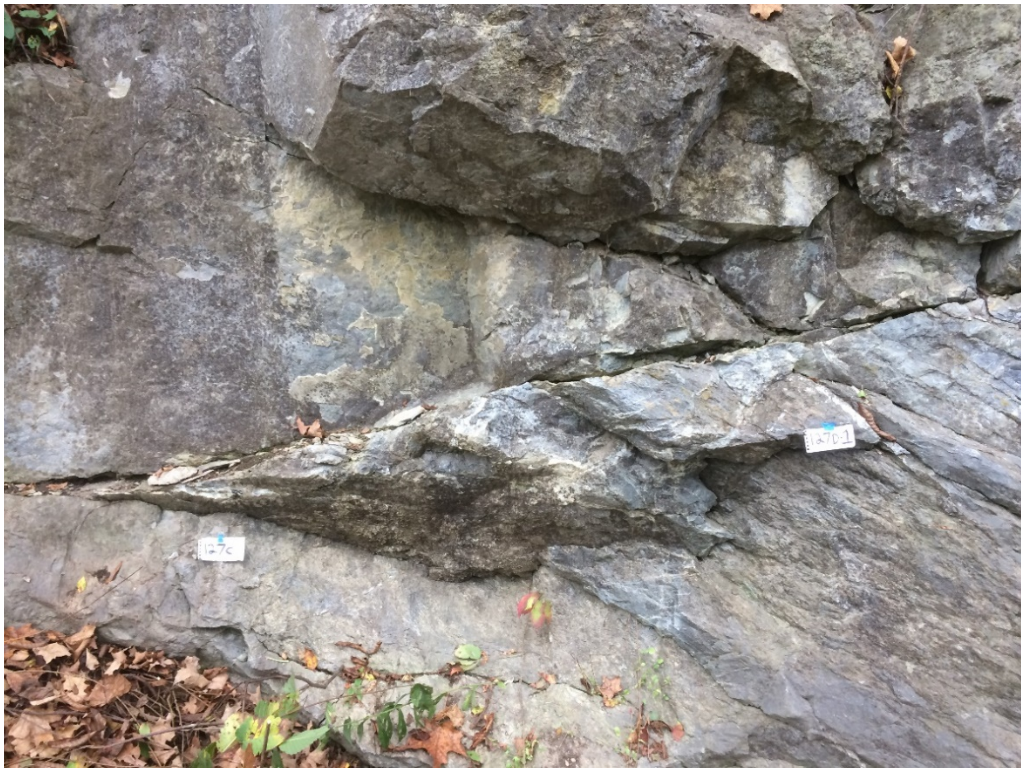

C127C:

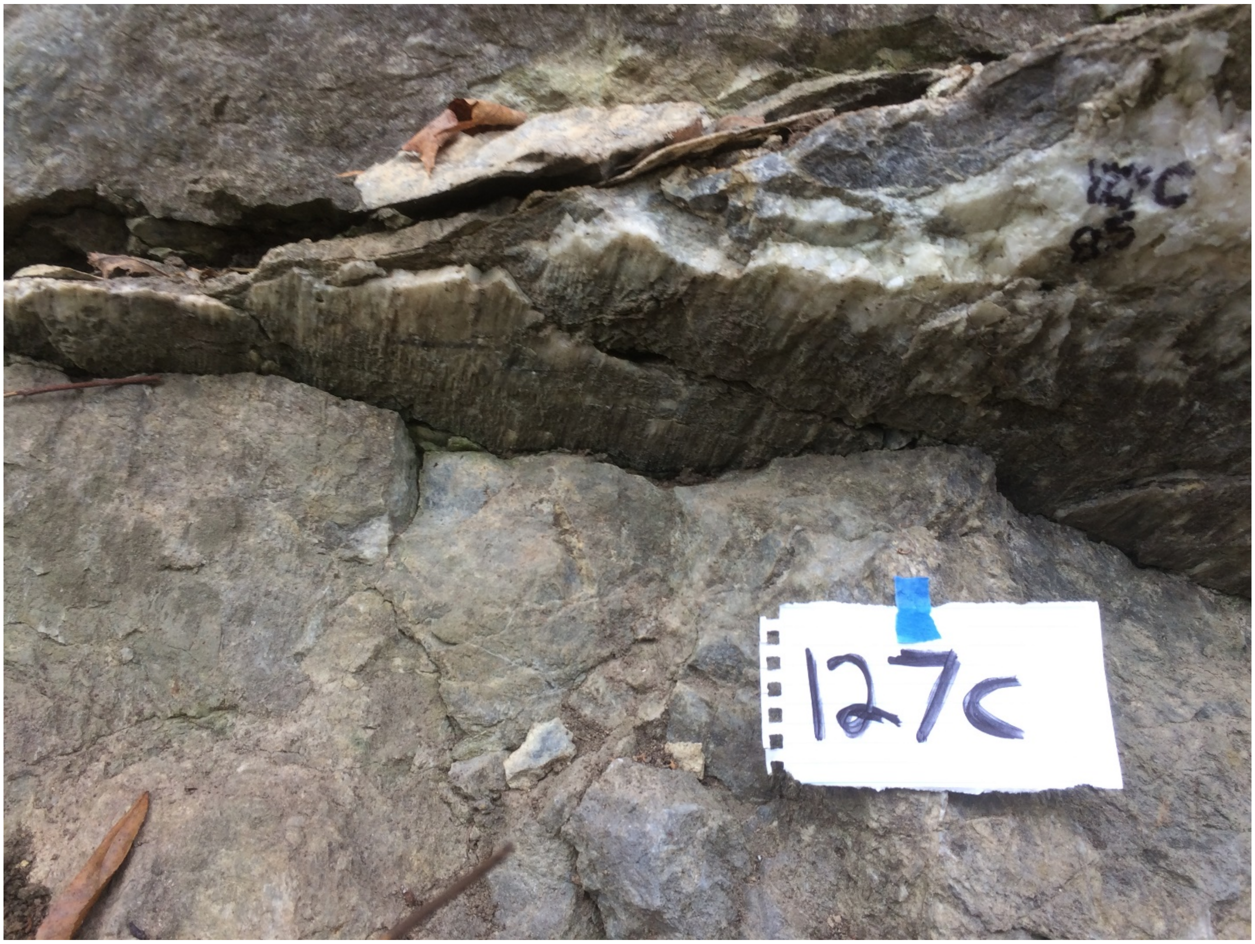

C127D-1:

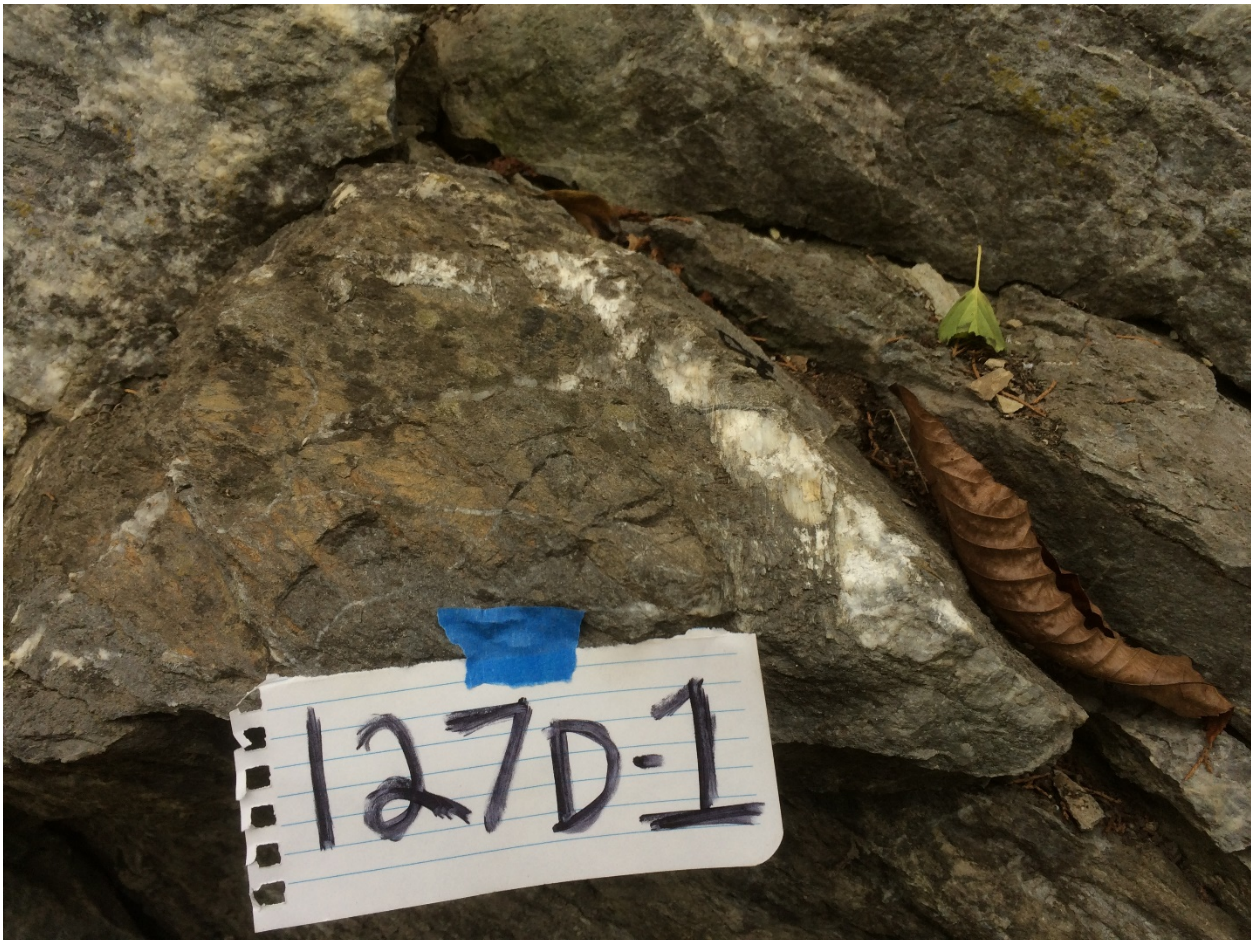

C243A:

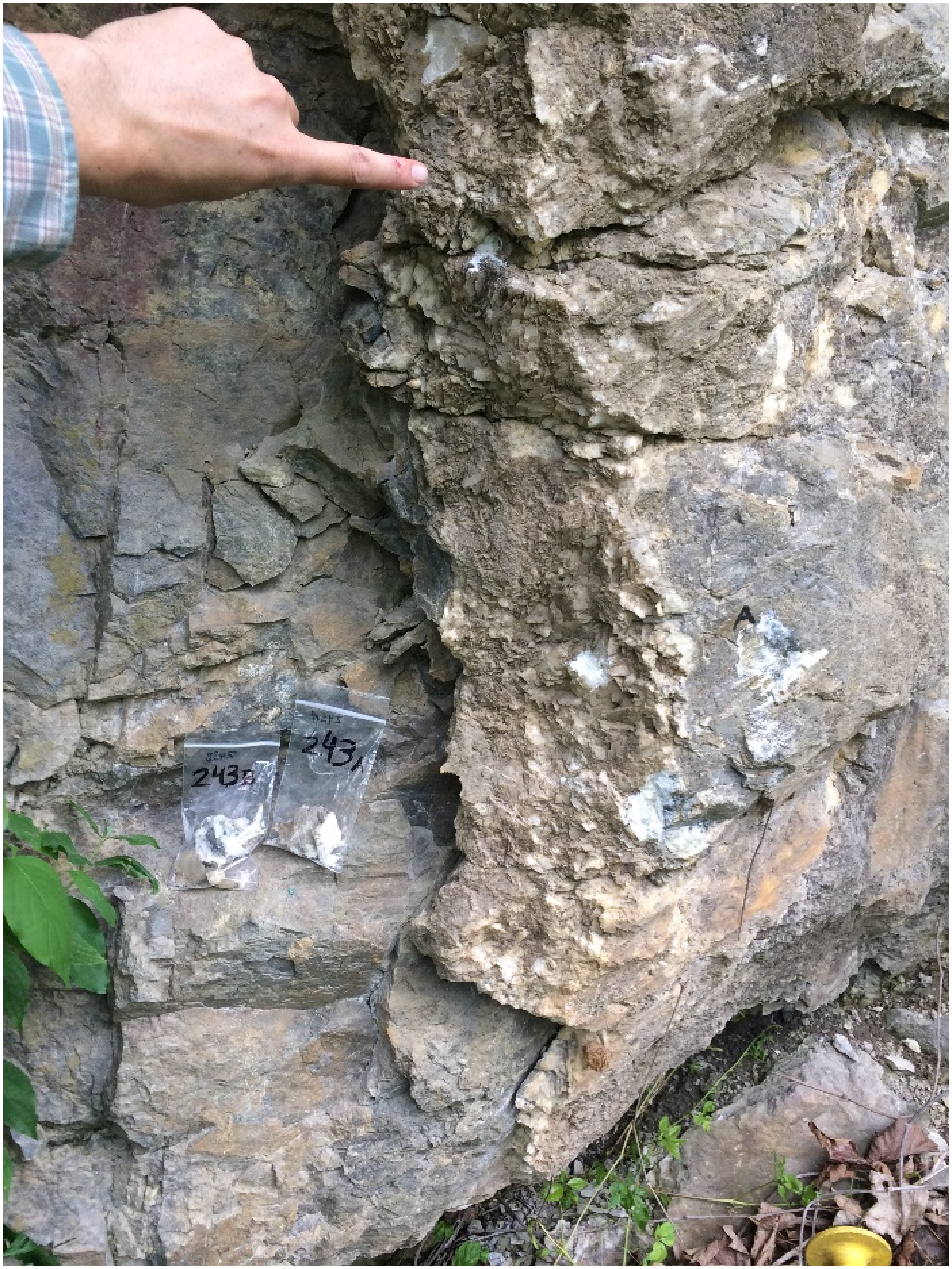

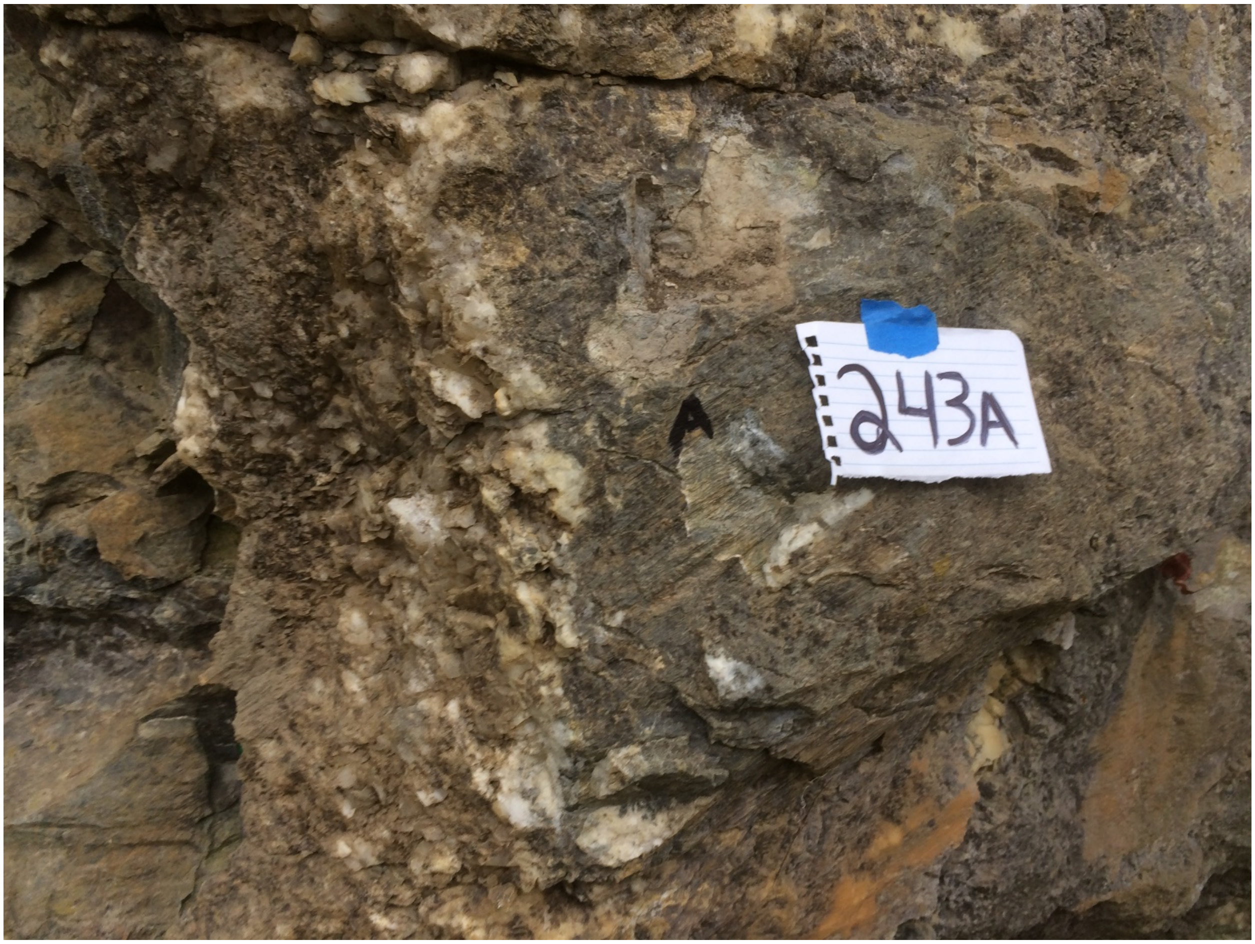
A
243A

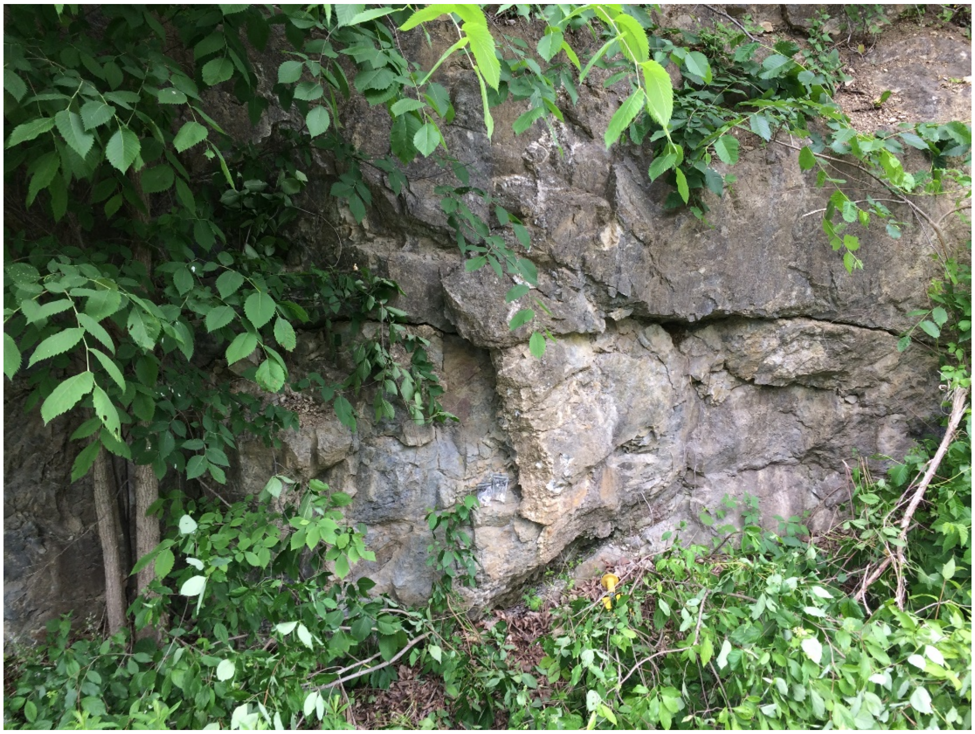

C441A:

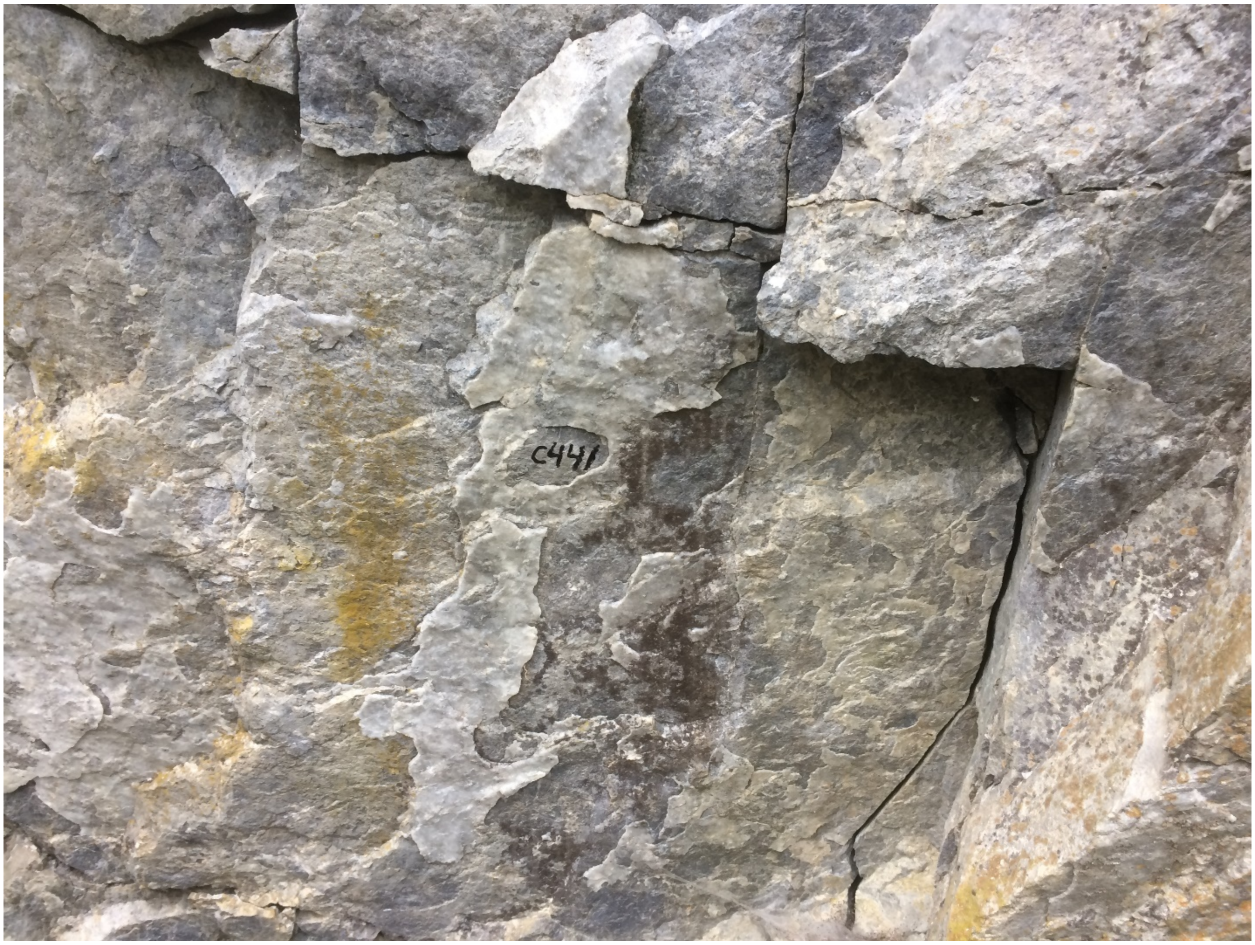

Lab Description: Circles denote areas of interest for dating, not laser shots.

C127C

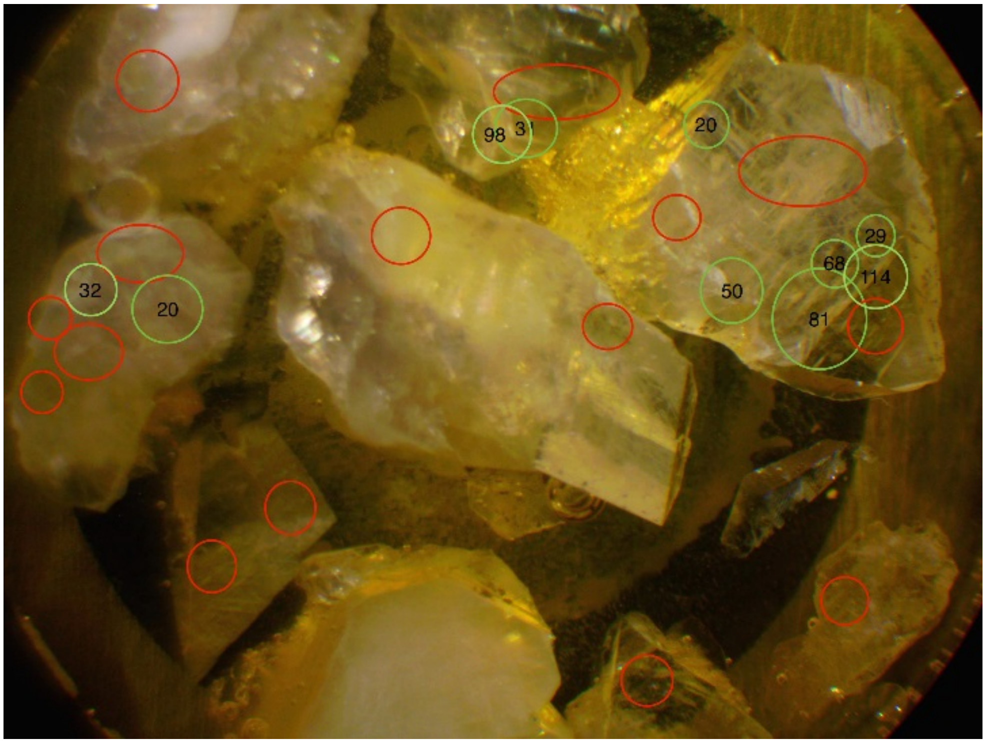

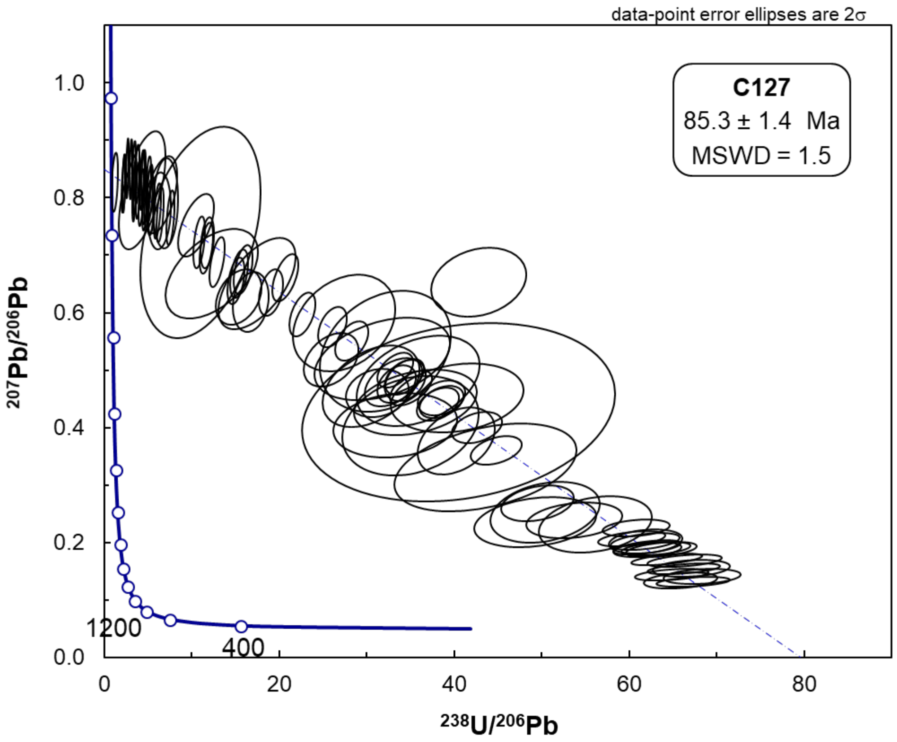

C127D1: Circles denote areas of interest for dating, not laser shots.

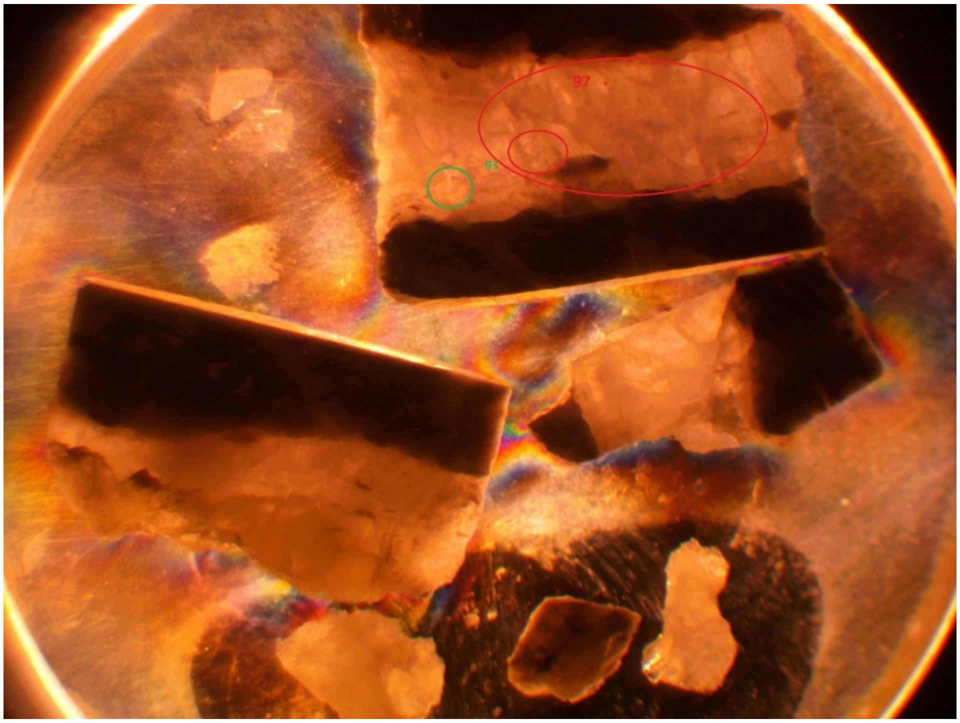

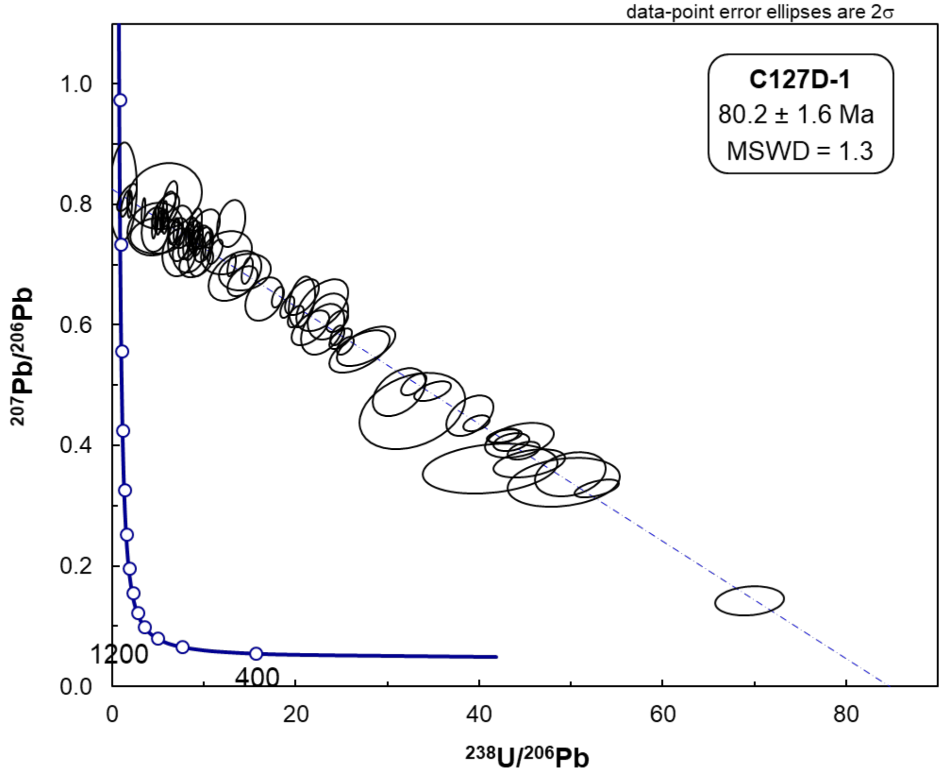

C243A:

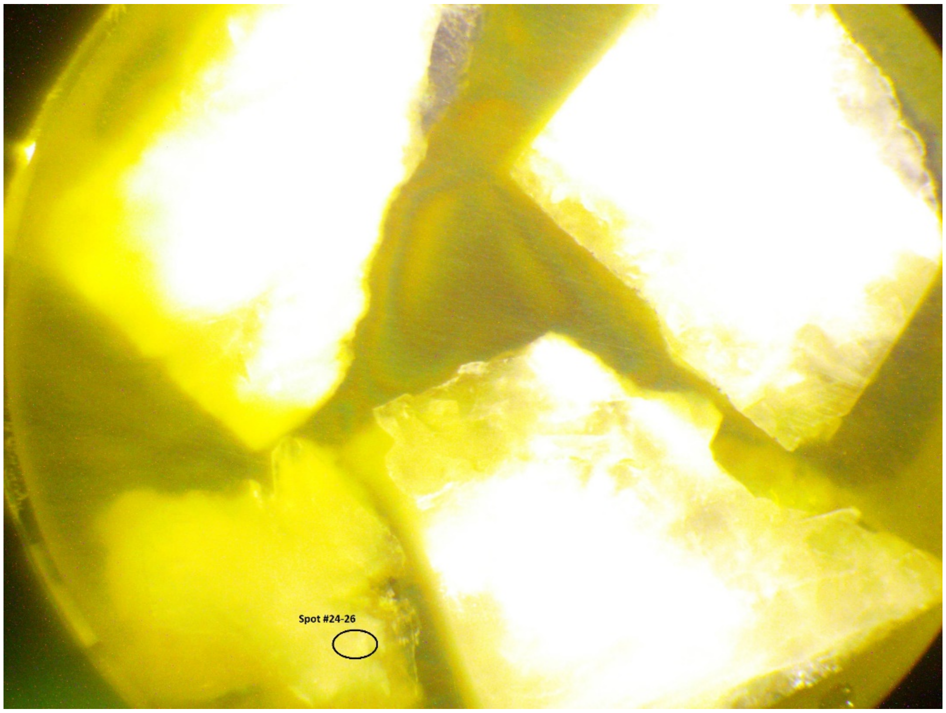

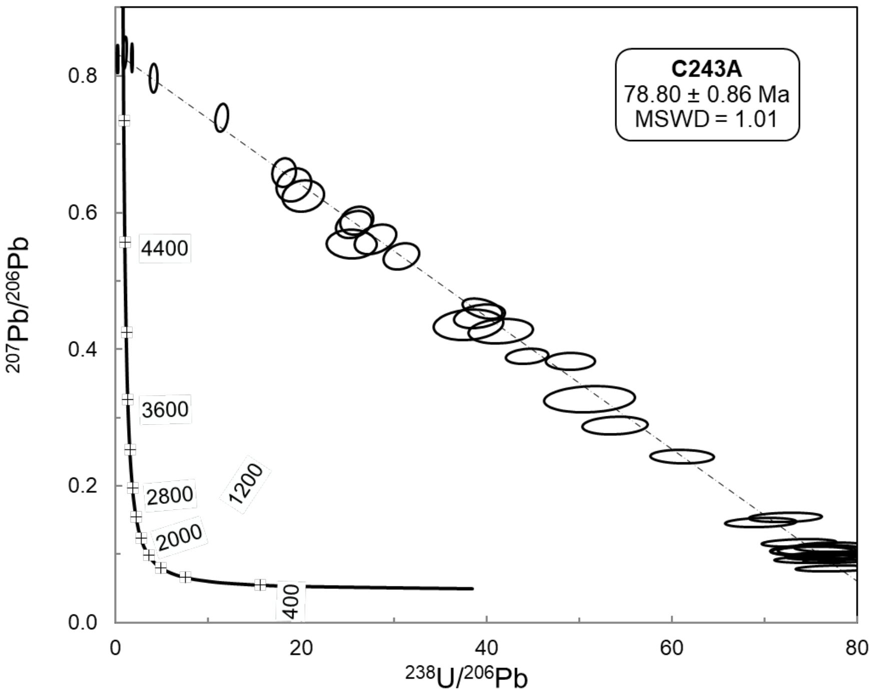
C243A
78.80 ± 0.86 Ma
MSWD = 1.01
$^{207}Pb/^{206}Pb$
$^{238}U/^{206}Pb$
0.0
0.2
0.4
0.6
0.8
0
20
40
60
80
4400
3600
2800
2000
1200
400

C441A

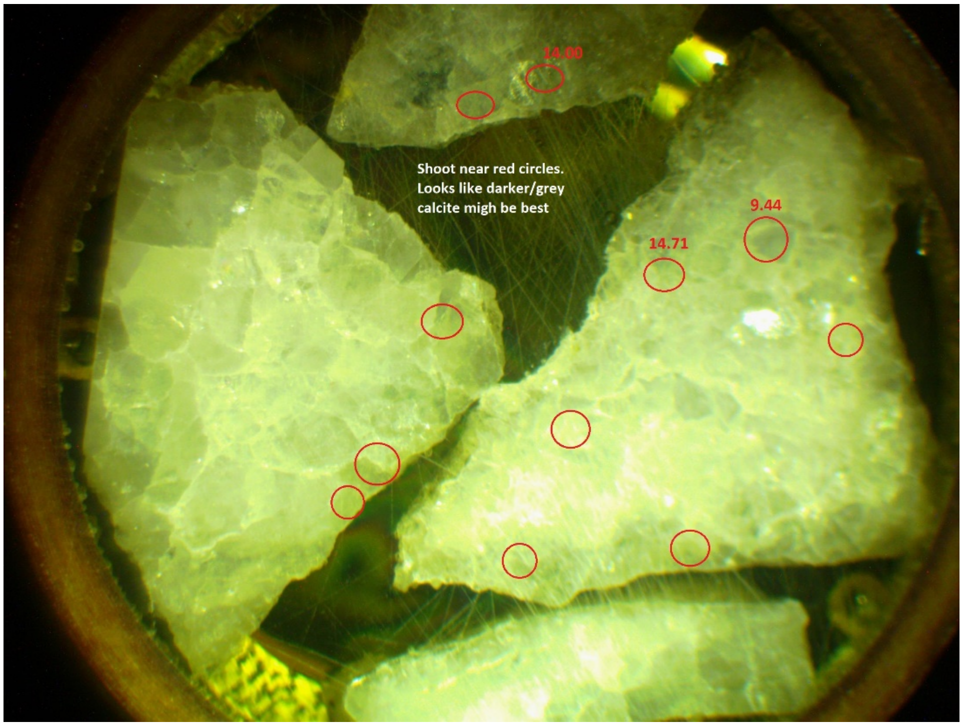
14.00
Shoot near red circles.
Looks like darker/grey
calcite migh be best
9.44
14.71

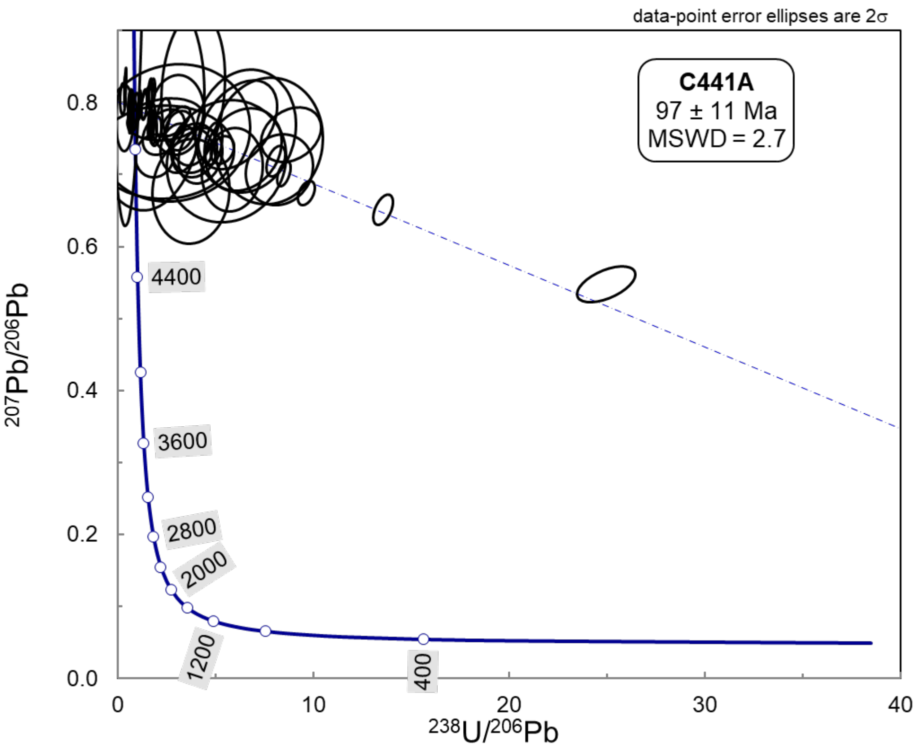

data-point error ellipses are 2σ
C441A
97 ± 11 Ma
MSWD = 2.7
4400
3600
2800
2000
1200
400
0.8
0.6
0.4
0.2
0.0
0
10
20
30
40
207Pb/206Pb
238U/206Pb
