# Supplementary material for: Pace of passive margin tectonism revealed by U-Pb dating of fracture-filling calcite
Source: Nat Commun. 2022 Apr 12;13:1953. doi: 10.1038/s41467-022-29680-z (PMC9005700; doi:10.1038/s41467-022-29680-z)

6: Satterly Quarry, Vergennes Site (44.18876, -73.24255)

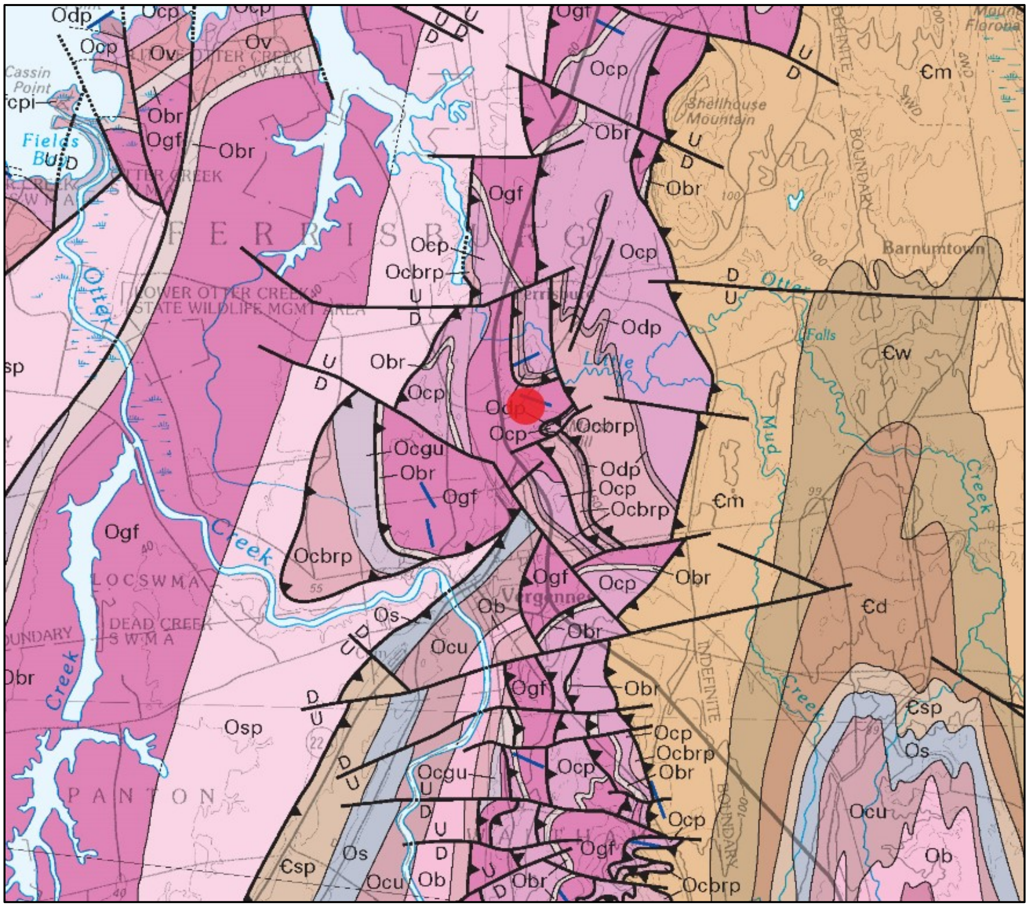

Figure 1: Sample location on state geologic map of Ratcliffe at al. (2011)

<u>Failed samples from same site:</u> C135A,B,F,I,J,K,M,N,O,P,Q,R,U,V,W,X,Y,AA,AB

<u>Bedrock Unit:</u>, Glens Fall Limestone (Ogf)

<u>Ages</u>: **C135C**: 10 ± 6 Ma **C135G**: 2.71 ± 0.88 and 57.3 ± 1.4 Ma **C135H**: 2.90 ± 0.29 Ma

C135L: 4.9 ± 2.9 Ma **C135AE**: 7 ± 30 Ma

<u>Summary</u>: A crushed stone quarry off Satterly Road just east of Vergennes, VT. Access by car with permission of owner David Shlansky (david.shlansky@slglawfirm.com; (802) 877-6872). The weak rocks of the Iberville Shale are extremely deformed with numerous faults and a pervasive high angle cleavage. The cleavage has been reactivated to accommodate both top-to-the-east extension and top-to-the-west compression as evidenced by frequent dip-slip slickensides and minor cleavage-slip offsets (see photos below). The overall structure of the quarry is a N-S oriented thrust placing Glens Falls Limestone over Iberville shale and associated hanging wall folds that have intensely shattered the rock. Although samples from the major fault zone hinted at Cretaceous ages, none were deemed high enough quality for inclusion in this dataset. Instead, all of the publishable ages from this site record a sequence of up to three Mio-Pliocene events.

First, Sample 135C dates to ~10 Ma, and comes from a N-S trending low angle (32°) fracture, which is presumed to be a fault zone although it was destroyed before detailed observations could be made. We thus record the kinematics as 'unknown' for this sample. Second, sample 135L records E-W extension at ~ 5 Ma along a discrete, high angle extensional fault, which cuts lower angle fabrics associated with the primary N-S fault zone (see photos below). This surface has well preserved slickensides that record top to the east displacement, inferred to have formed during E-W extension. Samples C135G and C135H were collected from calcite fabrics formed during extensional slip on reactivated cleavage planes fromed at ~ 3 Ma (see photos below). As shown in the stereonets below, surfaces strike roughly N-S and dip to the east at ~40° and exhibit well-preserved top to the east slickenfibres and downward stepping tiling structures. Finally, sample C135AE was collected from tiled calcite slickenfibres that record top-to-the-west thrusting at 7 ± 30 Ma.

In summary, the shattered and weak rocks of the Satterly Quarry accommodated a variety of Mio-Pliocene tectonic adjustments. In all cases slip appears to have been highly distributed across multiple small structures and cleavage planes, with very small amounts of slip, typically a few cm and always <10 cm. It seems very likely that the existence of a major fault zone and the pervasively fractured nature of this site contributed to its ability to record minor amounts of Mio-Pliocene strain. However, the Iberville and Hortonville shale show ubiquitous cleavage-parallel slip at many outcrops in the Champlain Valley, begging the question of whether these week rocks could have accommodated a large amount of distributed strain.

<u>Field Observations</u>:

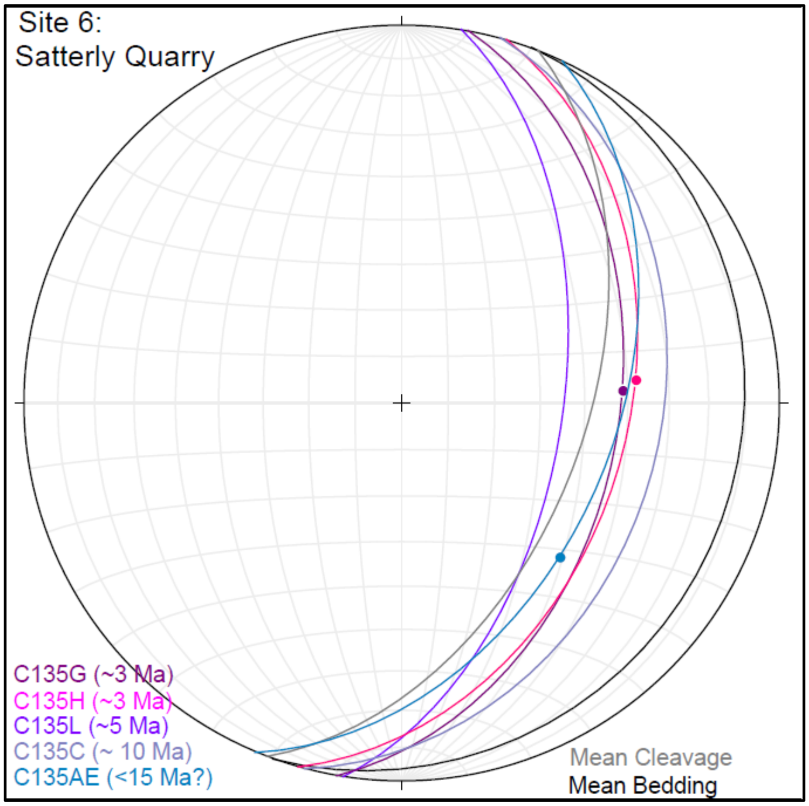

Figure 2: Field measurements of dated structures, mean cleavage, and mean bedding at Satterly Quarry.

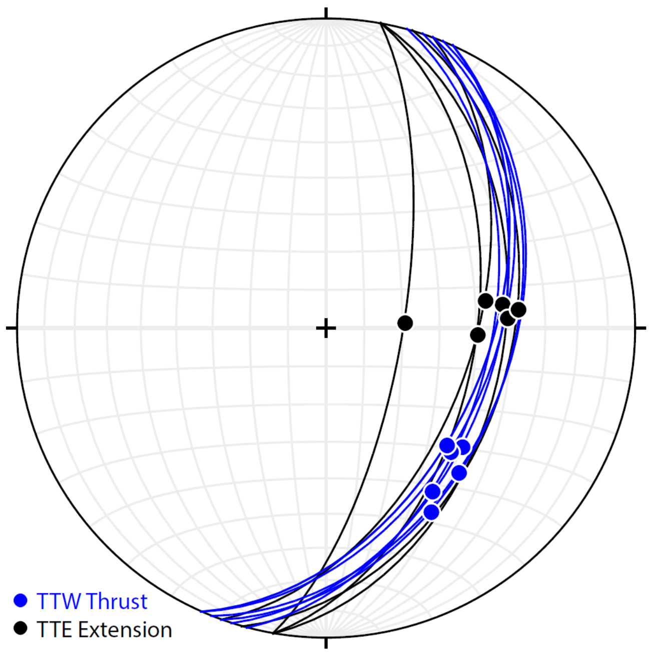

Figure 3: Stereonet plot showing measurements of two sets of cleavage-parallel slickensides along the south and west walls of the Satterly Quarry. Black symbols record top-to-the-east extensional slip, whereas blue symbols record top-to-the-west thrust motion. Photos below show examples of cleavage-parallel slip at Satterly Quarry.

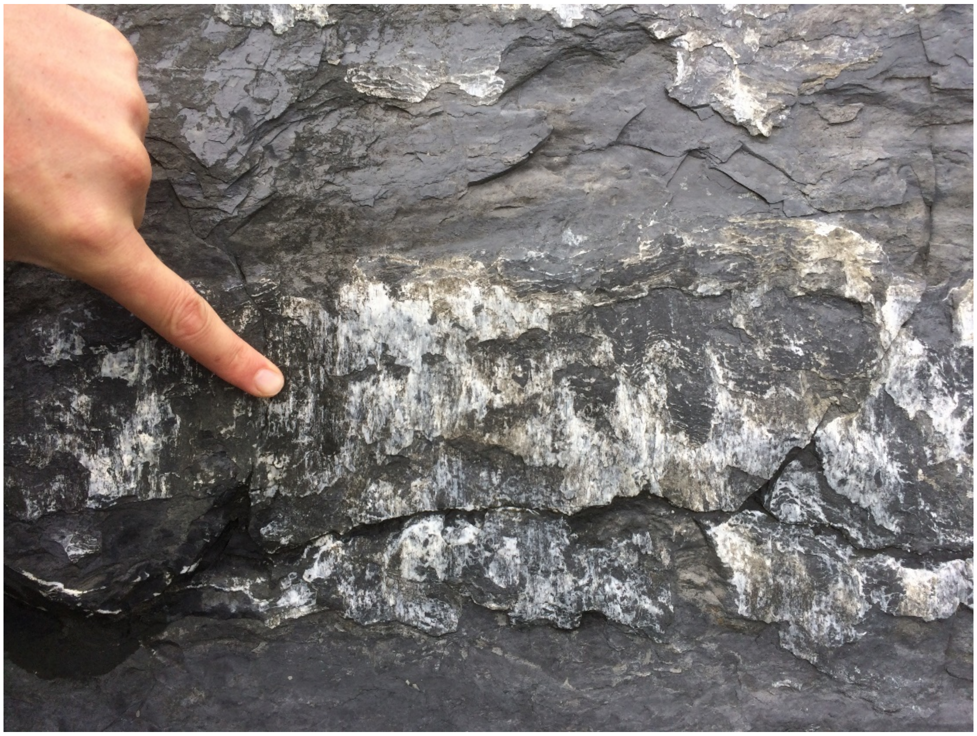

Figure 4: *Example of cleavage-parallel, top-to-the-east extensional slickensides on the west wall of the Satterly Quarry.*

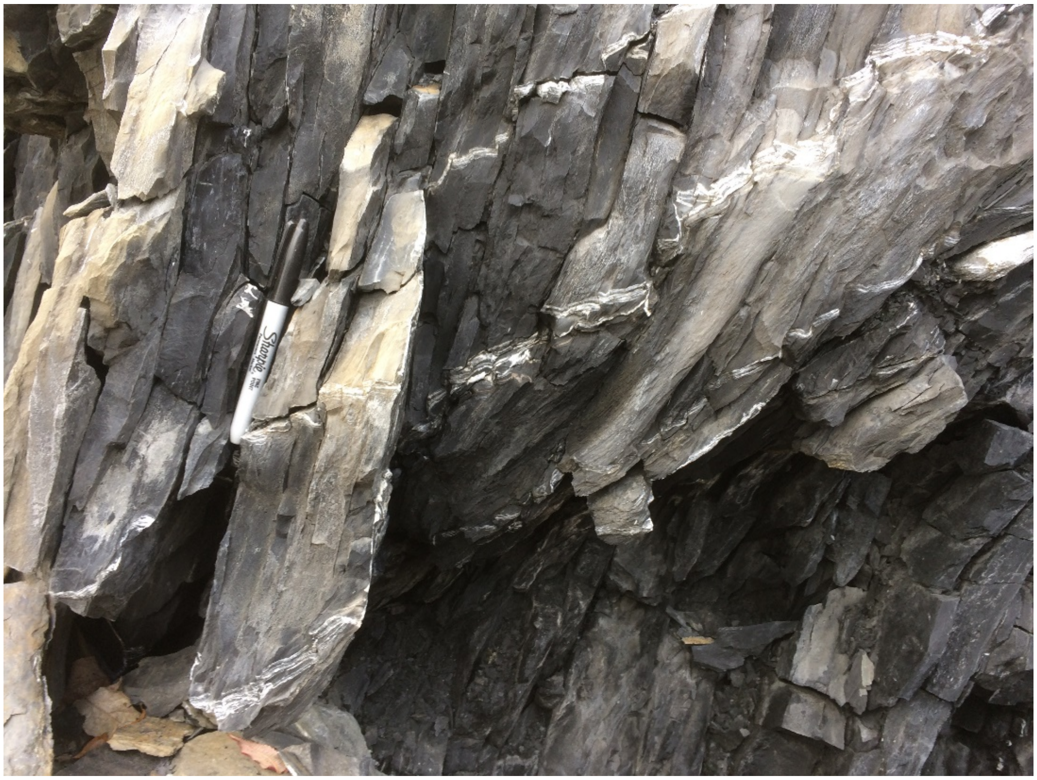

***Figure 5**: Roughly 6" normal-sense offset along a cleavage plane at the Satterly Quarry*

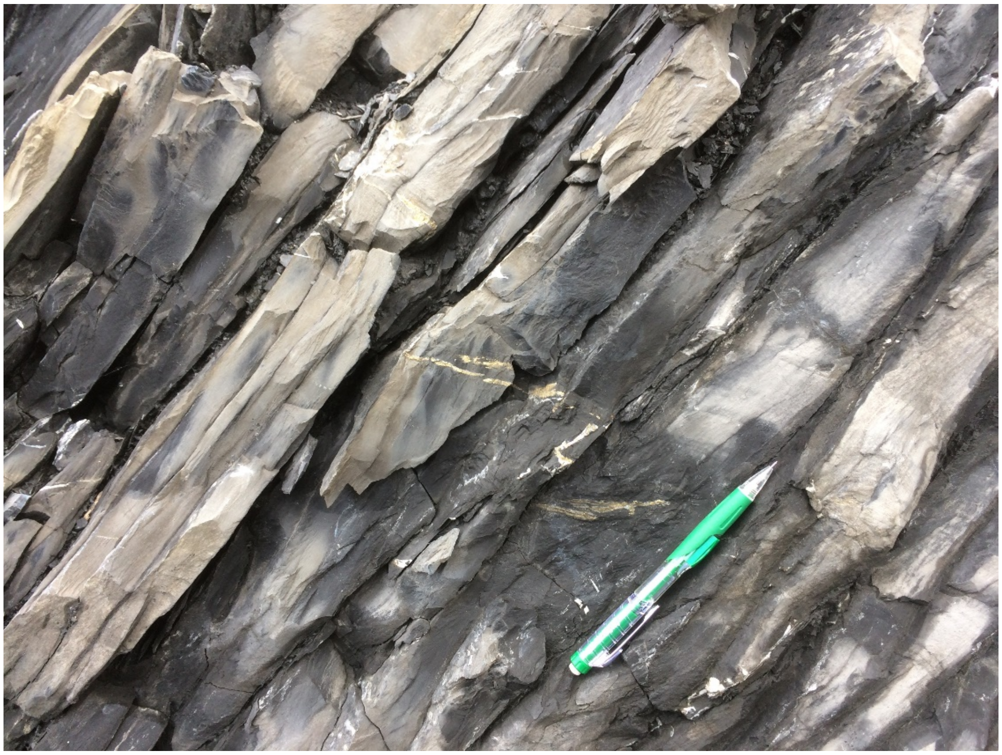

***Figure 6**: Offset of a pyrite-rich sedimentary layer demonstrates cleavage-parallel, thrust sense offset*

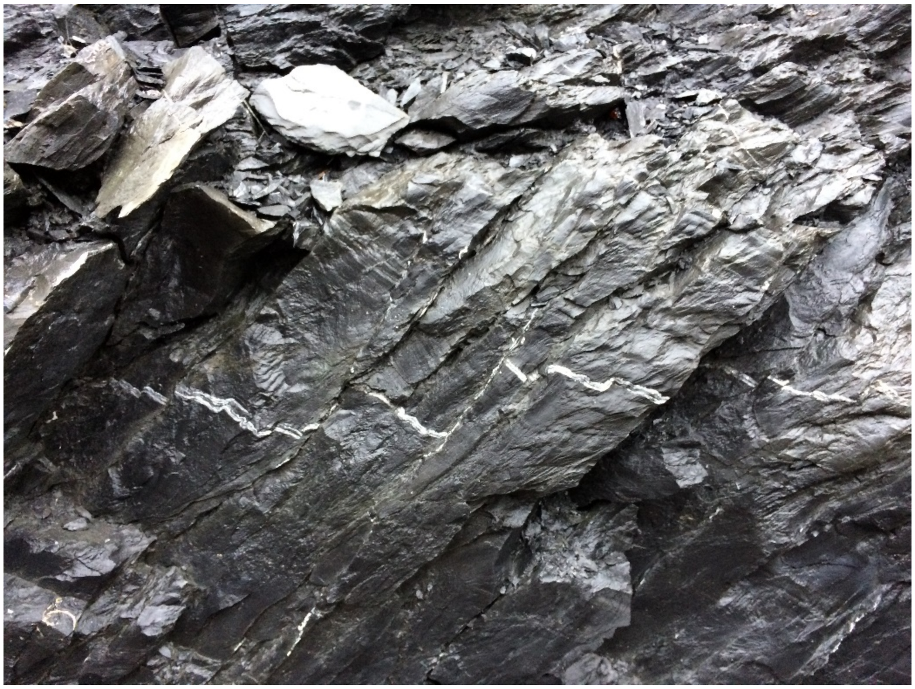

Figure 7: *Normal-sense offset of a bedding-parallel calcite vein at Satterly quarry*

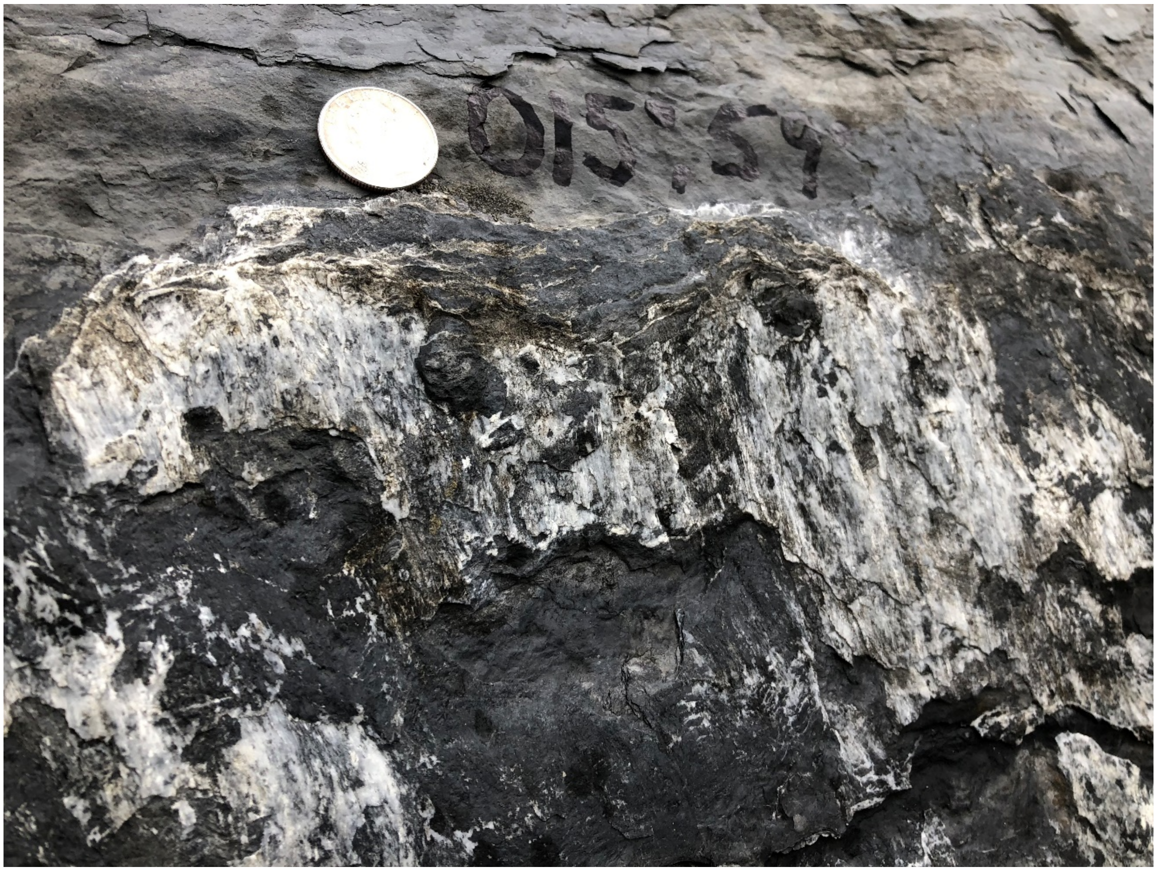

Figure 8: *Normal-sense offset of a bedding-parallel calcite vein at Satterly quarry*

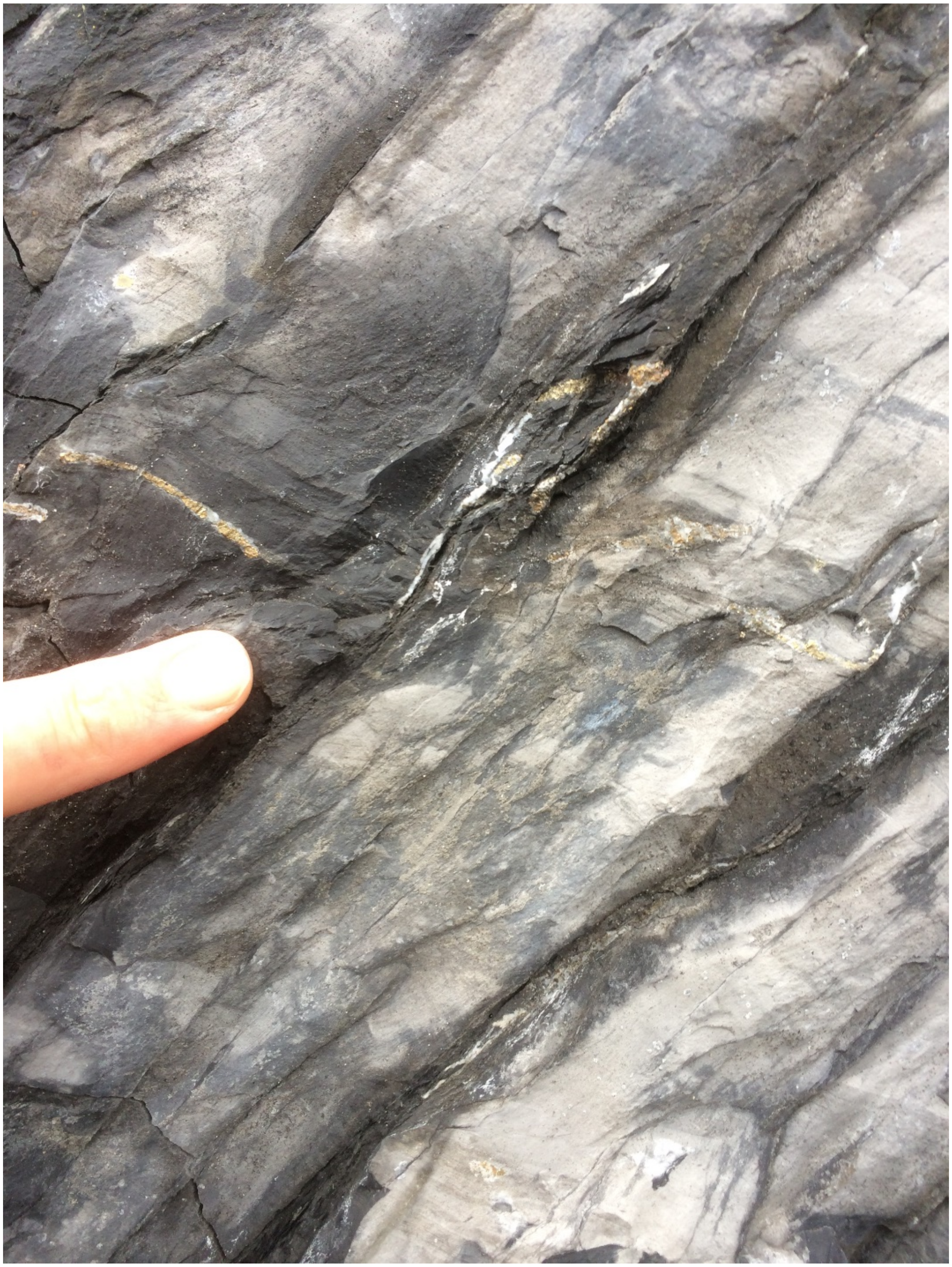

***Figure 9**: Example of a pyrite-rich sedimentary layer that was first offset in a thrust sense and then offset in a normal sense to its present state*

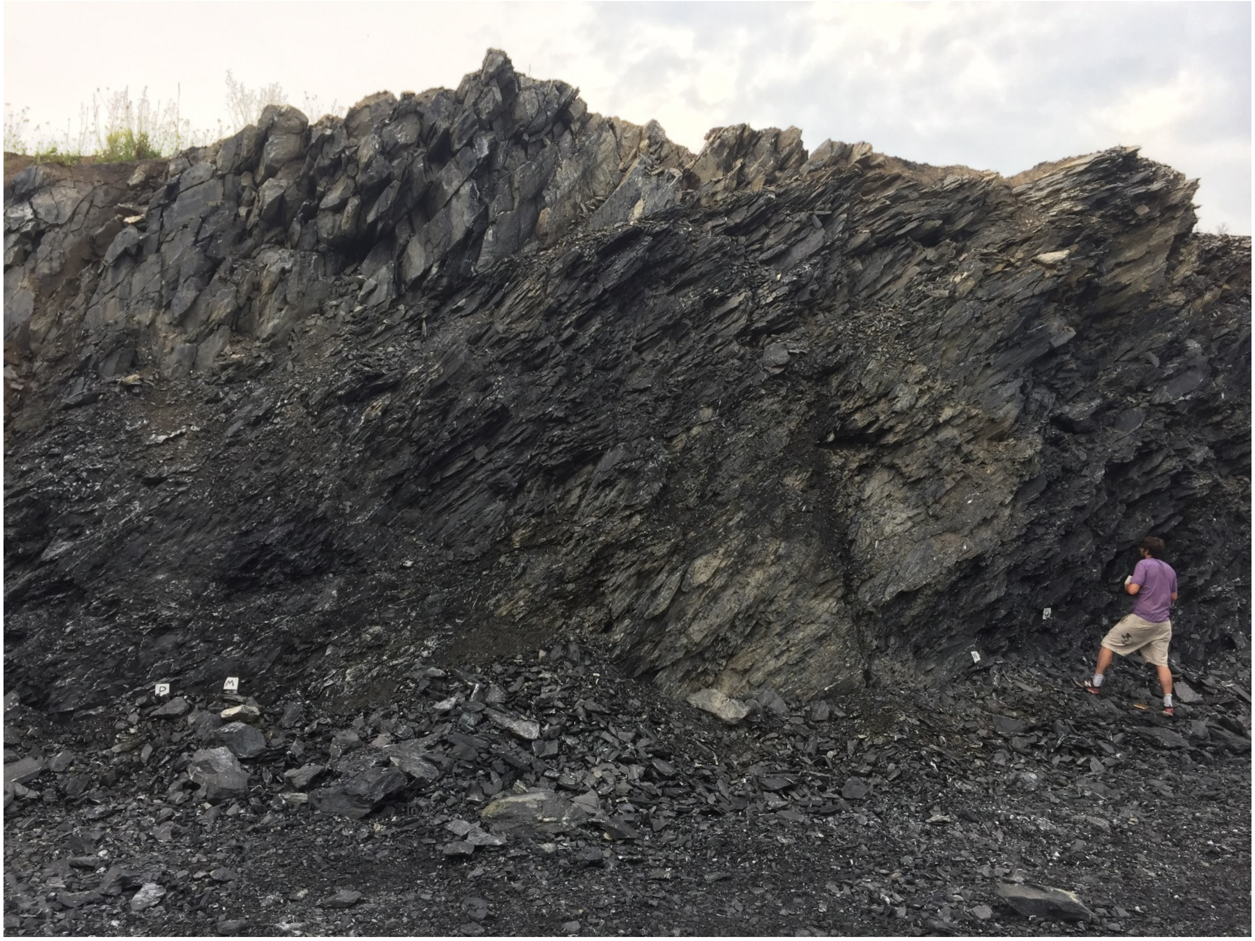

***Figure 10**: Photograph of the main thrust zone with Glens Falls Limestone (top left) thrust over Iberville Shale (lower right).*

Regional Lineament Analysis:

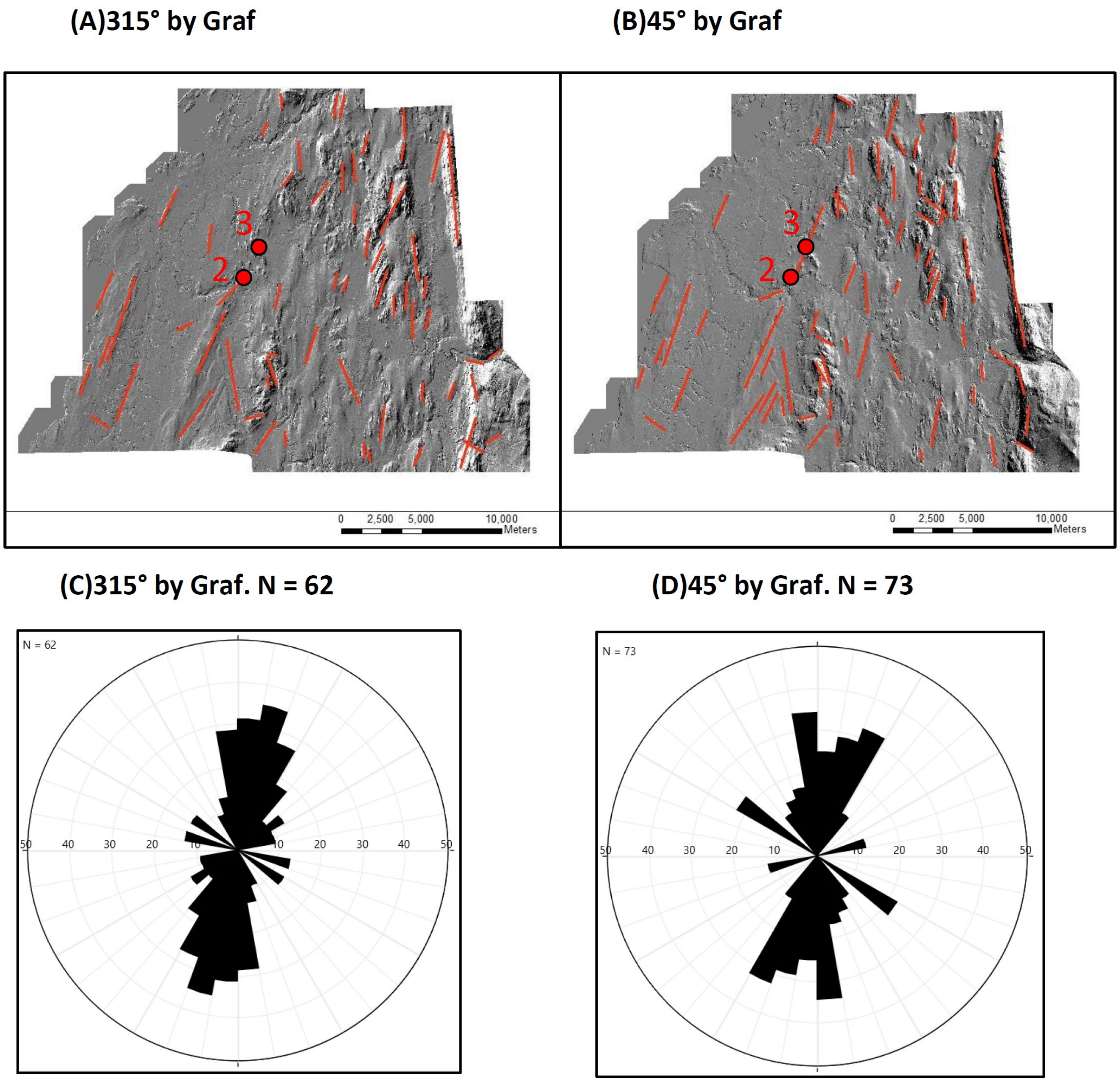

Figure 11: Lineaments mapped by student Sam Graf around Satterly Quarry (labeled site 3) using hillshade images with azimuth angles 315° (left) and 45° (right). Study sites are shown by red dots. Rose diagrams depict a general north-northeast to south-southwest trend of plotted lineaments.

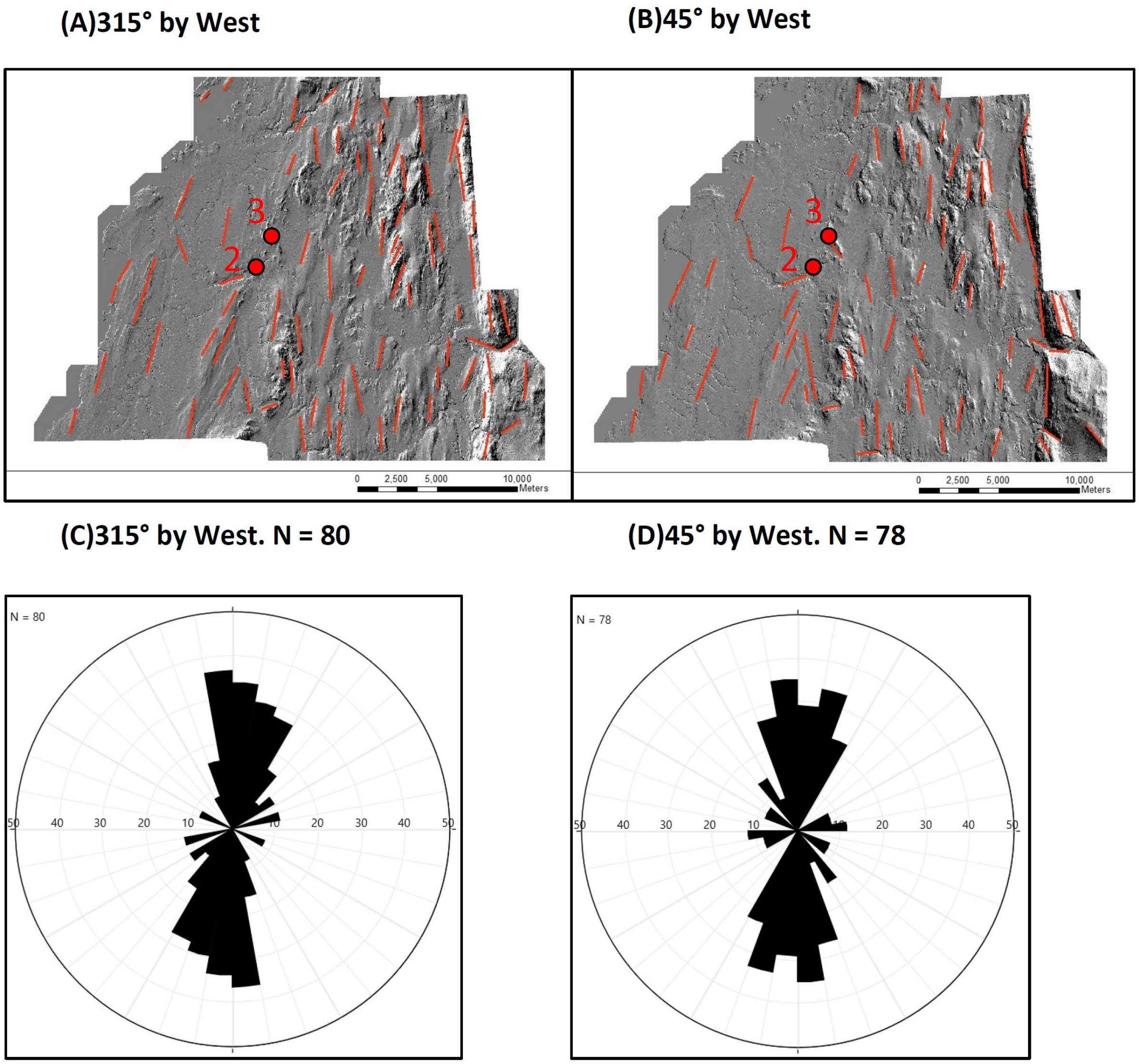

Figure 12: Lineaments mapped by Professor Dave West around Satterly Quarry (labeled site 3) using hillshade images with azimuth angles 315° (left) and 45° (right). Study sites are shown by red dots. Rose diagrams depict a general north-northeast to south-southwest trend of plotted lineaments.

C135C:

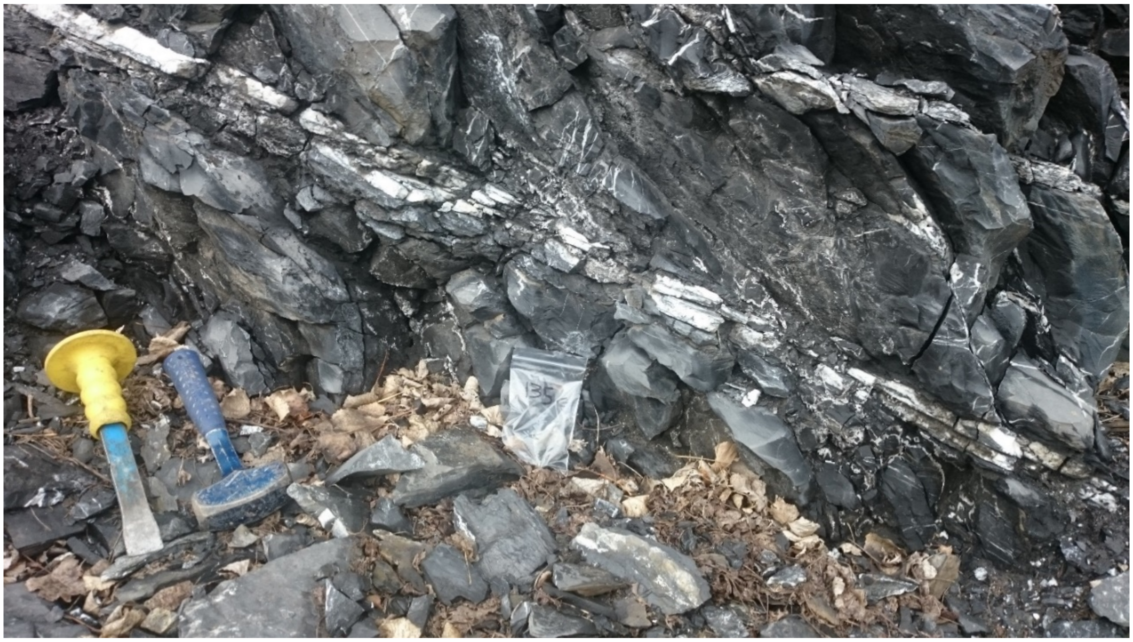

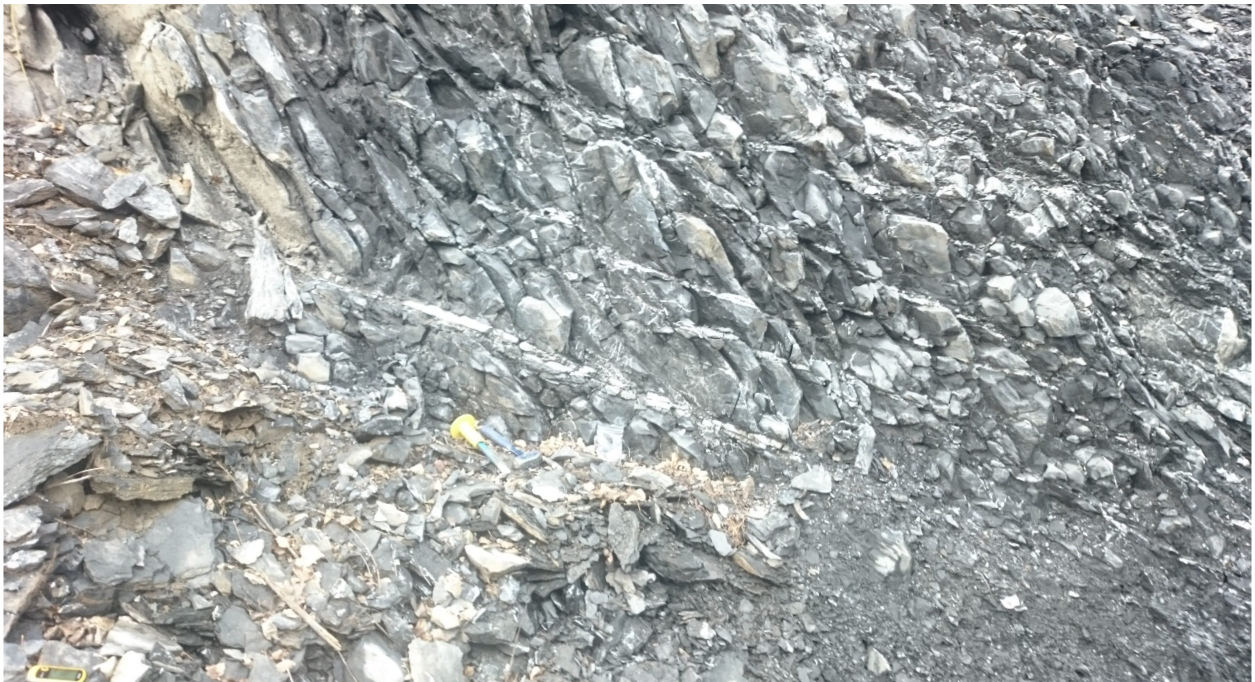

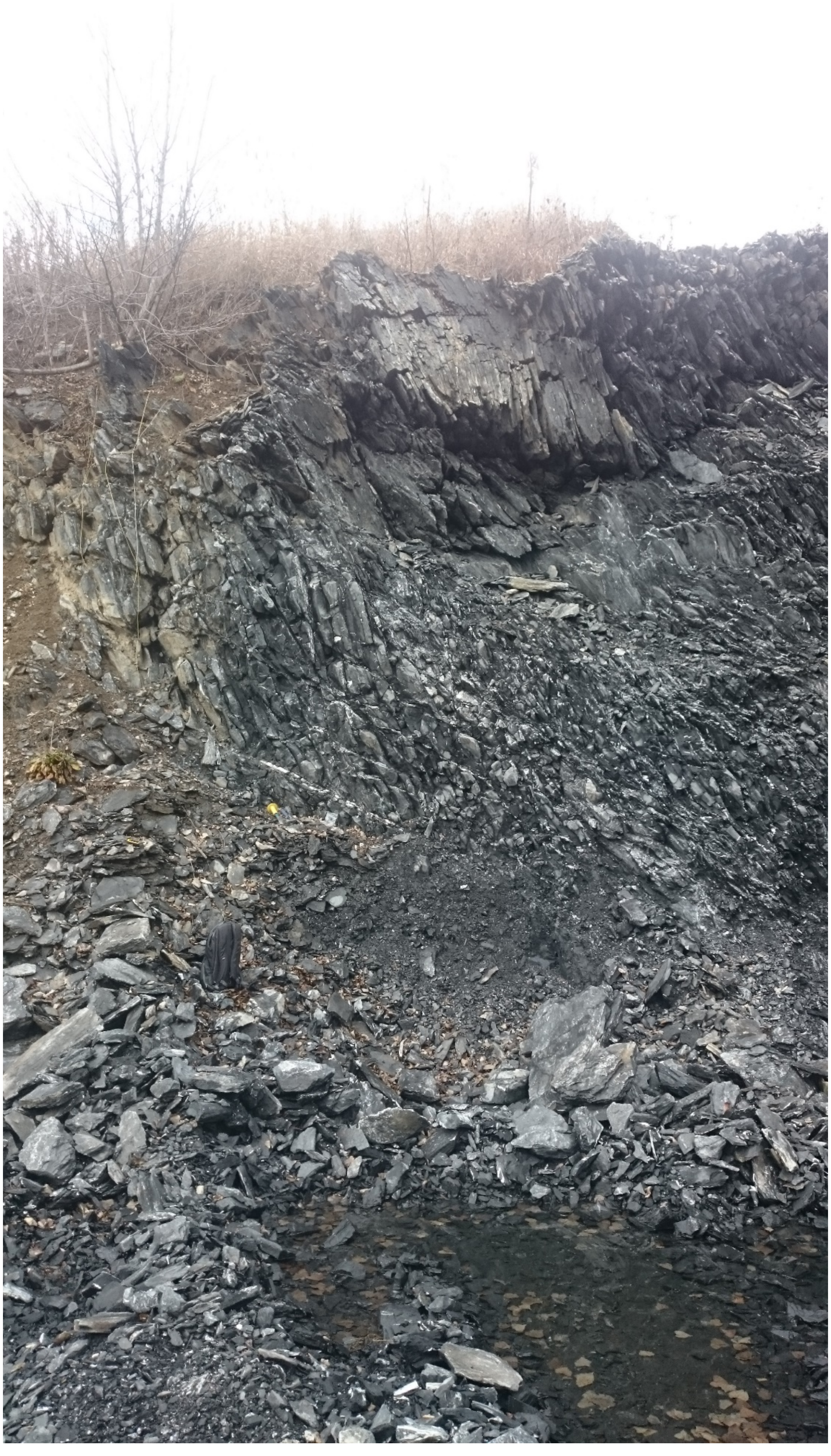

C135G: No photo available

C135H: No photo available

C135L:

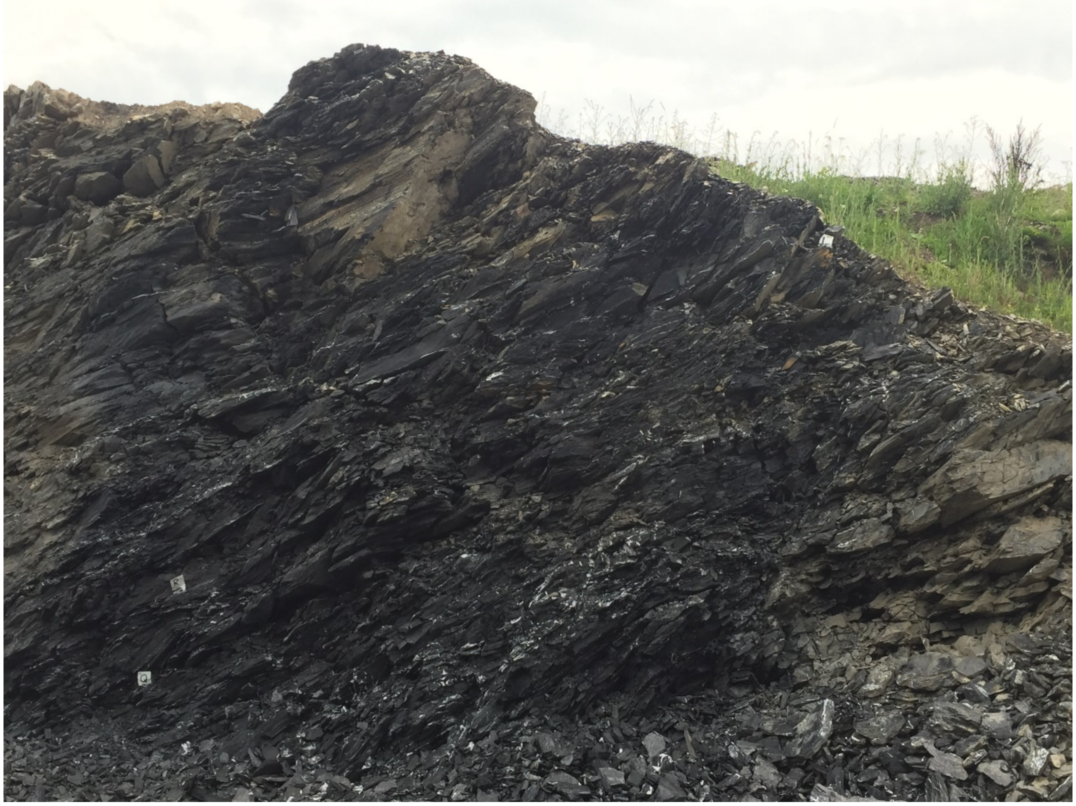

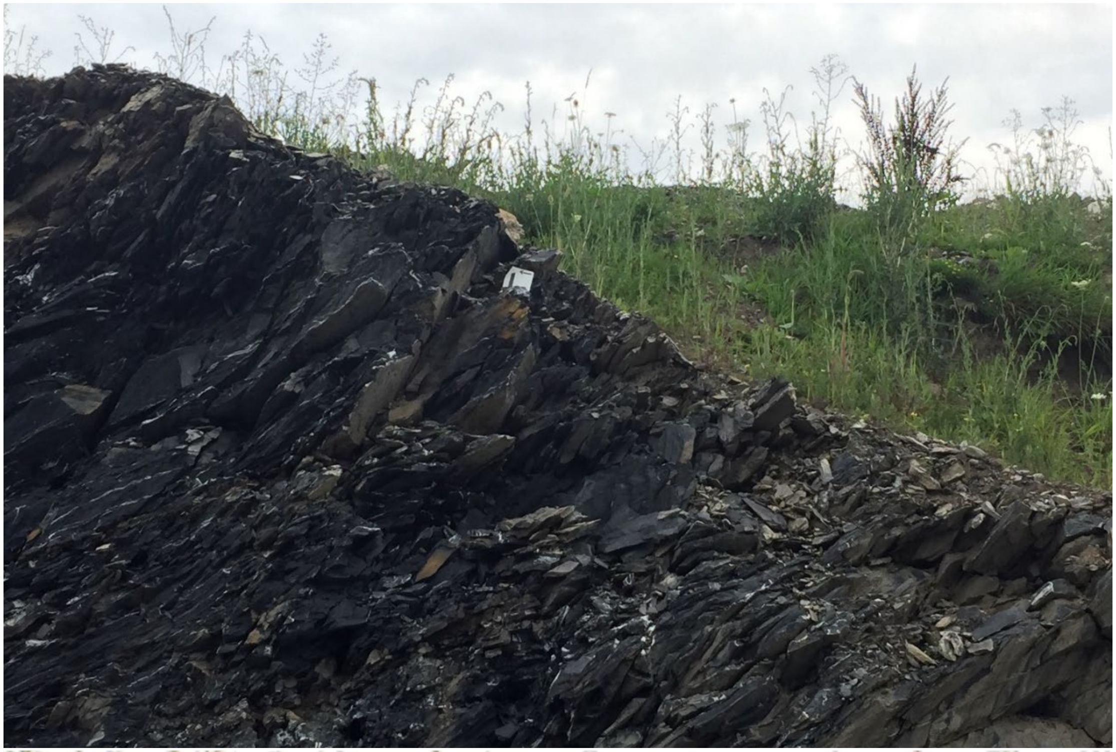
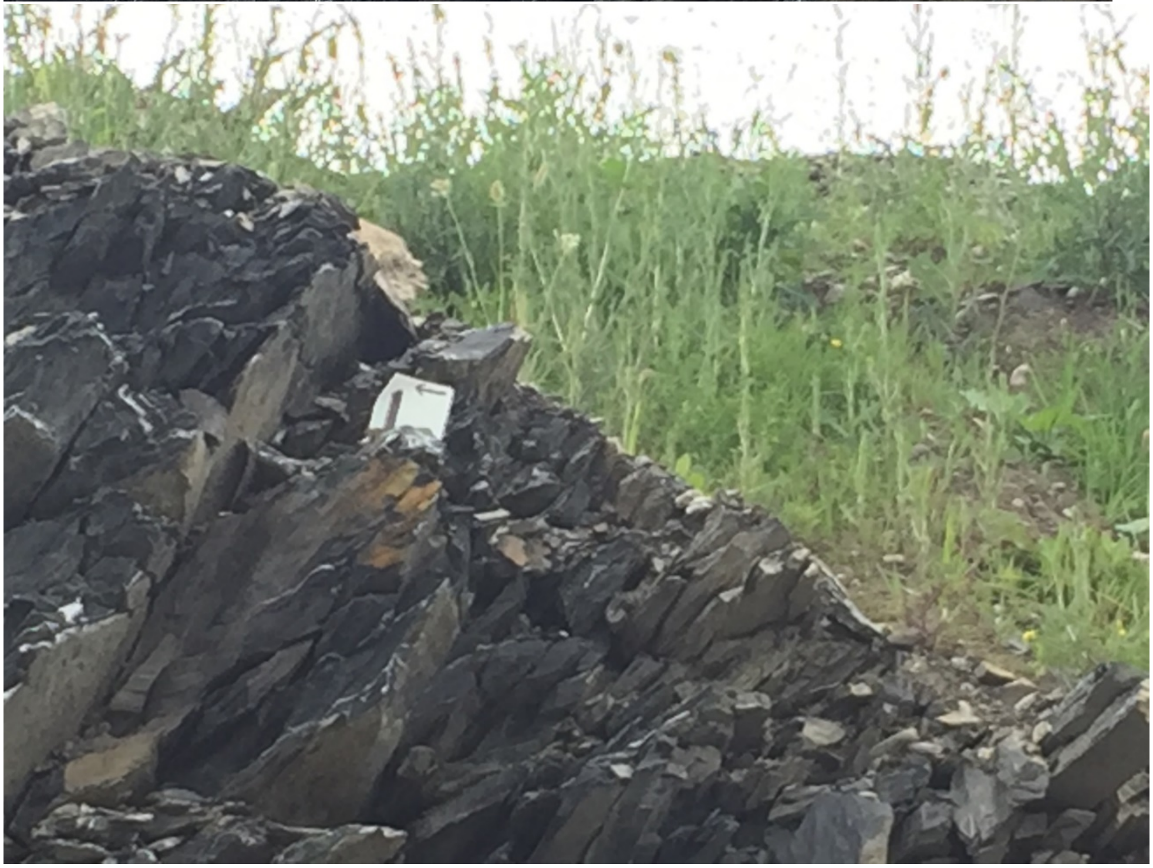

135AE (originally labeled ‘Z’ in field’):

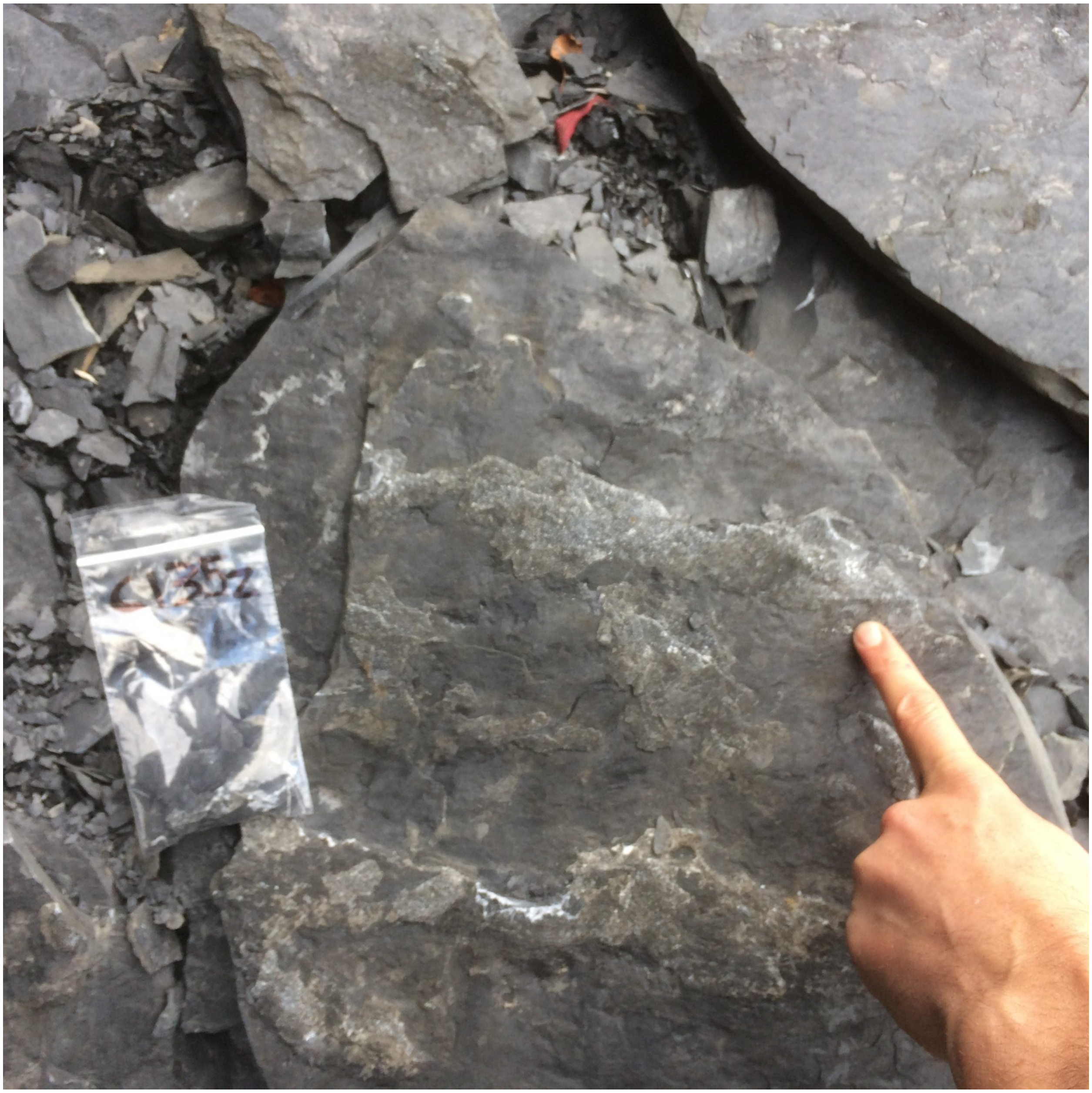

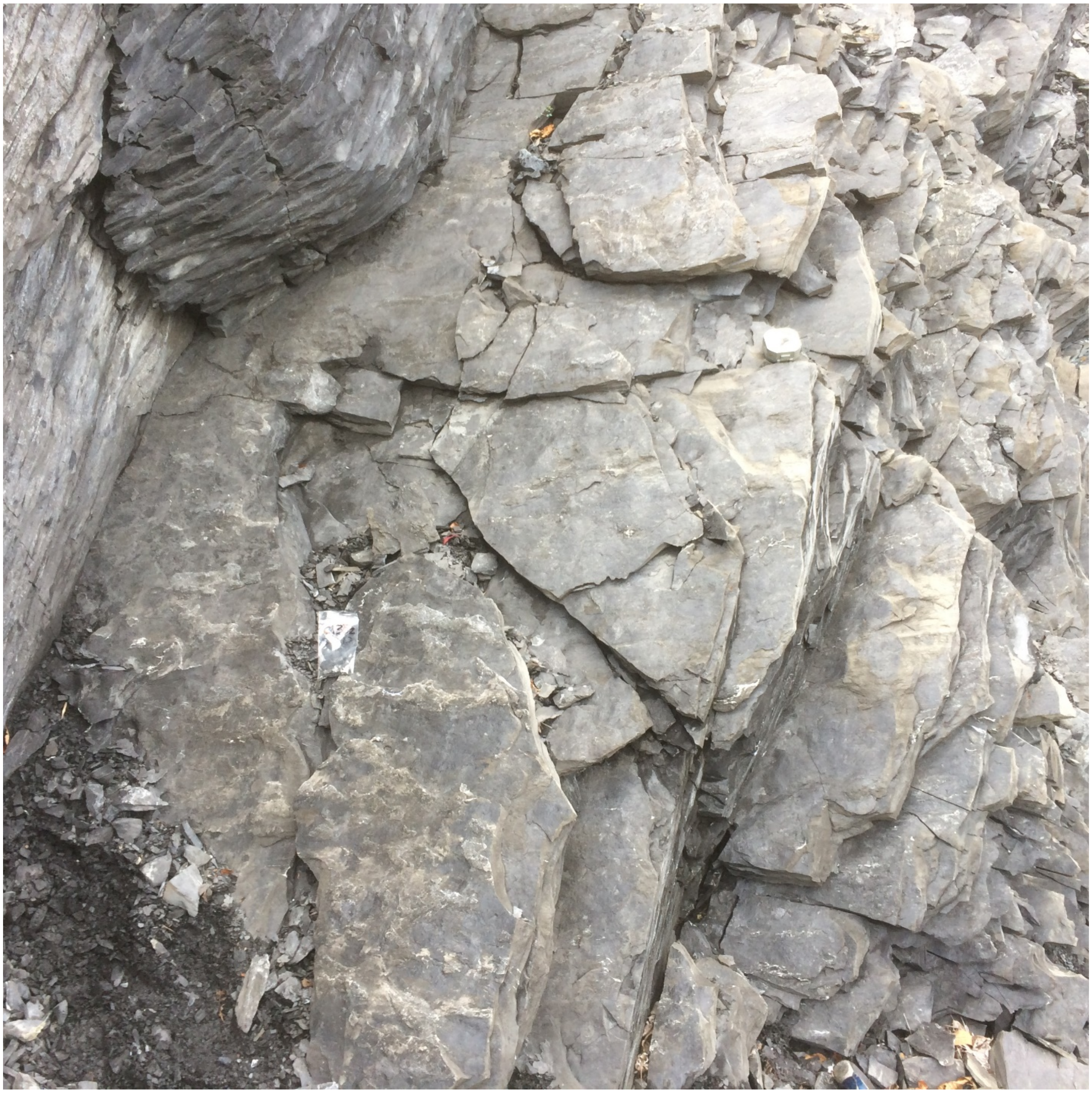

Lab Description:

C135C: Circles denote areas of interest for dating, not laser shots.

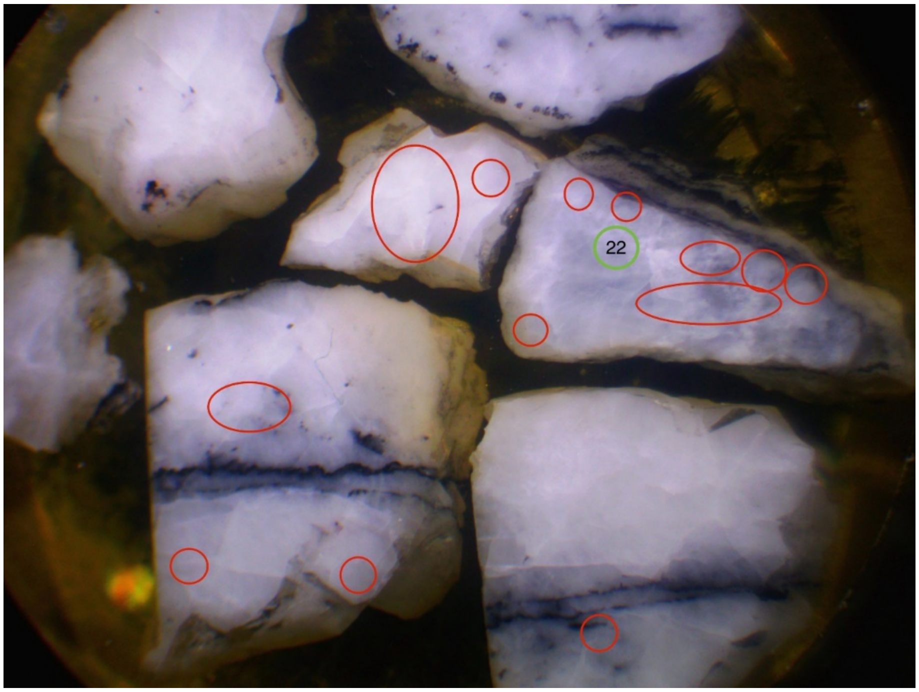

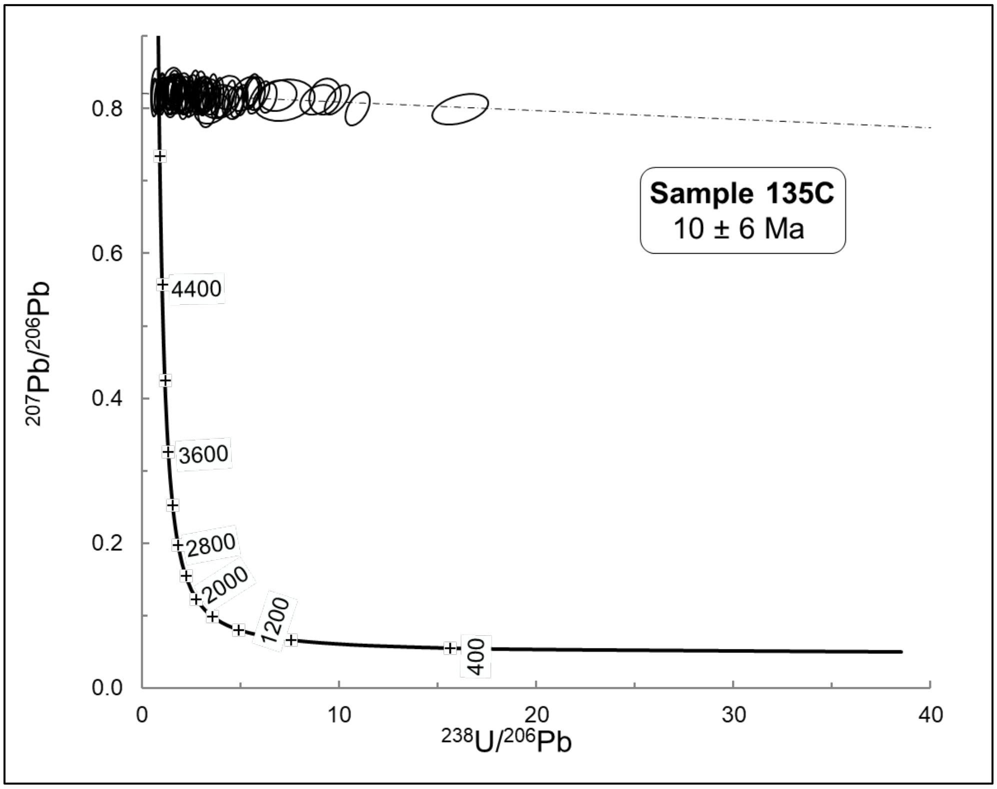

C135G: Circles denote areas of interest for dating, not laser shots.

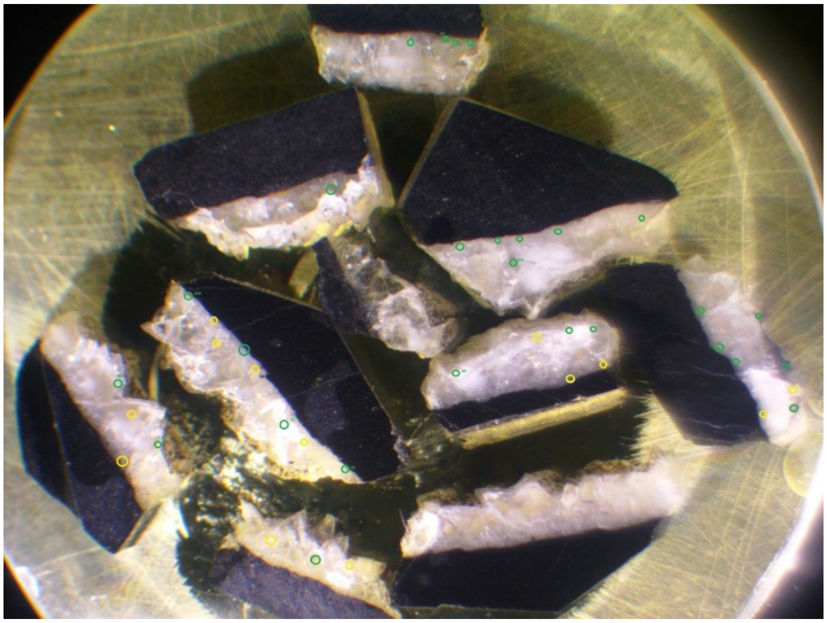

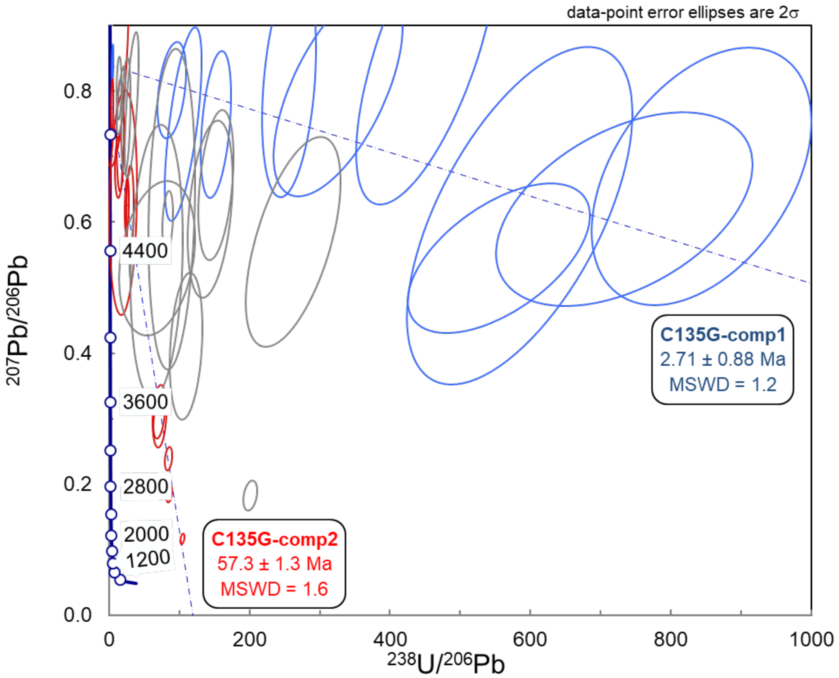

C135H: Circles denote areas of interest for dating, not laser shots.

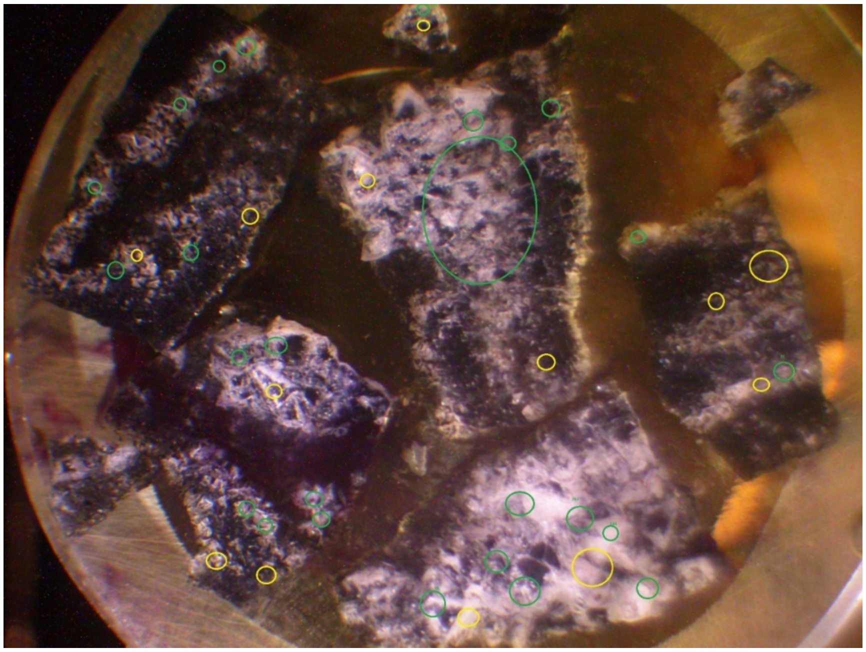

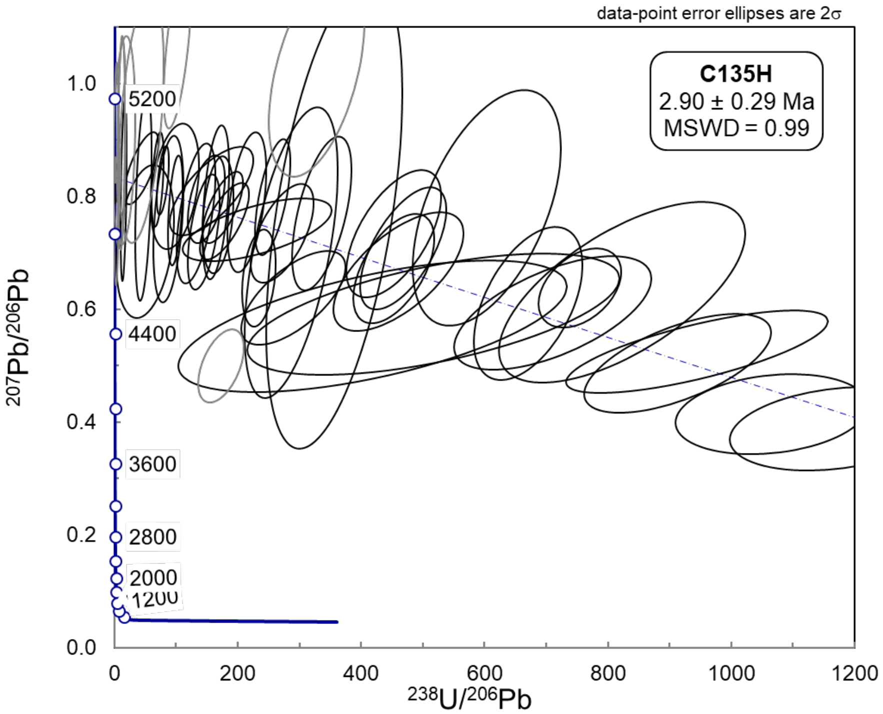

C135L: Circles denote areas of interest for dating, not laser shots.

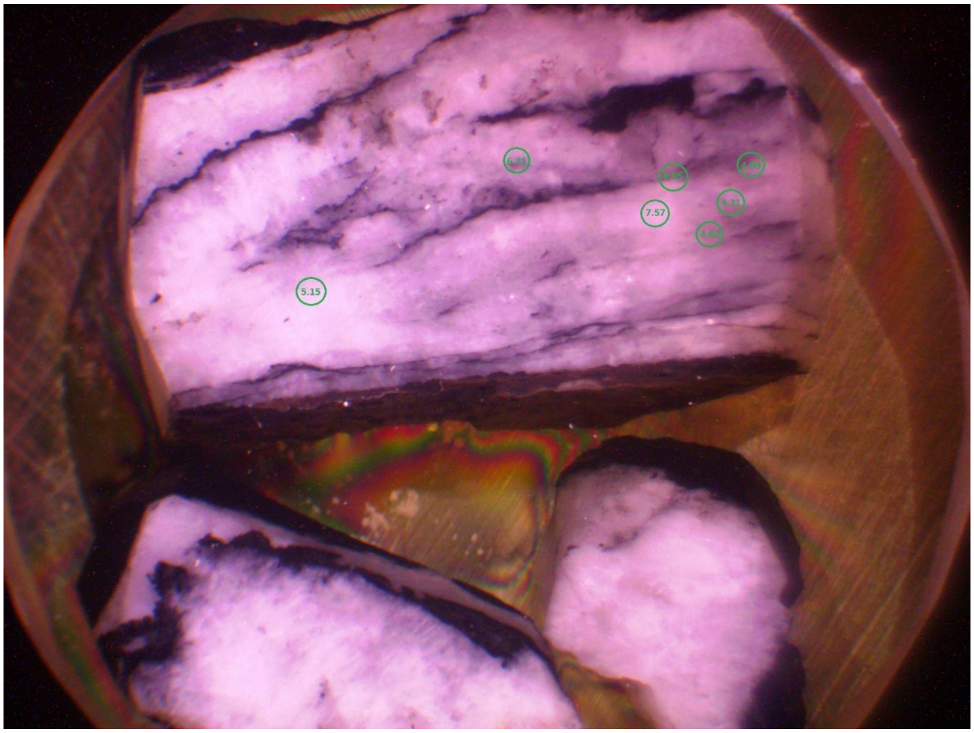

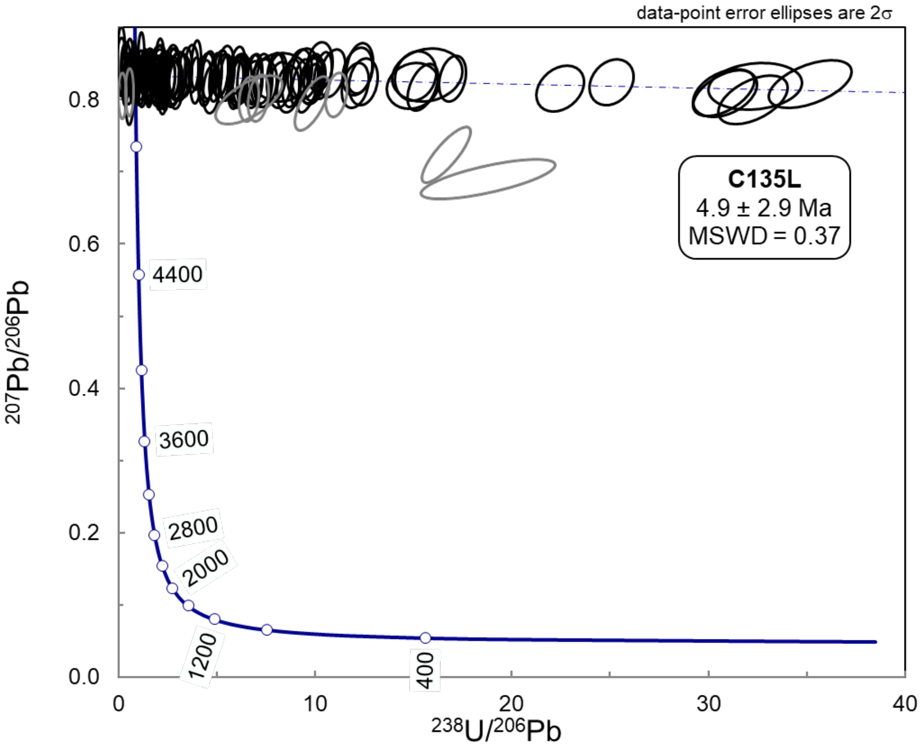

C135AE: Circles denote areas of interest for dating, not laser shots.

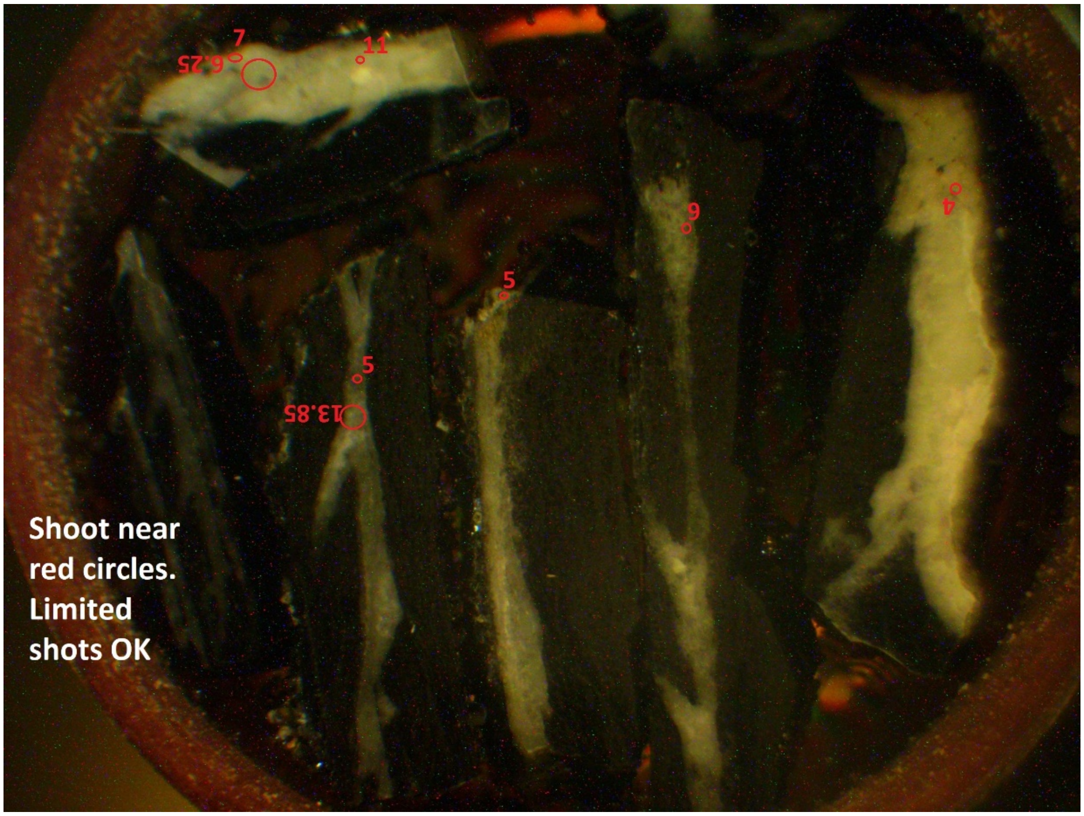

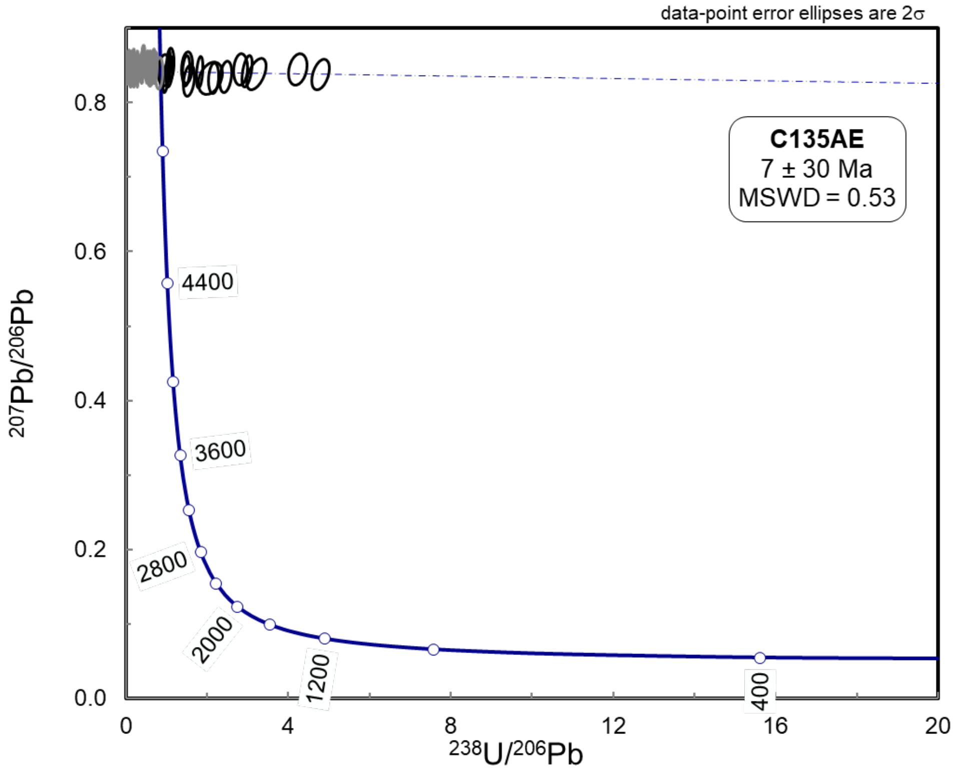

Supplement: Supplementary file 6 — Source Data [file 41467_2022_29680_MOESM6_ESM.zip › 1.zip!/appendix2_site_summaries_1to20/6_satterly_quarry_final.pdf]
