# Supplementary material for: Pace of passive margin tectonism revealed by U-Pb dating of fracture-filling calcite
Source: Nat Commun. 2022 Apr 12;13:1953. doi: 10.1038/s41467-022-29680-z (PMC9005700; doi:10.1038/s41467-022-29680-z)

7a: Downtown Vergennes (44.16792, -73.25447)

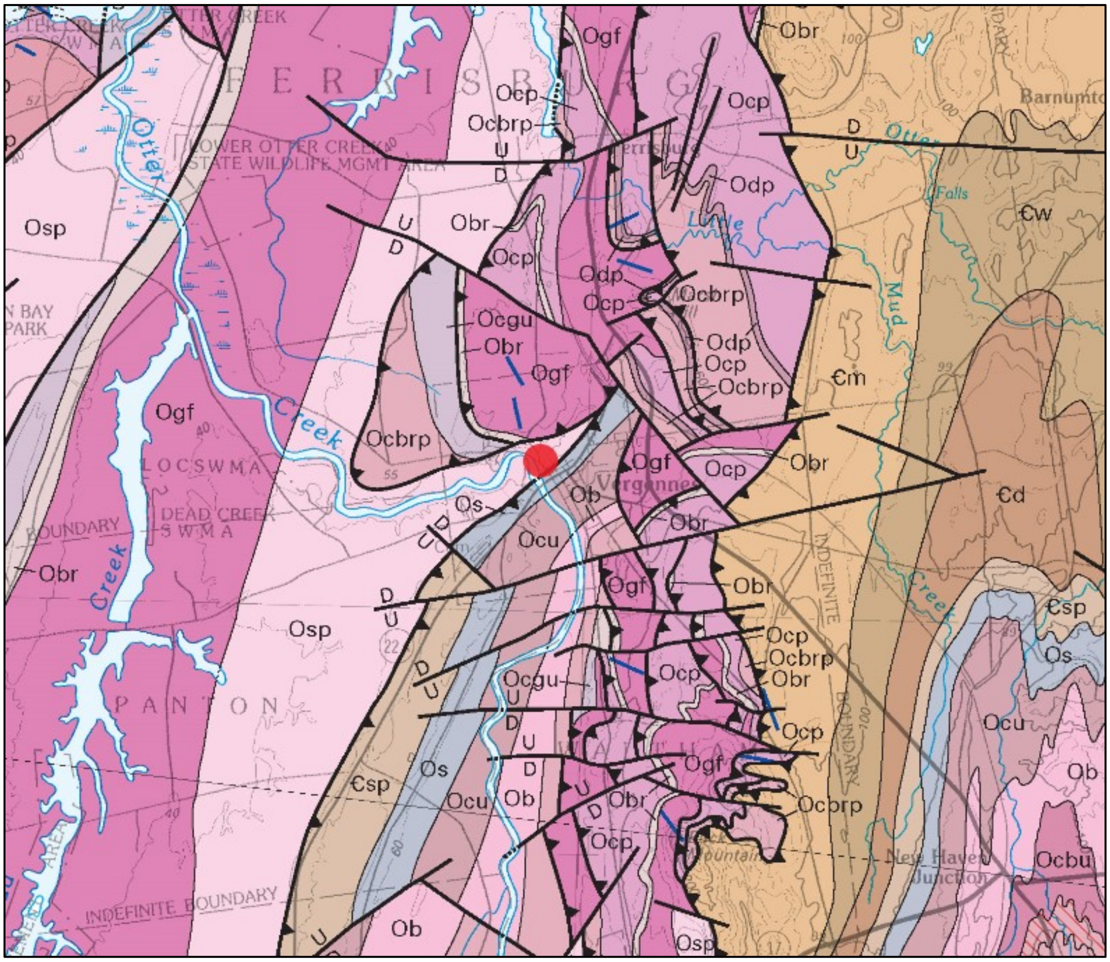

Figure 1: Sample location on state geologic map of Ratcliffe at al. (2011)

<u>Number of failed samples from same site:</u> none

<u>Bedrock Unit</u>: Stony Point Formation (Osp)

<u>Ages</u>: **C130**: 95.5 ± 4 Ma

<u>Summary</u>: This vein is exposed on east side of MacDonough Drive and is easily accessible by vehicle and/or sidewalk. Vein is composed of quartz + calcite from a 2-5 cm thick Mode 1 vein with strike/dip of 289°/71°NE. Vein has minimal offset and is very discrete, although not entirely planar. The surrounding rocks are intact and not shattered. This is interpreted as a fluid-assisted mode 1 dilational vein, apparently recording a NNE-SSW σ_3 stress orientation. The presence of significant quartz in the vein is fairly rare in our study area and suggests fluids may be sourced from depth and not derived from local bedrock. The roughly E-W orientation of this vein is broadly consistent with set of mapped faults in the Vergennes area (Fig. 1), but does not match well with major topographic lineaments mapped on a shaded relief image (Fig. 2 and 3).

__Regional Lineament Analysis__:

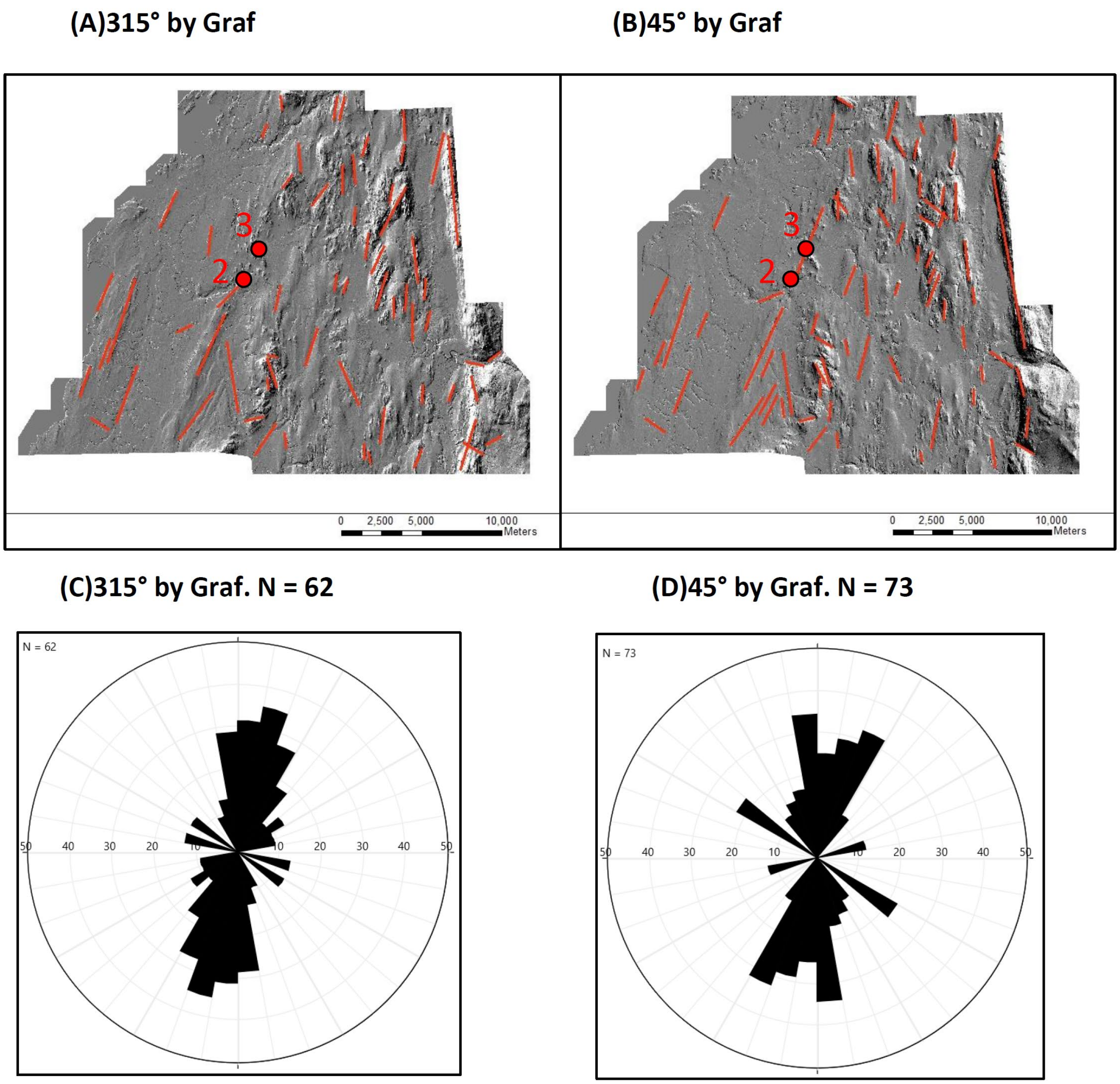

Figure 2: Lineaments mapped by student Sam Graf around the Vergennes Outcrop (labaled site 2) and Satterly Quarry (labeled site 3) using hillshade images with azimuth angles 315° (left) and 45° (right). Study sites are shown by red dots. Rose diagrams depict a general north-northeast to south-southwest trend of plotted lineaments.

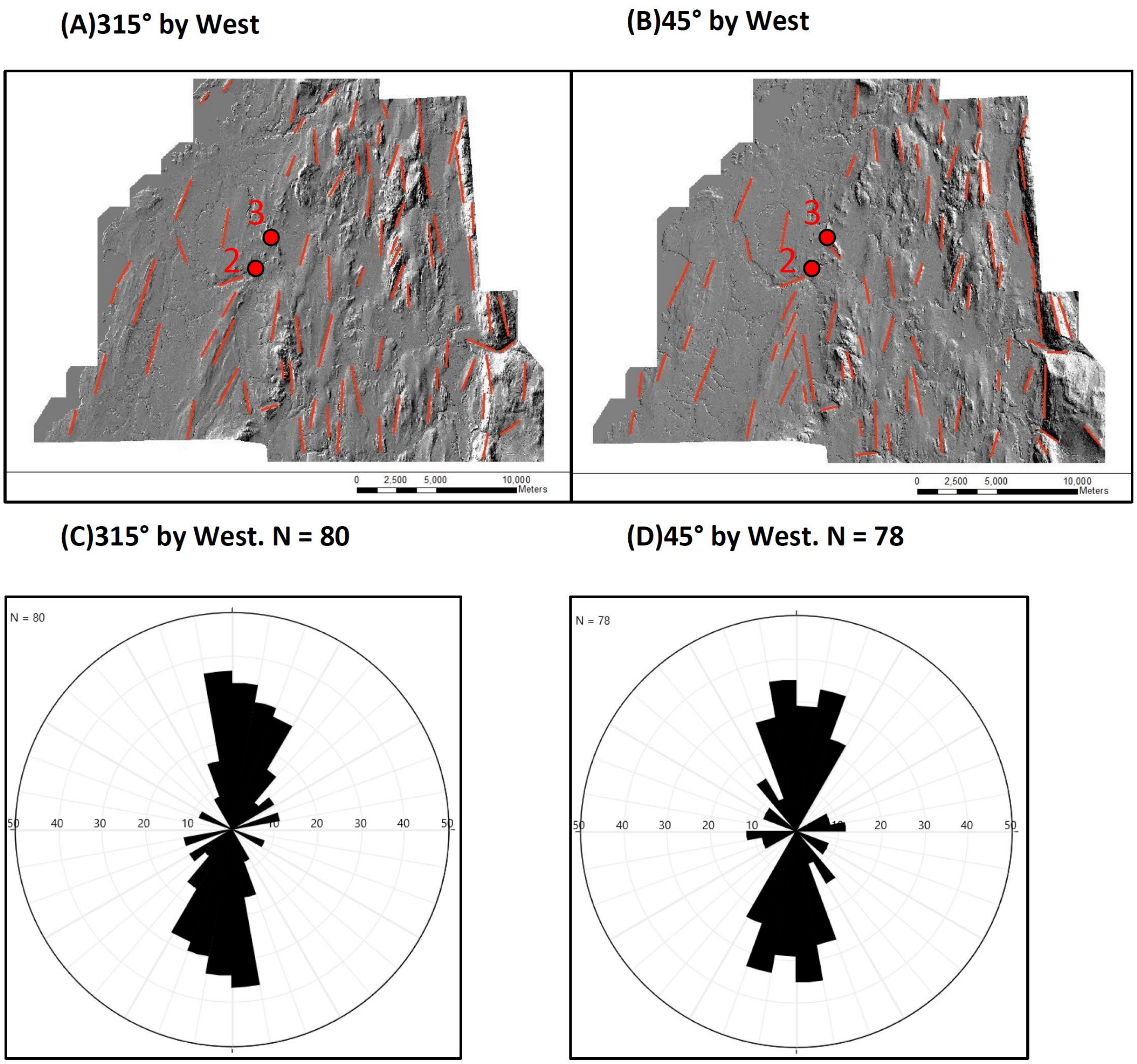

Figure 3: Lineaments mapped by Professor Dave West around the Vergennes Outcrop (labeled site 2) and Satterly Quarry (labeled site 3) using hillshade images with azimuth angles 315° (left) and 45° (right). Study sites are shown by red dots. Rose diagrams depict a general north-northeast to south-southwest trend of plotted lineaments.

Field Observations:

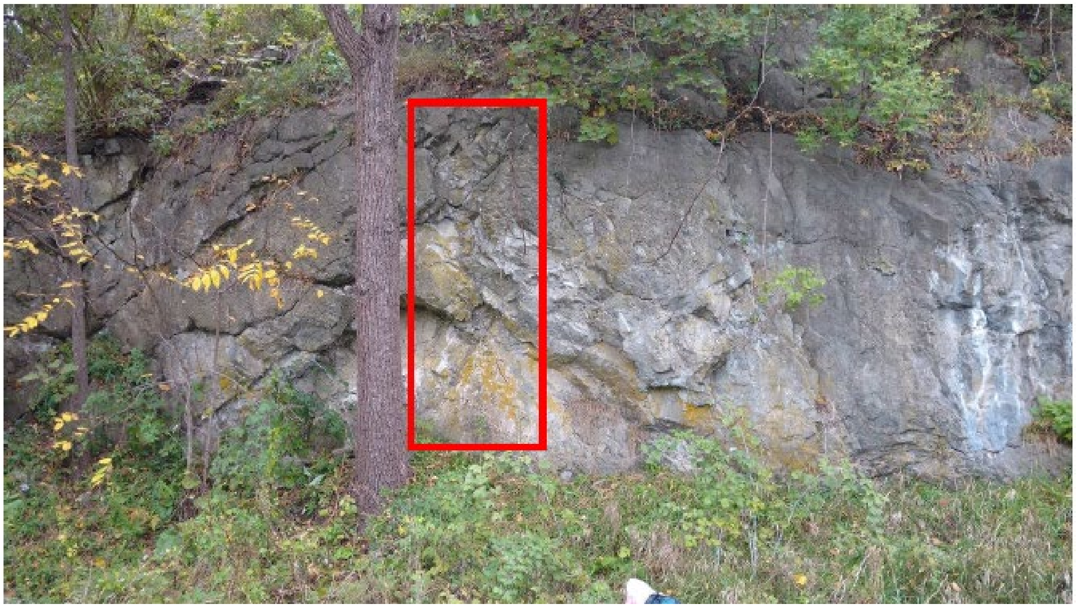

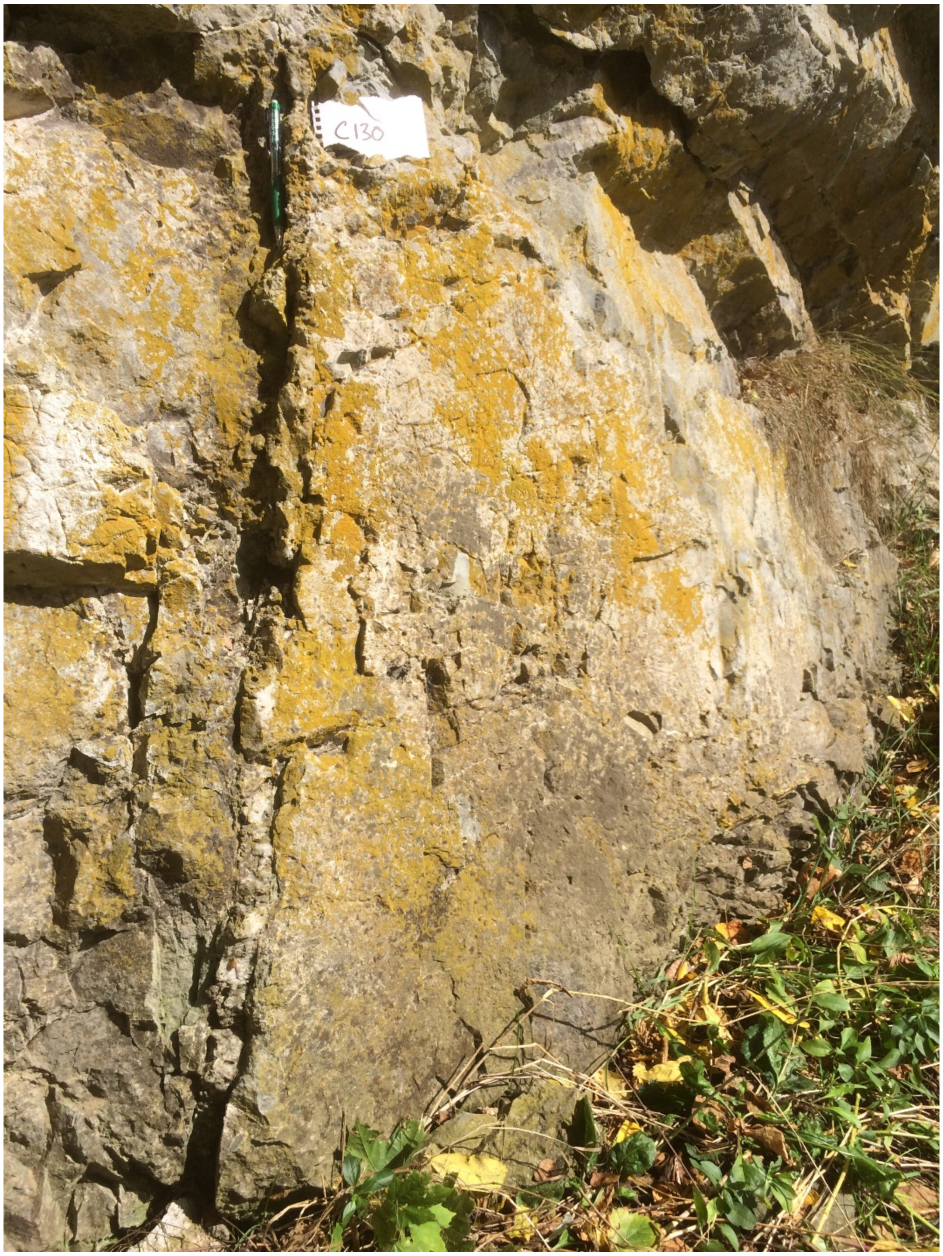
C130

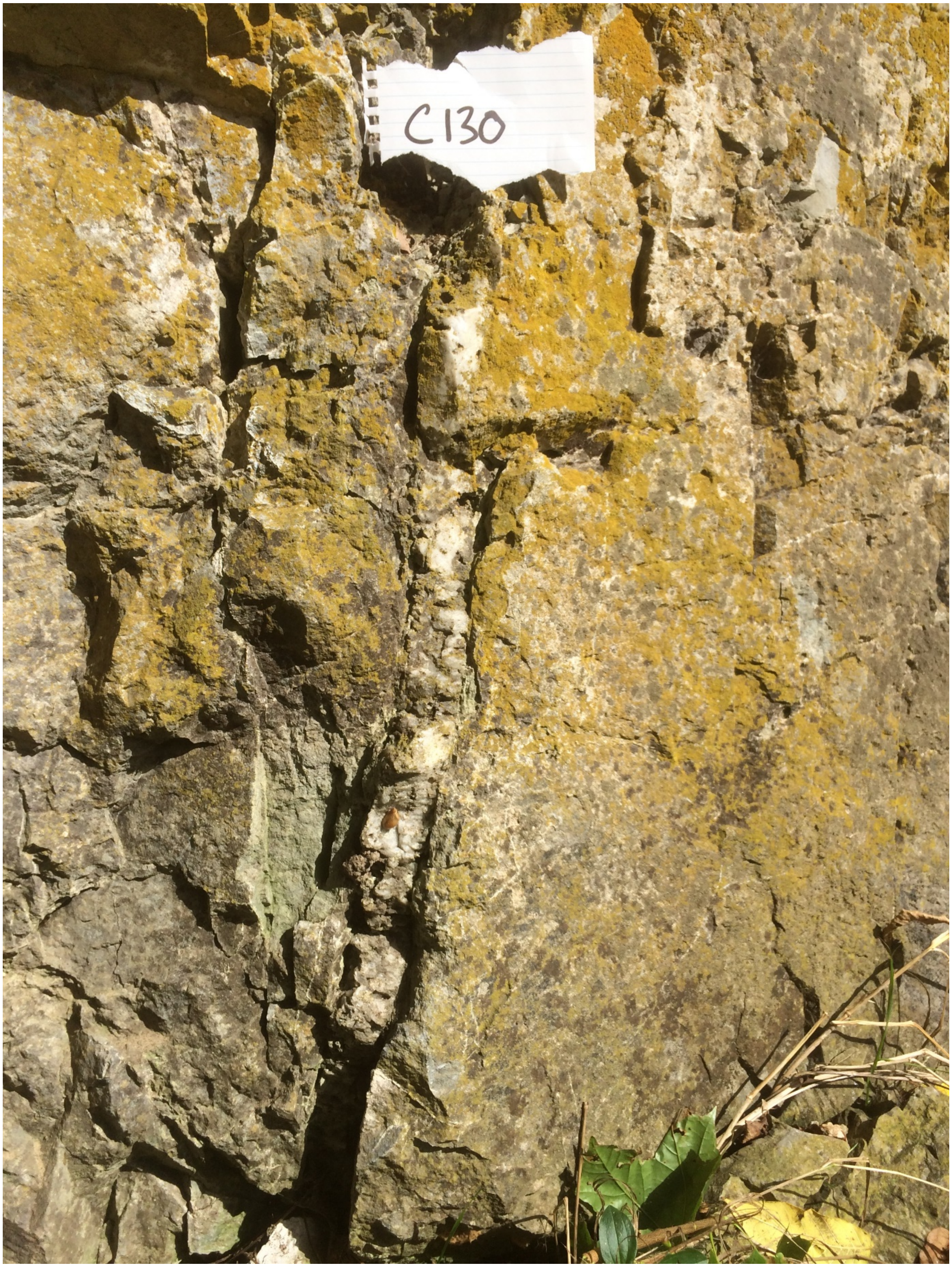
C130

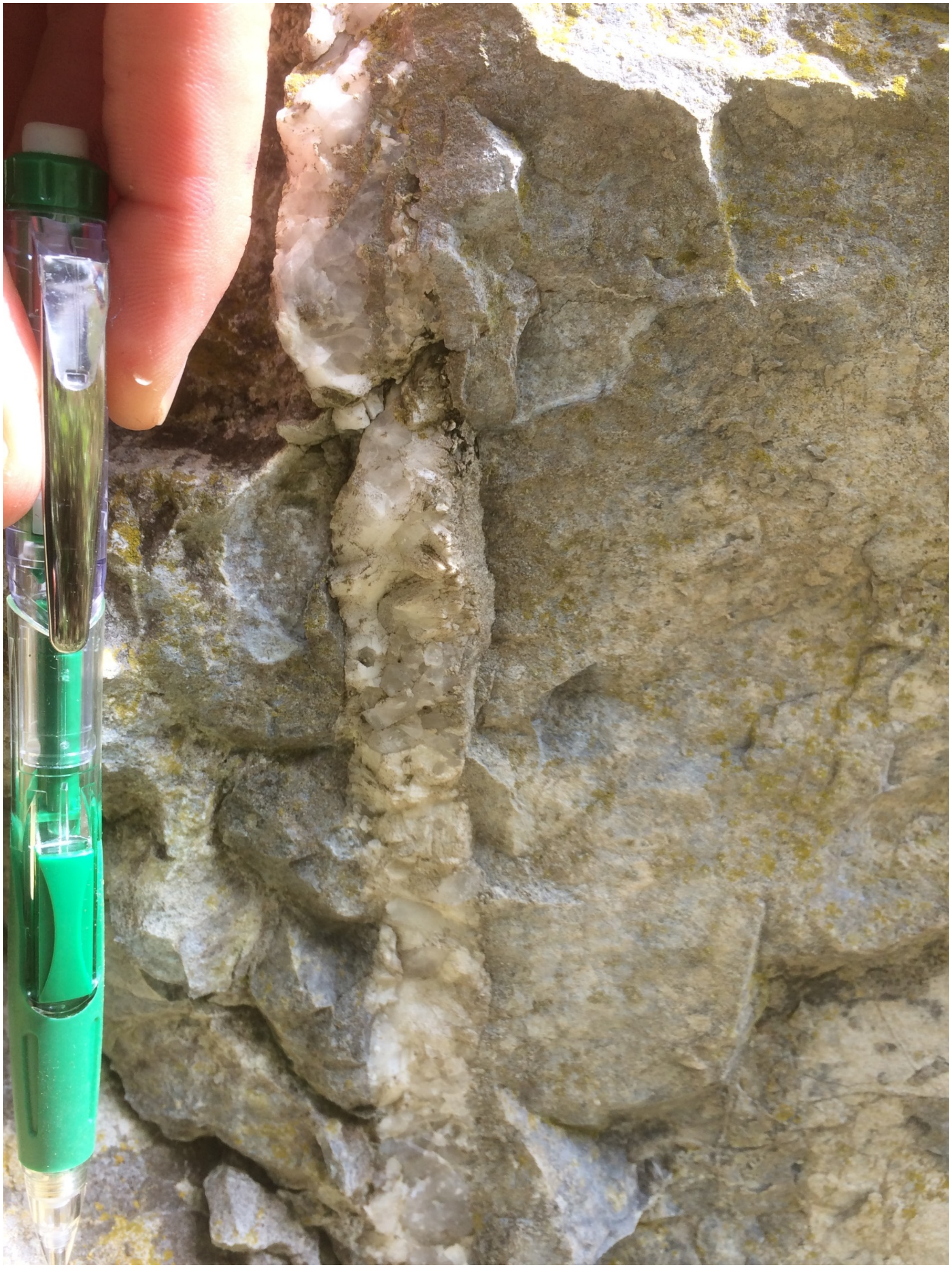

Lab Description: circles denote areas of interest, not laser shots

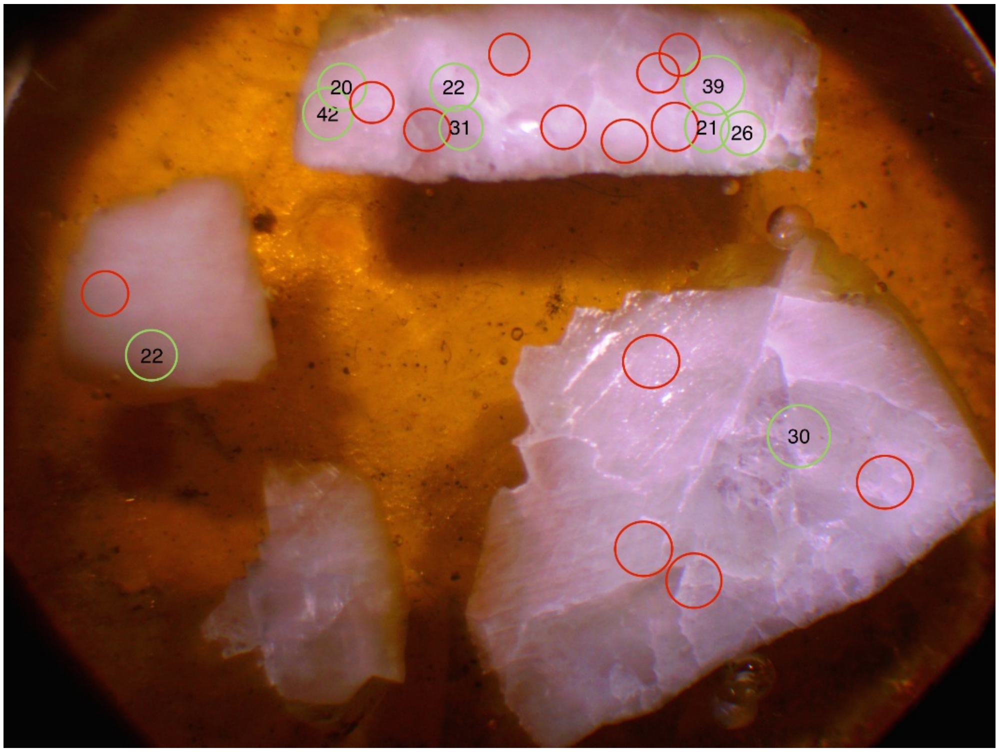

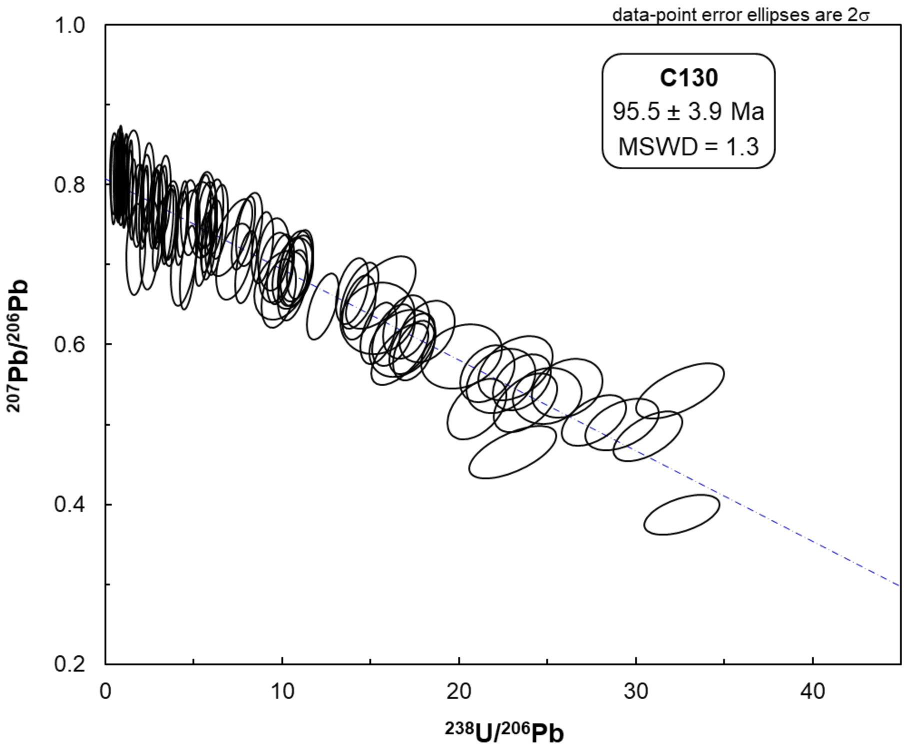

Supplement: Supplementary file 6 — Source Data [file 41467_2022_29680_MOESM6_ESM.zip › 1.zip!/appendix2_site_summaries_1to20/7a_vergennes_final.pdf]
