# Supplementary material for: Pace of passive margin tectonism revealed by U-Pb dating of fracture-filling calcite
Source: Nat Commun. 2022 Apr 12;13:1953. doi: 10.1038/s41467-022-29680-z (PMC9005700; doi:10.1038/s41467-022-29680-z)

7b: Avery Road, Ferrisburgh, VT (44.19851, -73.25789)

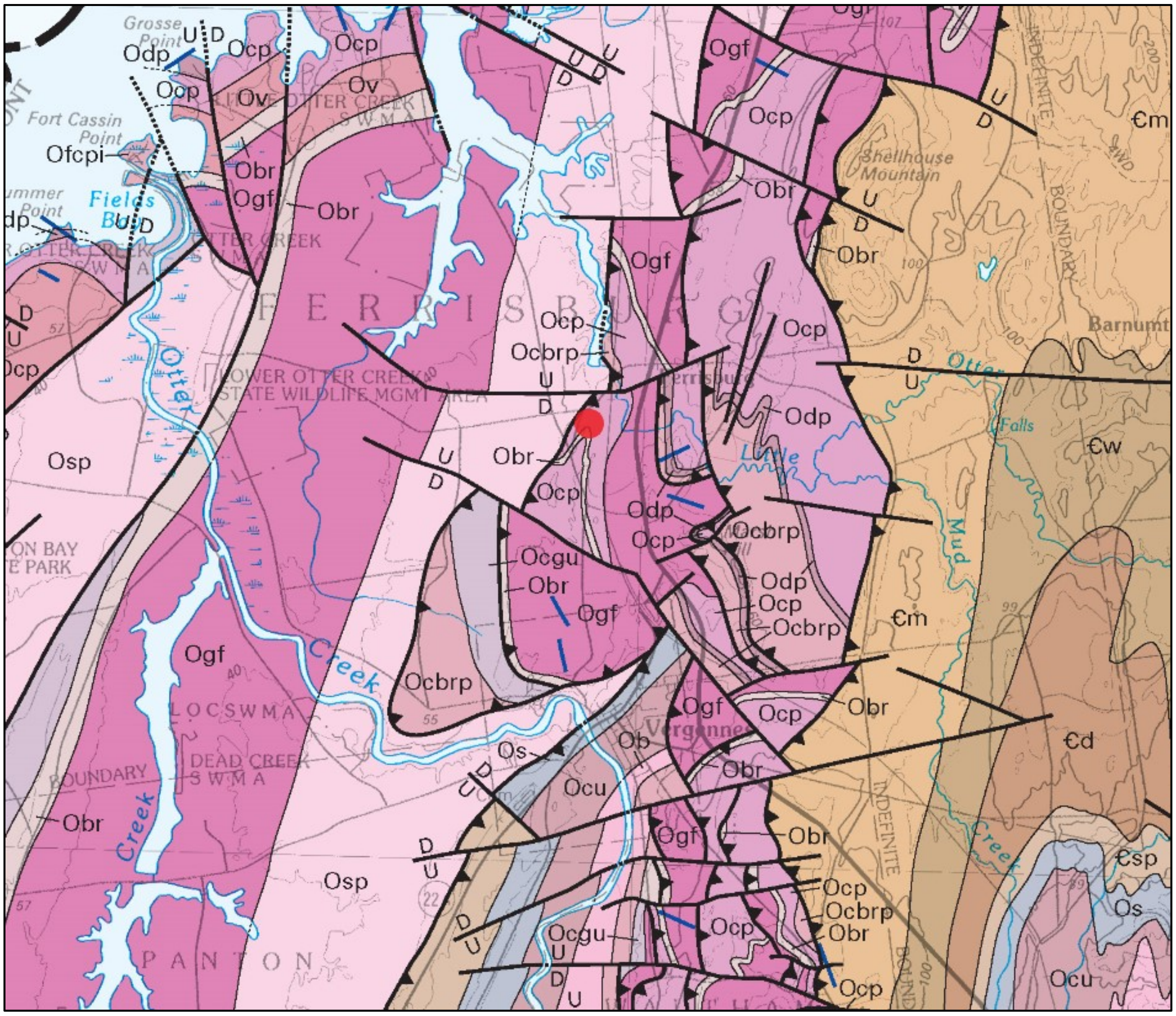

Figure 1: Sample location on state geologic map of Ratcliffe at al. (2011)

Failed samples from same site: C131A,B,C,D,F

Bedrock Unit: Crown Point Formation (Ocp)

Ages: C131E: 444 ± 76 Ma

Summary: This site is located in the roadside ditch on the east side of Avery Rd., just east of the town of Ferrisburgh, VT. It is a complex set of orthogonal veins which intersect and cross-cut each other. Contradictory cross-cutting relationships suggests the orthogonal veins are roughly contemporaneous. The U-Pb data suggests the veins are “Taconic” in age. No detailed structural analysis has been attempted.

<u>Field Descriptions</u>:

C131E: (left-most bag in image)

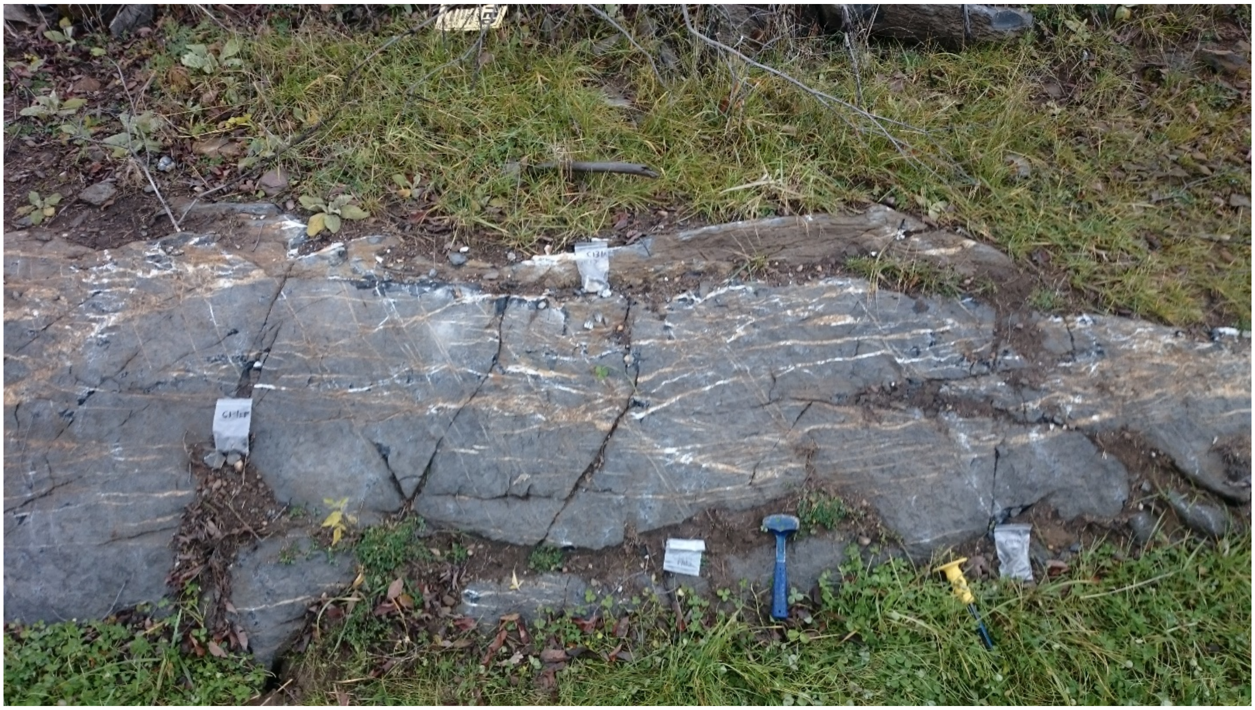

Lab Description:

C131E:

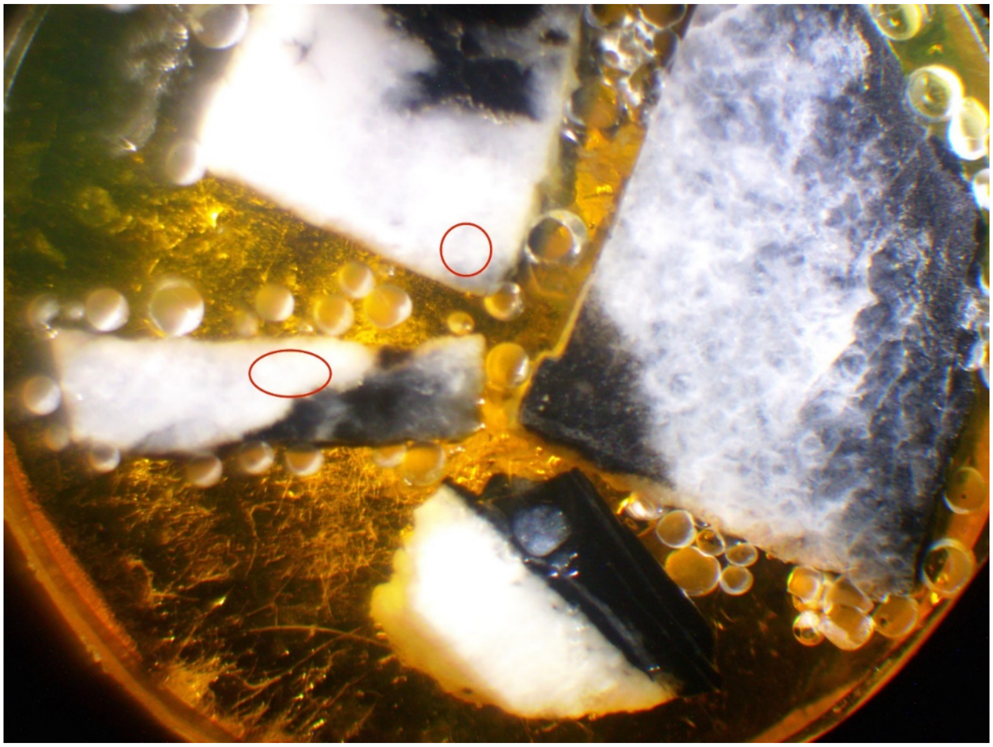

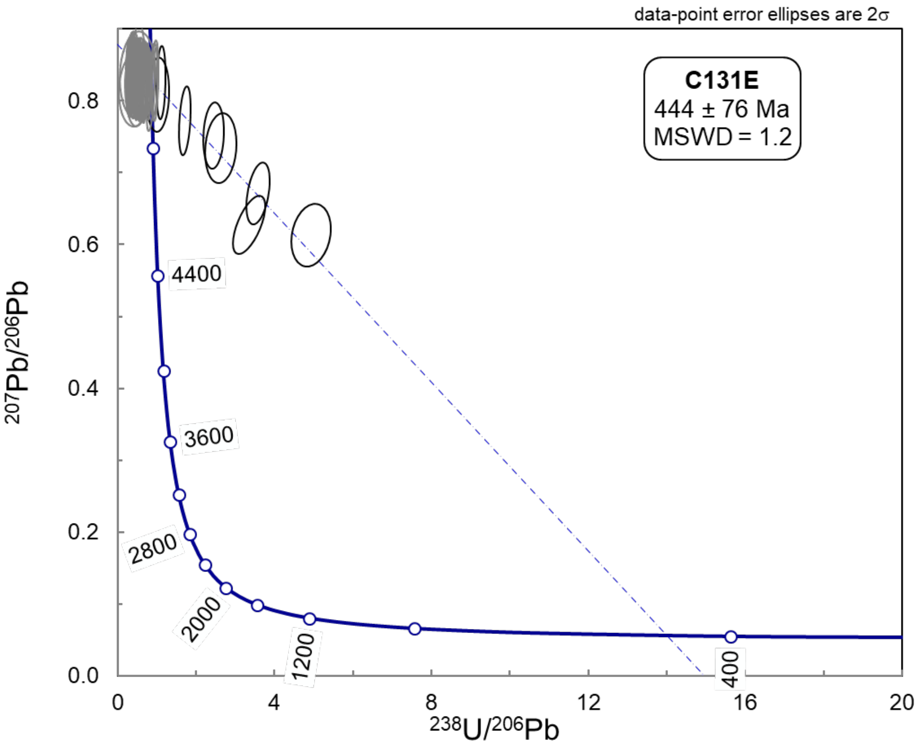

Supplement: Supplementary file 6 — Source Data [file 41467_2022_29680_MOESM6_ESM.zip › 1.zip!/appendix2_site_summaries_1to20/7b_avery_road_final.pdf]
