# Supplementary material for: Pace of passive margin tectonism revealed by U-Pb dating of fracture-filling calcite
Source: Nat Commun. 2022 Apr 12;13:1953. doi: 10.1038/s41467-022-29680-z (PMC9005700; doi:10.1038/s41467-022-29680-z)
Supplement: Supplementary file 6 — Source Data [file 41467_2022_29680_MOESM6_ESM.zip › 1.zip!/appendix2_site_summaries_1to20/8_Fisk_Quarry_final.pdf]

8: Fisk Quarry, Isla La Motte (44.845413, -73.361513)

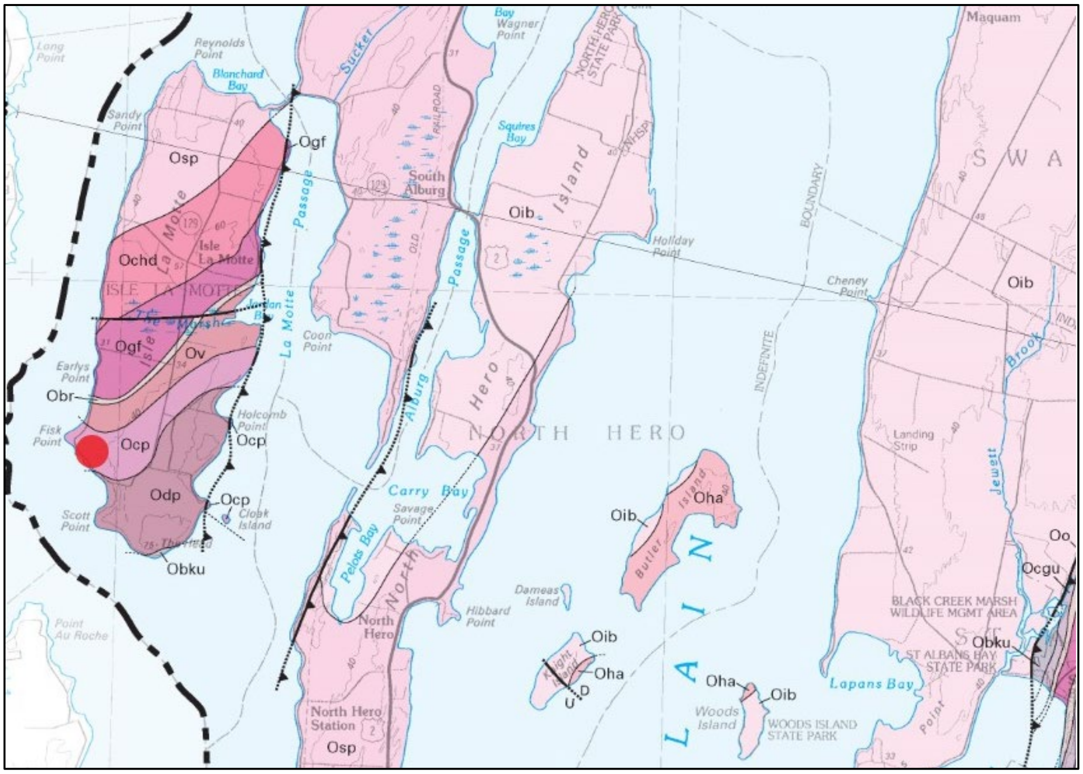

Figure 1: Sample location on state geologic map of Ratcliffe at al. (2011).

Failed samples from same site: C170

Bedrock Unit: Crown Point Formation (Ocp)

Ages: C169: 97.6 ± 3.7 Ma

Summary: This is site is located in the Fisk Quarry on Isle La Motte and is accessible by car. The single dated sample is an ultra-planar, continuous, ~3 mm thick vein exposed on the flat bedding plane of the quarry floor with a strike/dip of 20°/90°. The vein is remarkable in that it fills a very discrete mode 1 fracture in a massively bedded rock unit that is nearly devoid of other fractures or faults. No evidence for slickenfibres or offset. Calcite texture and U-Pb isochron suggest a single crystallization event at the time of crack formation. We thus infer that the date of 97.6 Ma records a primary crack-forming event under an E-W σ_3 stress with a trend/plunge 110°/0°.

Field Observations:

C169

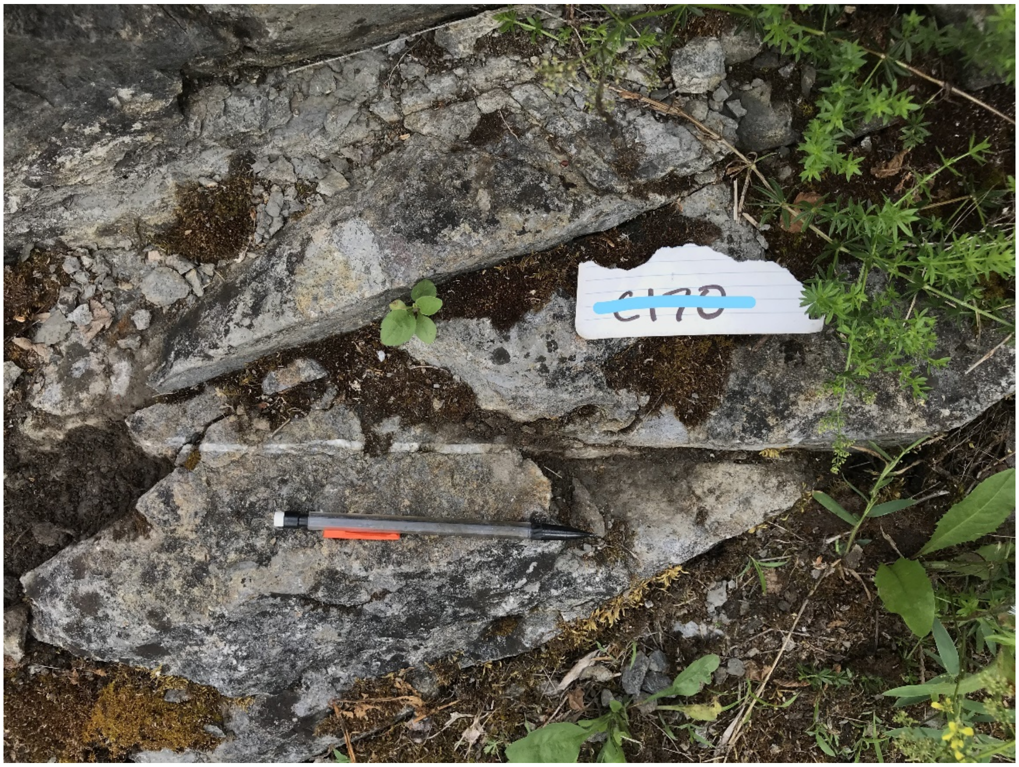

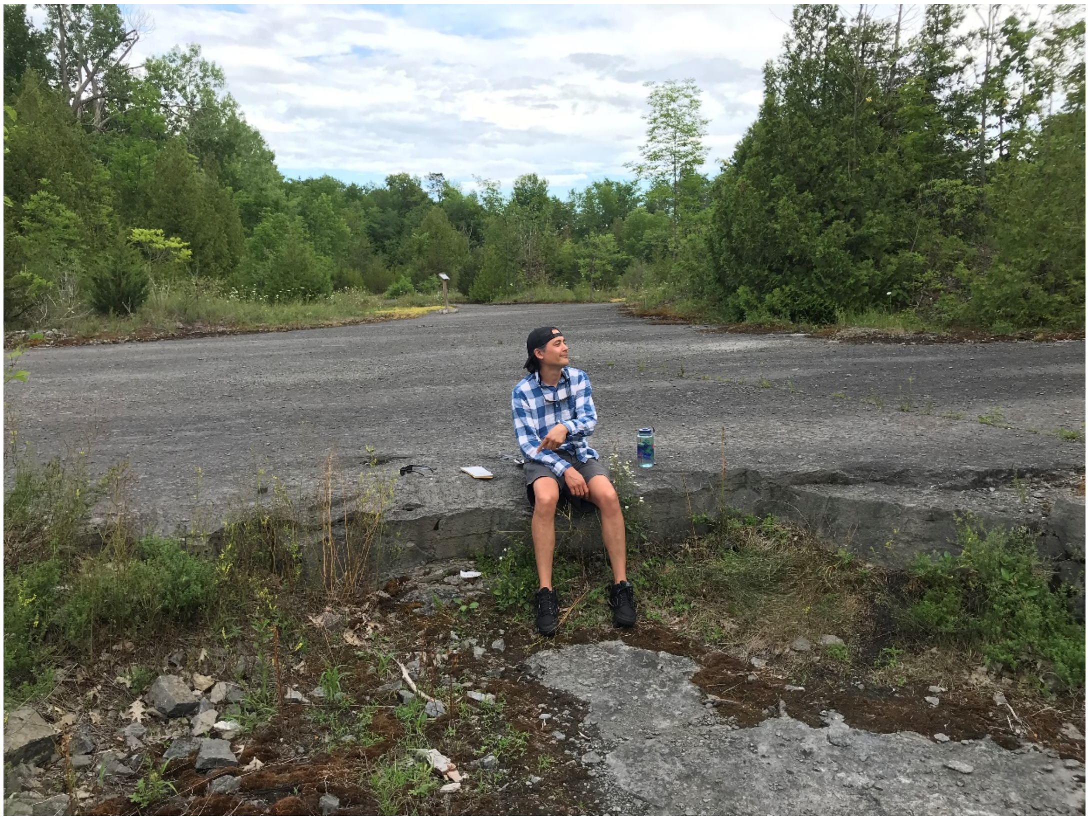

Lab Description:

C169: Clear, blocky calcite. Appears to be single generation, representative of fracture age.

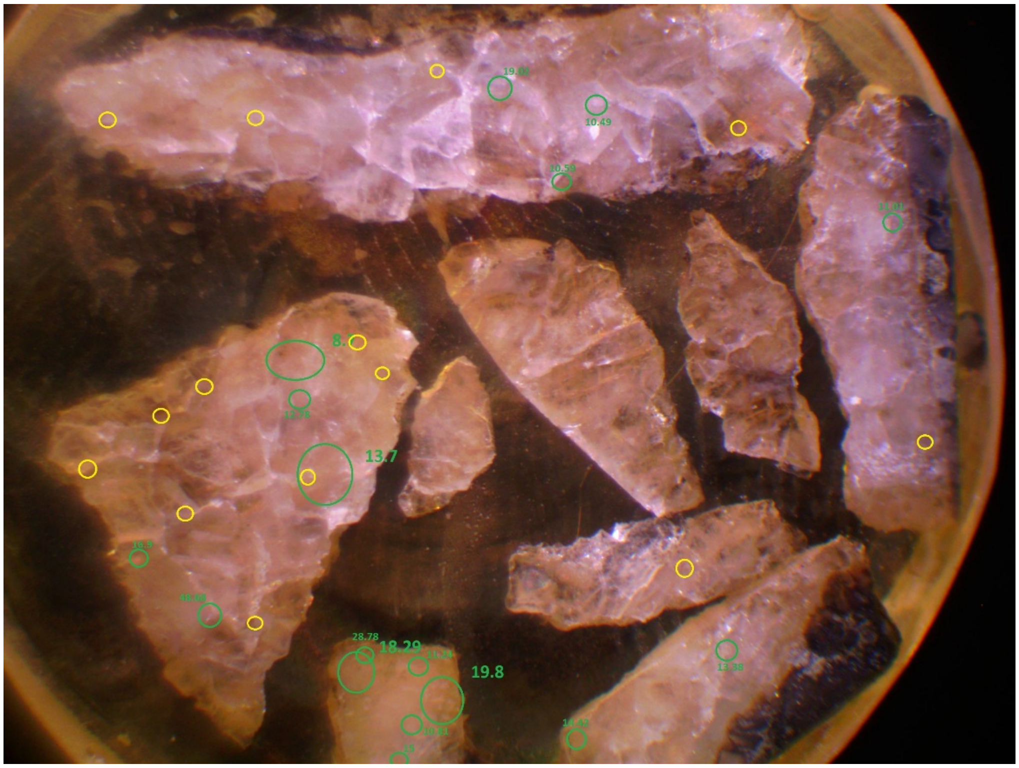

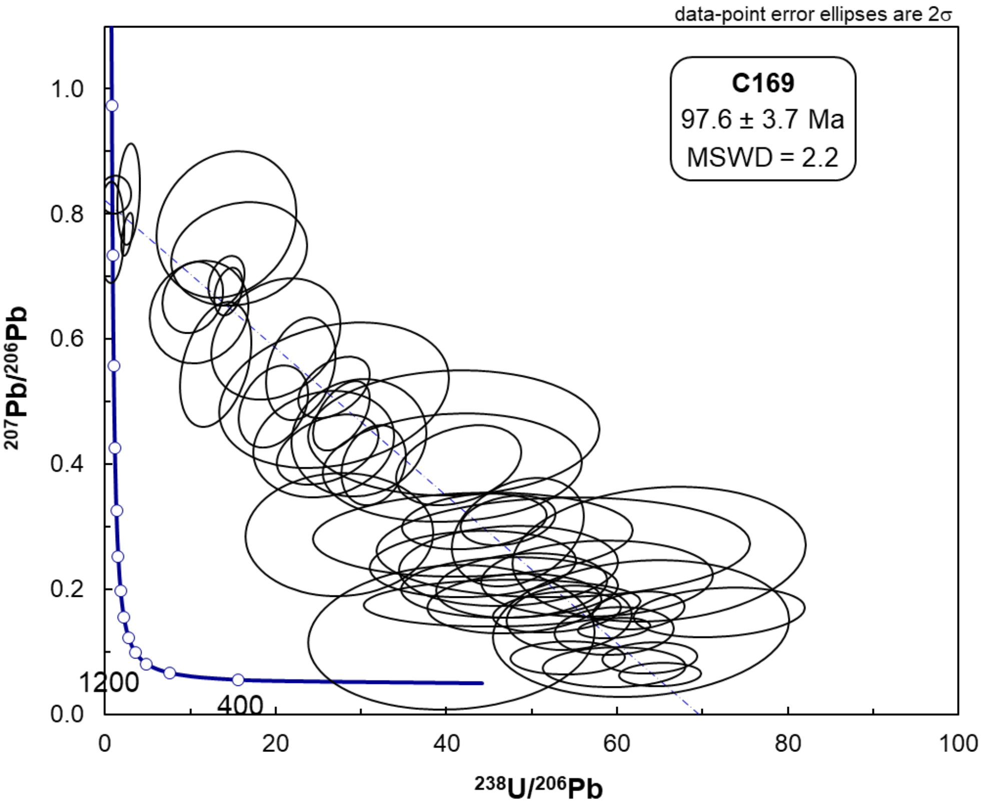

data-point error ellipses are 2σ
C169
97.6 ± 3.7 Ma
MSWD = 2.2
207Pb/206Pb
238U/206Pb
1200
400
0.0
0.2
0.4
0.6
0.8
1.0
0
20
40
60
80
100
