# Supplementary material for: Pace of passive margin tectonism revealed by U-Pb dating of fracture-filling calcite
Source: Nat Commun. 2022 Apr 12;13:1953. doi: 10.1038/s41467-022-29680-z (PMC9005700; doi:10.1038/s41467-022-29680-z)
Supplement: Supplementary file 6 — Source Data [file 41467_2022_29680_MOESM6_ESM.zip › 1.zip!/appendix2_site_summaries_1to20/9_apple_island_campground_final.pdf]

9: Apple Island Resort, South Hero (44.633367, -73.268208)

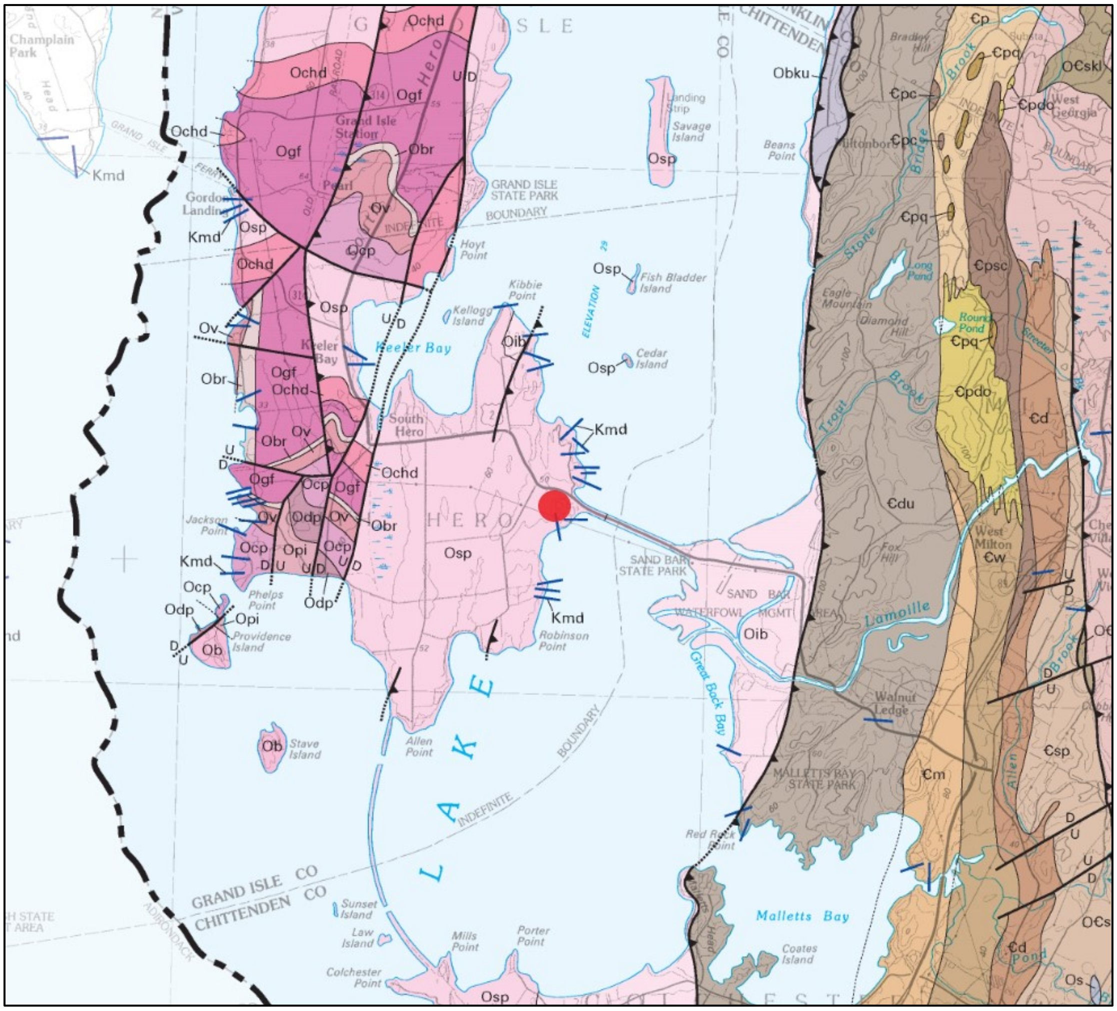

Figure 1: Sample location on state geologic map of Ratcliffe at al. (2011)

<u>Failed samples from same site</u>: C177B,D; C178 (slightly to north in campground?)

<u>Bedrock Unit:</u> Stony Point Formation (Osp)

<u>Ages</u>: **C177A**: 64.7 ± 1.2 & 16.7 ± 1.0; **C177C**: 74.5 ± 1.7 & 61.2 ± 1.0

<u>Summary</u>: This site is located at the southern side of the main camping area at the Apple Island Campground. Access is by car with permission. It consists of the Stony Point shale with a strong N-S oriented cleavage that appears to have been reactivated several times. The surfaces have calcite mineralized slip-surfaces with well-preserved slickensides, at least some of which record E-W extension. Clearly these weak rocks have accommodated several episodes of slip, with the earliest about 75 Ma, another about 62 Ma, and perhaps two more after that. Given the multi-generational nature of the calcite samples we are unable to reliably infer kinematics at this site and categorize at as ‘Fault With No Slip Sense. Interestingly, this is one of only two places where we see an event near ~62 Ma, the other being Queneska Island off of Shelburne Point.

<u>Field Descriptions</u>:

C177: 6 cm vein cross-cutting brittle bedding of Stony Point calcareous shale.   Sub-vertical.

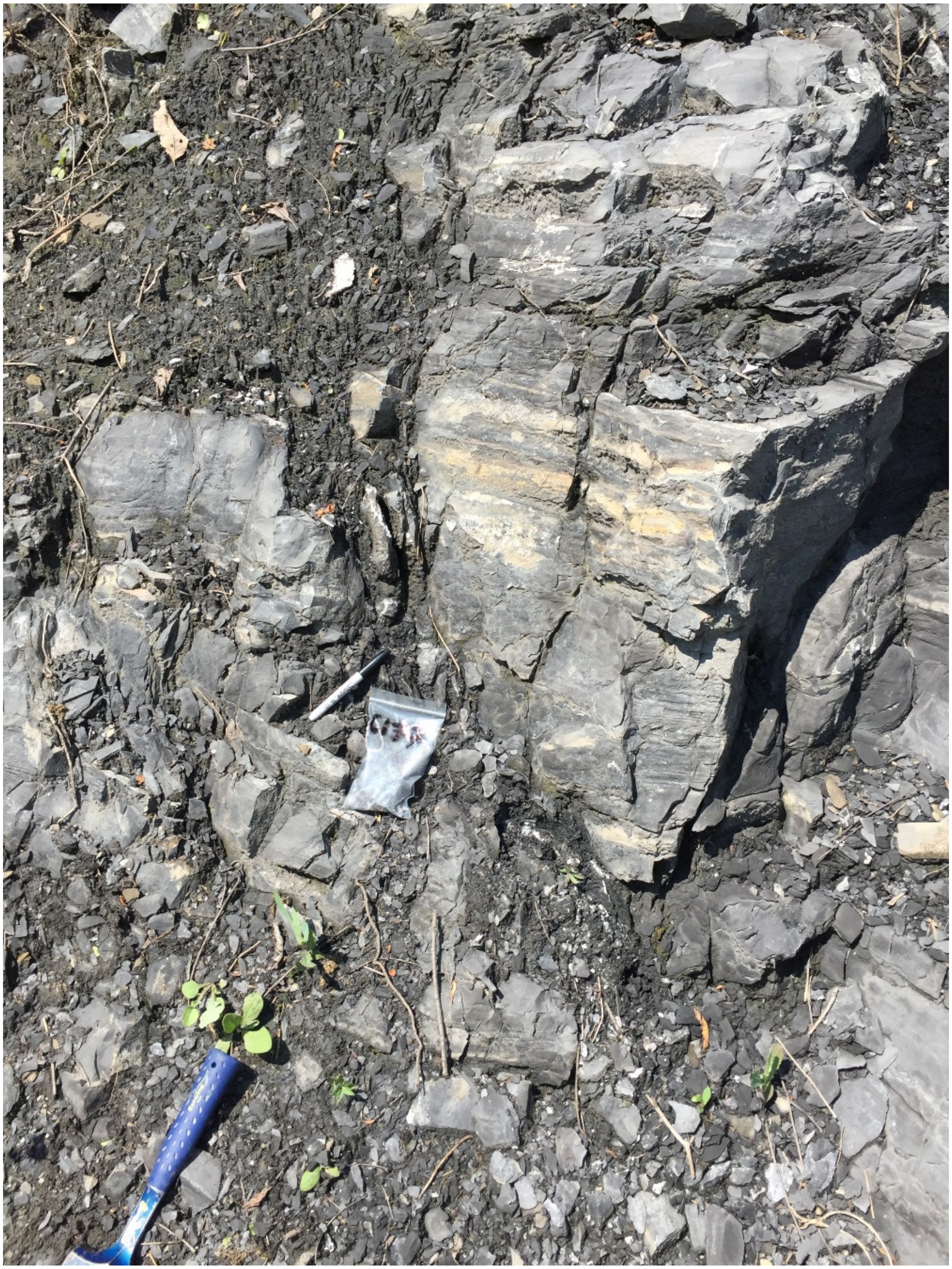

C177C:

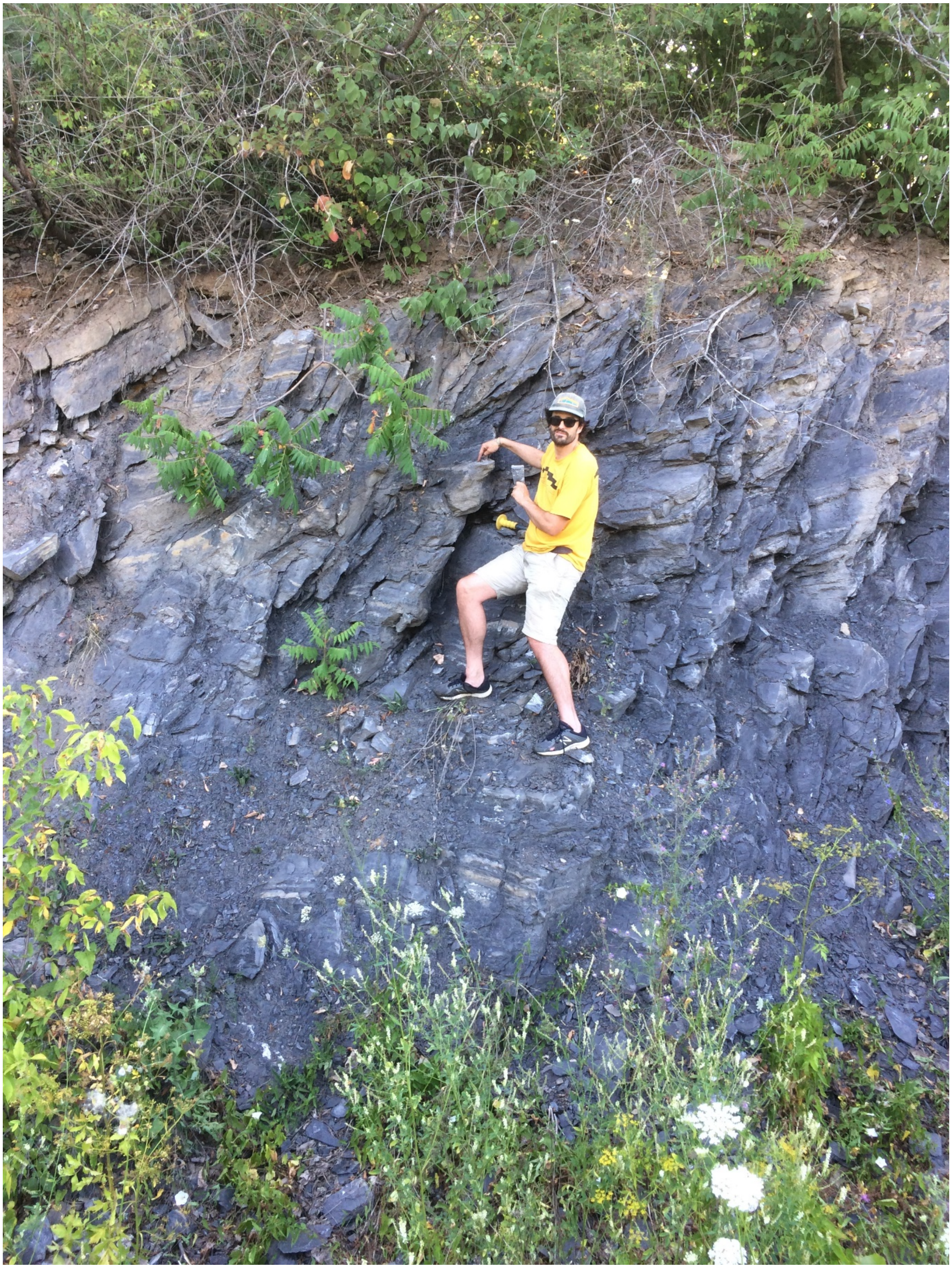

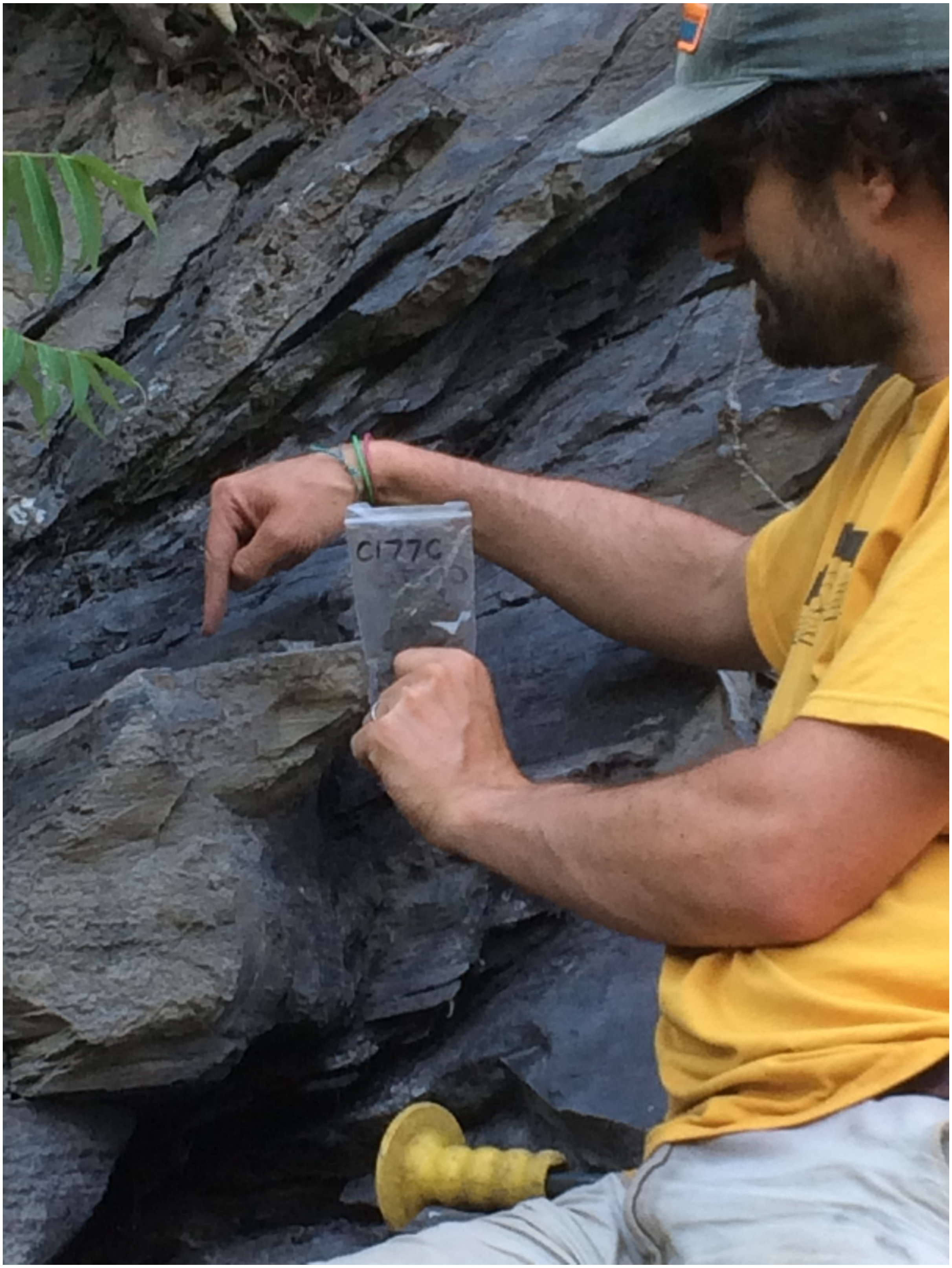
C177C

Lab Description:

C177: Blocky calcite ranging from milky to clear. Evidence for free growth of large crystals. Elemental mapping and dating suggest multiple growth domains. Younger Miocene age may come from a later generation of transluscent calcite on the right-most fragment, radiating from the black piece of country rock. Need to cross check GIS files or Liesel's figures. Thus, bulk of vein is probably 65 Myr and Miocene age may be a recrystallization event. Possibly third event around 45 Ma.

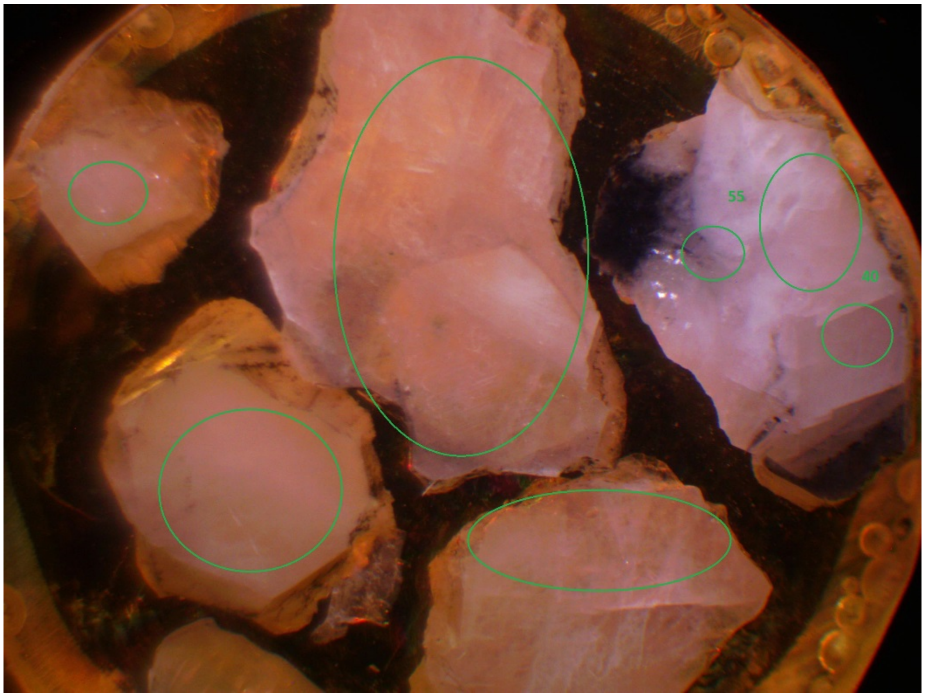

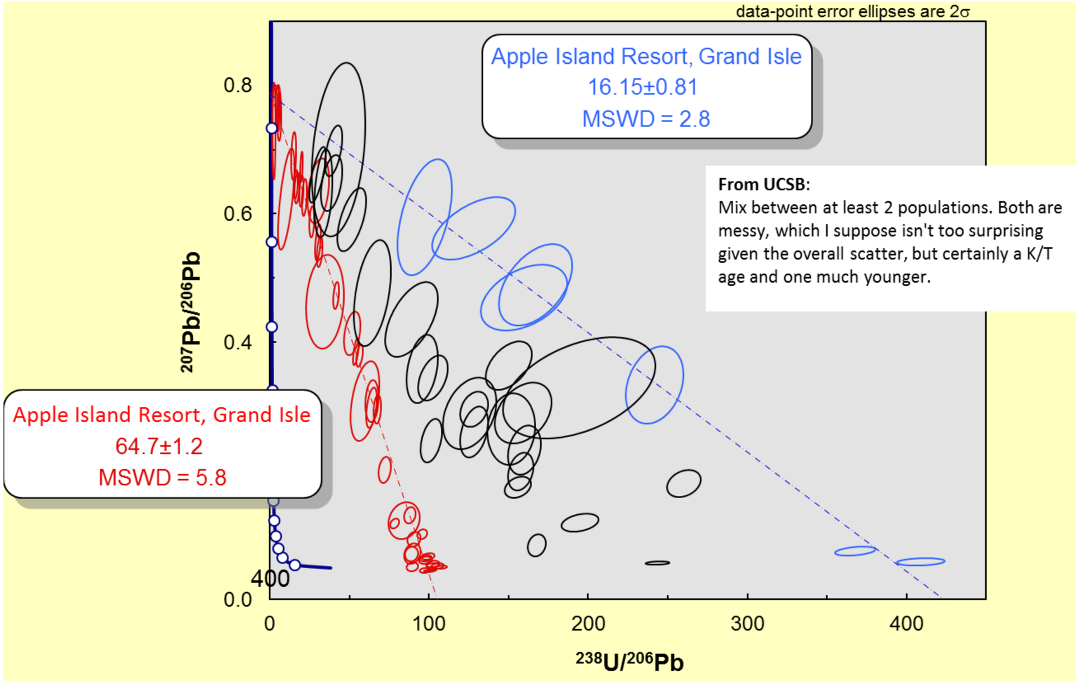

C177C:

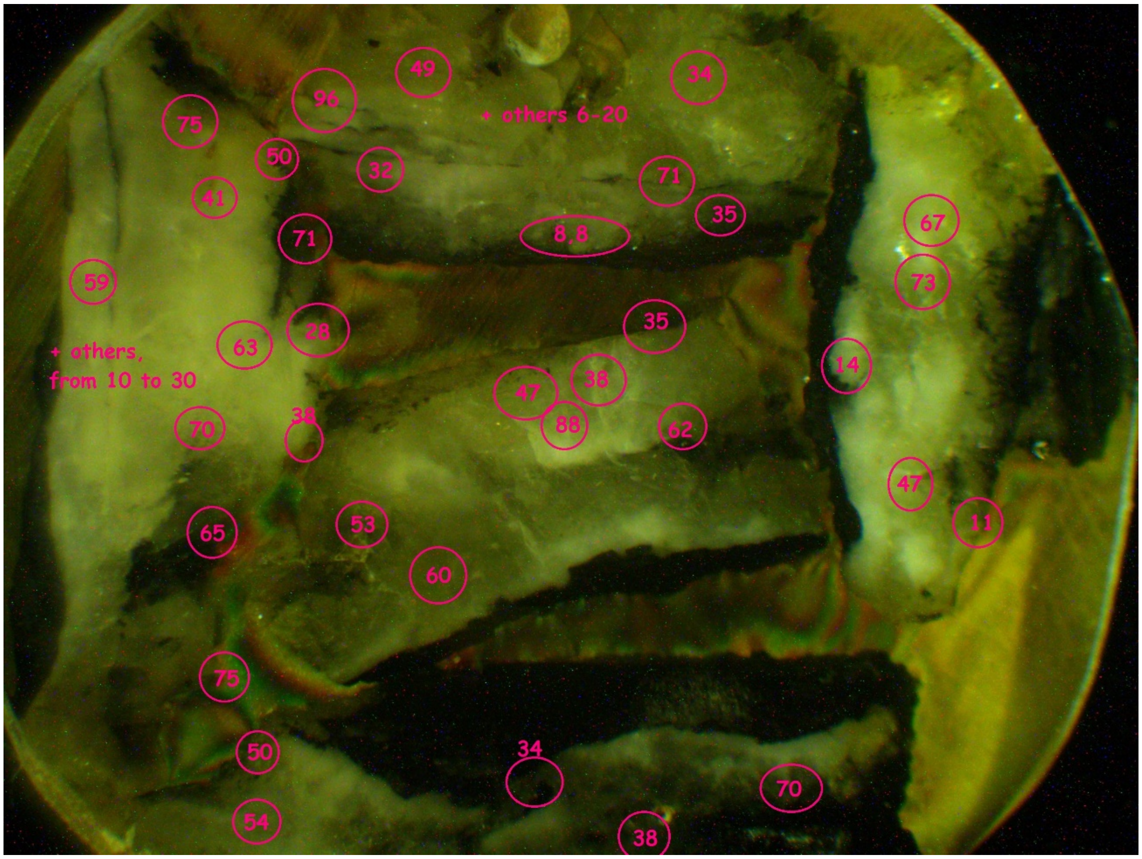

+ others 6-20
+ others, from 10 to 30

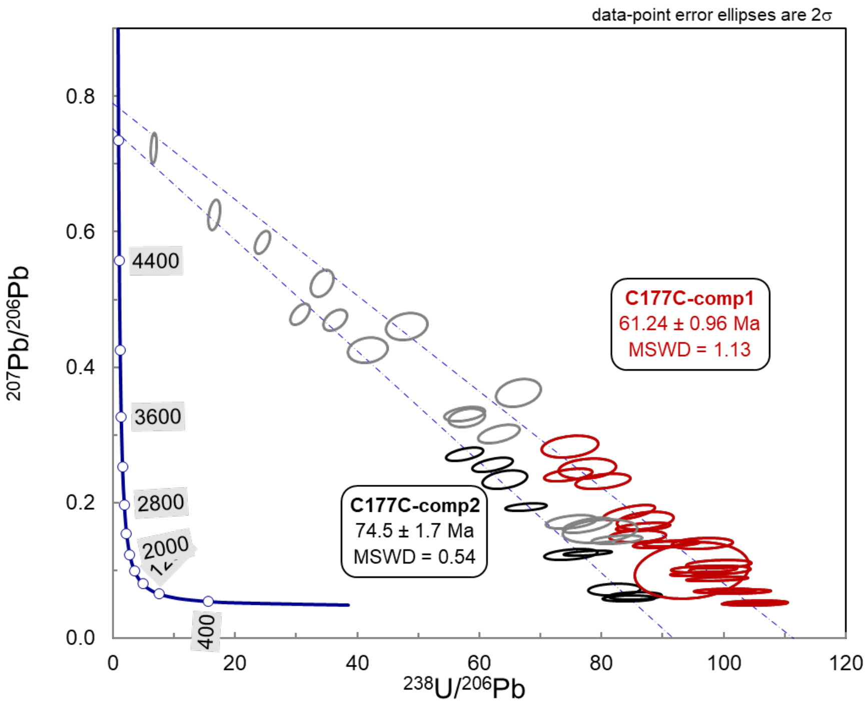

data-point error ellipses are 2σ
C177C-comp1
61.24 ± 0.96 Ma
MSWD = 1.13
C177C-comp2
74.5 ± 1.7 Ma
MSWD = 0.54
4400
3600
2800
2000
400
207Pb/206Pb
238U/206Pb
