# Supplementary material for: Pace of passive margin tectonism revealed by U-Pb dating of fracture-filling calcite
Source: Nat Commun. 2022 Apr 12;13:1953. doi: 10.1038/s41467-022-29680-z (PMC9005700; doi:10.1038/s41467-022-29680-z)

21a: Kingsland Bay Fault North (44.23895, -73.3078)

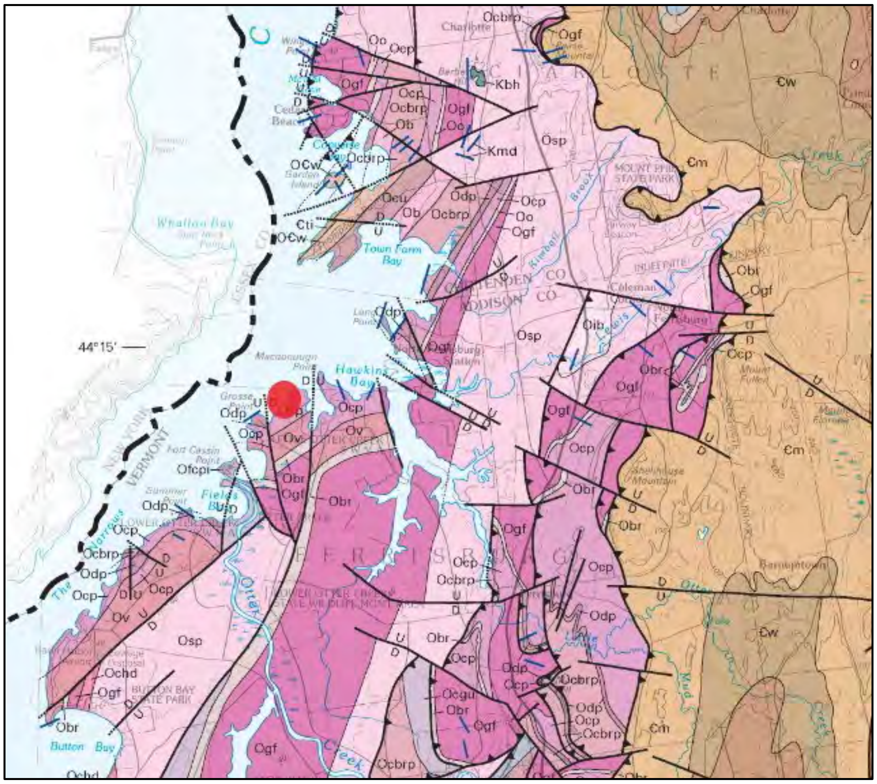

Figure 1: Sample location on state geologic map of Ratcliffe at al. (2011).

__Failed samples from same site:__ C271A

__Bedrock Unit:__ Crown Point Formation (Ocp)

__Ages__: **C271B**: 79 ± 4 Ma; **C271C**: 87.4 ± 1.2 Ma; **C271D**: 85.5 ± 1.8 Ma; **C271E**: 80.5 ± 2.2 Ma; **C271F**: 88.5 ± 1.8 Ma

Summary: This site is on the west shore of Kingsland bay peninsula and is easily reached by canoe or by parking at the Ferrisburgh Town Beach and following a walking trail to the west side of the peninsula. It is a spectacular example of a top to the east extensional fault zone, complete with abundant slickenfibres, calcite tabs, offset bedding, and drag folding (see overview photo below). All of these features suggest E-W extension between ~88 and 79 Ma. Dated features can be divided into two groups shown in figure 2 below: 1) tabular lenses of calcite within the vertical fault zone exposure bearing clear slickenfibres (C270B,C,D), and 2) sub-vertical mode 1 fractures exposed on the shore platform (C271E, F). Assuming that the vertical mode 1 fractures have σ_3 stresses perpendicular to the vein, then all of the dated features record σ_3 stresses of almost exactly 90° , presumably resulting in E-W extension.   At face value these results suggests this fault zone was active at multiple times in the Late Cretaceous and that the region may have been under an E-W σ_3 stress from ~88 to 79 Ma. U-Pb isochron ages are high quality, single-domain ages with only a few outlying laser shots. This combined with the massive nature of the calcite samples suggests the calcite ages reliably record activity on the sampled structures.

Field Observations:

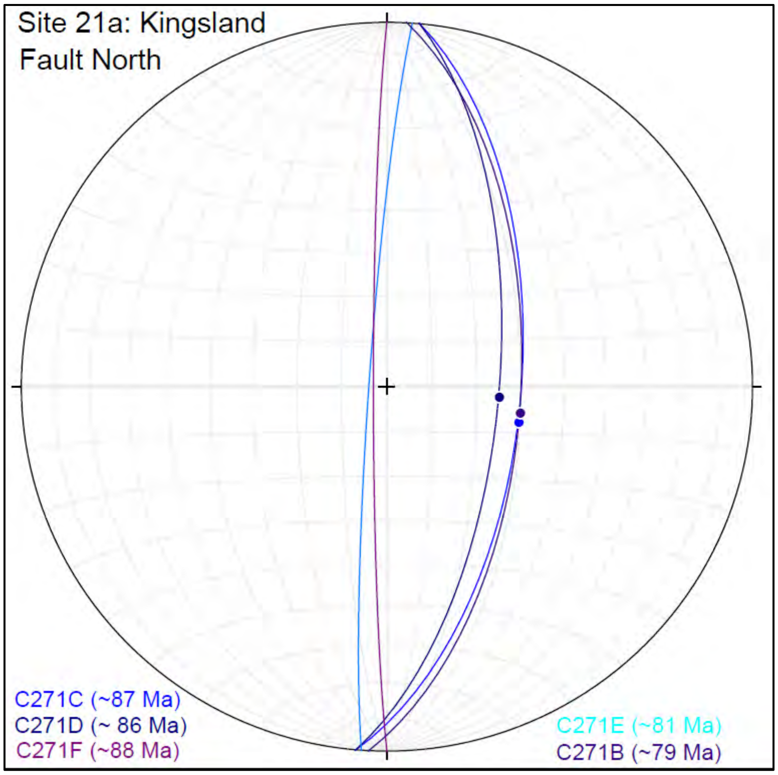

Figure 2: Stereonet showing orientation of dated structures at site 21a. Samples 271B,C, and D are calcite tabs taken from within the fault zone.

Overview Photo:

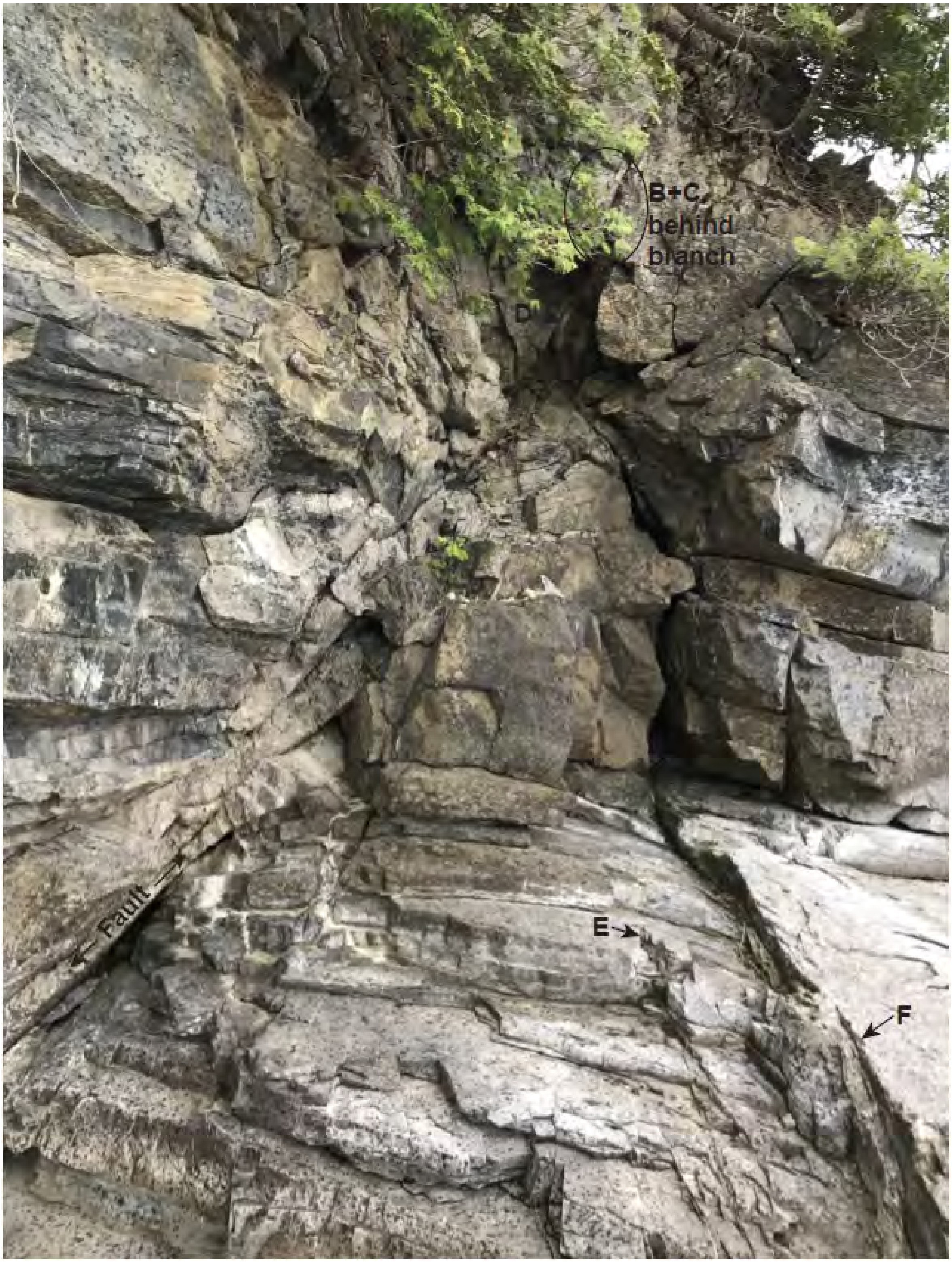

C271B:

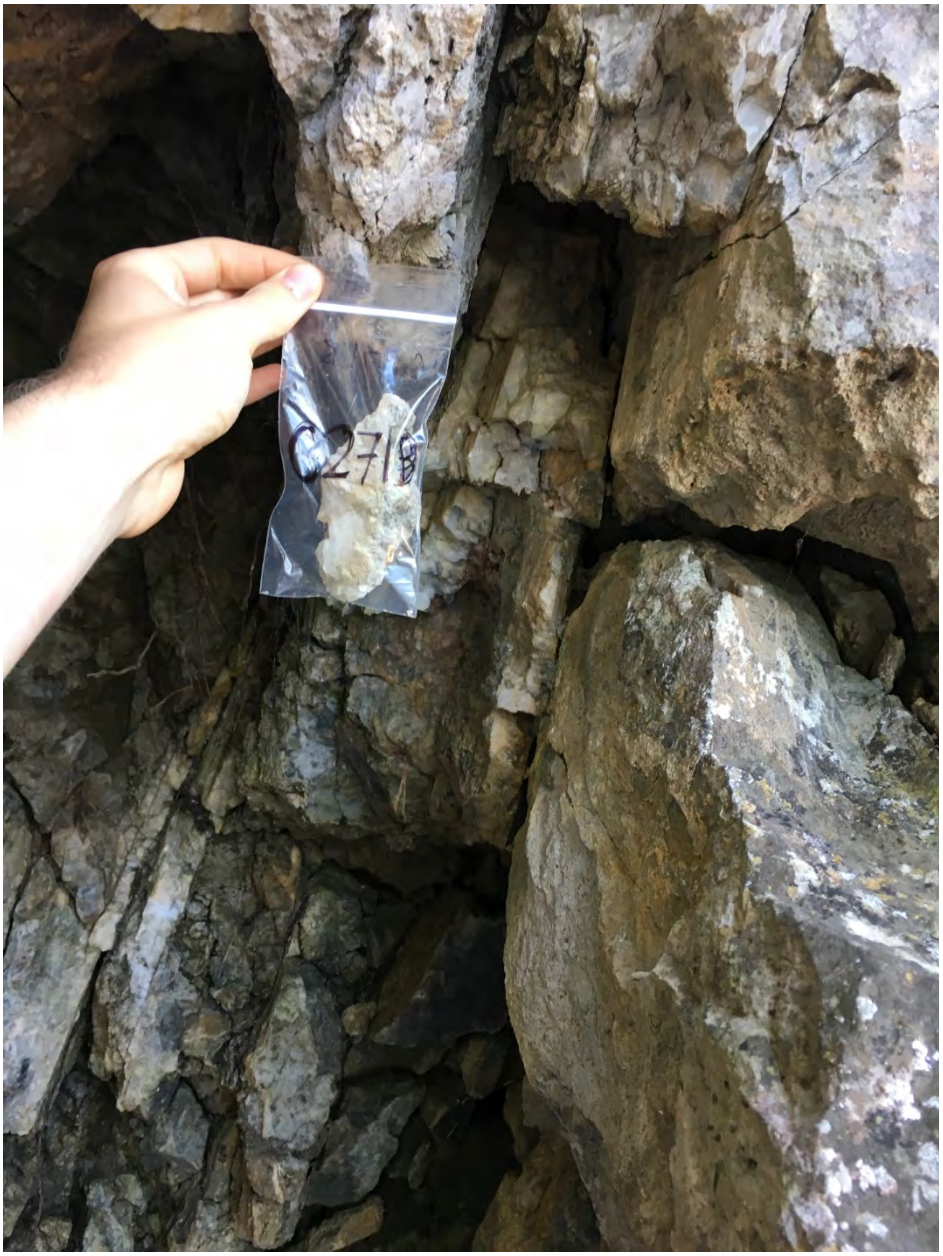

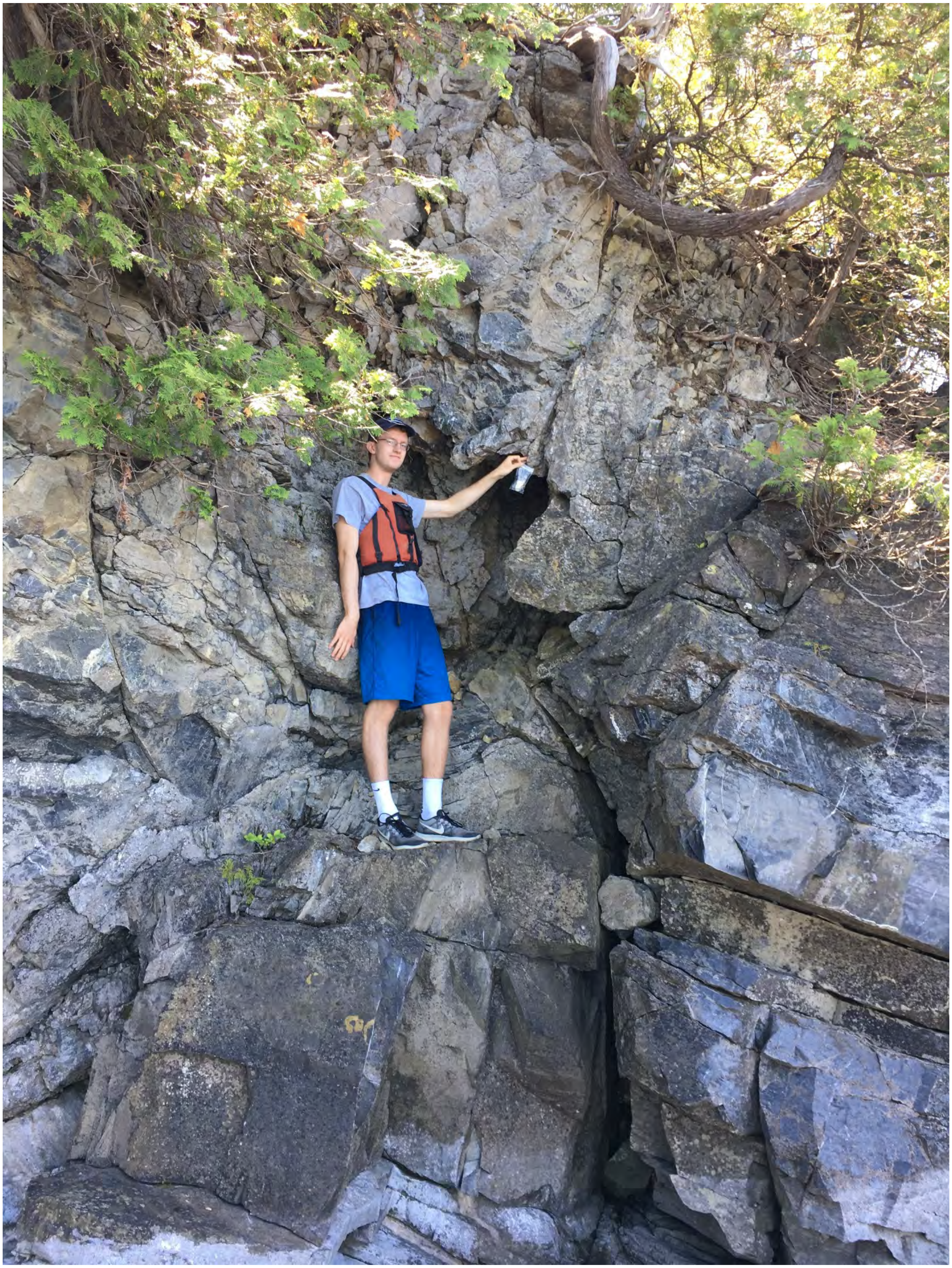

271C

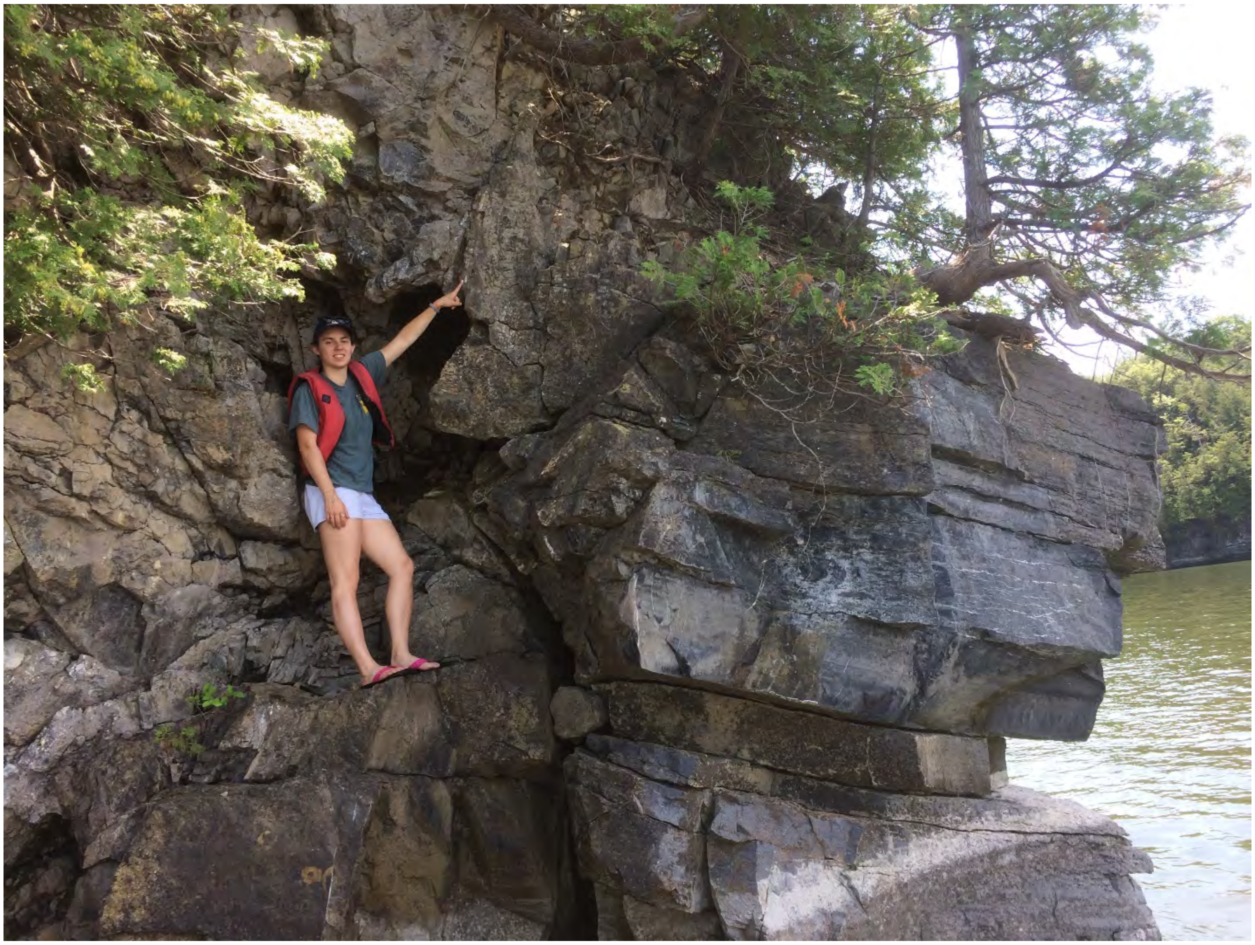

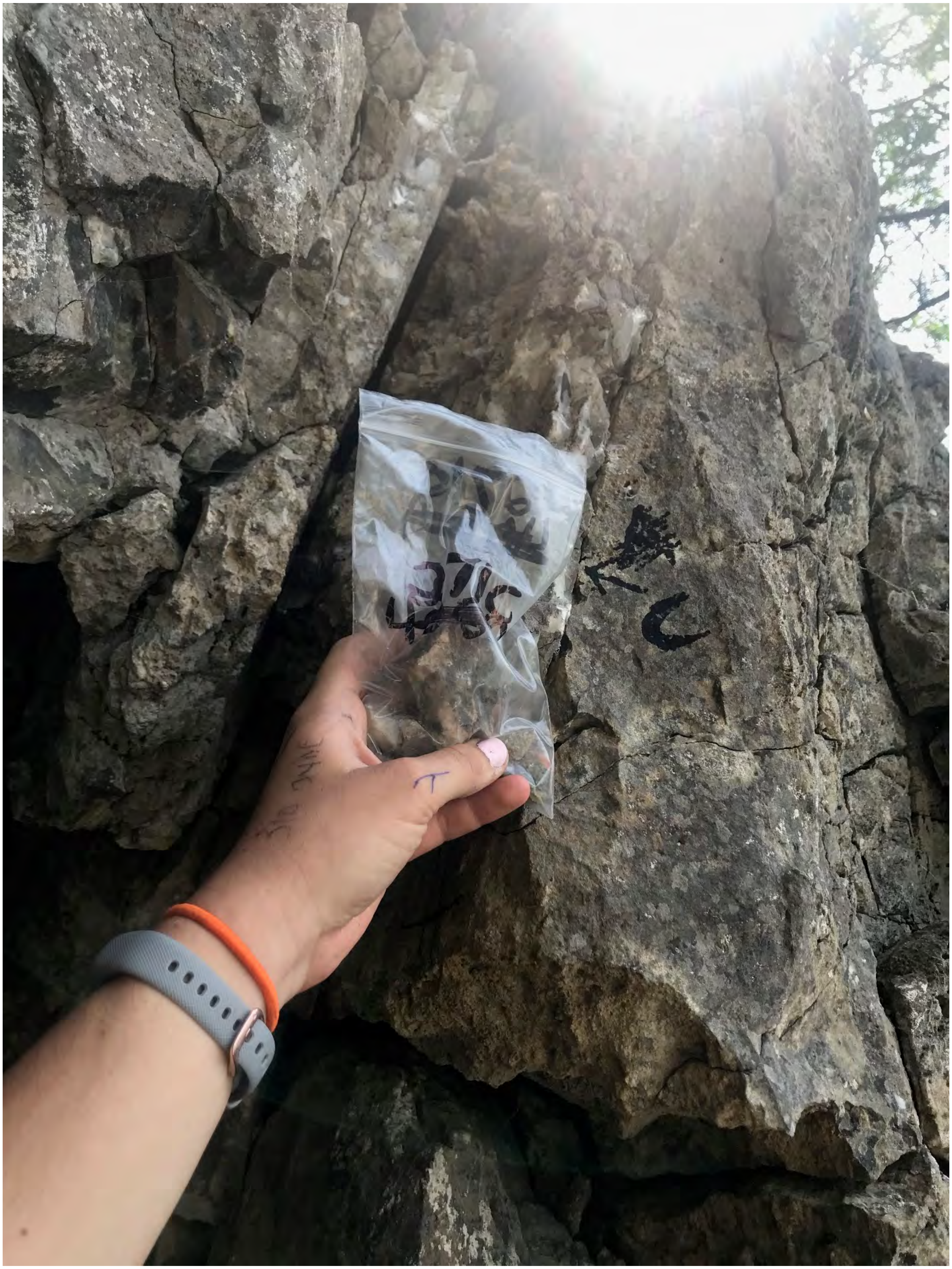

271D

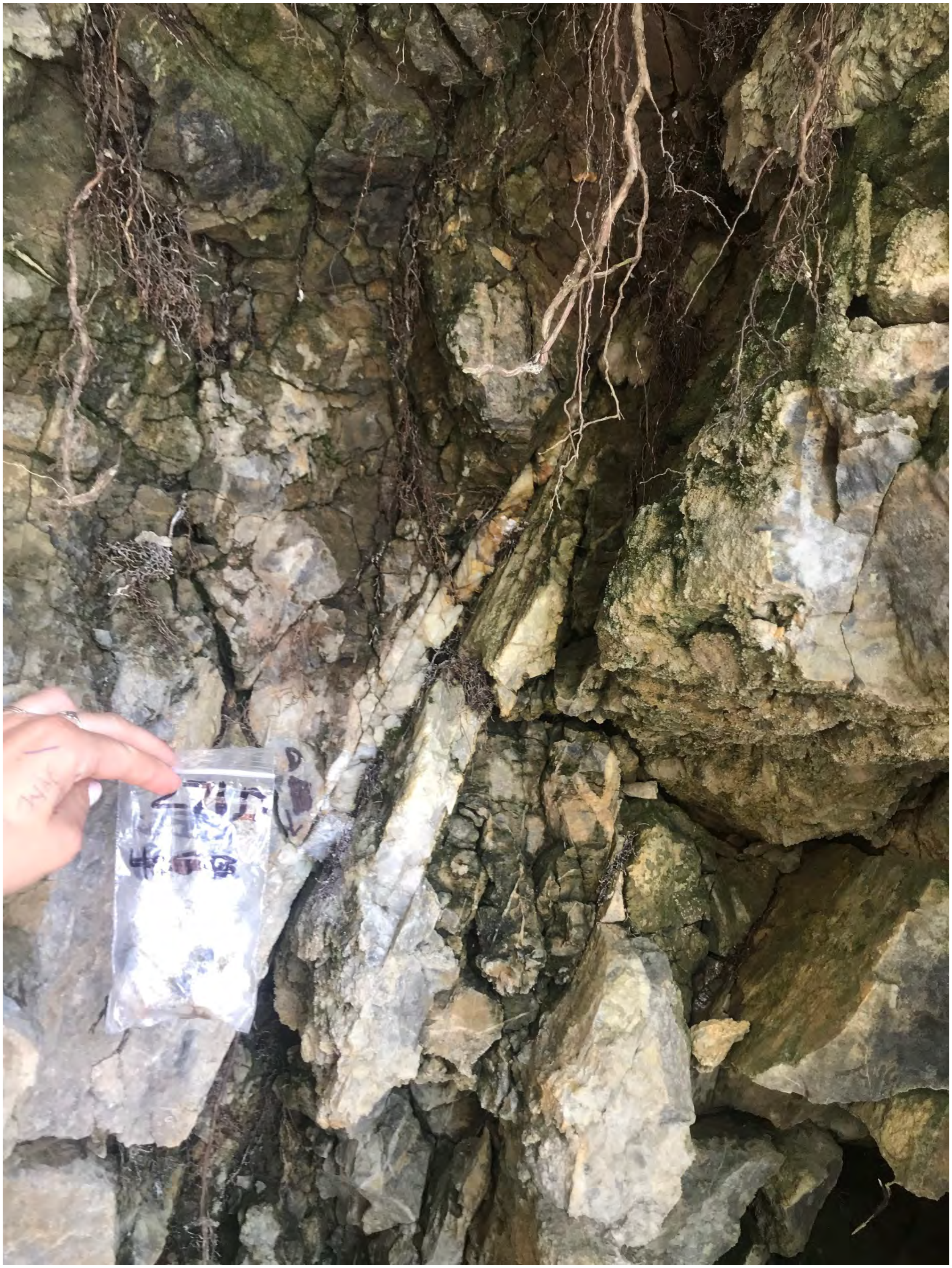

C271E:

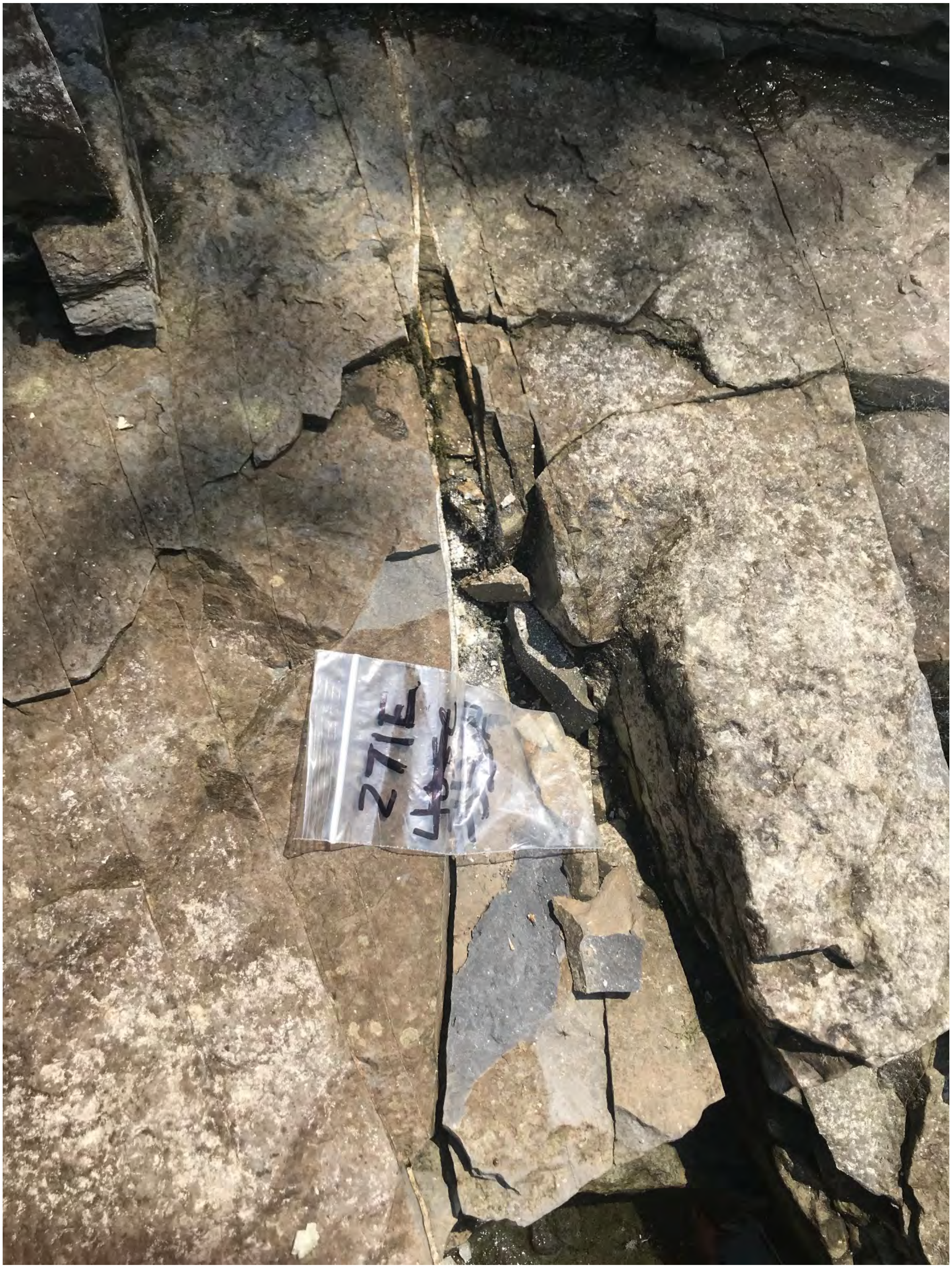

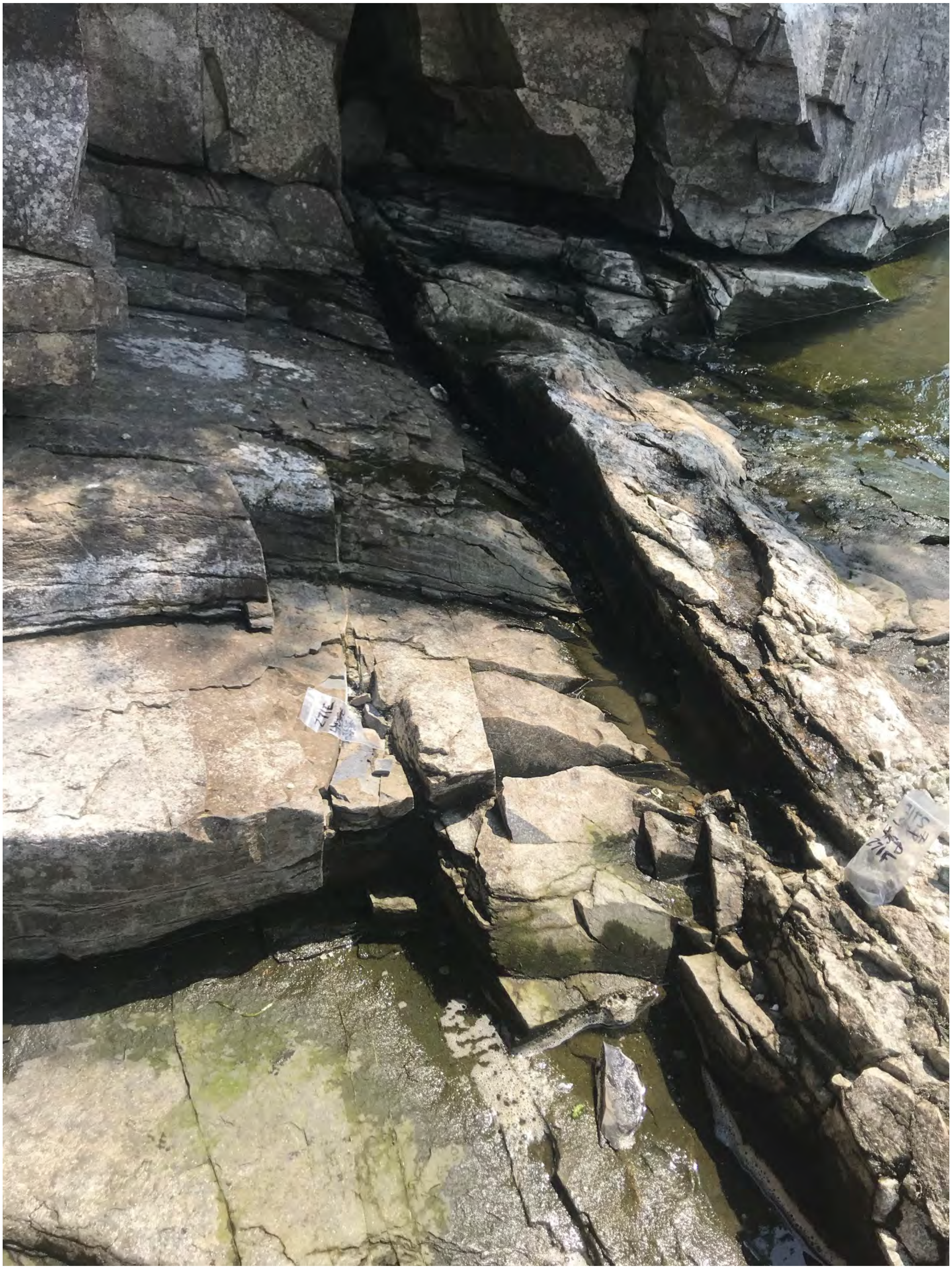

C271F :

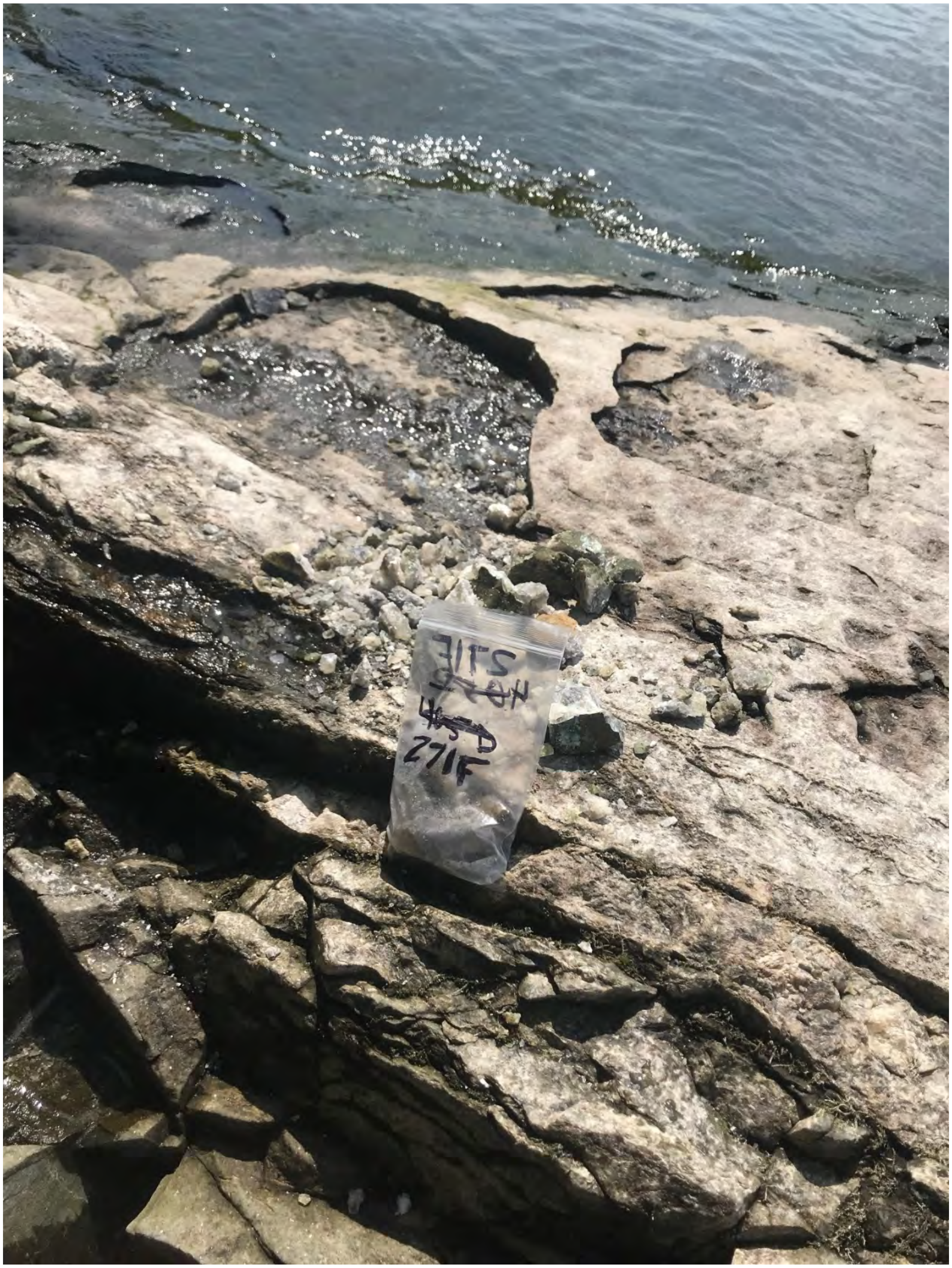

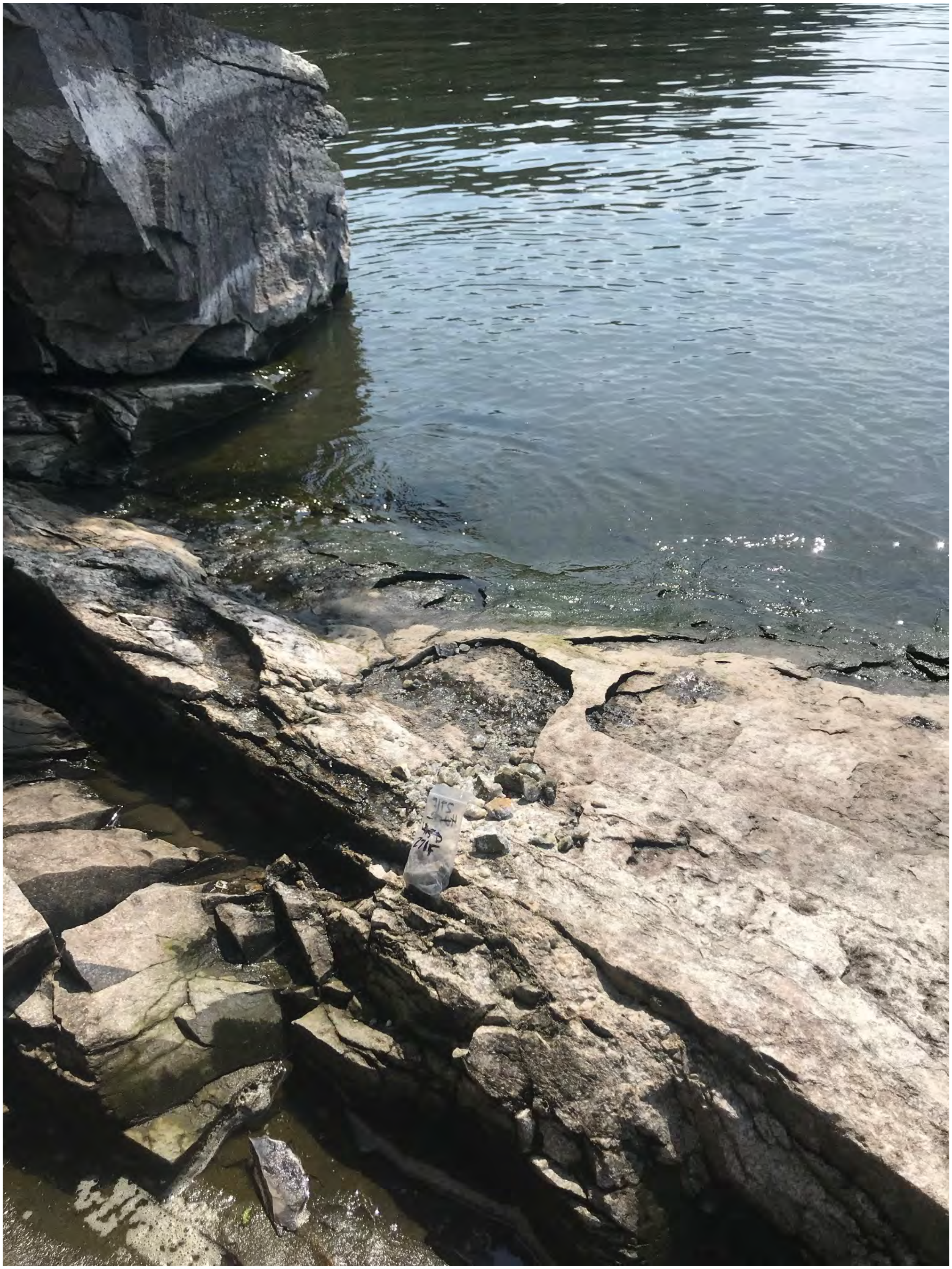

Lab Description:

C271B:

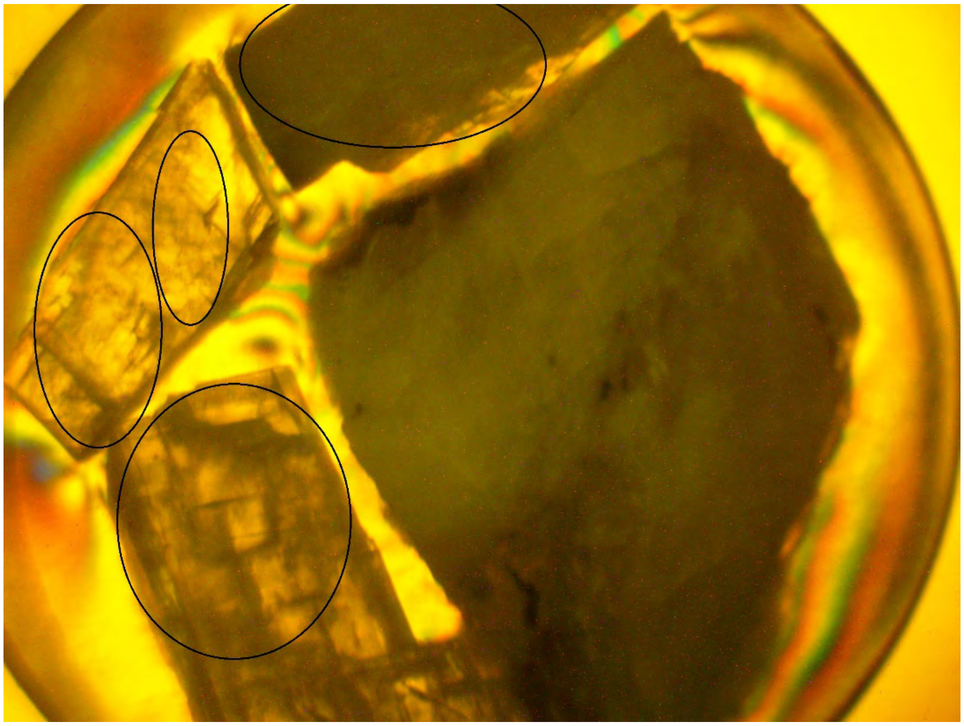

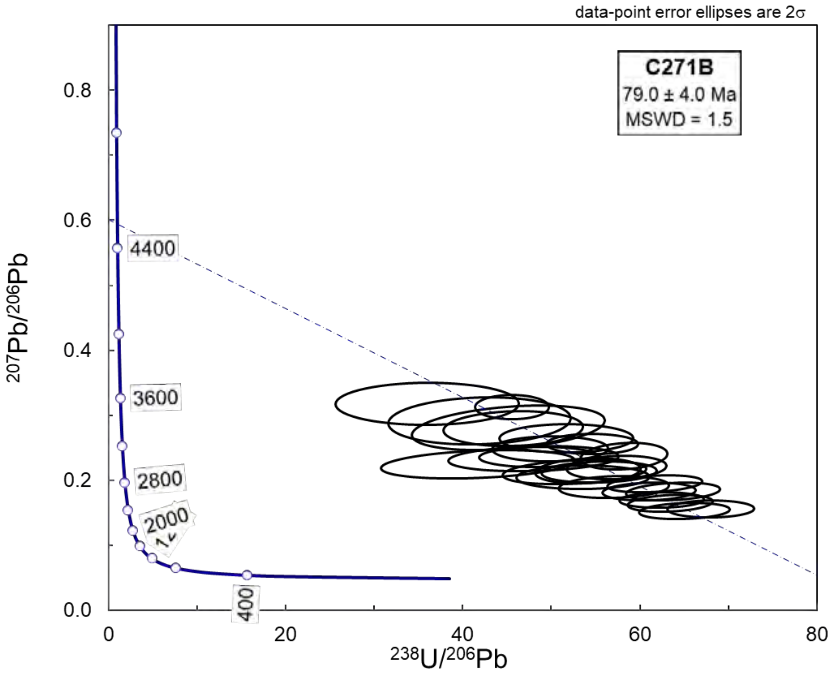

C271C: Circles in photo denote areas targeted for dating, not actual laser shots

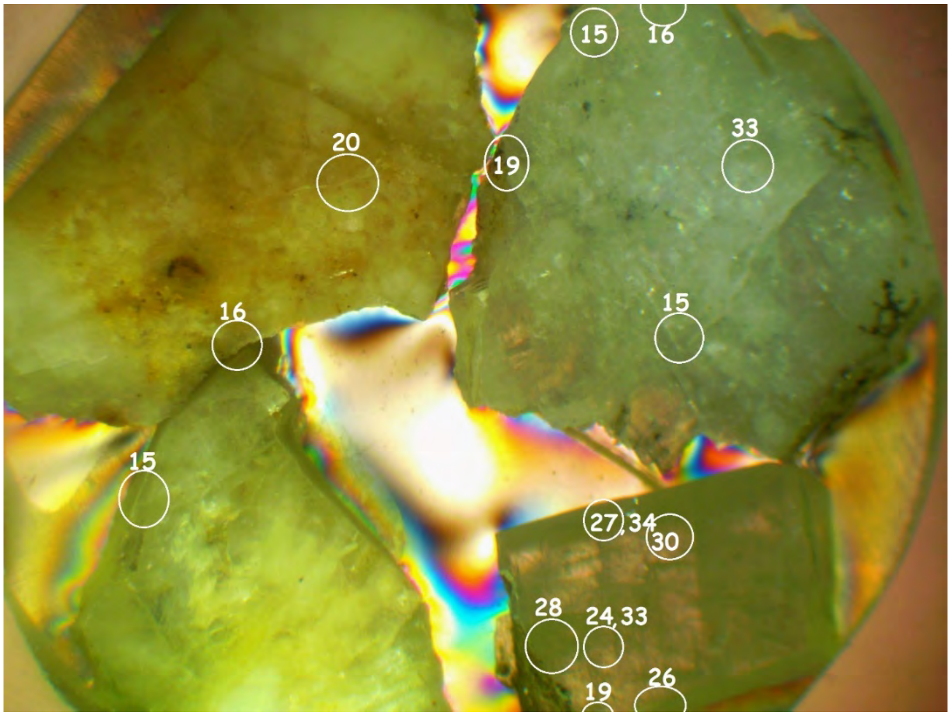

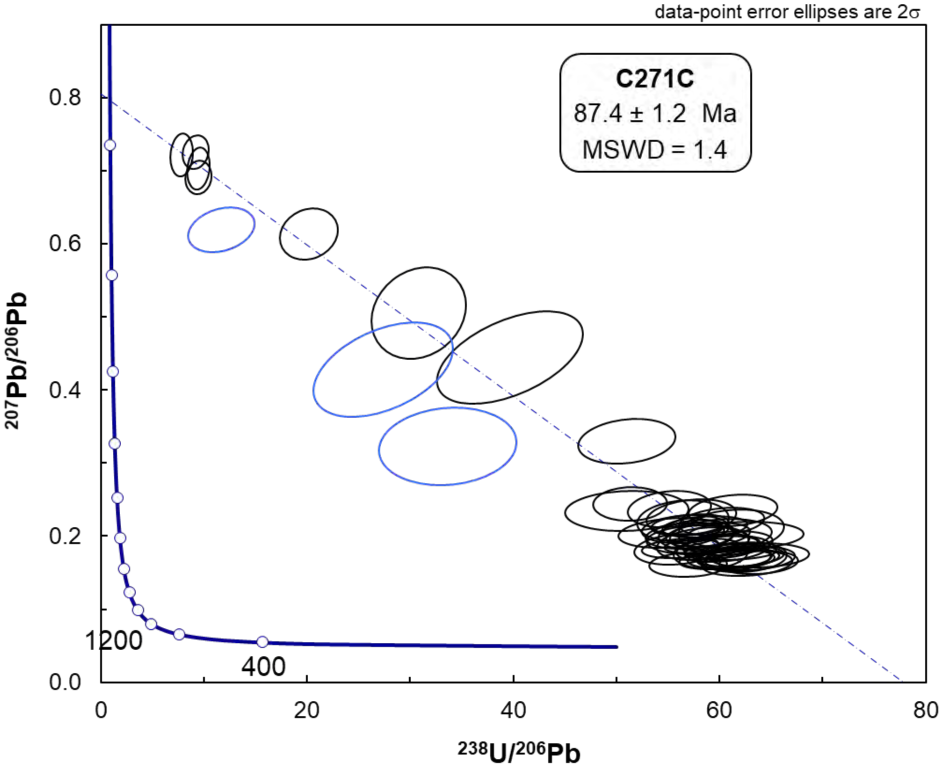

C271D: Circles in photo denote areas targeted for dating, not actual laser shots

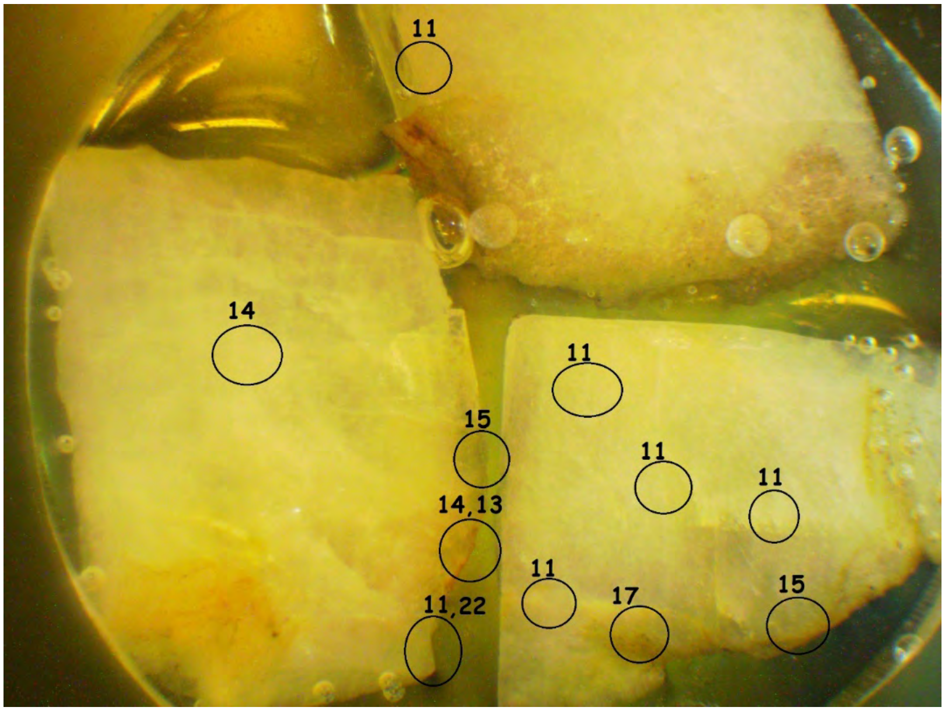

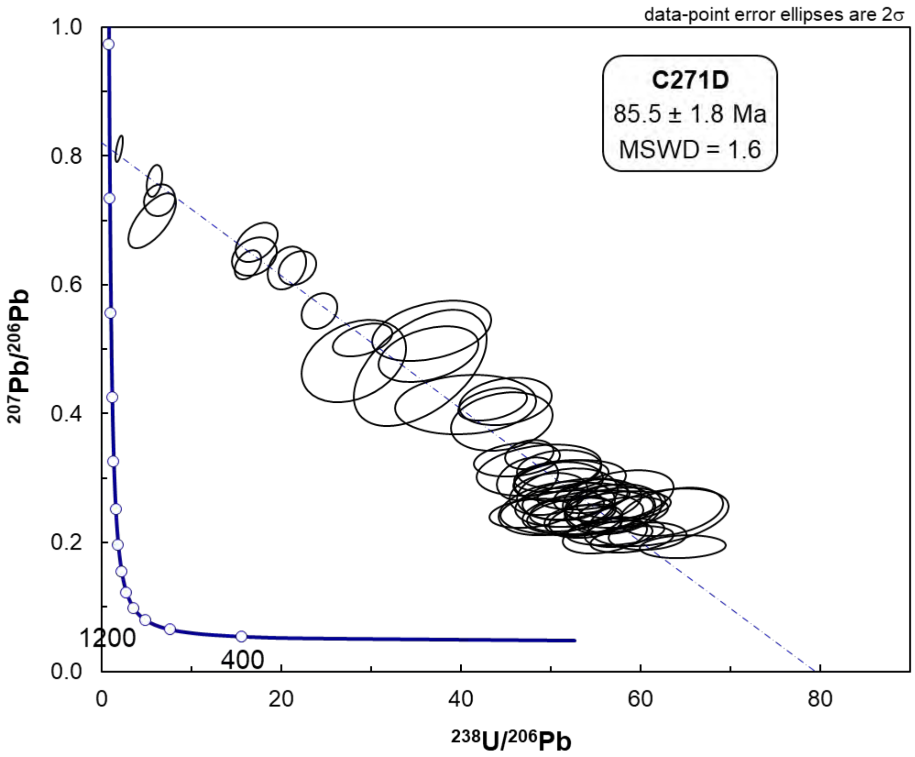

C271E: Circles in photo denote areas targeted for dating, not actual laser shots

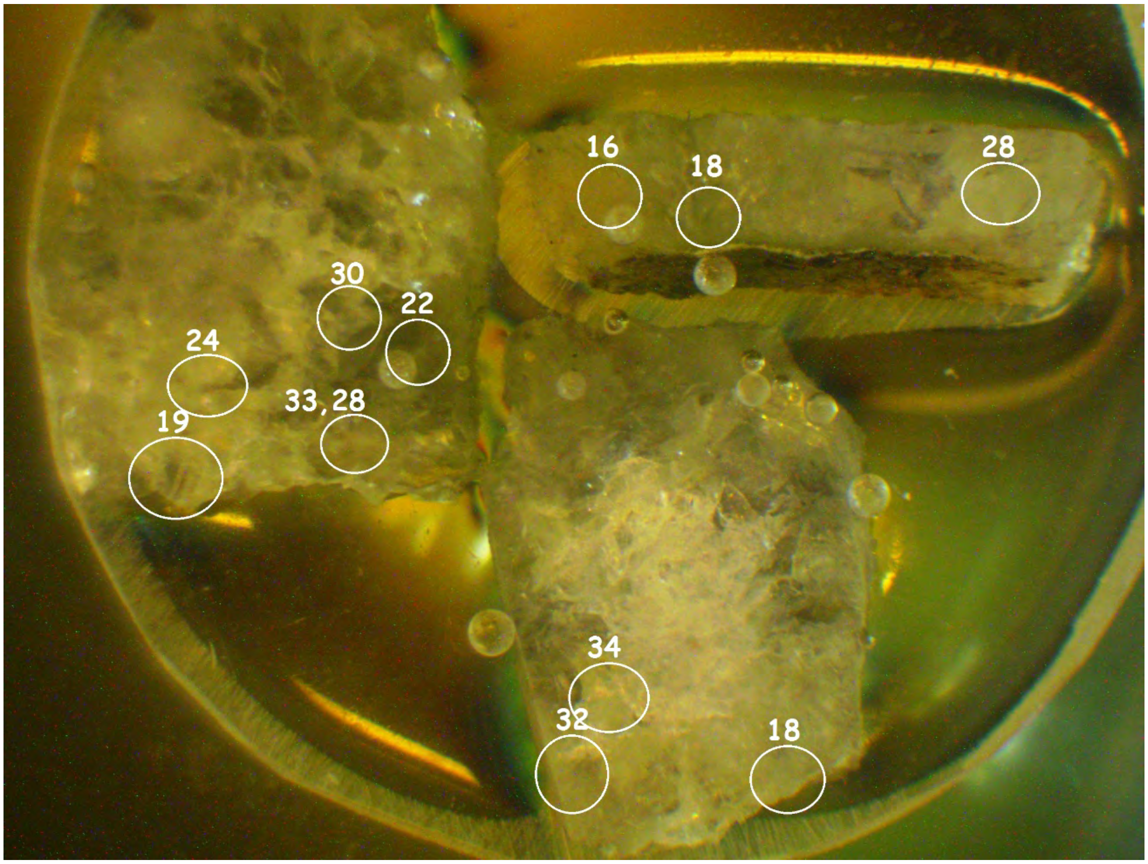

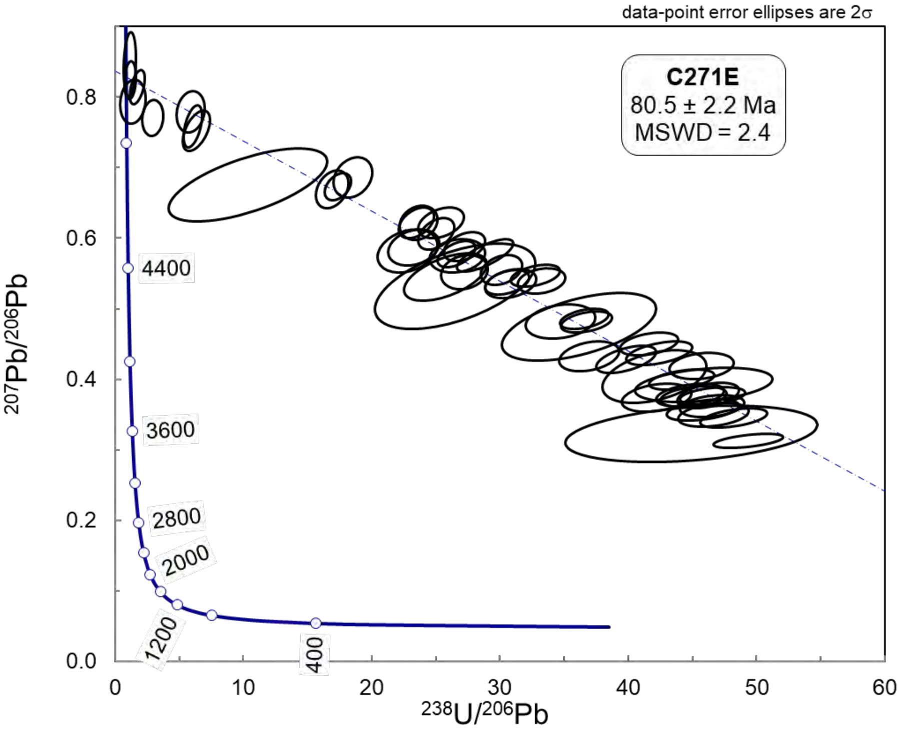

C271F: Circles in photo denote areas targeted for dating, not actual laser shots

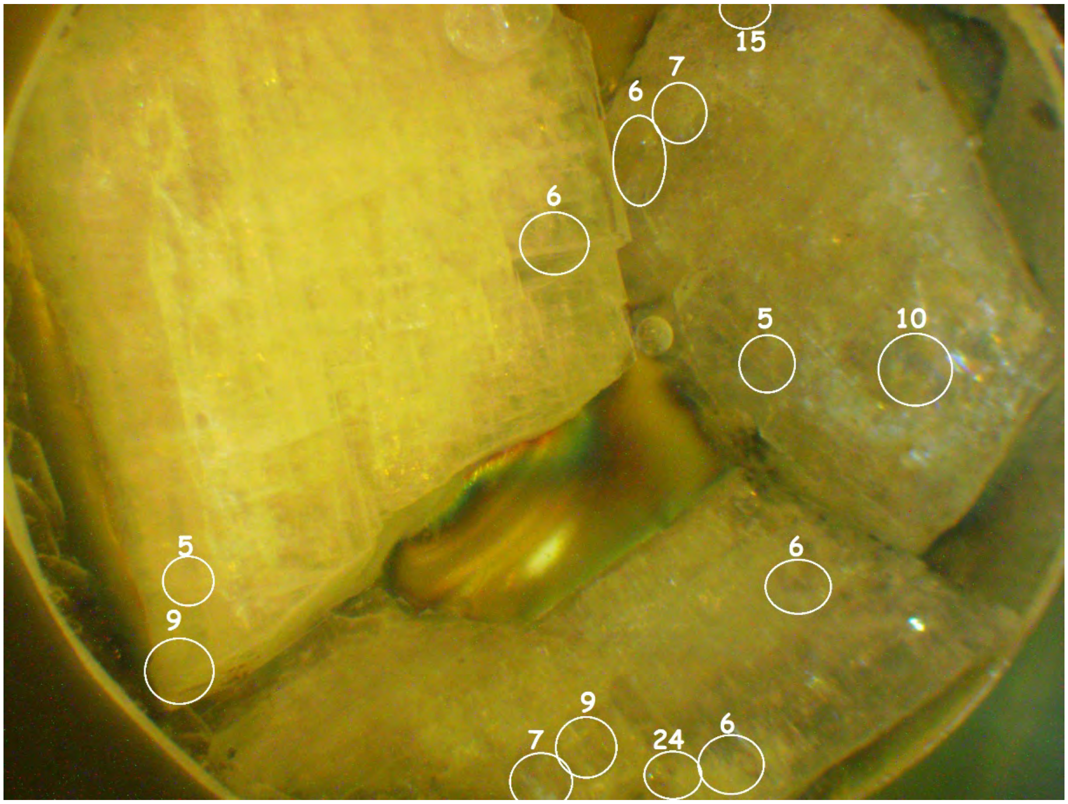

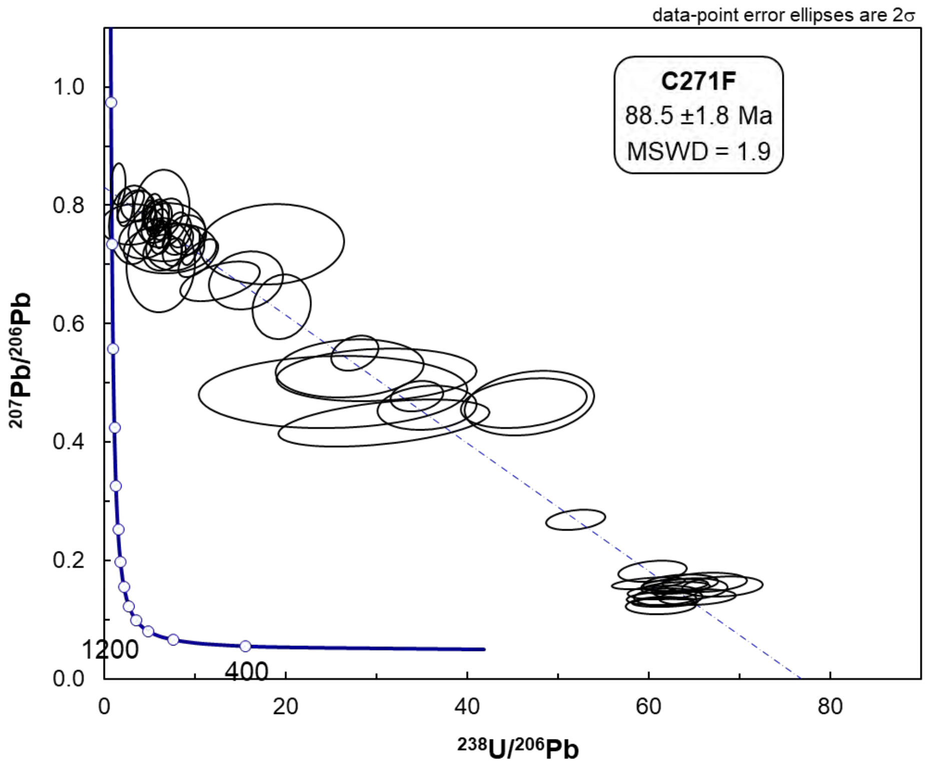

Supplement: Supplementary file 6 — Source Data [file 41467_2022_29680_MOESM6_ESM.zip › 2.zip!/appendix2b_site_summaries_21to50/21a_kingsland_bay_fault_north_final.pdf]
