# Supplementary material for: Pace of passive margin tectonism revealed by U-Pb dating of fracture-filling calcite
Source: Nat Commun. 2022 Apr 12;13:1953. doi: 10.1038/s41467-022-29680-z (PMC9005700; doi:10.1038/s41467-022-29680-z)

21B: Kingsland Bay Fault Central (44.238810, -73.307800)

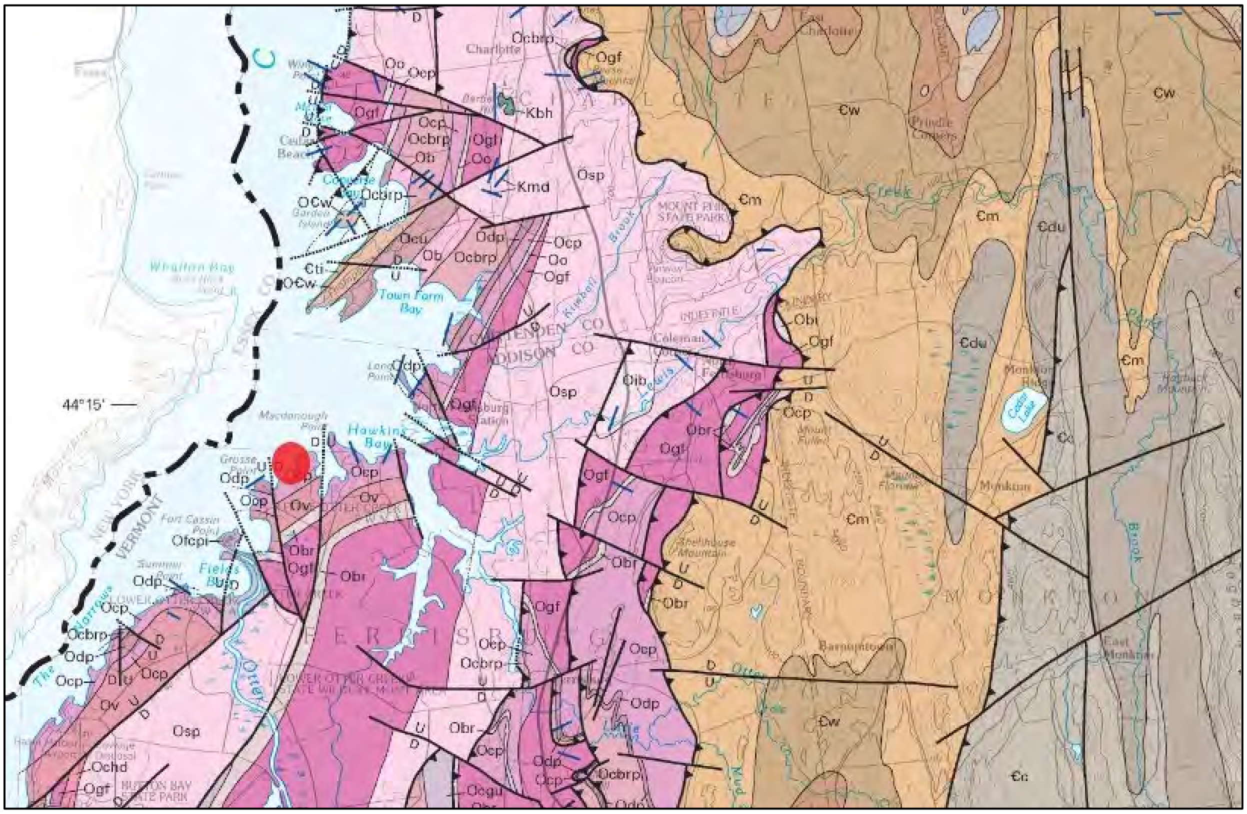

Figure 1: Sample location on state geologic map of Ratcliffe at al. (2011).

<u>Failed samples from same site:</u> C271A; C272B,C,D; C273B

<u>Bedrock Unit:</u> Crown Point Formation (Ocp)

<u>Ages</u>: C272A: 107 ± 6.5 Ma

<u>Summary</u>: This site is located between sites 21a and c on a small protrusion of bedrock at an elevation near 99' asl. Can be reached in same manner as those sites. It is characterized by an anastomosing network of veins which are 1-3 cm wide, non-planar and intersect each other frequently. Sampled vein strikes 0° and dips 60° to the east, suggesting these veins may have been intruded under and roughly E-W oriented $\sigma 3$ stress. That said, the anastomosing style of the vain precludes reliable stress interpretation and is thus categorized as unknown. The anastomosing texture of these veins and their non-planar character strongly suggest they were intruded by hot fluids seeking a path upwards rather than by opening of a fracture. For this reason, this is the 'type section' of the hydrothermal 'vein swarm' style. Interesting, another very similar swarm of calcite veins can be observed just to the north at site 22 (C270A,C), perhaps suggesting a unique structural property or fluid pathway that lent itself to formation of these non-planar vein deposits.

The U-Pb age is of high quality with uniform calcite texture and no outliers discarded, suggesting it is a reliable indicator the timing of vein emplacement.

<u>Field Observations</u>:

C272A:

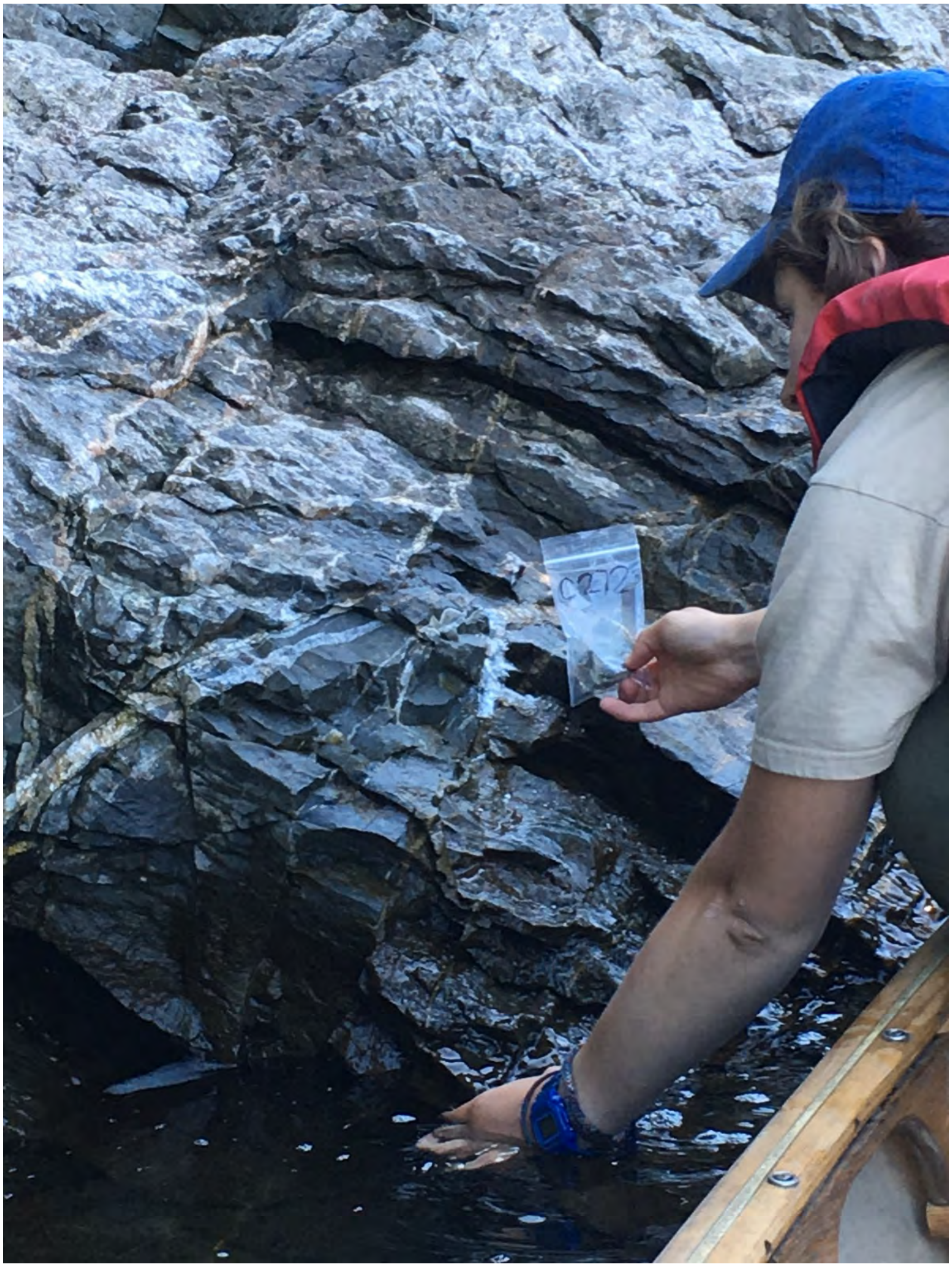

Lab Description:

C272A:

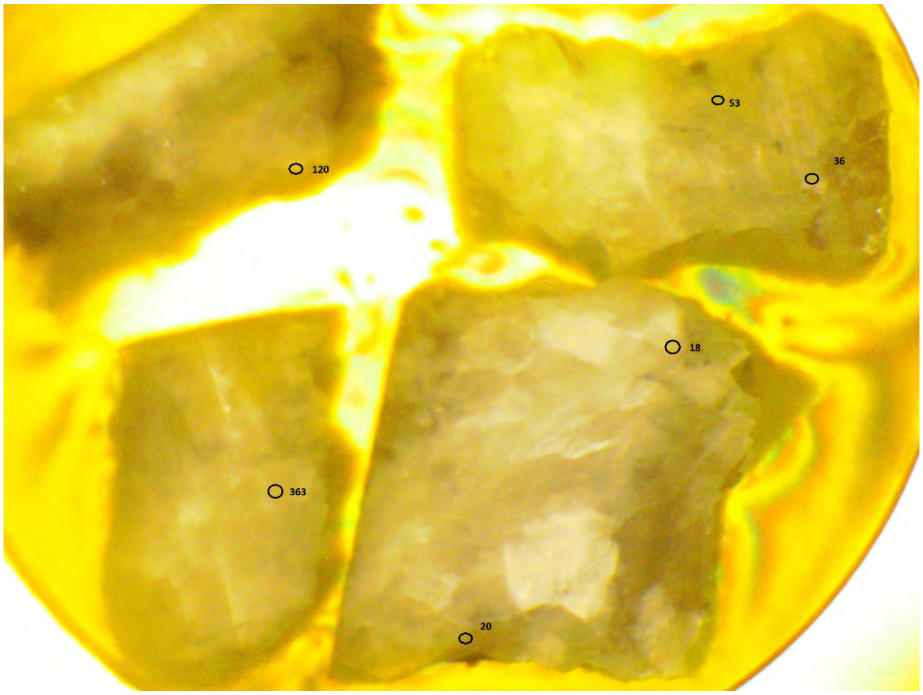

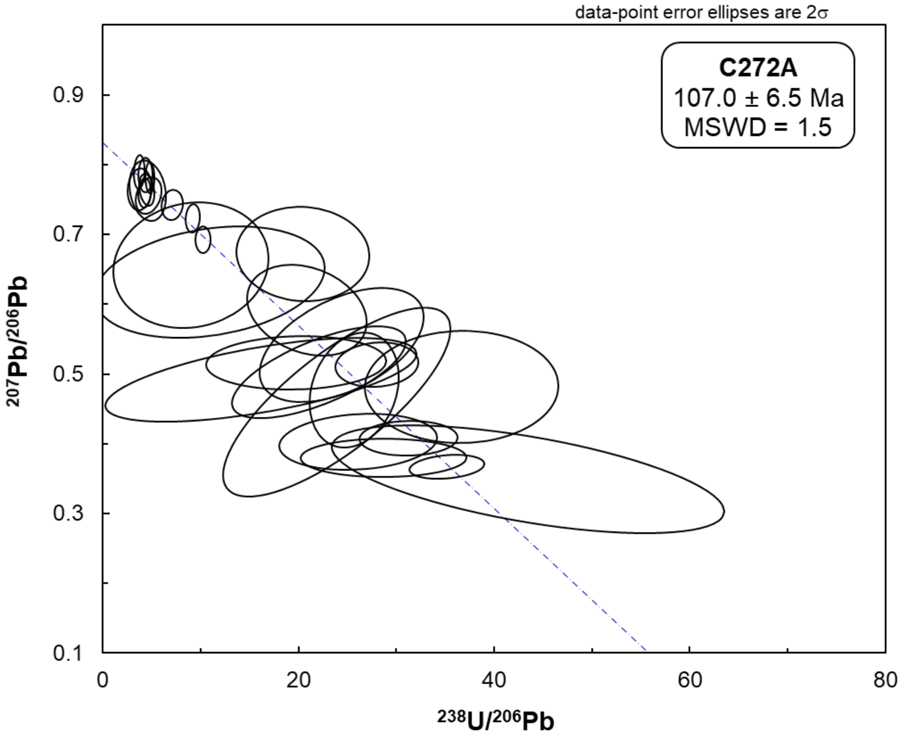

Supplement: Supplementary file 6 — Source Data [file 41467_2022_29680_MOESM6_ESM.zip › 2.zip!/appendix2b_site_summaries_21to50/21b_kingsland_fault_central_final.pdf]
