# Supplementary material for: Pace of passive margin tectonism revealed by U-Pb dating of fracture-filling calcite
Source: Nat Commun. 2022 Apr 12;13:1953. doi: 10.1038/s41467-022-29680-z (PMC9005700; doi:10.1038/s41467-022-29680-z)

21C: Lake Champlain West Side Kingsland Site (44.23850, -73.30790)

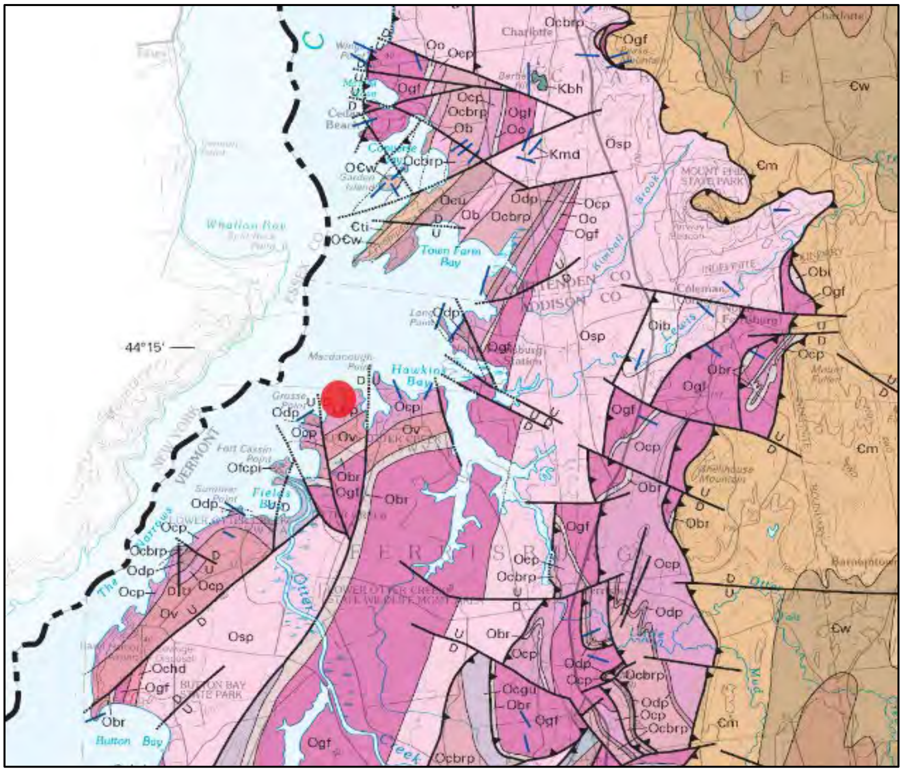

Figure 1: Sample location on state geologic map of Ratcliffe at al. (2011).

<u>Failed samples from same site:</u> C271A; C272B; C273B

<u>Bedrock Unit:</u> Crown Point Formation (Ocp)

<u>Ages</u>: **C273A**: 129 ± 18*; **C273C**: 83 ± 2; **C273D**: 108 ± 3; **C273E**: 85.8 ± 1; **C273F**: 95.7 ± 3; **C273G**: 107.7±3.9; **C273H**: 86 ± 2 **only ~15 shots, low quality age*

Summary: This is the southern extension of the extensional fault zone described at site 21a and can be accessed by boat or by foot from the south at low water. One can look down-dip along the fault zone where it is exposed along a shore platform, and can then look along strike where it is exposed in a roughly 5 m high cliff exposure. Moving south towards the cliff exposure, the fault zone steepens and bends eastward, transitioning from a N-S orientation to a NW-SE orientation. This change in orientation has created a complex slip history within the fault, where an inability of the sub-vertical fault zone to accommodate lateral motion documented at site 21a has created an abrupt widening of the breccia zone about 3 m up the vertical exposure (see overview photo below).

Dated samples from this site can be divided into four groups: 1) samples from within the NW-SE trending part of the fault zone (C273A,C,E), 2) a single sample from within N-S trending part of the fault zone (C273H), 3) a single sub-vertical mode 1 fracture on the left side of the fault zone (C273F), and 4) a set of sub-vertical mode 1 fractures on the right side of the fault zone, some of which are associated with en-echelon fractures (C273D,F,G).

These veins record at least four events. First, sample C273A is a mixed-mode fracture with top to the southeast slickenfibres interpreted to record NW-SE extension at ~ 129 +/- 18 Ma. The more southerly NW-SE oriented part of the fault zone experienced high-angle slip in a nearly down-dip sense, where the σ_3 stress had a trend/plunge of 57°/13° and the σ_1 stress was 194°/54°. This is one of the oldest dated structures in the study area and its tectonic significance is unclear.

Second, samples C273D and G are from mode 1 fractures interpreted to open under E-W oriented σ_3 stresses at ~ 108 Ma. Third, sample C273F is from a mode 1 fracture interpreted to open under ENE-WSW oriented σ_3 stresses at ~ 95 Ma. Finally, sample H is a mixed mode (mode 2) fracture with top to the east slickenfibres recording E-W extensional slip on the fault zone at ~86 Ma, where the σ_3 stress had a trend/plunge of 99°/1° and the σ_1 stress was nearly vertical at 347°/84°. This last period is supported by the ~86 Ma age of sample C273E collected from a massive spar within the fault zone.

U-Pb ages from this site are very high quality defining single-domain isochrons with tight clustering and high U/Pb ratios. The one exception is sample C273A, which has fewer shots defining a more scattered isochron. Texturally the calcite is massive and homogenous, suggesting it is primary and its age directly records formation of or slip on the relevant structures

Field Observations:

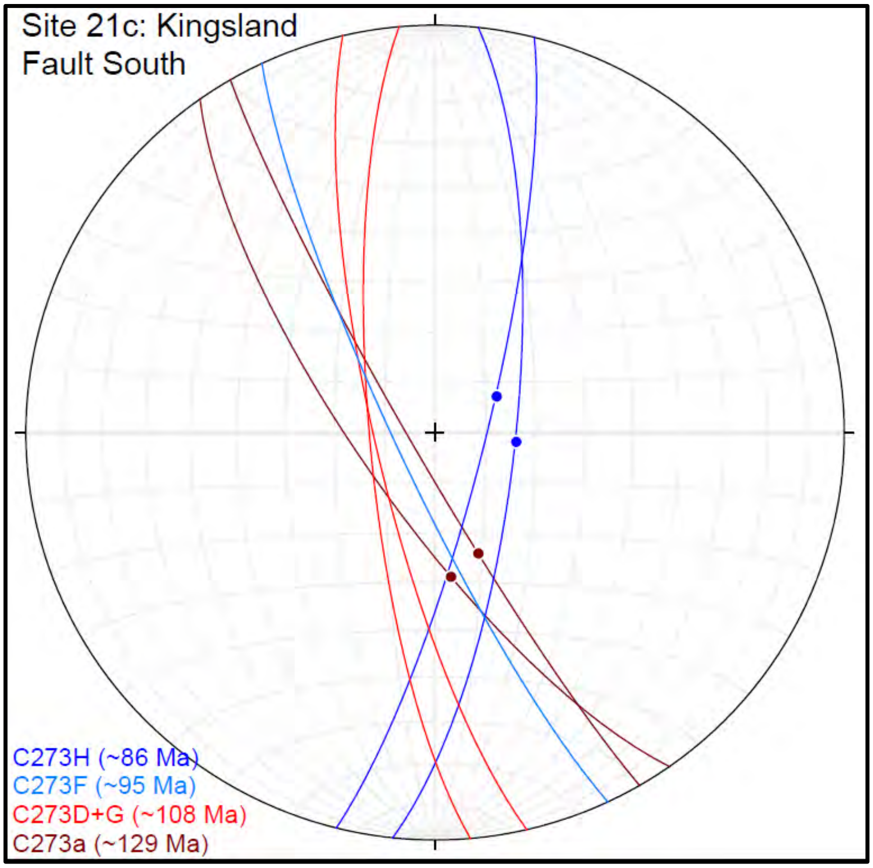

Figure 2: Stereonet plot showing orientation of dated structures at site 21c

Overview Photo:

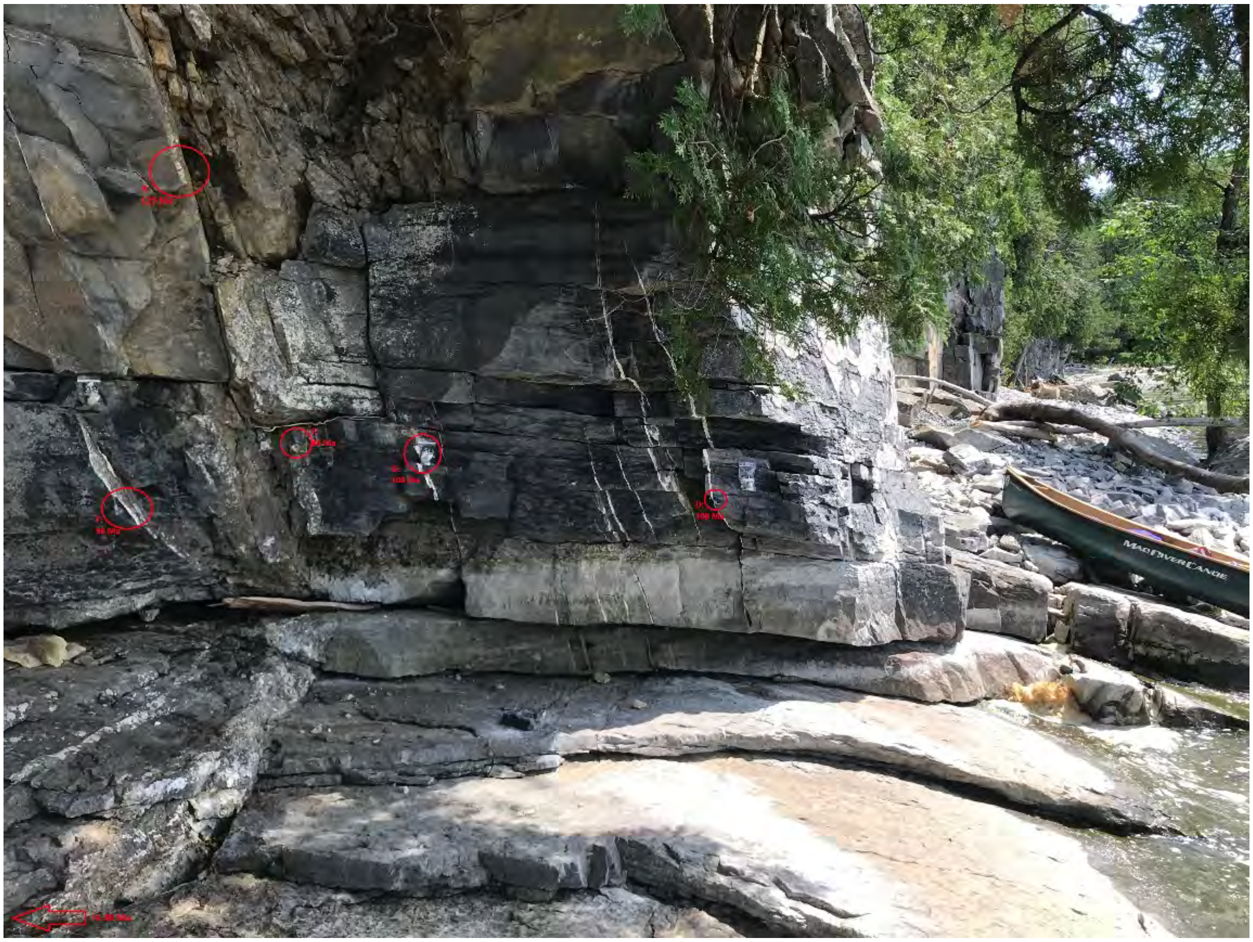

C273A: Slip surface on LHS of fault zone, weathered to brown with angular, needle-like calcite crystals

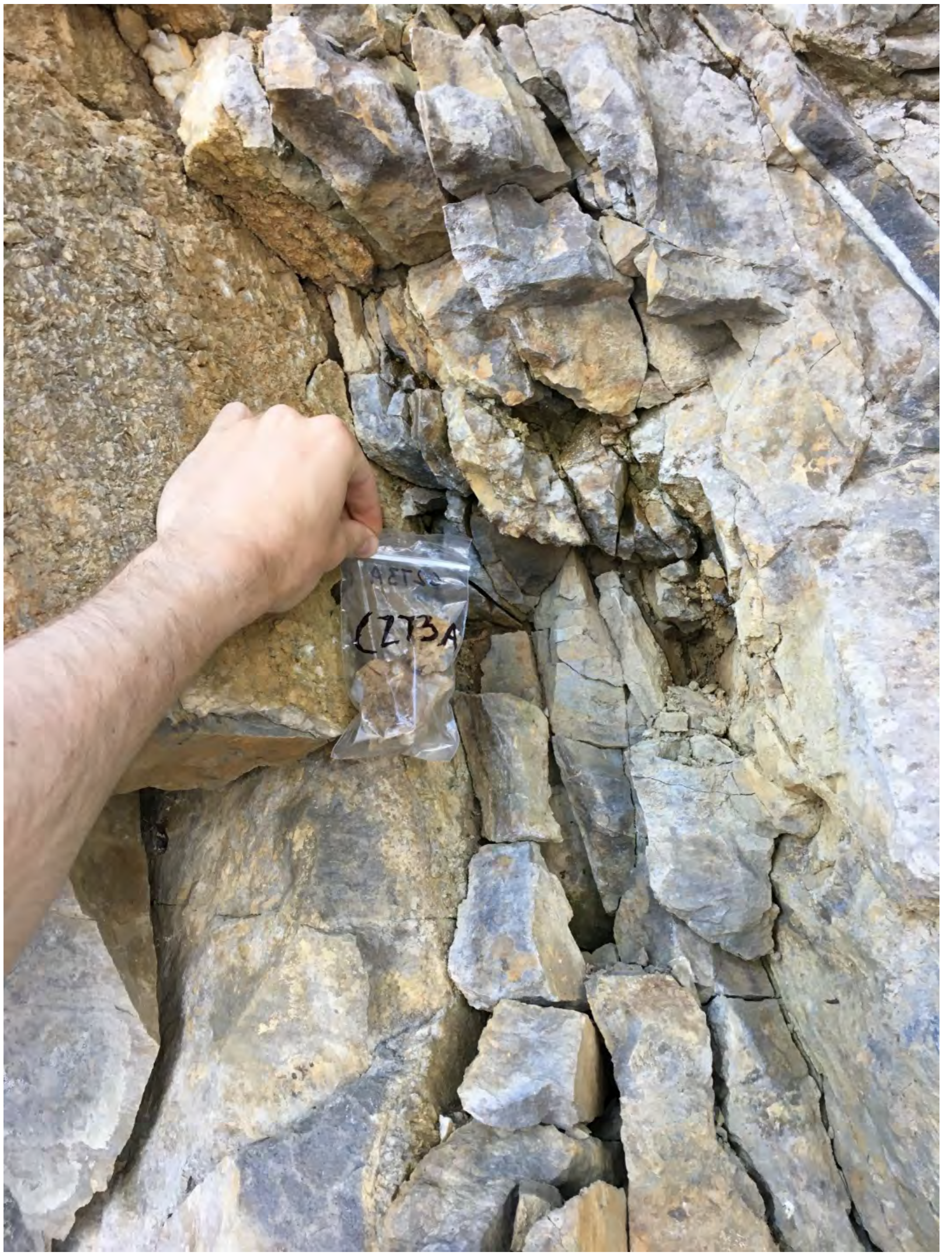
C273A

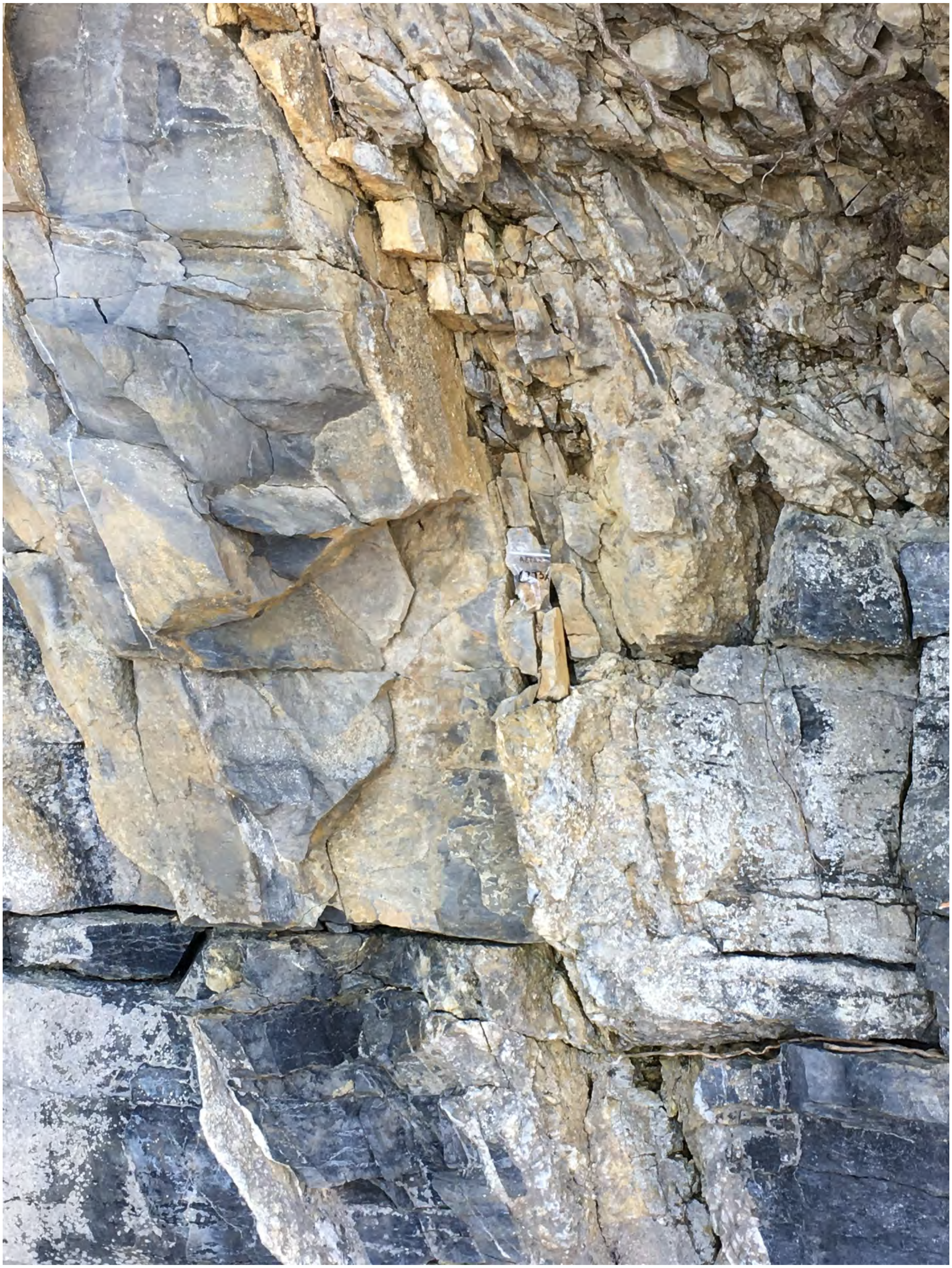

C273C:

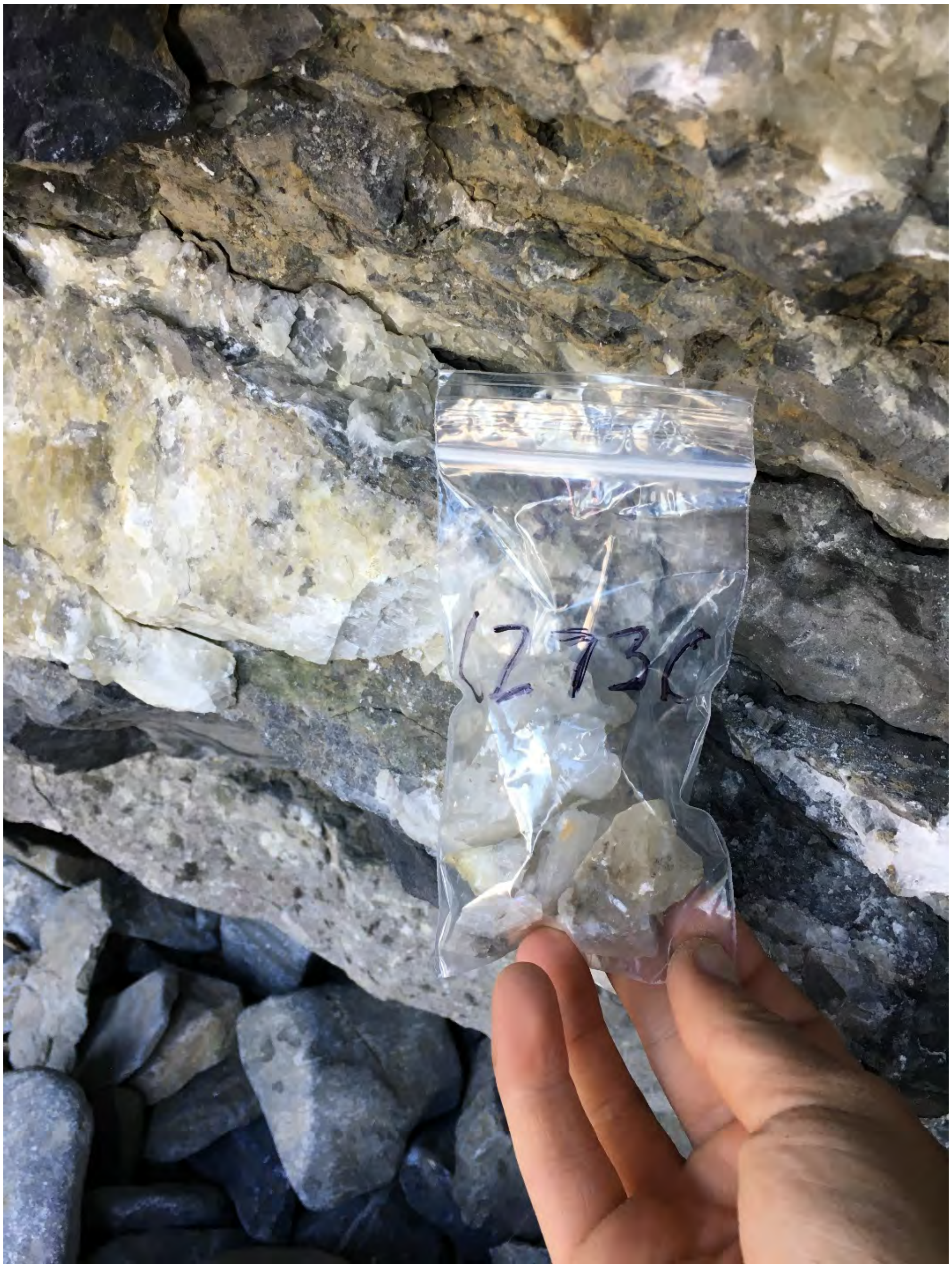
12730

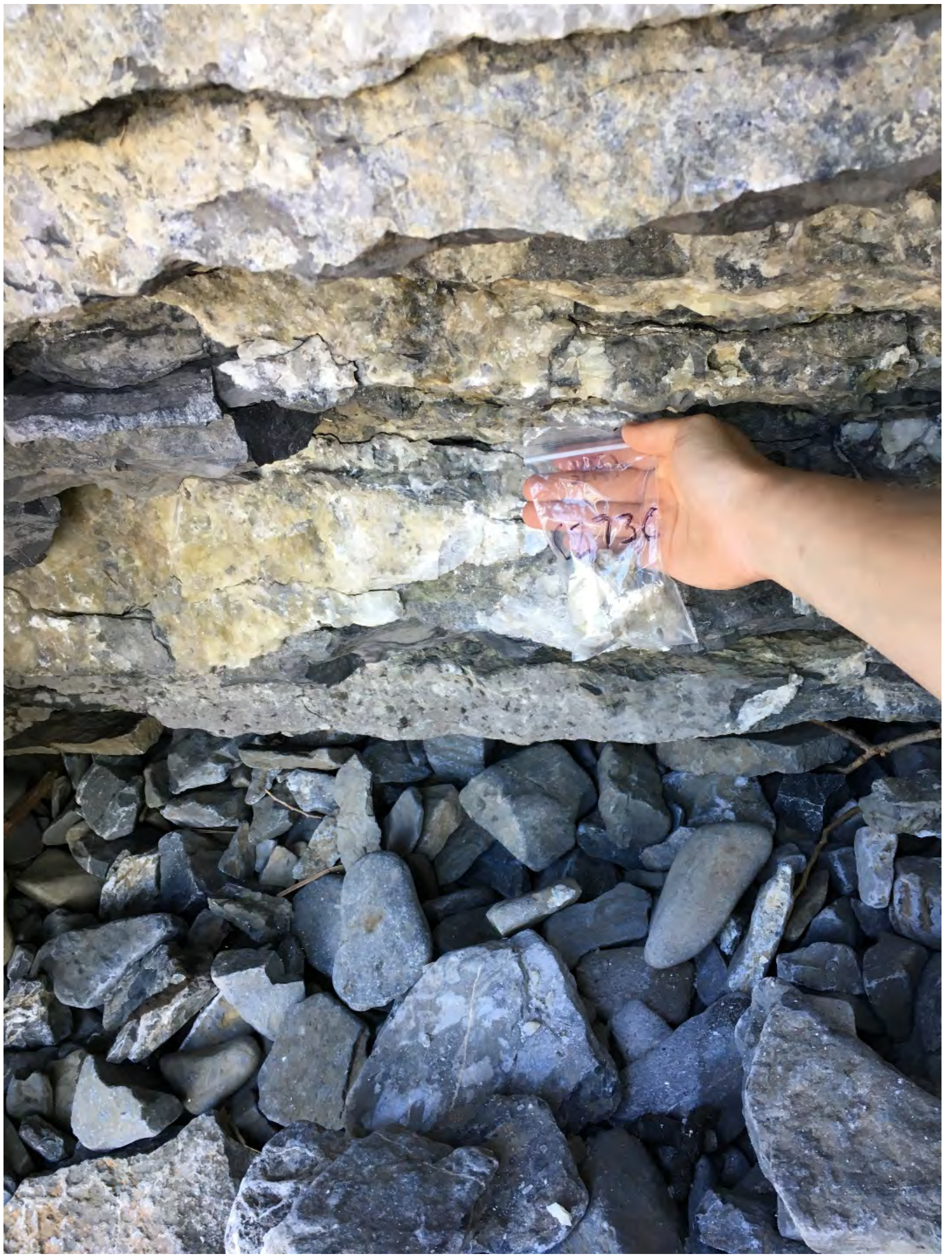

C273D: sub-vertical planar fracture to right of FZ

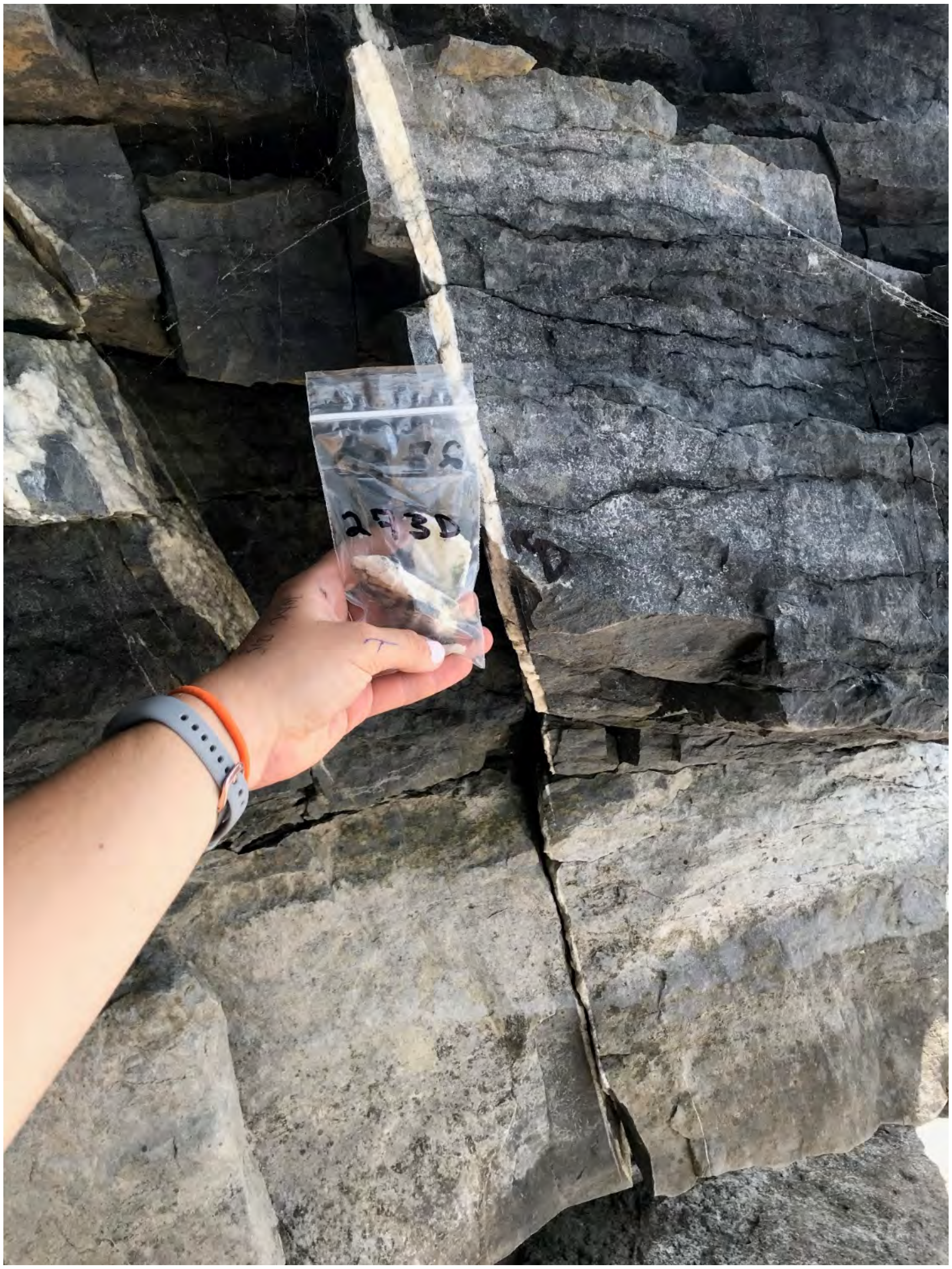

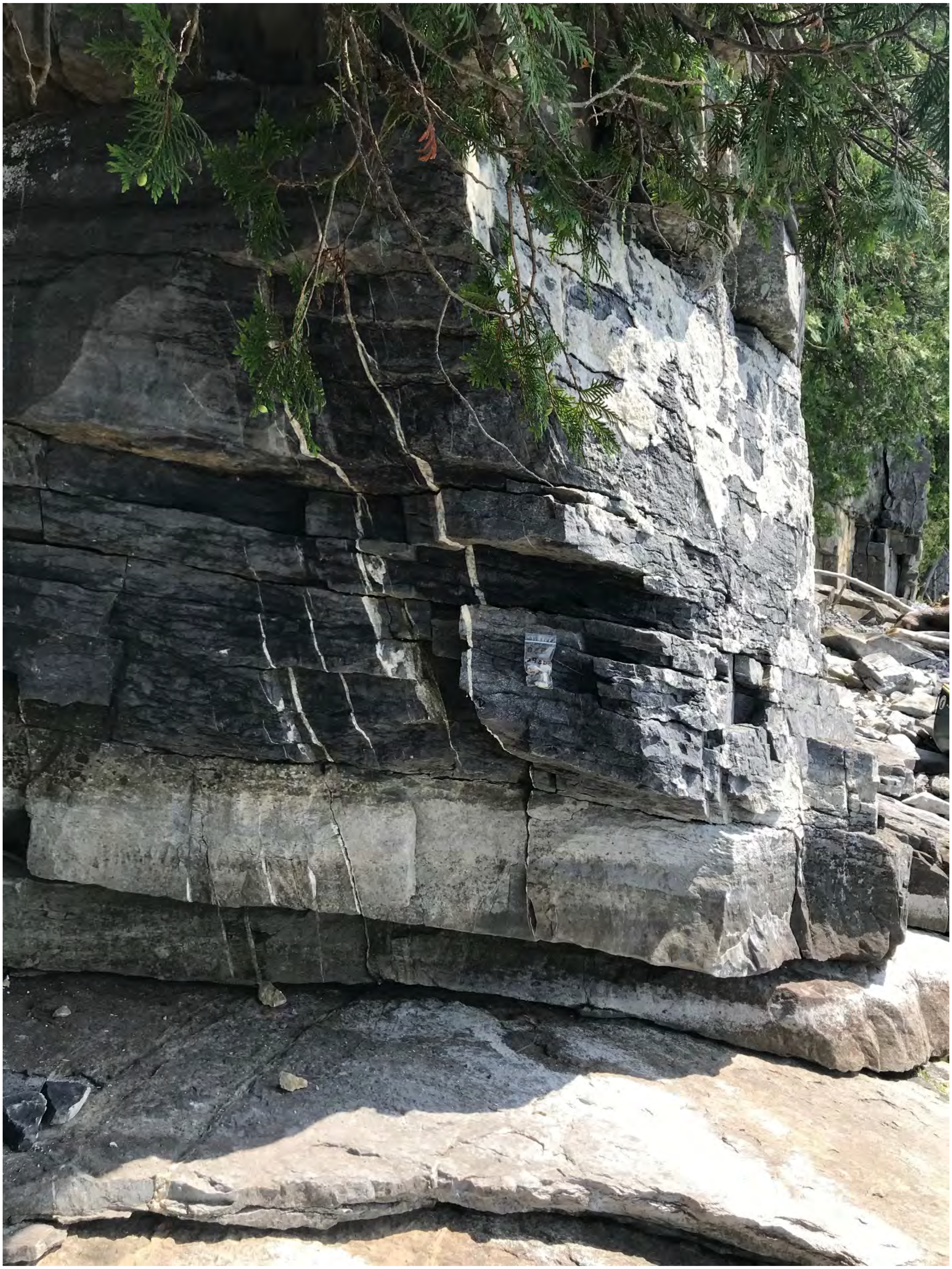

C273E: Massive spar within fault zone, no measurable strike/dip

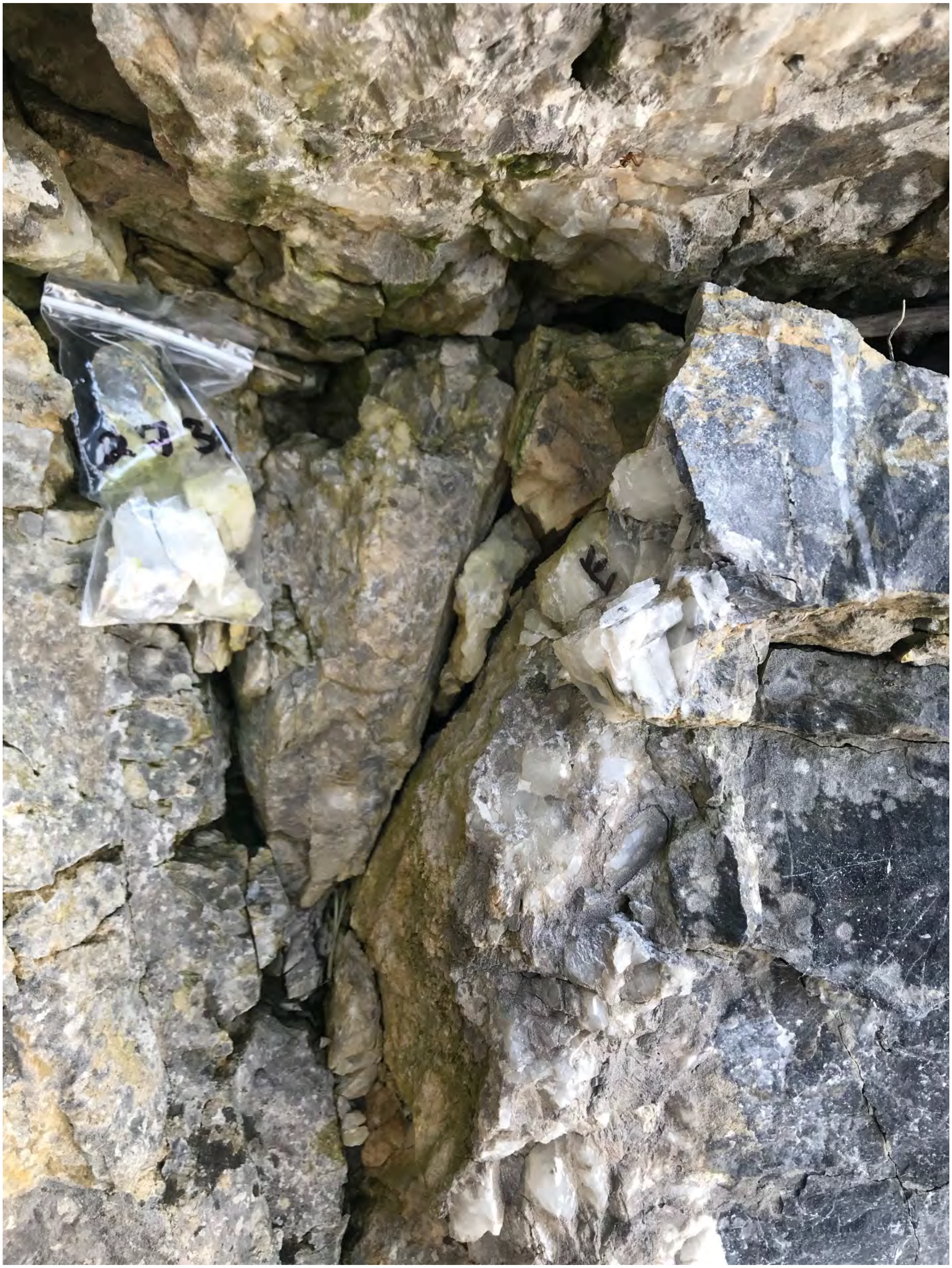

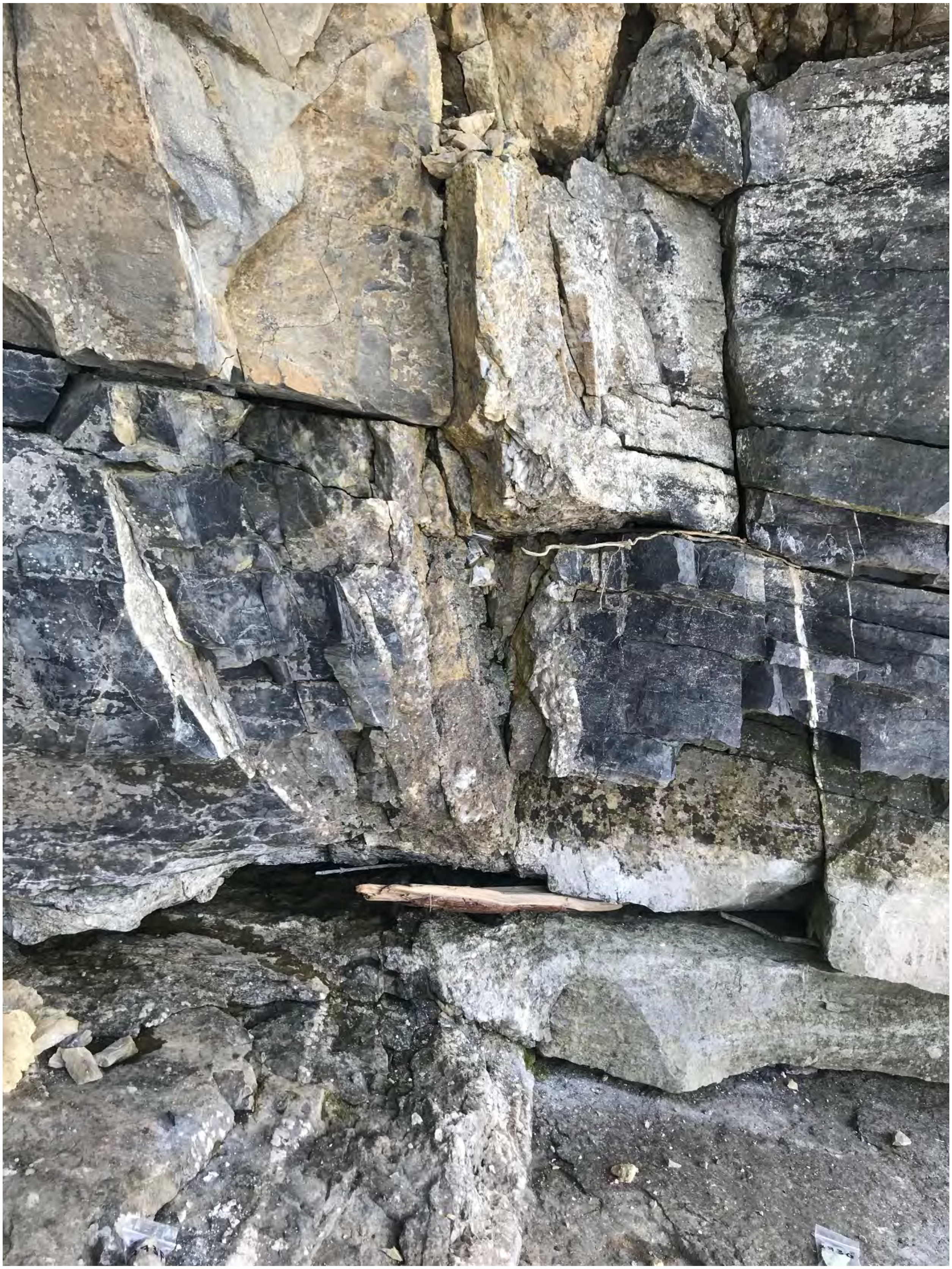

C273F: Sparry mode 1 fracture to left of fault zone

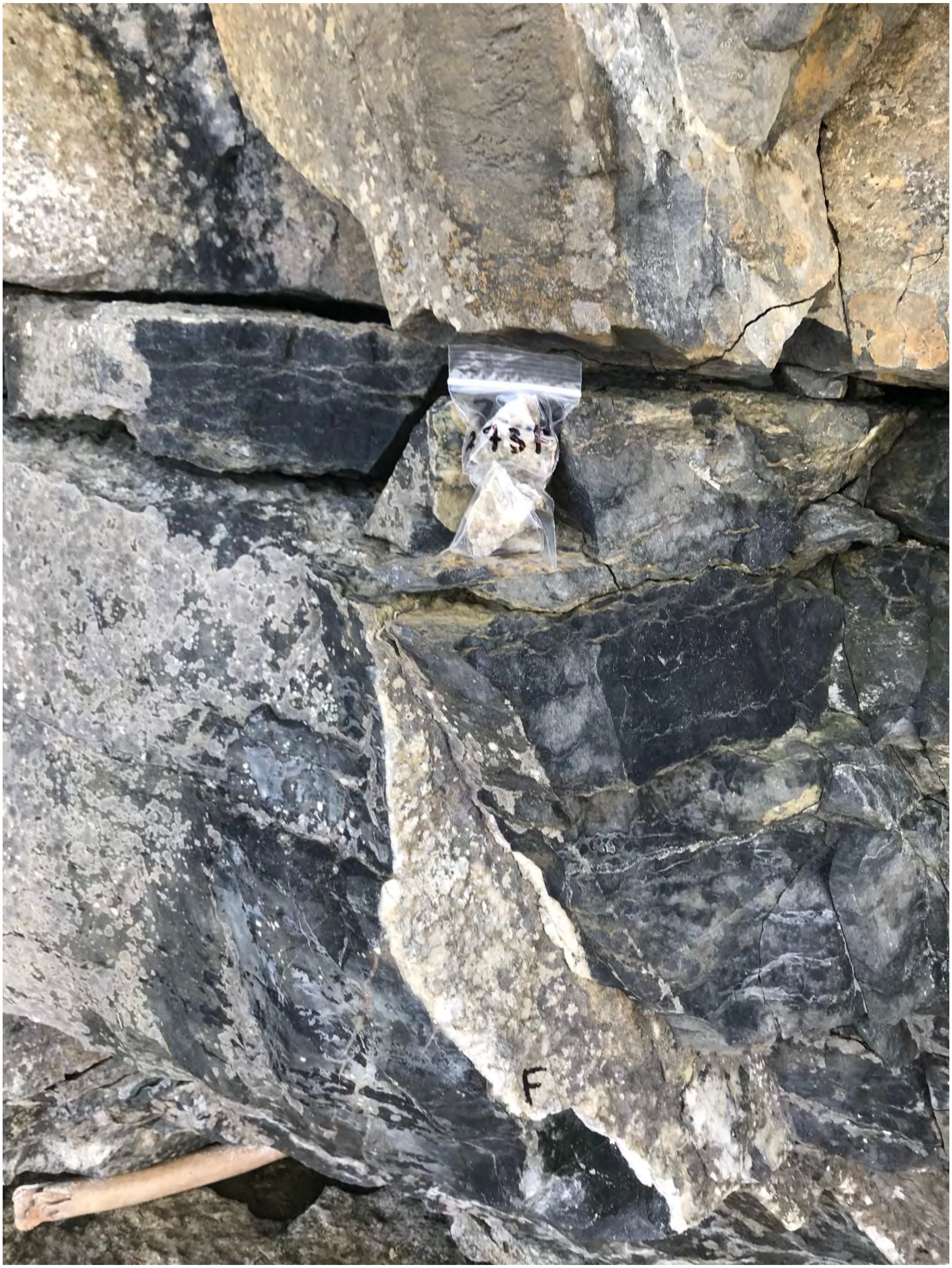

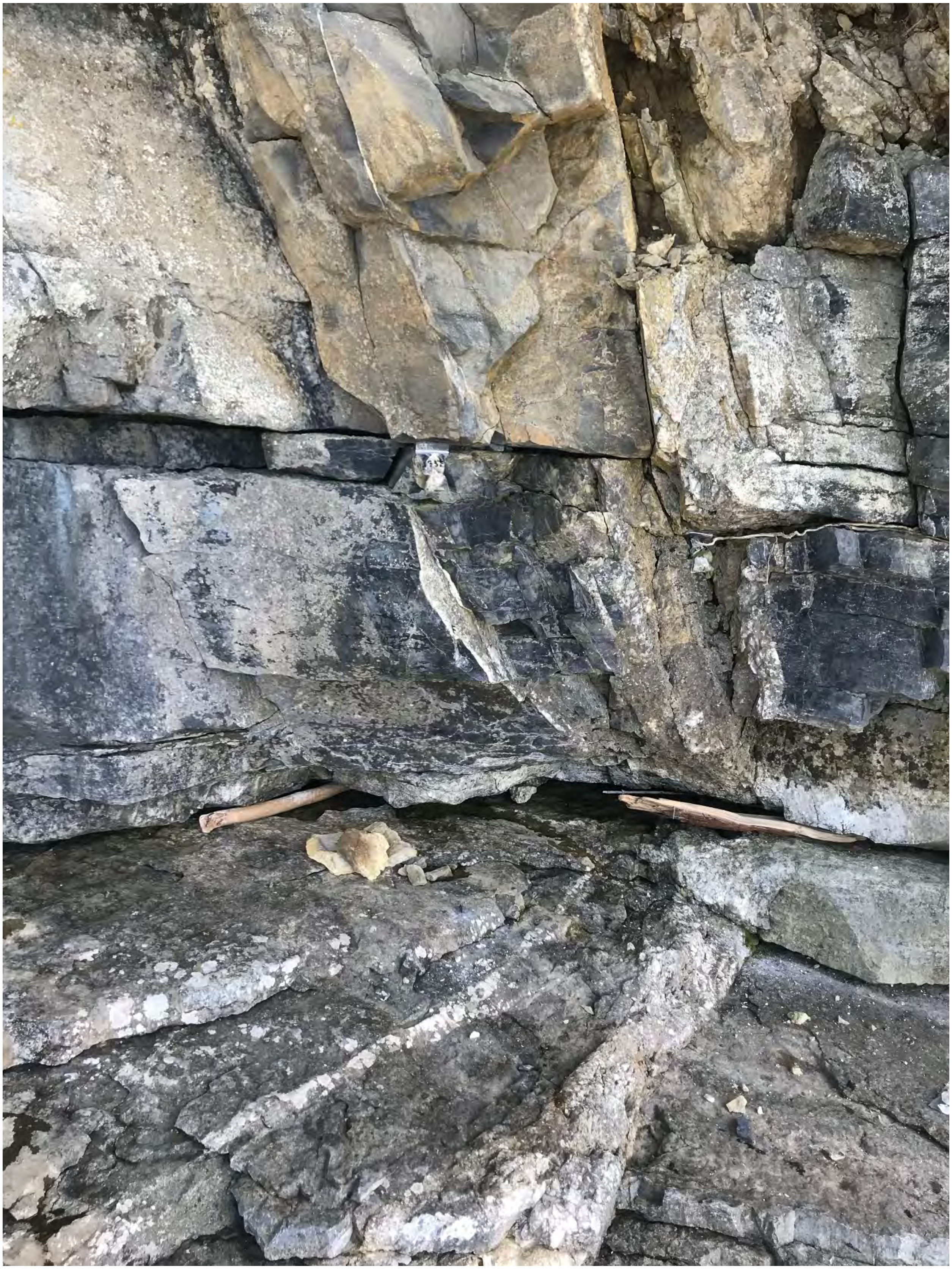

C273G: sub vertical vein to right of fault zone

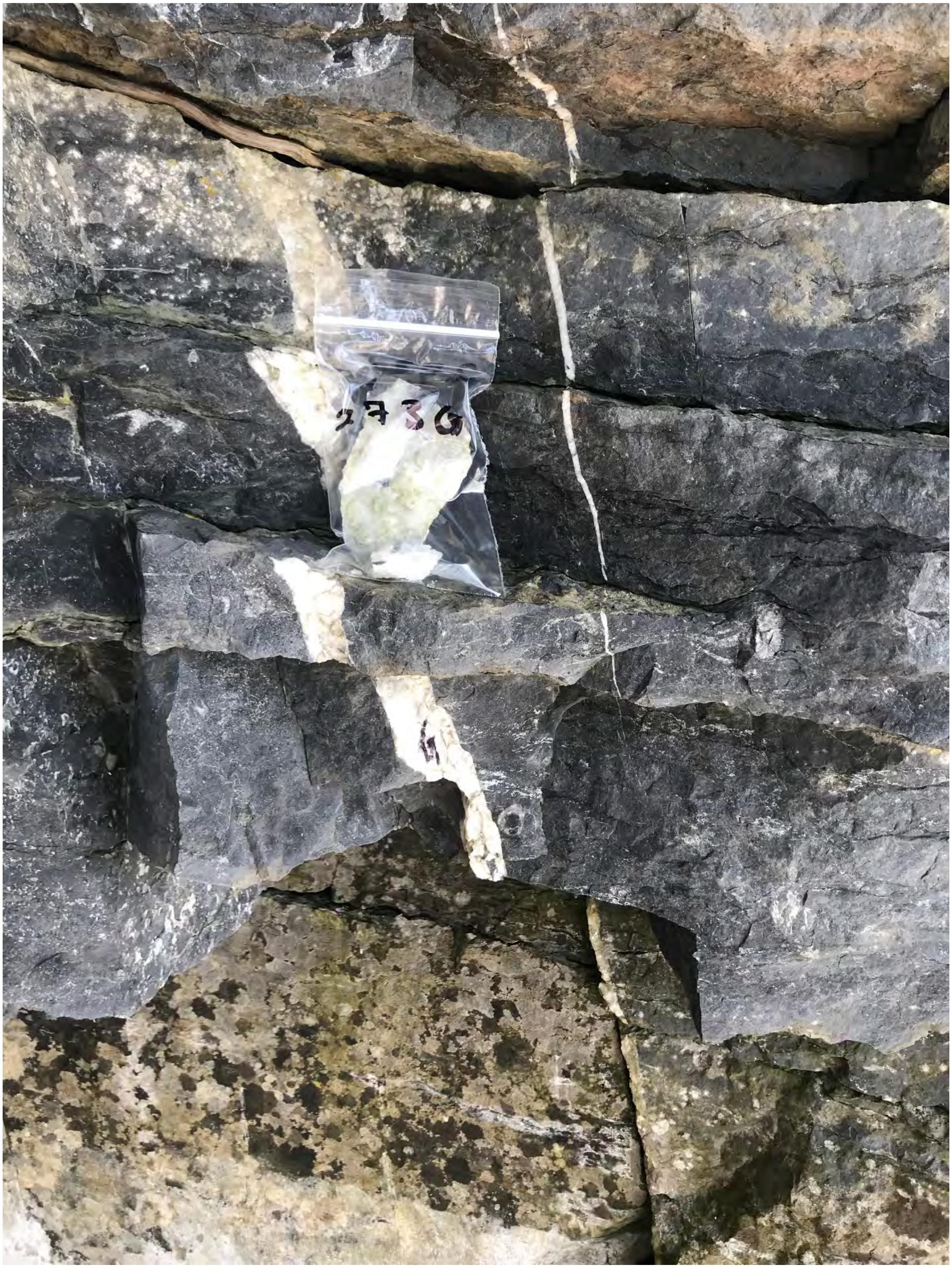

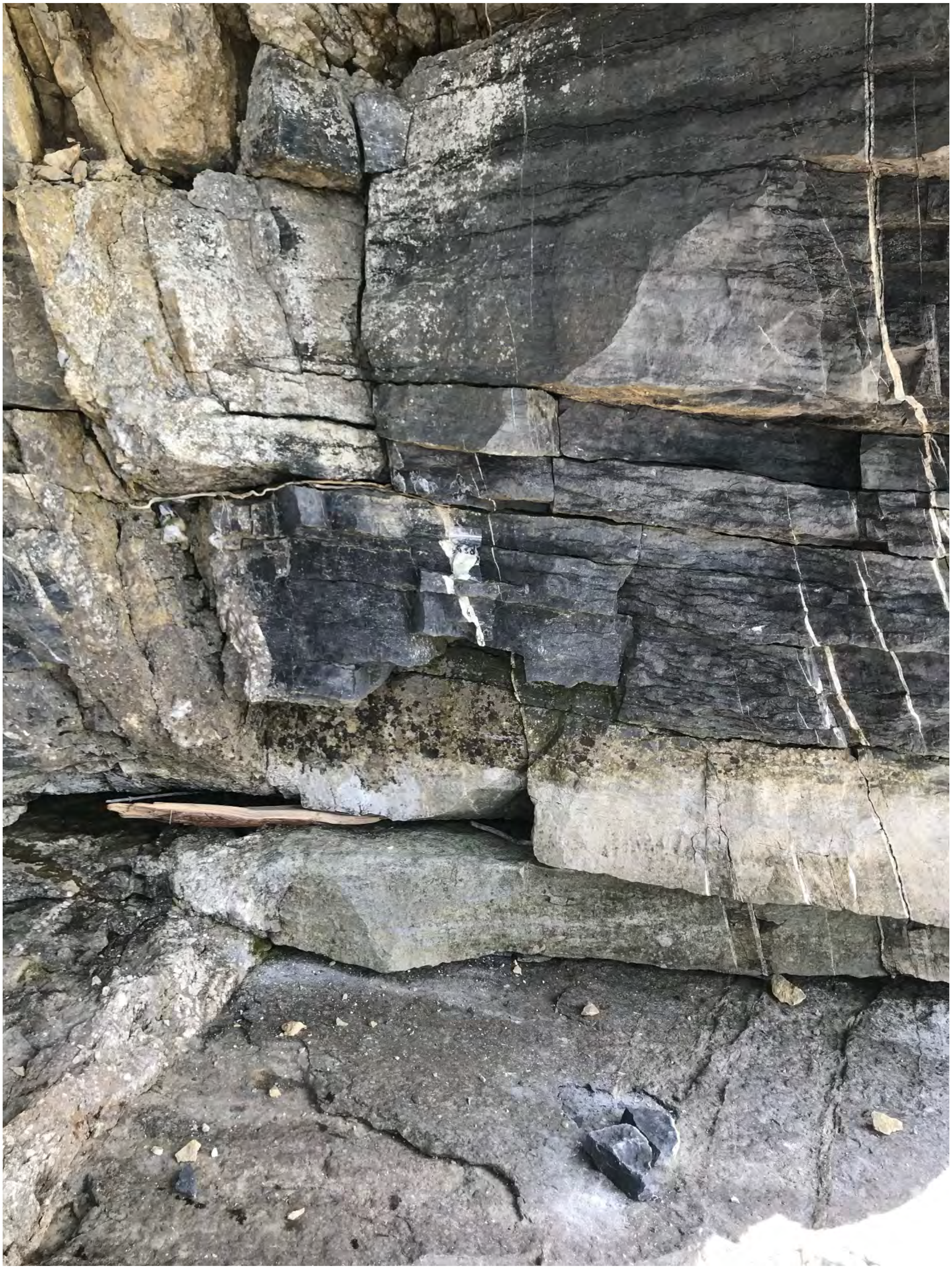

C273H: fault zone where it is exposed on shore platform about 15 m north of vertical exposure

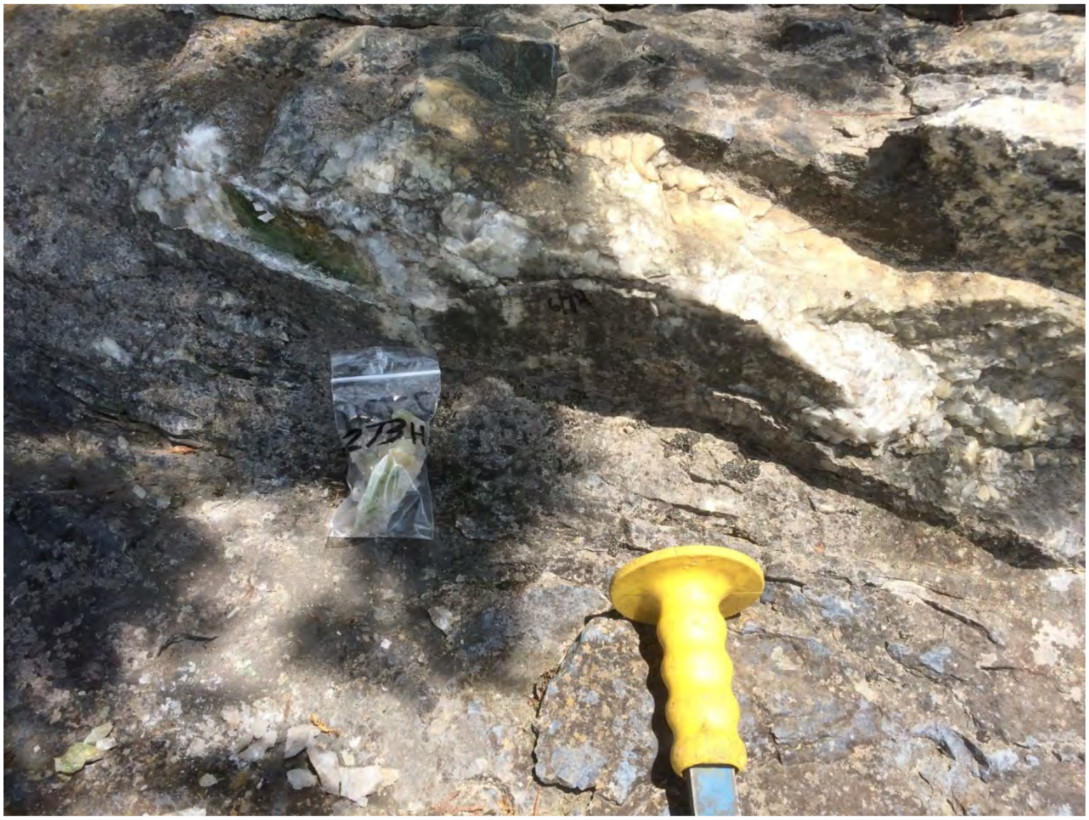

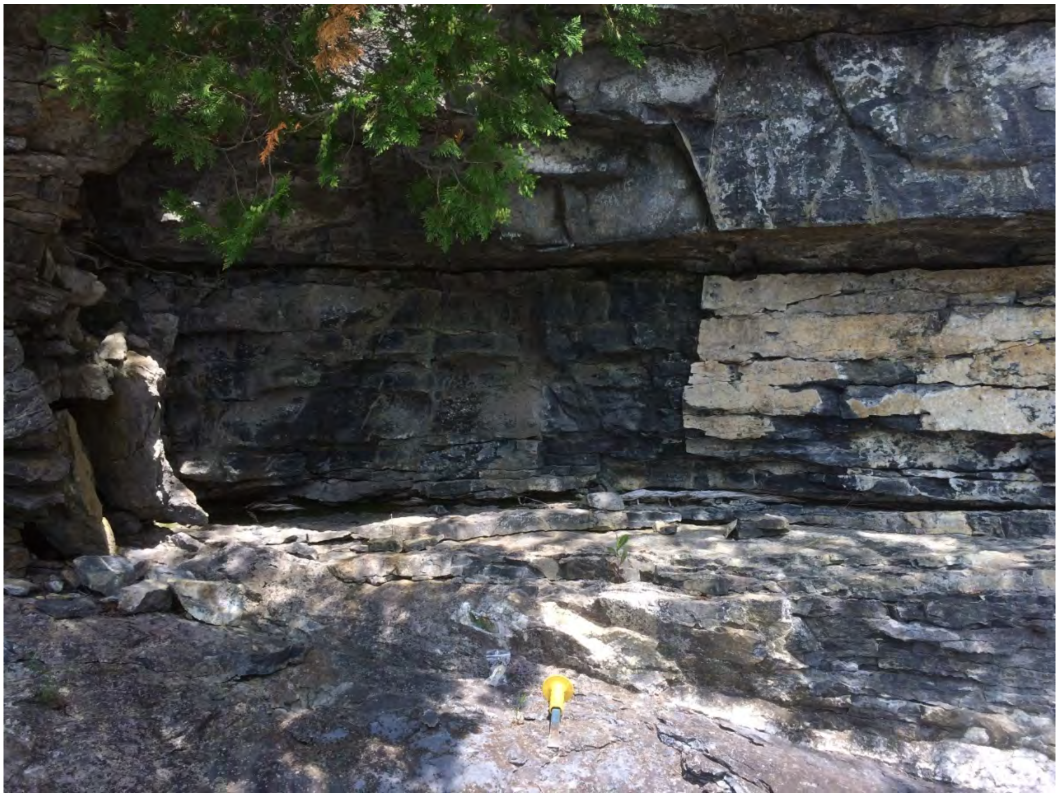

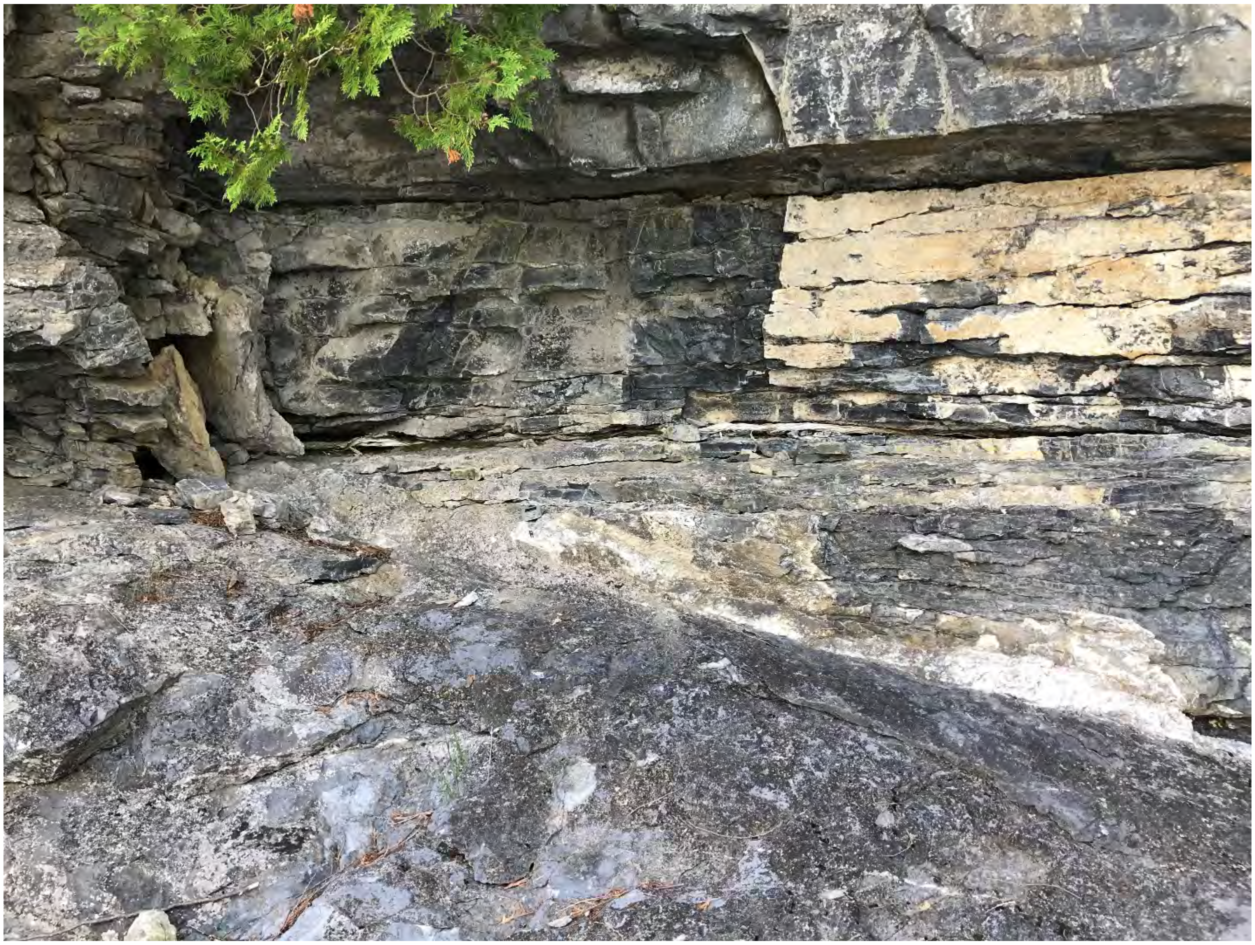

Lab Observations:

C273A: Circles in image denote areas targeted for U-Pb dating

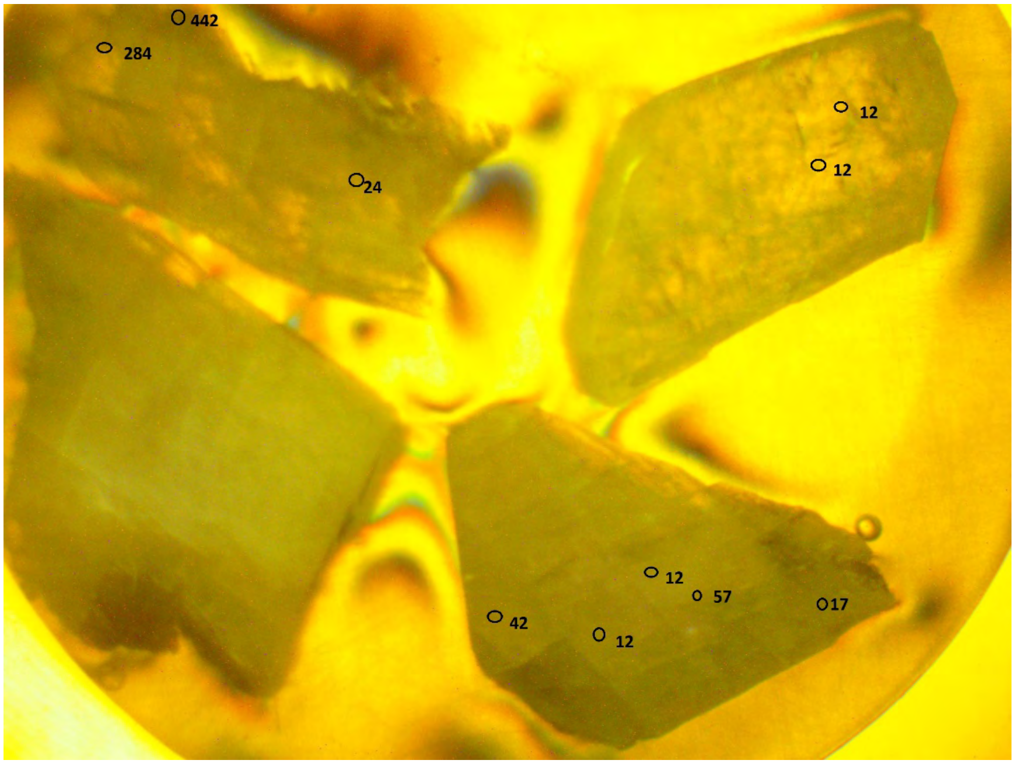

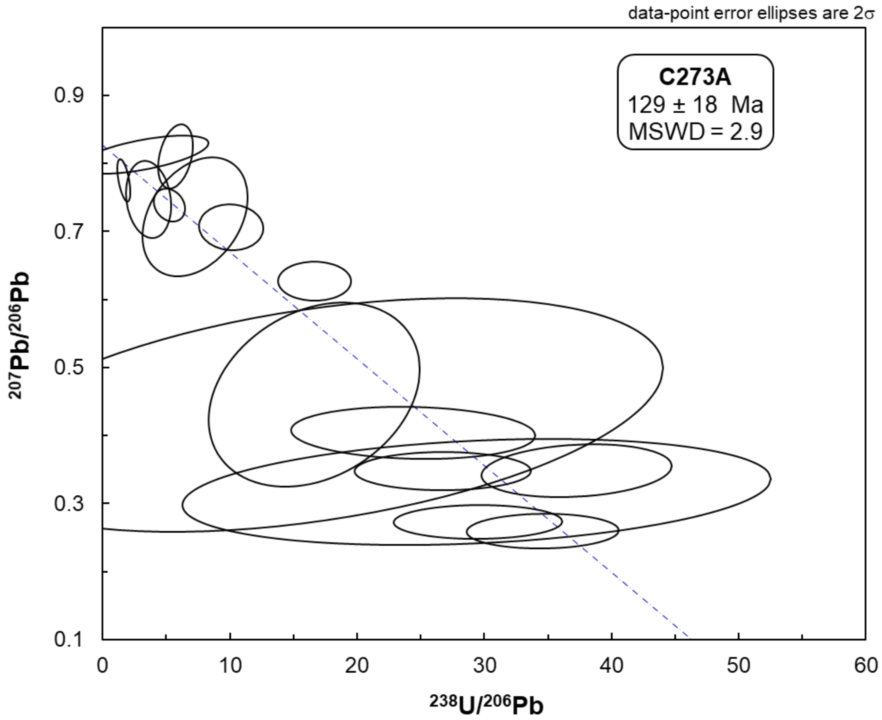

C273D: Circles in image denote areas targeted for U-Pb dating

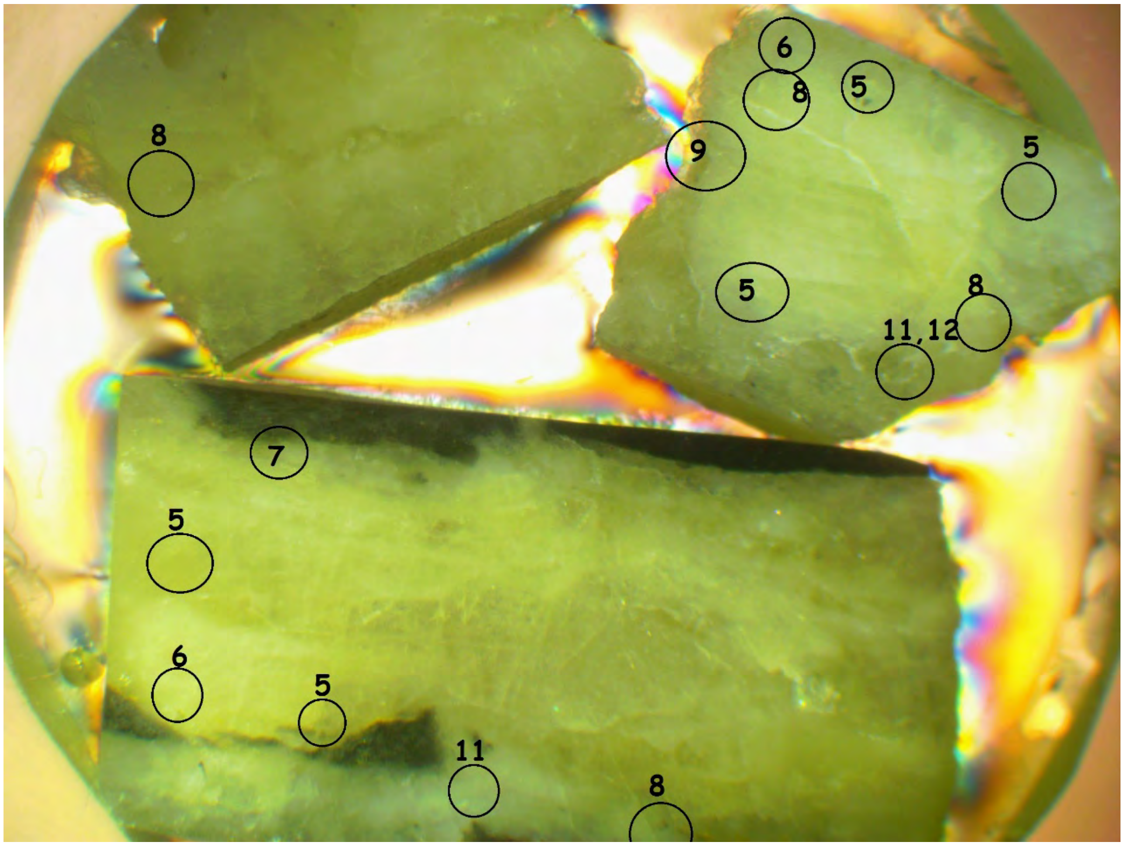

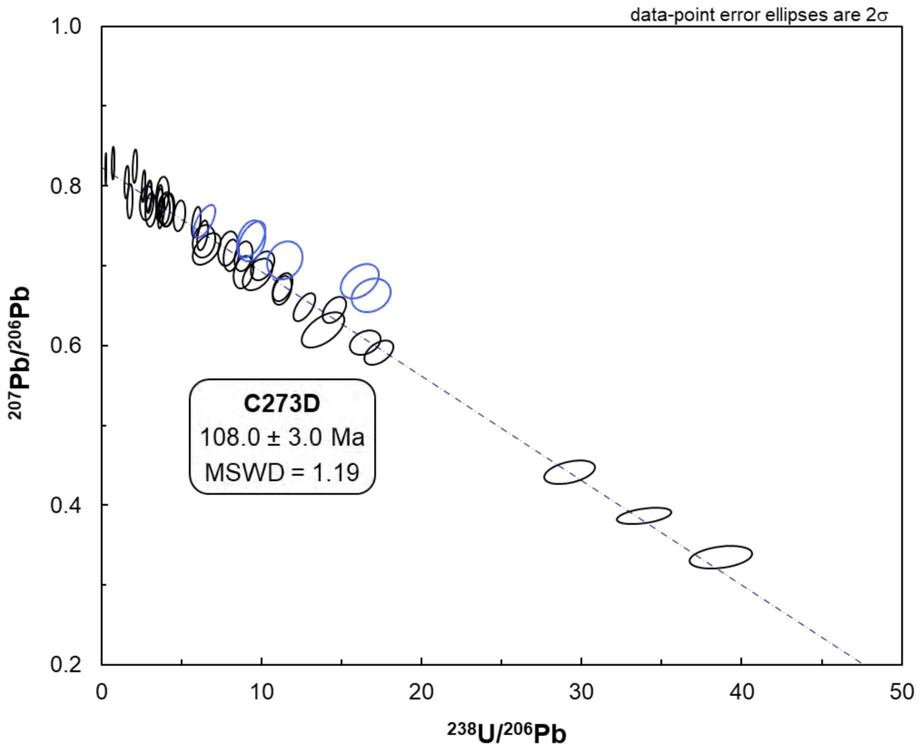

C273E: Circles in image denote areas targeted for U-Pb dating

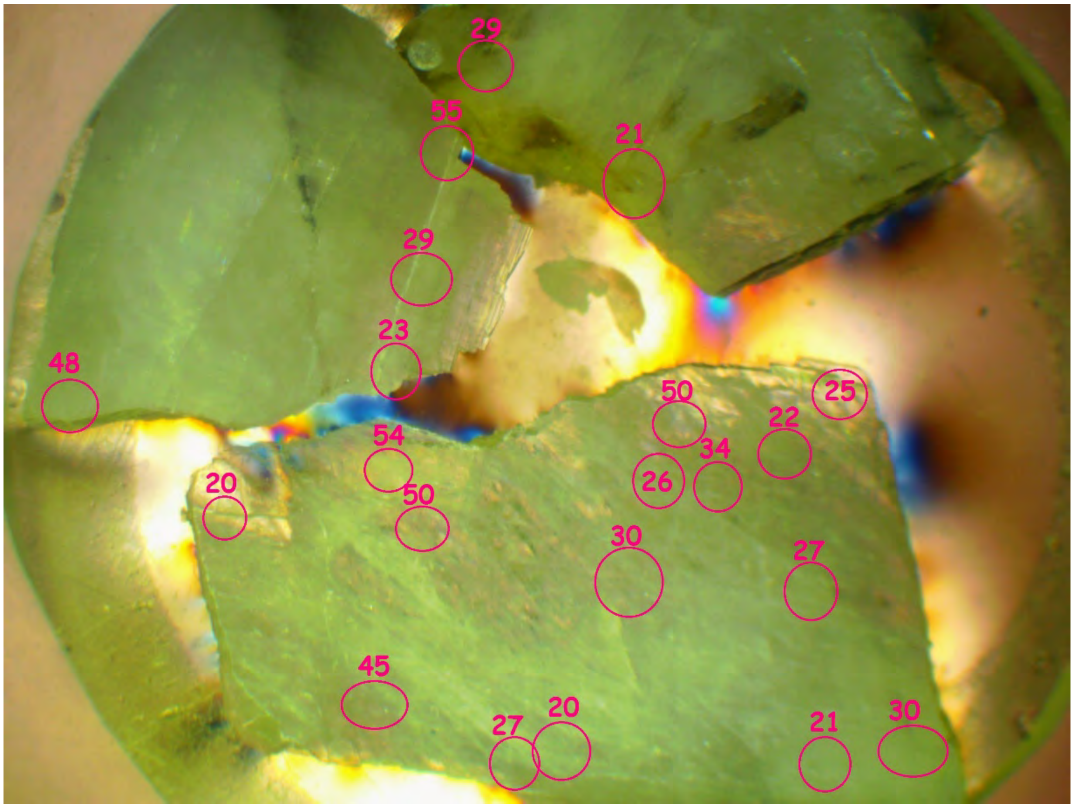

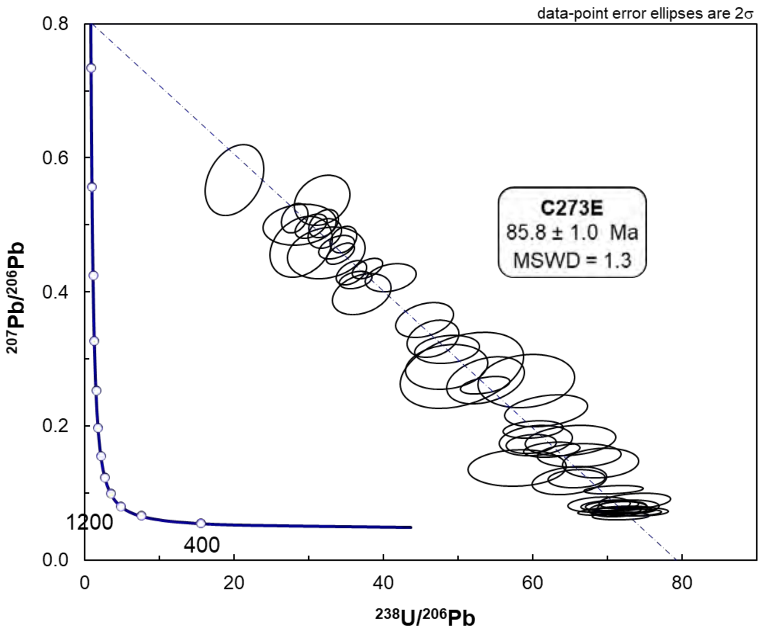

C273F: Circles in image denote areas targeted for U-Pb dating

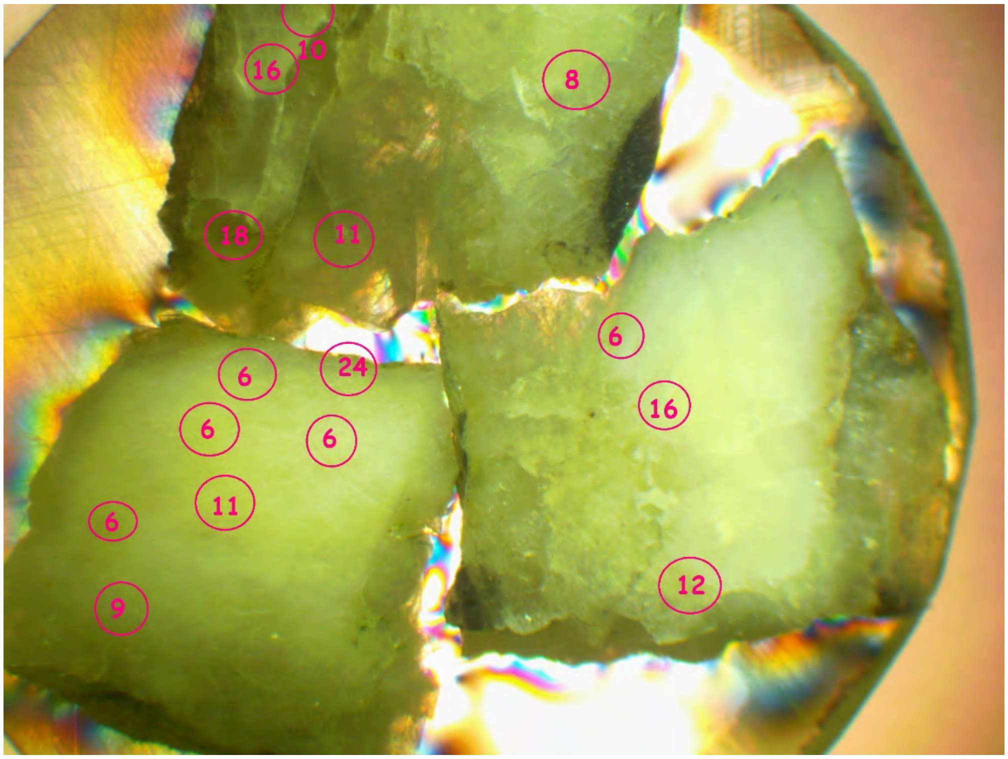

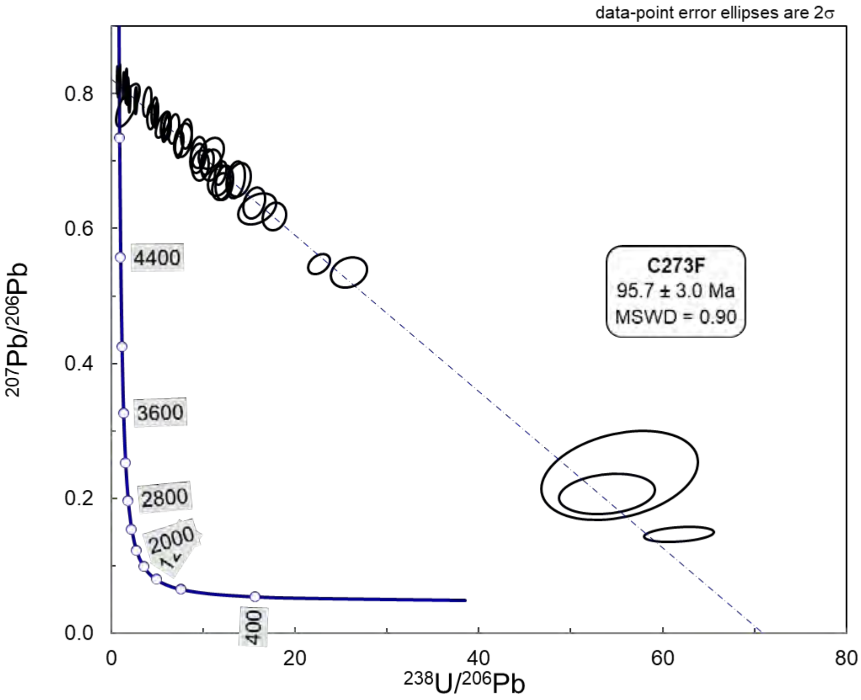

C273G: Circles in image denote areas targeted for U-Pb dating

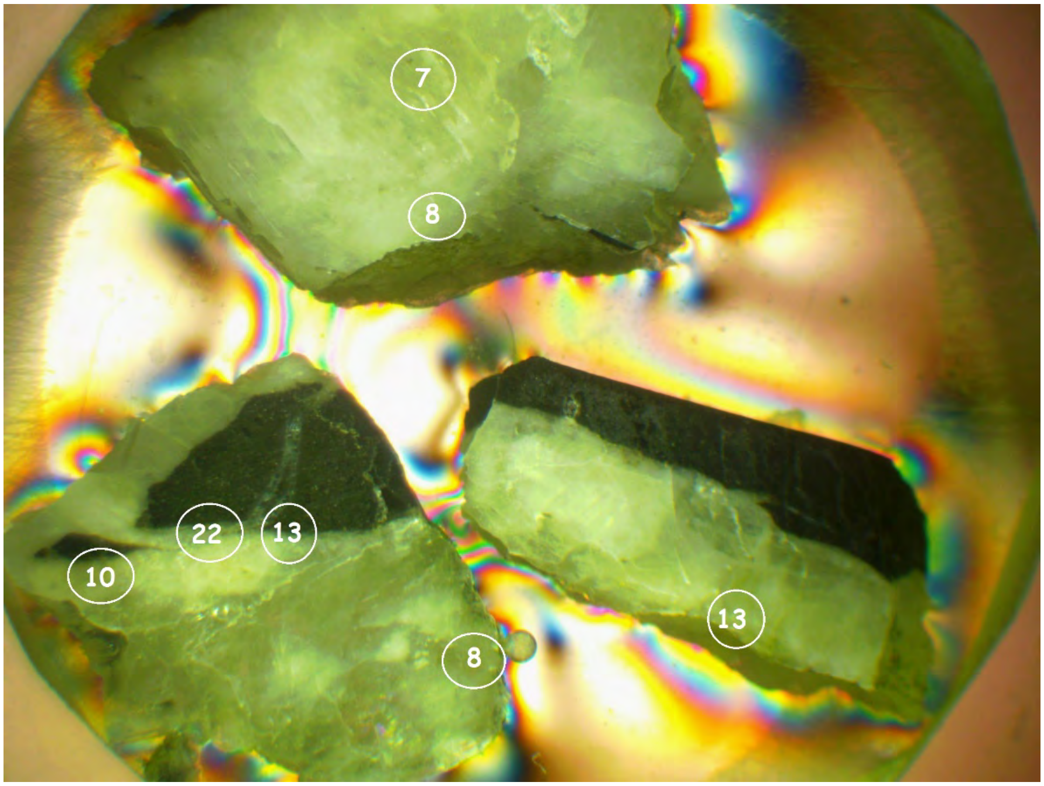

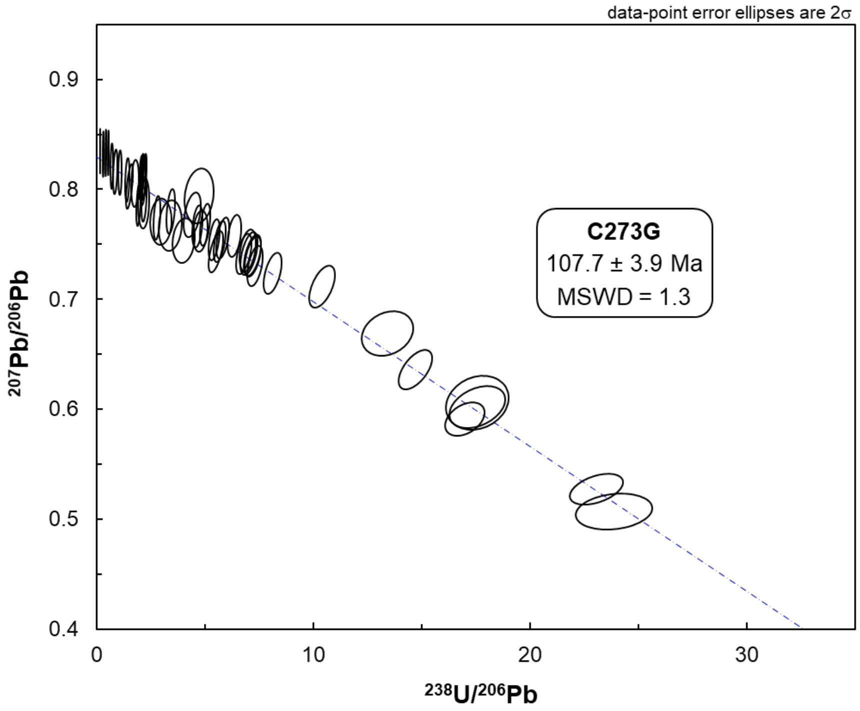

C273H: Circles in image denote areas targeted for U-Pb dating

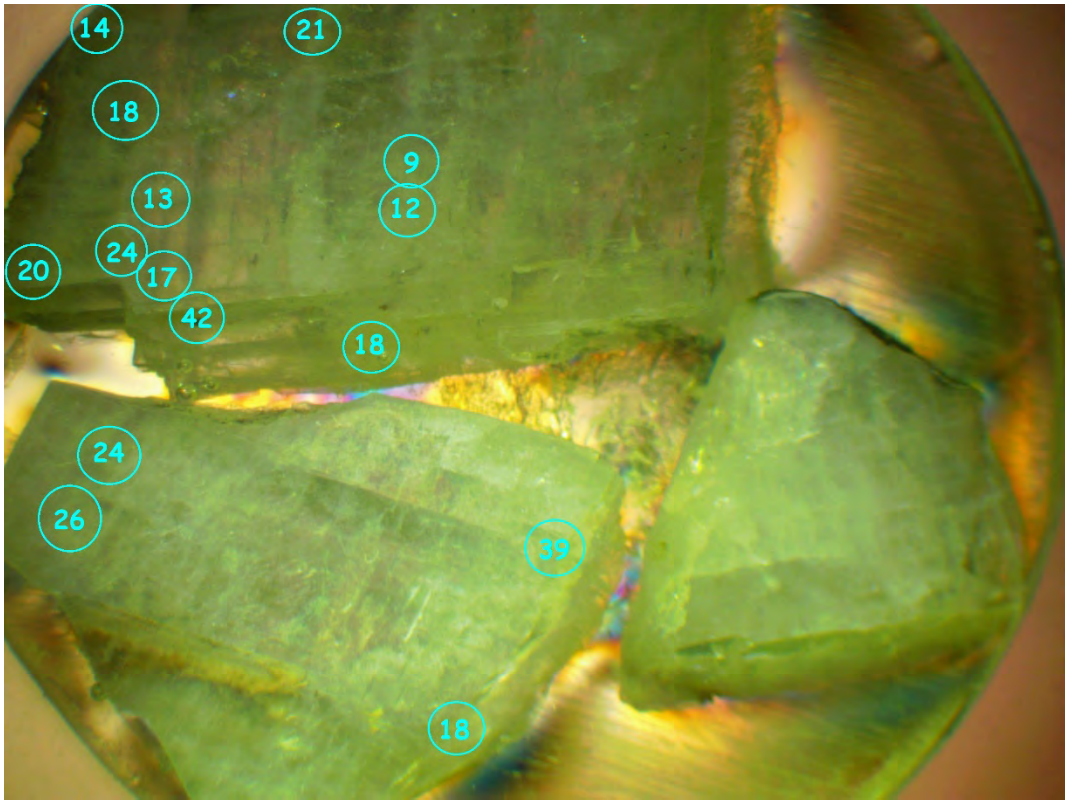

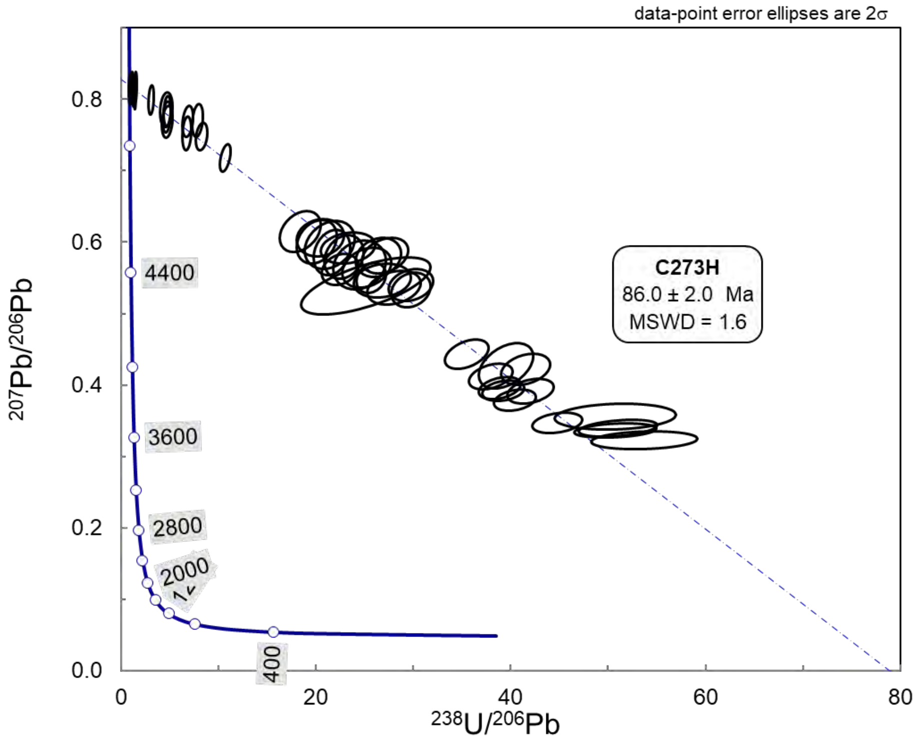

Supplement: Supplementary file 6 — Source Data [file 41467_2022_29680_MOESM6_ESM.zip › 2.zip!/appendix2b_site_summaries_21to50/21c_kingsland_bay_fault_south_final.pdf]
