# Supplementary material for: Pace of passive margin tectonism revealed by U-Pb dating of fracture-filling calcite
Source: Nat Commun. 2022 Apr 12;13:1953. doi: 10.1038/s41467-022-29680-z (PMC9005700; doi:10.1038/s41467-022-29680-z)

22: Lake Champlain Porter Bay Site (44.23203, -73.3183)

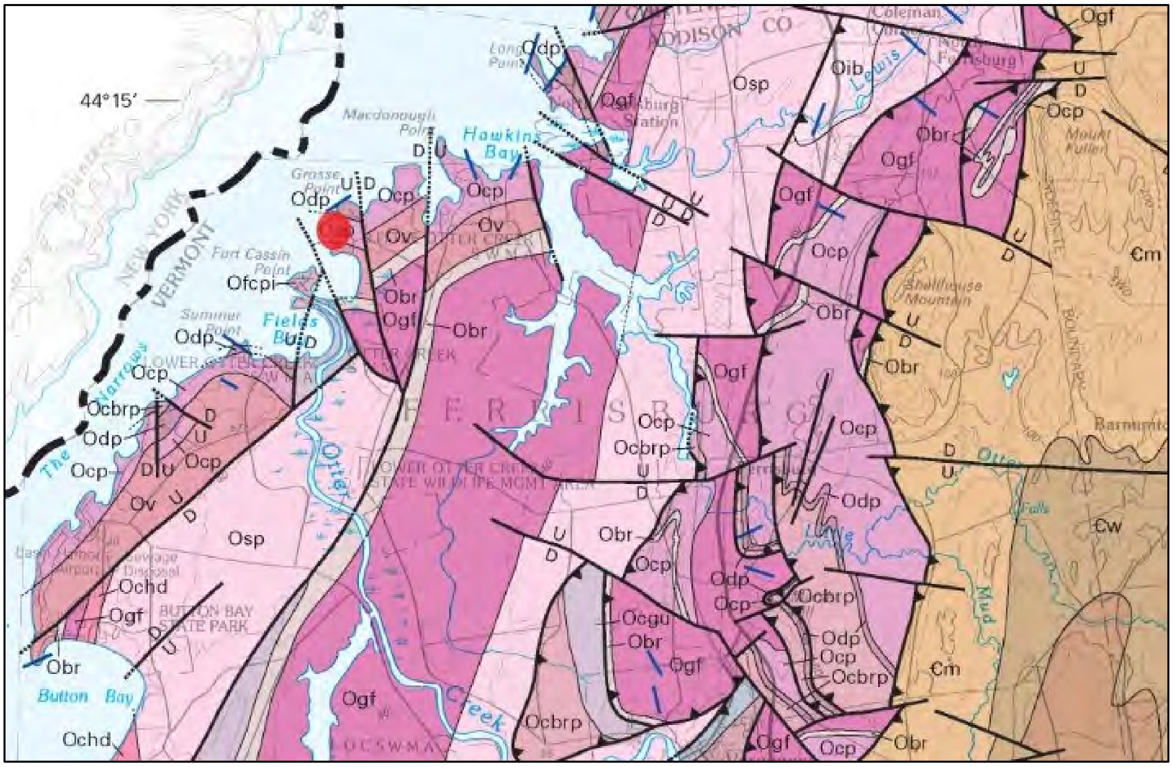

Figure 1: Sample location on state geologic map of Ratcliffe at al. (2011).

<u>Failed samples from same site:</u> C274B

<u>Bedrock Unit:</u> Crown Point Formation (Ocp)

<u>Ages</u>: C274: 108.7 ± 2.2 Ma **C274C**: 108.5 ± 4.6 Ma

Summary: This site sites on the north shore of Porter Bay about 1 mile southwest of Kingsland Bay. It is best accessed by boat from the Ferrisburgh Town Beach or Fort Cassin Boat Launch on Otter Creek. It consists of a roughly 2.5 wide zone of vertical, planar veins ranging from ~0.1 to 2 cm wide cutting through a massively bedded dolostone with relatively view joints or bedding planes. En-echelon fractures are also visible stepping both to the right and left as you follow them upward looking westward along their strike. Sample C274 comes from a ~1-2 cm wide syntaxial vein with ~ 1 mm scale spars and a visible center line in the vein. Sample C274C comes from a similar vein roughly 15 cm to the left of C274 and yields an almost identical age of 108.5 Ma. This generation of veins is well expressed in the outcrop and has an apparently conjugate set of calcite veins (see stereonet in figure 2 below), although this conjugate set remains undated.

We suggest all veins at site 22 formed in a single event at ~108 Ma based on: 1) the similar appearance and nature of all the veins and the consistent appearance of the calcite within them, 2) the lack off visible offsets of veins, 3) the high-quality, single domain U-Pb ages suggesting a single generation of crystallization, and 4) the excellent agreement of the stresses inferred from analysis of the conjugate vein sets and the en-echelon veins. Based on the presence of en-echelon fractures and

apparent conjugate vein sets we interpret these as mixed mode 'type II' fractures and infer σ_1 and σ_3 paleo-stresses as the line bisecting the intersection of the planes. By this method, the conjugate veins yield a σ_3 stress with trend and plunge of 356°/01° and a σ_1 stress of 103°/17°. The en-echelon veins yield a σ_3 stress of 182°/20° and a σ_1 stress of 275°/08°. The excellent agreement between the stresses inferred from en-echelon and apparent conjugates lends confidence to our structural interpretation.

Field Descriptions:

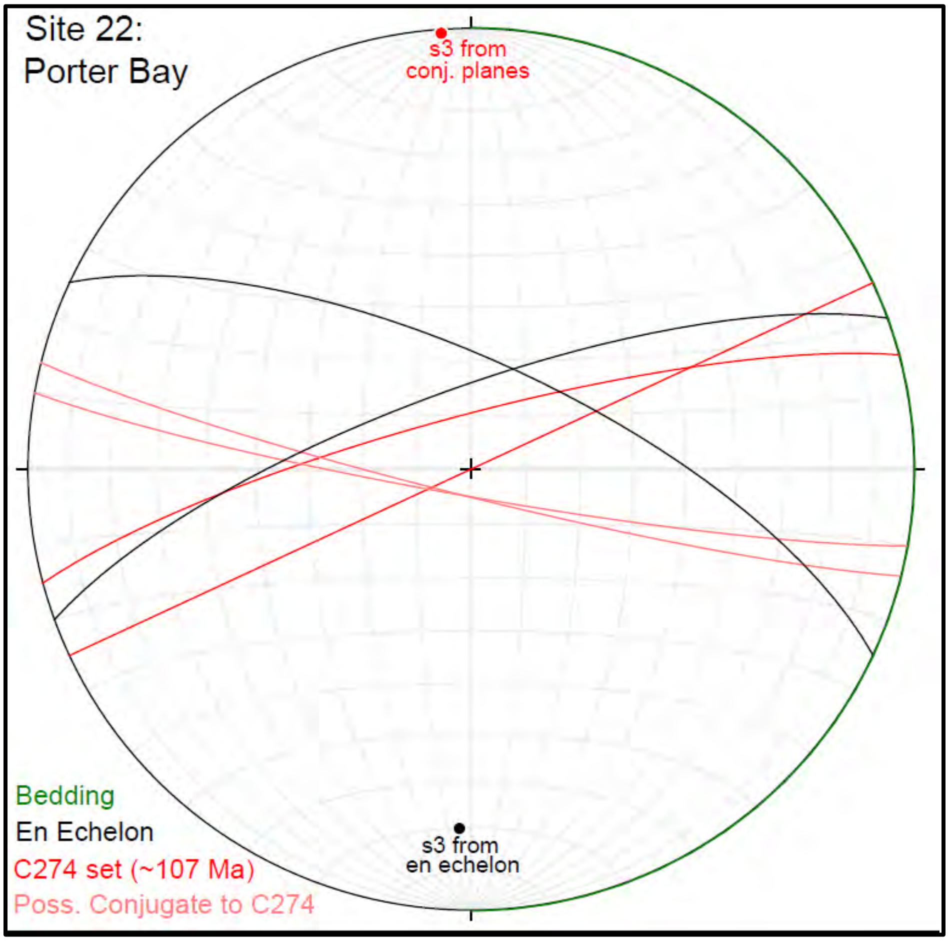

Figure 2: Stereonet showing the set of planes associated with C274 and a possibly conjugate set as well as the mean orientation of two sets of en echelon fractures interpreted as Type II (e.g. Bons et al., 2012).

Overview photos:

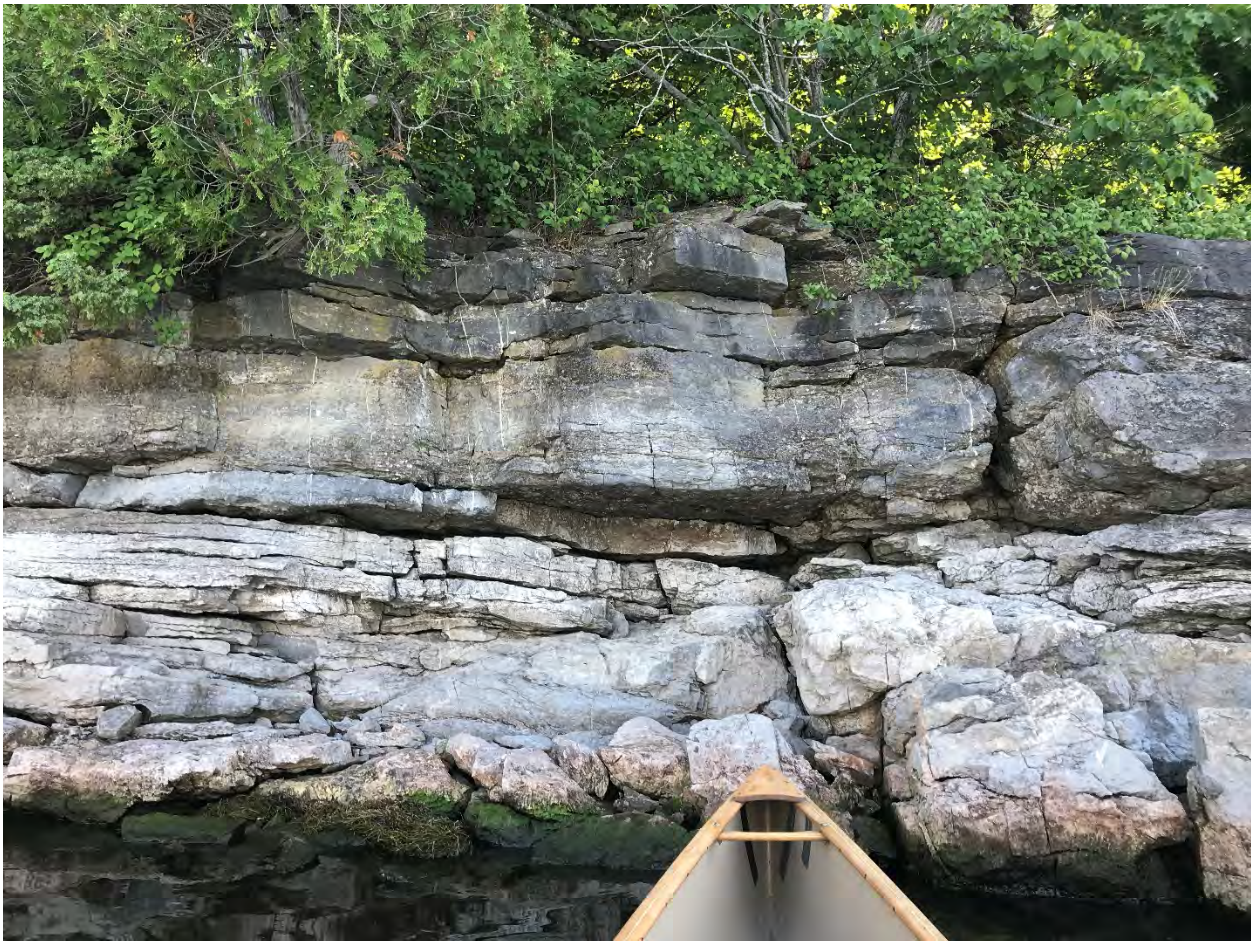

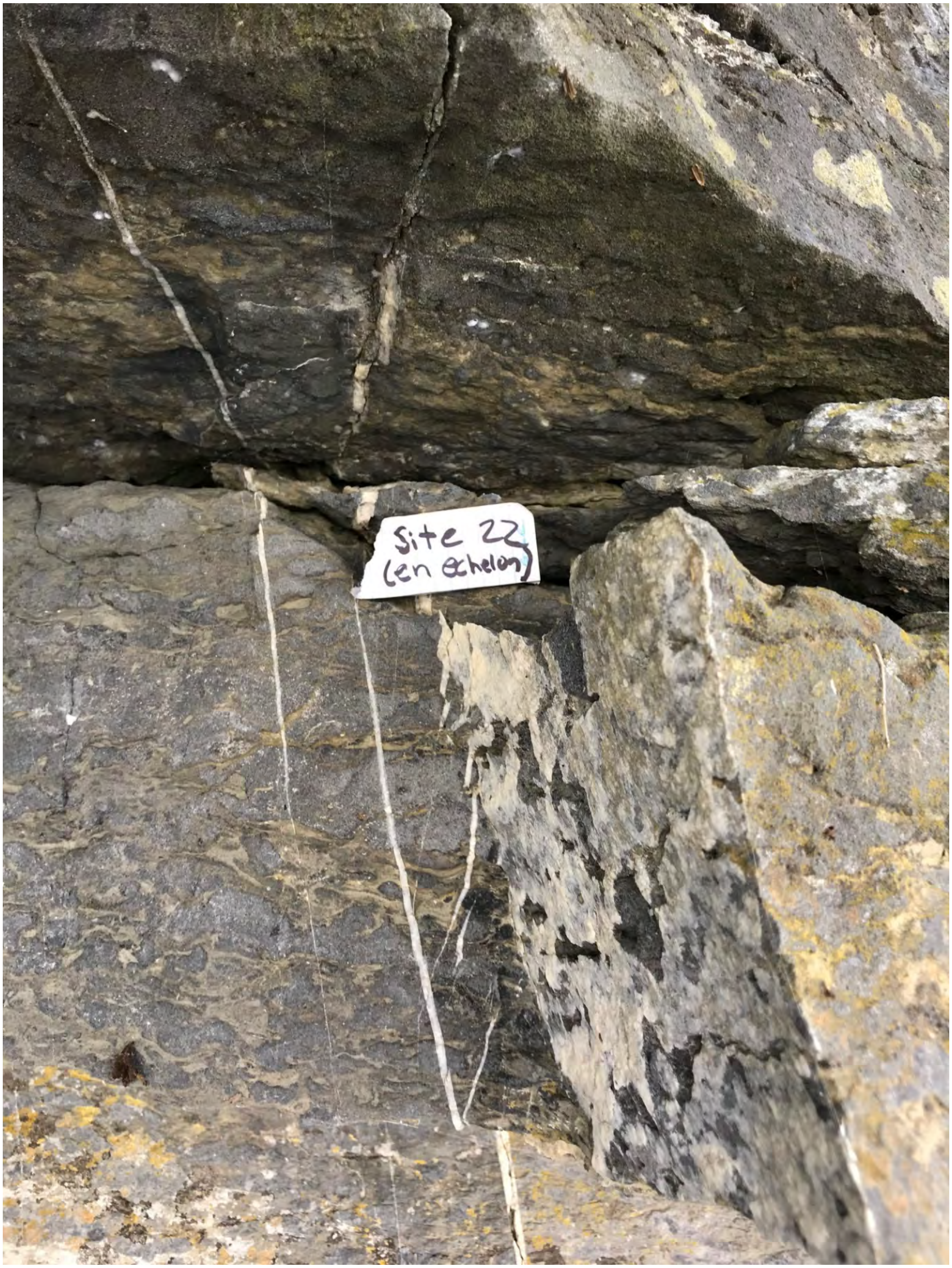
Site 22
(en echelon)

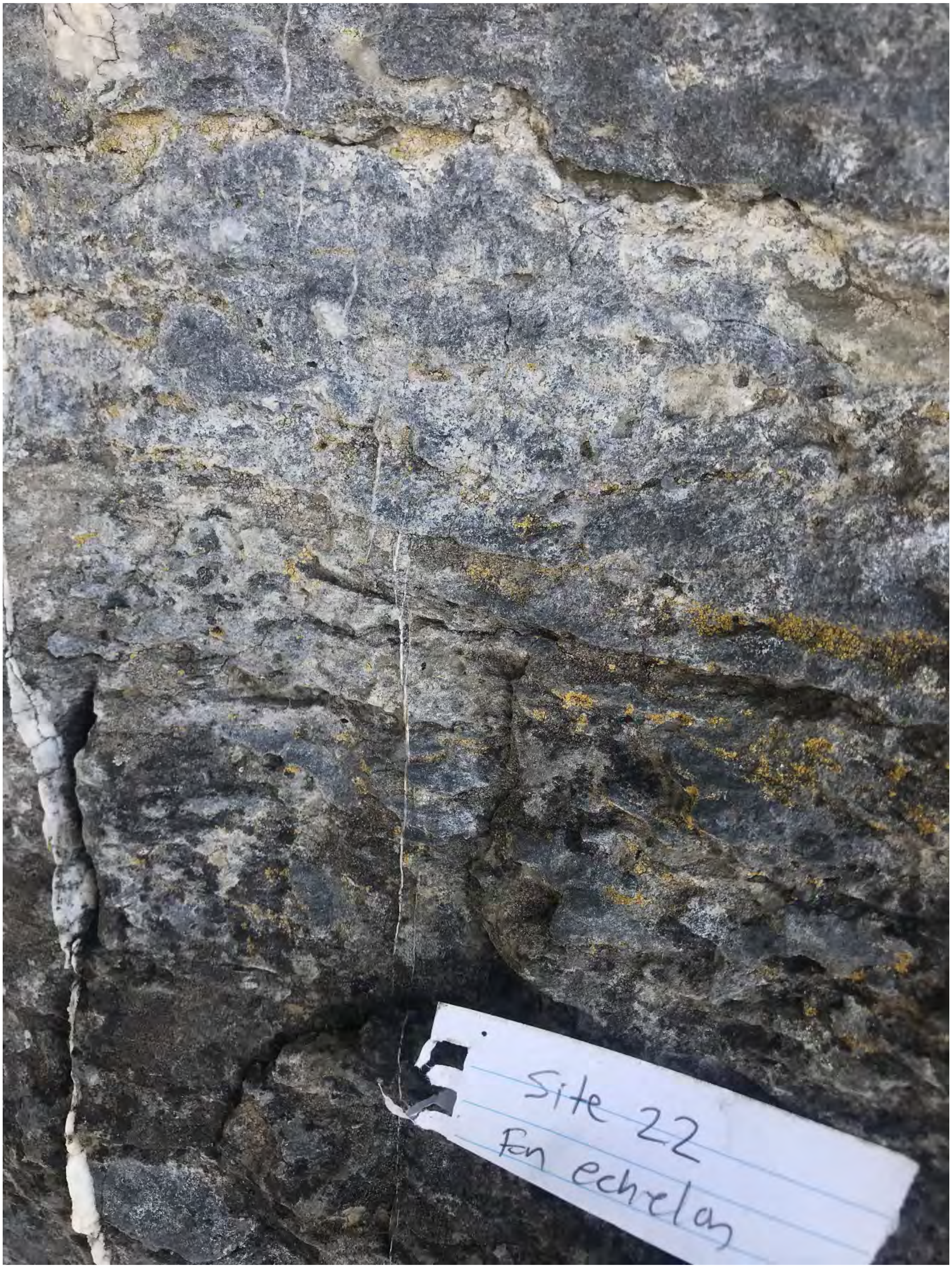
Site 22
En echelon

C274:

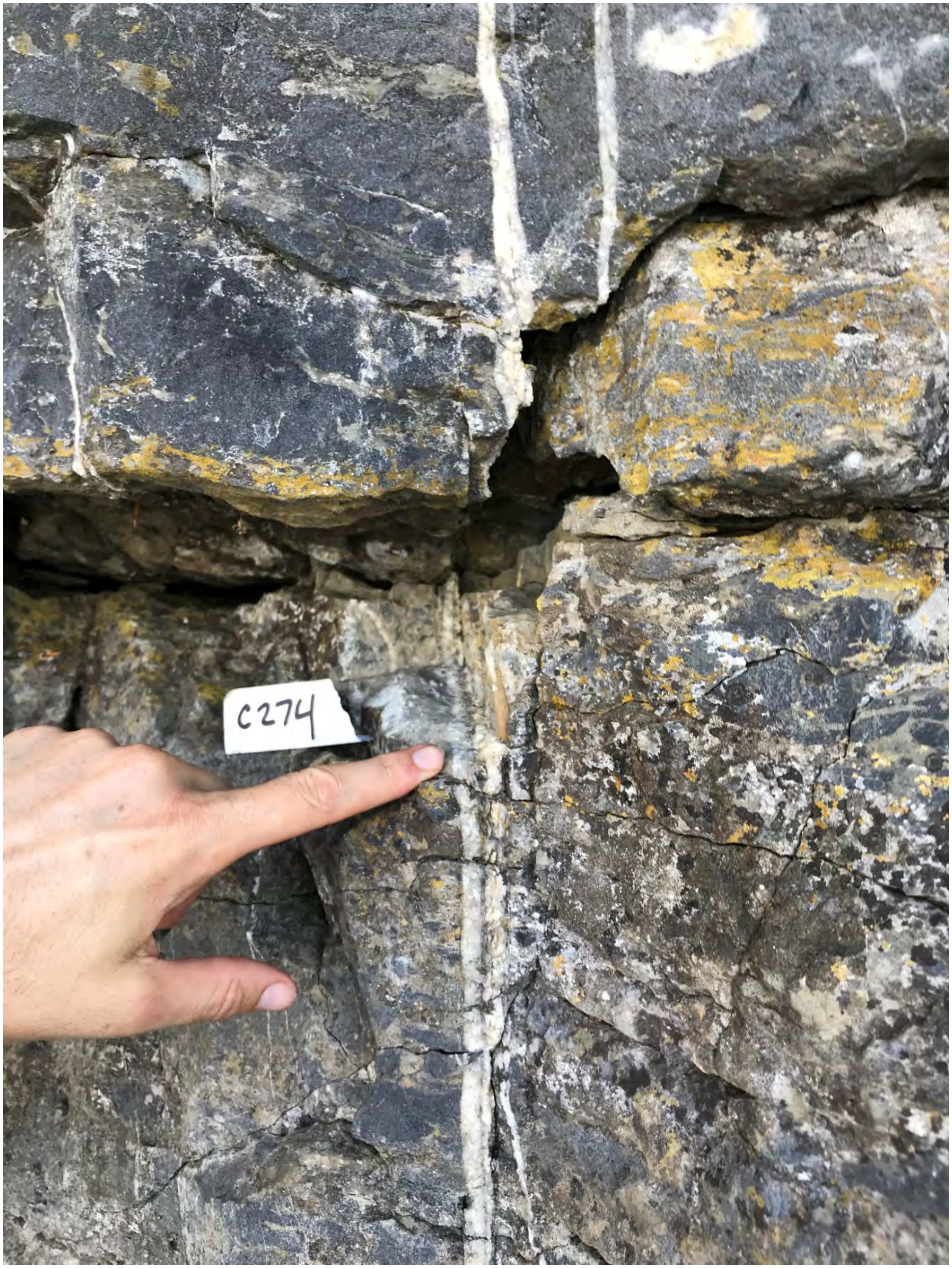

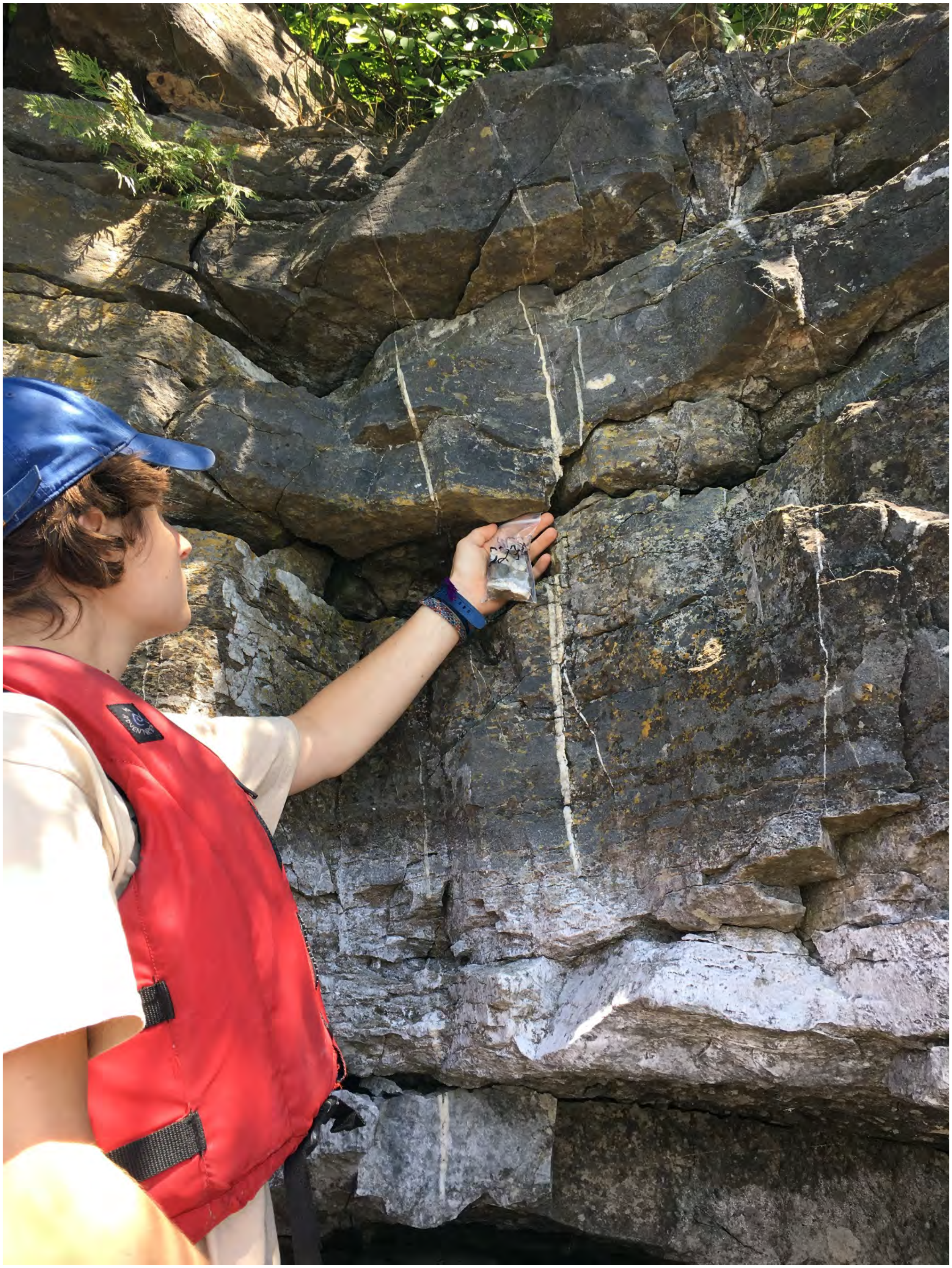

C274C:

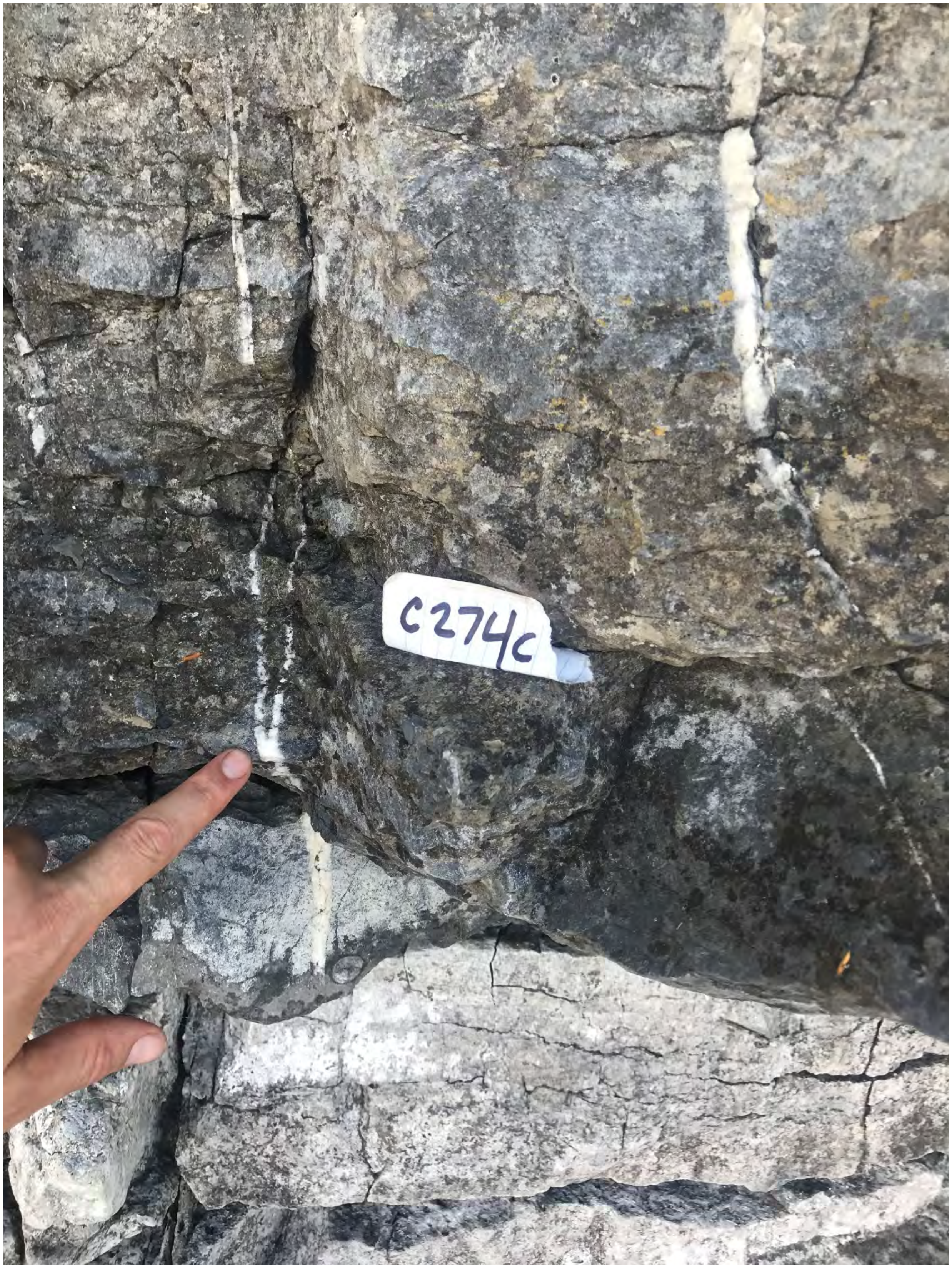

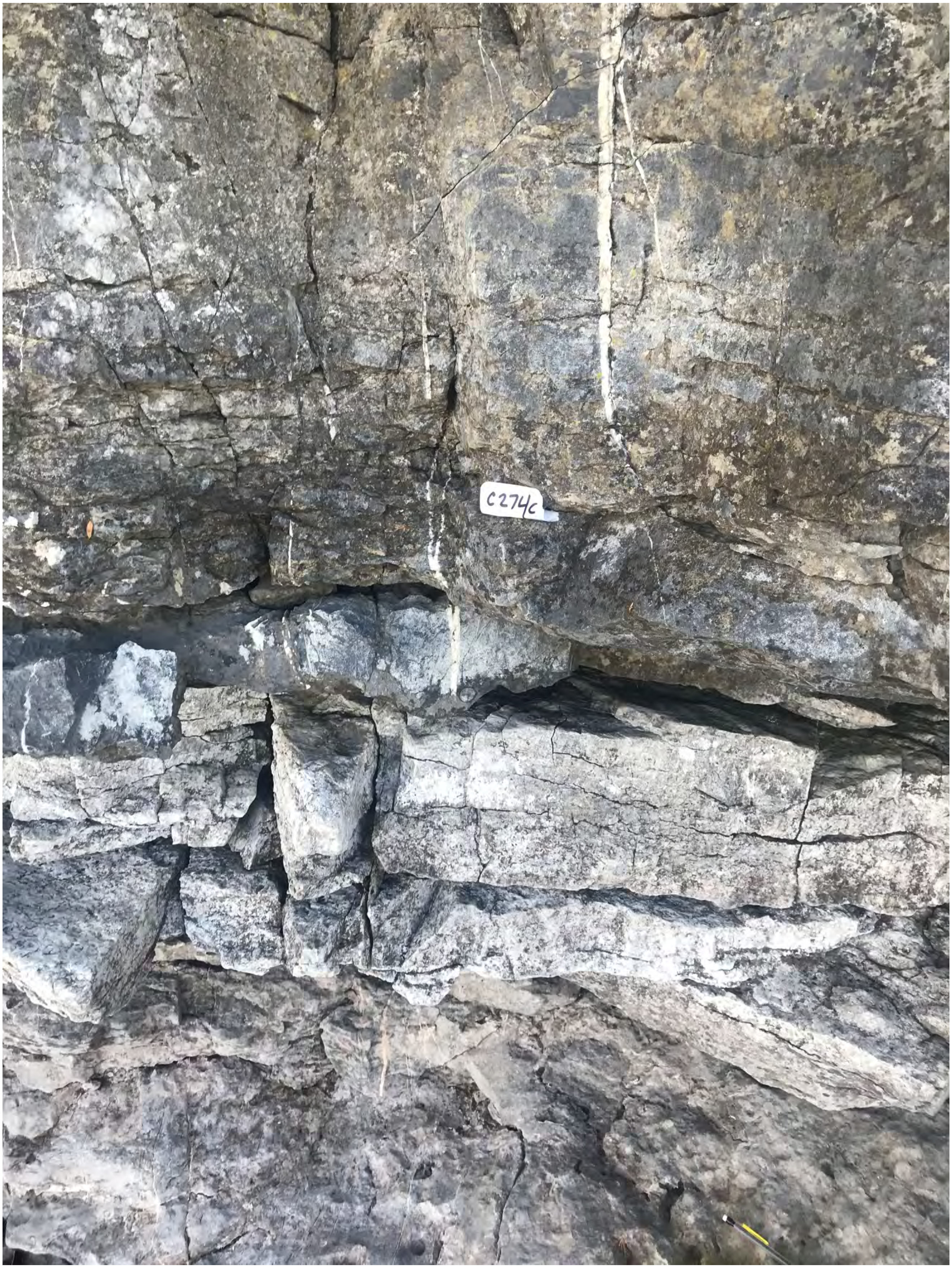
C274c

Lab Description: Circles in image are areas of interest, not laser shots

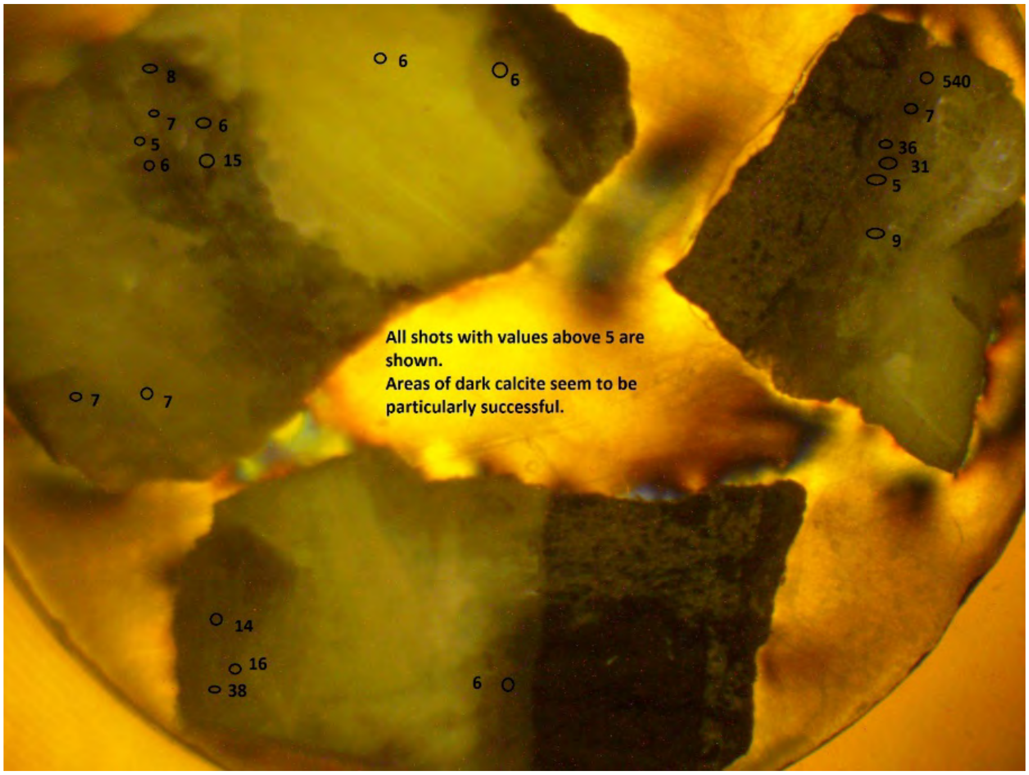

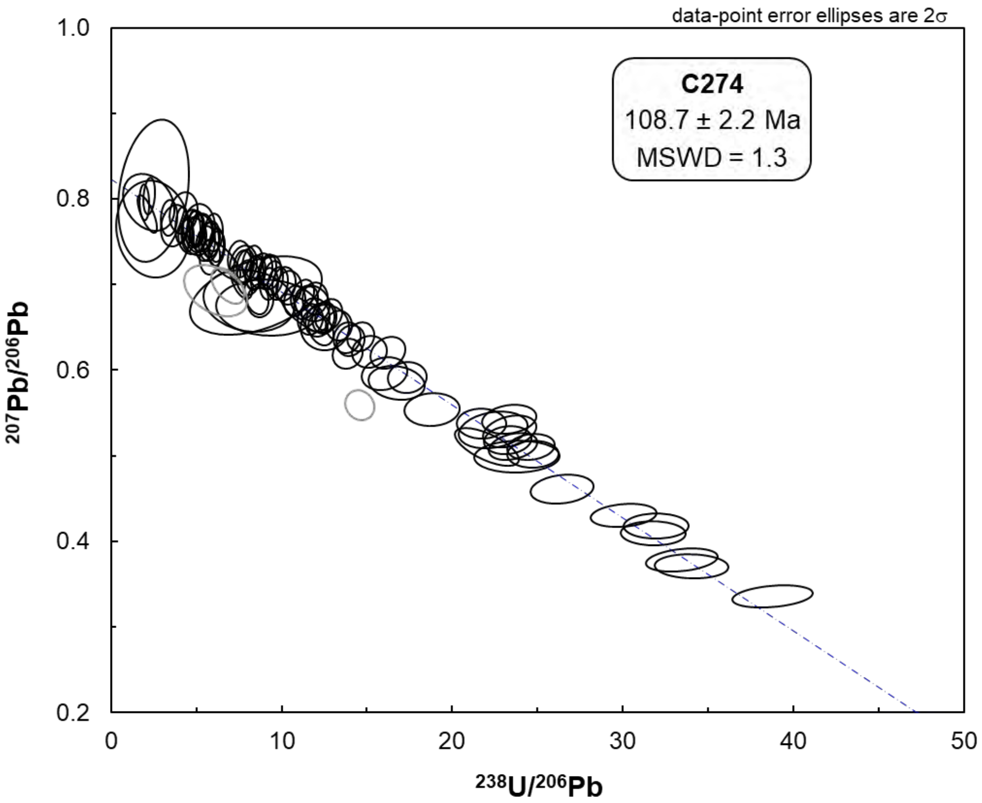

C274C: Circles/squares in image are areas of interest, not laser shots

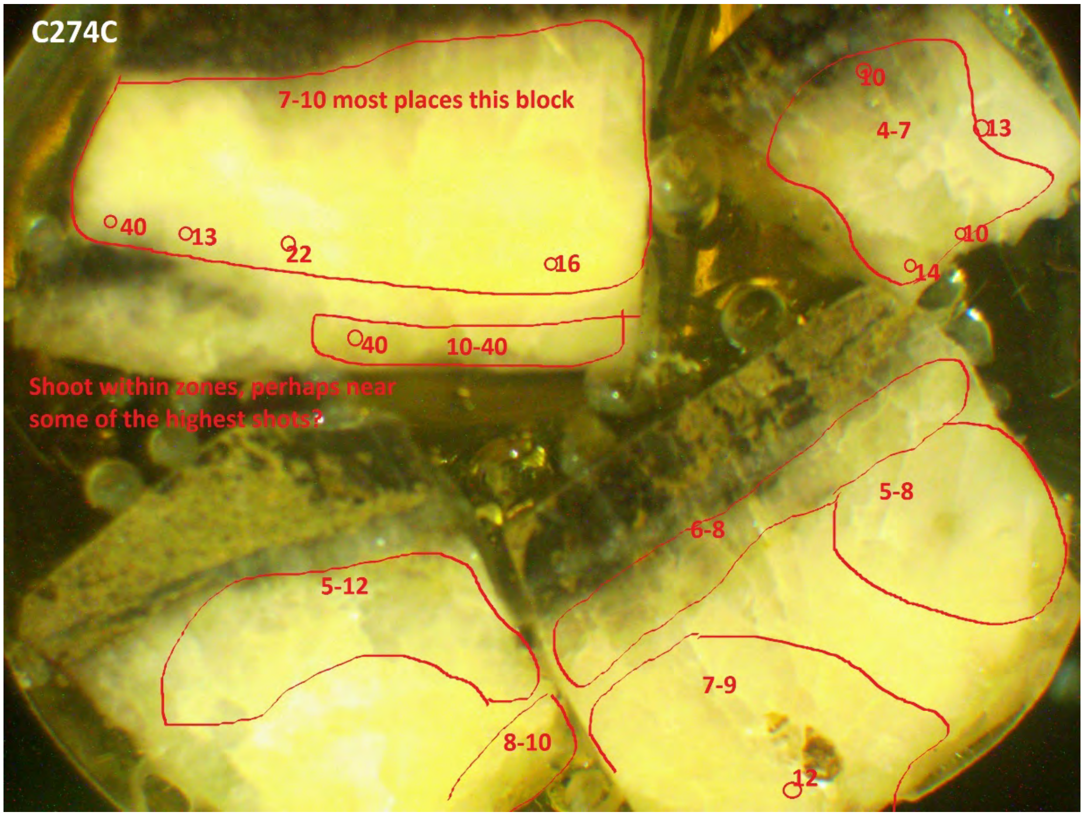

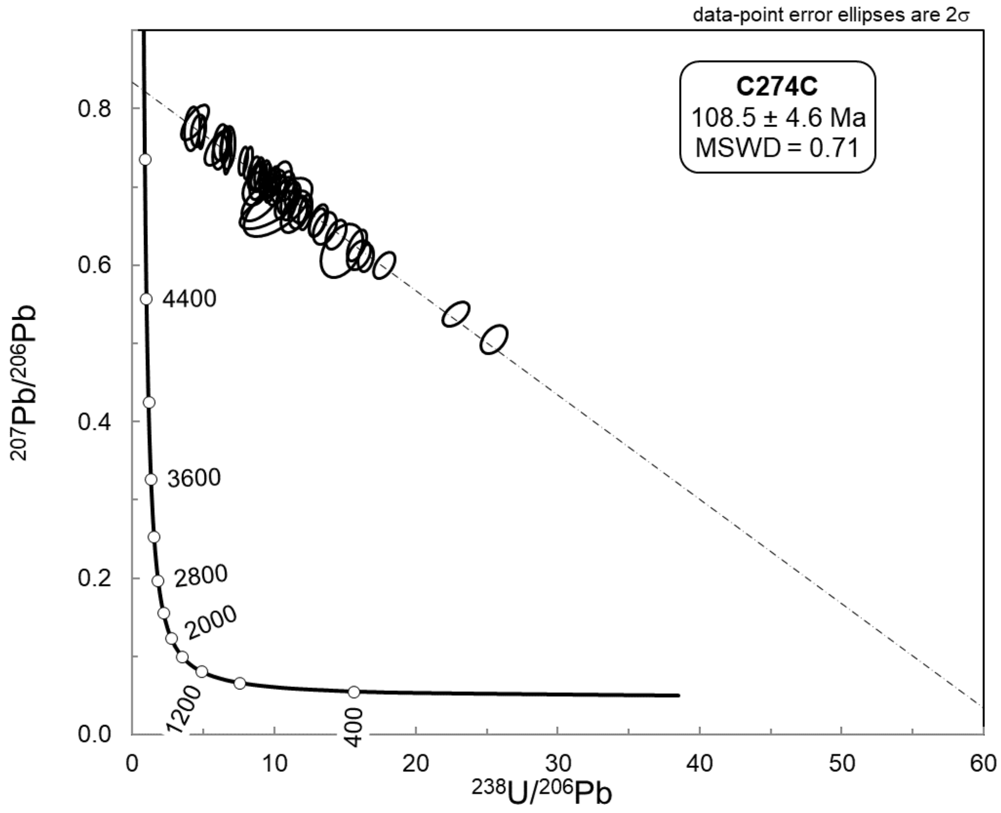

Supplement: Supplementary file 6 — Source Data [file 41467_2022_29680_MOESM6_ESM.zip › 2.zip!/appendix2b_site_summaries_21to50/22_porter_bay_final.pdf]
