# Supplementary material for: Pace of passive margin tectonism revealed by U-Pb dating of fracture-filling calcite
Source: Nat Commun. 2022 Apr 12;13:1953. doi: 10.1038/s41467-022-29680-z (PMC9005700; doi:10.1038/s41467-022-29680-z)

23: Carleton's Prize Island, Lake Champlain (44.604649 -73.346538)

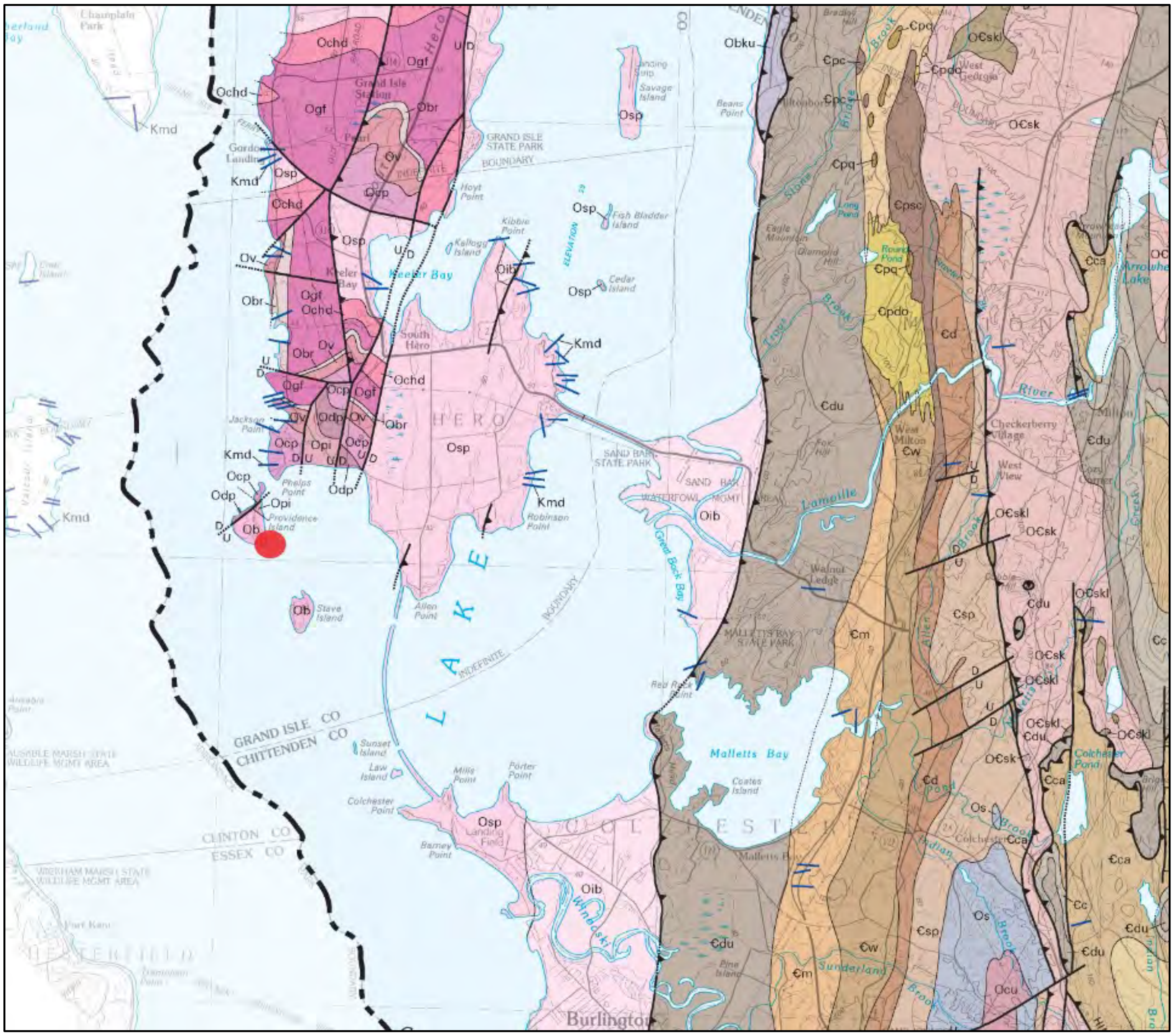

Figure 1: Sample location on state geologic map of Ratcliffe at al. (2011).

<u>Failed samples from same site:</u> C283B,D,E,F,G *(E, F, G have some potential!)*

<u>Bedrock Unit:</u> Bascom Formation (Ob)

<u>Ages</u>: C283A: 431 ± 13 Ma; **C283C:** 438 ± 16 Ma; **C283H:** 441 ± 11 Ma (44.604649, -73.346538)

Summary: This is a suite of sites on Carleton's Prize Island, about 1.5 miles south of South Hero, Vermont. Access is only by boat and is possible from a launch on the south shore of South Hero island. The island is dominated by a complex N-S trending fault zone with accessory faults and mineralized breccias filled with calcite and reddish-brown fe-oxide(?) mineral. Deposition was accompanied by voluminous fluid flow. U-Pb ages point to all structures being Taconic in age, but we have not even begun to unravel the structural complexity of the island. This island would be an excellent location for a follow up study on Taconic tectonism Three additional calcite samples (E,F, and G) show promise for dating and need to be revisited or just shipped to UCSB in a final round.

Field Descriptions:

C283A:

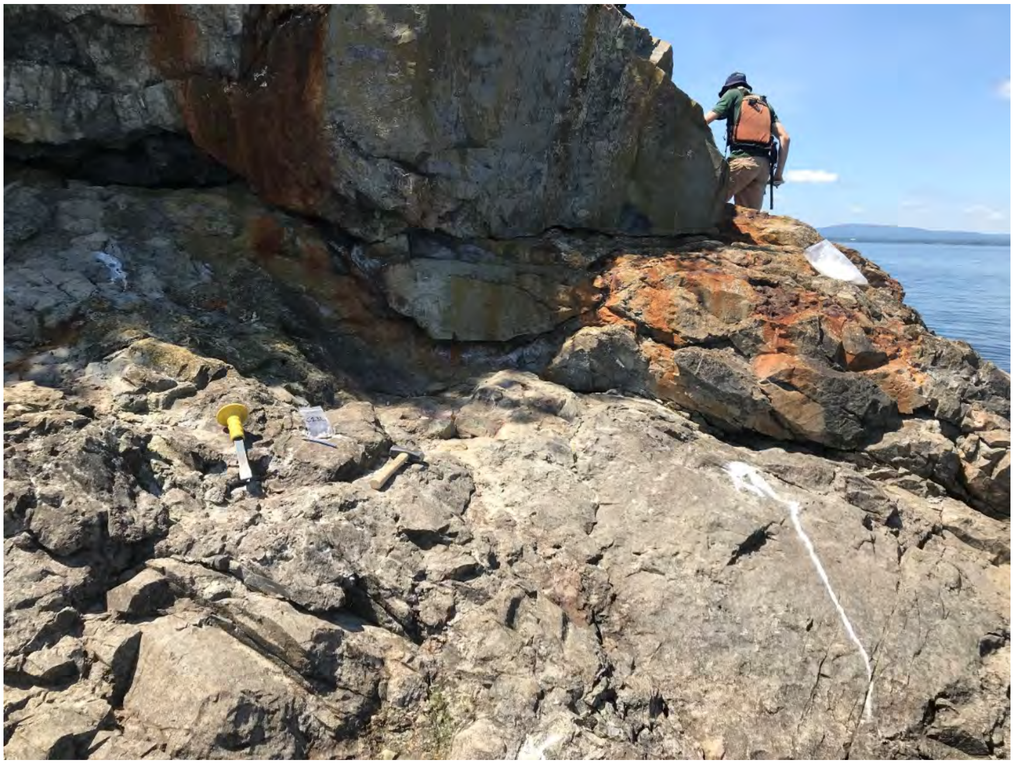

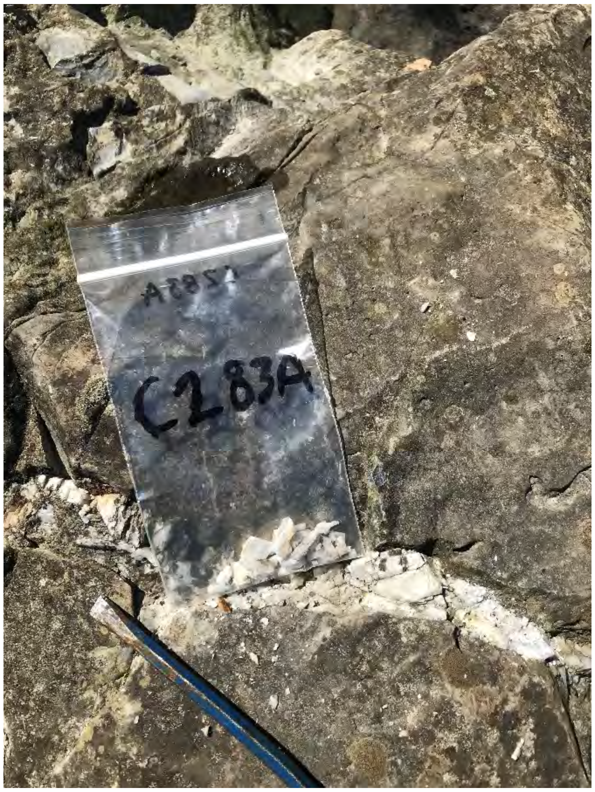

C283C:

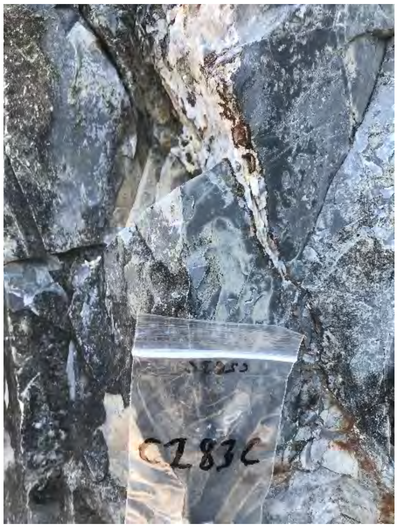

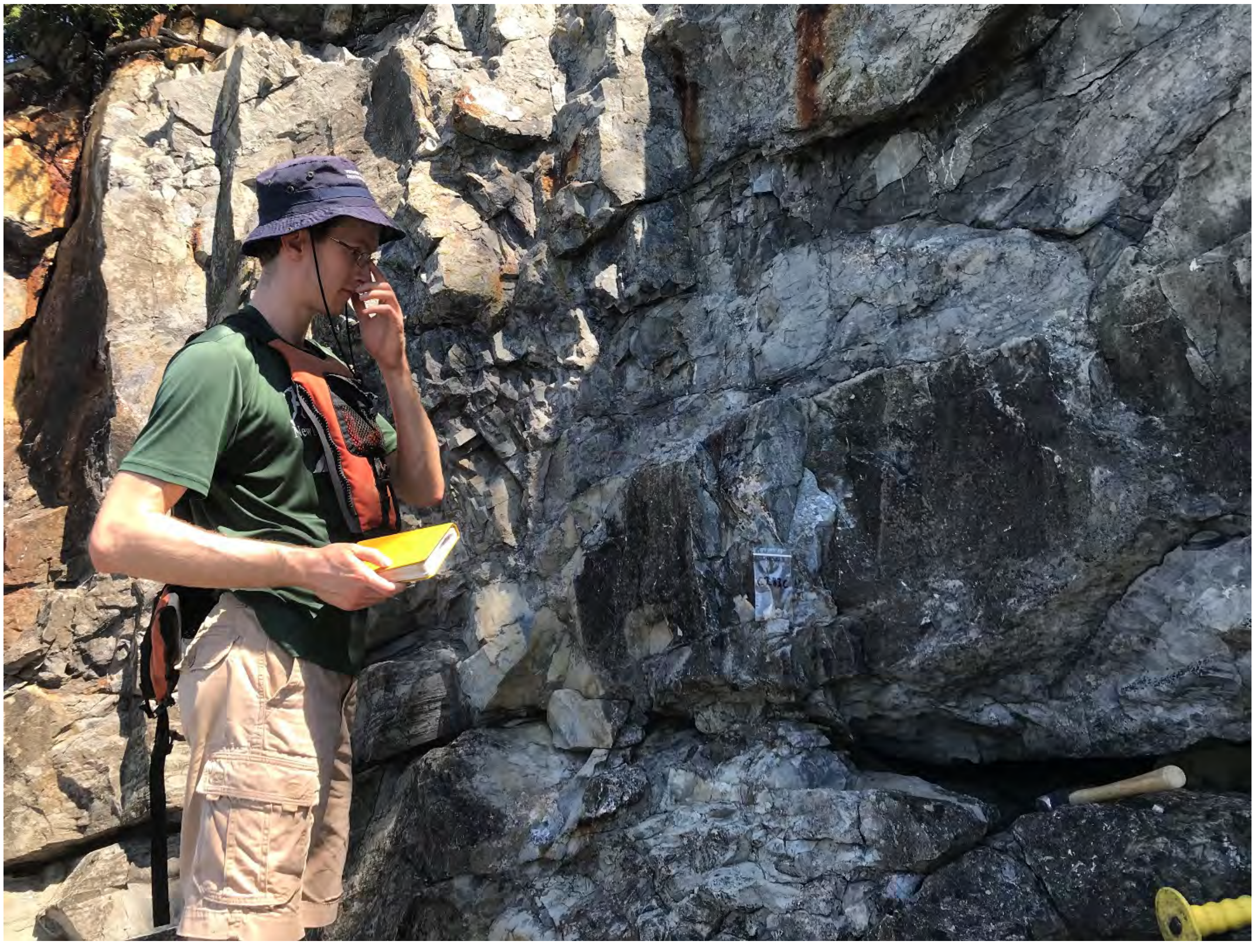

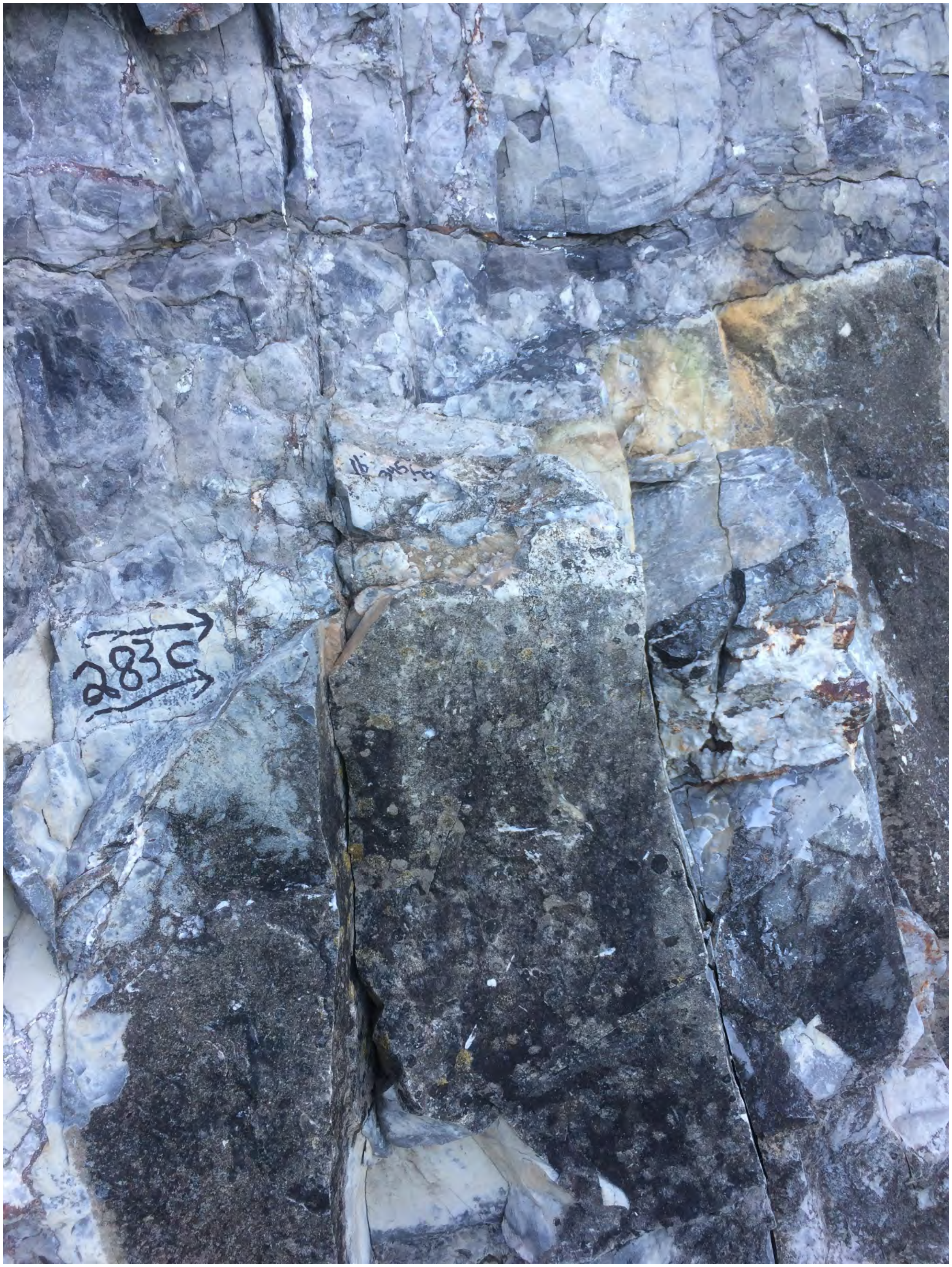
283C

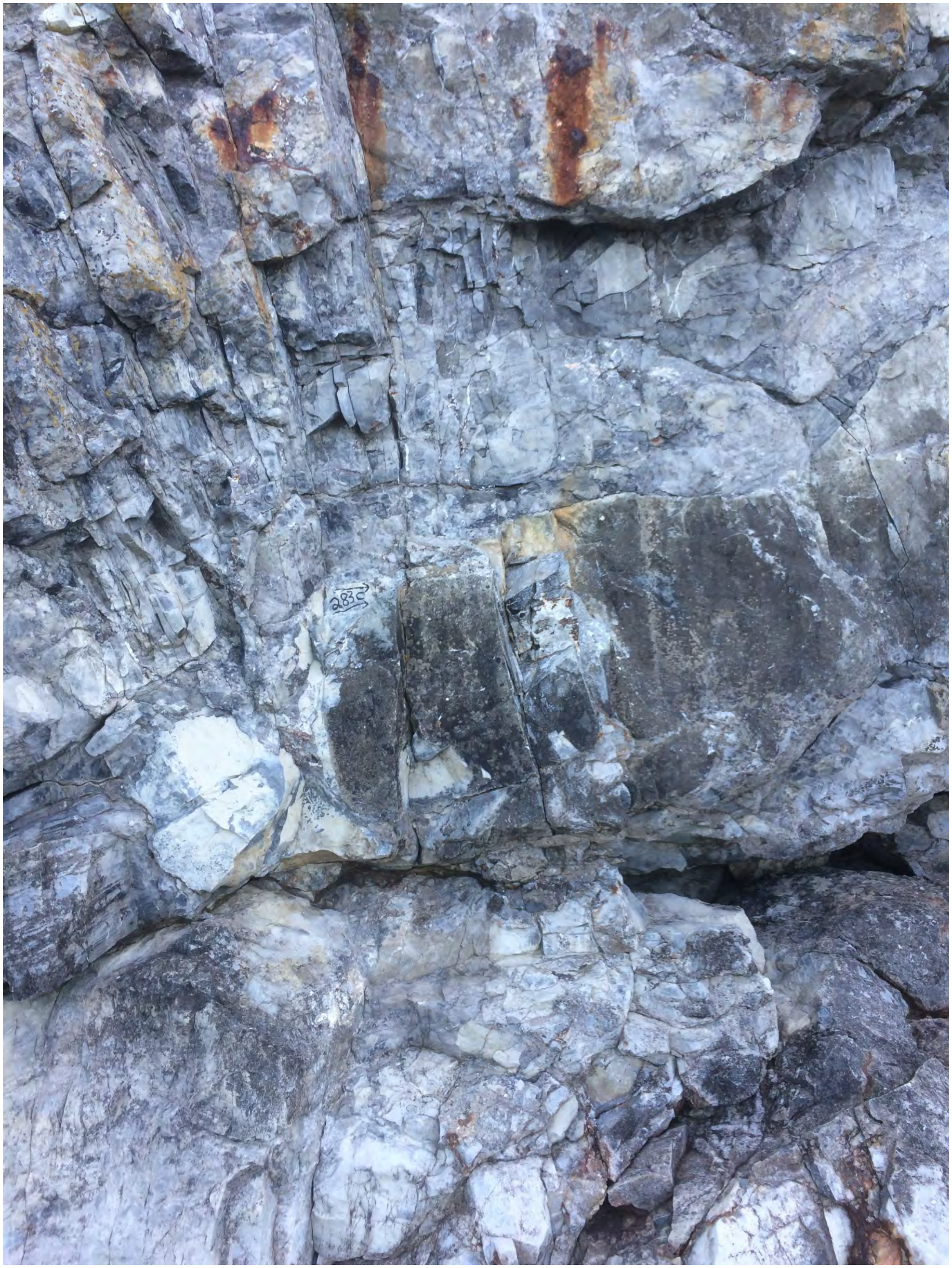
283C

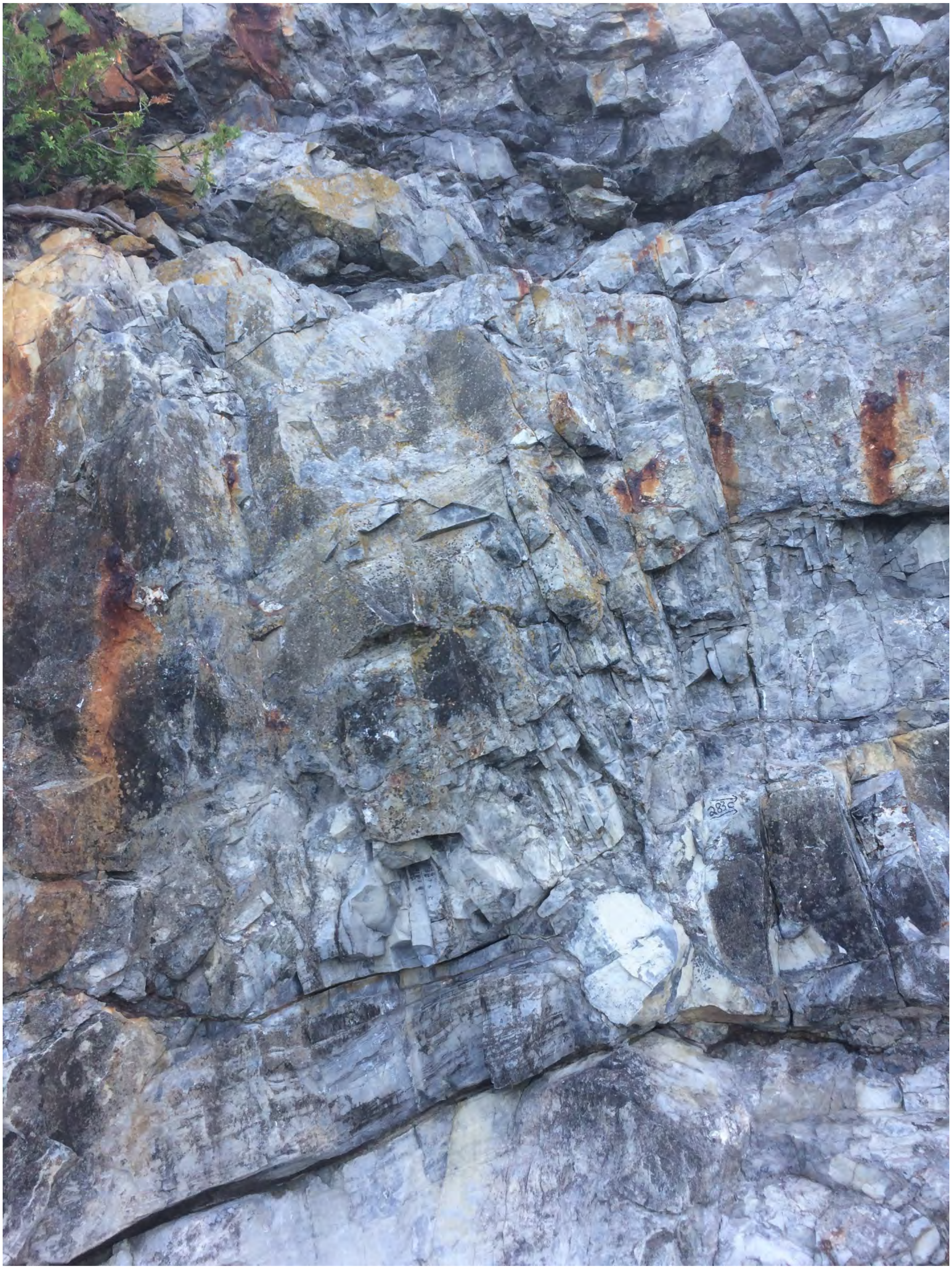

C283H:

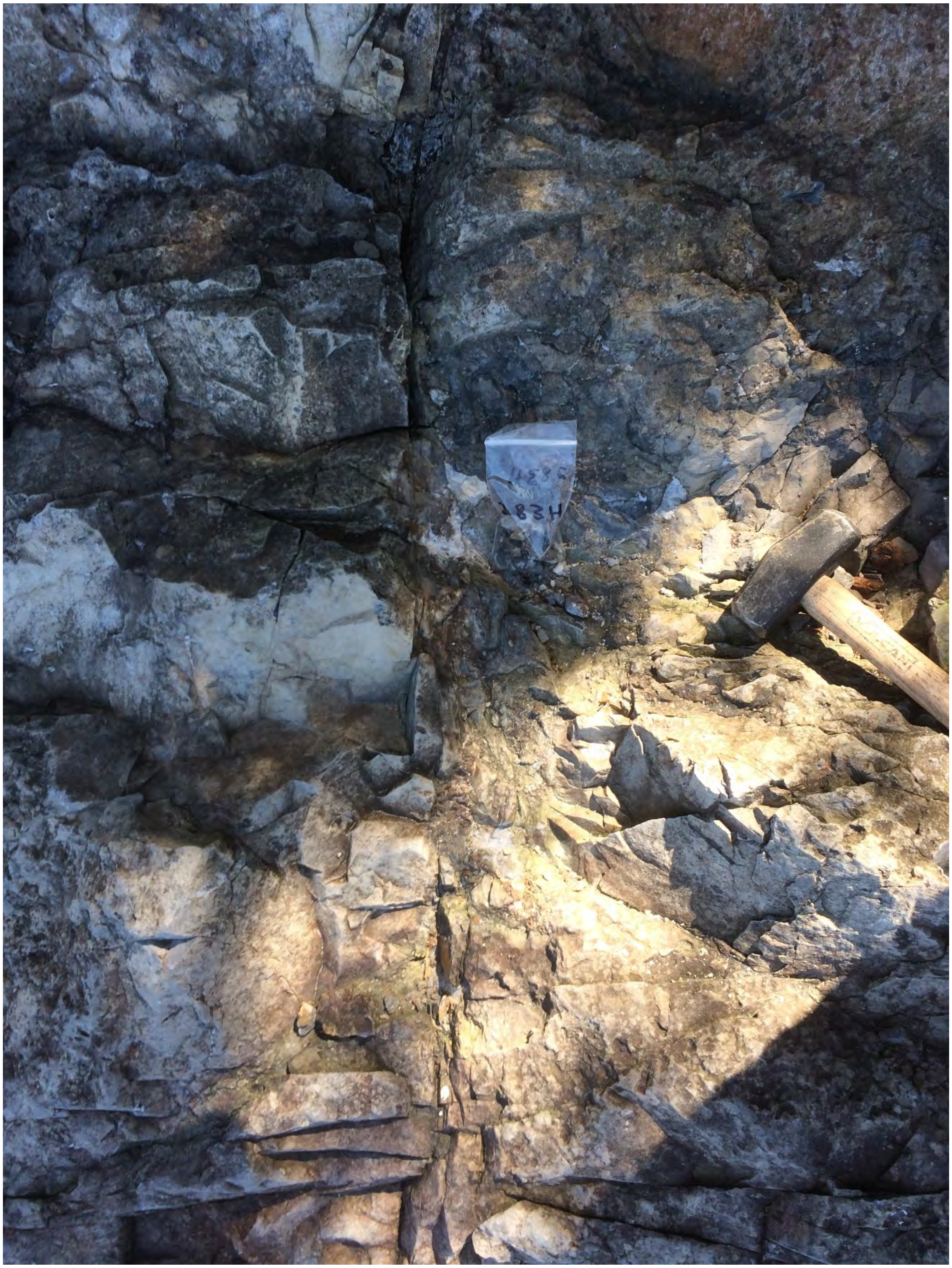

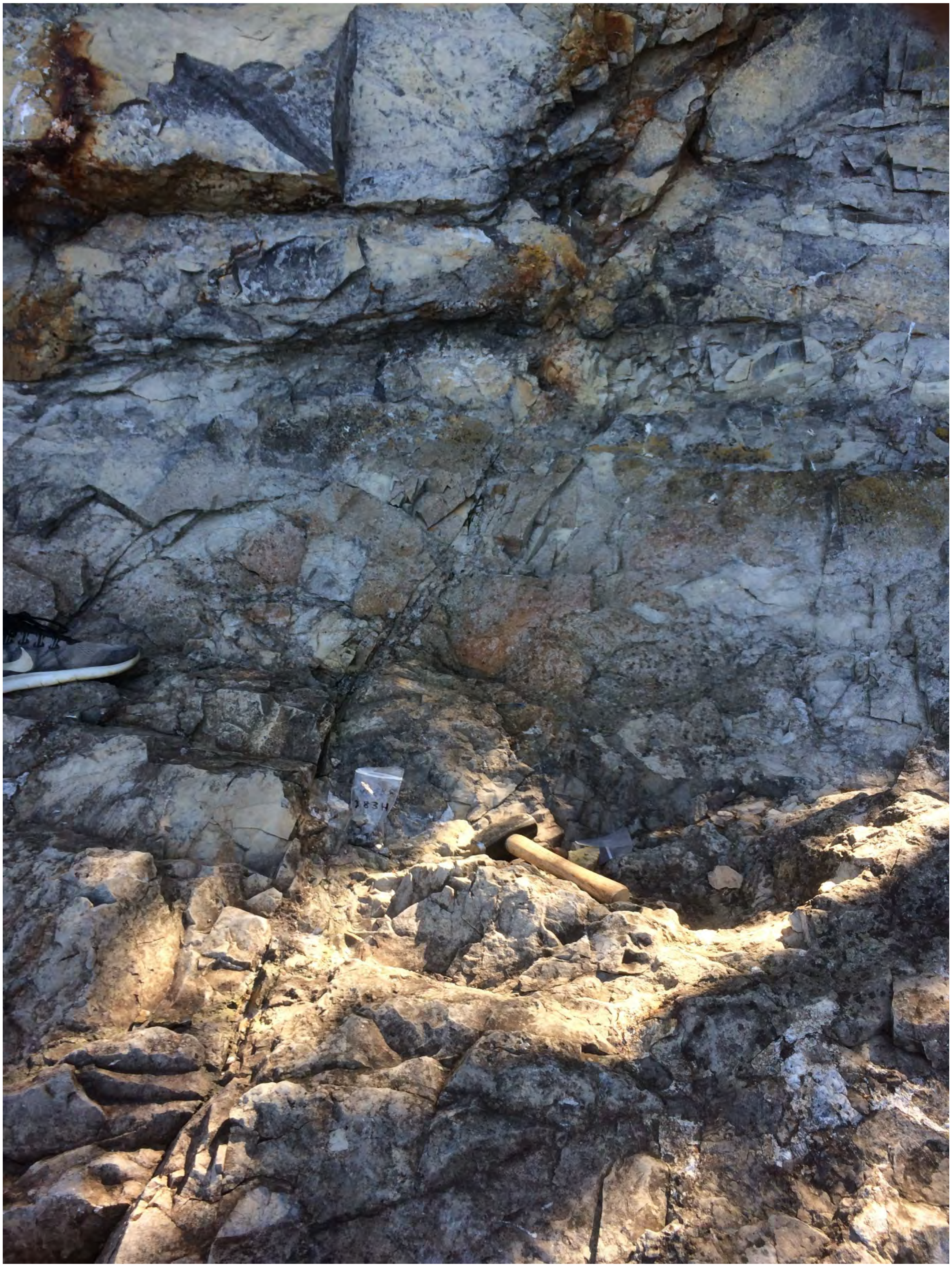

Lab Description:

C283A: Circles on image denote areas of interest, not laser shots

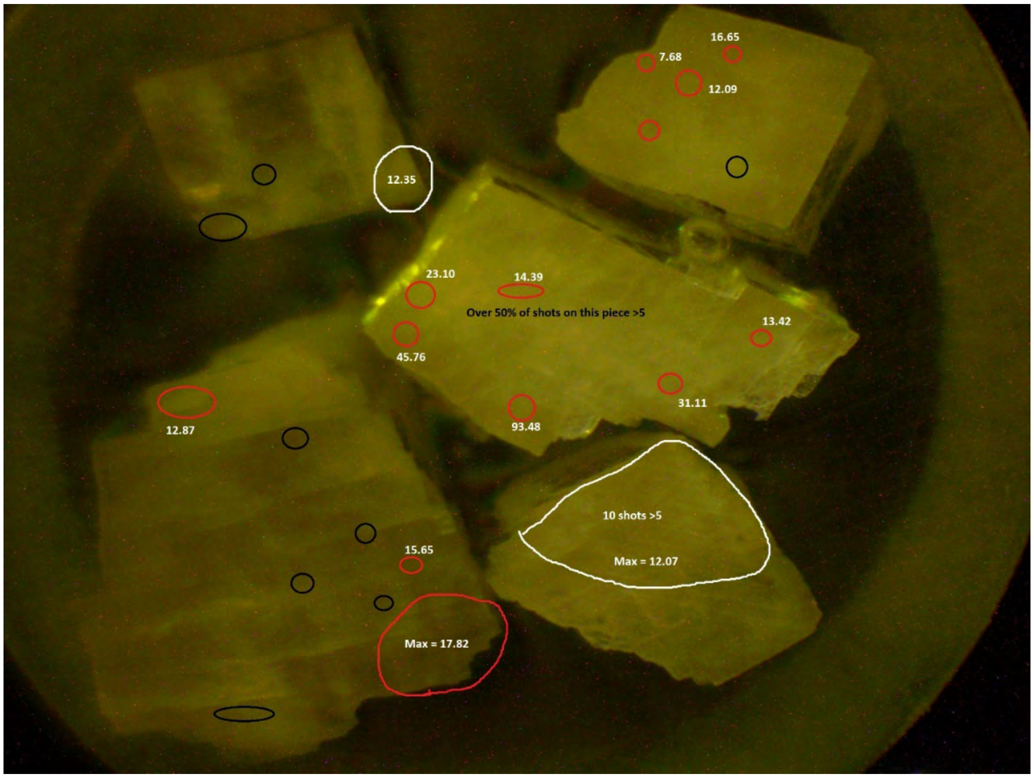

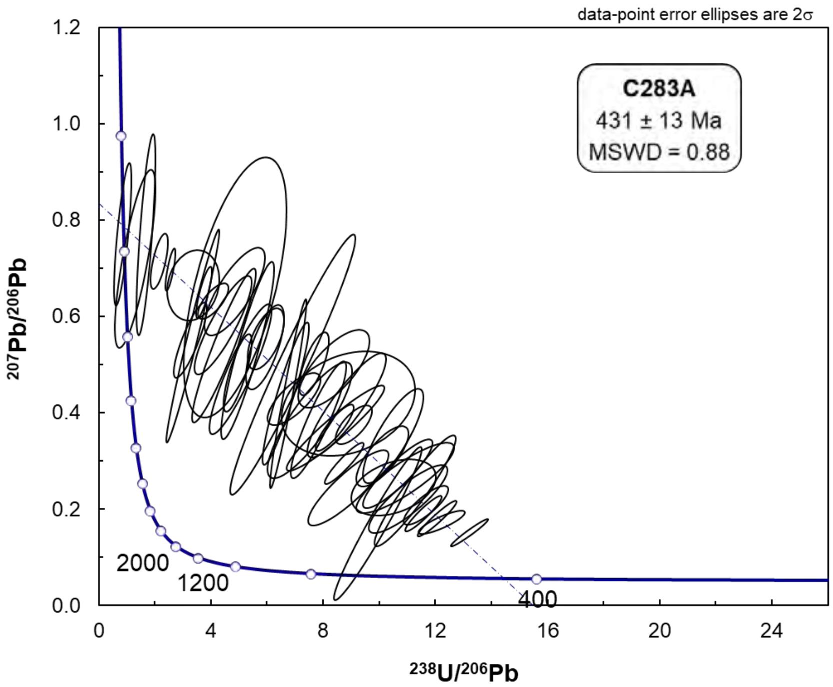

C283C: Circles on image denote areas of interest, not laser shots

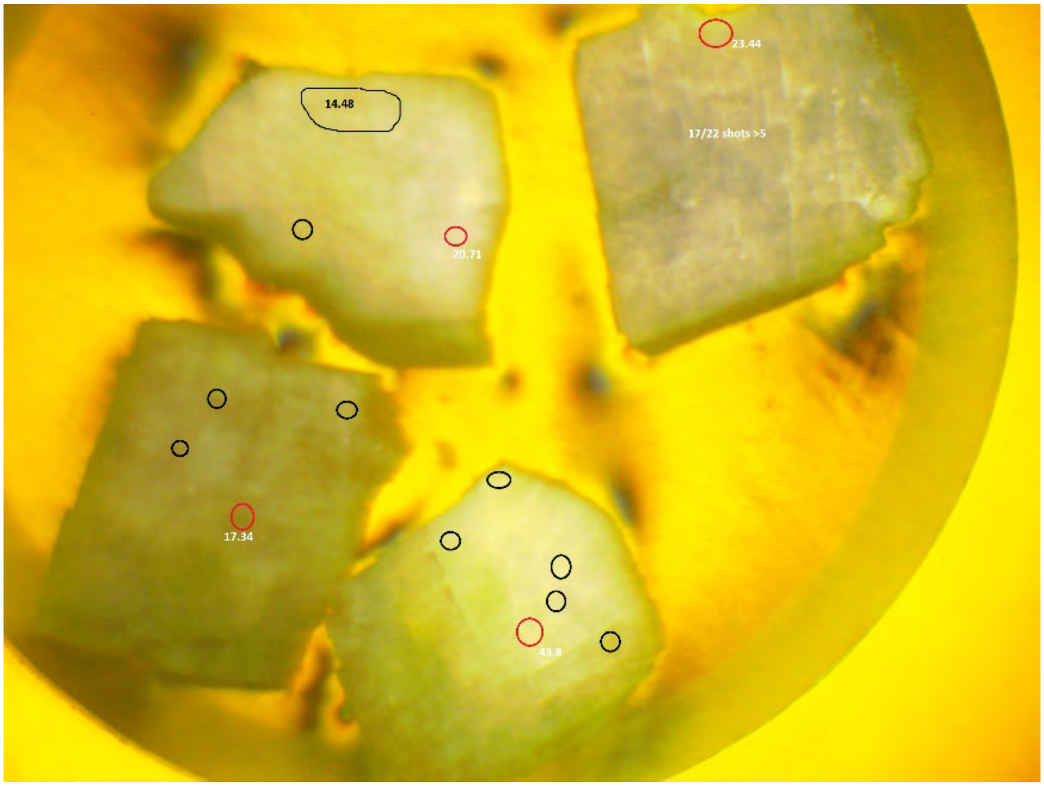

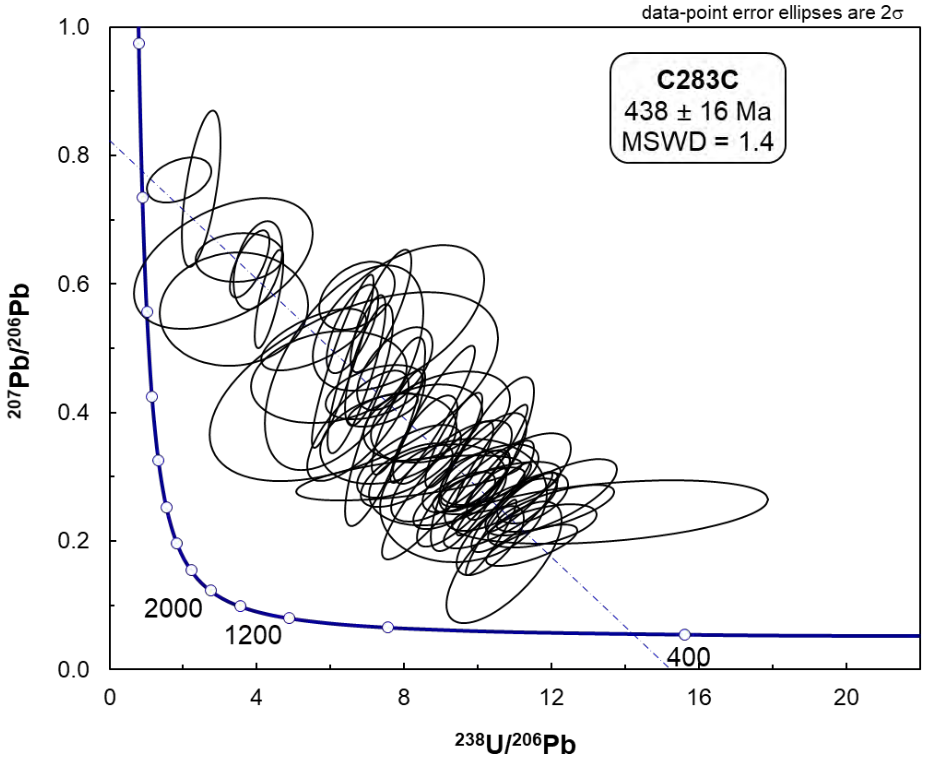

C283H: Circles on image denote areas of interest, not laser shots

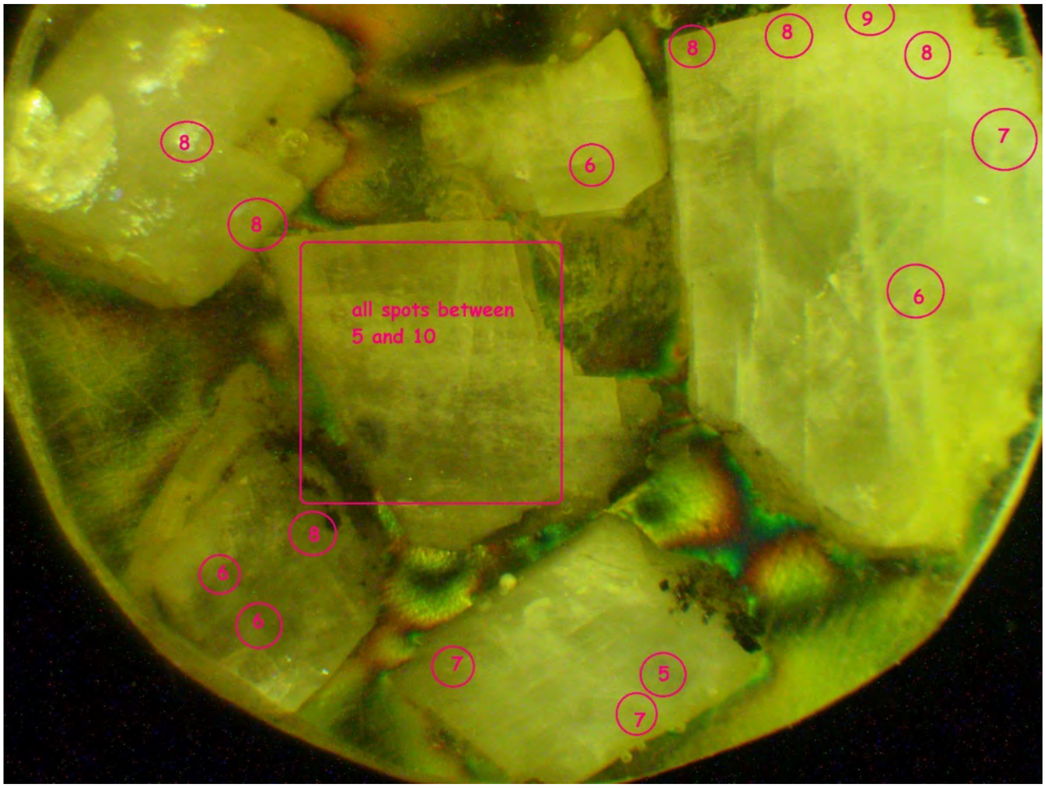

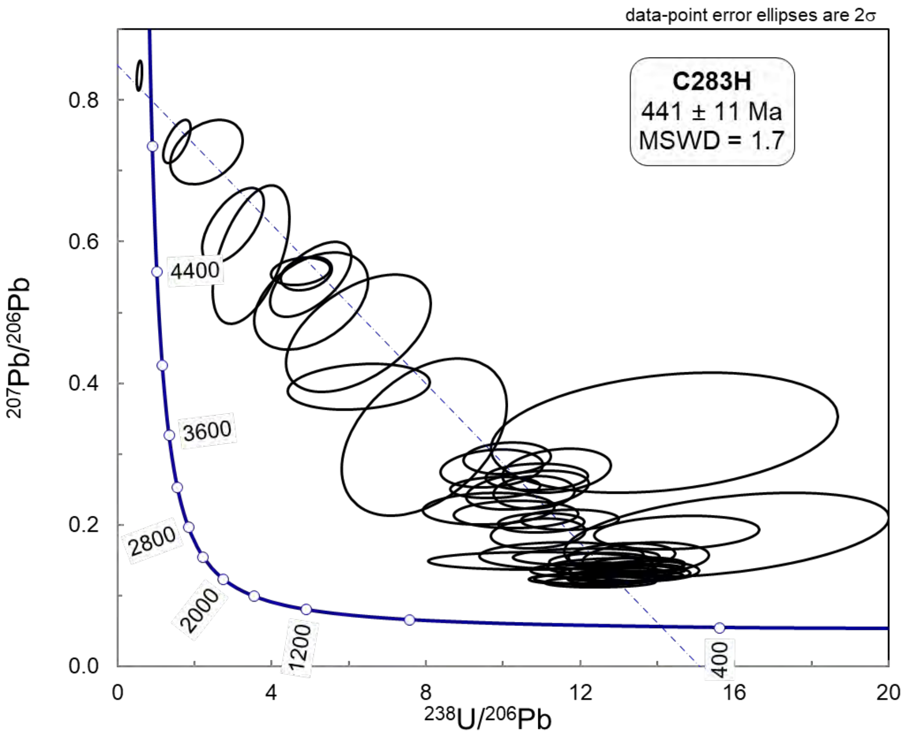

Supplement: Supplementary file 6 — Source Data [file 41467_2022_29680_MOESM6_ESM.zip › 2.zip!/appendix2b_site_summaries_21to50/23_carletons_prize_island_final.pdf]
