# Supplementary material for: Pace of passive margin tectonism revealed by U-Pb dating of fracture-filling calcite
Source: Nat Commun. 2022 Apr 12;13:1953. doi: 10.1038/s41467-022-29680-z (PMC9005700; doi:10.1038/s41467-022-29680-z)

24: Providence Island East (44.6167, -73.3502)

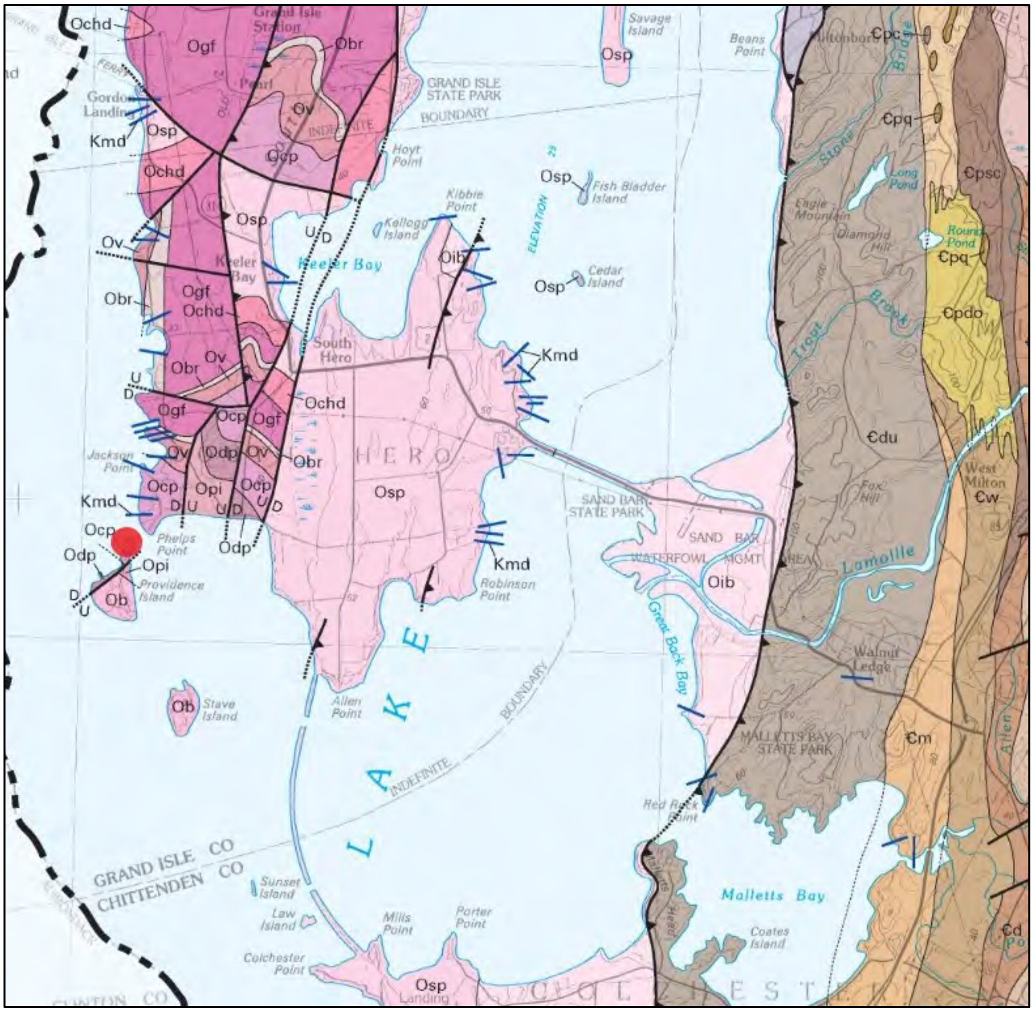

Figure 1: Sample location on state geologic map of Ratcliffe at al. (2011).

<u>Failed samples from same site:</u> none

<u>Bedrock Unit:</u> Crown Point Formation (Ocp)

<u>Ages</u>:   **C284**: 43 +/- 13 Ma

<u>Summary</u>: This site is located on the northernmost part of the eastern side of Providence Island. It can only be reached by boat, ideally from the launch on the southern shore of South Hero. Sample taken from an isolated thin vertical vein in bedrock striking approximately 300 (no measurement on surface possible). Vein lacks slip indicators and is interpreted as a mode 1 dilational structure with σ_3 stress 30°. No visible faulting or slickensides or evidence of offset.   Single-domain isochron age and lack of complex textures suggests this is a primary vein and not recrystallized. The rare Eocene age suggests this could have formed during the same event as samples at site 18, the Port Henry, NY campground.

Field Observations:

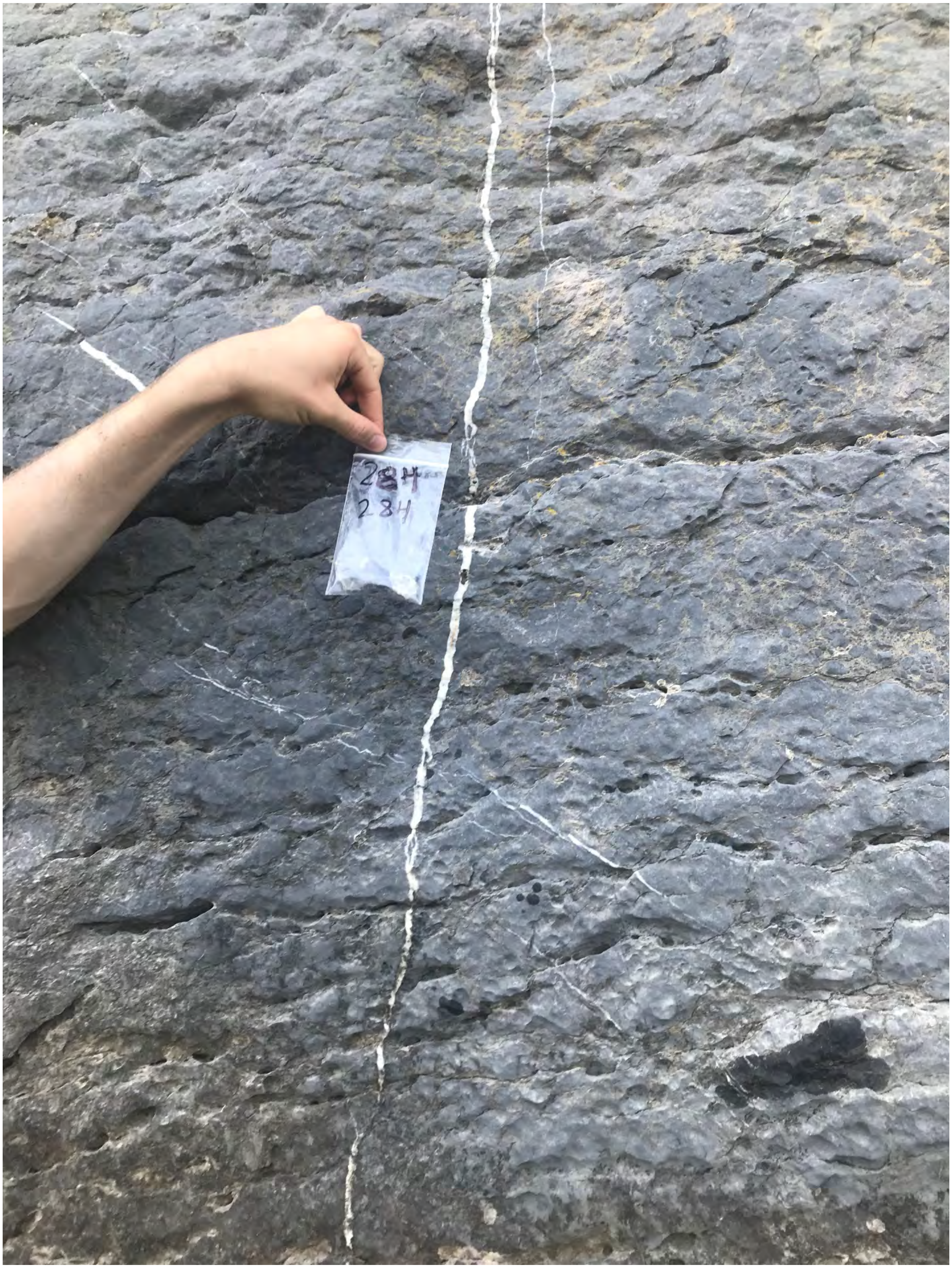

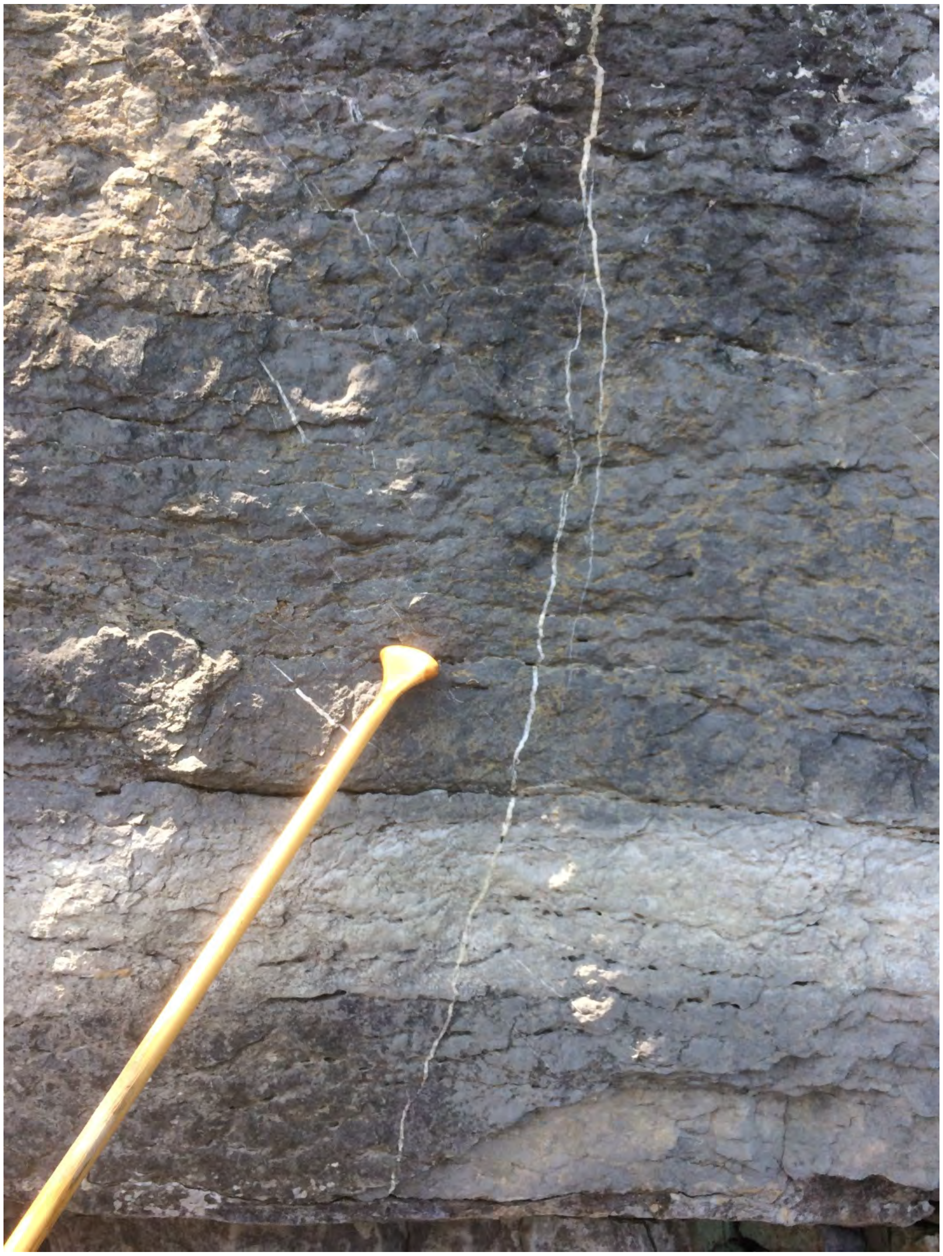

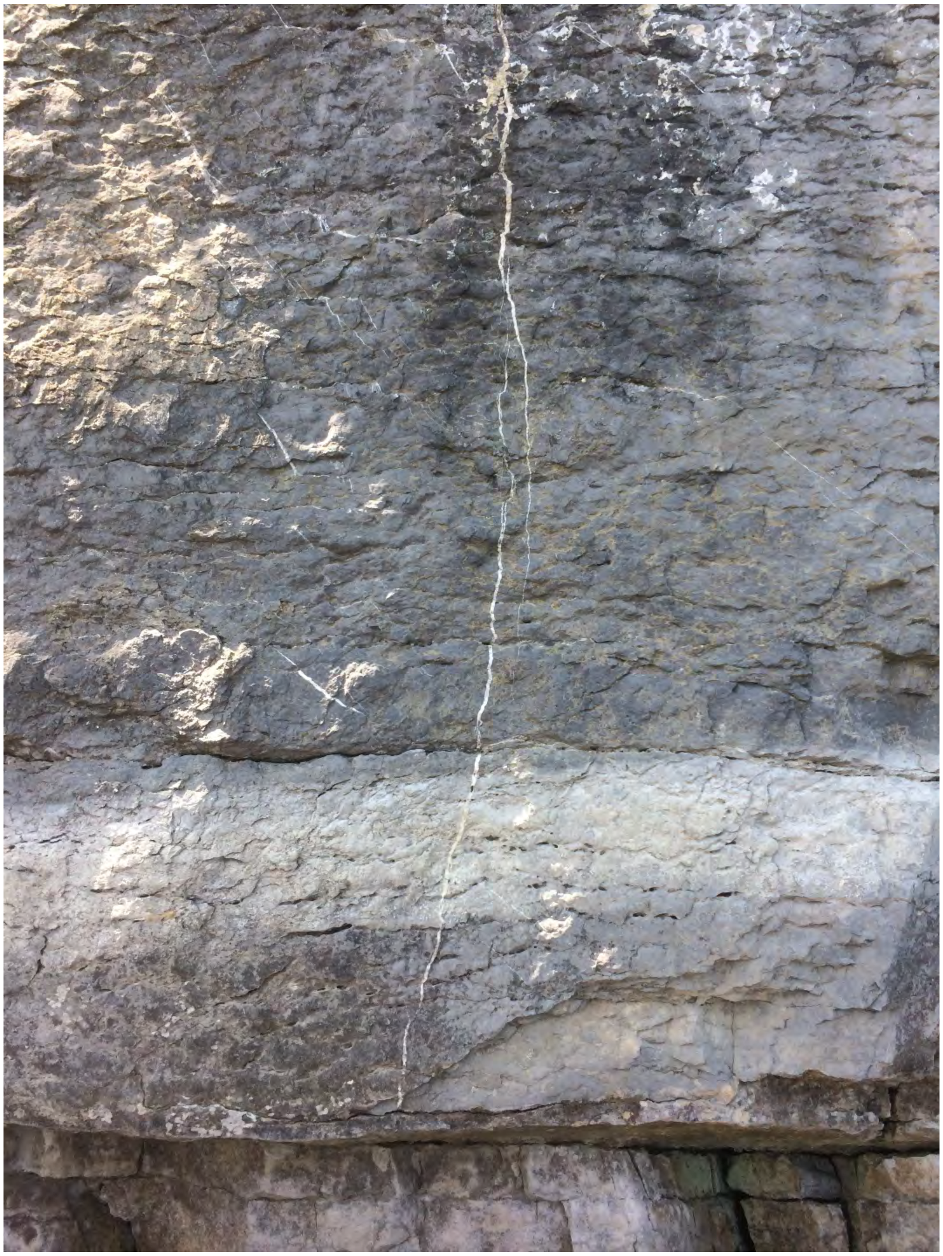

Lab Description: Circles denote areas of interest for dating

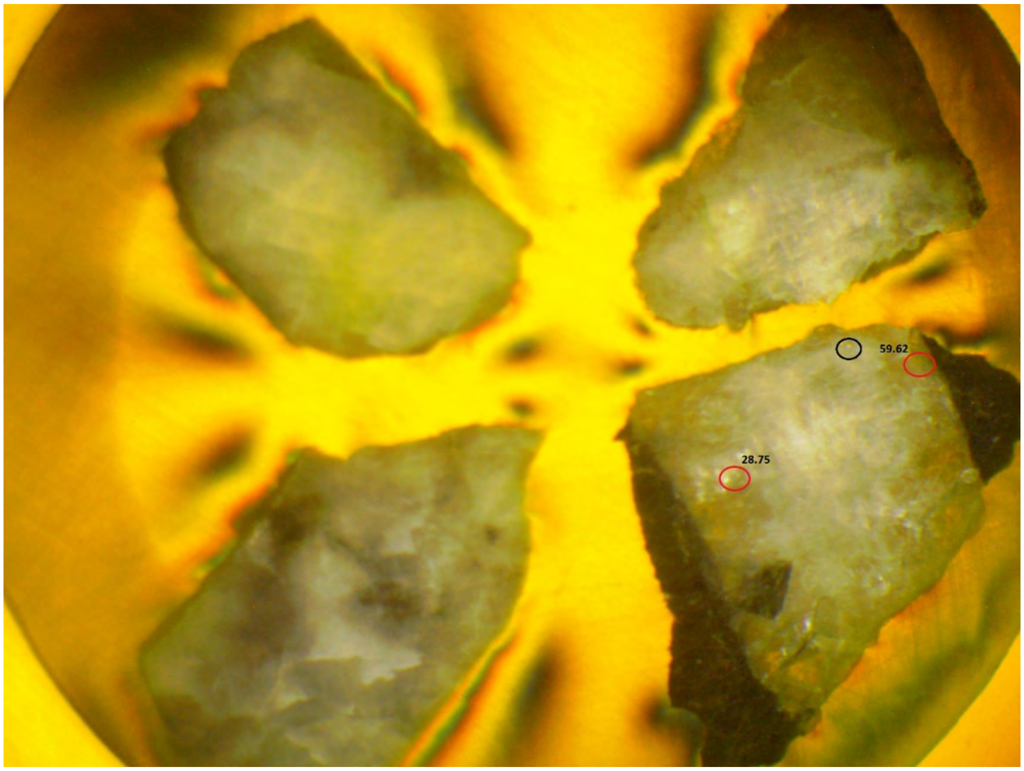

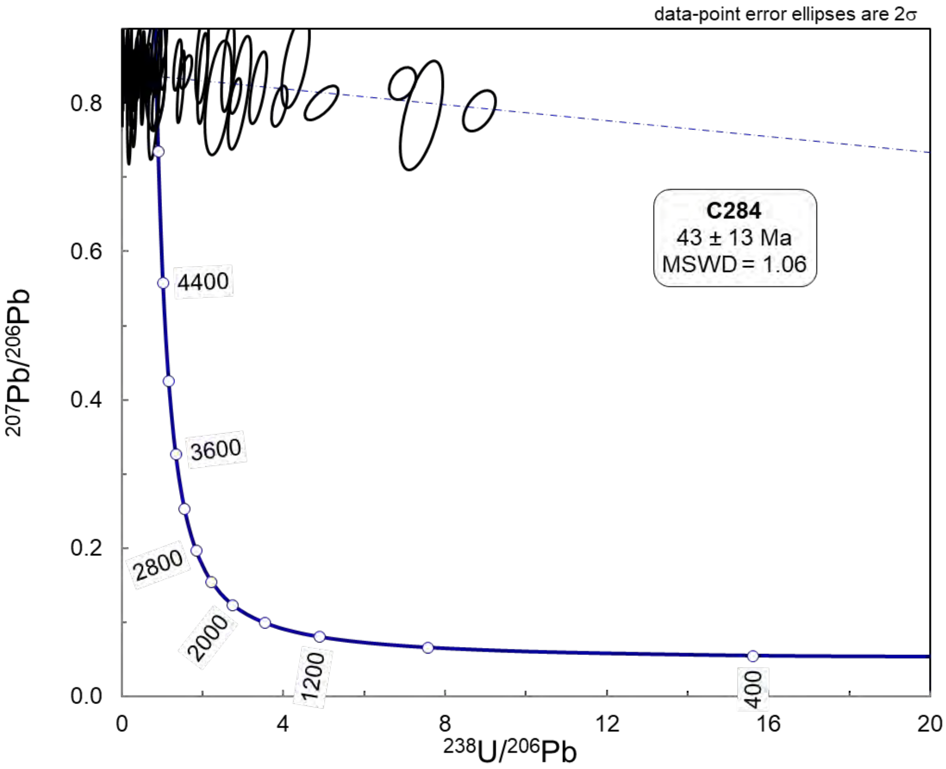

Supplement: Supplementary file 6 — Source Data [file 41467_2022_29680_MOESM6_ESM.zip › 2.zip!/appendix2b_site_summaries_21to50/24_providence_island_east_final.pdf]
