# Supplementary material for: Pace of passive margin tectonism revealed by U-Pb dating of fracture-filling calcite
Source: Nat Commun. 2022 Apr 12;13:1953. doi: 10.1038/s41467-022-29680-z (PMC9005700; doi:10.1038/s41467-022-29680-z)

25: Bennington Plastics, Bennington, VT (42.921253, -73.247229)

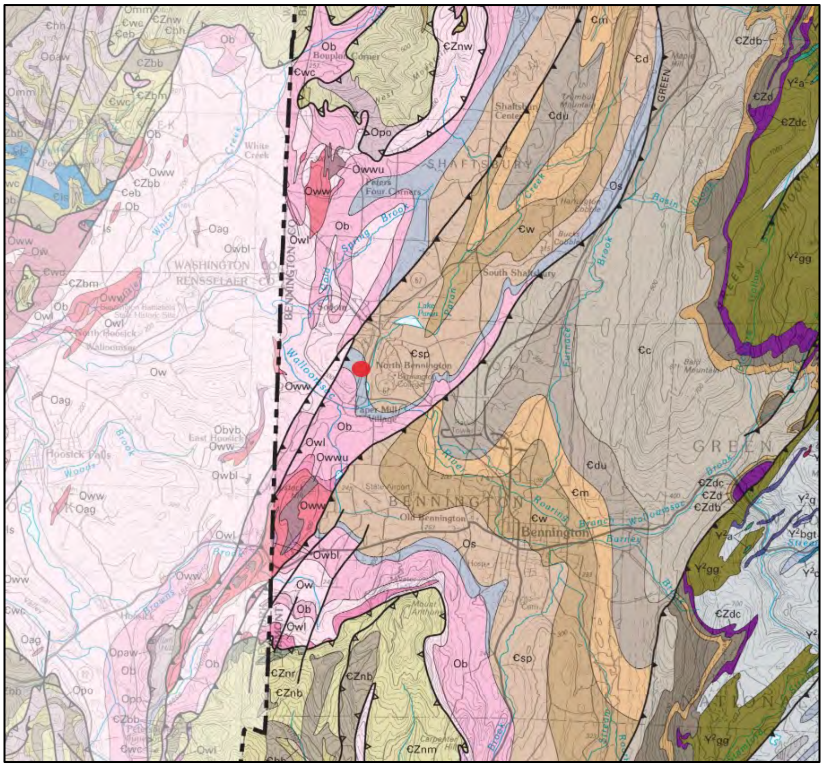

Figure 1: Sample location on the VT geologic map (Ratcliffe et al., 2011)

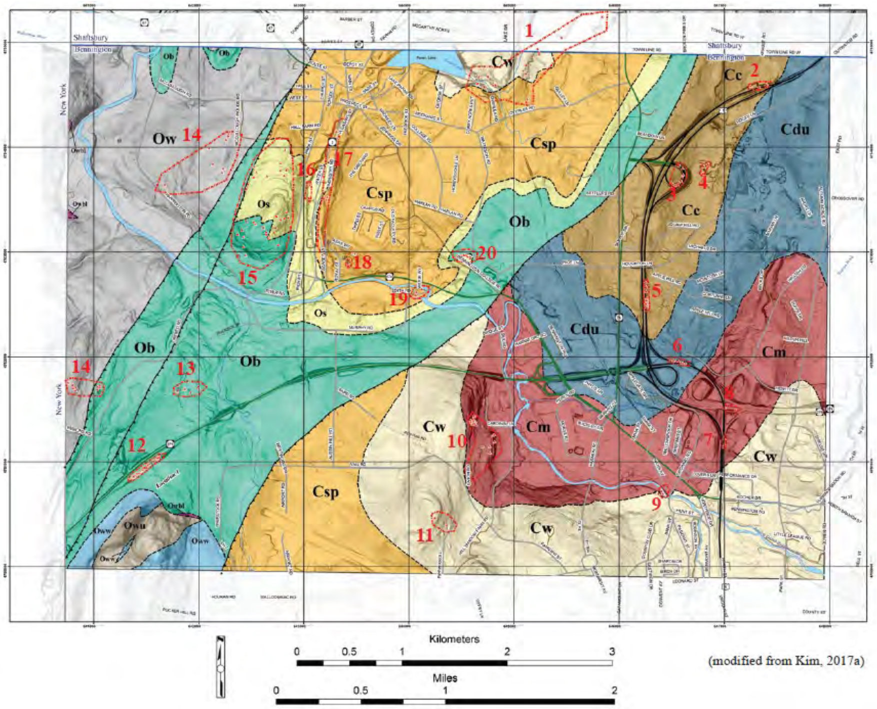

Figure 2: Sample collected from domain 16 on this map of Kim et al. (2017). Reference: VG2017-4B Kim, J. J., 2017, Preliminary Fracture Map of the Bennington Area, Vermont: Vermont Geological Survey Open File Report VG2017-4B, scale 1:12,000.

Failed samples from same site: 72018-18,-19,-22,-23,-24 (C303A-B, C304B-C, C305, C306)

Ages: **C302A:** 87.5 ± 2.5 Ma **C304A:** 111.6 ± 1.6 Ma

Bedrock Unit: Shelburne Marble (Os)

Summary: This site is located behind the Bennington Plastics factory on the west side of Route 67A about ½ mile north of the Walloomsac River. It can be accessed directly by car. At least two events are recorded in a set of mostly N-S fractures running parallel to the river valley. The veins dated here are part of a family of mostly vertical N-S oriented veins and fractures which are widely observed in the Bennington area as described in the work of Jon Kim and other at the Vermont Geological Survey, for example: *VG2017-4B Kim, J. J., 2017, Preliminary Fracture Map of the Bennington Area, Vermont: Vermont Geological Survey Open File Report VG2017-4B, scale 1:12,000.* Neither set has slickensides or trans-tensional stepovers so they are interpreted as mode 1 tensional fractures recording periods of primarily E-W tensile stress at ~112 and 88 Ma. Sample C302A has a strike/dip of 13°/83°E, whereas sample C304A is oriented at 0°/66°E. As shown in photos below, sample C304A is thicker and connected with very massive sparry nodules suggesting abundant fluids at this time. The isochron ages are high quality and reliable, although sample C302A has a cluster of low $^{238}U/^{206}Pb$ shots that are are excluded from the isochron fit.

Field Observations:

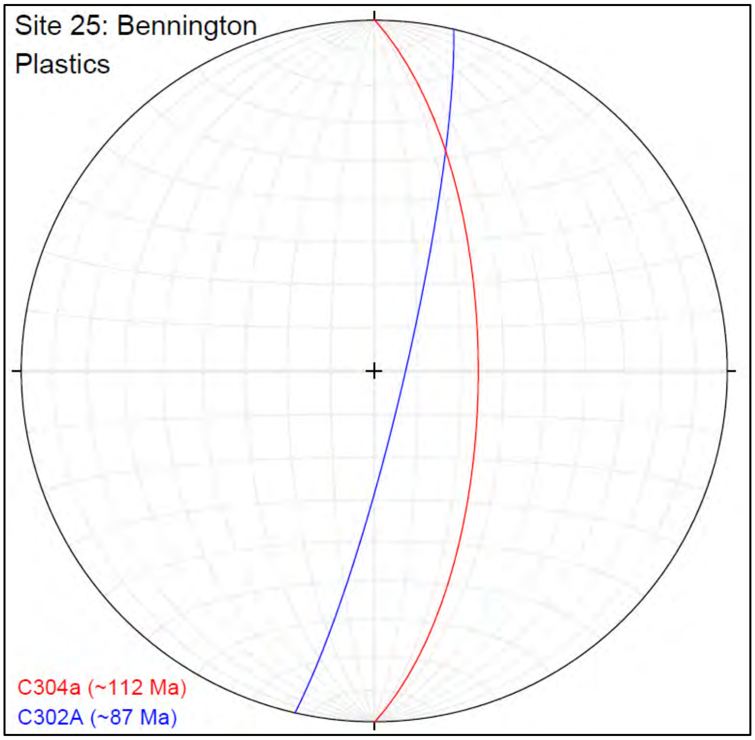

Figure 3 Orientations of two dated veins at site 25

C302A (labeled as 17):

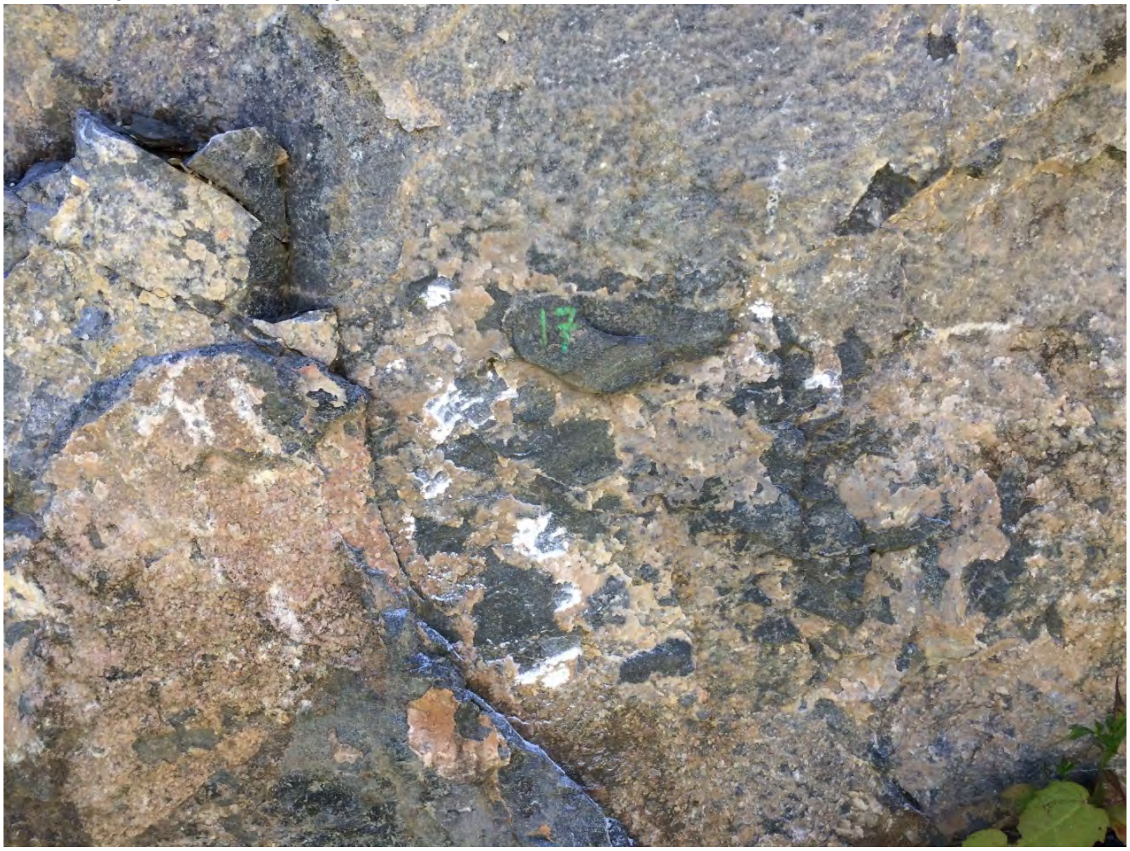

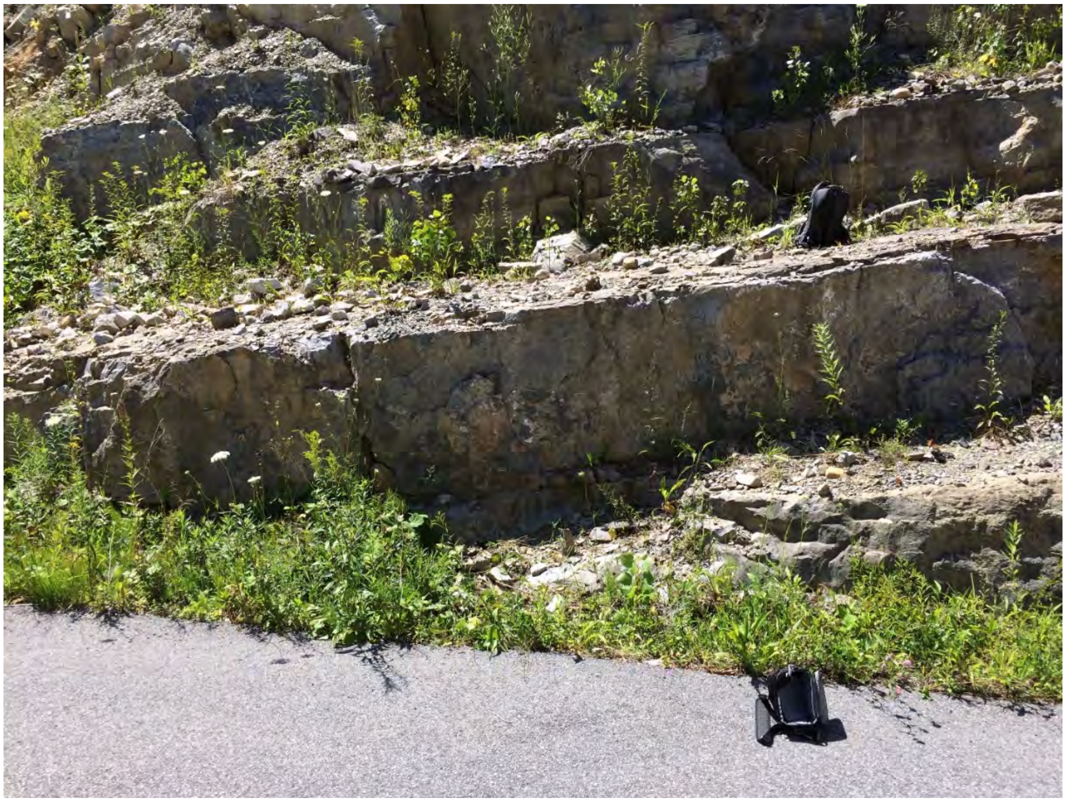

C304A (labeled as 20): (all photos looks vertically down on vein from ledge)

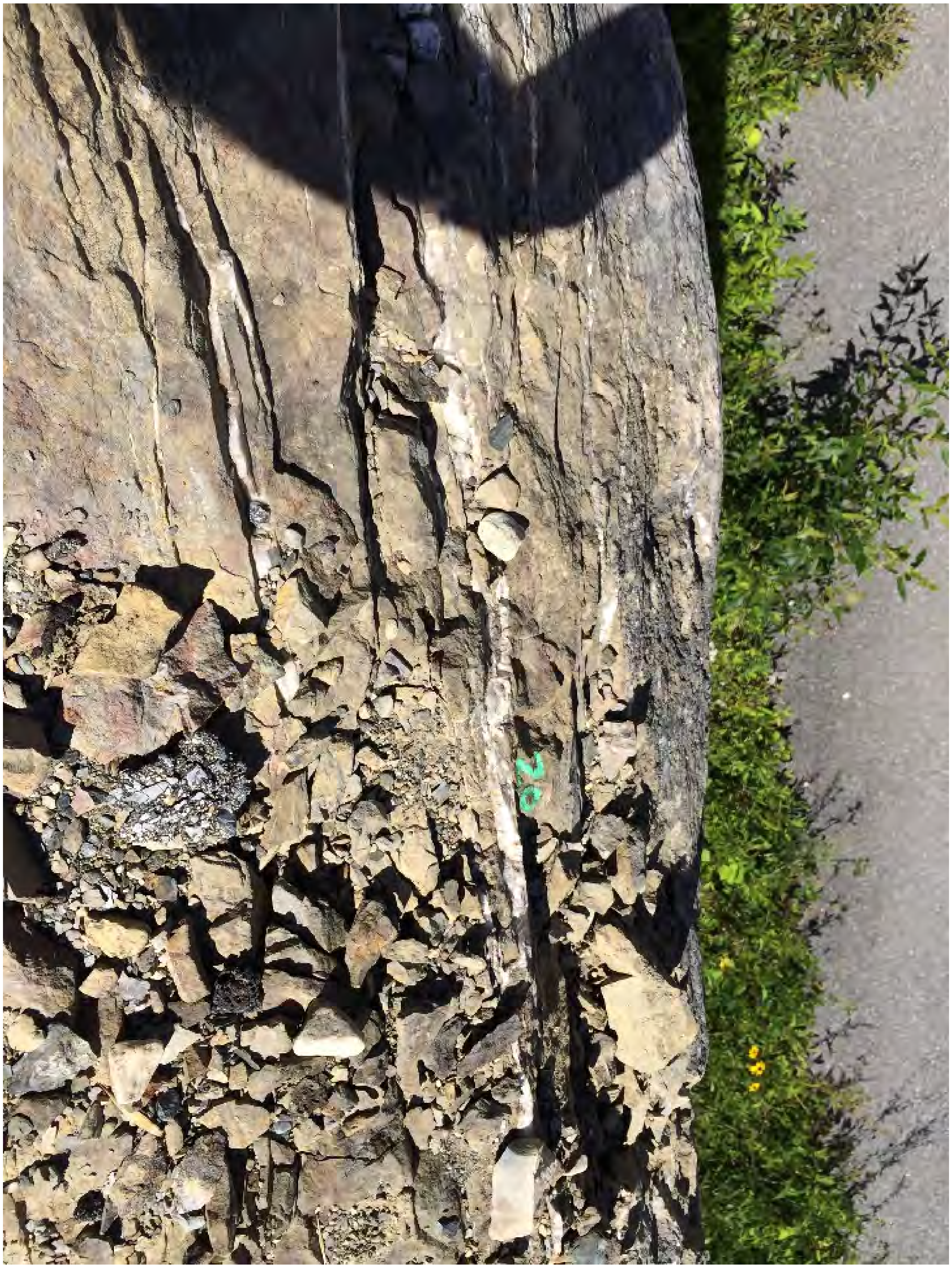

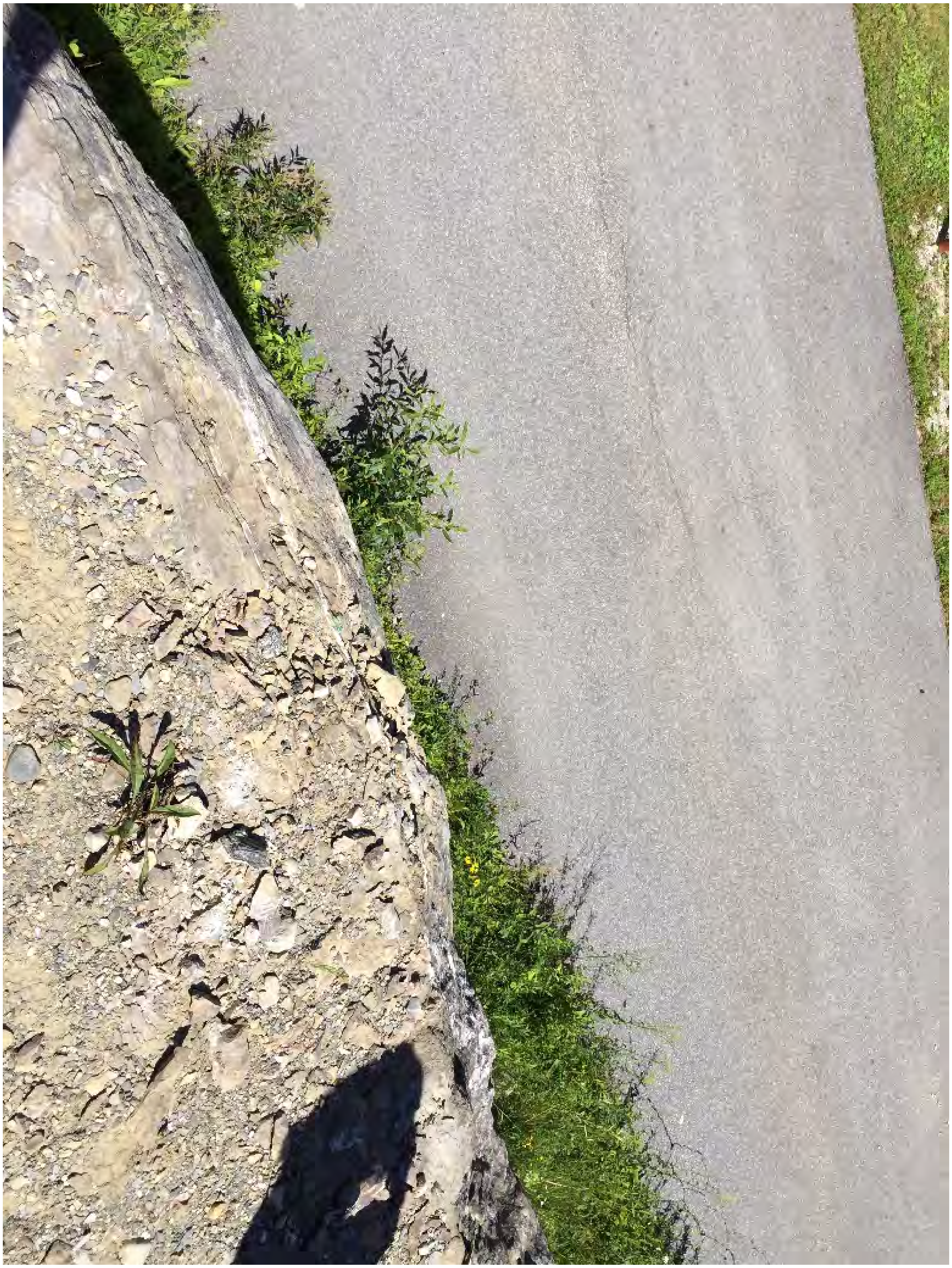

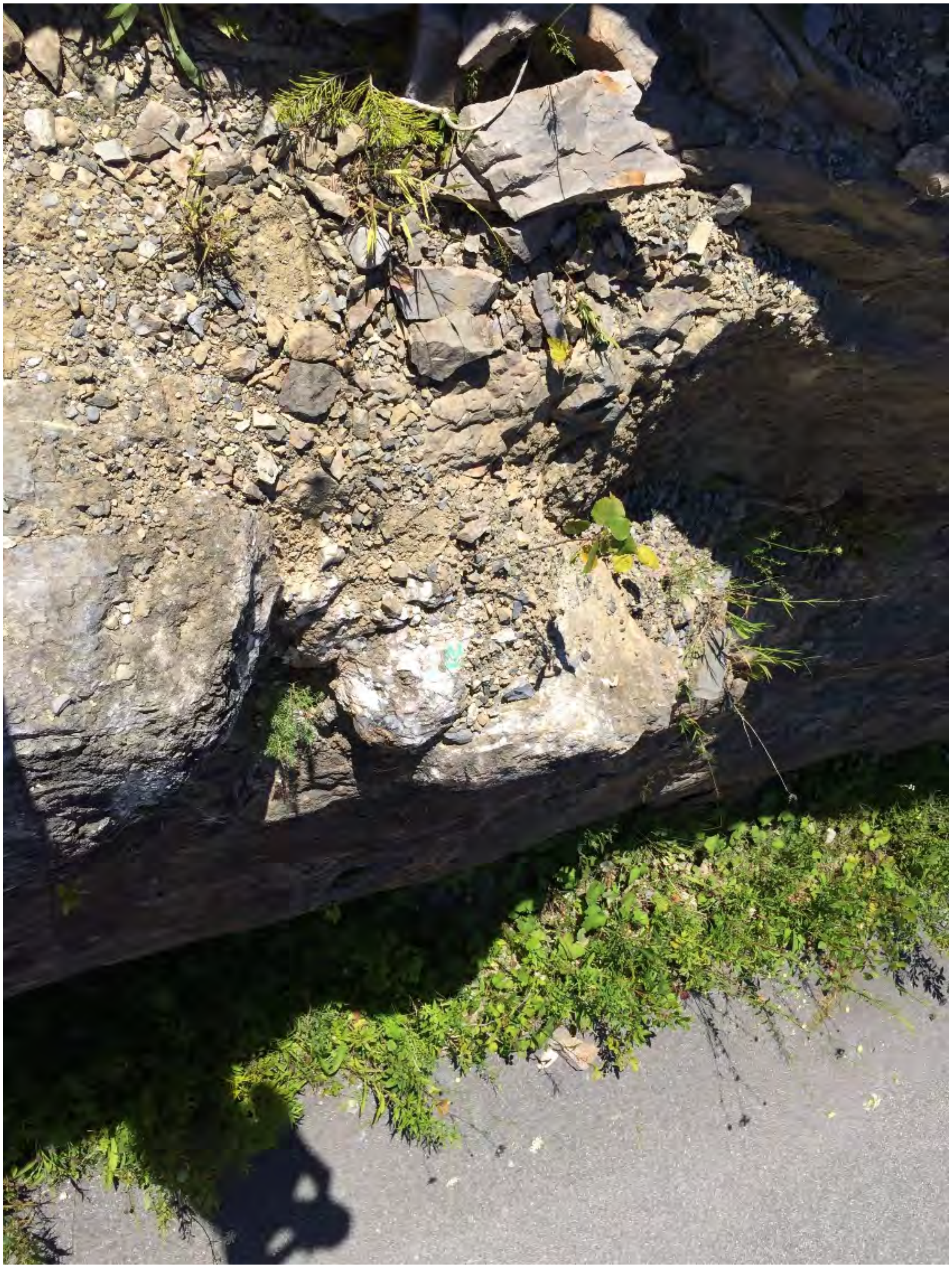

Lab Description:

72018-17 (C302): circles in image denote areas of interest for dating

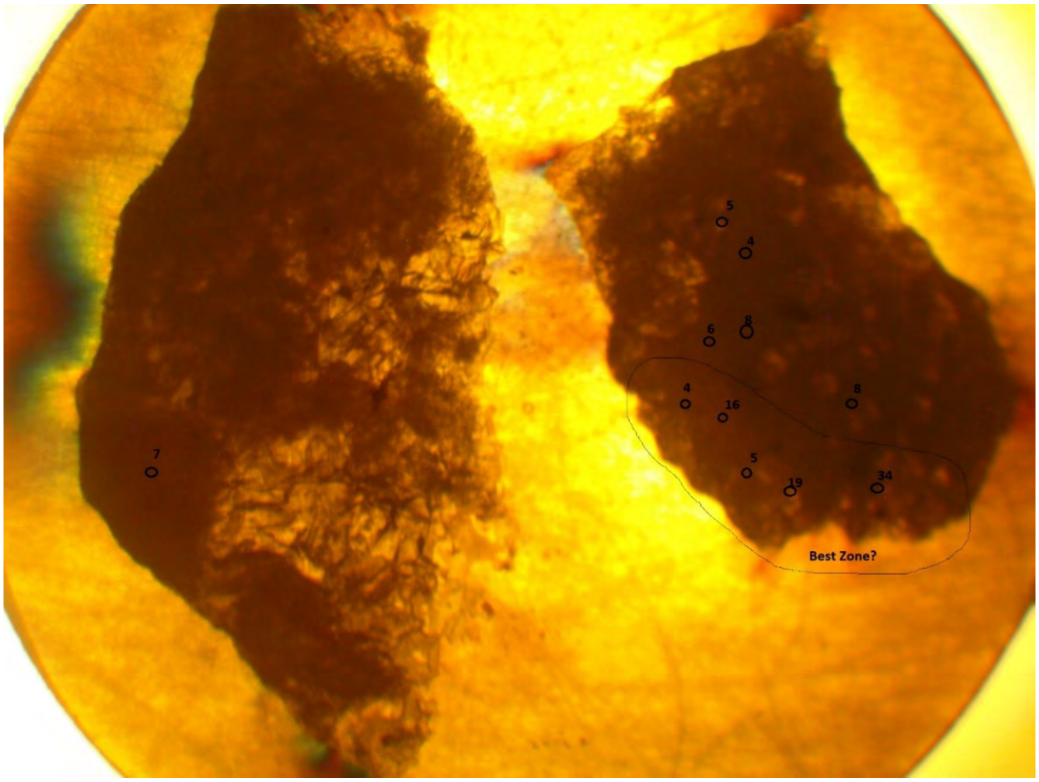

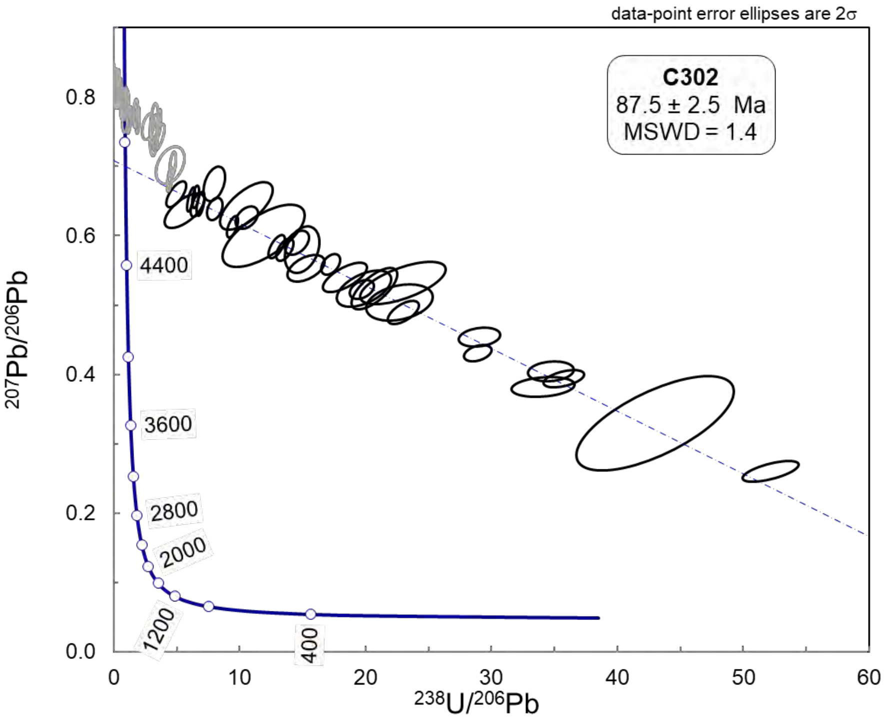

72018-20 (C304A): circles in image denote areas of interest for dating

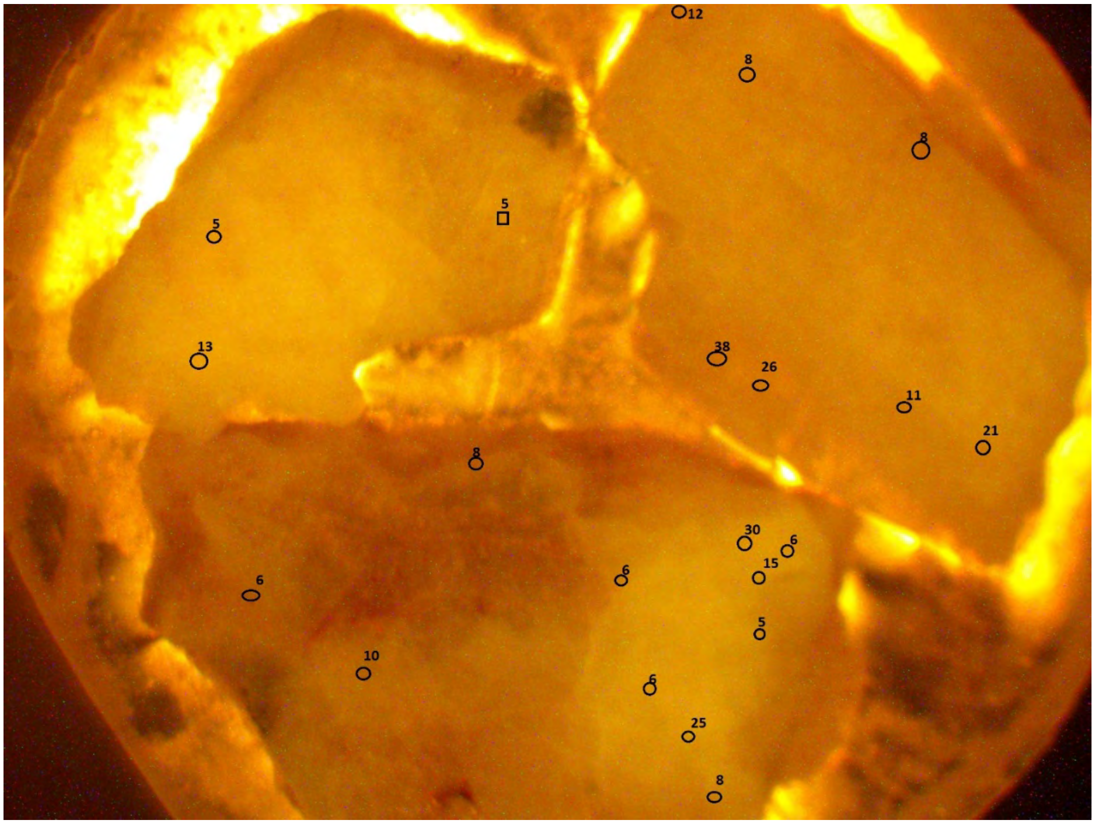

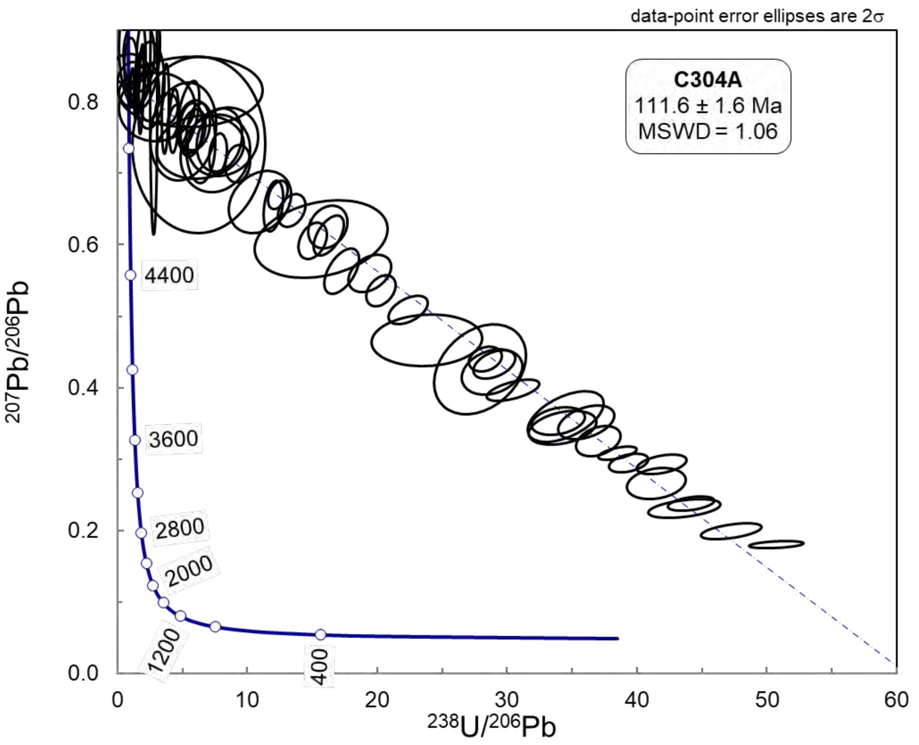

Supplement: Supplementary file 6 — Source Data [file 41467_2022_29680_MOESM6_ESM.zip › 2.zip!/appendix2b_site_summaries_21to50/25_bennington_plastics_final.pdf]
