# Supplementary material for: Pace of passive margin tectonism revealed by U-Pb dating of fracture-filling calcite
Source: Nat Commun. 2022 Apr 12;13:1953. doi: 10.1038/s41467-022-29680-z (PMC9005700; doi:10.1038/s41467-022-29680-z)

# 26: Quaker Smith Pt., Lake Champlain (44.374046, -73.269524)

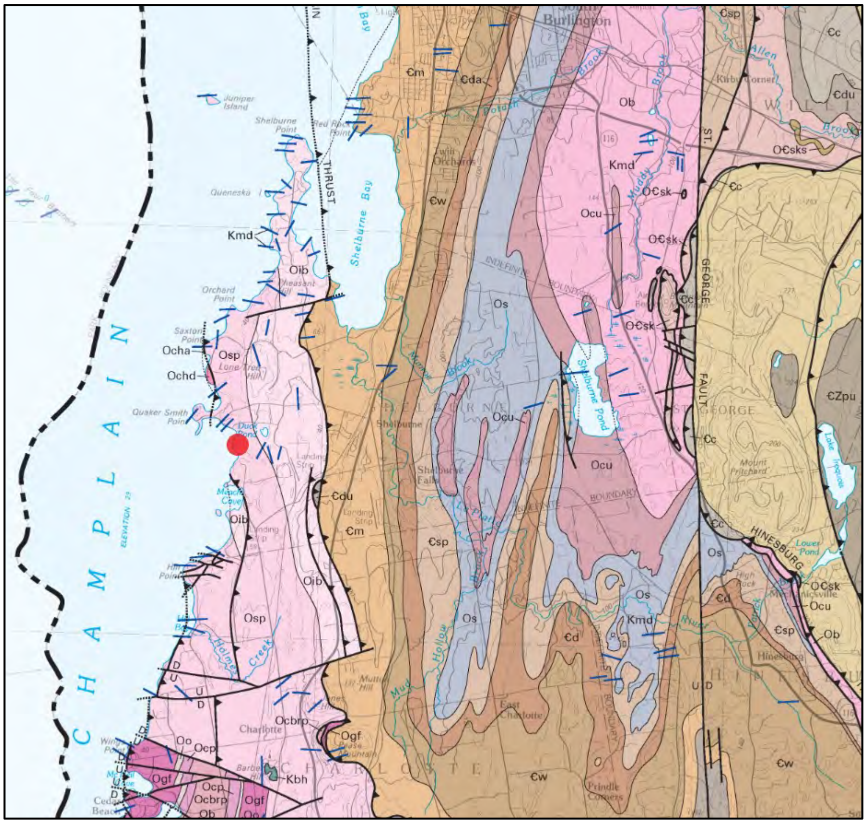


**Figure 1**: Sample location on state geologic map of Ratcliffe at al. (2011).

**<u>Failed samples from same site:</u>** C342A

**<u>Bedrock Unit:</u>** Stony Point Formation (Osp)

**<u>Ages</u>: C342B:** 83.4 ± 1.9 & 52.4 ± 1.6 Ma

**Summary**: This site is located in a cliff-bound cove along the shoreline south of Quaker Smith Point and can only be reached by boat. Shelburne beach is the nearest boat access. A very low angle fault zone wraps around a point, initially parallel to cleavage, but cutting upward across cleavage along strike to the north. The fault zone disappears into a high angle extensional fault oriented E-W and is itself offset by a small accessory fault a few feet away from the high-angle fault. The fault zone contains fairly consistent tabular calcite lenses ranging from 0.5 to 1.5 cm thick. In some places veins appear to be quartz+ calcite. The fault zone strikes ~86° and dips ~10° to the south, with very developed elongate striae trending roughly 316°. Although the sense of slip on the growth striae could not be determined, their trend is extremely consistent suggesting a single episode of slip recorded here (figure 2). We infer the fault to be oblique extensional based on the continuity and thickness of the calcite lenses, which would require open void space to precipitate. The face value interpretation of this site is thus E-W extension at 84 Ma, with a later episode of fluid flow or faulting near 52 Ma. A second episode of crystallization is clear from the U-Pb isochron plot and the drastically different textures (clear vs. translucent) visible in the sample itself.

## Field Observations:

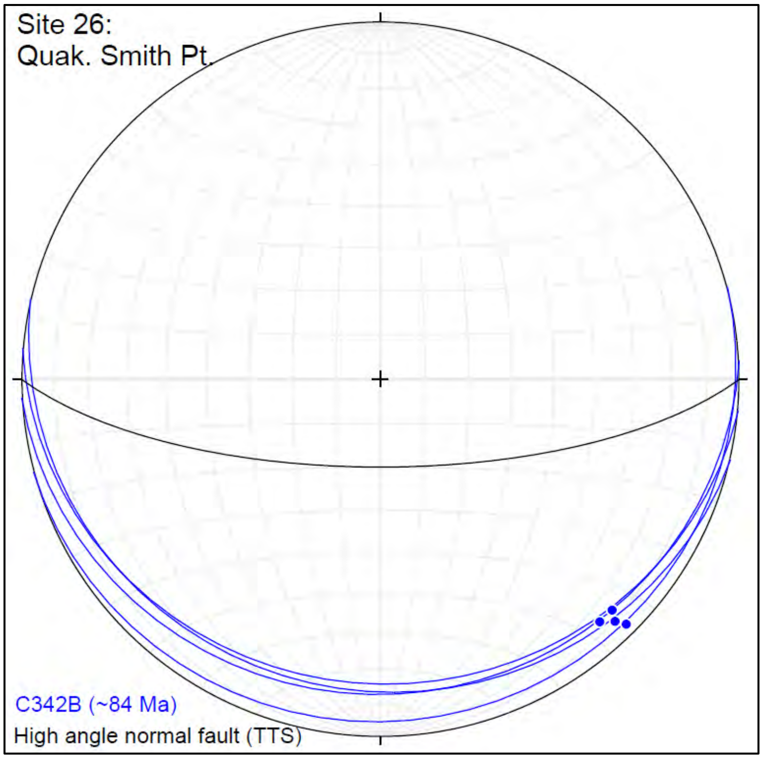


**Figure 2**: Stereoplot showing orientation of fault and striae ate site 26.

**C342B:** red circle shows sample location, high angle fault at left in 2nd photo

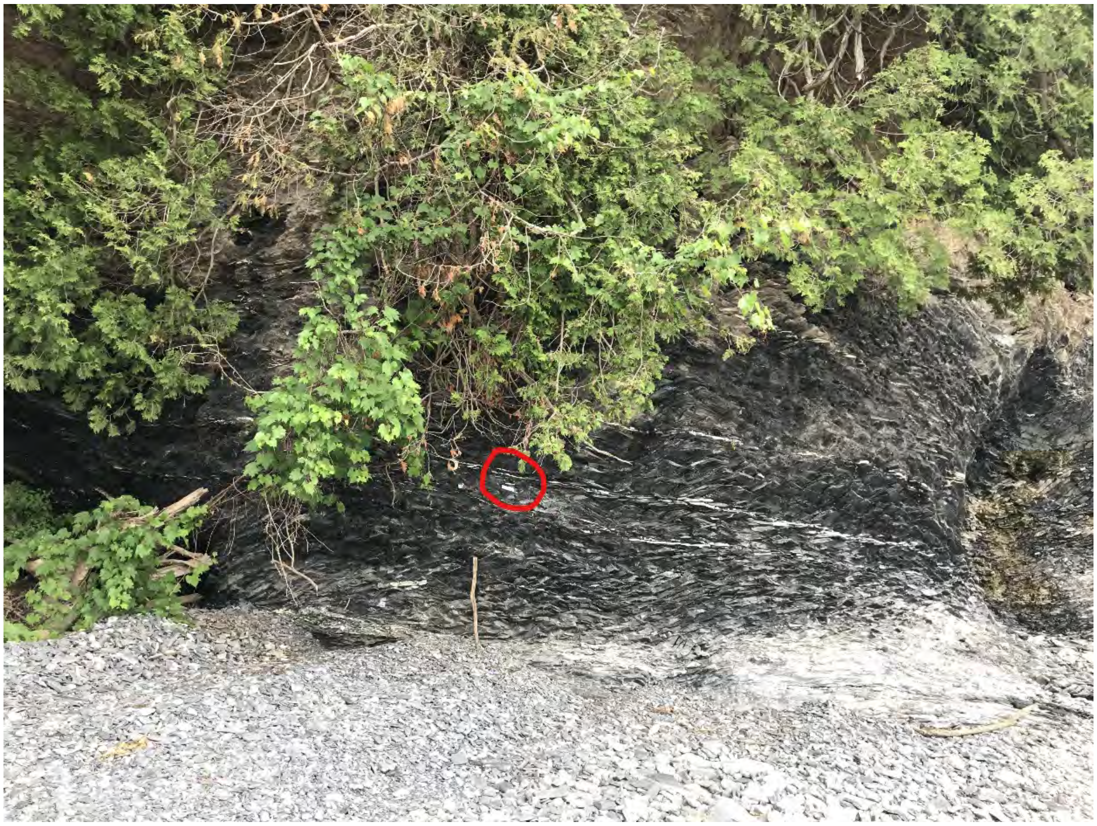

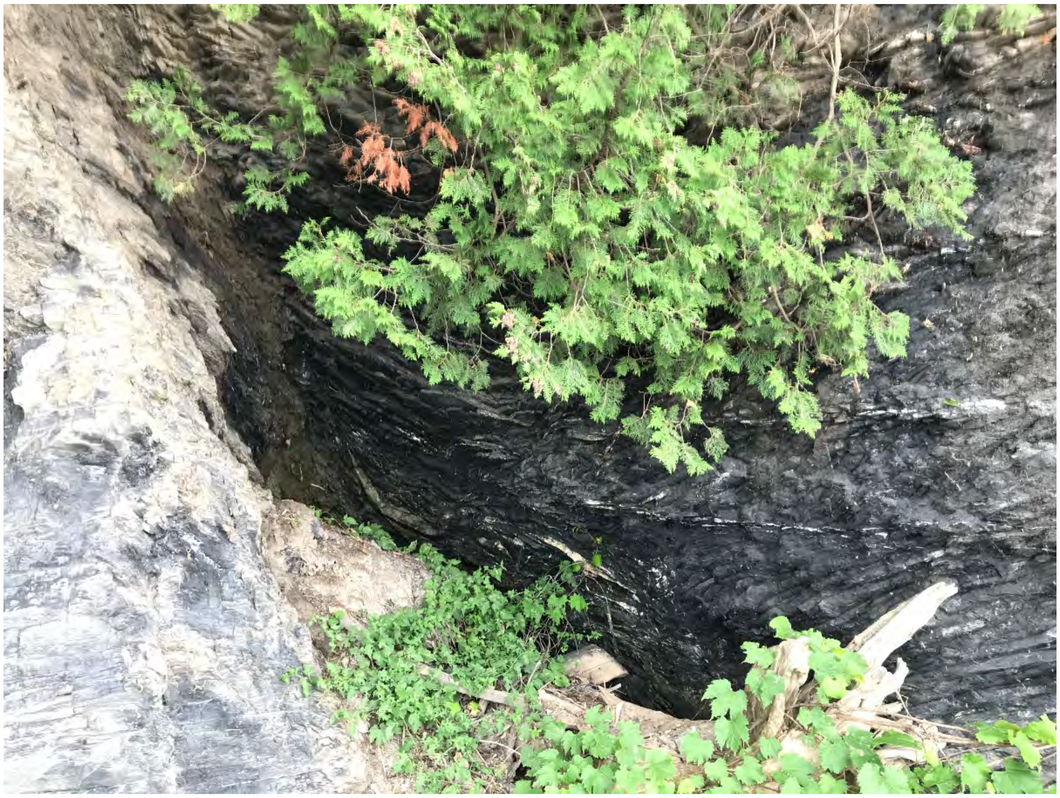

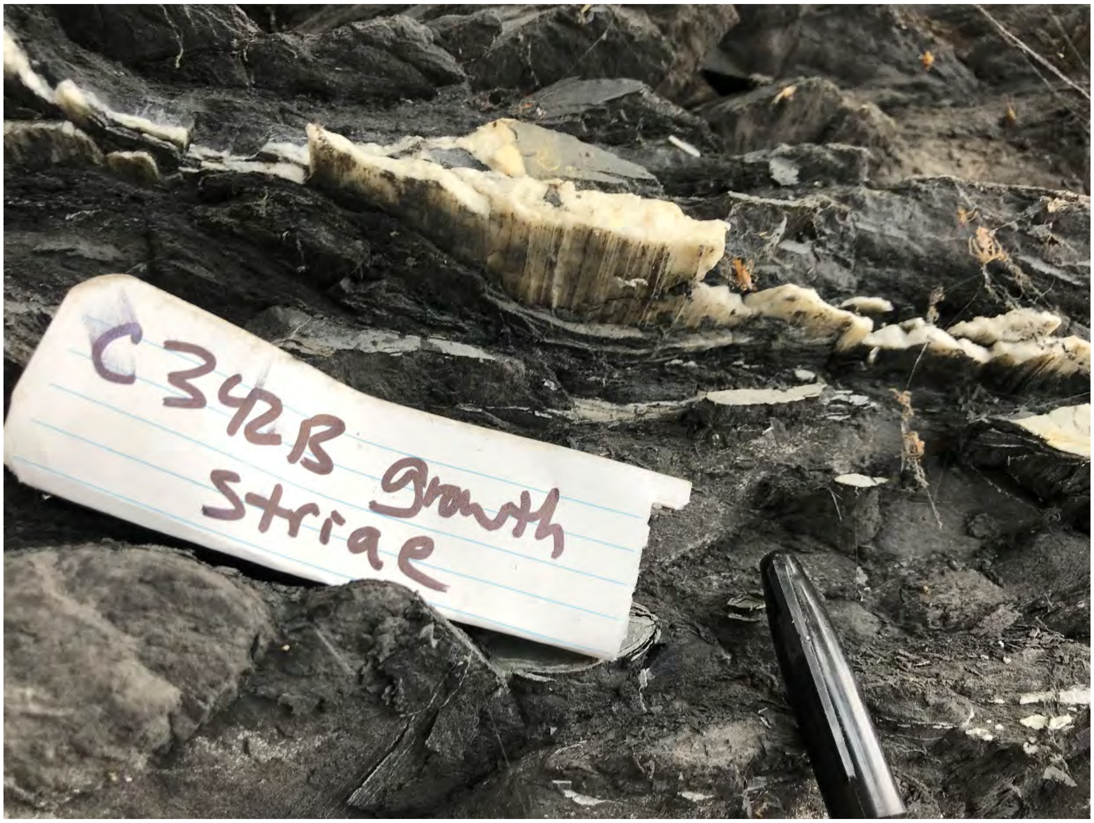
C342B growth
striae

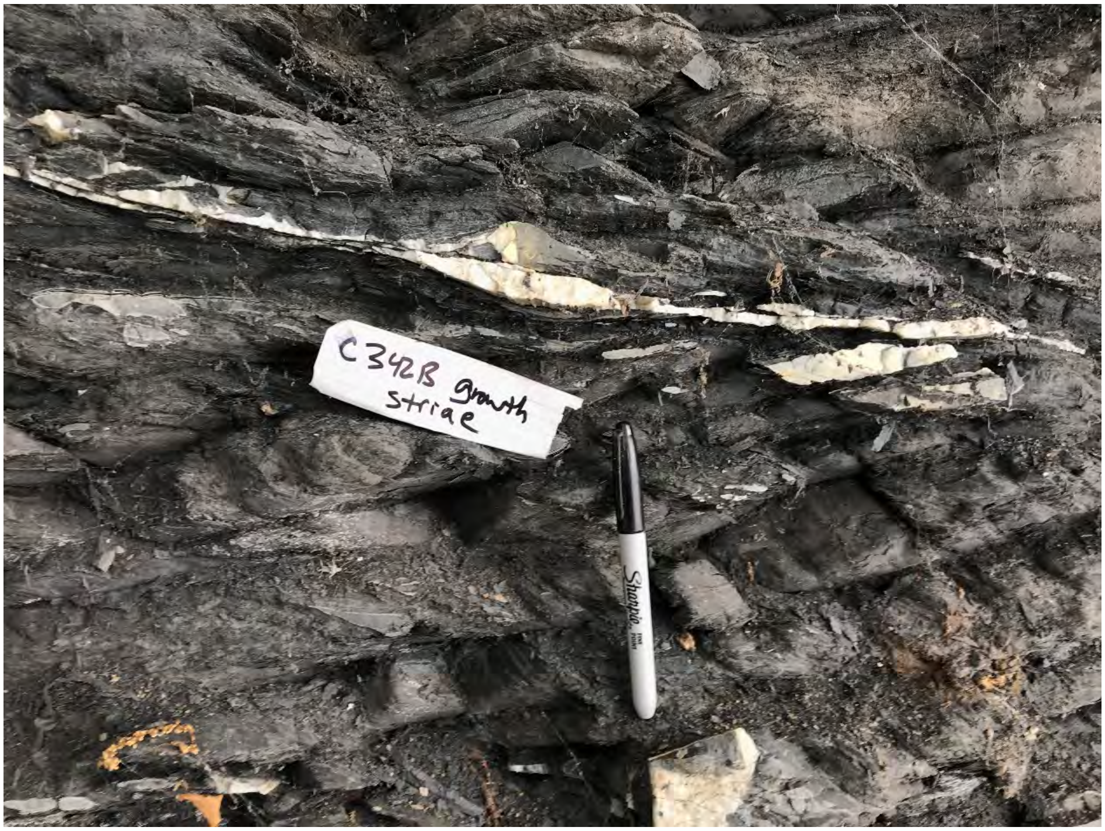
C342B growth
striae
Sharpie

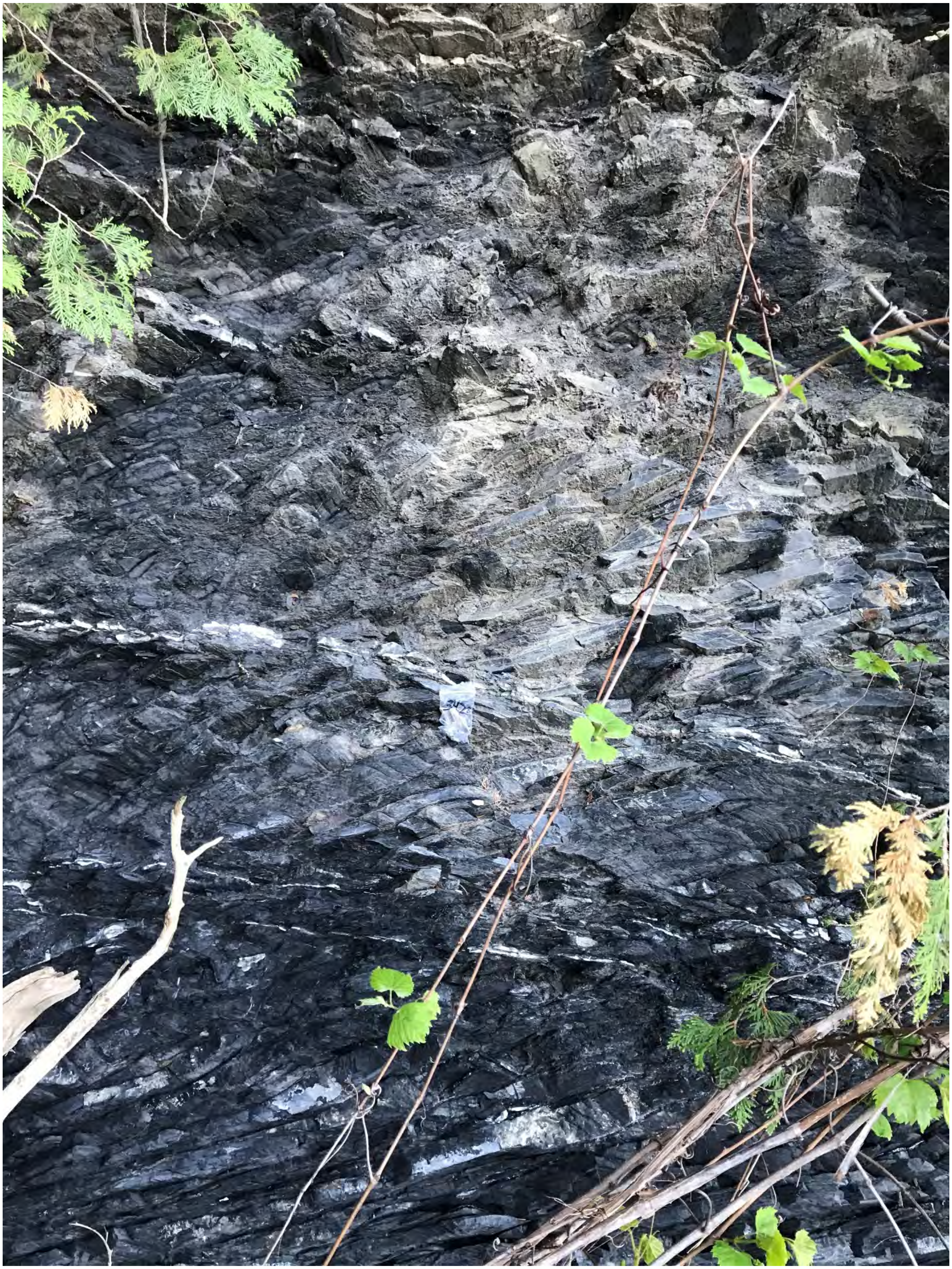

**Lab Description**:

C342B:

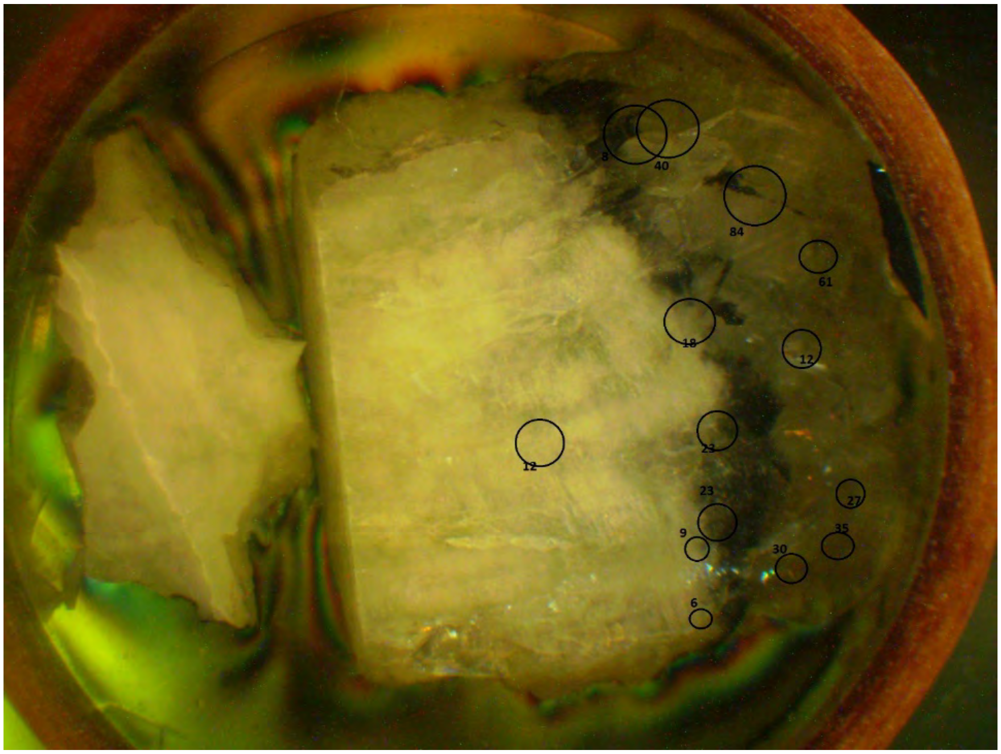

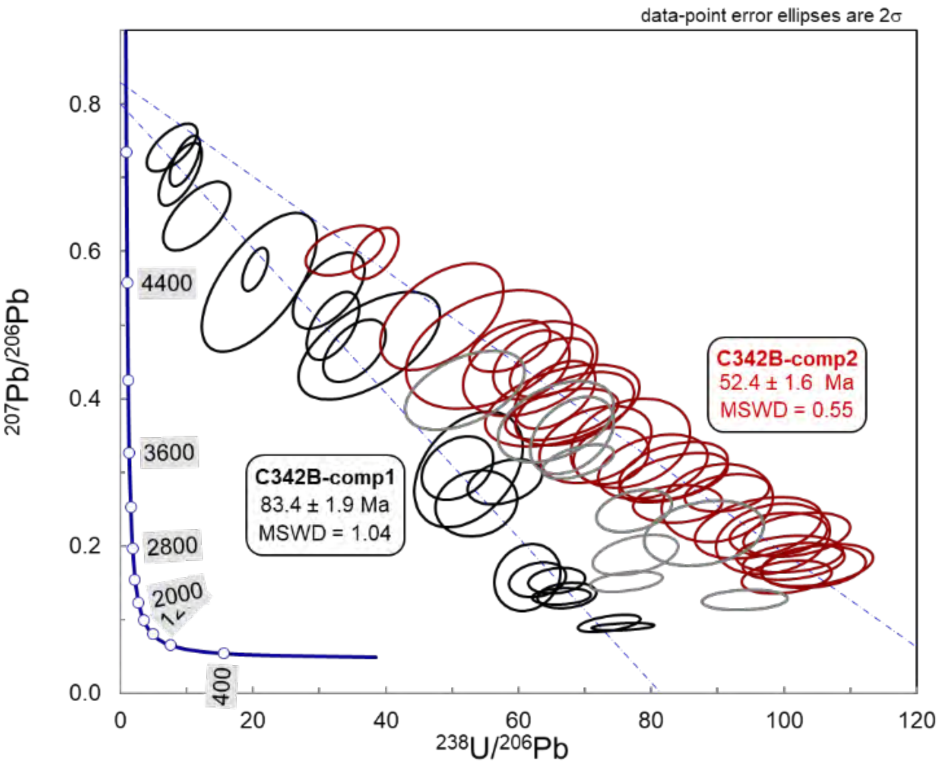

Supplement: Supplementary file 6 — Source Data [file 41467_2022_29680_MOESM6_ESM.zip › 2.zip!/appendix2b_site_summaries_21to50/26_quaker_smith_pt_final.pdf]
