# Supplementary material for: Pace of passive margin tectonism revealed by U-Pb dating of fracture-filling calcite
Source: Nat Commun. 2022 Apr 12;13:1953. doi: 10.1038/s41467-022-29680-z (PMC9005700; doi:10.1038/s41467-022-29680-z)

27: Shelburne Farms Inn (44.395035, -73.279024)

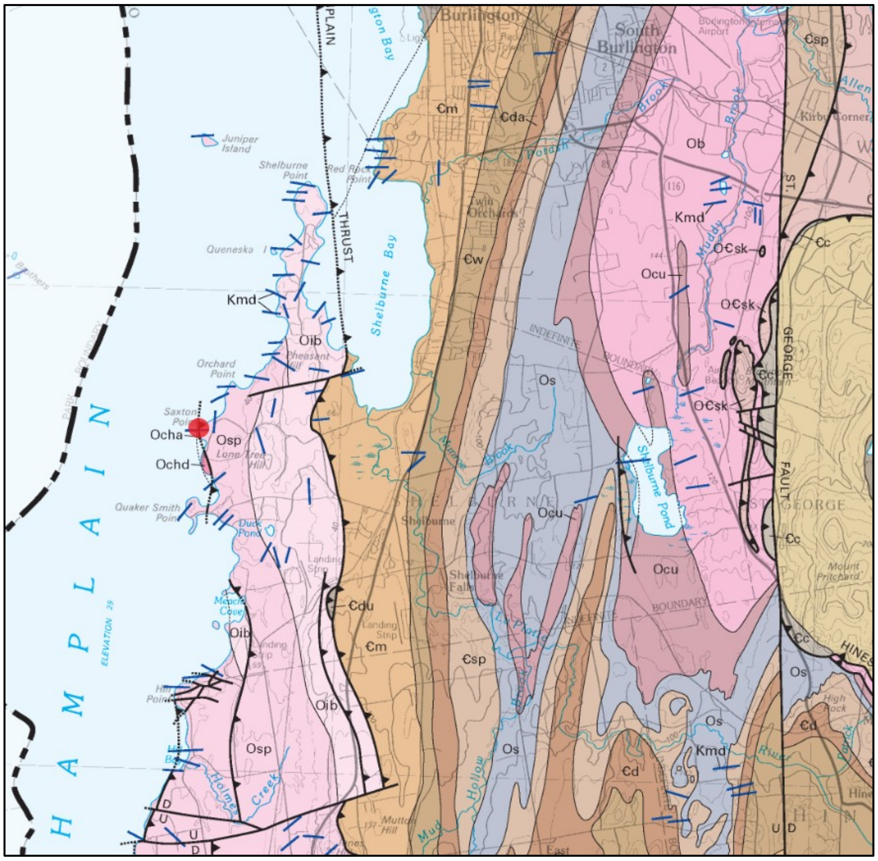

Figure 1: Geologic map of the sample site (Ratcliffe et al., 2011).

<u>Failed samples from same site:</u> C353B

<u>Bedrock Unit:</u> Stony Point Formation (Osp) or Cumberland Head Formation (Ochd) – Contact?

<u>Ages</u>: C353A: 83.8 ± 2.7 & 65.5 ± 2.8 Ma;

<u>Summary</u>: This site is located at the base of a cliff roughly ½ mile SW of the Shelburne Farms Inn. At low water the site can be reached by walking east along the north shore of the cove and around the point to the north. The date comes from a ~ 1 cm thick calcite tab within a fault zone striking roughly 328° and dipping roughly 28° to the NE. The age is bimodal, with laser shots forming two distinct isochron lines. . The dated calcite tab has well developed striae oriented in a nearly down-dip direction, suggesting extensional slip in a roughly E-W direction (see figure 2 below). Well developed striae on undated calcite in the lower part of the fault zone show slip in a more NE-SW sense, perhaps during a separate episode of faulting. Taken at face value we interpret this site to record ENE-WSW extension at roughly 84 Ma, in agreement with many other sites in the region. Potential for further study at this site is high if it can be linked to the work Jon Kim, Keith Klepeis and others have done just around the corner to the south along the north shore of the cove (e.g. Kim et al., 2019).

Field Descriptions:

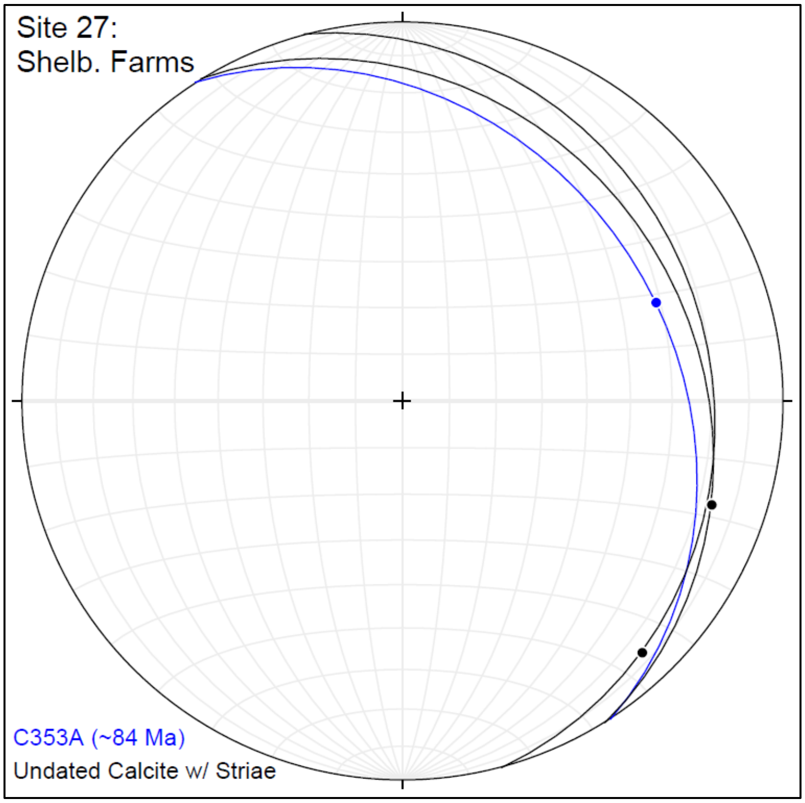

Figure 2: Stereonet showing structural measurements from the fault zone at site 27.

C353A:

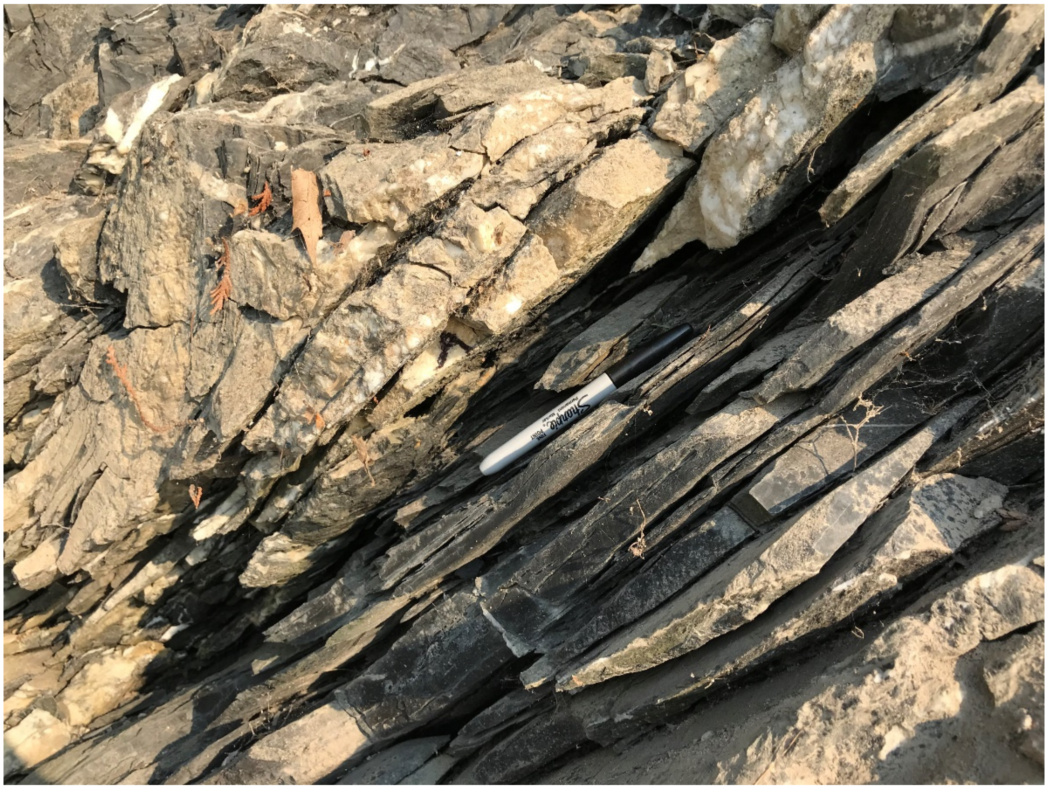

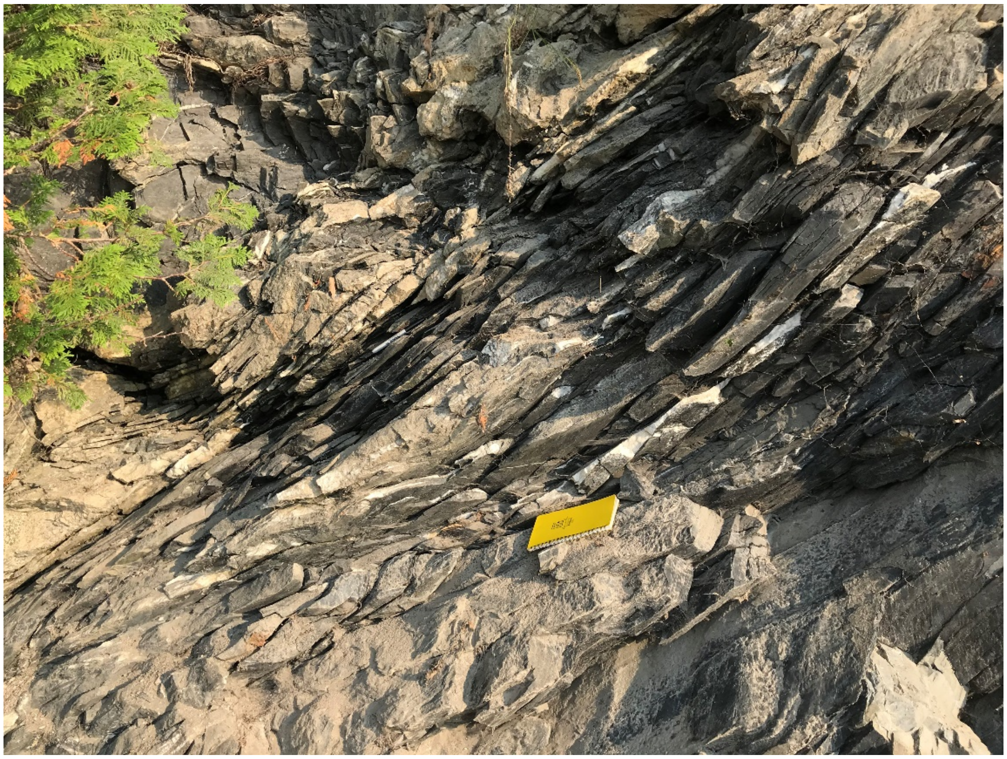

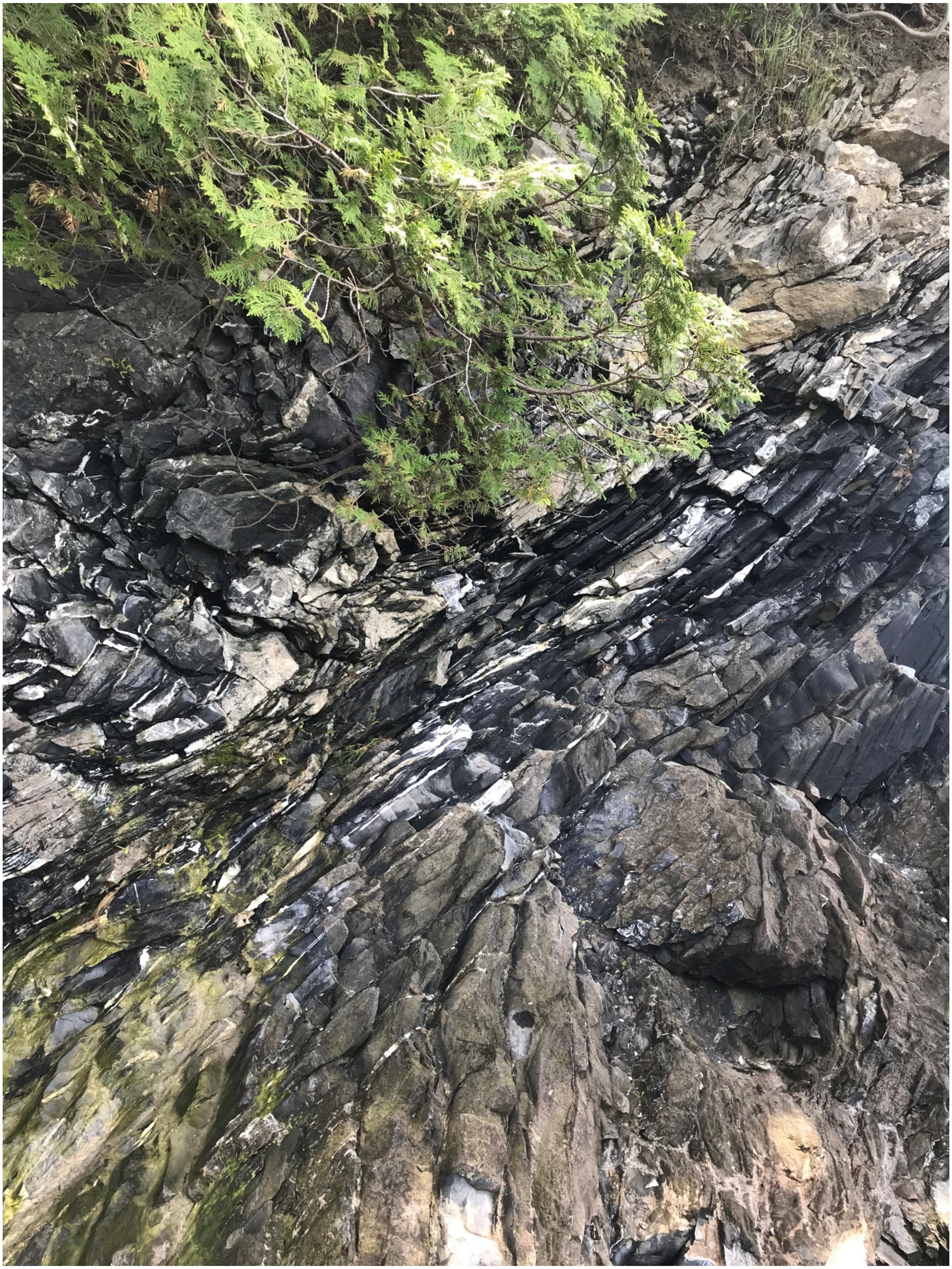

Lab Description:

C353A: circles represent areas of interest, not laser shots

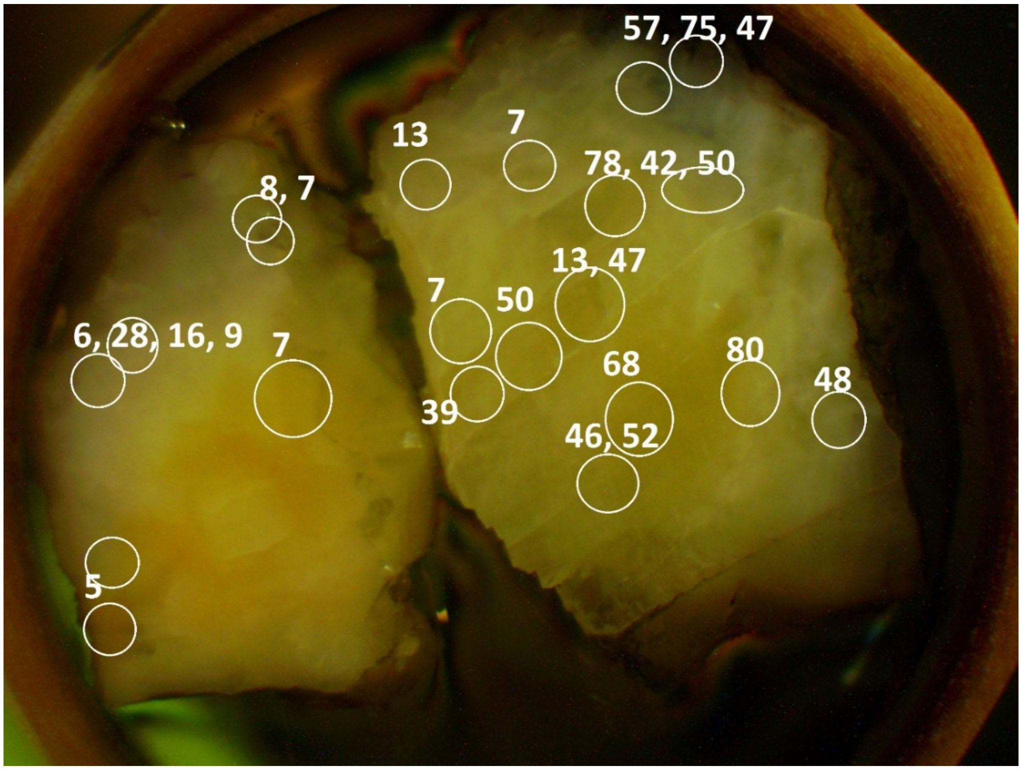

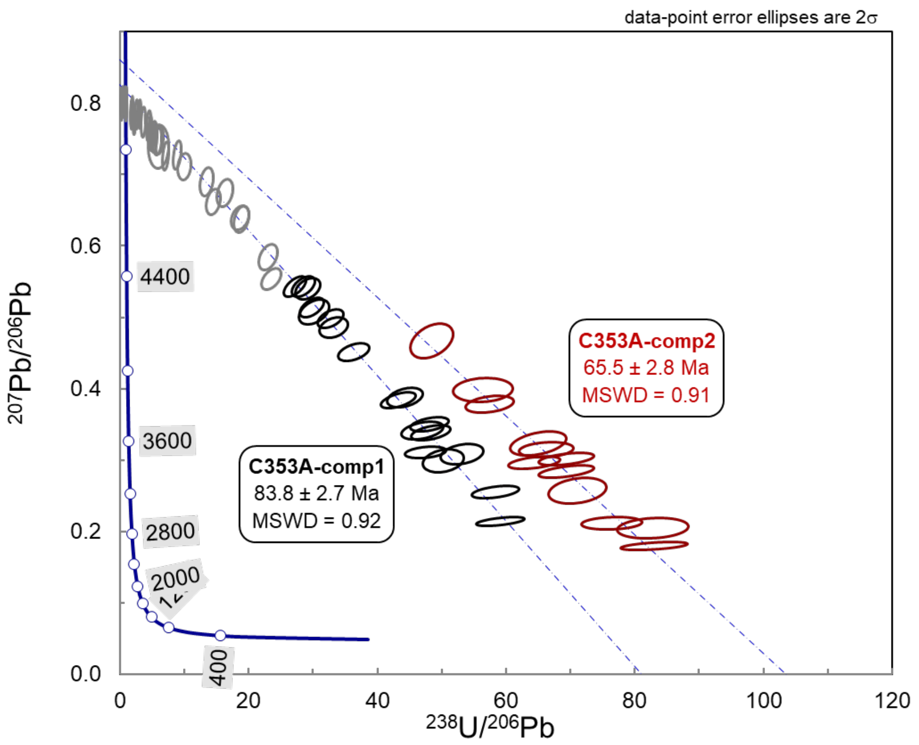

Supplement: Supplementary file 6 — Source Data [file 41467_2022_29680_MOESM6_ESM.zip › 2.zip!/appendix2b_site_summaries_21to50/27_shelburne_farms_final.pdf]
