# Supplementary material for: Pace of passive margin tectonism revealed by U-Pb dating of fracture-filling calcite
Source: Nat Commun. 2022 Apr 12;13:1953. doi: 10.1038/s41467-022-29680-z (PMC9005700; doi:10.1038/s41467-022-29680-z)

28: Hartford: Rte. 4 + I84 junction (41.723913, -72.811007)

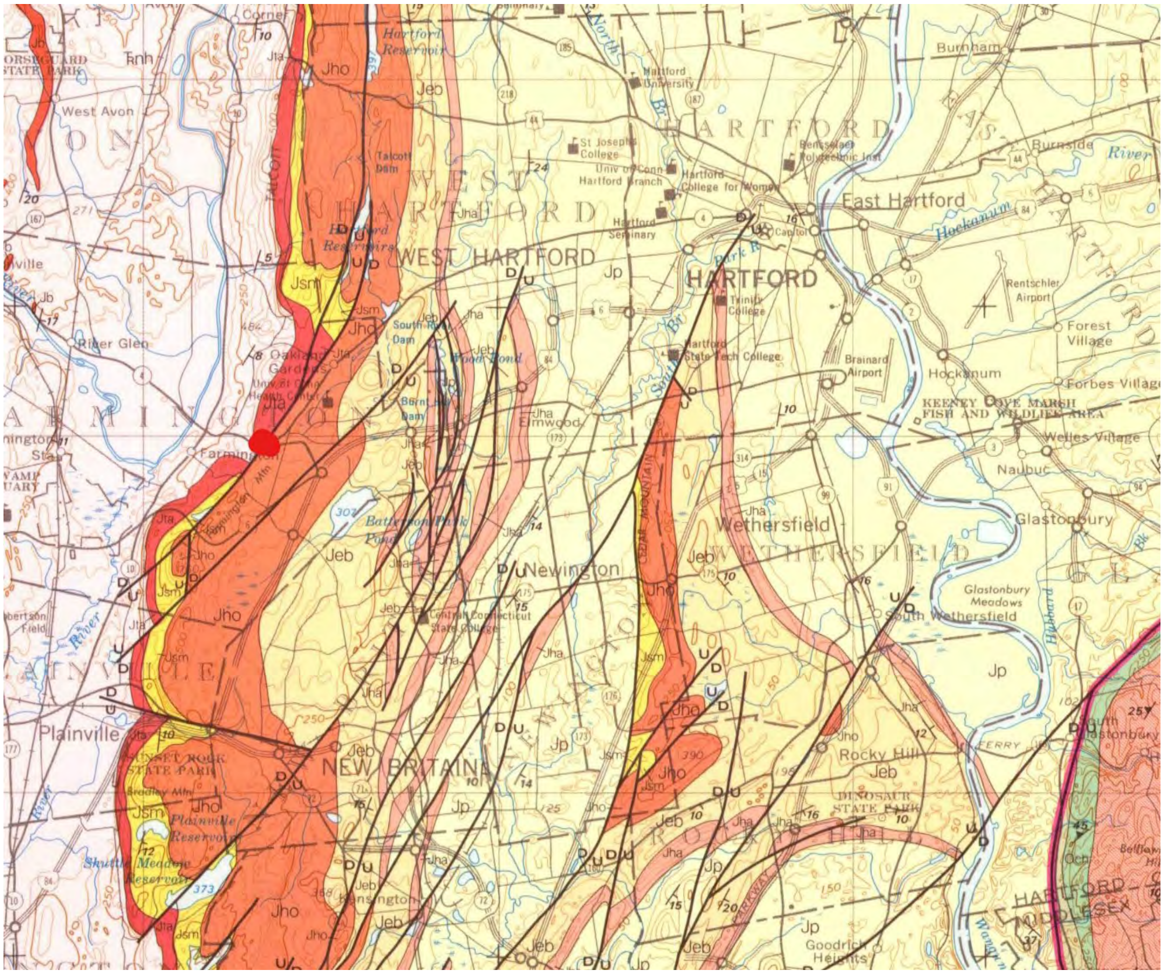

Figure 1: Sample location (red dot) on Connecticut state geologic map of Rodgers, 1985.

Failed samples from same site: C356A + C356C

Bedrock Unit: Talcott Formation (Jta)

Ages: C356B: 5.3 ± 0.6 Ma

Summary: This sample is collected from a sub-vertical strike-slip fault cutting through the Talcott Basalt in Farmington, CT. The fault strikes 27° and dips 80°E but no slickensides are observed on this surface. Slickensides observed on a parallel fault surface show a right-lateral sense of slip. The U-Pb isochron suggests only one age component and yields a fairly convincing late Miocene age. However, the image of the polished sample shows multiple textures, which could reflect multiple tectonic events, quartz + calcite, or weathering. Further investigation is required to determine if this age represents slip on the fault or a secondary crystallization event.

Field Descriptions:

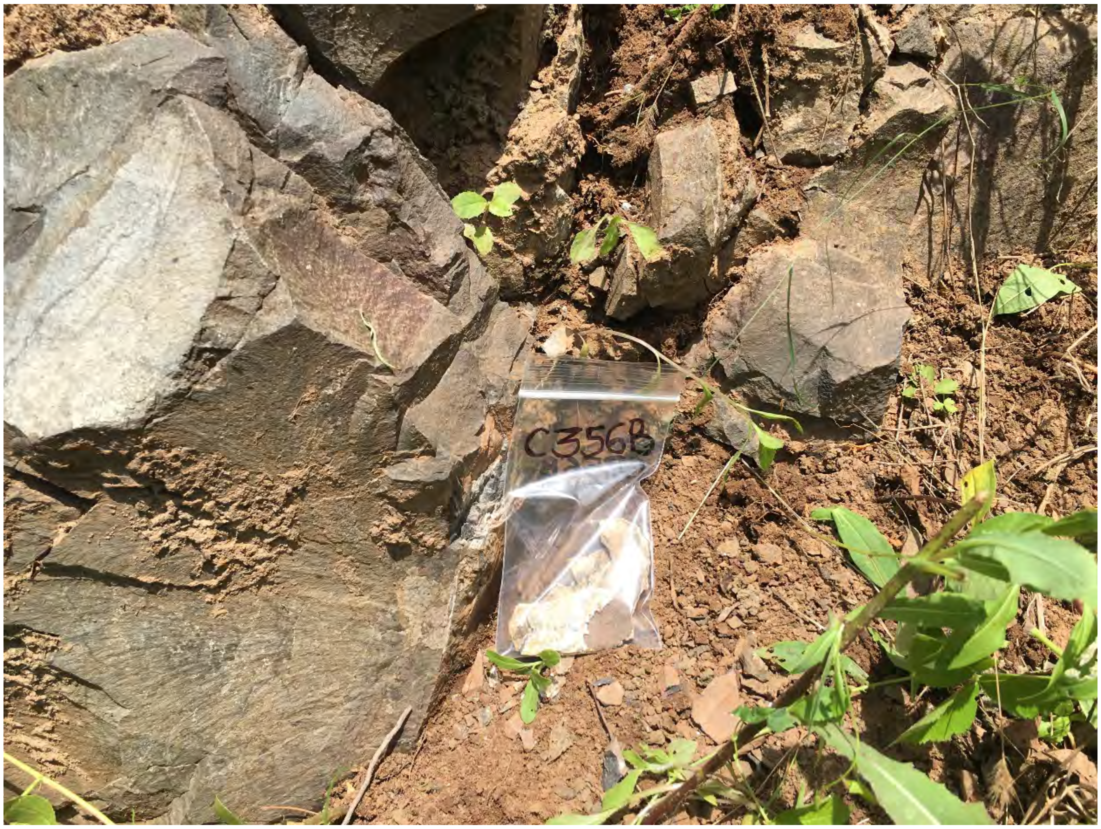

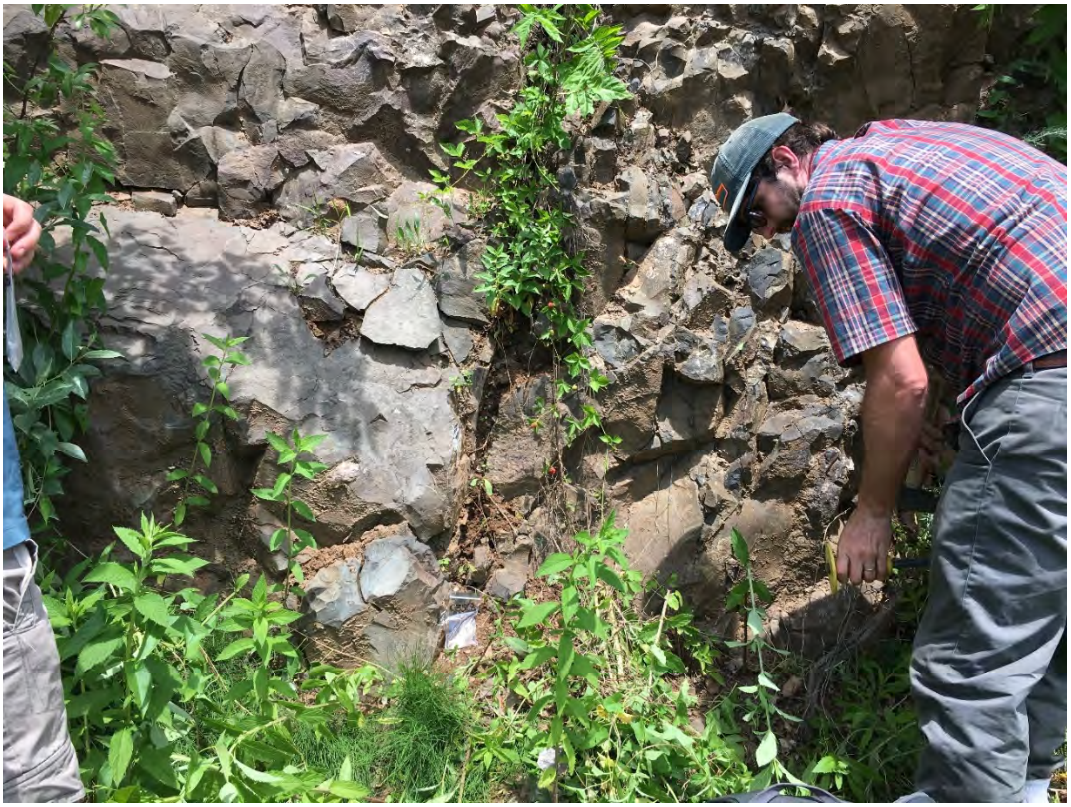

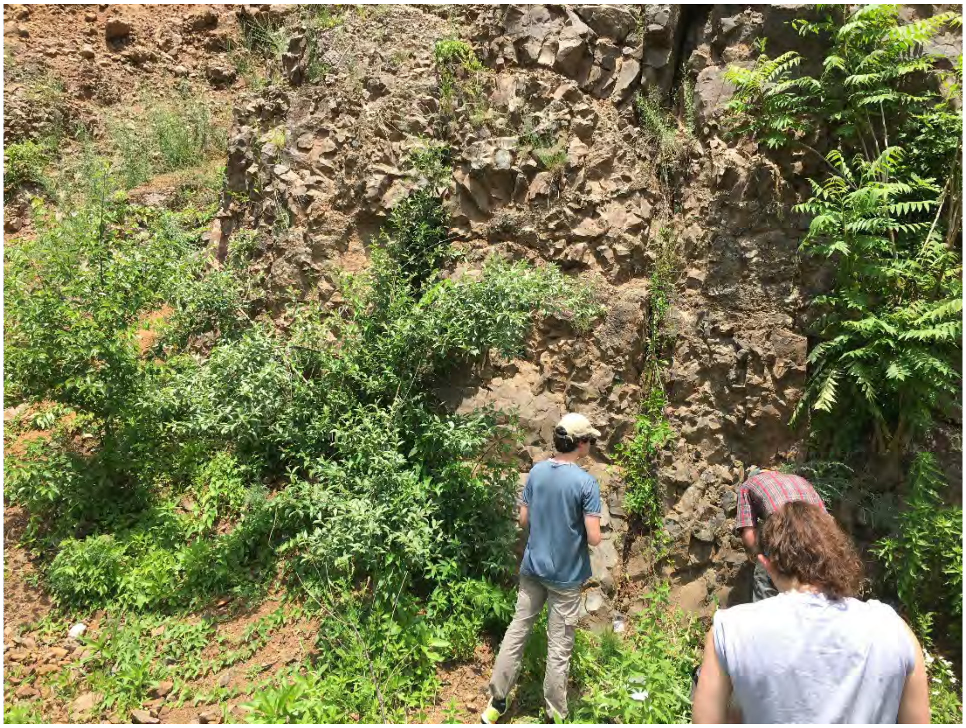

Lab Description: Circles I image are areas of interest for dating

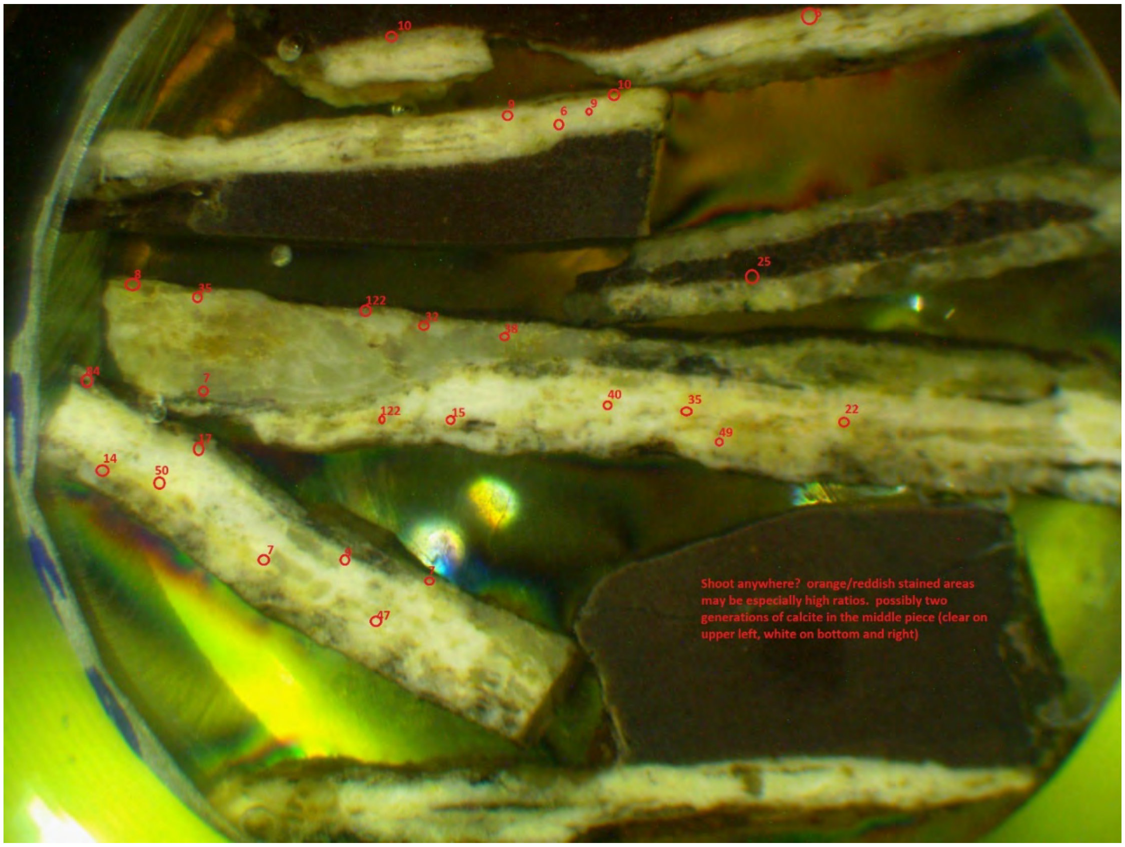

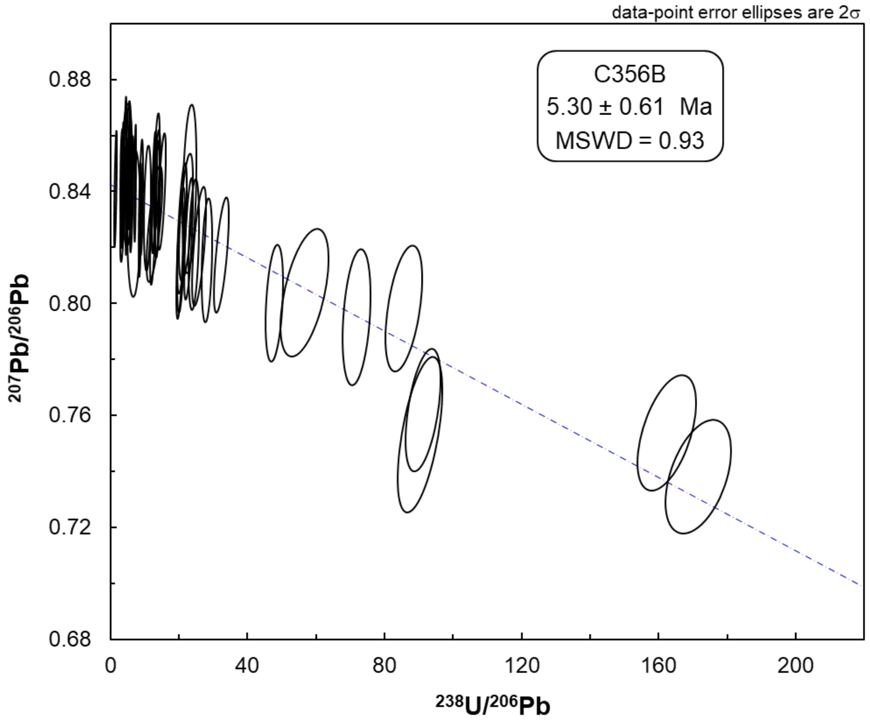

Supplement: Supplementary file 6 — Source Data [file 41467_2022_29680_MOESM6_ESM.zip › 2.zip!/appendix2b_site_summaries_21to50/28_Hartford_rte4_I84_final.pdf]
