# Supplementary material for: Pace of passive margin tectonism revealed by U-Pb dating of fracture-filling calcite
Source: Nat Commun. 2022 Apr 12;13:1953. doi: 10.1038/s41467-022-29680-z (PMC9005700; doi:10.1038/s41467-022-29680-z)
Supplement: Supplementary file 6 — Source Data [file 41467_2022_29680_MOESM6_ESM.zip › 2.zip!/appendix2b_site_summaries_21to50/29_plainville_cityoil_final.pdf]

29: Plainville, CT: Rte. 372, behind City Oil (41.672942, -72.835382)

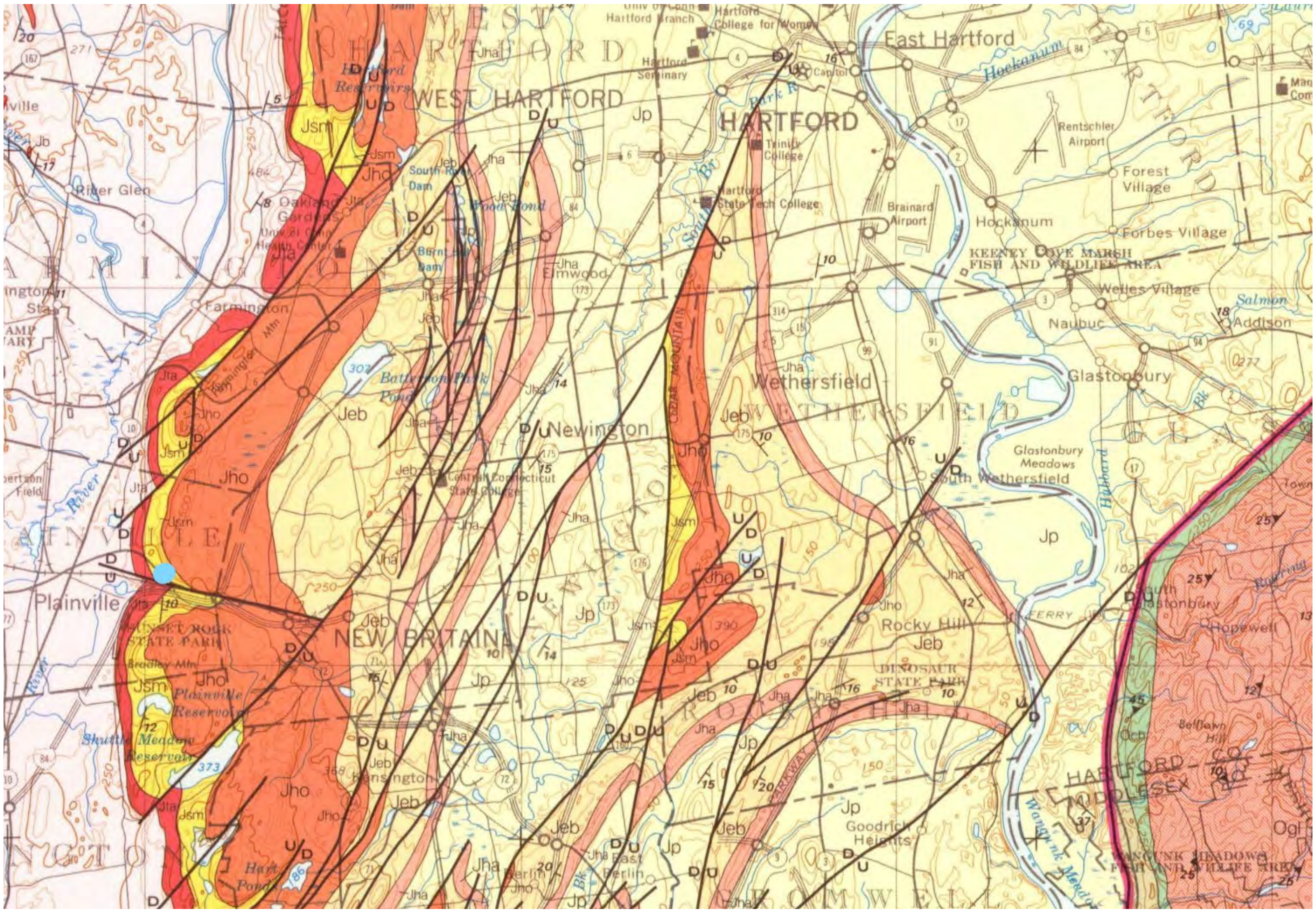

Figure 1: Sample location (blue dot) on the CT state geologic map of Rodgers, 1995.

Failed samples from same site: none

Bedrock Unit: Shuttle Meadow Formation (Jsm)

Ages: **C362A**: 4.85 ± 0.51 Ma **C362B**: 6.03 ± 0.27 Ma

Tectonic context: This site is located in a large outcrop directly behind the parking lot of City Oil in Plainville, CT. The Shuttle Meadow formation is a relatively weak bedded sandstone with 1-3 mm thick veins running horizontally and vertically through the outcrop. Veins lack slip indicators and are likely dilational veins formed under fluid pressure, although further study is needed. Sample C362B is collected from the horizontal fracture and dates to ca. 6.0 Ma, whereas C362B is from the vertical fracture and dates to ca. 4.9 Ma. These relative ages are confirmed by cross-cutting relationships showing the horizontal veins are truncated by the apparently younger vertical veins in a few places. Textural analysis and U-Pb isochrons for both samples suggest a single crystallization event. The primacy of the ages I also supported by similar ages between the two veins. However, the veins do contain some complex textures that warrant further exploration before concluding we have dated the actual formation age of the veins.

Field Descriptions:

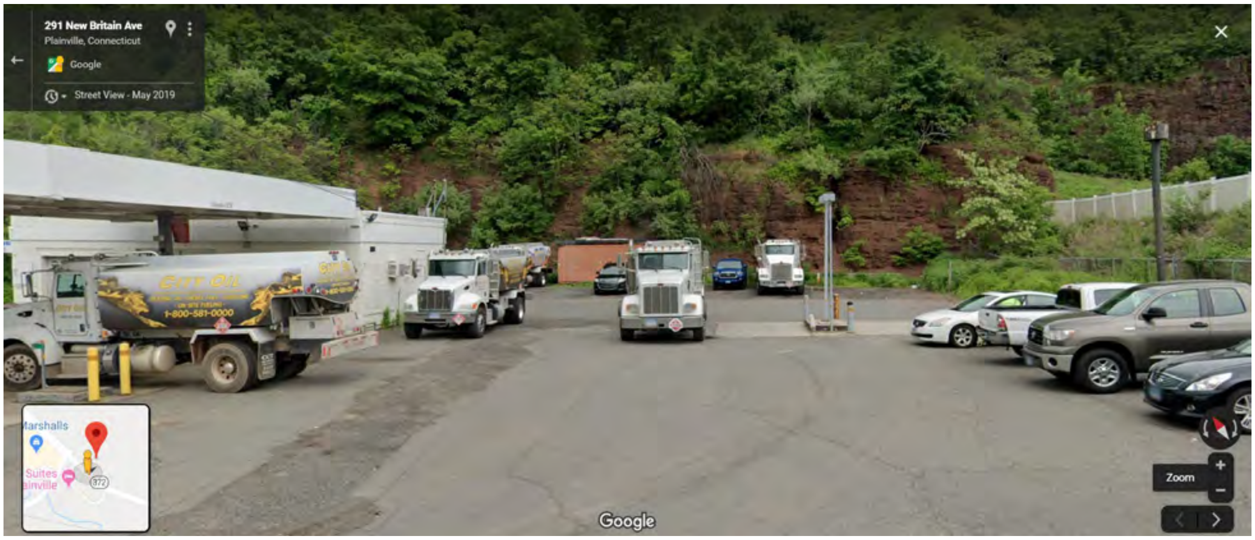

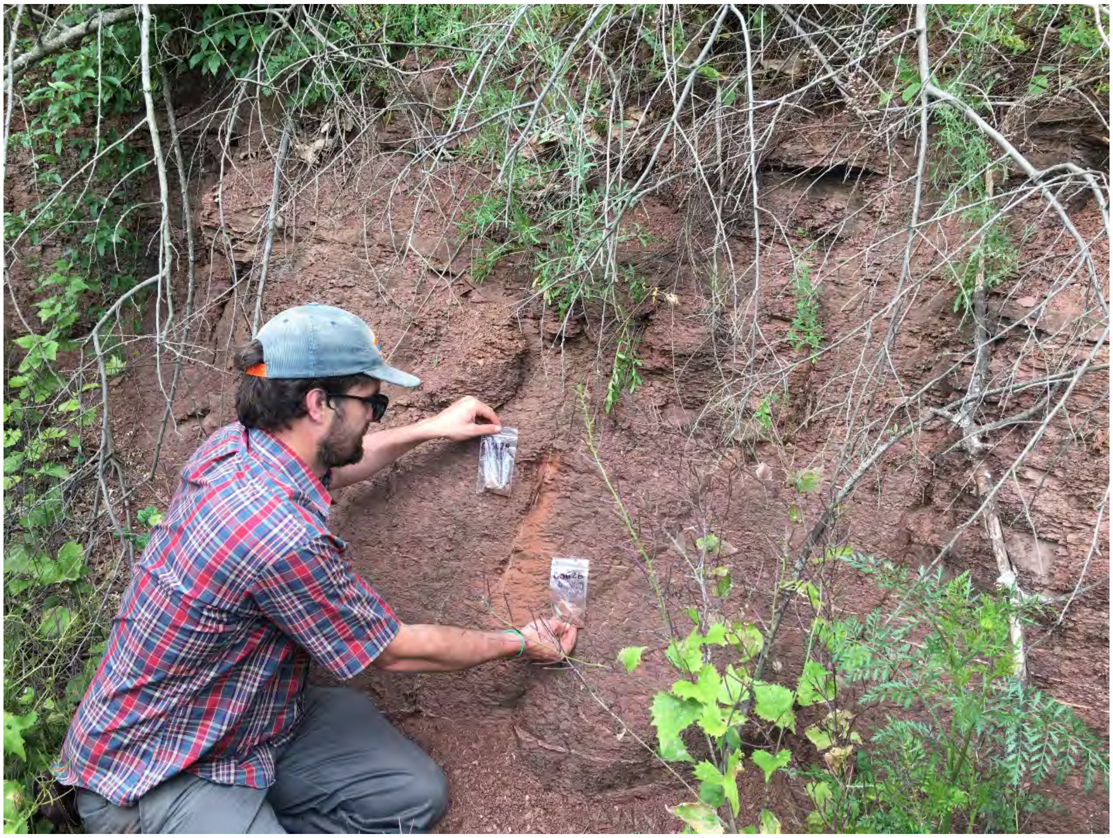

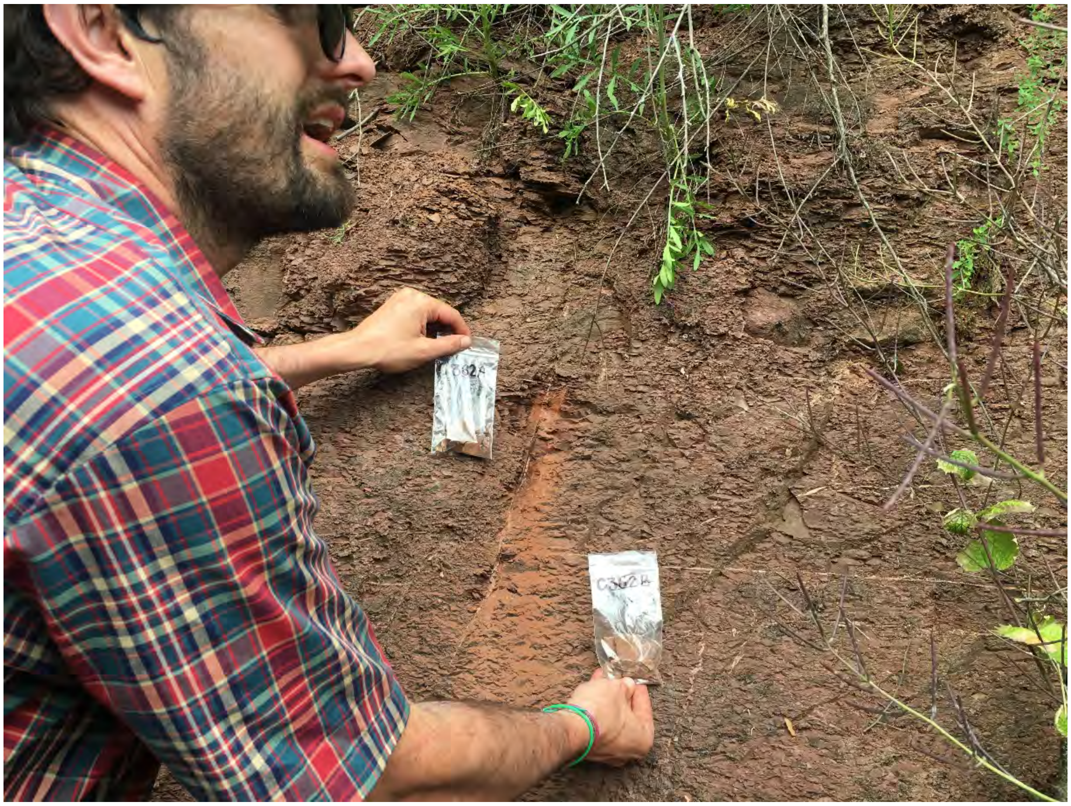

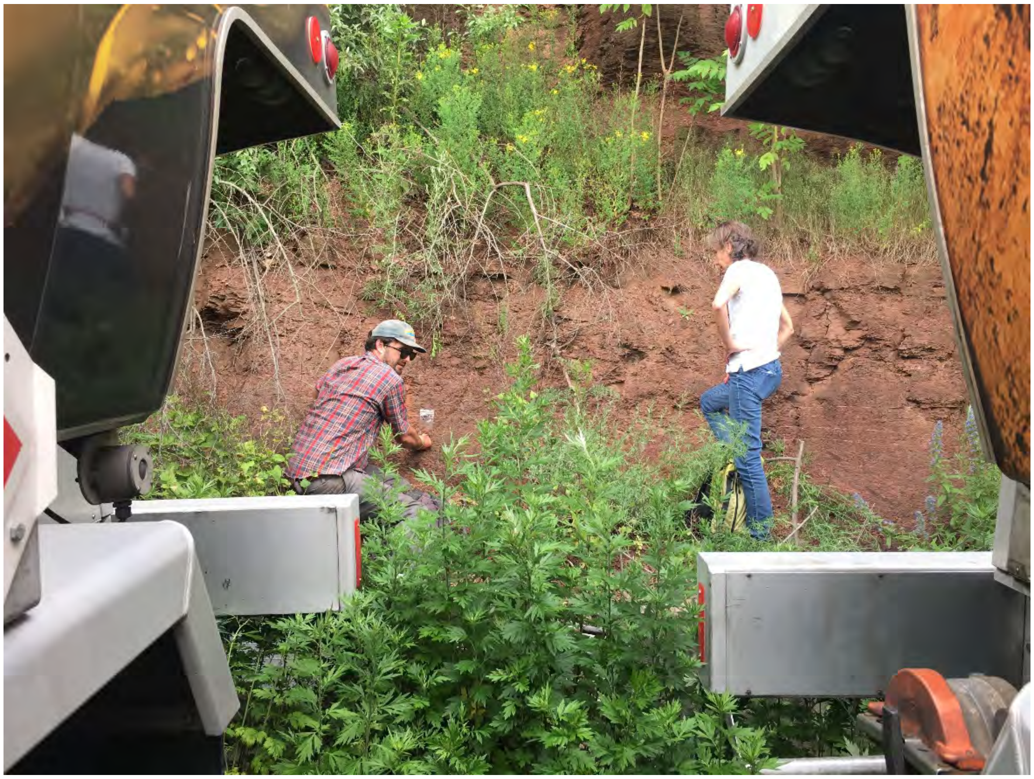

Lab Description: 362A:

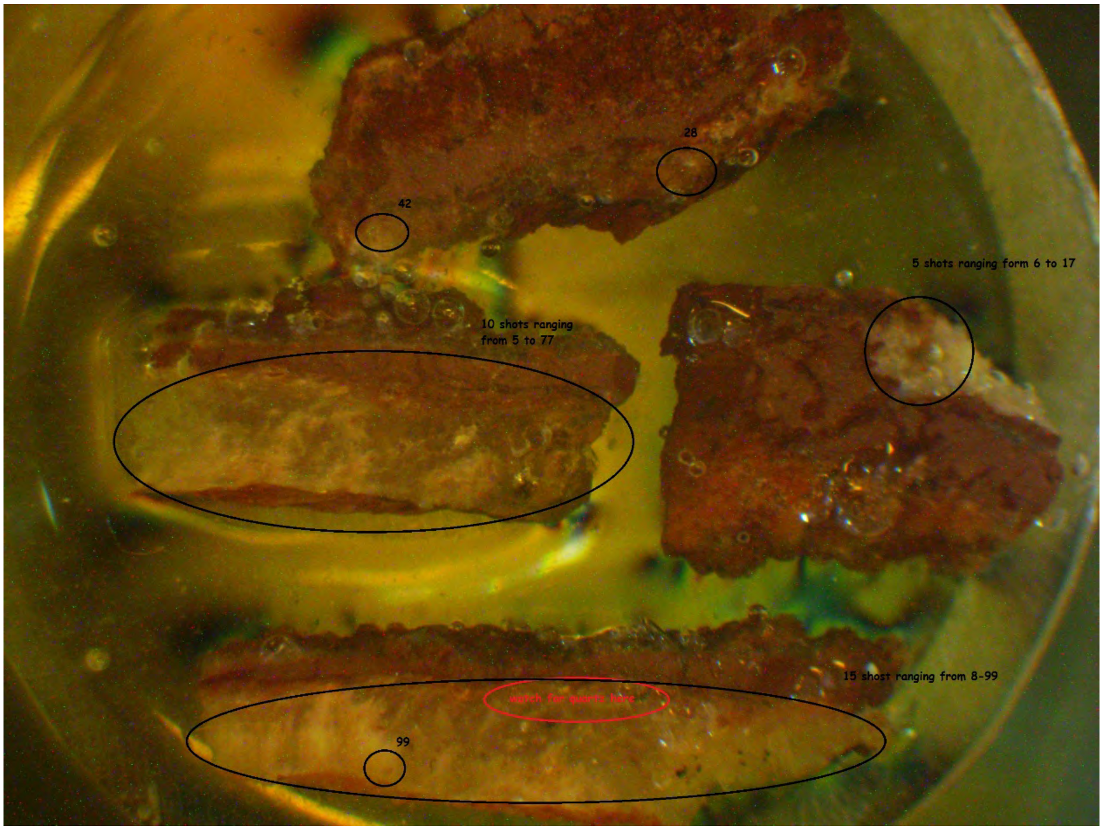

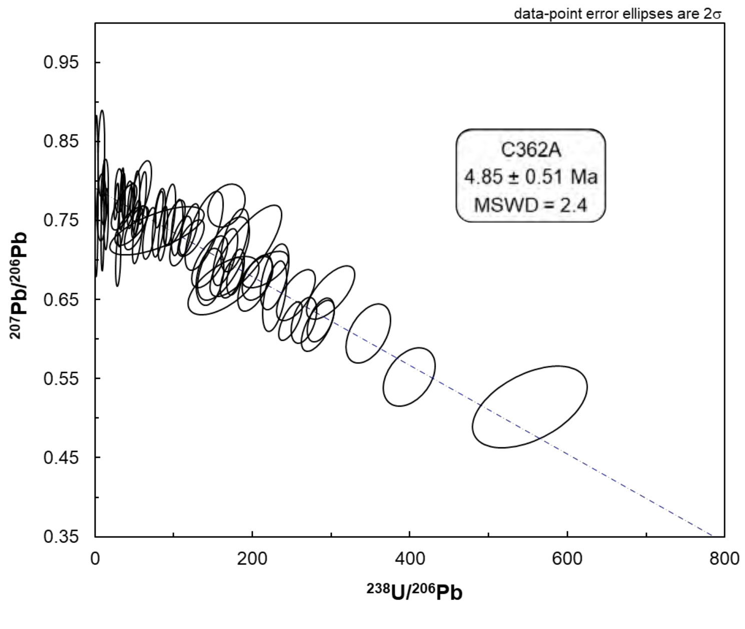

362B:

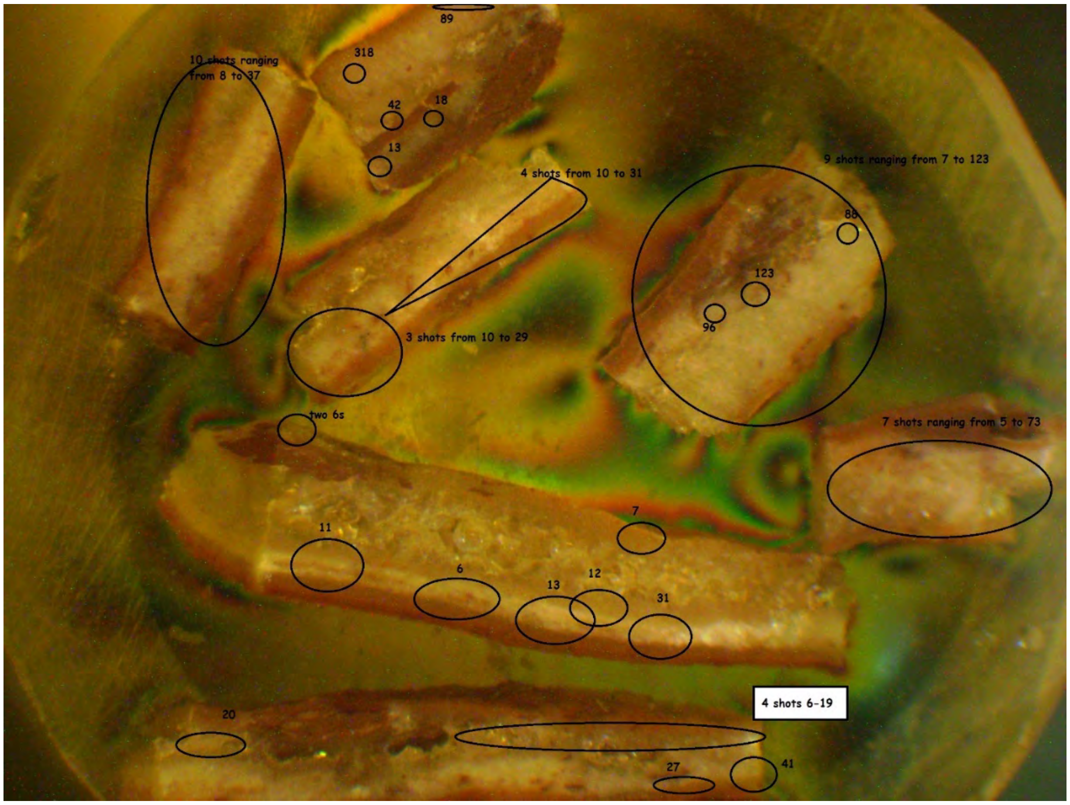
89
318
10 shots ranging from 8 to 37
42
18
13
4 shots from 10 to 31
9 shots ranging from 7 to 123
123
96
3 shots from 10 to 29
two 6s
7 shots ranging from 5 to 73
7
11
6
13
12
31
4 shots 6-19
20
27
41

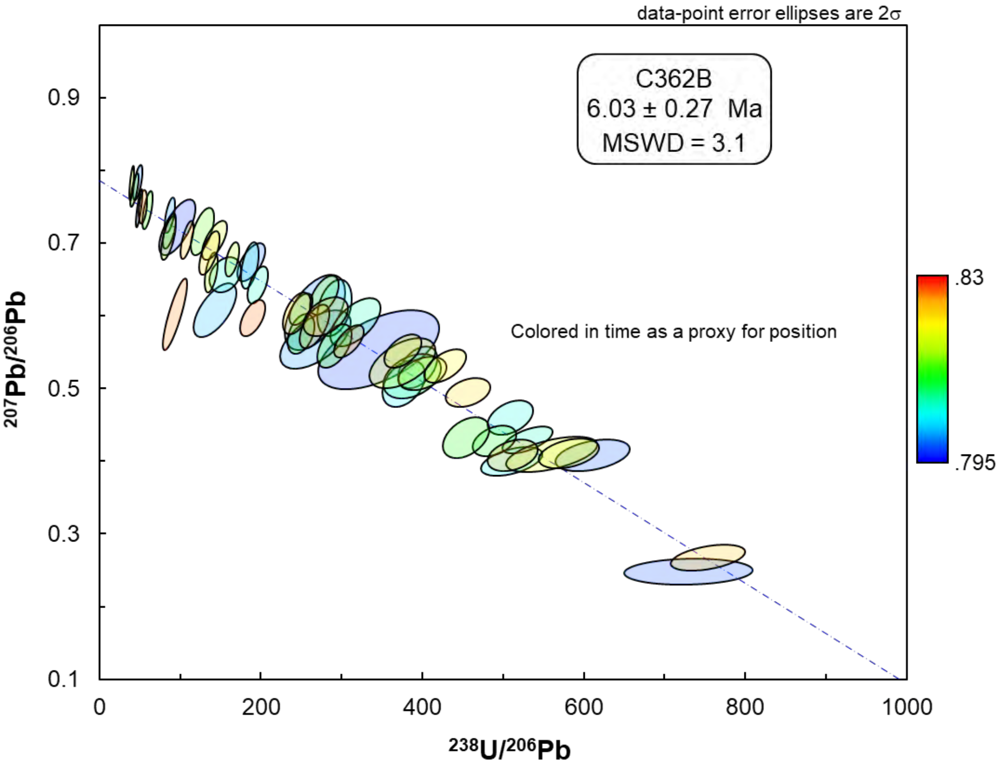
data-point error ellipses are 2σ
C362B
6.03 ± 0.27 Ma
MSWD = 3.1
Colored in time as a proxy for position
.83
.795
0.9
0.7
0.5
0.3
0.1
0
200
400
600
800
1000
^{207}Pb/^{206}Pb
^{238}U/^{206}Pb
