# Supplementary material for: Pace of passive margin tectonism revealed by U-Pb dating of fracture-filling calcite
Source: Nat Commun. 2022 Apr 12;13:1953. doi: 10.1038/s41467-022-29680-z (PMC9005700; doi:10.1038/s41467-022-29680-z)
Supplement: Supplementary file 6 — Source Data [file 41467_2022_29680_MOESM6_ESM.zip › 2.zip!/appendix2b_site_summaries_21to50/30_meriden_target_final.pdf]

30: Meriden, CT behind Target (41.554011, -72.816590)

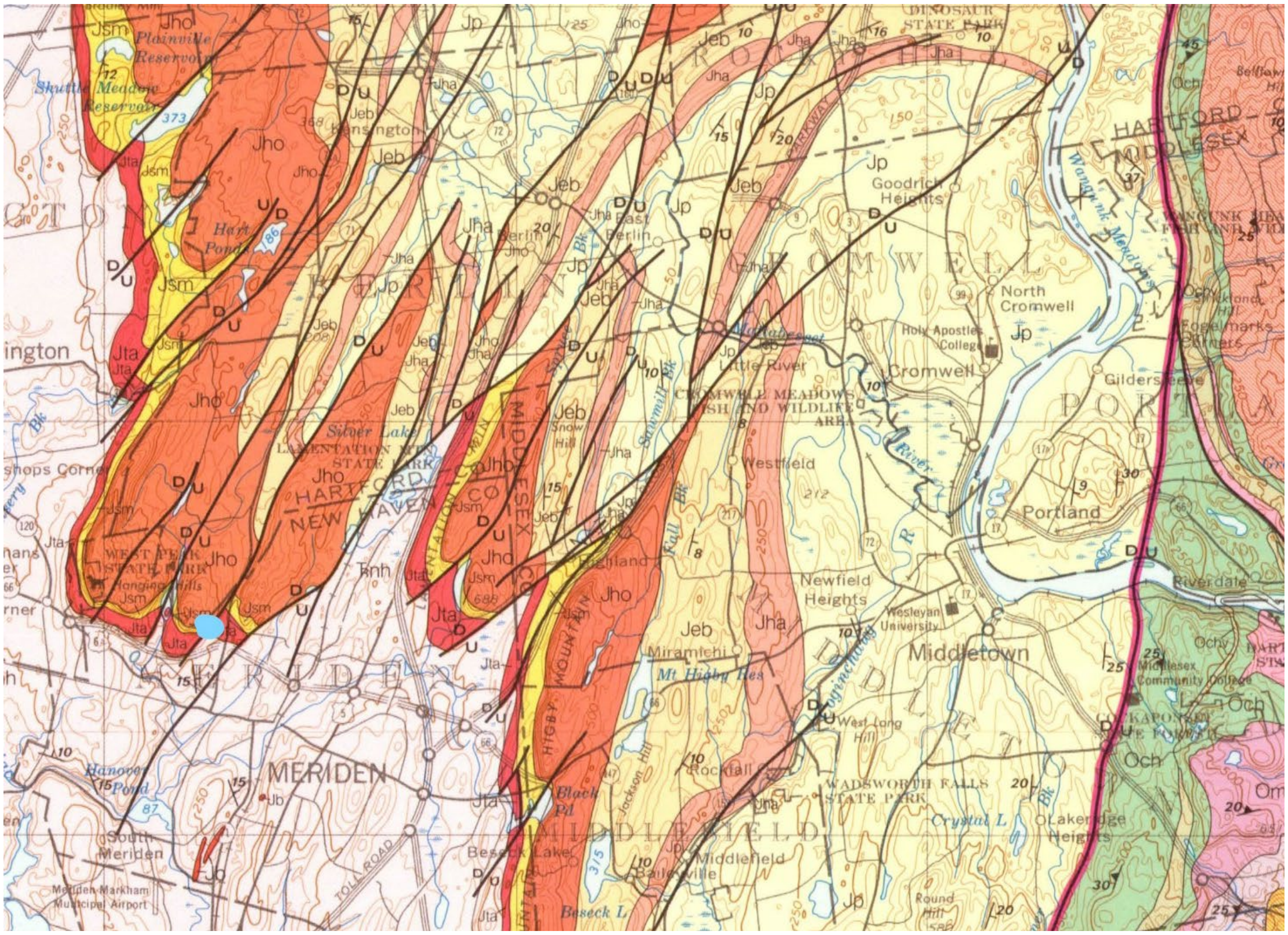

<u>Dated samples</u>: C366A +C363A

<u>Failed samples from same site:</u> C364A, C365A, C367A, C368A,C369A,C370A

<u>Ages</u>: C366A: 0.04 ± 0.32 Ma **C363A**: 3.7 ± 2.5 Ma

Summary: Fault zone exposed behind the Target in Meriden, CT. A set of high angle oblique slip surfaces cuts through the Talcott Basalt with abundant mineralization within the fault zones. Average strike is about 205 degrees with two surfaces containing slickensides dipping 30 degrees down from NE, suggesting oblique slip. Both ages yield single domain U-Pb isochron ages suggestive of a single event, but the extremely young ages and the low quality of the isochrons leave the ages highly uncertain,. This site is suggestive of Plio-Pleistocene tectonism, but needs further study to verify the ages are primary and establish the kinematics and stress regime.

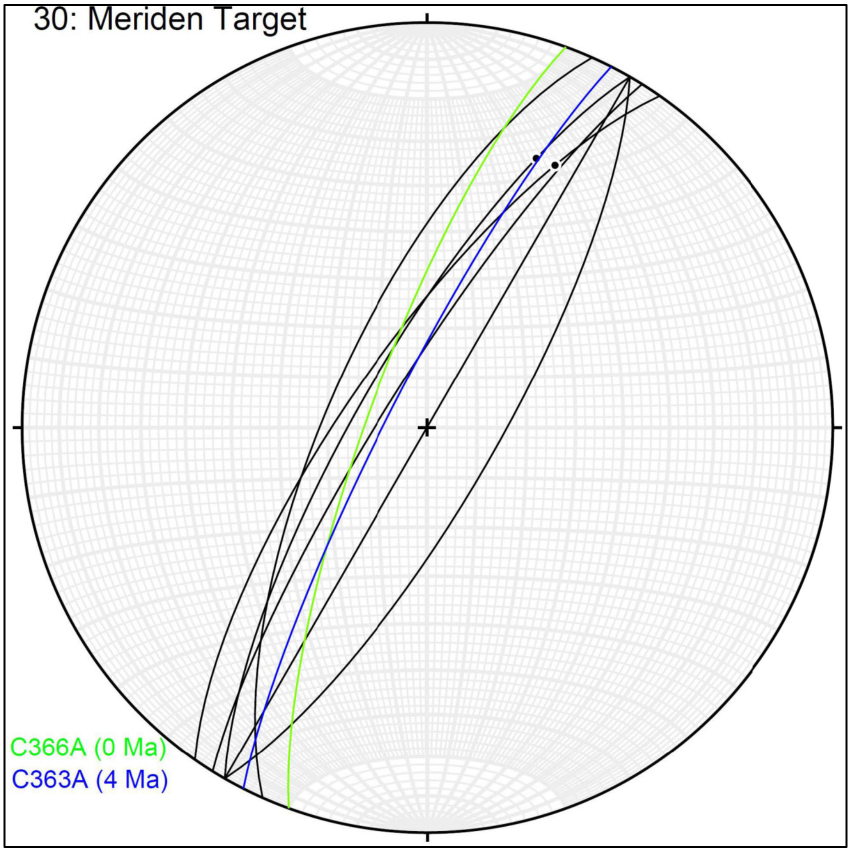
30: Meriden Target
C366A (0 Ma)
C363A (4 Ma)

<u>Field Descriptions</u>:

C363A:

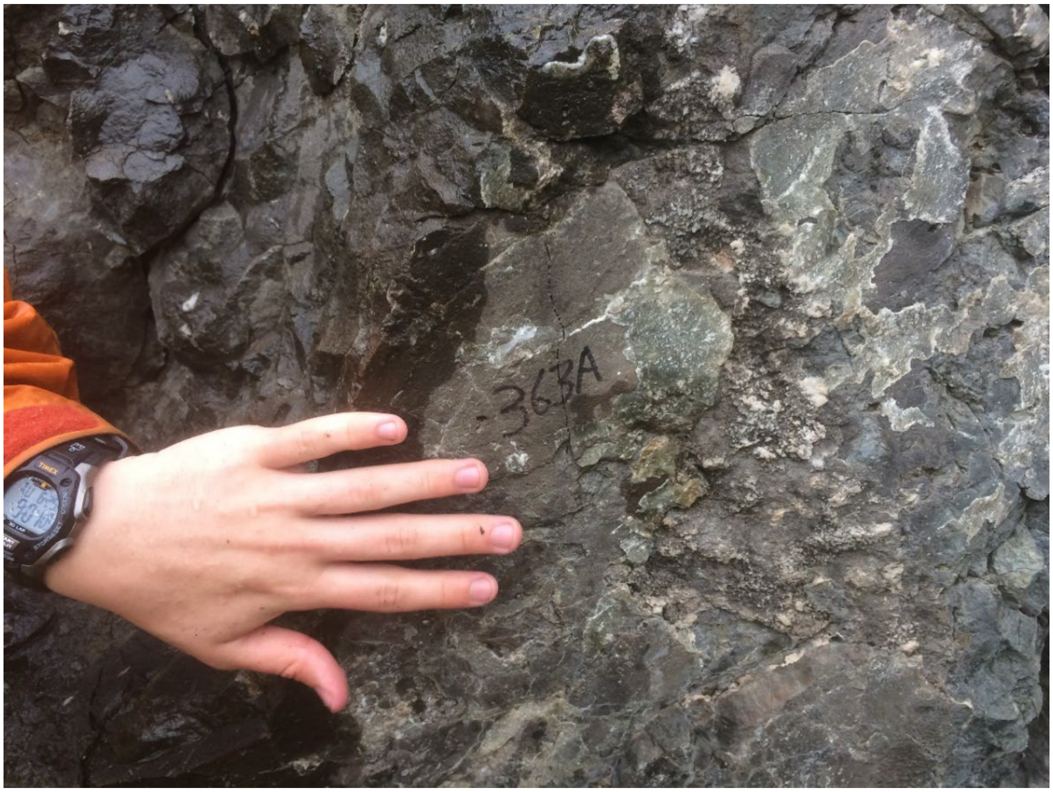

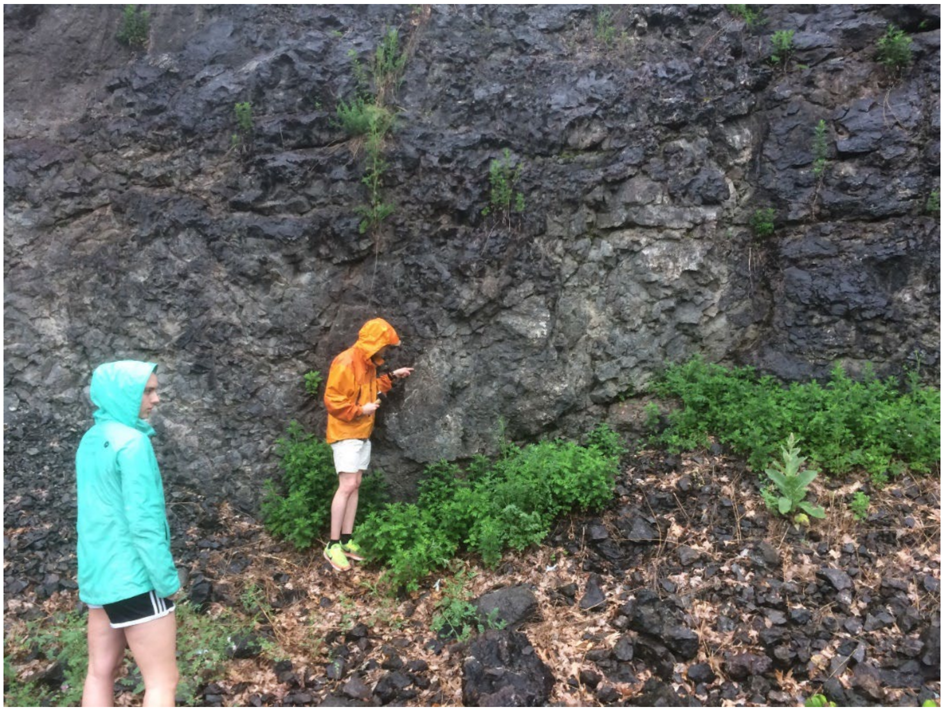

C366A:

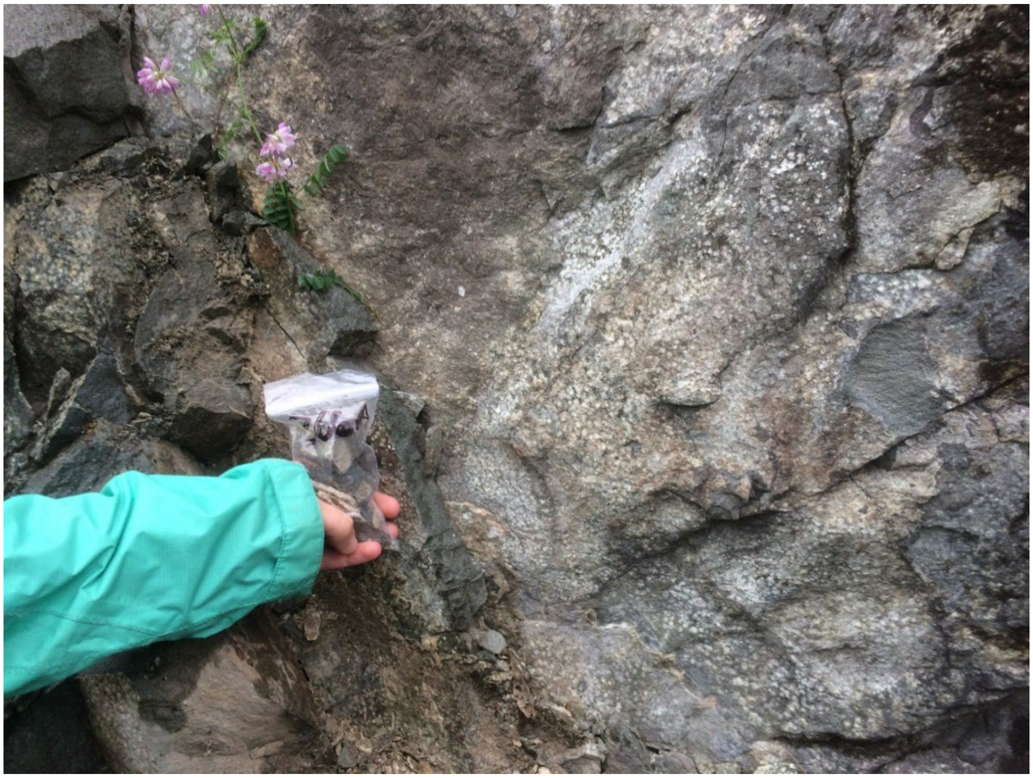

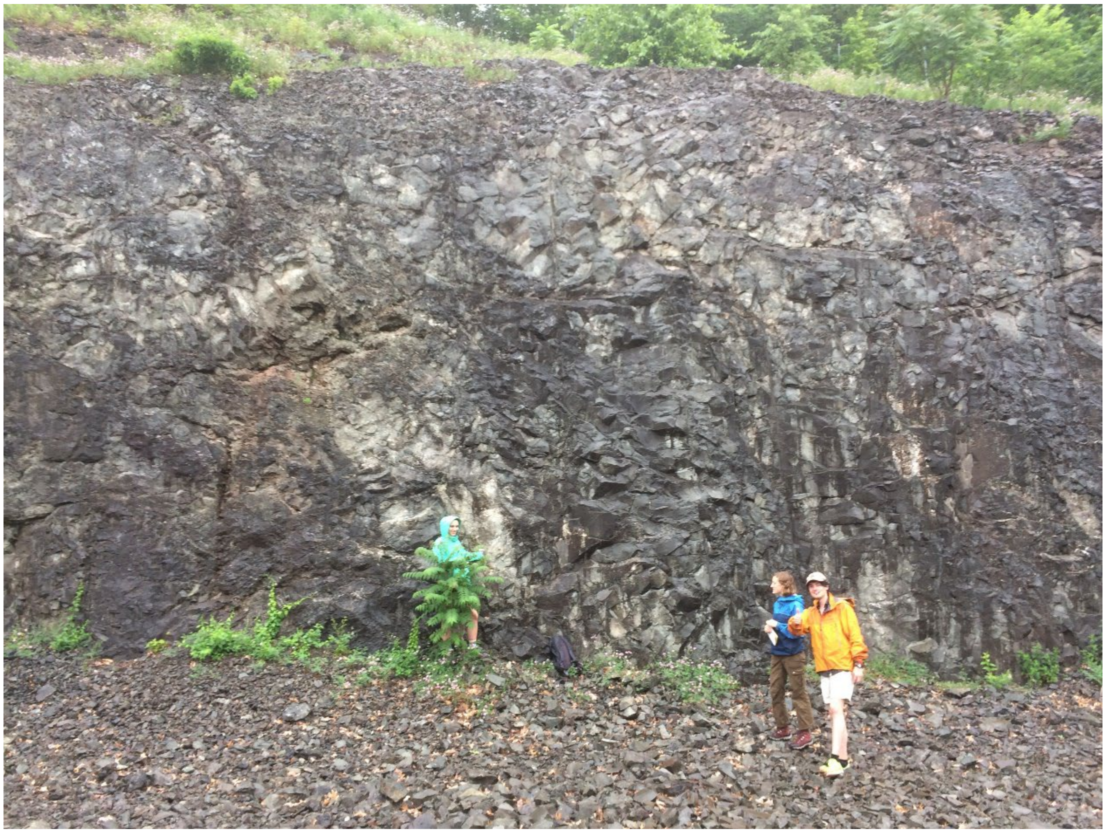

Lab Description:

363A: circles in image denote areas of interest, not actual laser shots

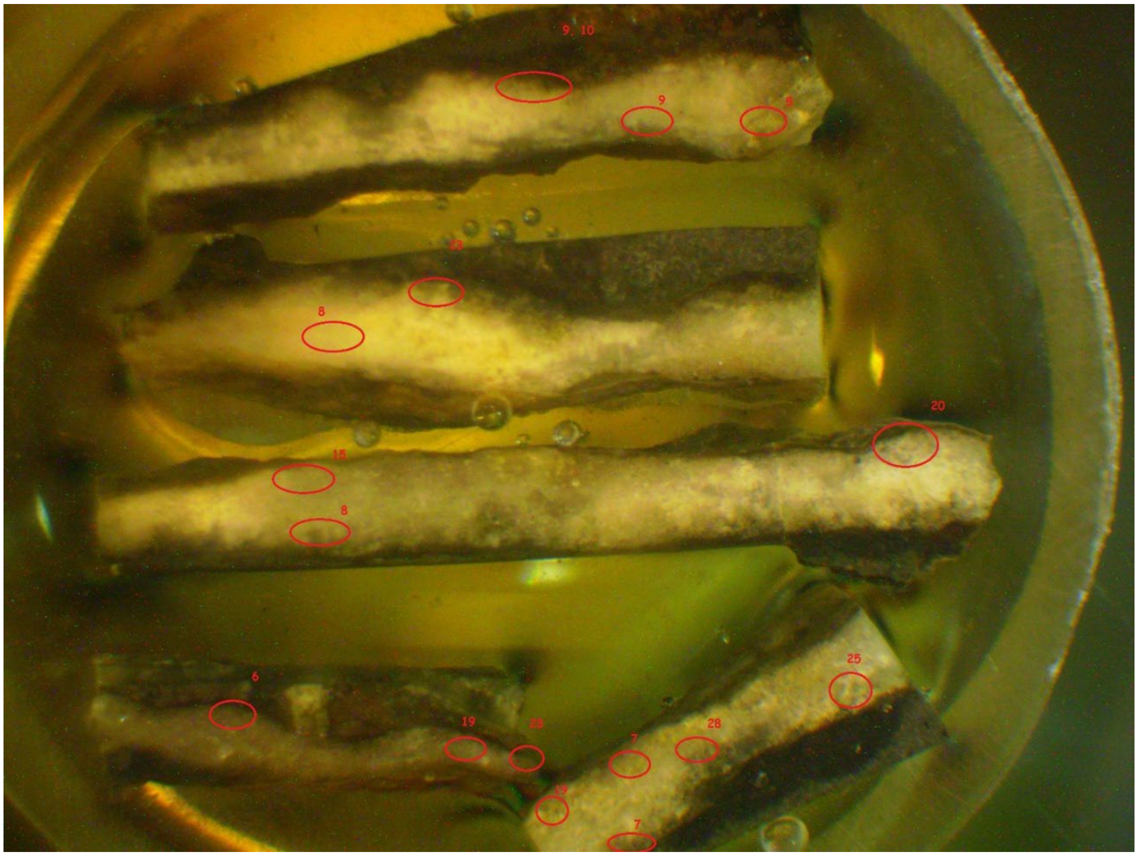

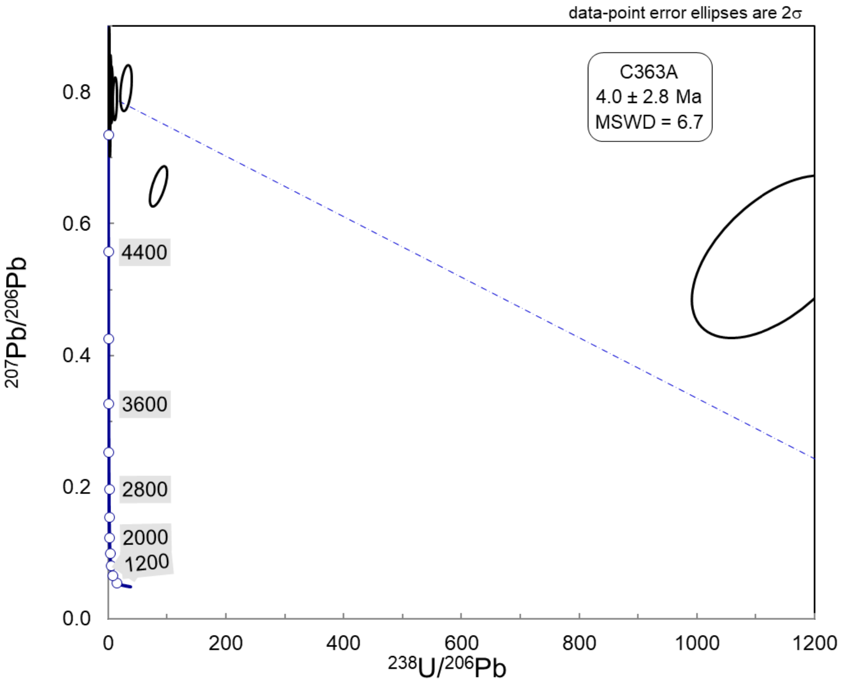

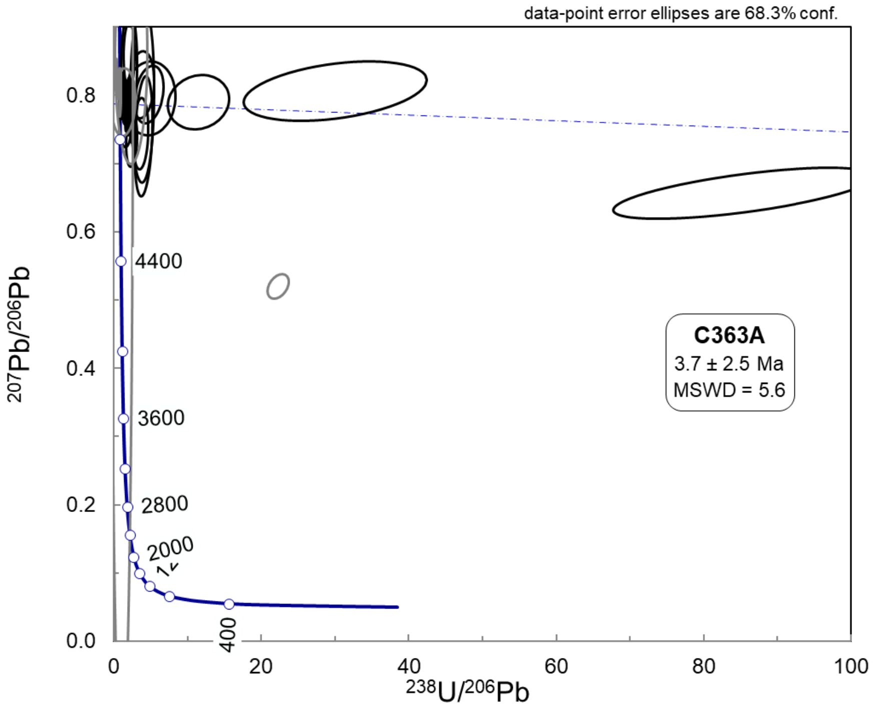

Results from C363A showing only shots with $^{238}U/^{206}Pb$ ratios <100.

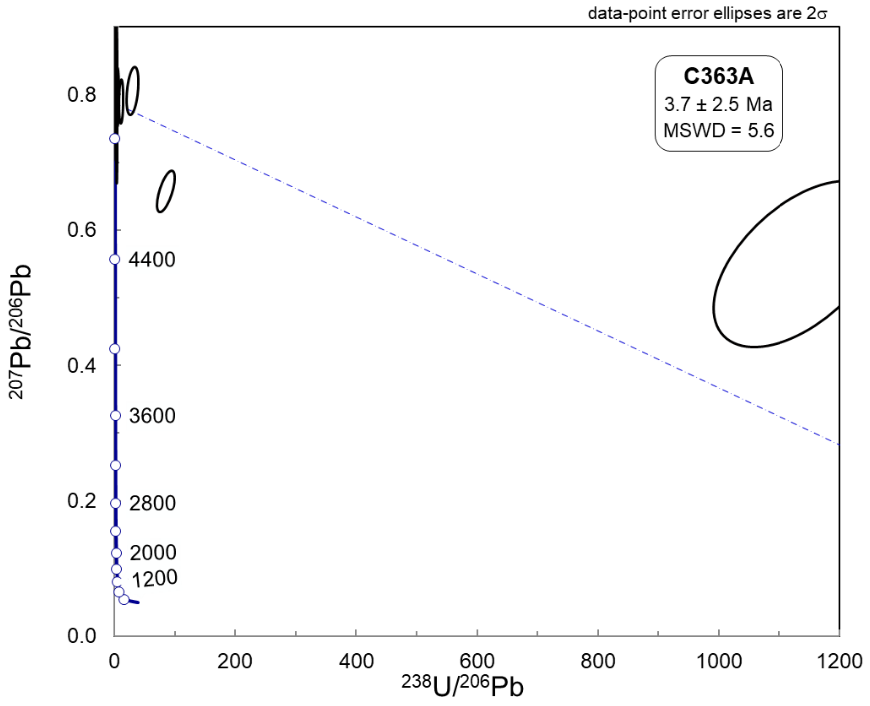

Results from C363A showing all accepted shots

366A: circles in image denote areas of interest, not actual laser shots

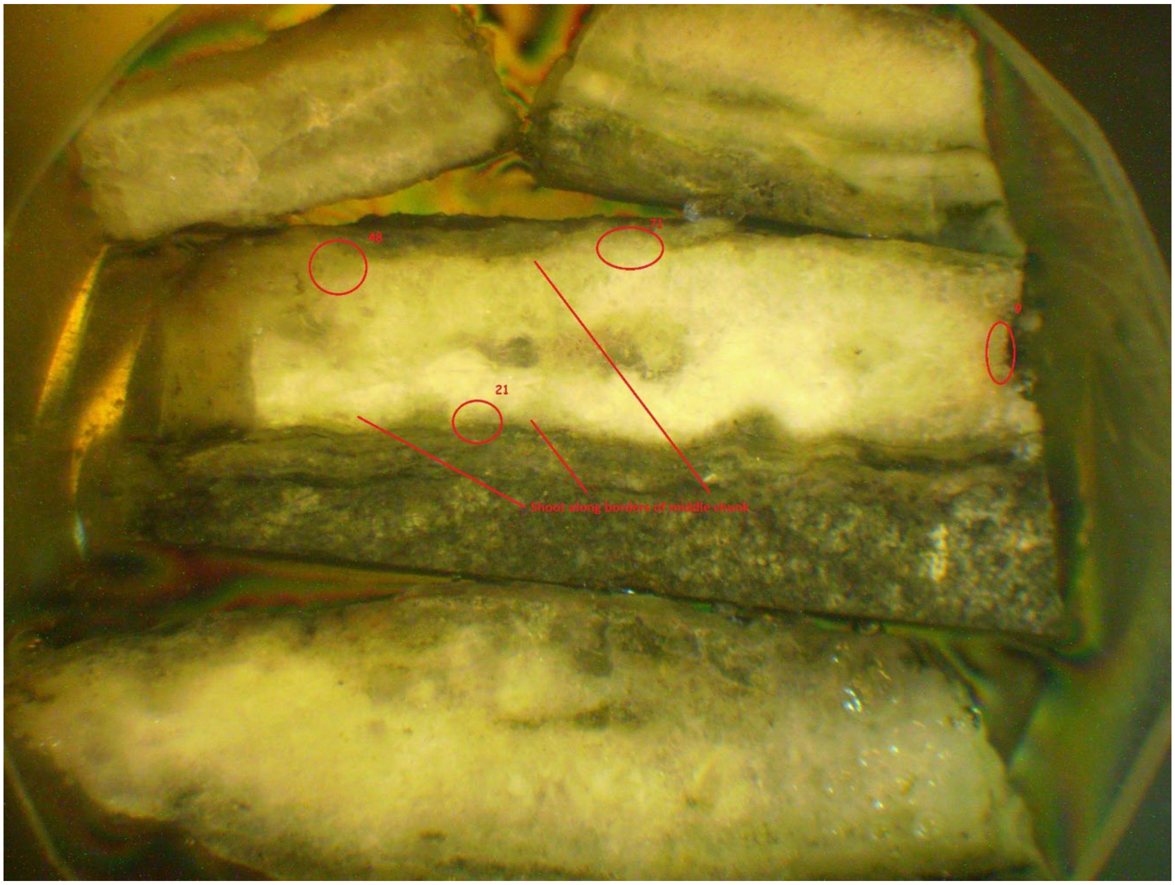

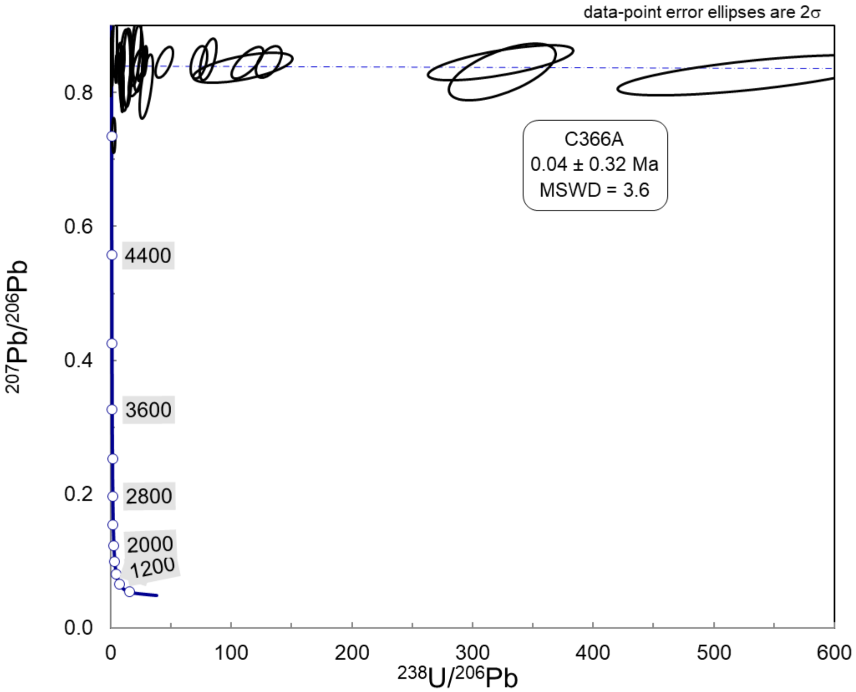

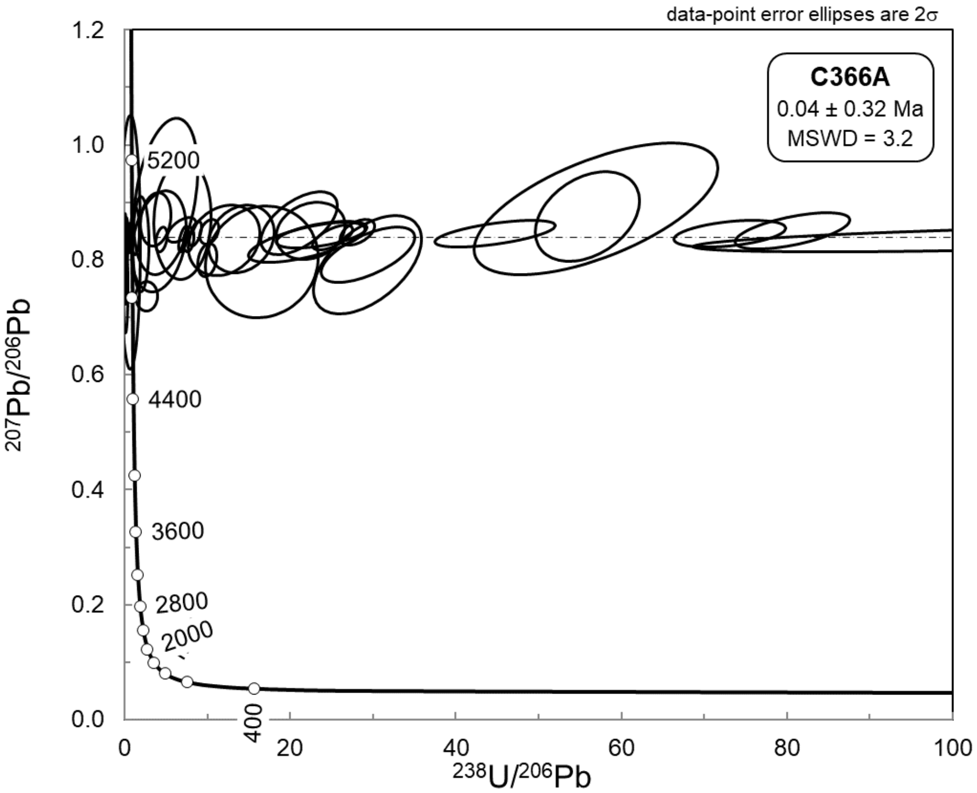

Results from C366A, showing only shots with $^{238}U/^{206}Pb$ ratios <100.
