# Supplementary material for: Pace of passive margin tectonism revealed by U-Pb dating of fracture-filling calcite
Source: Nat Commun. 2022 Apr 12;13:1953. doi: 10.1038/s41467-022-29680-z (PMC9005700; doi:10.1038/s41467-022-29680-z)
Supplement: Supplementary file 6 — Source Data [file 41467_2022_29680_MOESM6_ESM.zip › 2.zip!/appendix2b_site_summaries_21to50/31_shelburne_shipyard_final.pdf]

Site 31: Shelburne Shipyard (44.433168, -73.251756)

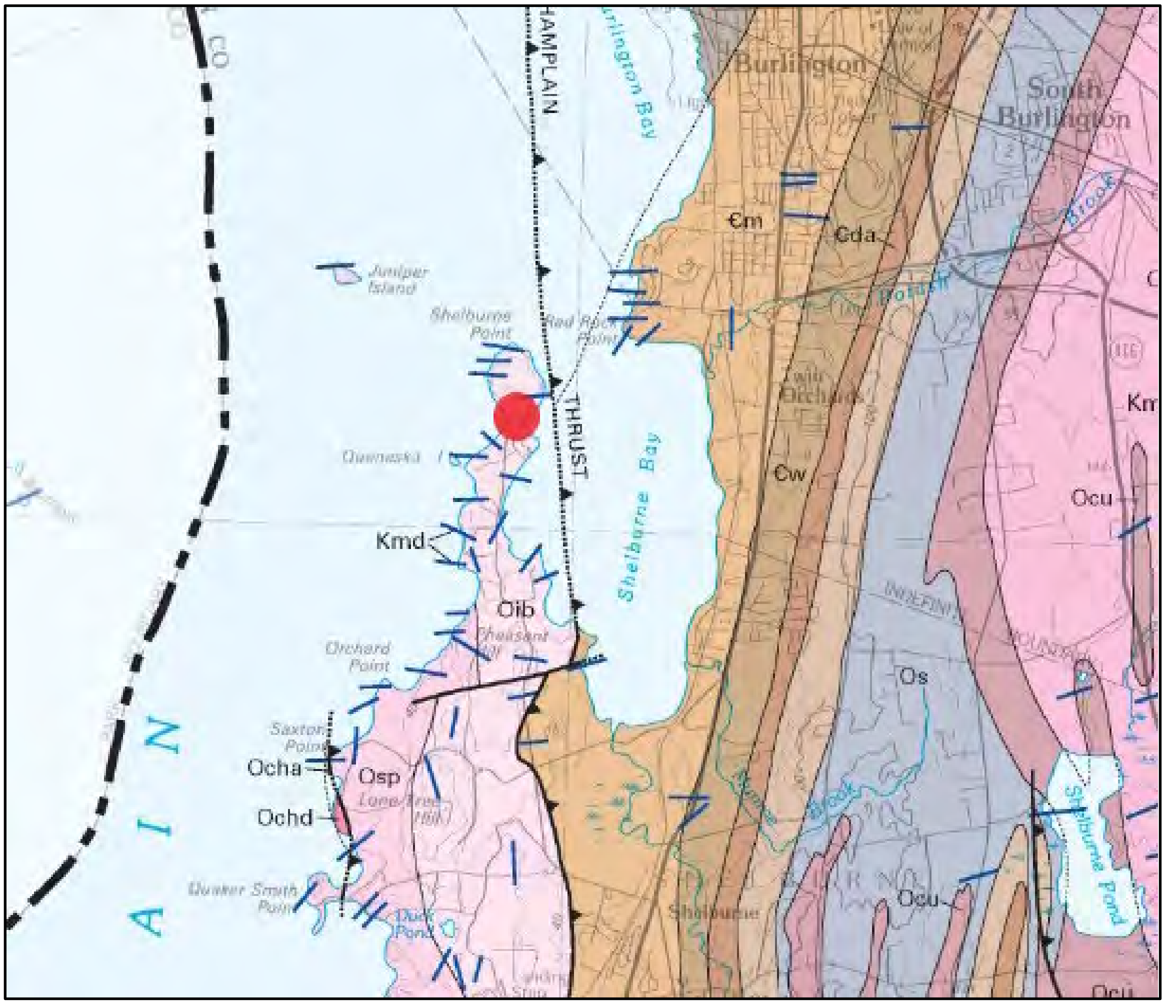

Figure 1: Sample location on state geologic map of Ratcliffe at al. (2011)

Bedrock Unit: Iberville Formation (Oib)

Failed samples from same site: C388A, B, E

Ages: **C388C**: 0.7 +/- 3.0 Ma **C388D**: 0.6 ± 0.3 Ma

Summary: This site sits on the west shore of Shelburne Point and can be reached by parking at or near the Shelburne Shipyard and walking south along the gravel beach just west of the main road. Three samples were collected from a north-plunging fold that is cross-cut by a dominant S1 and weaker S2 cleavage (see figure 2 below). Many of the S2 cleavage planes have been reactivated as mode 1 fractures and contain 1-3 mm thick syntaxial calcite veins dated to the Plio-Pleistocene time period. These veins occur only within stronger beds, terminating abruptly against weaker beds where strain was likely accommodated by cleavage-parallel slip along the more shallow-dipping S1 cleavage, which post-dates the folding but predates mineralization of the axial planar cleavage. No slickensides or growth strata are present on the mineralized fractures, thus their interpretation as mode 1 structures.

Two dated samples (C388C and C388D) collected from the reactivated S2 fractures suggest a crystallization age between roughly 0.5 and 1 Ma. These dates are inferred primarily from laser shots taken from a brownish zone of syntaxial fabric visible within ~500 um of the wall rock and smaller 'hairline' veins containing transluscent calcite (see microscope photos below). Based on the well-preserved syntaxial fabric we interpret this as a primary crystallization age. This idea is supported by the two 'clean' isochron plots that lack widely scattered shots and yield ages in excellent agreement with ach other. However, we cannot rule out the possibility of a recrystallization event within porous(?) fabrics along the wall rock boundary. If interpreted as primary syntaxial mode-1 veins, these veins record an E-W $\sigma 3$ stress.

Field Descriptions:

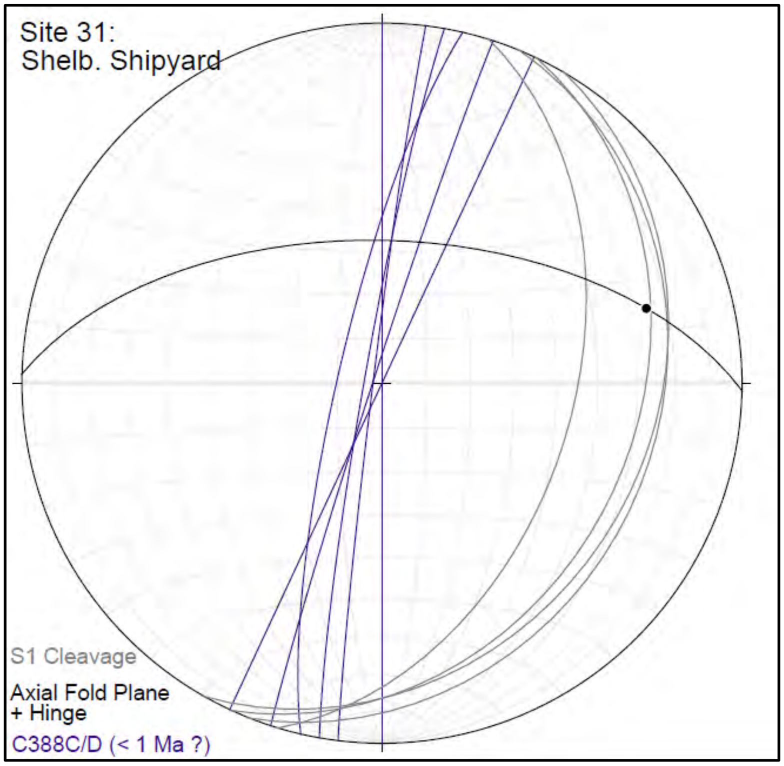

Figure 2: Structural data from site 31. Axial fold plane and hinge are computed in Stereoplot software from 7 bedding plane measurements.

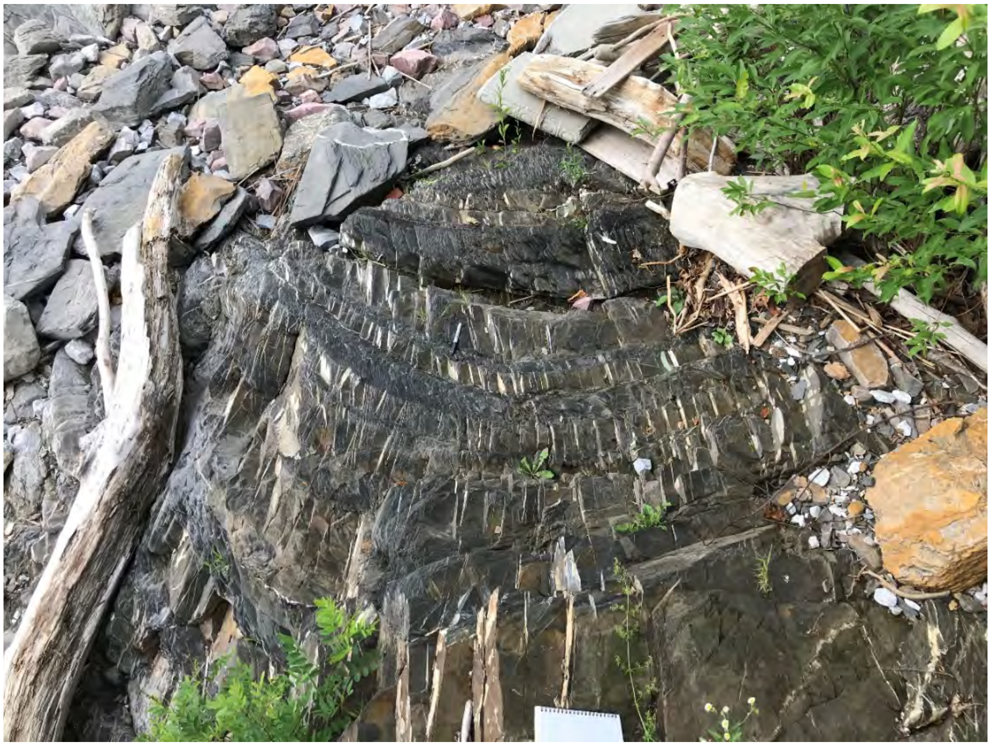

Figure 3*: Overhead view of plunging fold with axial planar cleavage reactivated as mode-1 dilational fractures and filled with 0.3 to 2 mm of calcite. Note that calcite veins do not continue through weaker beds where strain was accommodated by cleavage plain slip along the S1 cleavage.*

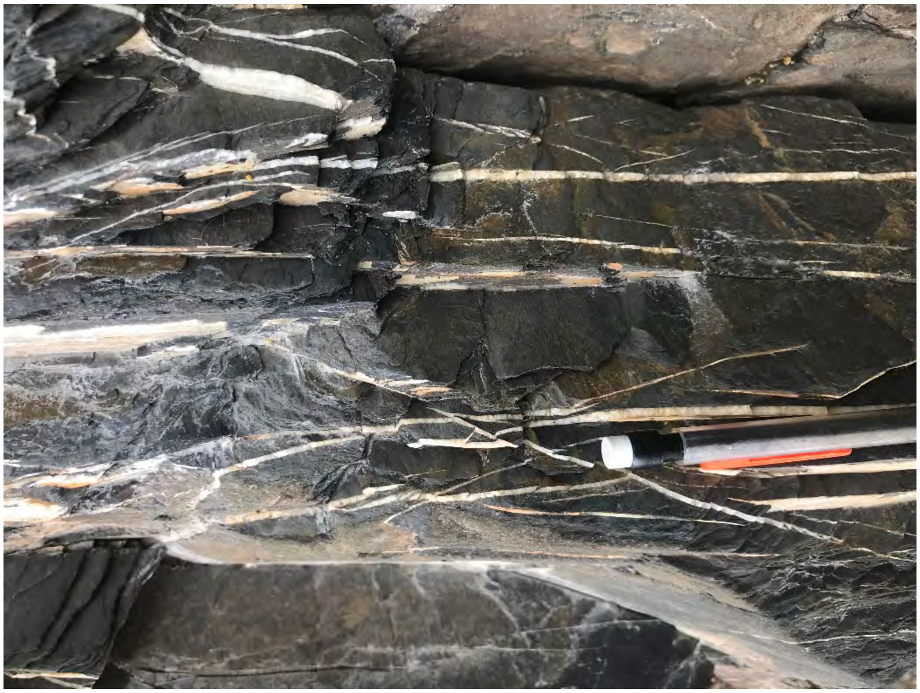

Figure 4: Intersection of two late-stage mode 1 vein sets. In this photo the NNE-SSW set seems to post-date the roughly N-S set, although the opposite relationship was observed elsewhere in the outcrop suggesting coeval formation of the two vein sets. No slickensides or growth striae are present on either set of veins.

C388C:

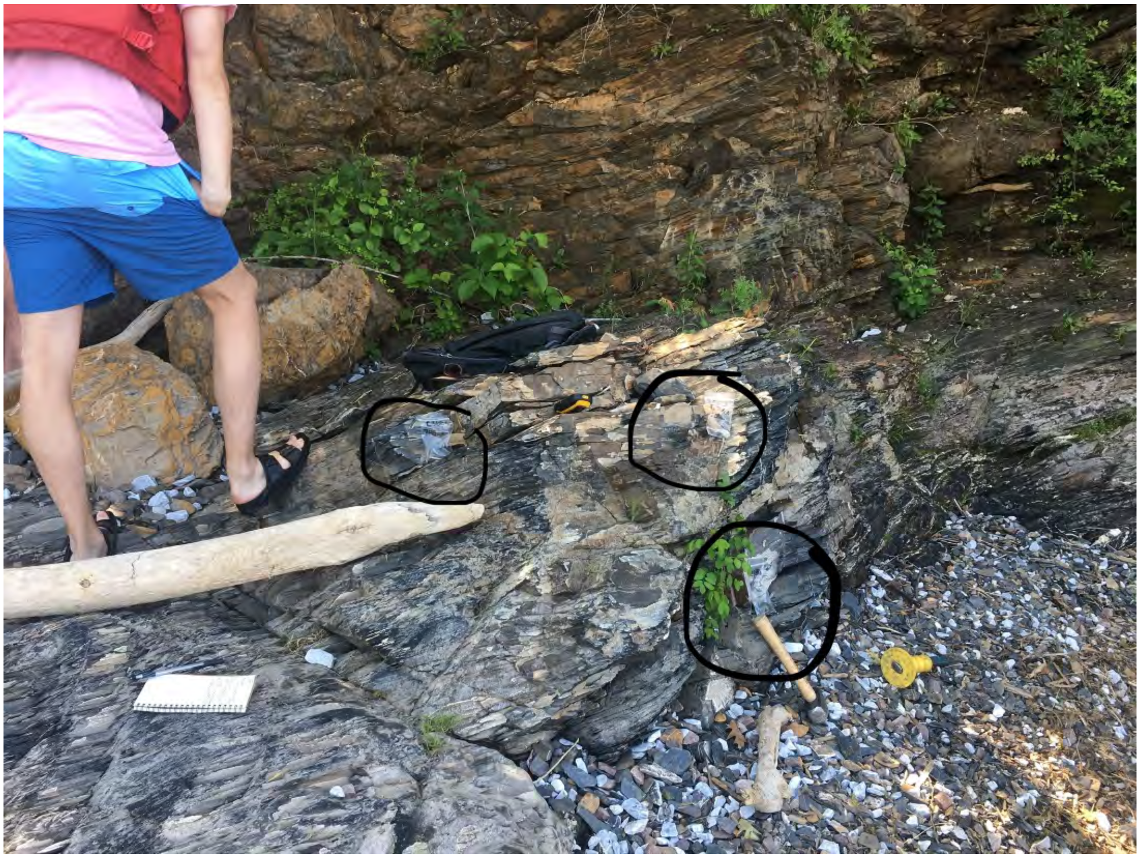

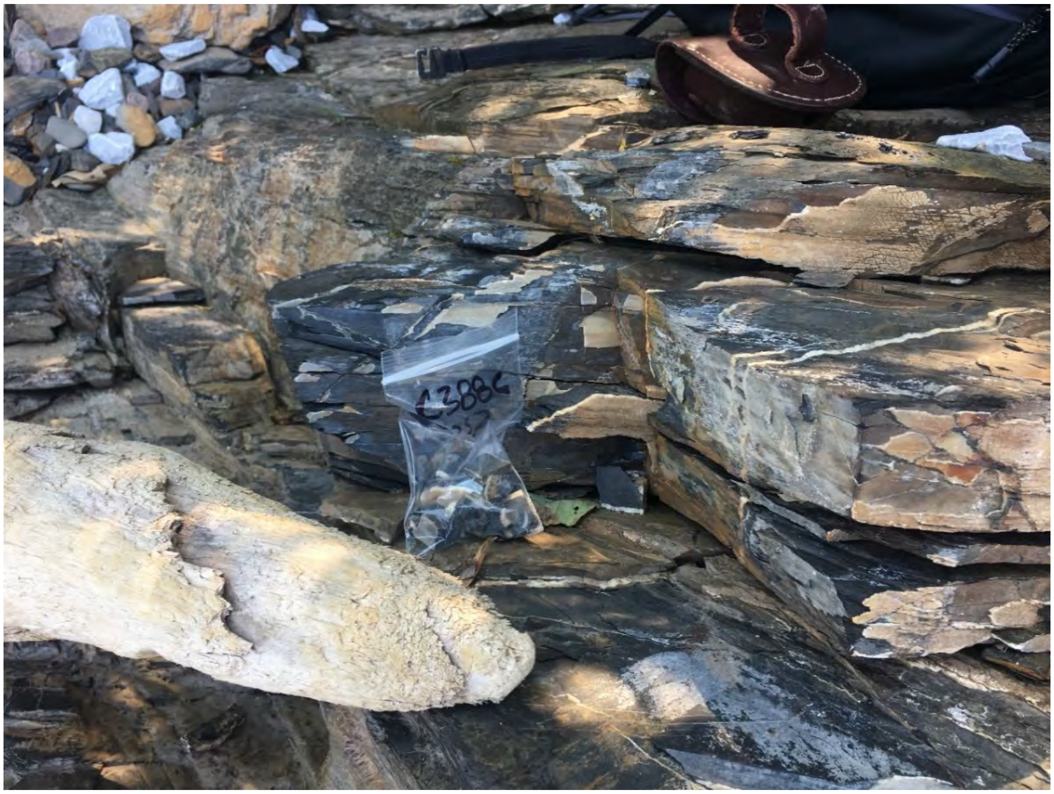

C388D:

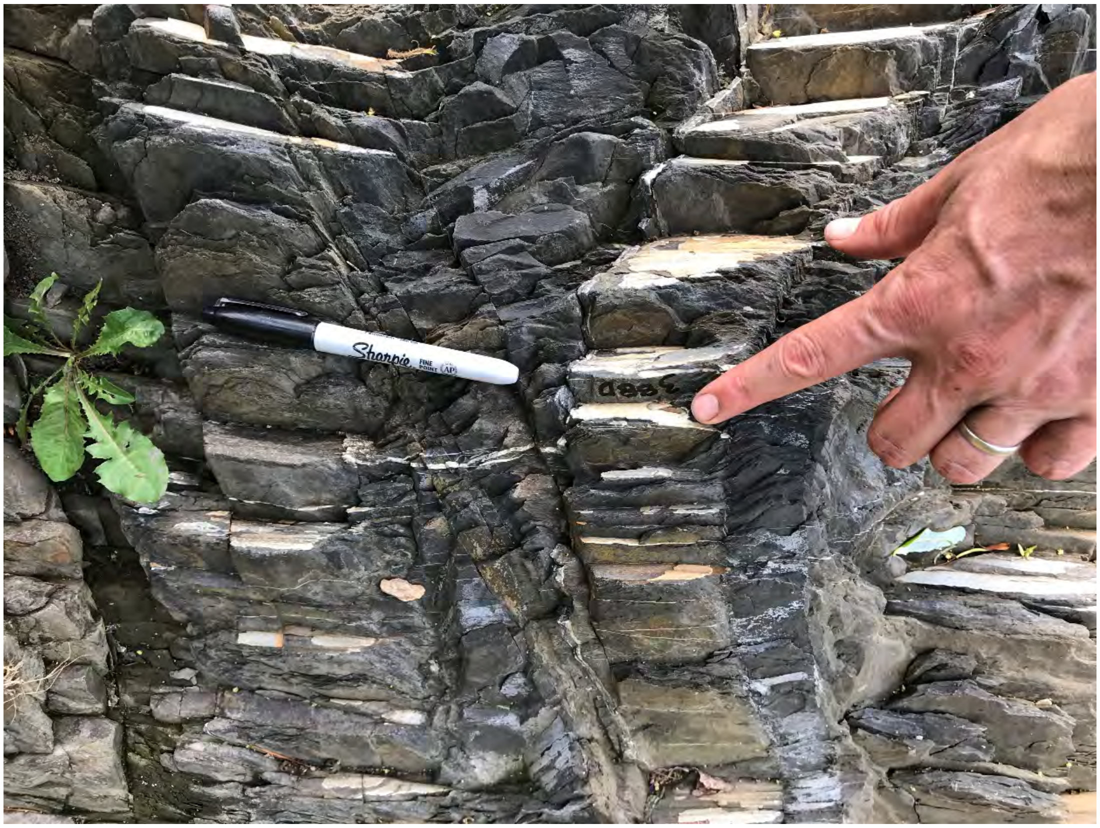

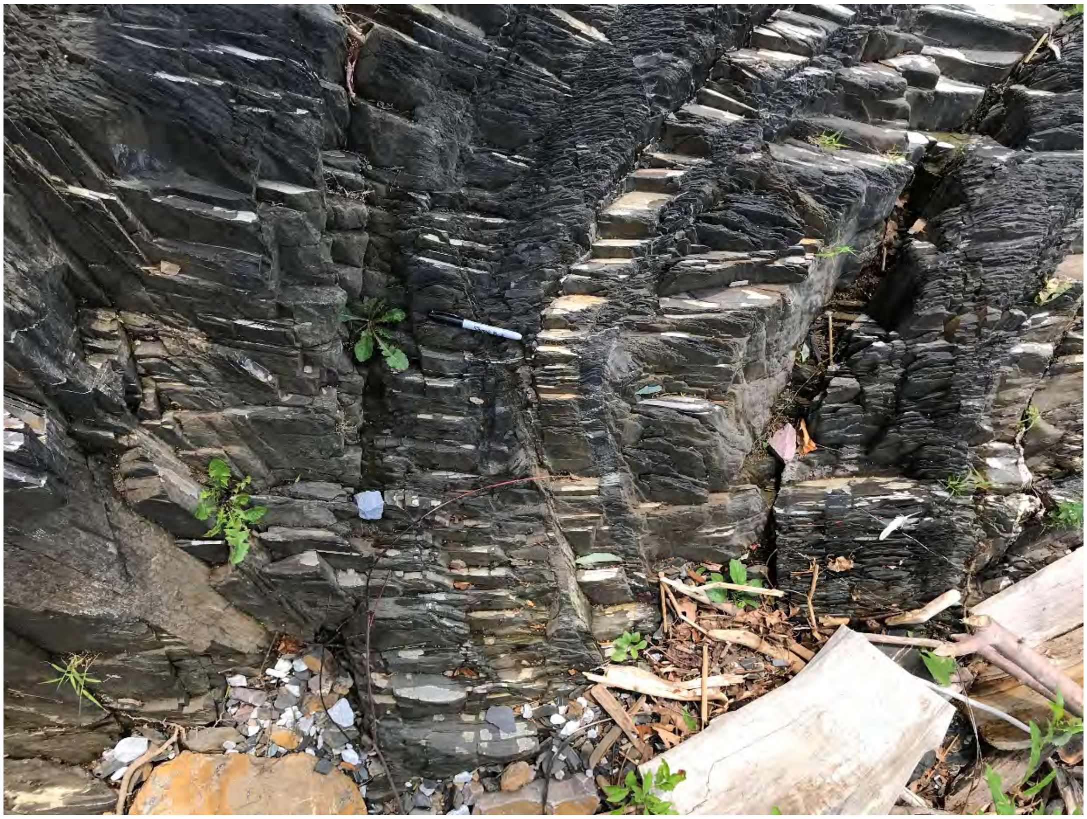

Lab Description:

C388C: circles denpt

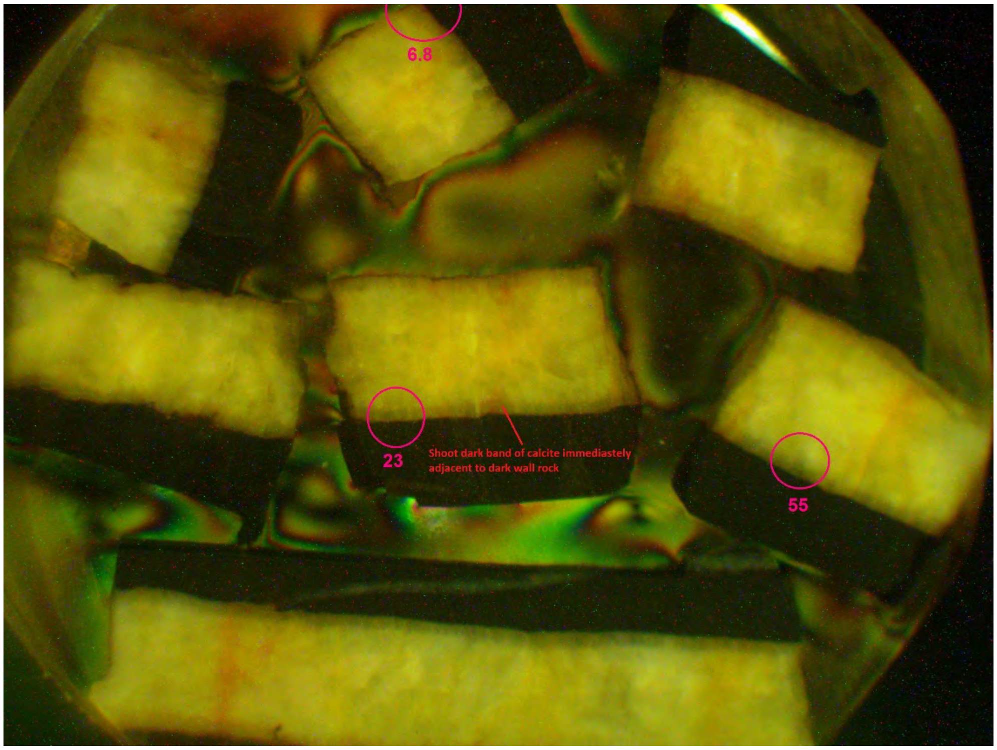

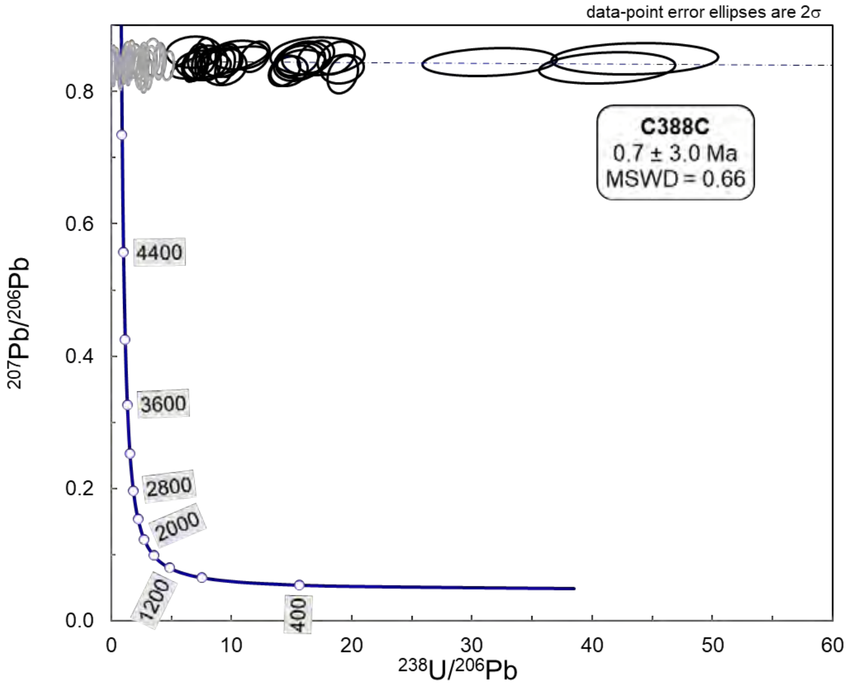

C388D:

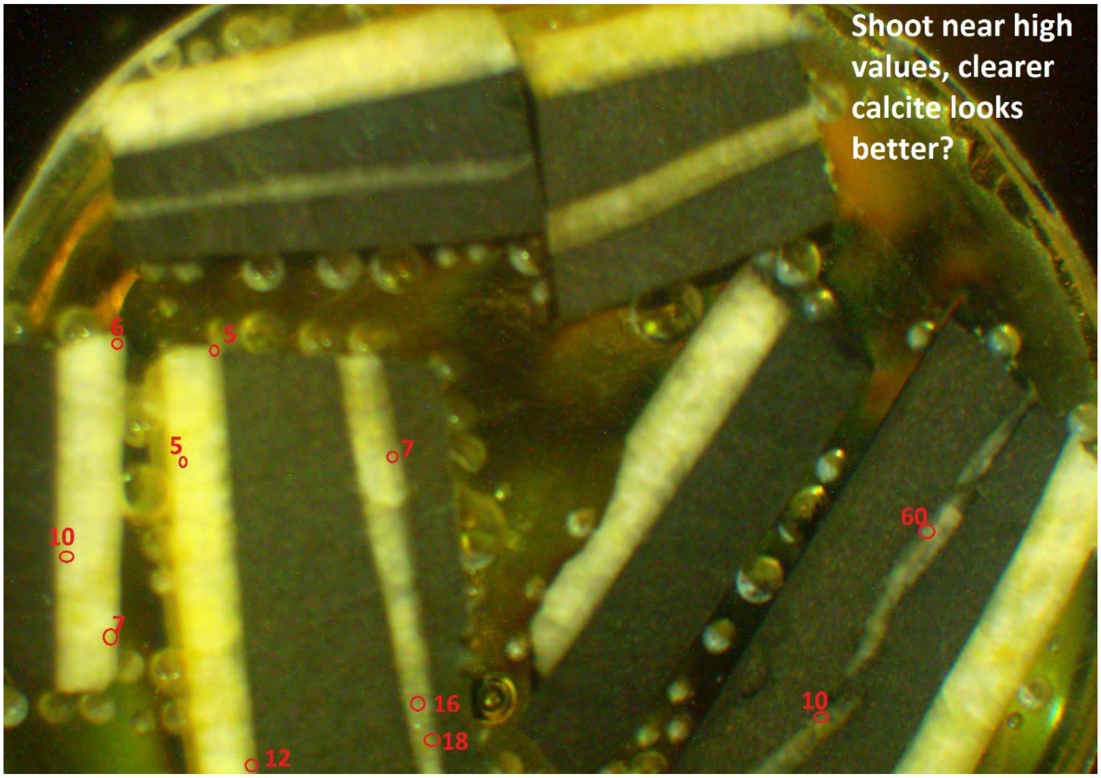
Shoot near high values, clearer calcite looks better?
6
5
5
7
10
7
60
16
18
12
10

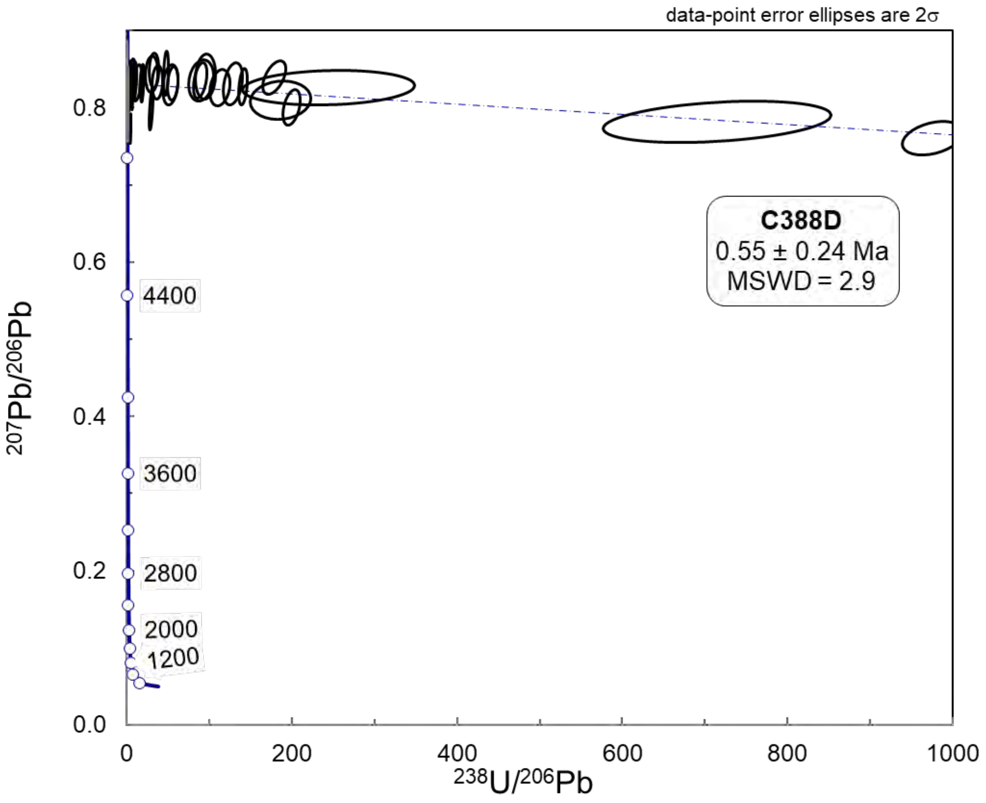
data-point error ellipses are 2σ
C388D
0.55 ± 0.24 Ma
MSWD = 2.9
4400
3600
2800
2000
1200
0.8
0.6
0.4
0.2
0.0
0
200
400
600
800
1000
207Pb/206Pb
238U/206Pb
