# Supplementary material for: Pace of passive margin tectonism revealed by U-Pb dating of fracture-filling calcite
Source: Nat Commun. 2022 Apr 12;13:1953. doi: 10.1038/s41467-022-29680-z (PMC9005700; doi:10.1038/s41467-022-29680-z)

32: Queneska Island (west side Shelburne Pt.) (44.395035, -73.279024)

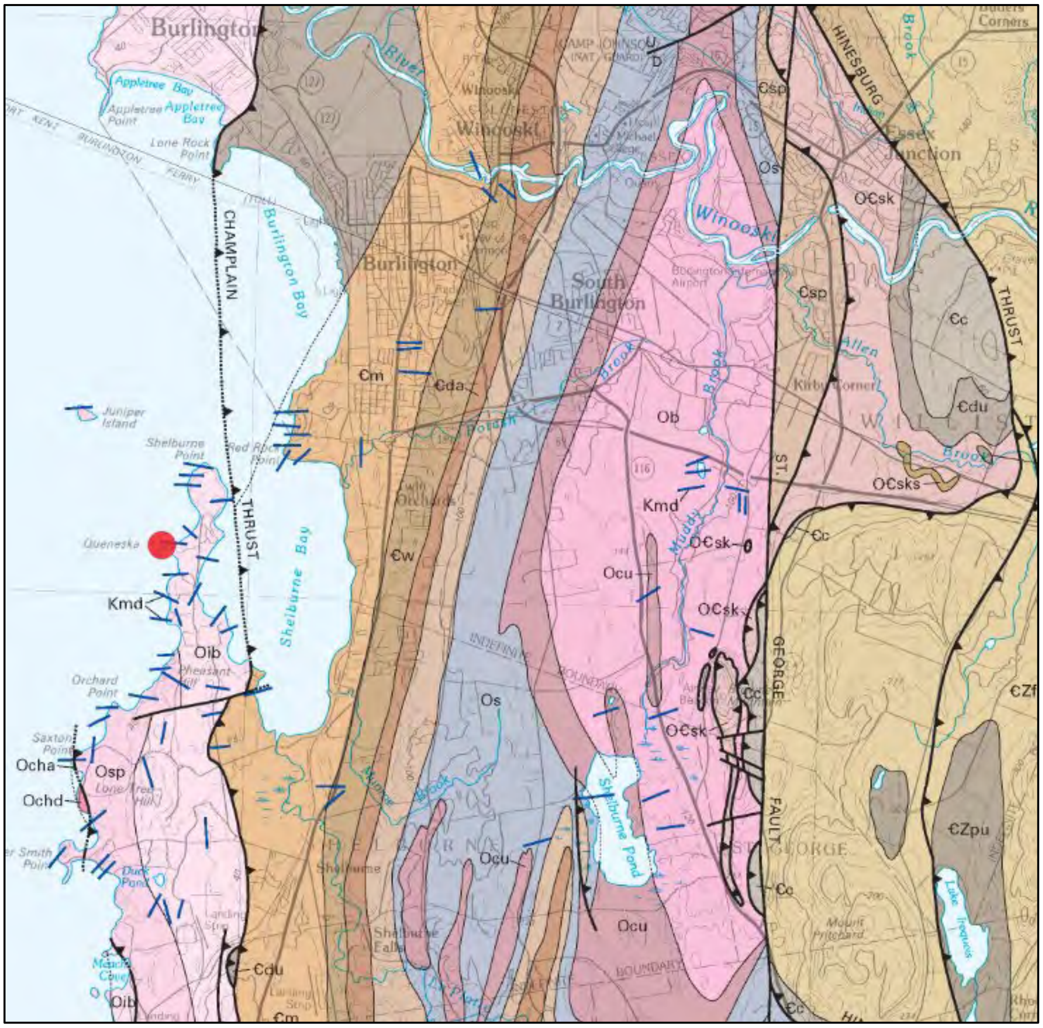

Figure 1: Sample location on state geologic map of Ratcliffe at al. (2011)

<u>Failed samples from same site:</u> C395B, C396, C397 (all on island)

<u>Bedrock Unit:</u> Iberville Formation (Oib)

<u>Ages</u>: C395A: 61.5 ± 5.7

<u>Summary</u>: This site sits on the south shore of Queneska island and is only accessible by boat. Sample was collected from a set of ultra-planar, parallel mode 1 veins, 1-2 cm in thick, with no slip indicators. Age of ~62 Ma is not commonly observed in the Champlain Valley, other than falls outside range of other ages observed in Champlain Valley. Texture of veins is also quite unique: perfect fibrous crystals oriented perpendicular to the wall rock in fashion often interpreted as antitaxial (growth from vein center towards wall). U-Pb yields a well-defined single isochron suggestive of single crystallization event. The face value interpretation of this site is a mode-1 fractures opening under an E-W oriented σ_3 stress, although more detailed study is warranted at this site.

Field Observations:

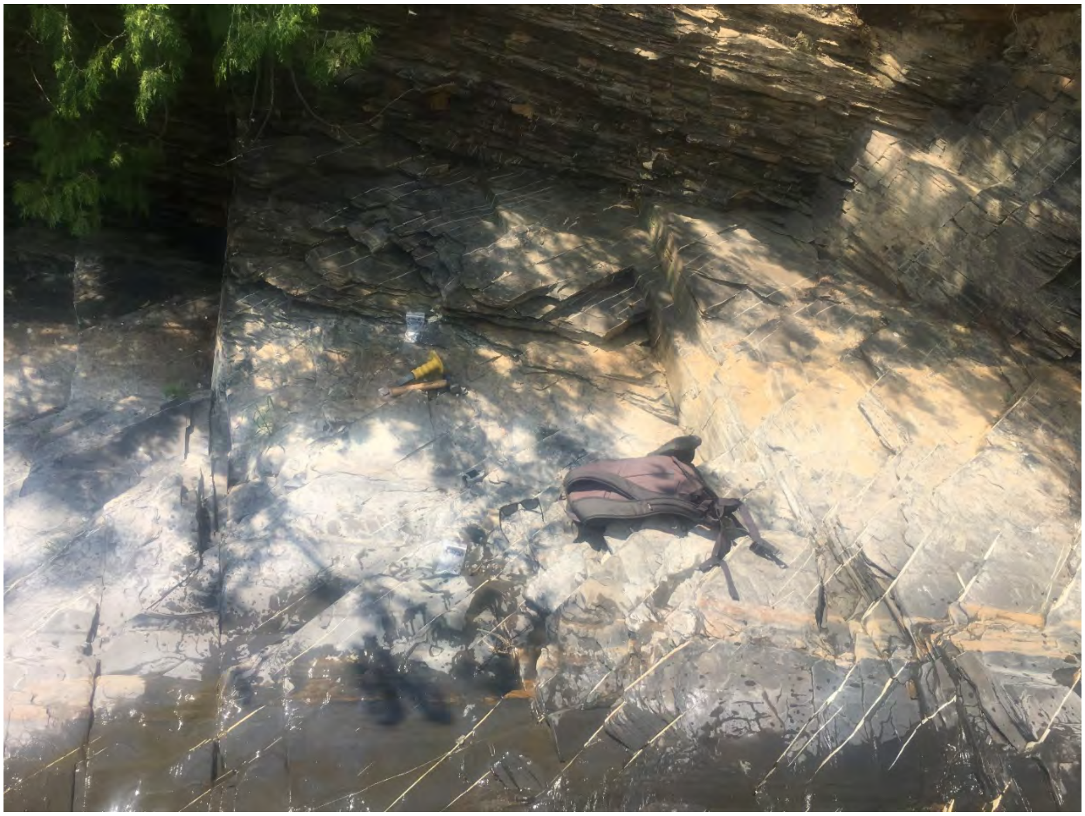

Lab Description:

C395A: Circles denote areas of interest, not actual laser shots

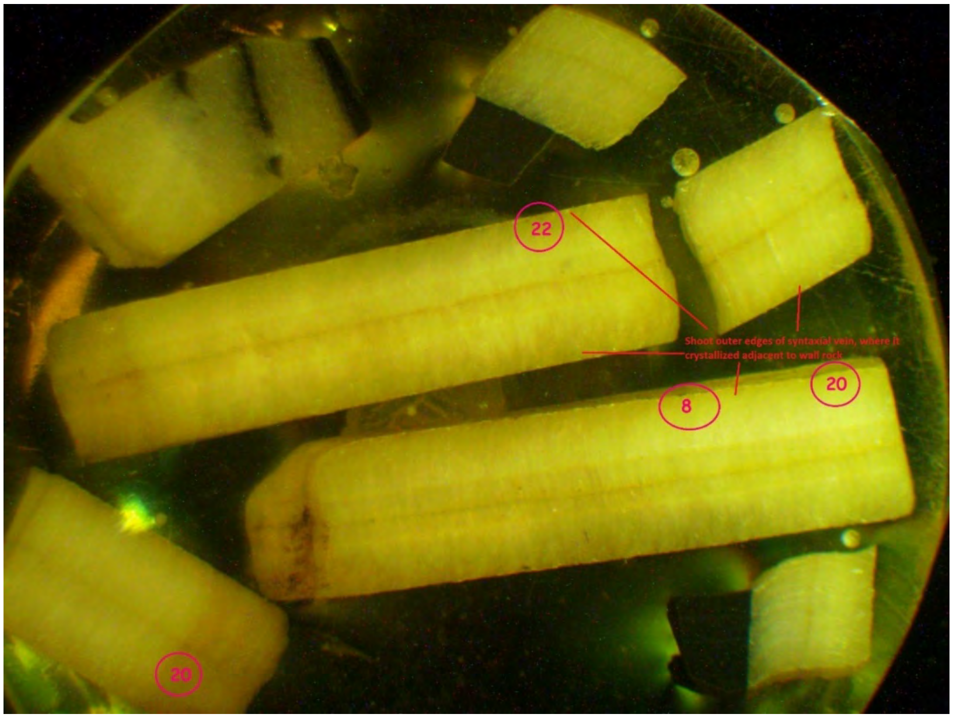

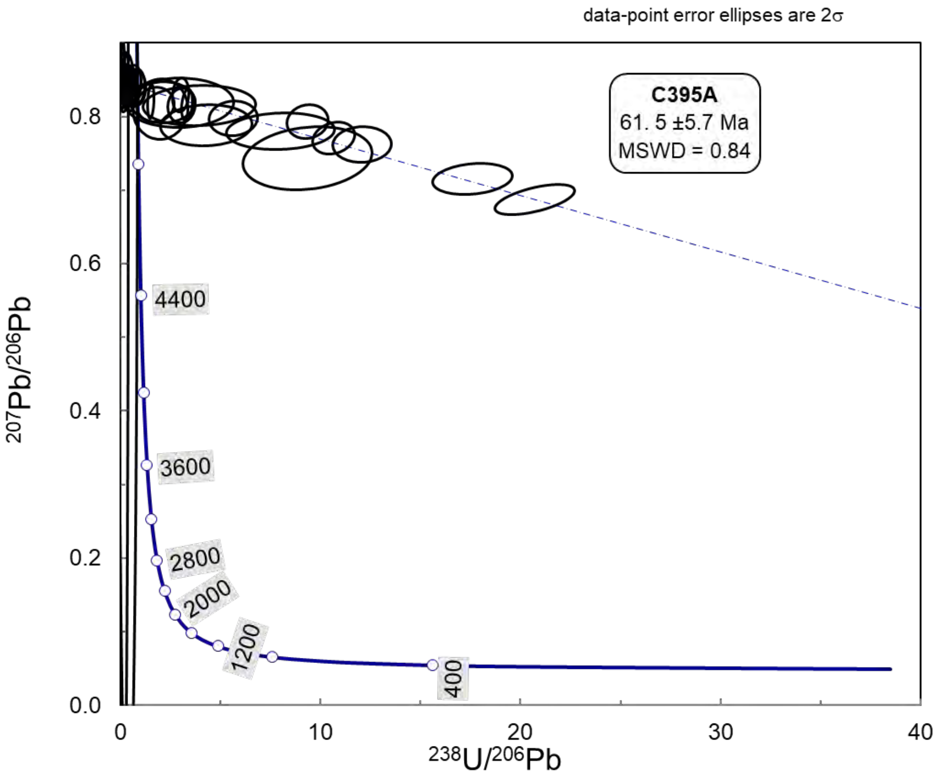

Supplement: Supplementary file 6 — Source Data [file 41467_2022_29680_MOESM6_ESM.zip › 2.zip!/appendix2b_site_summaries_21to50/32_queneska_island_final.pdf]
