# Supplementary material for: Pace of passive margin tectonism revealed by U-Pb dating of fracture-filling calcite
Source: Nat Commun. 2022 Apr 12;13:1953. doi: 10.1038/s41467-022-29680-z (PMC9005700; doi:10.1038/s41467-022-29680-z)
Supplement: Supplementary file 6 — Source Data [file 41467_2022_29680_MOESM6_ESM.zip › 2.zip!/appendix2b_site_summaries_21to50/33_charlotte_beach_final.pdf]

33: Charlotte Beach Dike (44.3341160, -73.2817540)

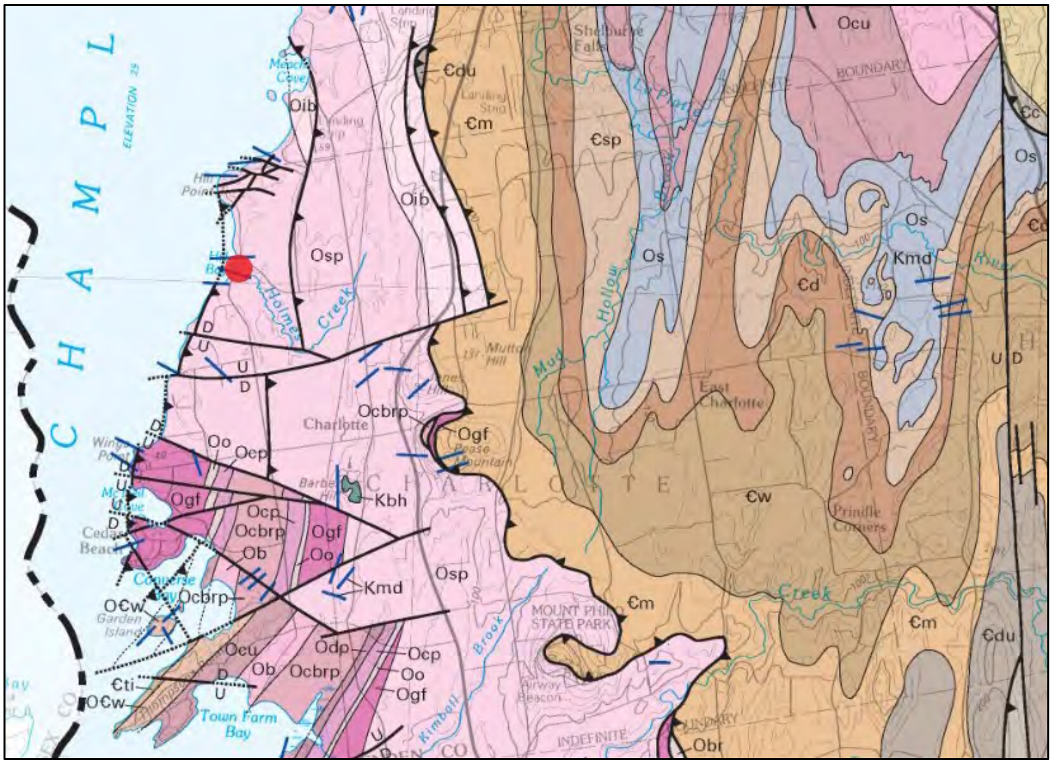

Figure 1: Sample location on state geologic map of Ratcliffe at al. (2011)

<u>Failed samples from same site:</u> C408C + C408D

<u>Bedrock Unit</u>: Trachyte dike cutting Stony Point Formation (Osp)

<u>Ages</u>: C408A: 83.6 ± 7.2 Ma ; **C408B:** 94.4 ± 4.5 Ma

Summary: This site is easy to access and make an amazing field trip stop! Large trachyte dike exposed at Charlotte Beach and cut by numerous minor faults, many containing calcite with slickensides and growth striae. Almost all of the faults are confined within the dike, presumably because it is more rigid than the surrounding shale. Sample C408B is a minor fault formed with growth striae trending ~45° and implying NE-SW extension records NE-SW extension at ~ 95 Ma (see figure 2 below). Sample C408A is another fault formed with slickensides trending roughly 270° implying E-W extension with a TTW sense of slip at ca. 84 Ma. This ~84 Ma fault (C408A) was subsequently truncated by a set of younger faults oriented NW-SE (~125°). Slickensides on this younger fault system suggest they accommodated an E-W oriented σ_3 stress by undergoing high angle slip with a trend of 270° and a dip of roughly 75° (Fig. 2). This high angle E-W extension is typical of Mio-Pliocene deformation in the Champlain Valley.

Sample C408B appears to be a high quality single-domain isochron age suggestive of a single crystallization event (see below). In contrast, sample C408A is a complex multi-domain sample that hints at a younger age domain near 37 Ma (shown in red below). We interpret this as a secondary crystallization event, perhaps associated with the youngest fault system, but do not consider it reliable enough for interpretation.

<u>Field Observations</u>:

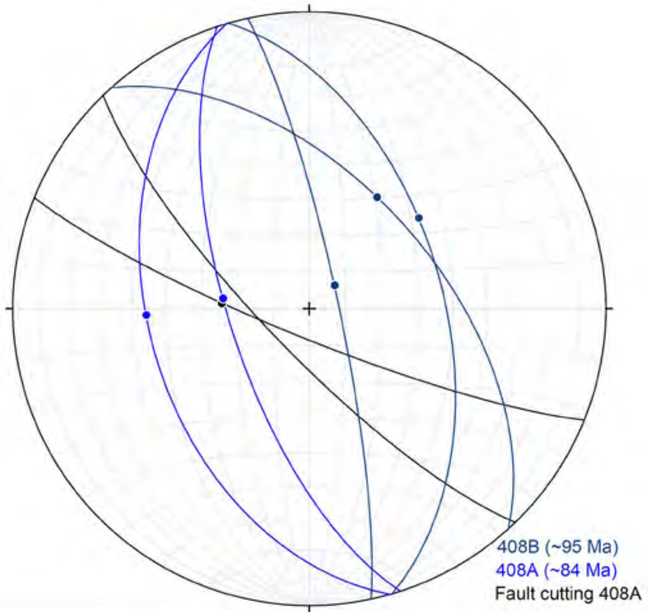

Figure 2: Stereonet plot showing measured faults at site 33

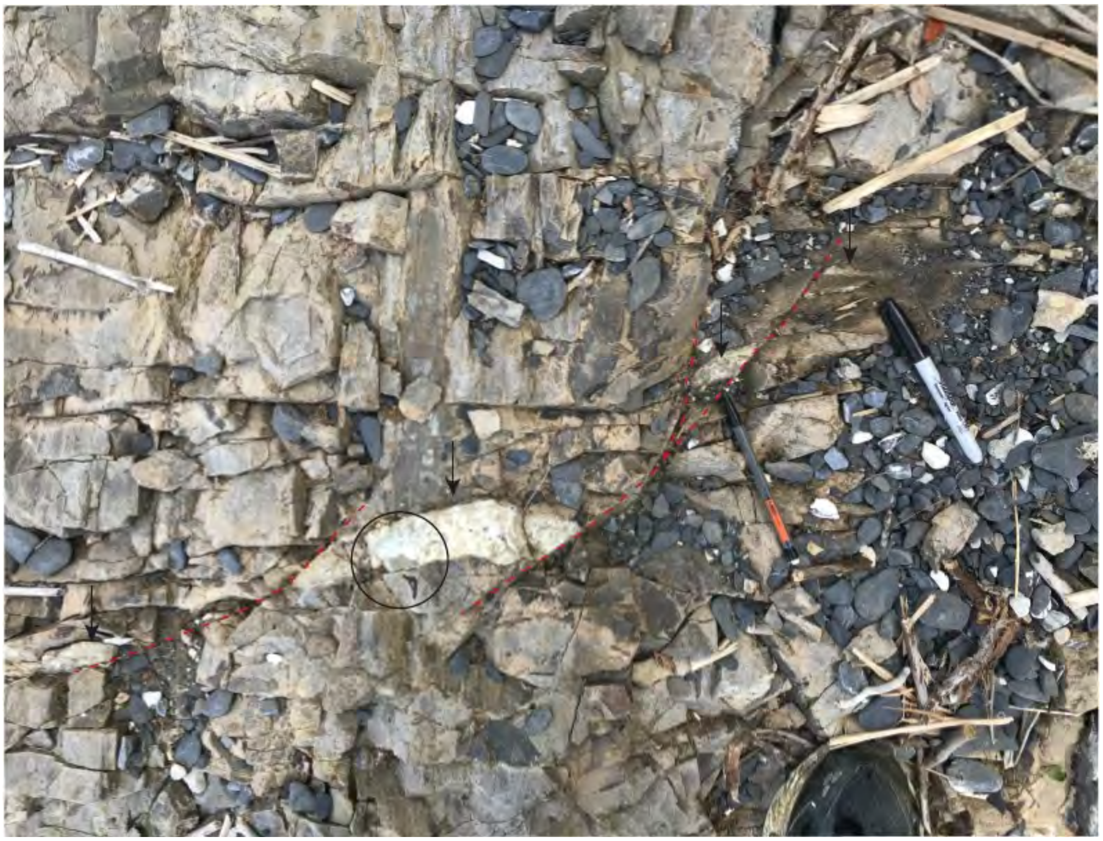

Figure 3: Vein C408A offset by a younger generation of faults shown as dashed red lines and plotted in figure 2. Thin lack arrows point to offset portions of vein C408A.

C408A:

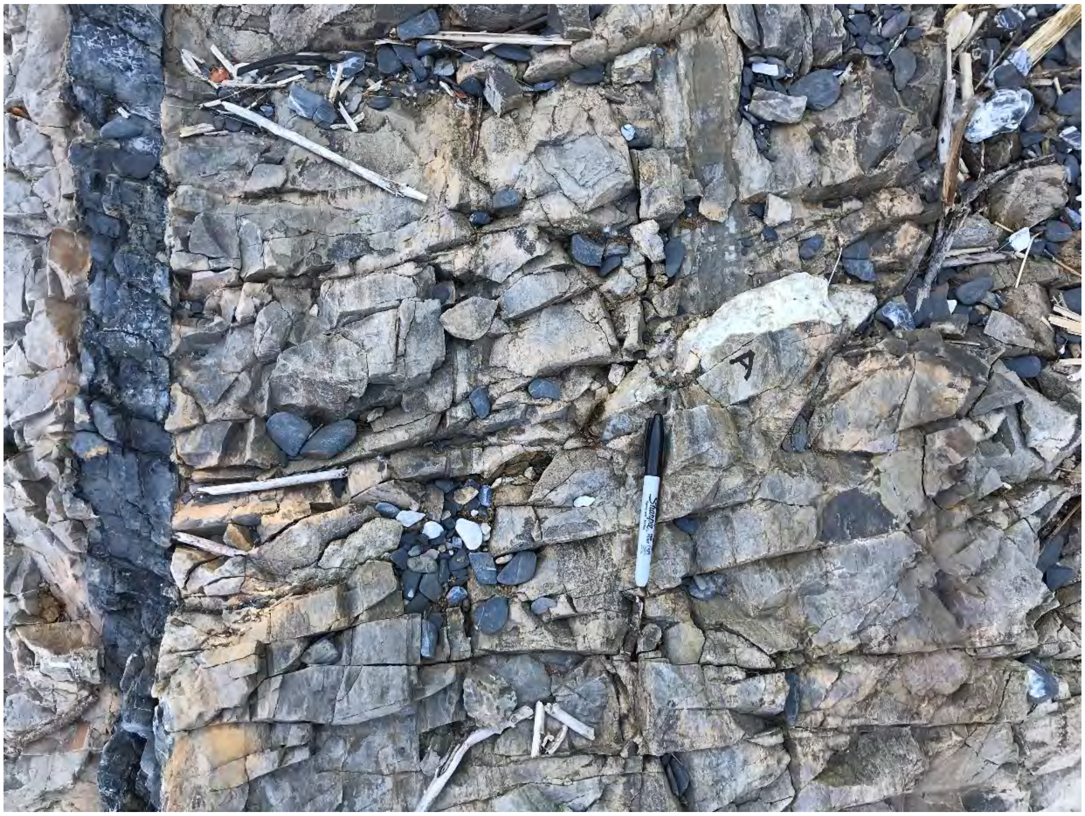

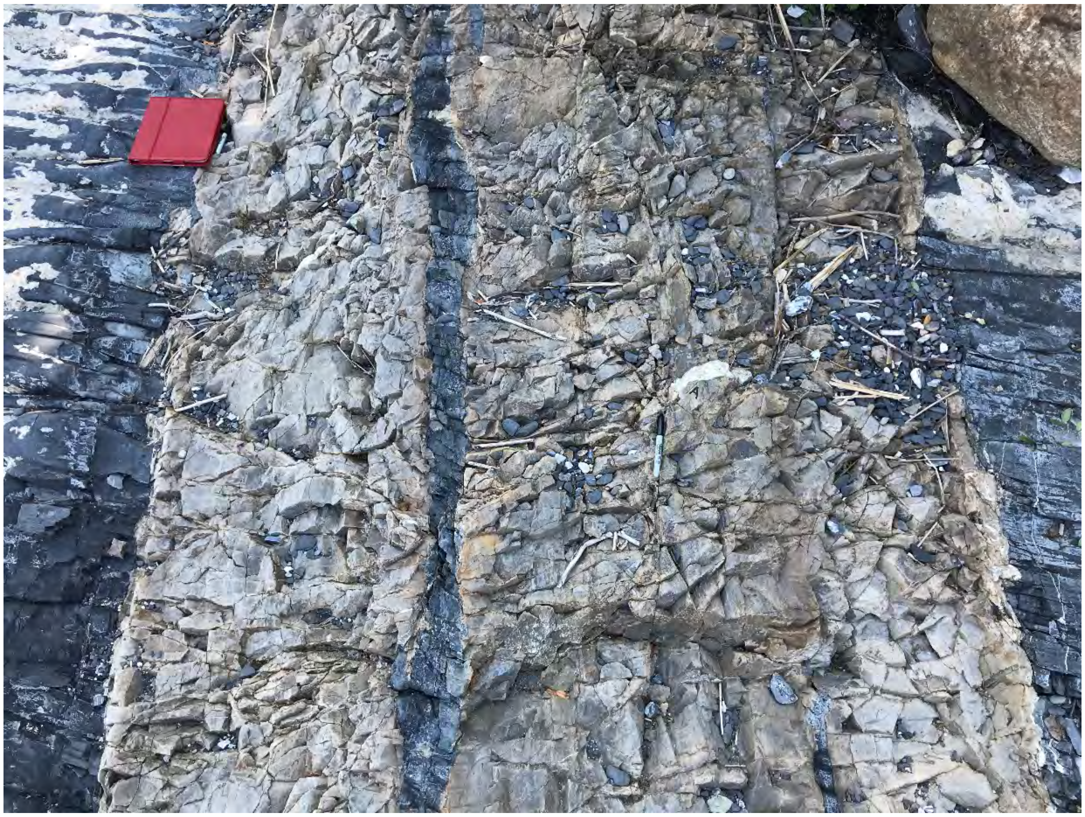

C408B:

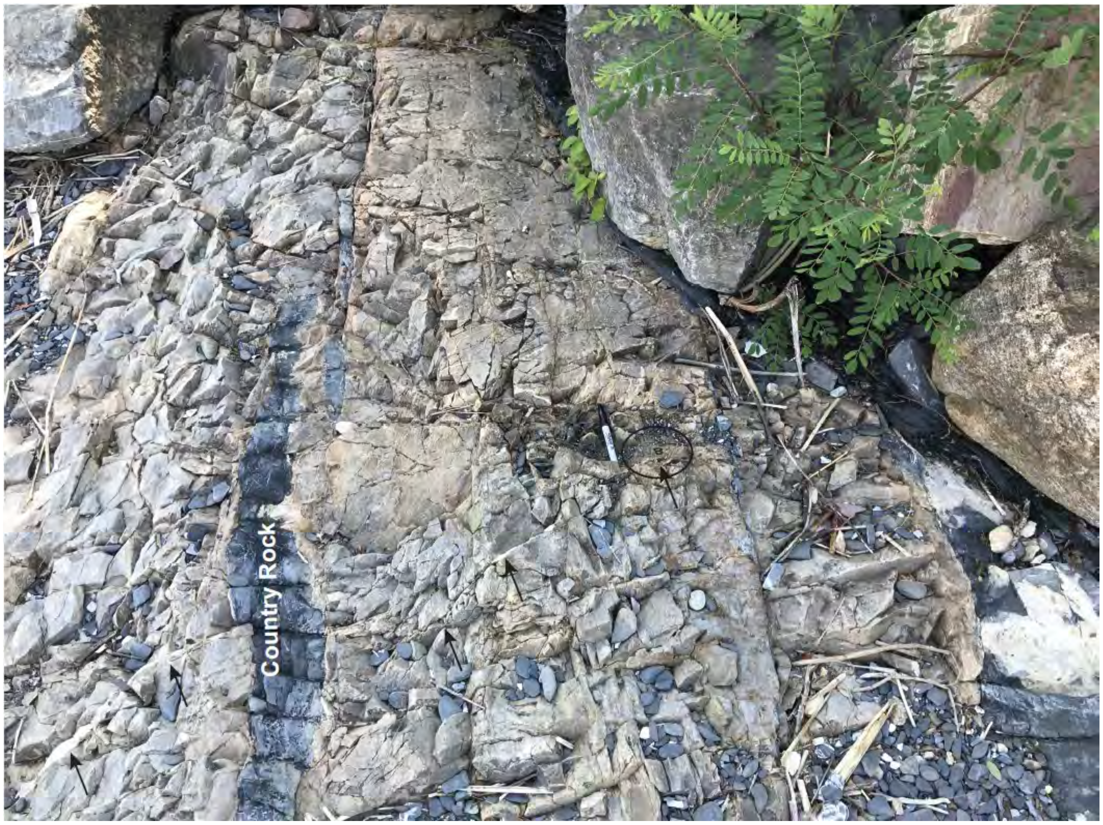

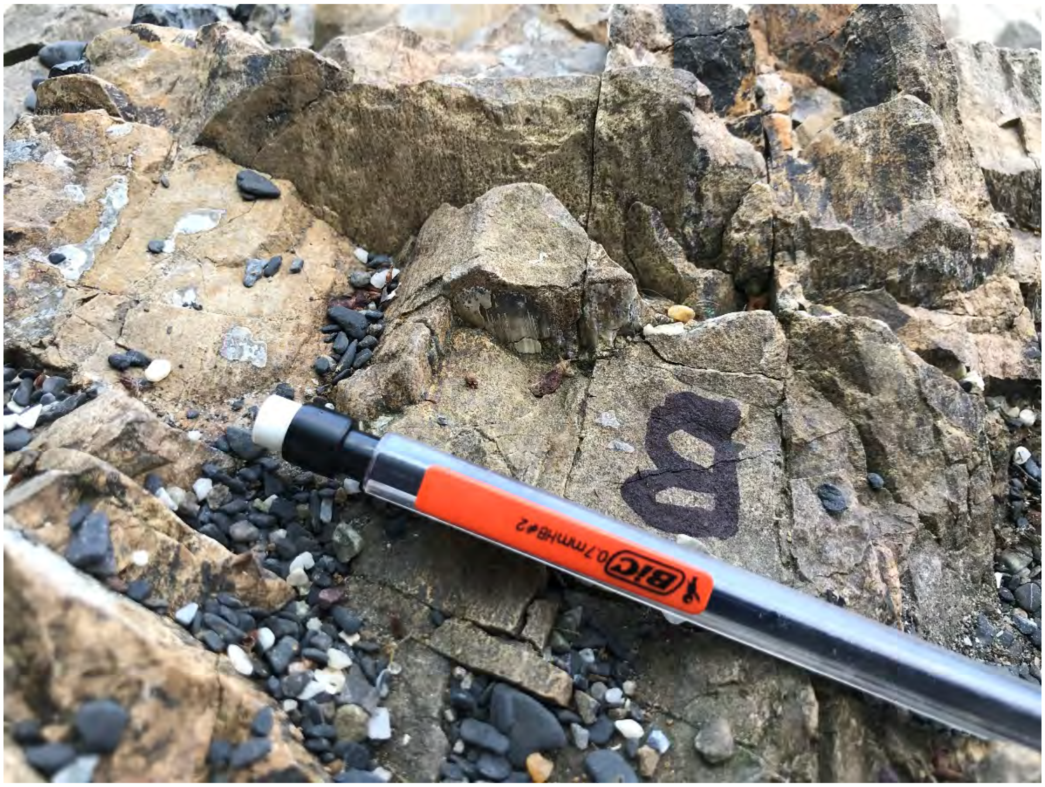

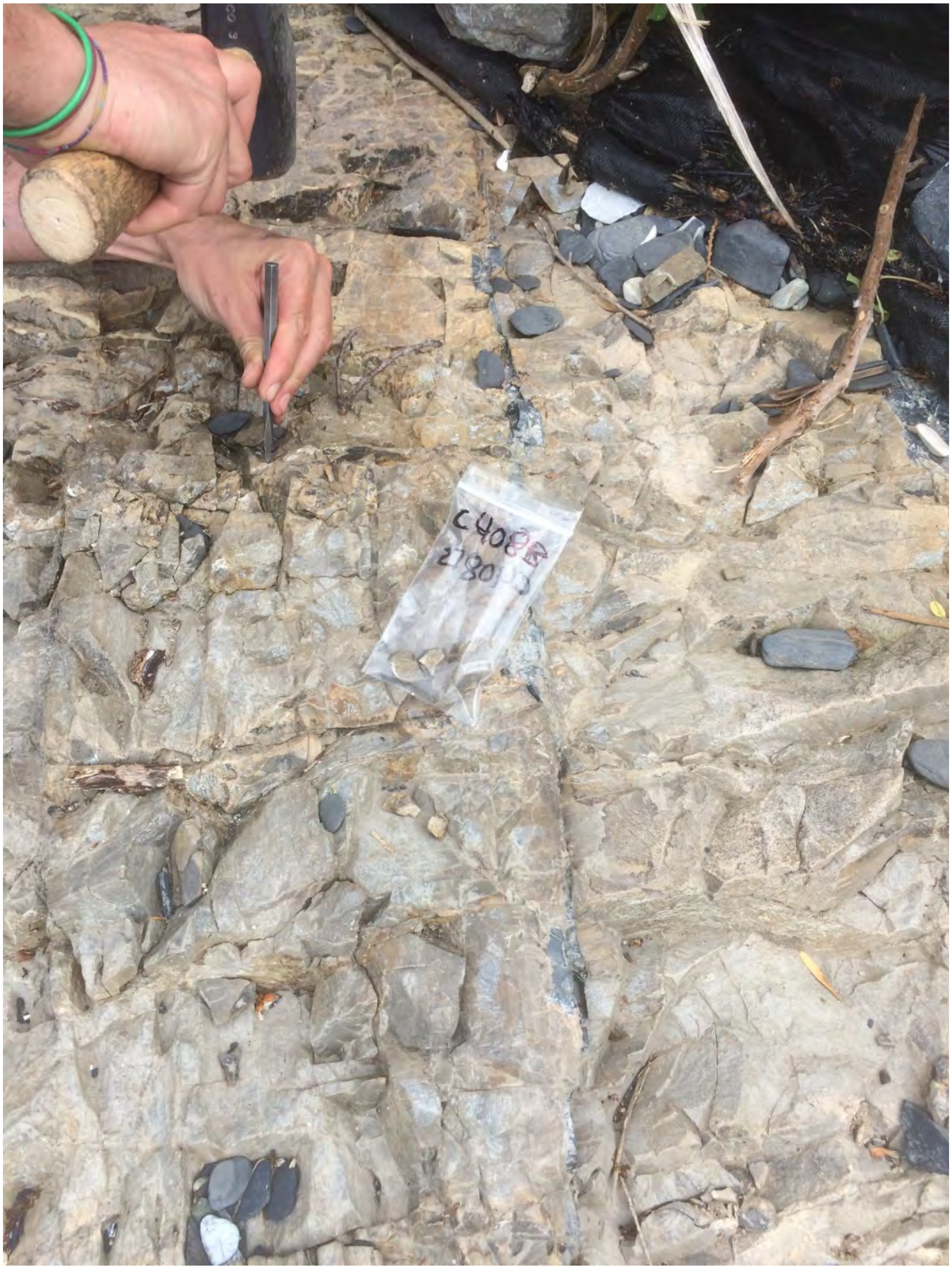
C408B

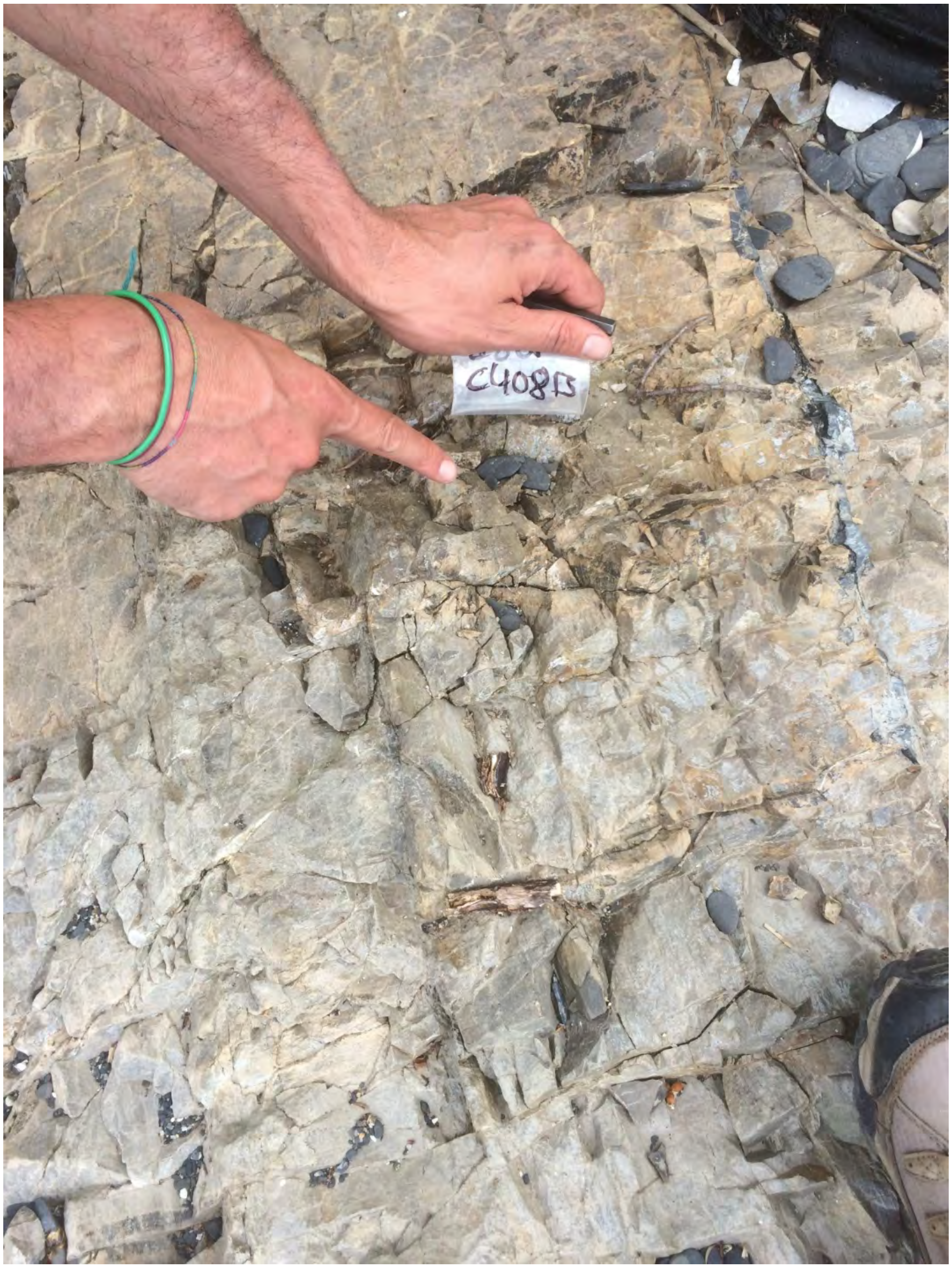

Lab Description: **C408A:**

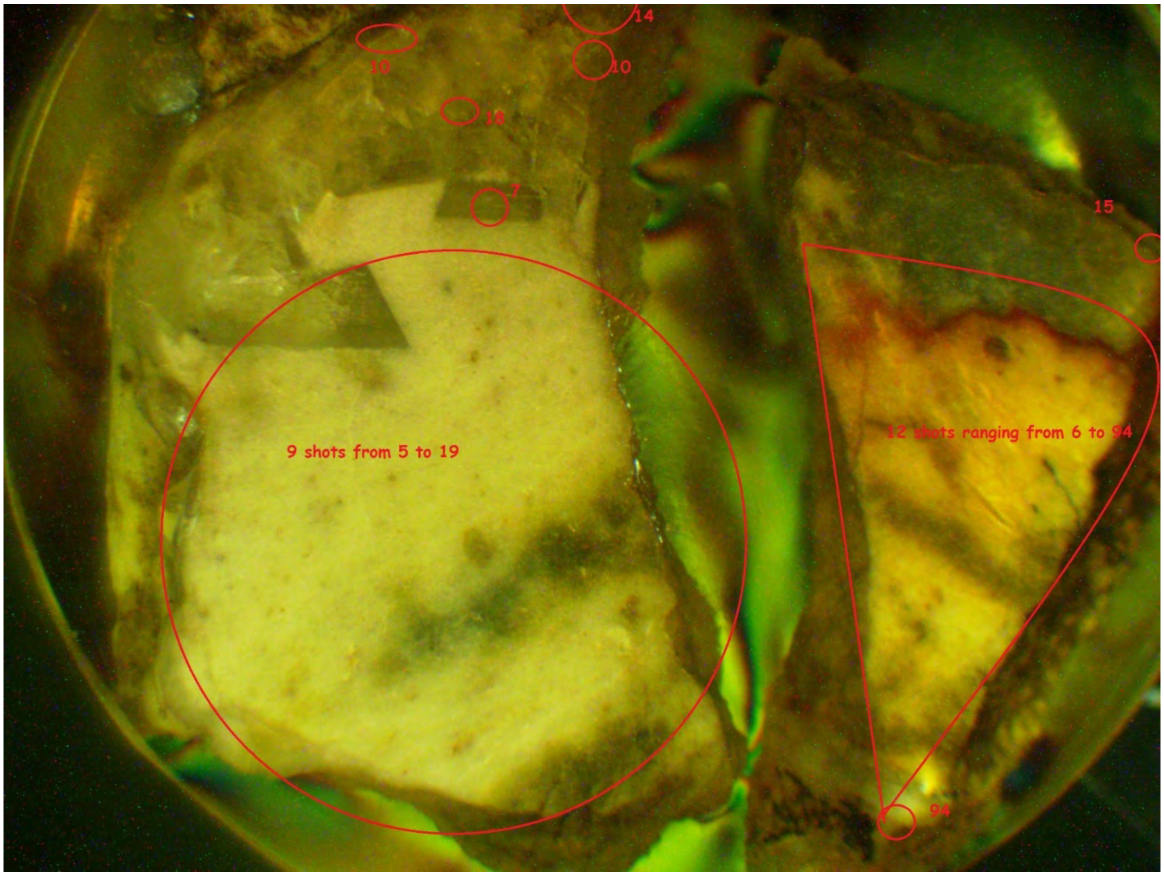

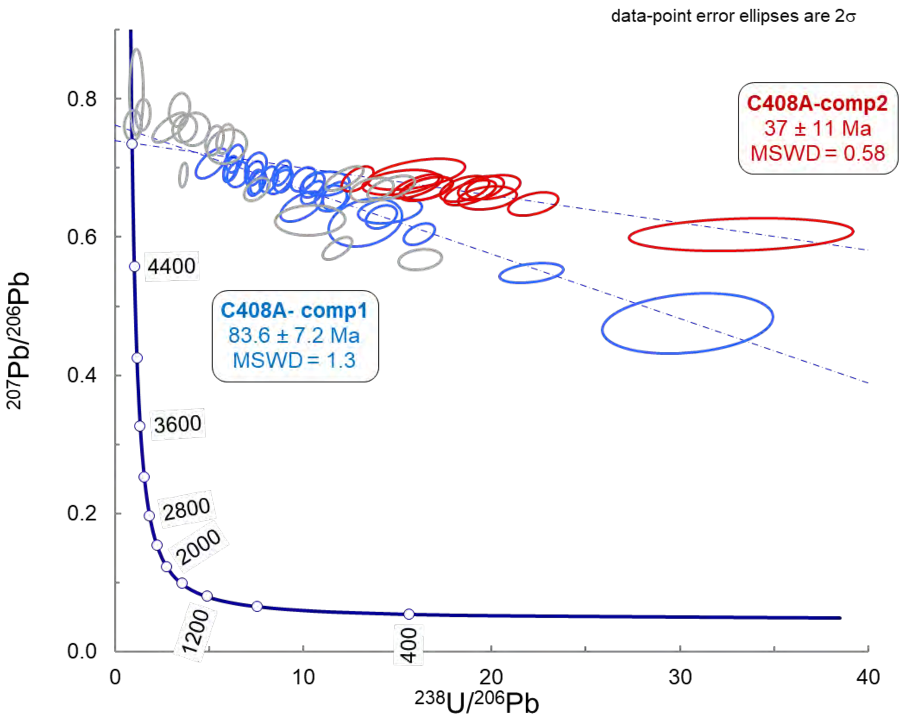

408B:

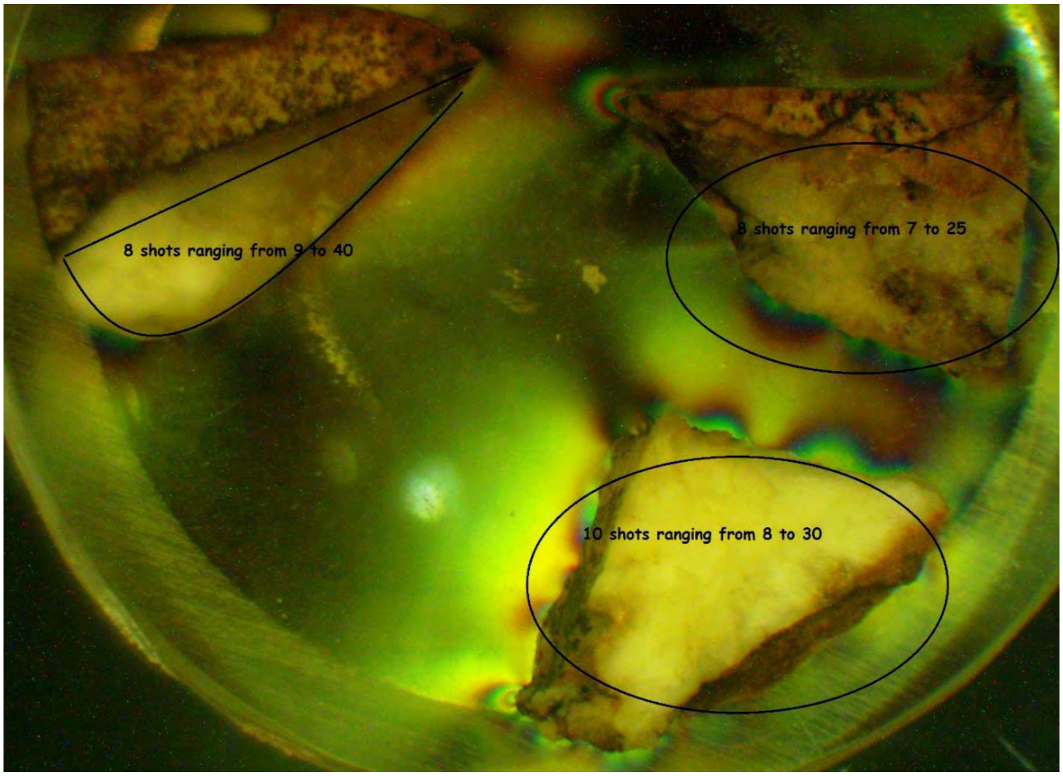
8 shots ranging from 9 to 40
8 shots ranging from 7 to 25
10 shots ranging from 8 to 30

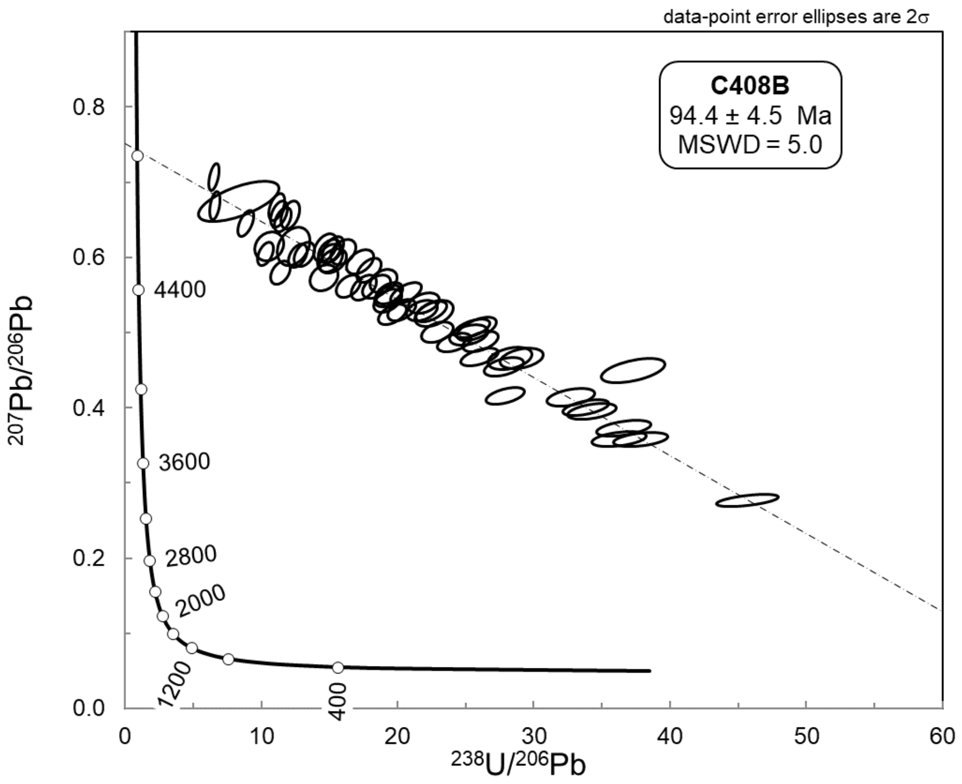
data-point error ellipses are 2σ
C408B
94.4 ± 4.5 Ma
MSWD = 5.0
$^{207}Pb/^{206}Pb$
$^{238}U/^{206}Pb$
0.0
0.2
0.4
0.6
0.8
0
10
20
30
40
50
60
4400
3600
2800
2000
1200
400
