# Supplementary material for: Pace of passive margin tectonism revealed by U-Pb dating of fracture-filling calcite
Source: Nat Commun. 2022 Apr 12;13:1953. doi: 10.1038/s41467-022-29680-z (PMC9005700; doi:10.1038/s41467-022-29680-z)

34: South Side Garden Island (44.28131991, -73.29766536)

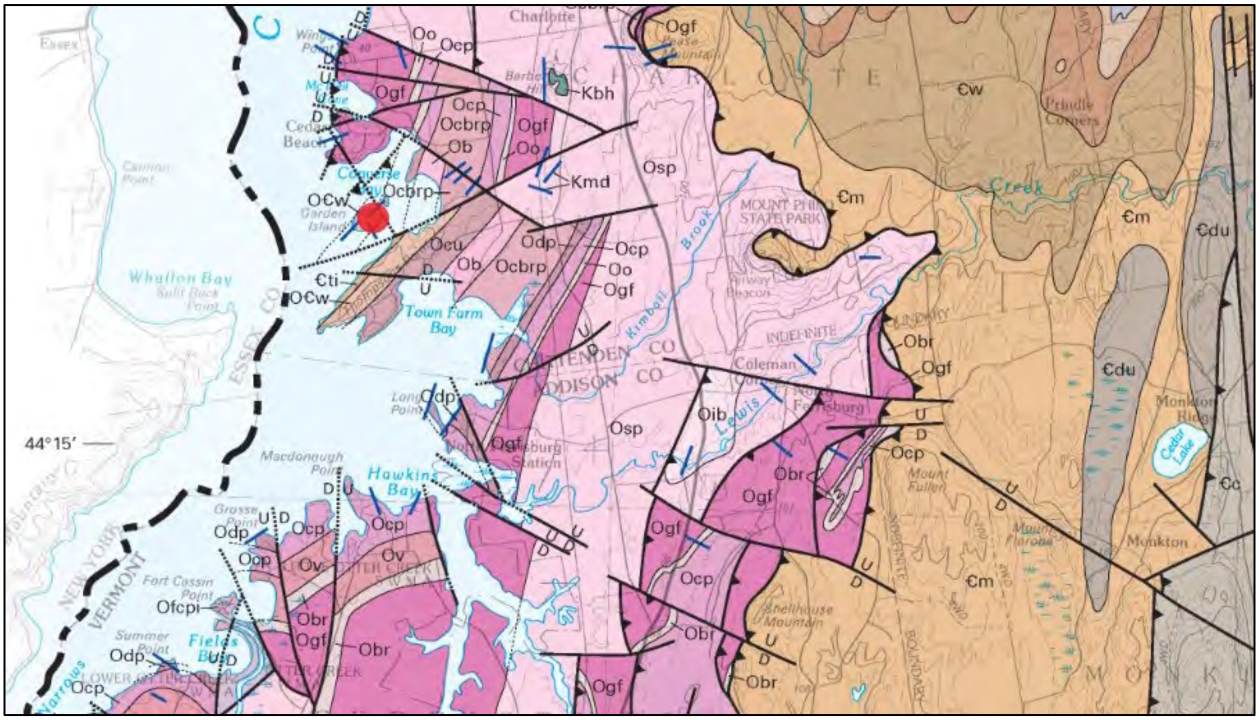

Figure 1: Sample location on state geologic map of Ratcliffe at al. (2011)

<u>Failed samples from same site:</u> none

<u>Bedrock Unit:</u> Whitehall Formation (Ocw)

<u>Ages</u>: C414A: 85.3 ± 1.7 Ma; **C414B:** 718 ± 23 & 111.8 ± 9.6 & 16 ± 11 Ma;

<u>Summary</u>: This site is located on the southern side of Garden Island and is only accessible by boat, most easily from the boating access at Converse Bay in Charlotte, VT. It consists of two samples collected roughly 100m east of a large volcanic sill at site D72. The older sample (C414B) is collected from a large sub-vertical breccia zone striking ~120°. The breccia matrix is composed of 2-4 cm sparry calcite crystals and contains clasts of a dark carbonaceous rock. It yields three age components: a ~16 Ma age suggesting a secondary crystallization event, a ~112 Ma age interpreted as the primary vein-forming crystallization event, and a ~718 Ma age from accidentally dating clasts of the dark carbonaceous country rock within the vein. These clasts are apparently a Cryogenian-aged carbonate rock older than the Champlain Valley Sequence and previously undescribed in the Champlain Valley. Because C414B is planar and lacks slip indicators we interpret it as a mode 1 structure that opened under high fluid pressure and records a NE-SW σ3 stress. However, this structure may represent a brecciated intrusion closer in style to magmatic intrusion than a dilational vein. Further study is required.

Sample C414A is from a ~ 1 cm wide vein with a strike of 320° emplaced at ca. 85 Ma. The discrete planar nature and lack of slickensides suggests this is a mode 1 fracture, that opened under a NE-SW σ3 stress. It records a high quality, single-domain U-Pb isochron age suggestive of a single crystallization event.

Field Observations

C414A:

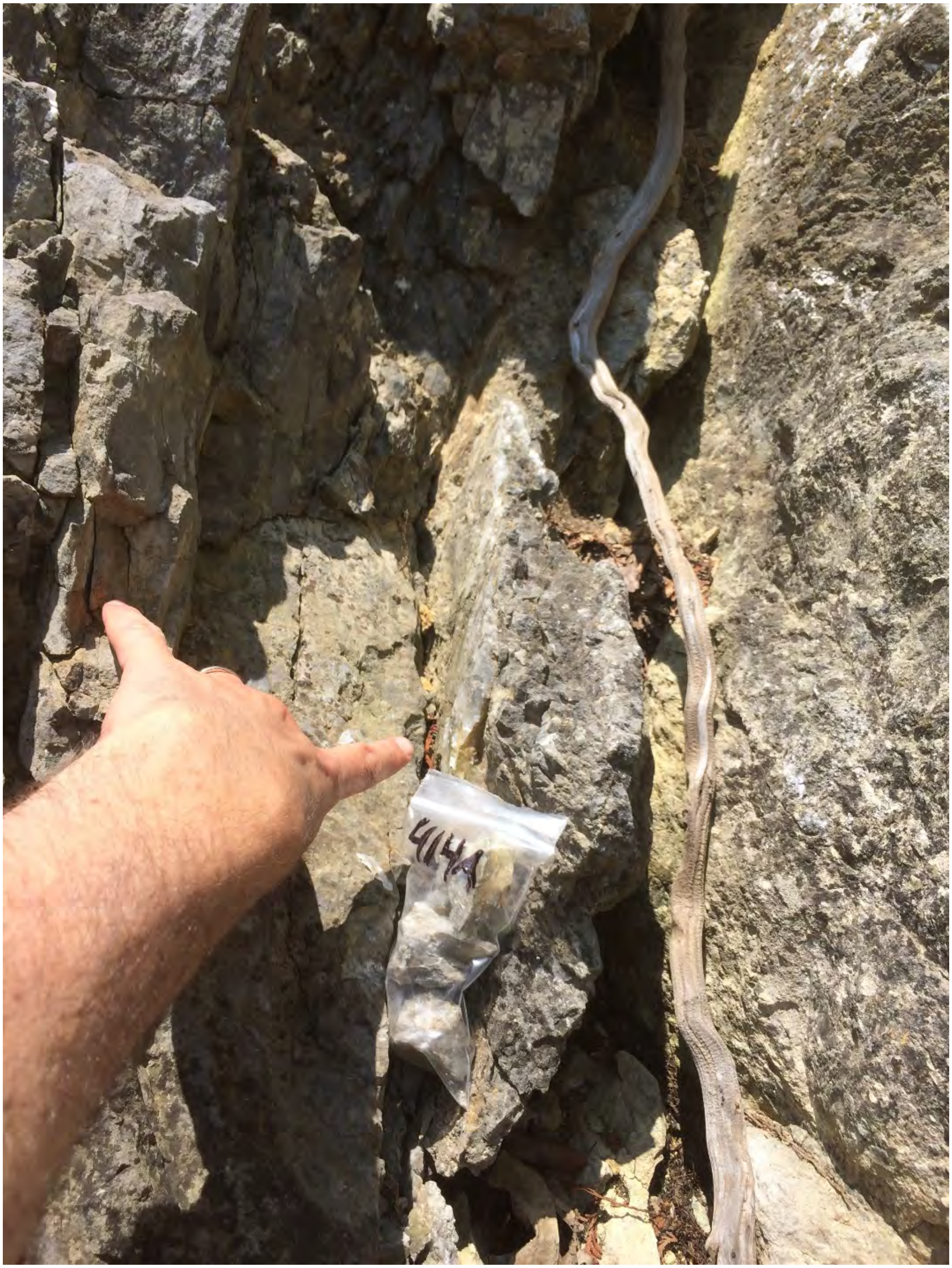

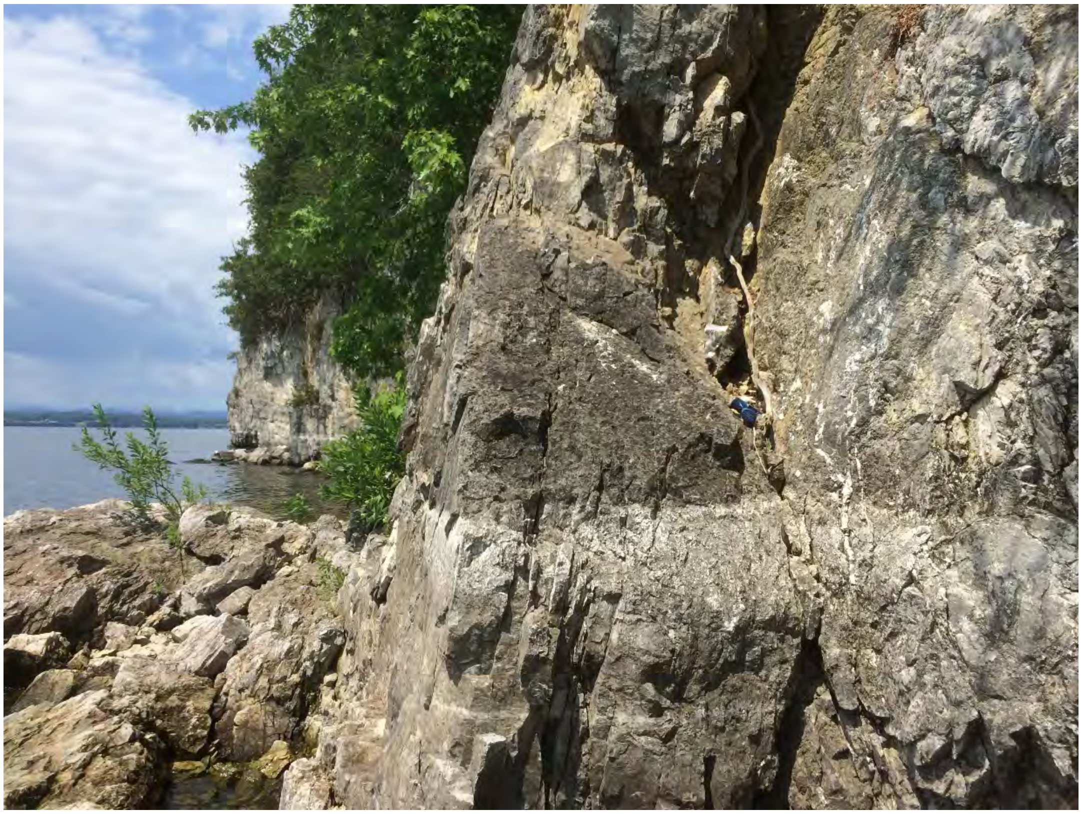

C414B:

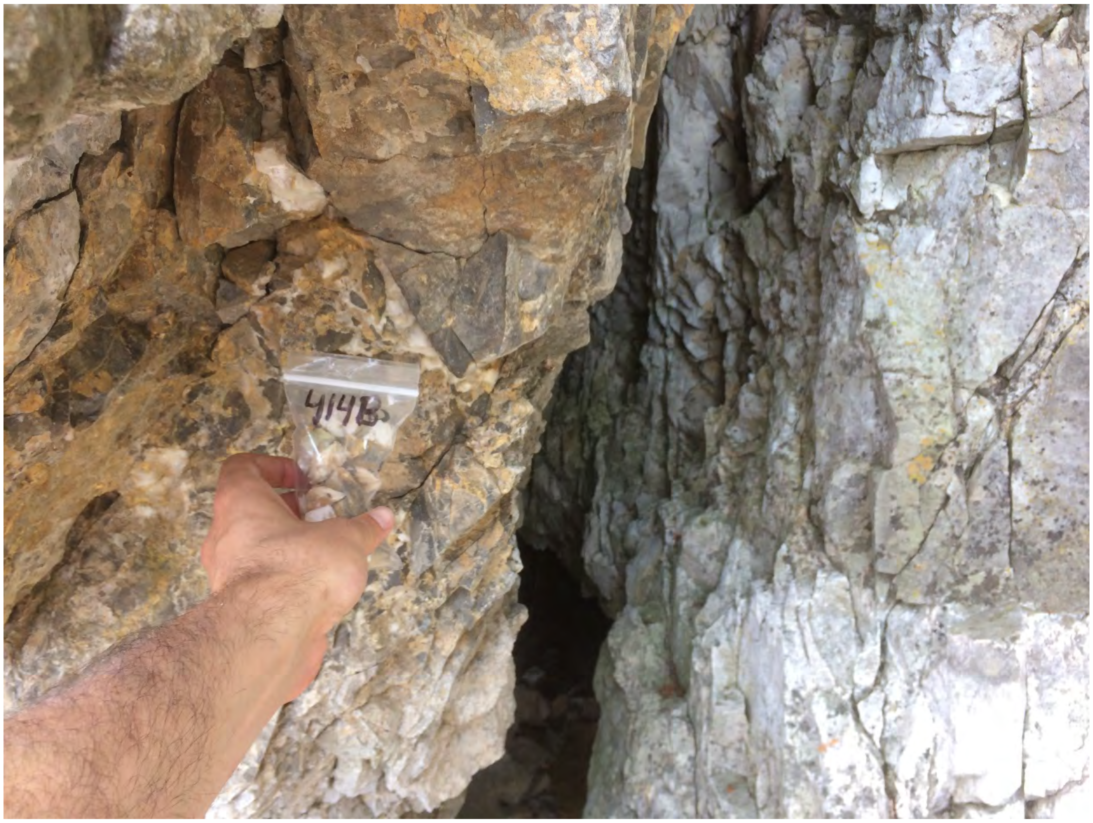

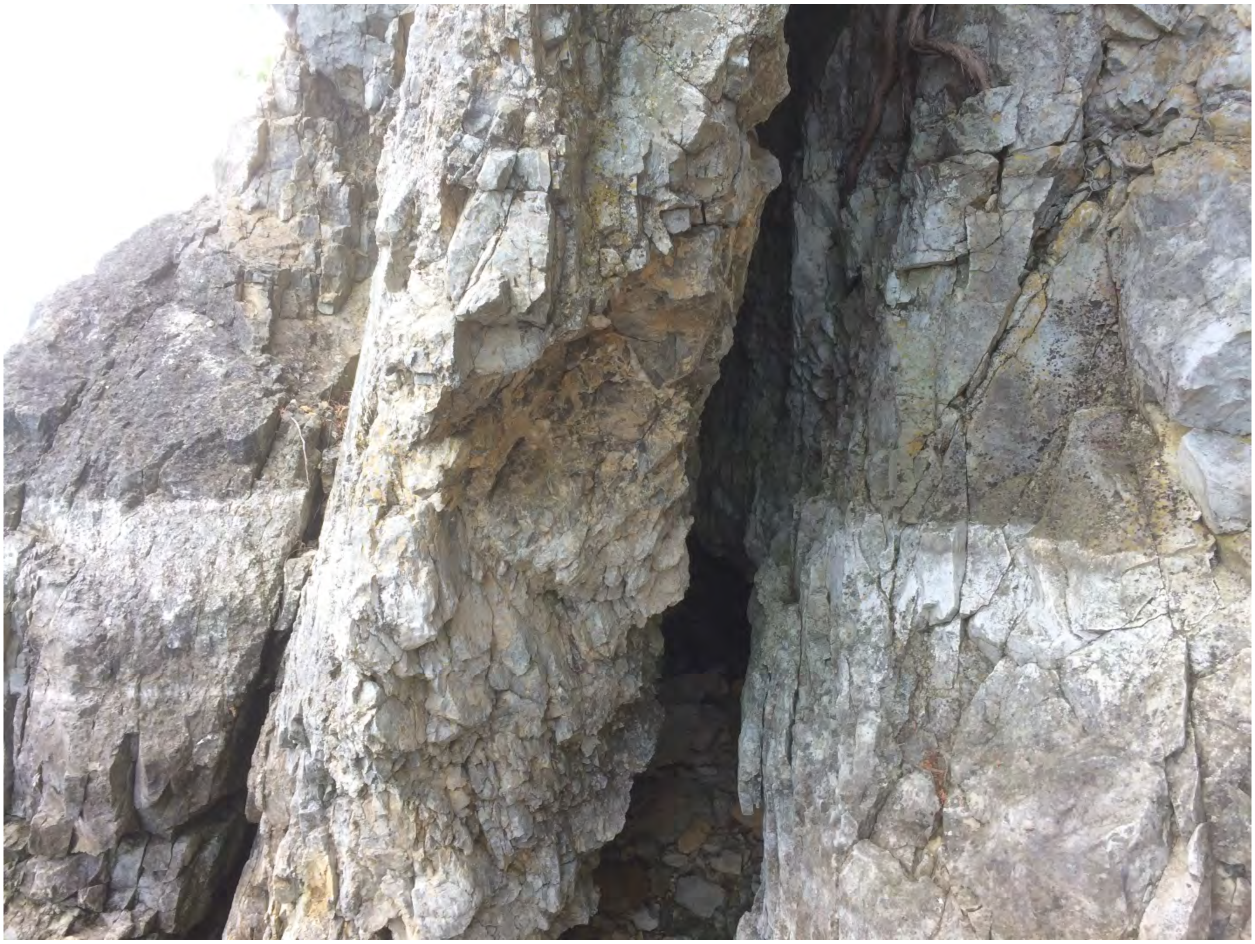

Lab Observations:

C414A: circles represent areas of interest, not laser shots.

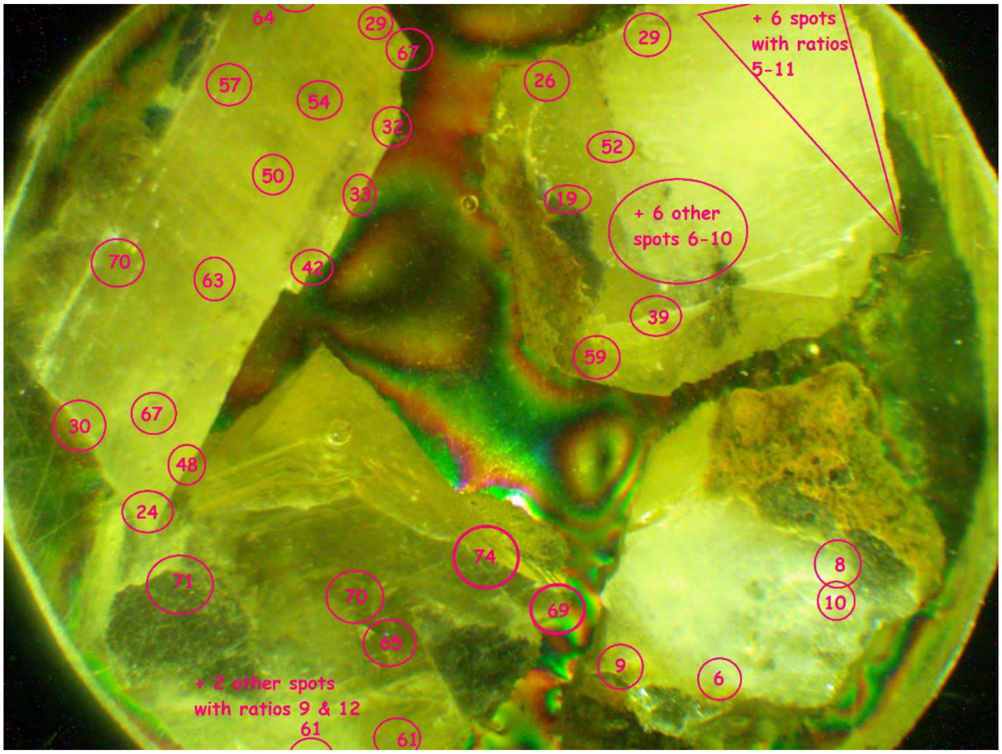

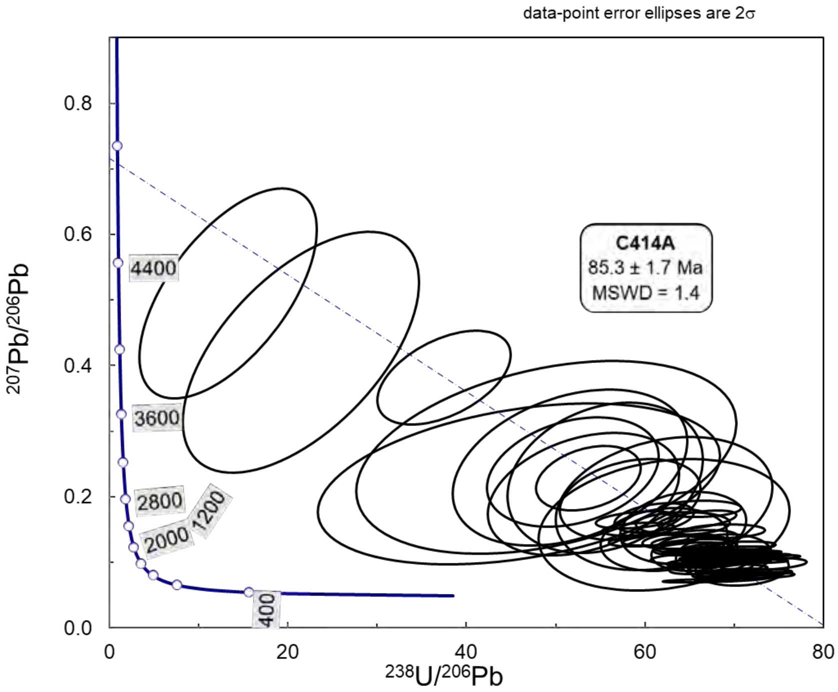

C414B: circles represent areas of interest, not laser shots.

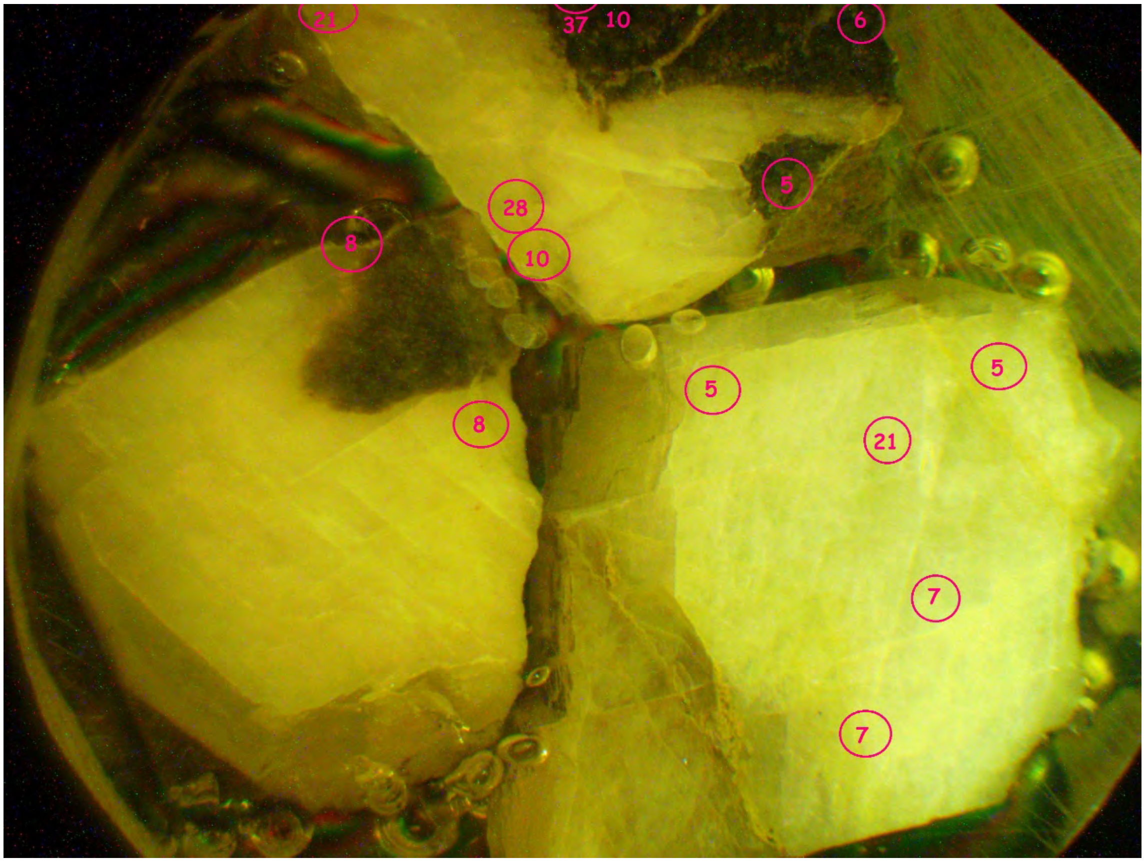

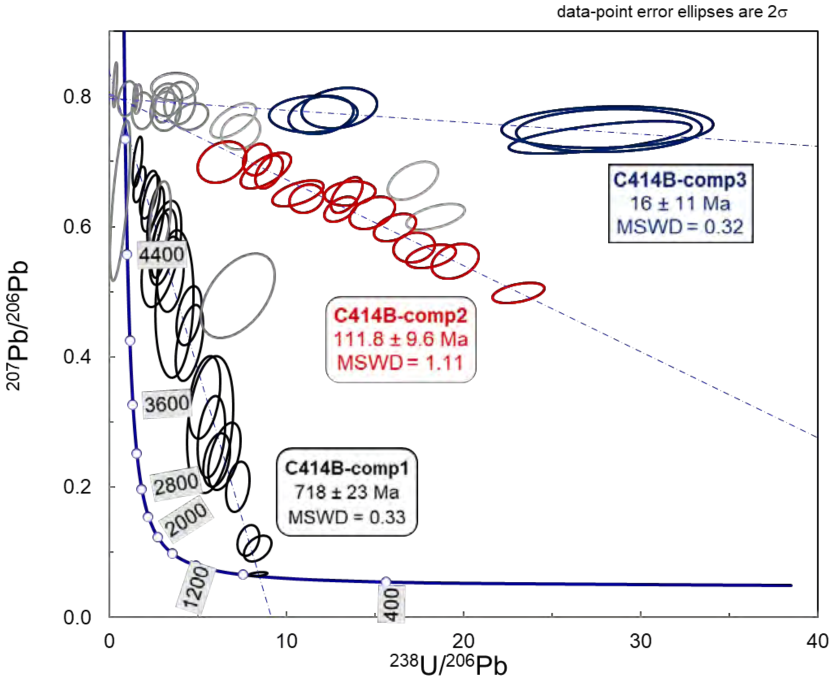

Supplement: Supplementary file 6 — Source Data [file 41467_2022_29680_MOESM6_ESM.zip › 2.zip!/appendix2b_site_summaries_21to50/34_garden_island_south_final.pdf]
