# Supplementary material for: Pace of passive margin tectonism revealed by U-Pb dating of fracture-filling calcite
Source: Nat Commun. 2022 Apr 12;13:1953. doi: 10.1038/s41467-022-29680-z (PMC9005700; doi:10.1038/s41467-022-29680-z)
Supplement: Supplementary file 6 — Source Data [file 41467_2022_29680_MOESM6_ESM.zip › 2.zip!/appendix2b_site_summaries_21to50/35_providence_island_west_final.pdf]

# 35: Providence Island West (44.615321, -73.352111)

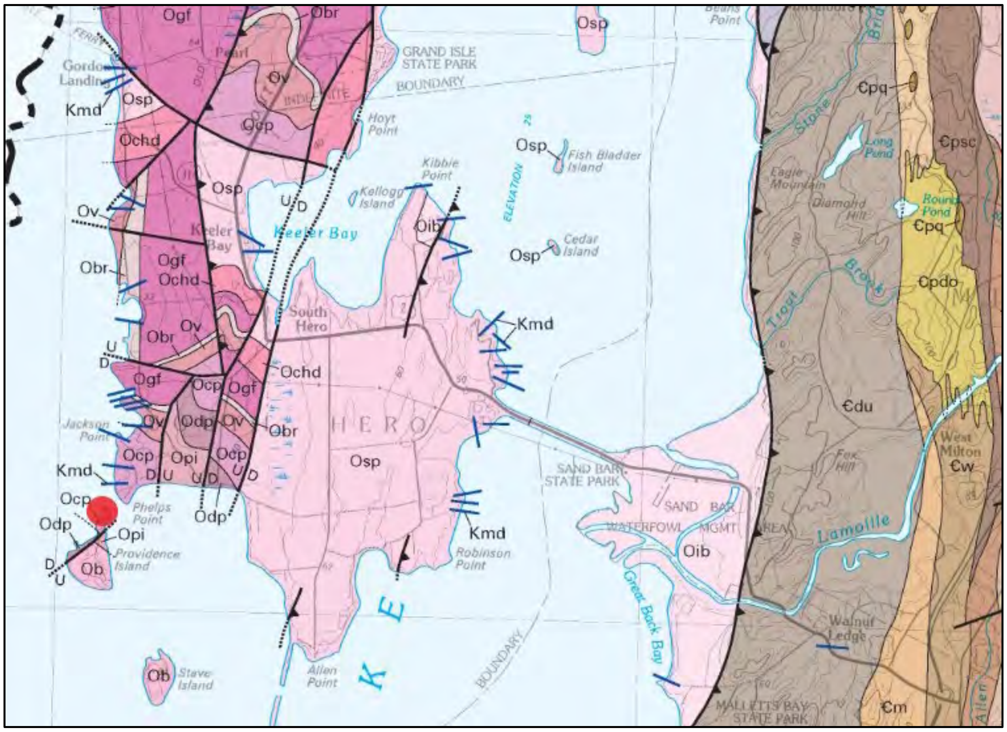


**Failed samples from same site:** C418A, B, C

**Bedrock Unit:** Crown Point Formation (Ocp)

**Ages**: **C418D**: 68.3 ± 7.2 Ma; **C418E**: 60.7 ± 2.1 Ma

**Summary**: This complex site sits in a small cove on the western side of Providence Island and can only be accessed by boat, most easily from a boat launch on the southern tip of South Hero. This site is characterized by NW striking bedding with evidence for bedding-parallel slip. In particular, a bedding-parallel fault zone is exposed along the north end of the cove with an indeterminable sense of slip (Fig. 4). Sample C418D records an event at ca. ~68 Ma along this fault zone, but the site precludes a stress interpretation. The southern end of the cove is characterized by a top to the north extensional fault zone which has several slip planes at various orientations (Fig. 3). A drag fold formed along this fault as nearly orthogonal beds were dragged to higher angle orientations during slip fault (see photos and stereoenet below). Sample C418E records an event on this fault zone at ca. 61 Ma. It comes from a discrete accessory fault/fracture that terminates upward into the extensional fault zone and was most likely formed during top-to-the-northwest slip on said fault. However, no slip indicators were observed on the C418E structure. Between these two fault zones numerous bedding planes show evidence for bedding-plane slip with stepped calcite tiling and slickenfibres. Sample C418C is from one of these bedding-parallel surfaces and records undated strike-slip motion with a trend/plunge of 144°/13°. These three fault surfaces have worked together to accommodate various stress regimes over time, and may be linked to a major NW-SE trending normal fault shown on the geologic map. Dated samples suggest at least two episodes of tectonism at this site.

**Field Observations**:

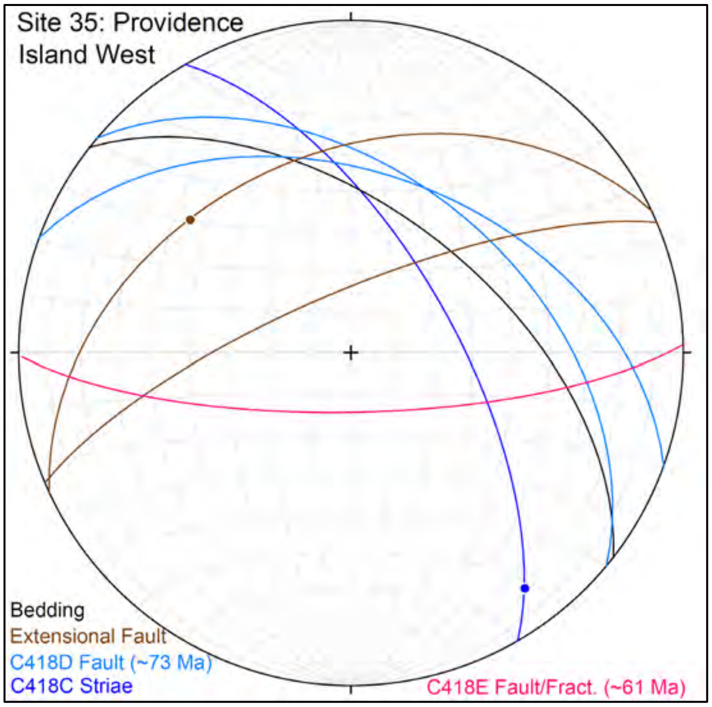


**Figure 2:** Stereonet plot showing orientations of structures at site 35

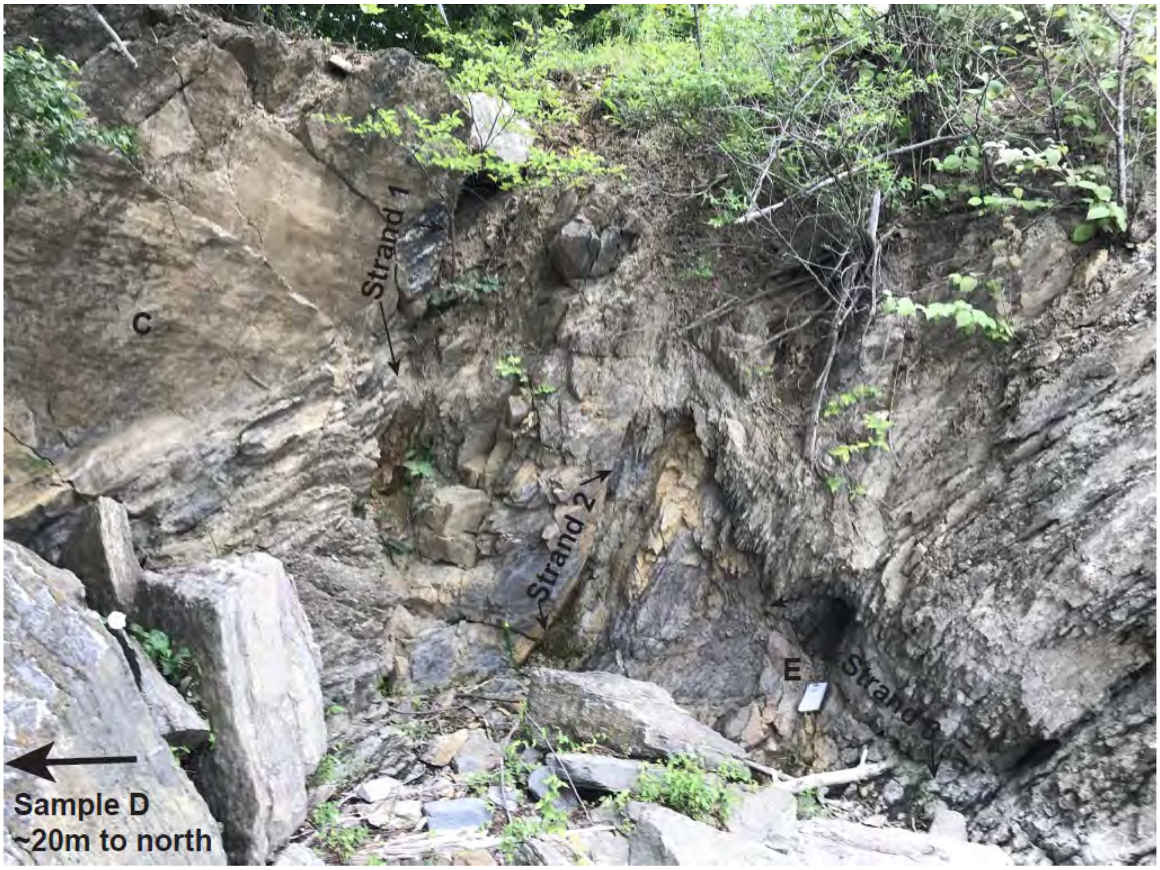


**Figure 3:** Overview of the high angle extensional fault zone at southern end of cove.

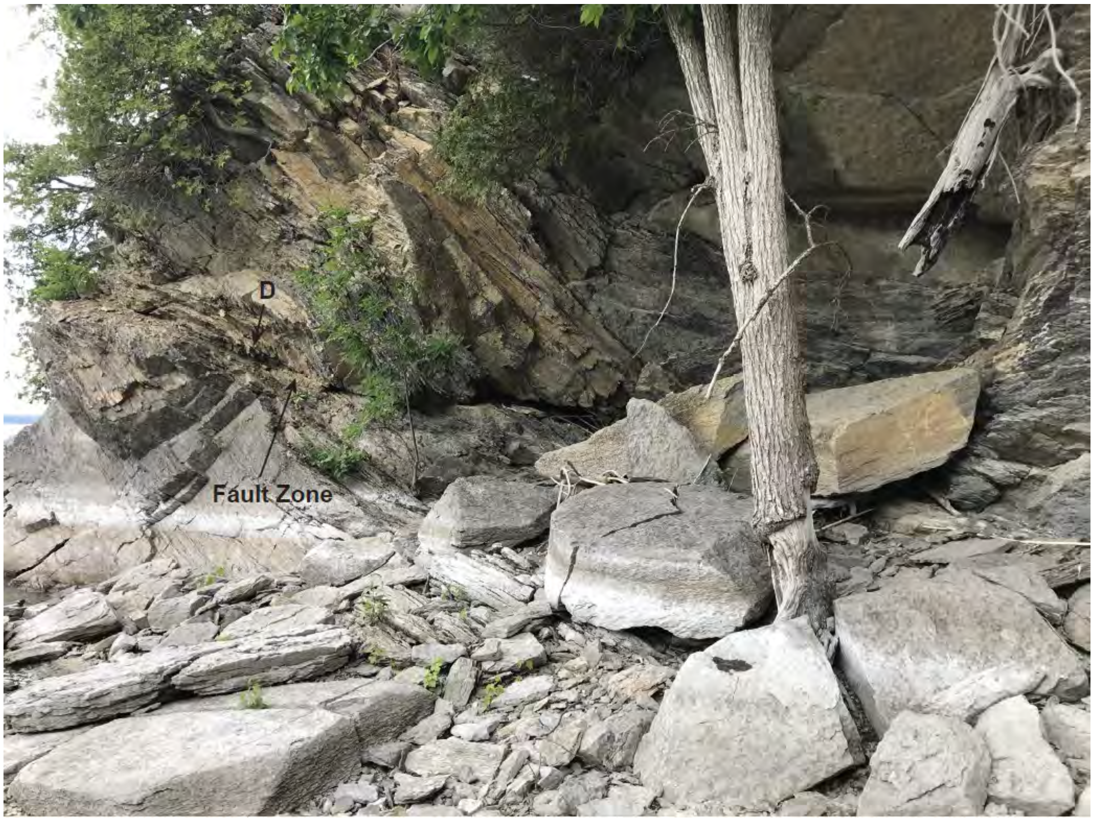

**Figure 4:** Overview of the bedding parallel thrust fault at northern end of cove

**C418D:**

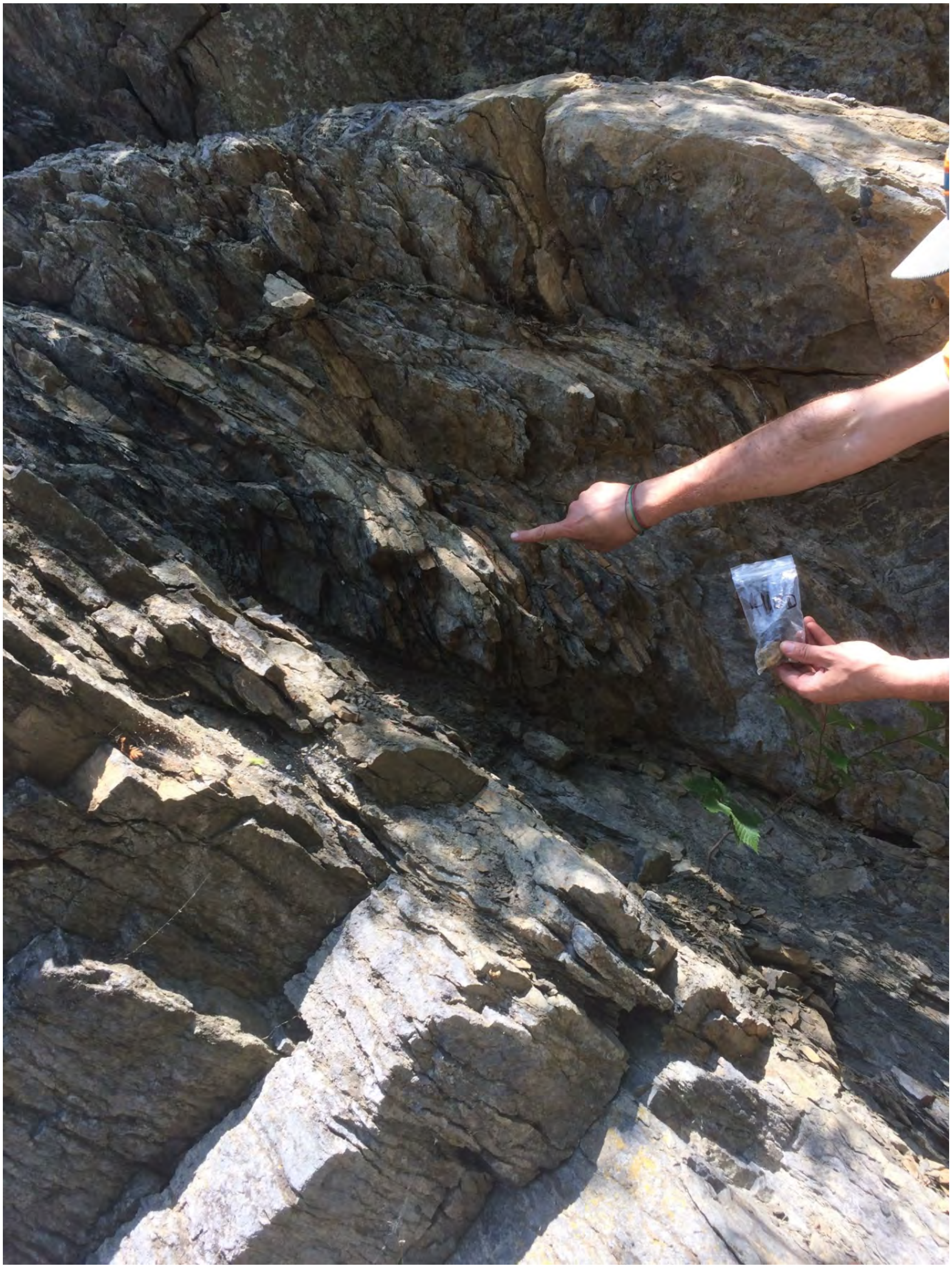

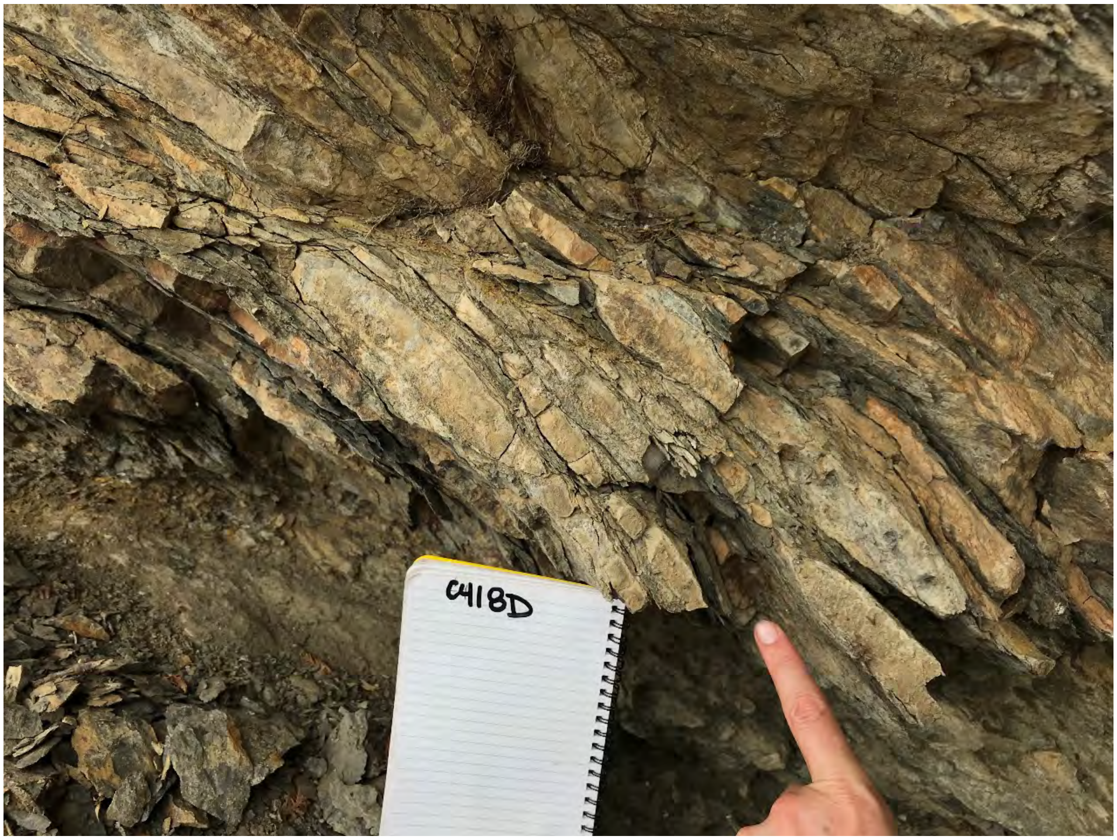
C418D

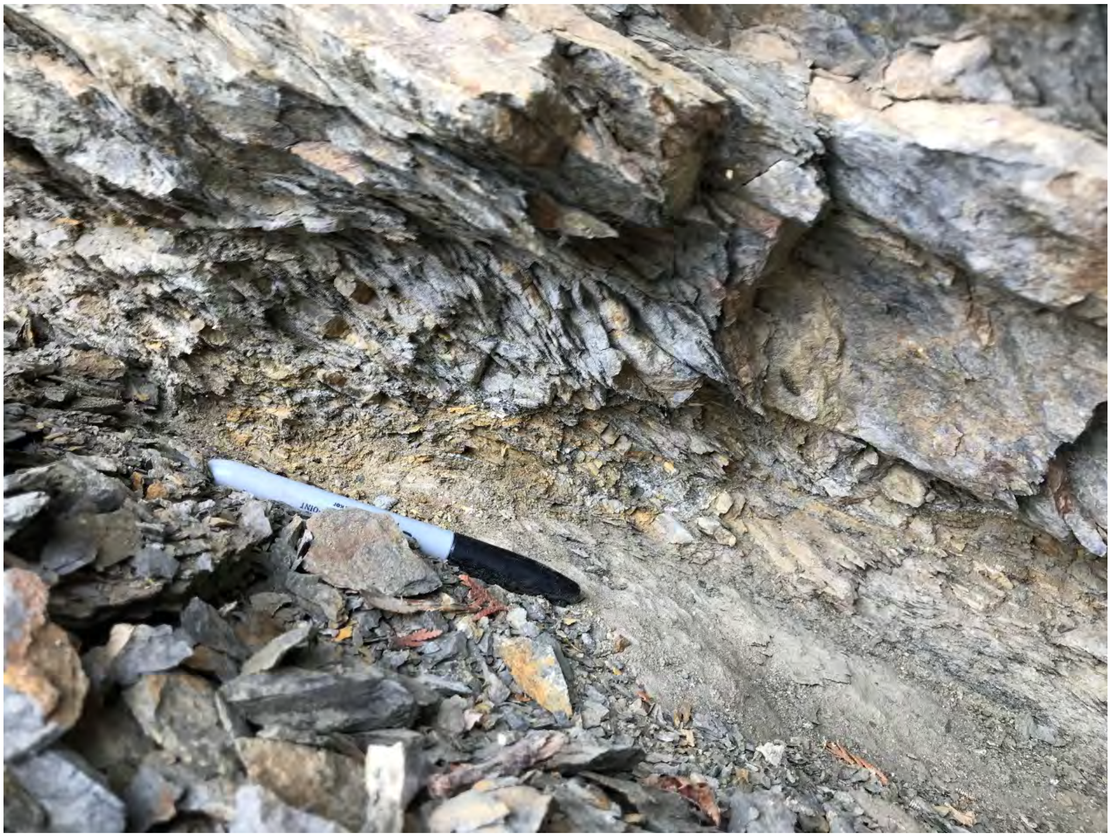

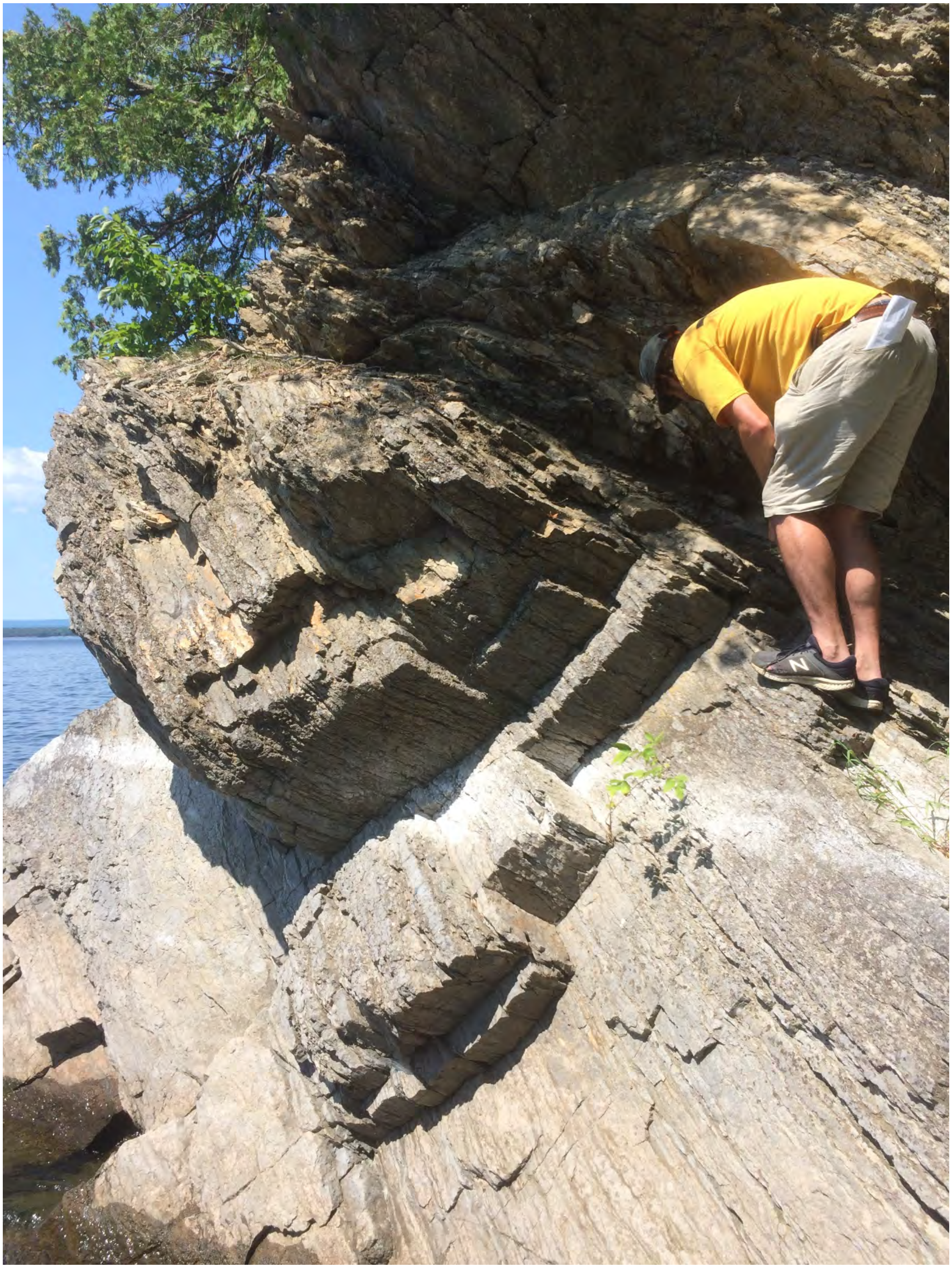

**C418E:**

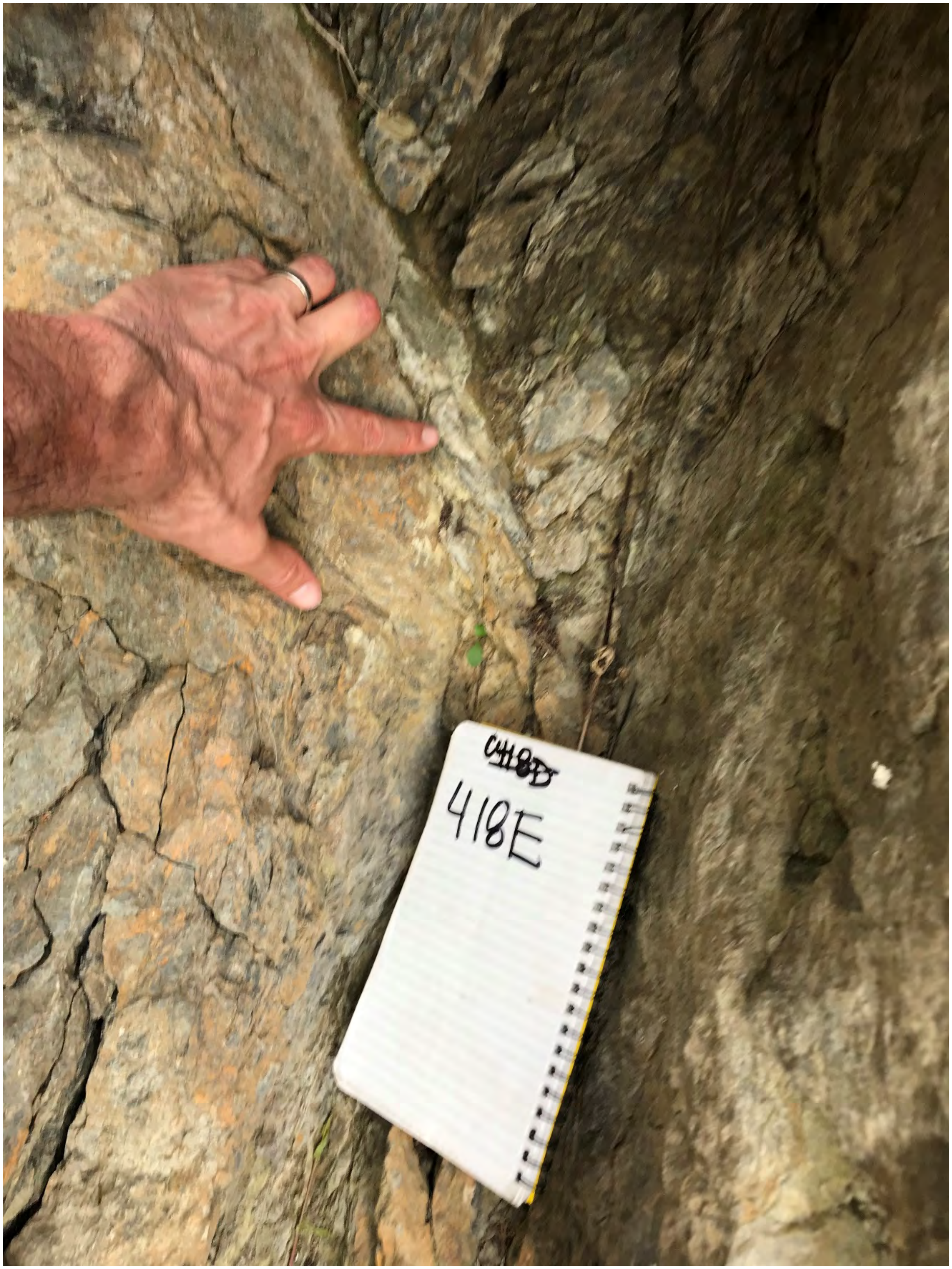

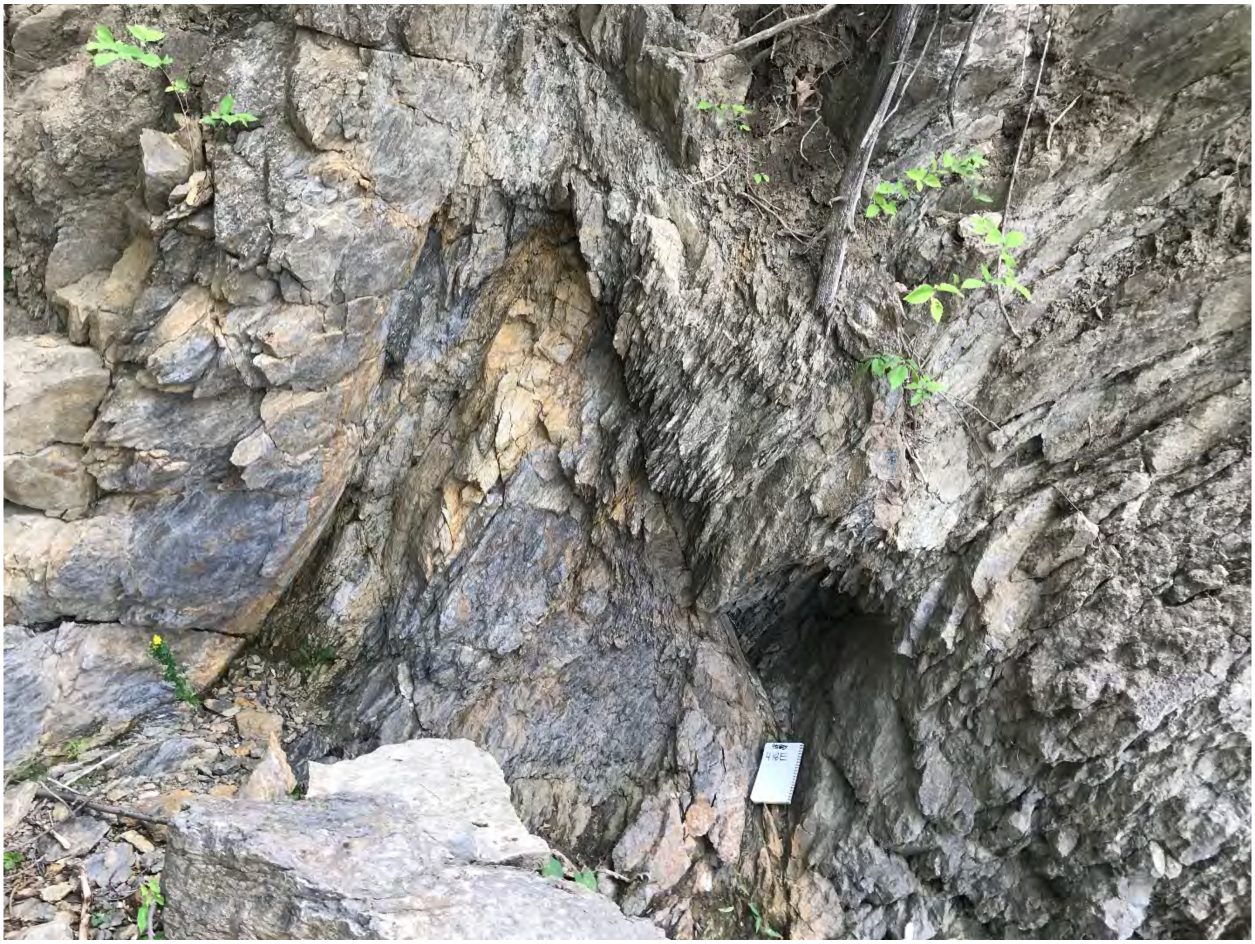

**Lab Observations**:

**C418D:**

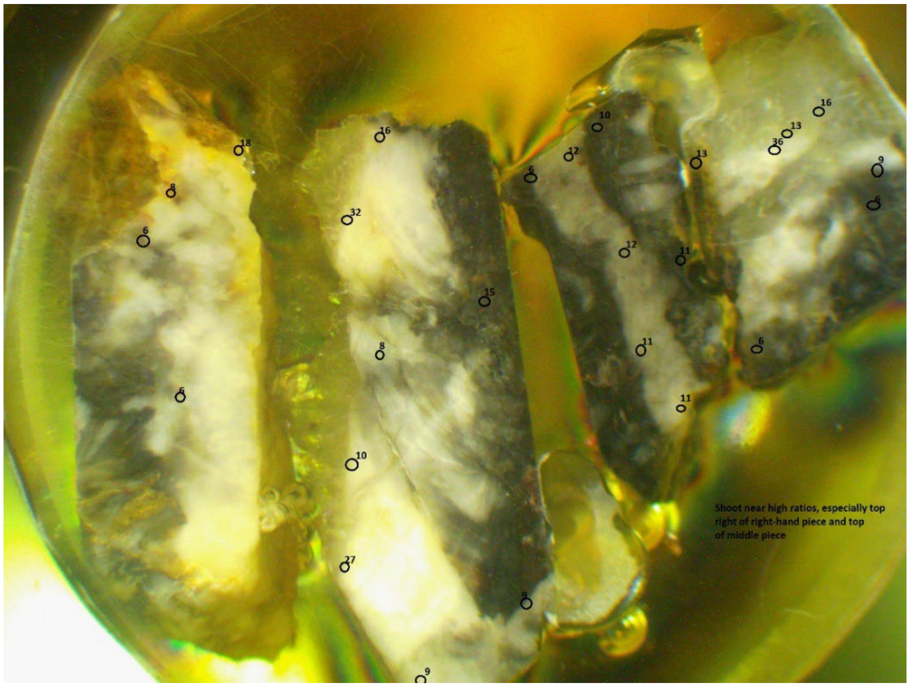


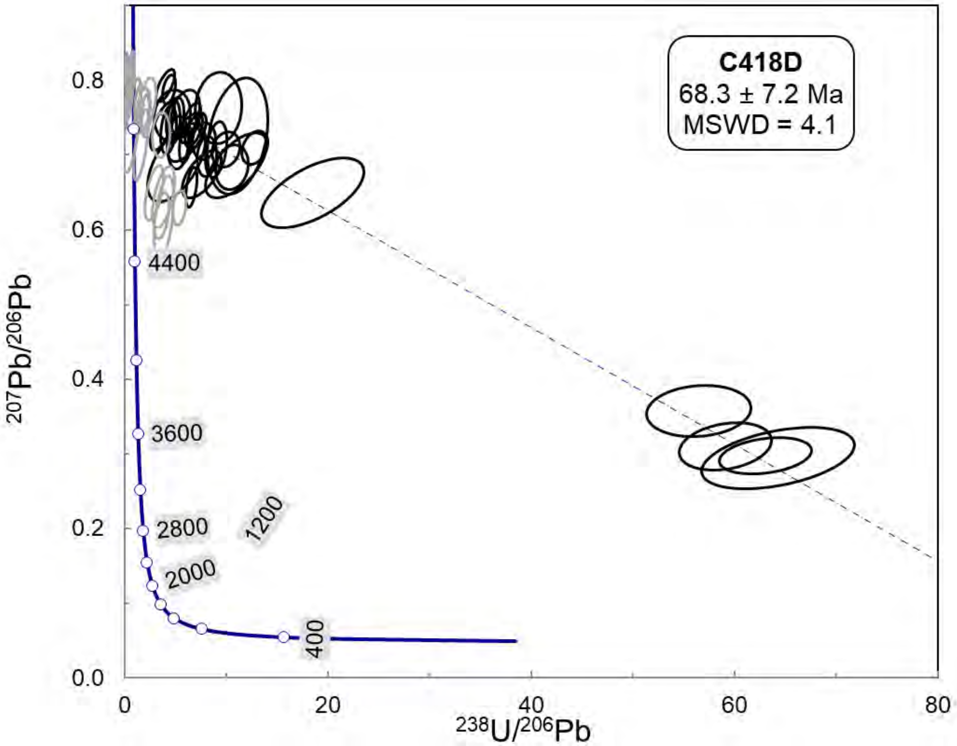

**C418E:**

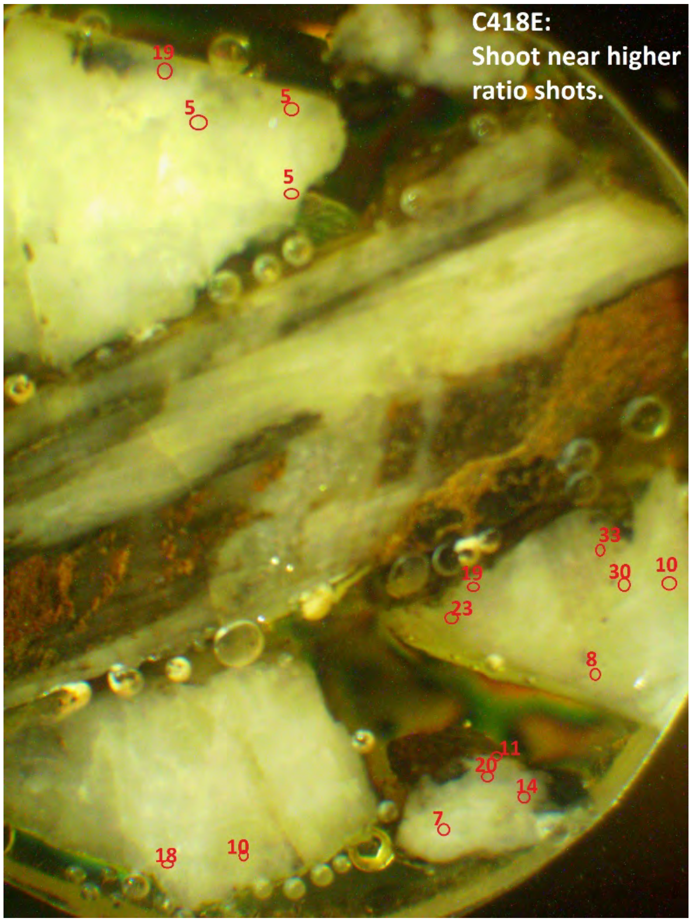

C418E:
Shoot near higher ratio shots.
19
5
5
5
33
19
30
10
23
8
11
20
14
7
18
10


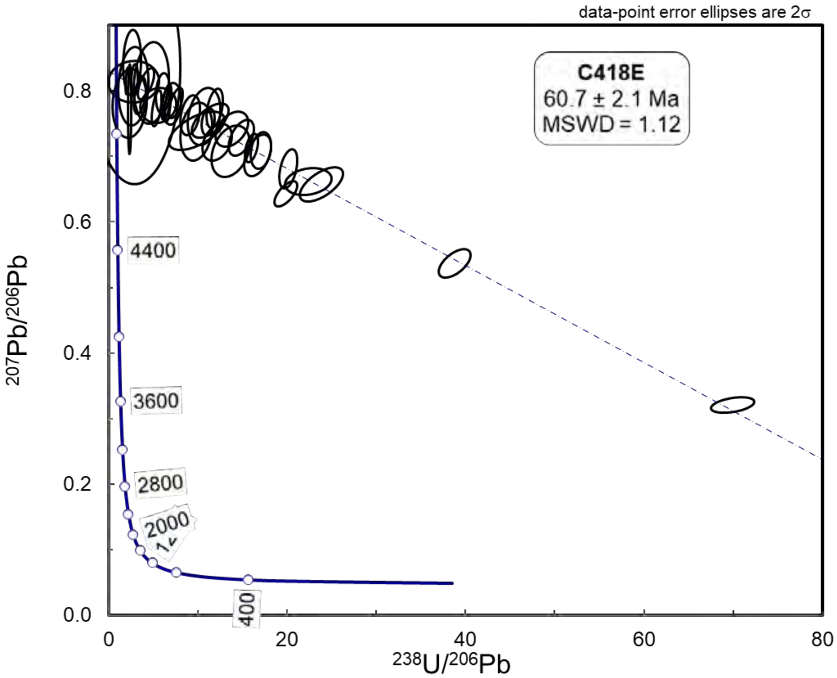

data-point error ellipses are 2σ
C418E
60.7 ± 2.1 Ma
MSWD = 1.12
4400
3600
2800
2000
400
0.8
0.6
0.4
0.2
0.0
0
20
40
60
80
207Pb/206Pb
238U/206Pb
