# Supplementary material for: Pace of passive margin tectonism revealed by U-Pb dating of fracture-filling calcite
Source: Nat Commun. 2022 Apr 12;13:1953. doi: 10.1038/s41467-022-29680-z (PMC9005700; doi:10.1038/s41467-022-29680-z)

36: Rte. 22, LaChute River, NY (43.843077, -73.406699)

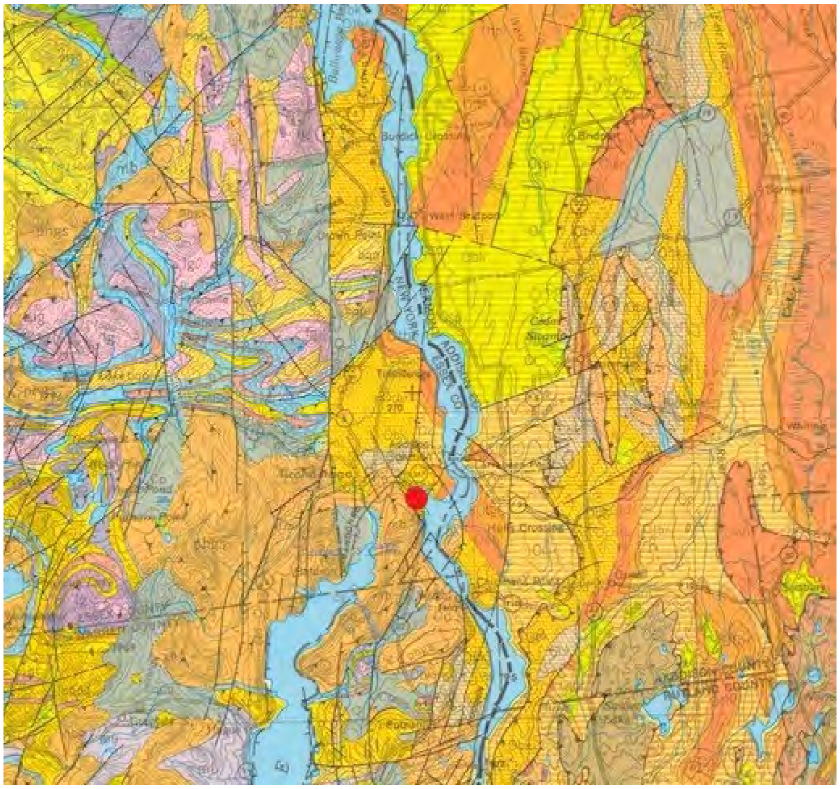

Figure 1: Sample location ADK bedrock sheet by Isachsen and Fisher, 1970. NW trending fault is incorrectly located.

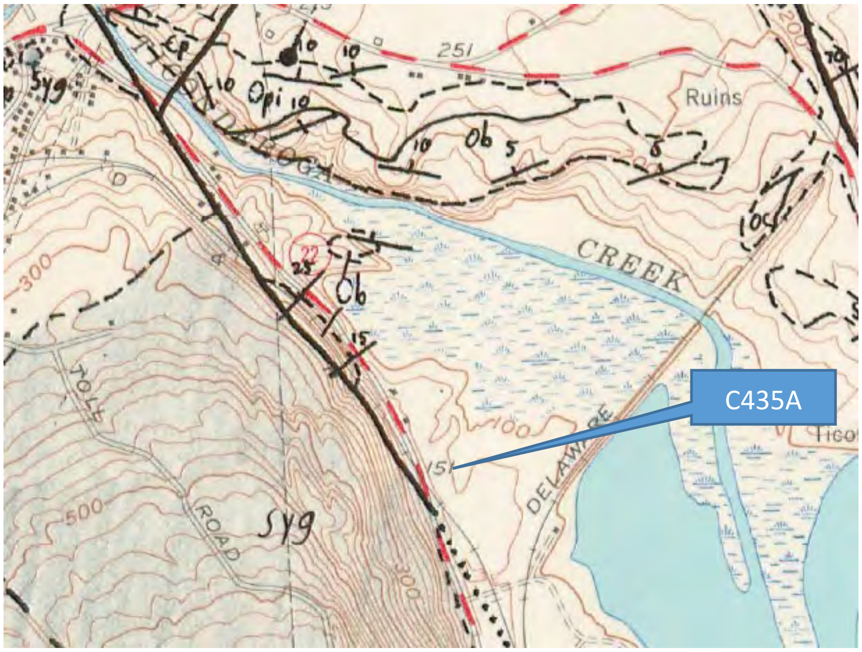

Figure 2: Sample location on USGS archive map by Walton (1966) where Ob denotes Bascom Formation and syg, denotes charnockite gneiss. Heavy black line is the fault. Reference: *Walton, M.S., 1966, Geologic map of the Ticonderoga quadrangle, NY: New York State Geological Survey, Open File Report 1g633, 1:24,000 Series.*

Failed samples from same site: C434A

Bedrock Unit: Bascom Formation (Ob) (Walton, 1966)

Ages: **C435A**: 79.8 ± 1.9 Ma

Summary: A major high angle fault zone along the west side of Rte. 22A a couple miles south of Ticonderoga, NY. This fault is mapped as a NW (325°) striking, steeply dipping, east-side-down normal fault, juxtaposing Mesoproterozoic charnockite gneiss on the west (footwall) with Ordovician Bascom Formation on the east (Walton, 1966). The fault zone is characterized by three 1-3 m wide zone covered intervals that are presumably fault gouge and/or saprolite, which are separated by large blocks of intact rock. Sample C435A was collected from a block of rock on the northwest side of the fault zone, which exhibits a 1-3 cm thick intense alteration rind with a rusty brown Fe-oxide rind and discontinuous calcite veins. Pockets of sparry calcite appear 'randomly' near the base of the rind often exhibiting large (~ 1 cm), transluscent, euhedral crystals. These crystals are not restricted to veins or fractures and appear to have crystallized freely in the presence of abundant space and fluids. Although no kinematic indicators can be clearly linked to the calcite, based on the mapped orientation of this fault and its high angle, we speculate that this fault may have accommodated NE-SW extension (or similar) at ~80 Ma. U-Pb isochron age is well defined and single domain, calcite is blocky and massive, both suggesting a single crystallization event.

Field Observations:

Overview

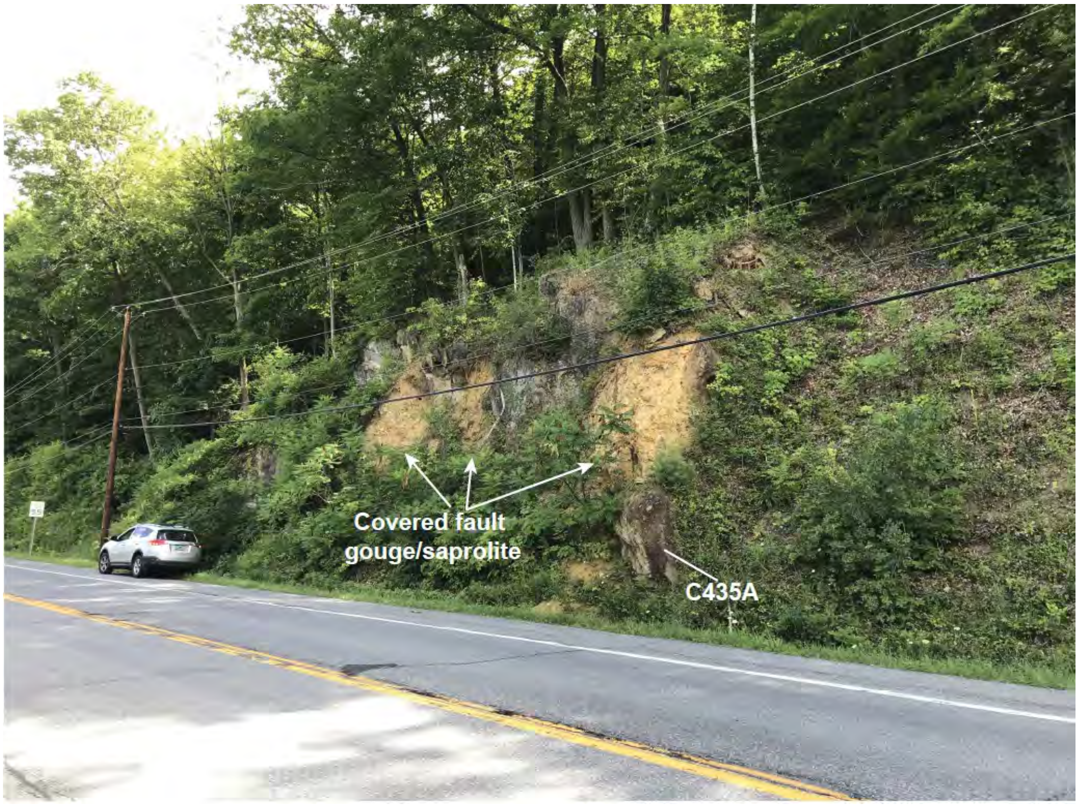

C435A:

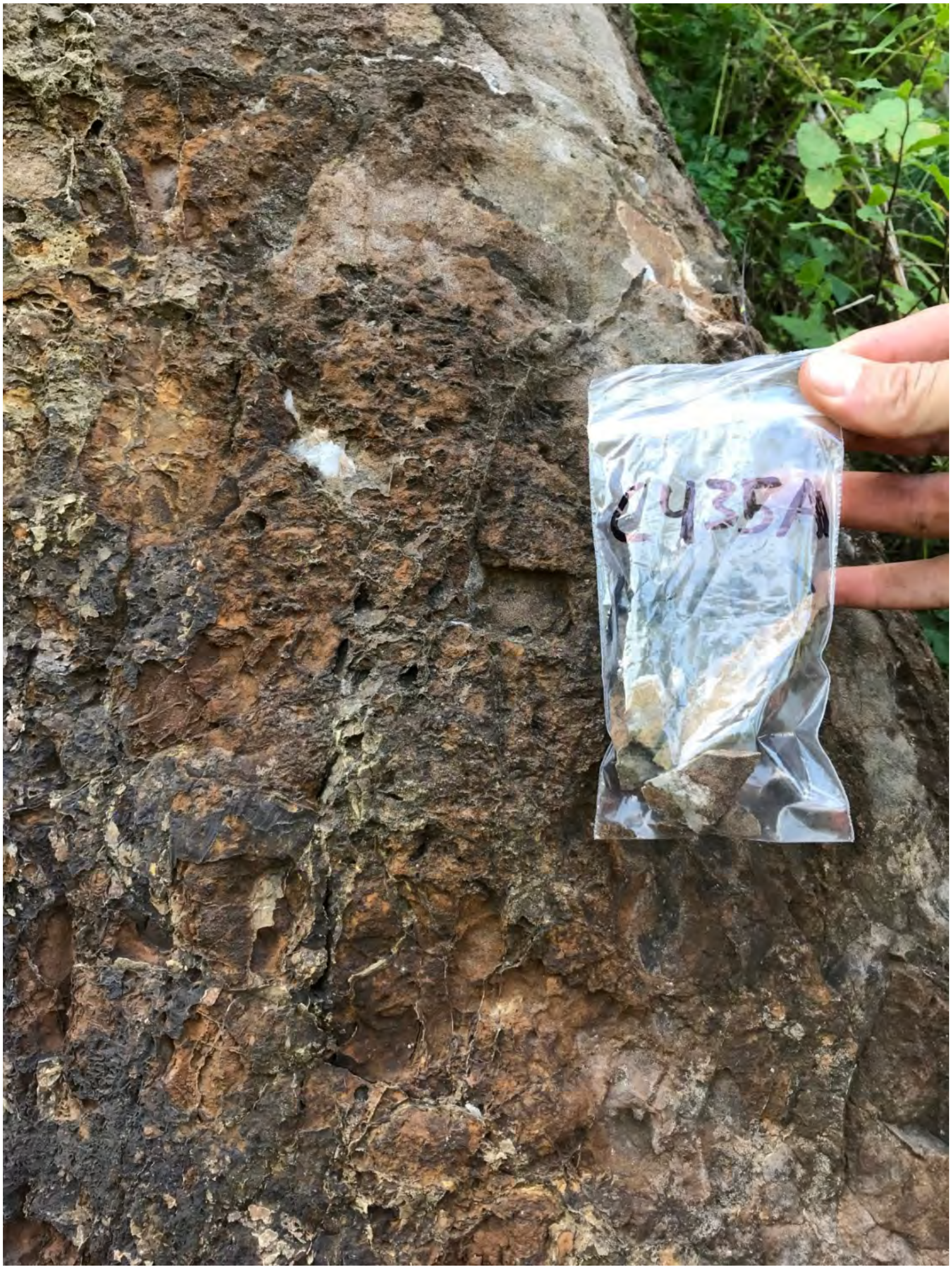

Lab Description:

C435A:

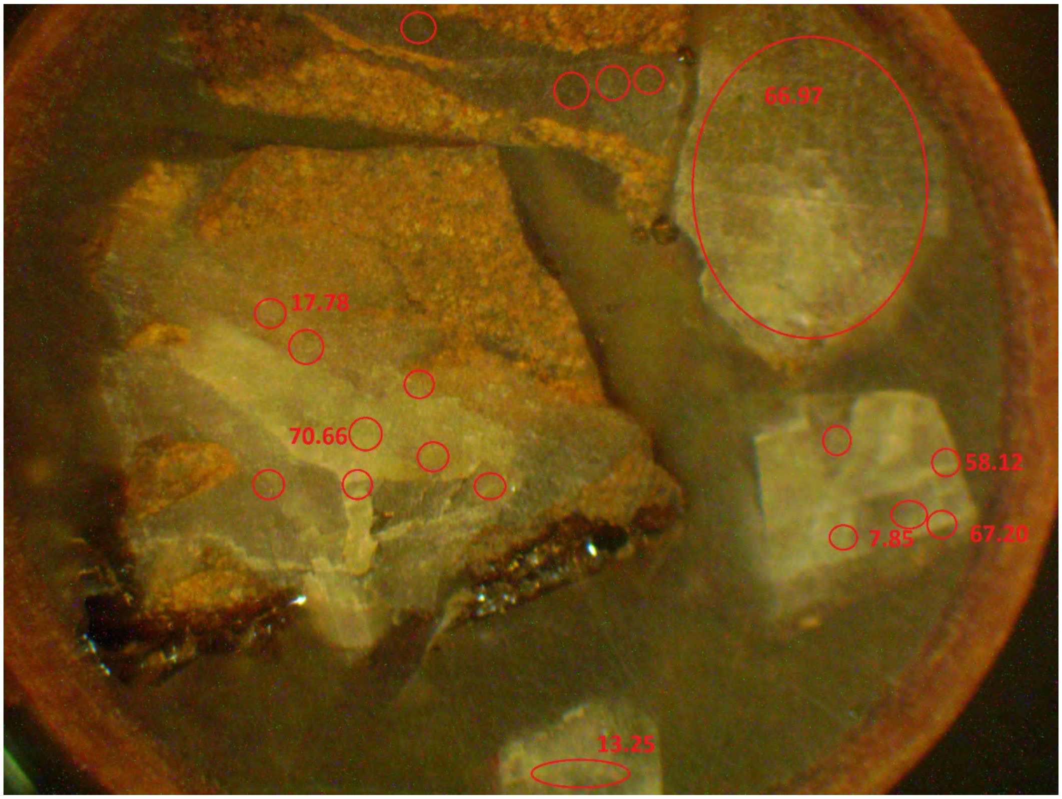

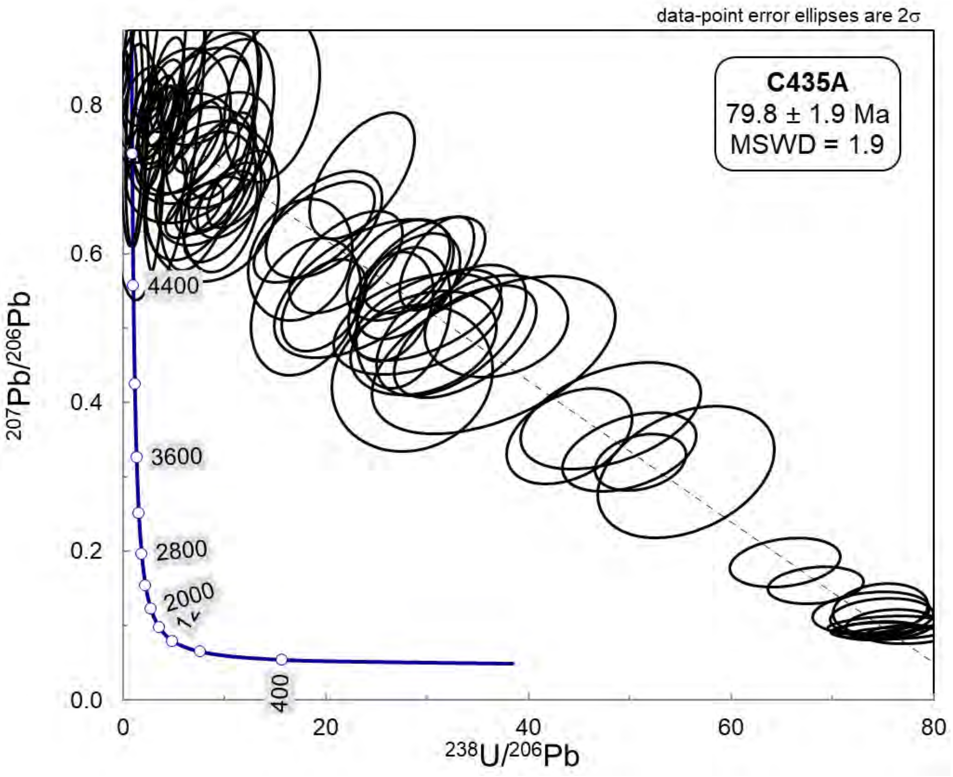

Supplement: Supplementary file 6 — Source Data [file 41467_2022_29680_MOESM6_ESM.zip › 2.zip!/appendix2b_site_summaries_21to50/36_rte22_lachute_river_final.pdf]
