# Supplementary material for: Pace of passive margin tectonism revealed by U-Pb dating of fracture-filling calcite
Source: Nat Commun. 2022 Apr 12;13:1953. doi: 10.1038/s41467-022-29680-z (PMC9005700; doi:10.1038/s41467-022-29680-z)

37: Rte. 22, Putnam Center, NY (43.7462360, -73.39920)

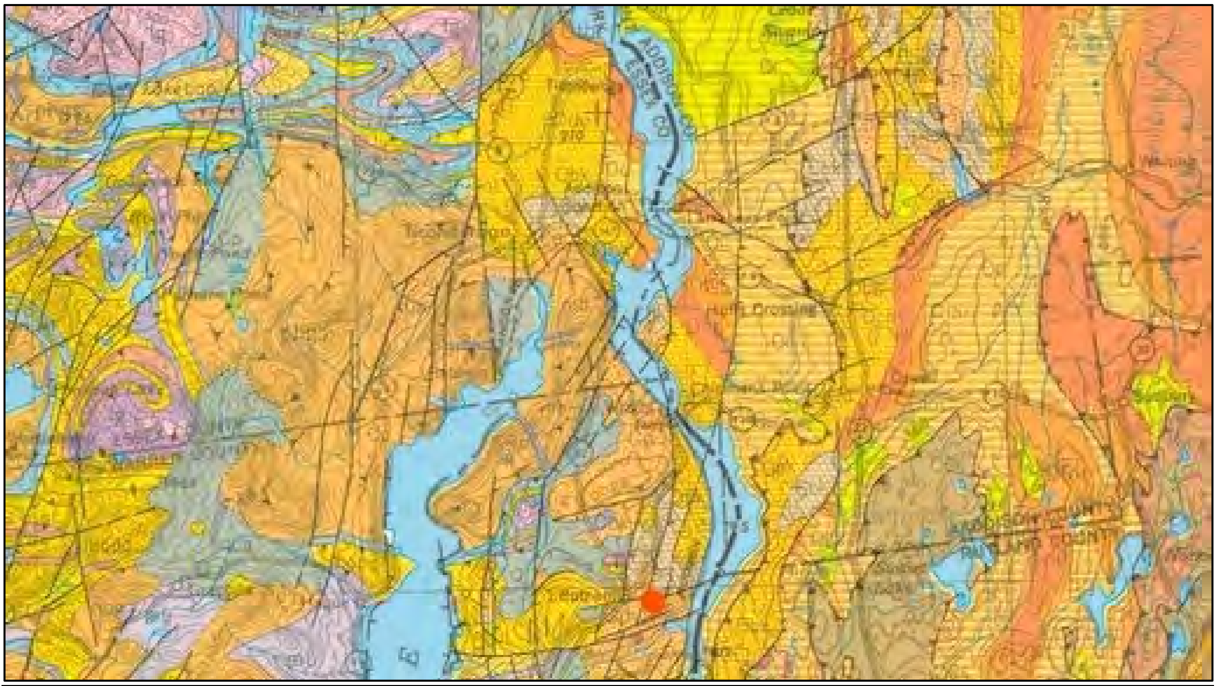

Figure 1: Sample located on Adirondack geologic map of Isachsen and Fisher, 1970.

Failed samples from same site: C438B

Bedrock Unit: Grenville Gneiss

Ages: **C438A**: 9.4 ± 9.9 Ma

Summary: This is a very large outcrop of gneiss on the west side of Rte. 22 near Putnam Center, NY. The gneiss is quite massive and broken apart by various fracture sets. However, a family of roughly N-S oriented fractures are coated with 0.3-1 cm thick sparry, translucent calcite veins, which are discontinuous yet abundant in the same orientation throughout the outcrop. Calcite is deposited more thickly in existing void spaces and recessions within the rock, wherever fluid was able to accumulate. It is also found in close proximity to pervasive set of high-angle striae or ‘grooves’ in the gneiss which record top down to the east and southeast slip (see Fig. 2). In at least two cases the striae can be seen ‘imprinted’ on the calcite vein, suggesting that the calcite is either coeval or younger than the bedrock striae. The idea that the calcite U-Pb age dates high-angle slip during a restricted time interval in the Miocene is supported by a single domain U-Pb isochron age and by the consistent visual character of the calcite throughout the outcrop. We thus interpret this site as evidence for late Miocene E-W extension with a nearly horizontal $\sigma 3$ and a vertical $\sigma 1$ stress. However, careful measurement and plotting of the striae suggests there may be two slip vectors, one oriented almost purely E-W and one oriented more NW-SE (Fig. 2).

Field Observations:

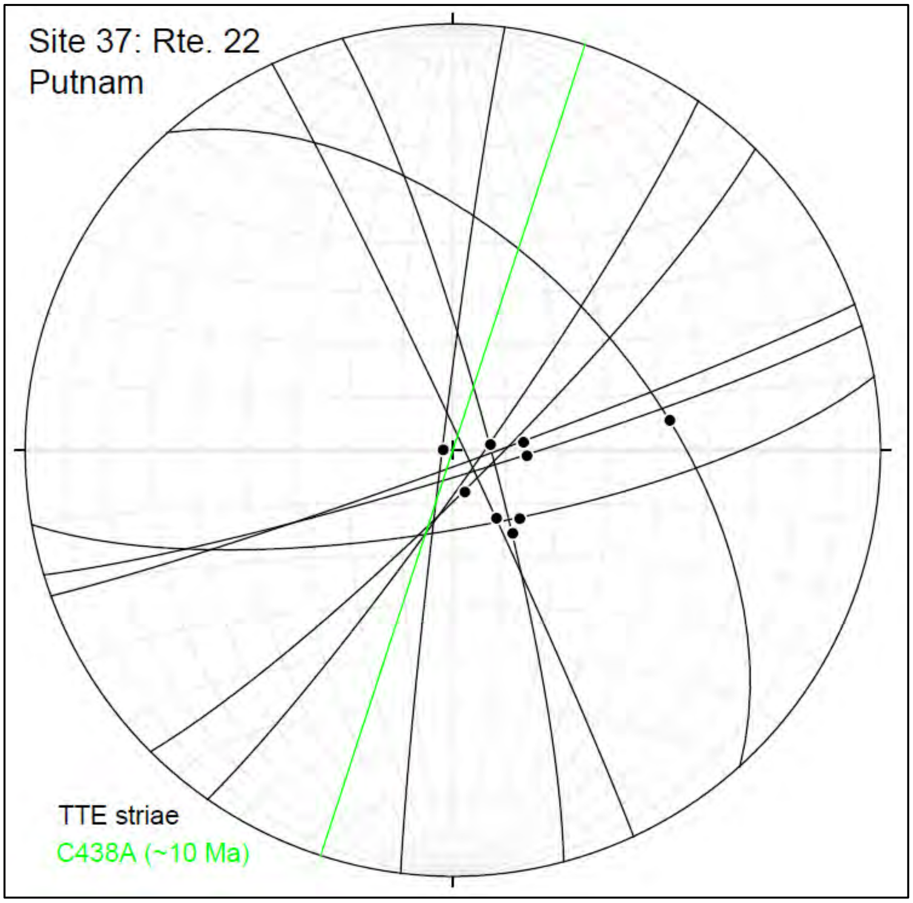

Figure 2: Stereonet showing measured calcite striae on fracture surfaces at site 37

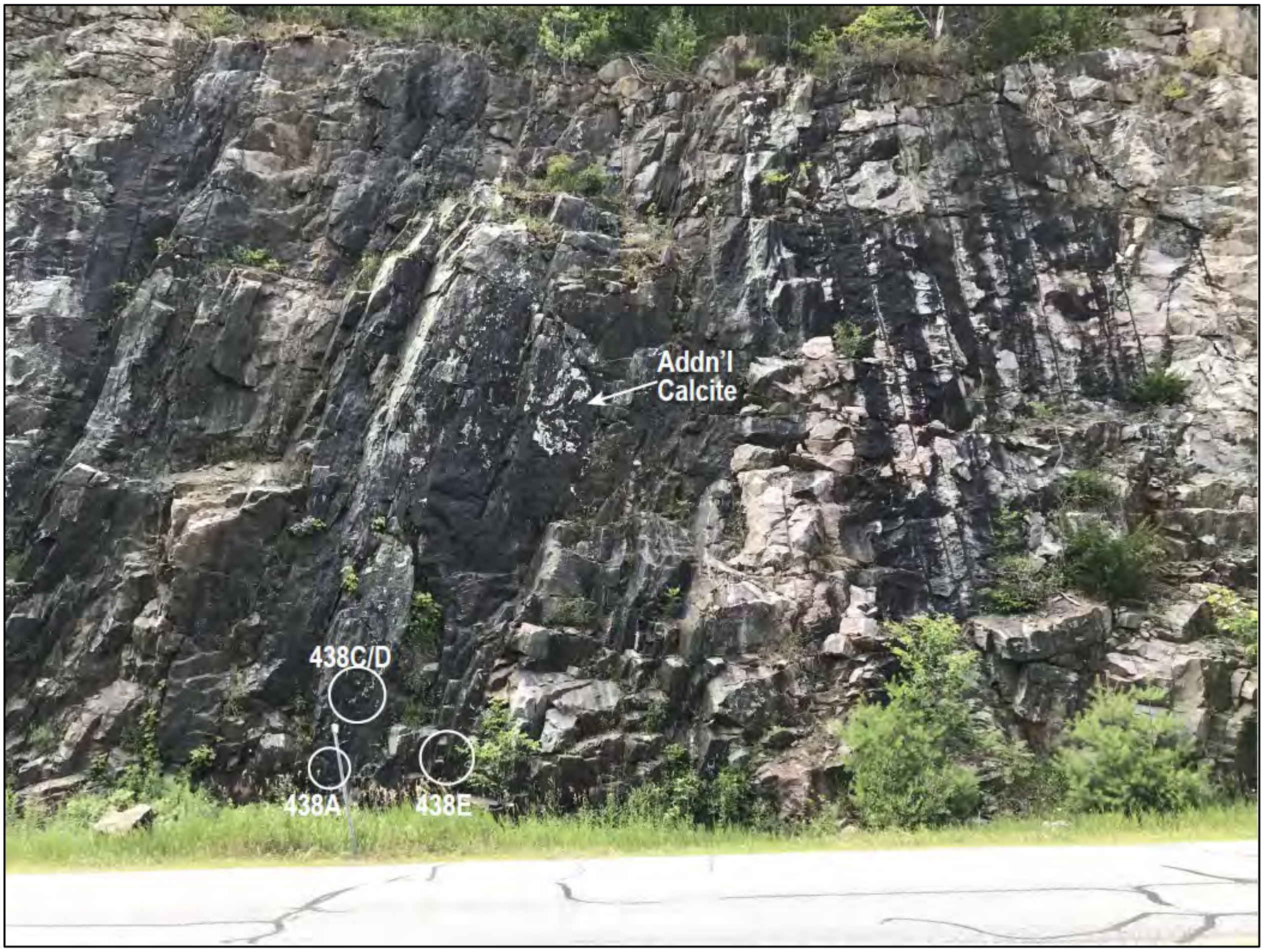

Figure 3: Overview of site 37 outcrop

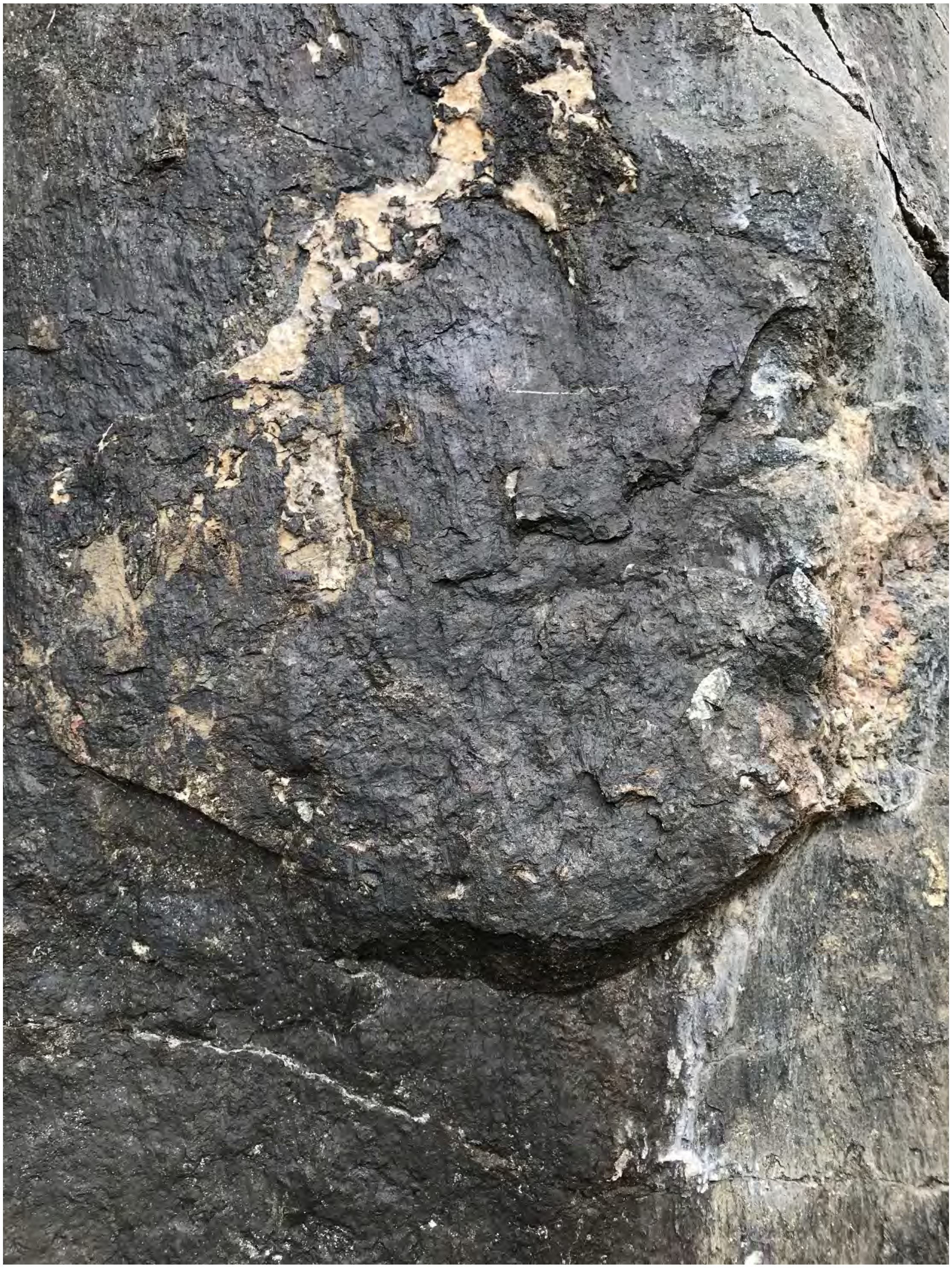

Figure 4: Example of calcite vein with striae overprinted on outer side. Apologies for lack of scale, I was clinging to the cliff face.

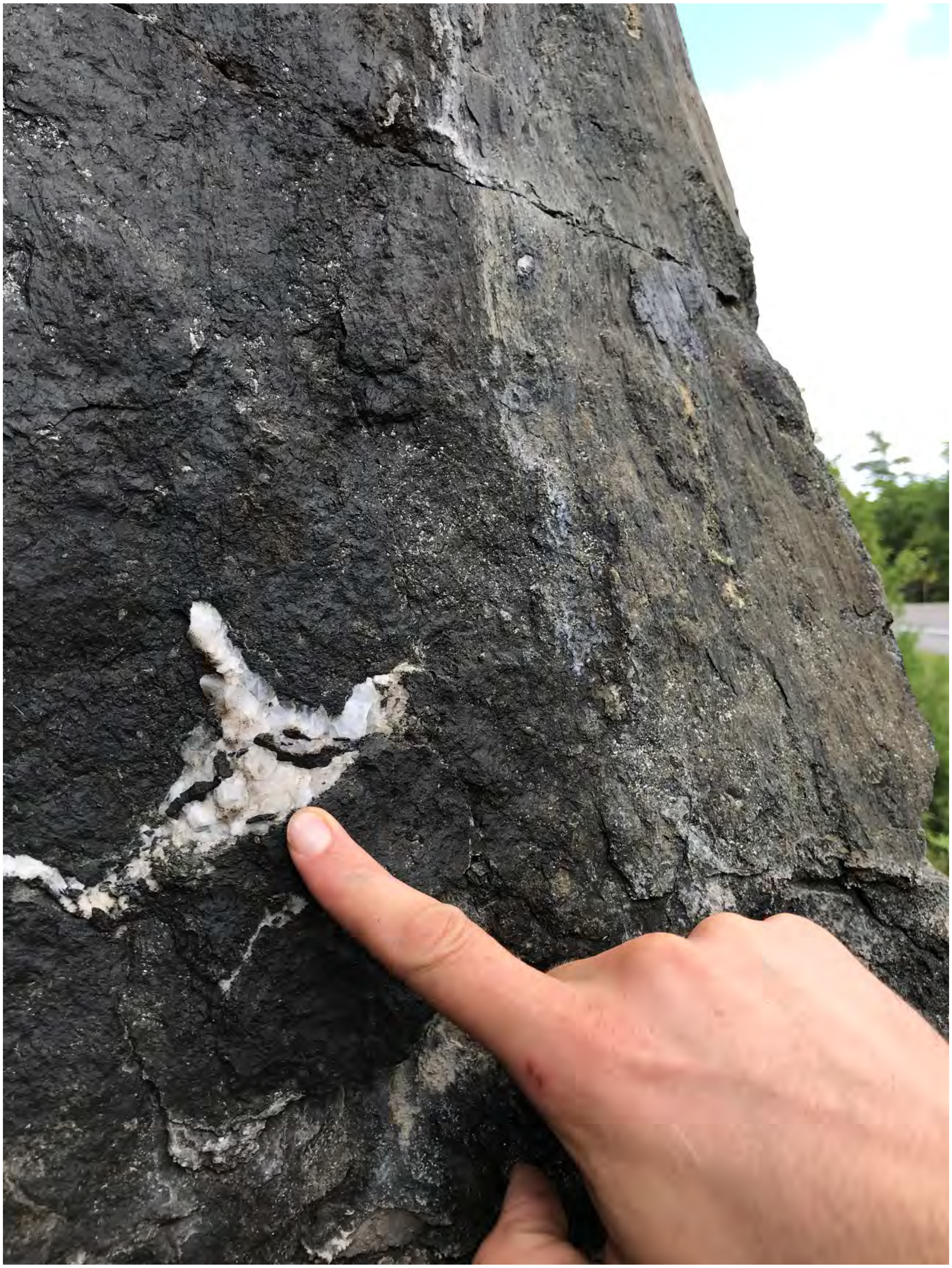

Figure 5: Pocket of calcite with host rock clast incorporated, suggesting calcite is associated with opening and slip along fractures. Not striae on surface to right.

C438A:

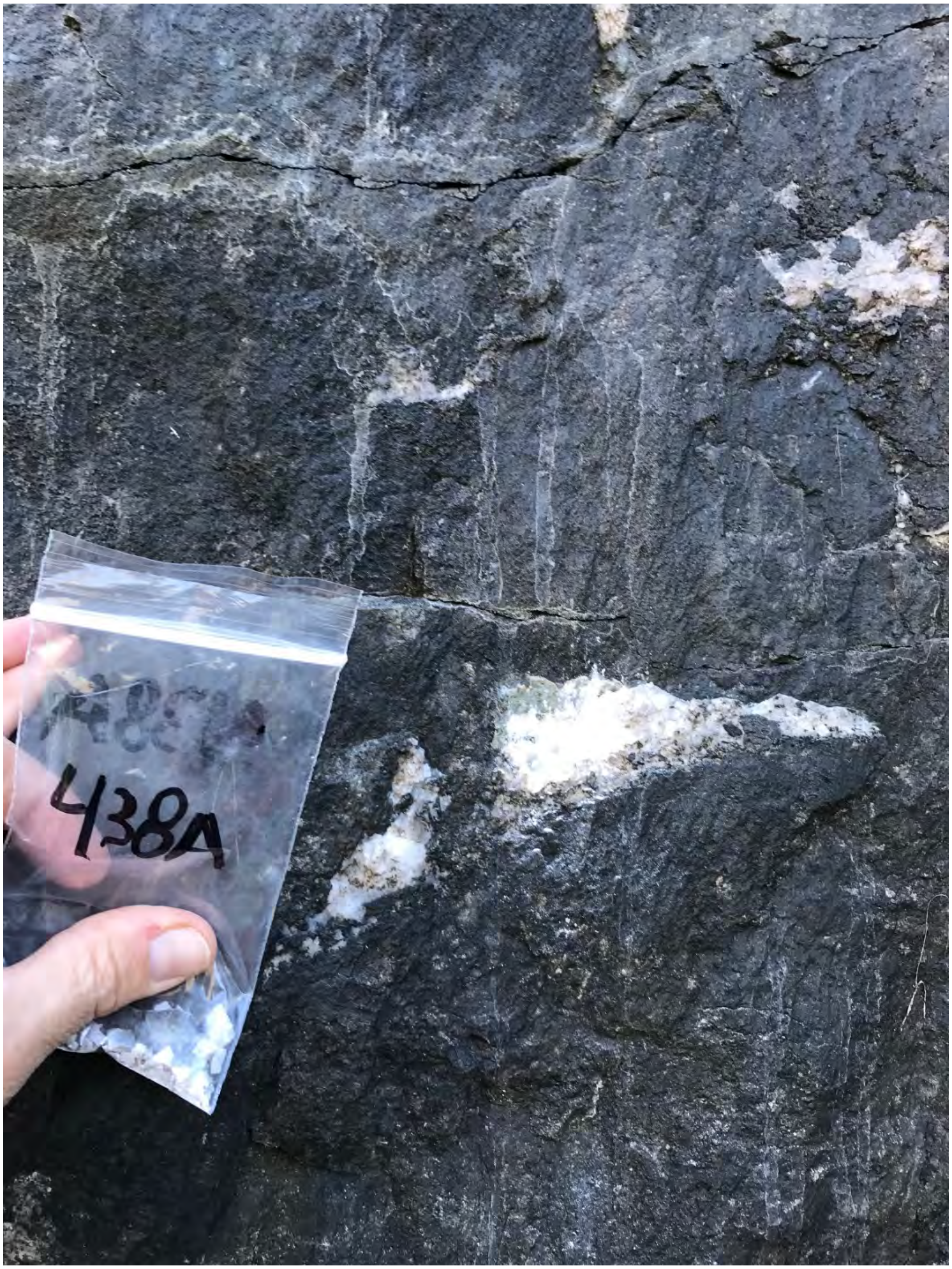

Lab Description:

C438A:

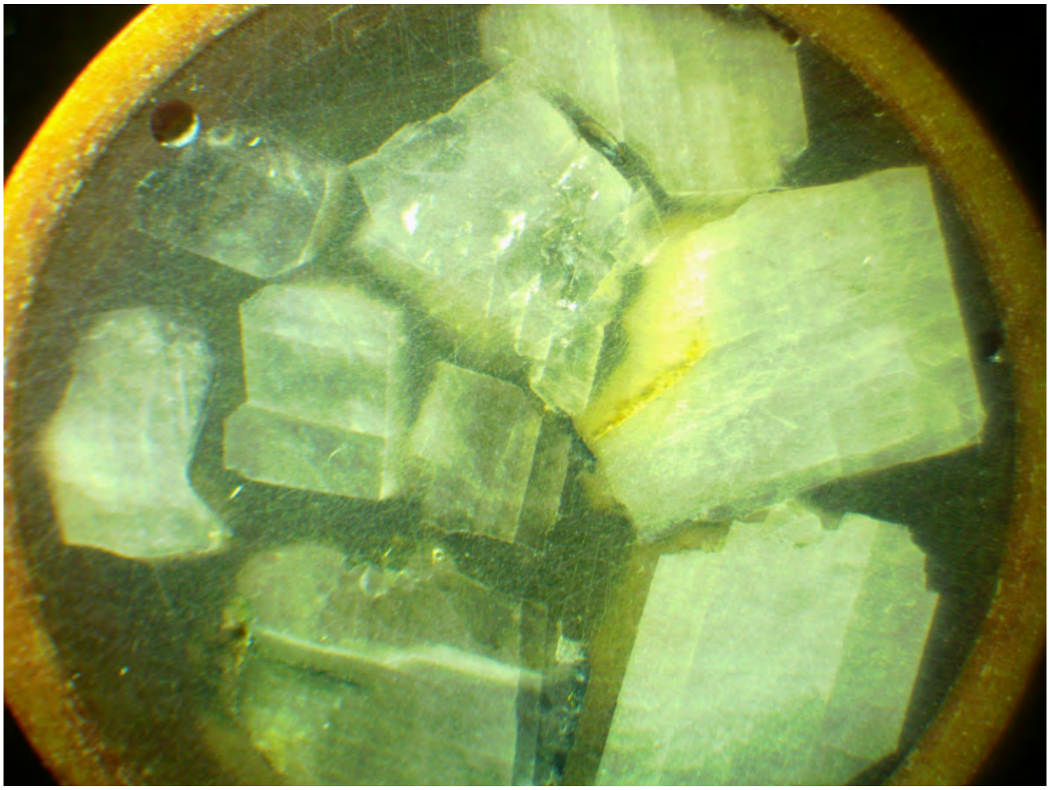

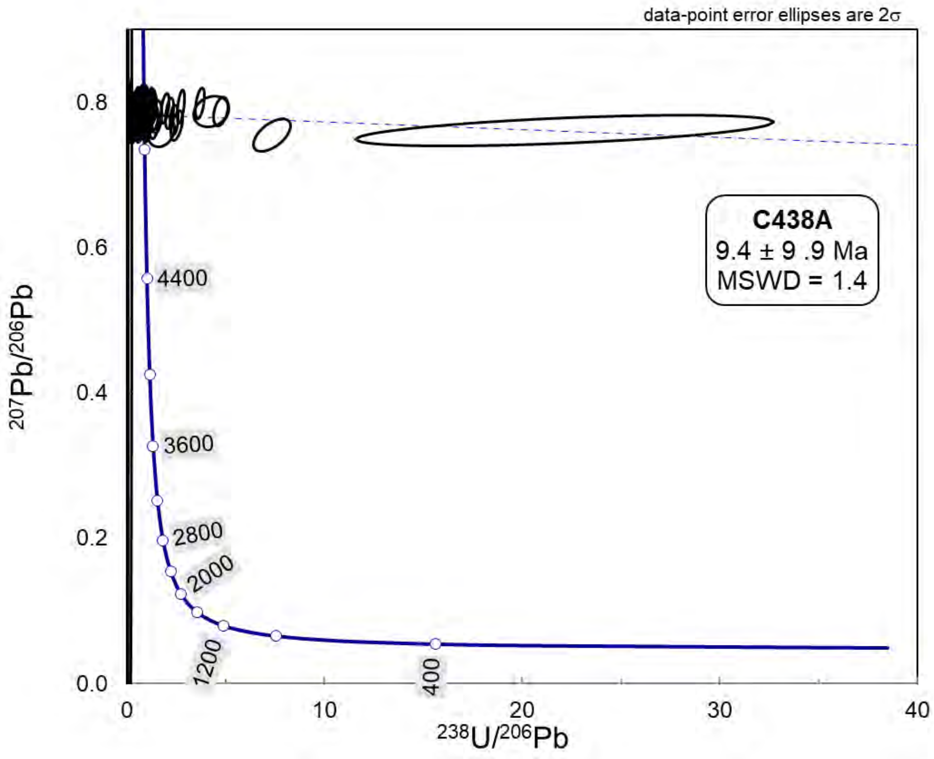

Supplement: Supplementary file 6 — Source Data [file 41467_2022_29680_MOESM6_ESM.zip › 2.zip!/appendix2b_site_summaries_21to50/37_rte22_putnam_center_final.pdf]
