# Supplementary material for: Pace of passive margin tectonism revealed by U-Pb dating of fracture-filling calcite
Source: Nat Commun. 2022 Apr 12;13:1953. doi: 10.1038/s41467-022-29680-z (PMC9005700; doi:10.1038/s41467-022-29680-z)

40: Garden Island North (44.28368515, -73.29733562)

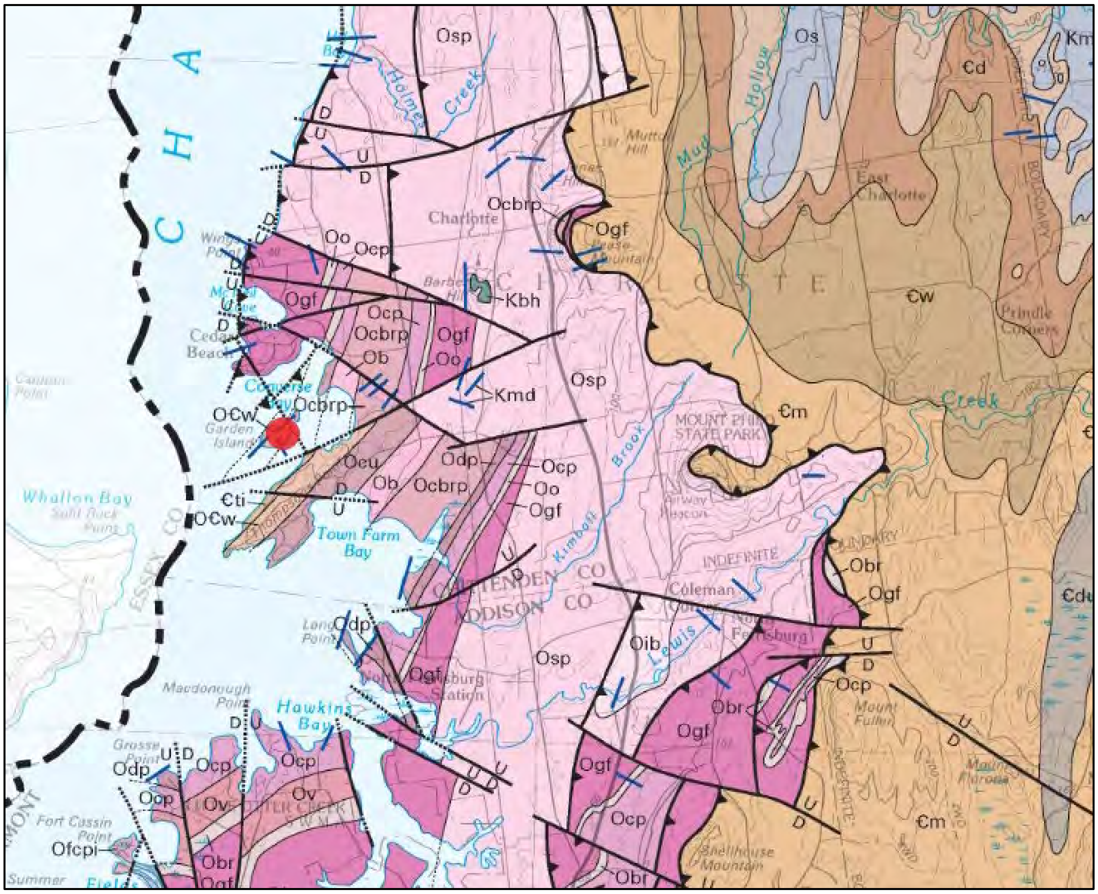

Figure 1: Sample location on state geologic map of Ratcliffe at al. (2011)

<u>Failed samples from same site:</u> None

<u>Bedrock Unit:</u> Whitehall Formation (Ocw)

<u>Ages</u>: C415A: 113.0 +/- 6.0 Ma

Summary: This site is located on a cliff on the north side of Garden Island in Charlotte, VT and is best accessed by boat from the Converse Bay fishing access. Thick solitary vein striking 297° and dipping vertically. No slip indicators and very continuous, planar nature suggest mode 1 fracture formed under a NNE-SSW sigma-3 stress orientation. This site is similar in age and orientation to the volcanic breccia at site 34 (C414B).

<u>Field Observations</u>:

C415A:

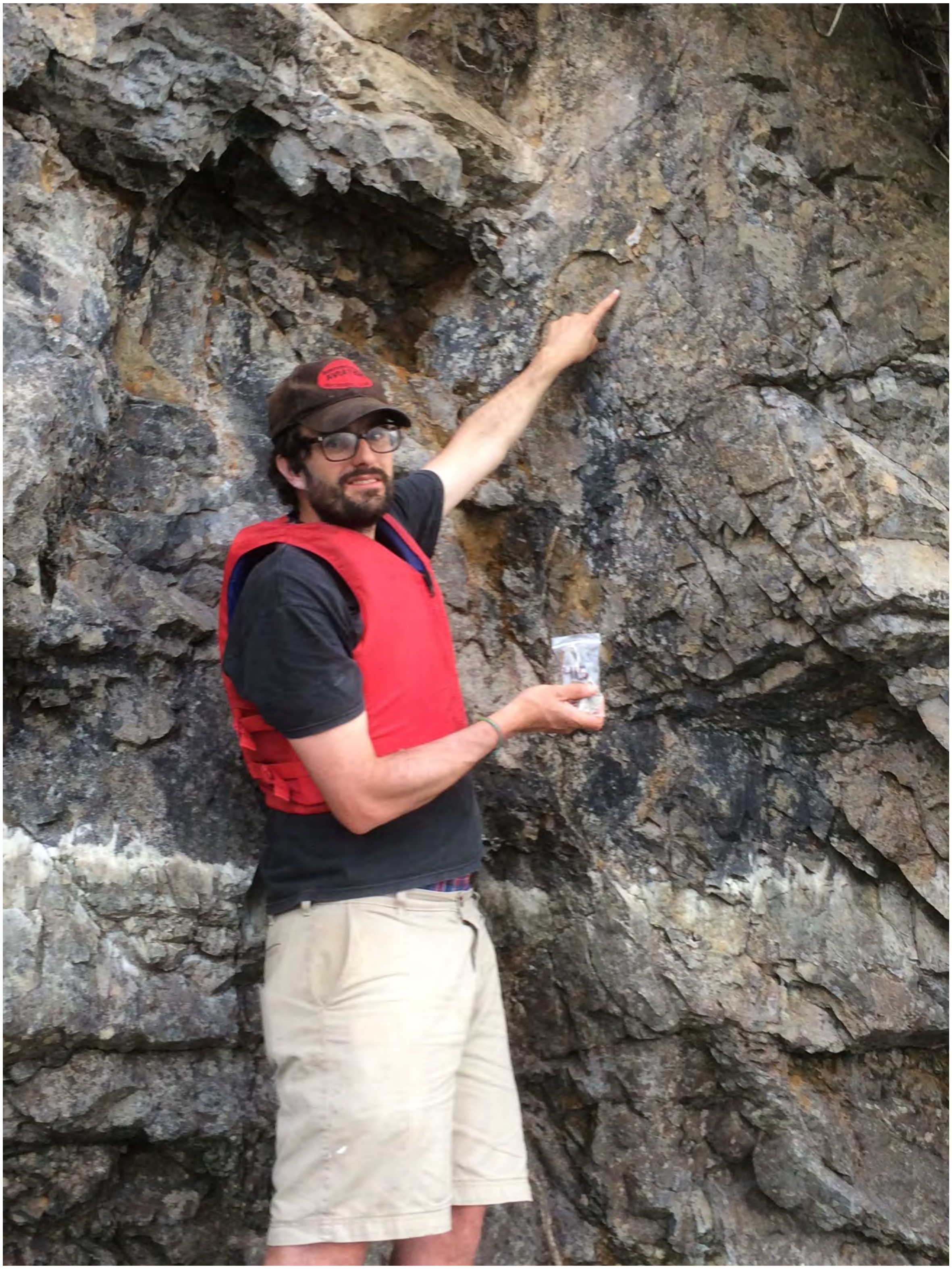

Lab Description:

C415A: Circles denote areas of interest, not laser shots

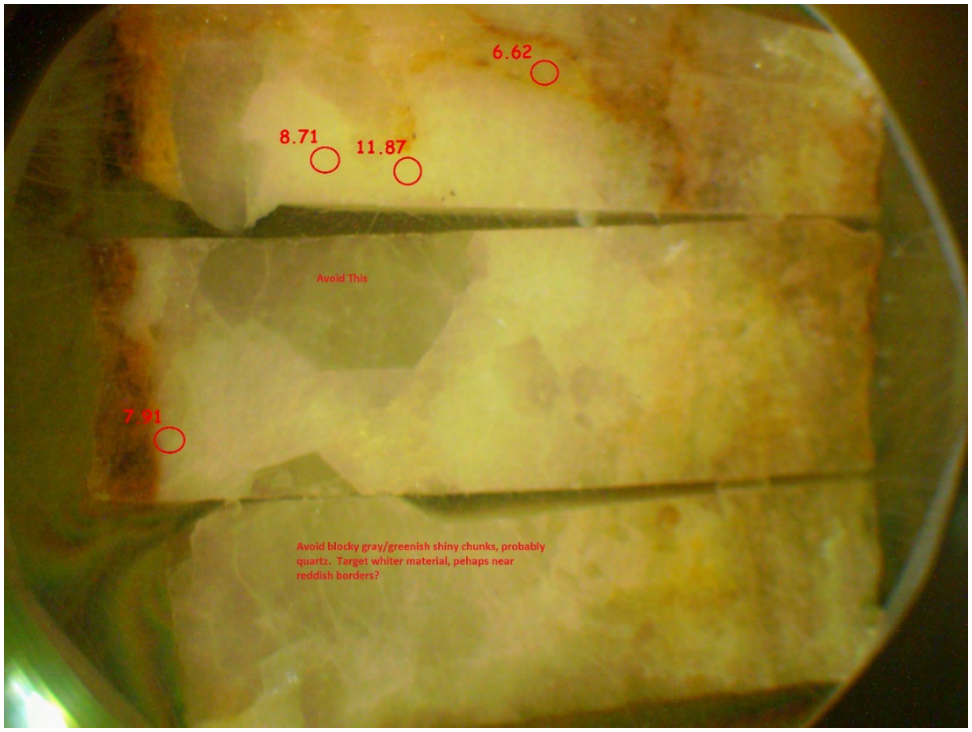

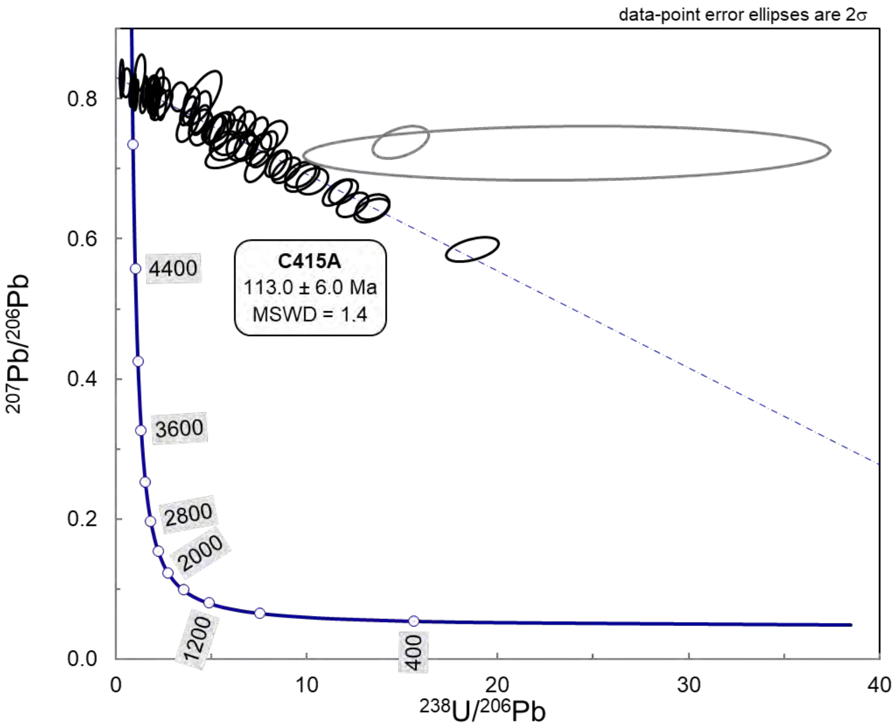

Supplement: Supplementary file 6 — Source Data [file 41467_2022_29680_MOESM6_ESM.zip › 2.zip!/appendix2b_site_summaries_21to50/40_garden_island_north_final.pdf]
