# Supplementary material for: Pace of passive margin tectonism revealed by U-Pb dating of fracture-filling calcite
Source: Nat Commun. 2022 Apr 12;13:1953. doi: 10.1038/s41467-022-29680-z (PMC9005700; doi:10.1038/s41467-022-29680-z)
Supplement: Supplementary file 6 — Source Data [file 41467_2022_29680_MOESM6_ESM.zip › 2.zip!/appendix2b_site_summaries_21to50/40_hartford_dunkin_final.pdf]

42: Dunkin Donuts, Newington, CT (41.686530, -72.706668)

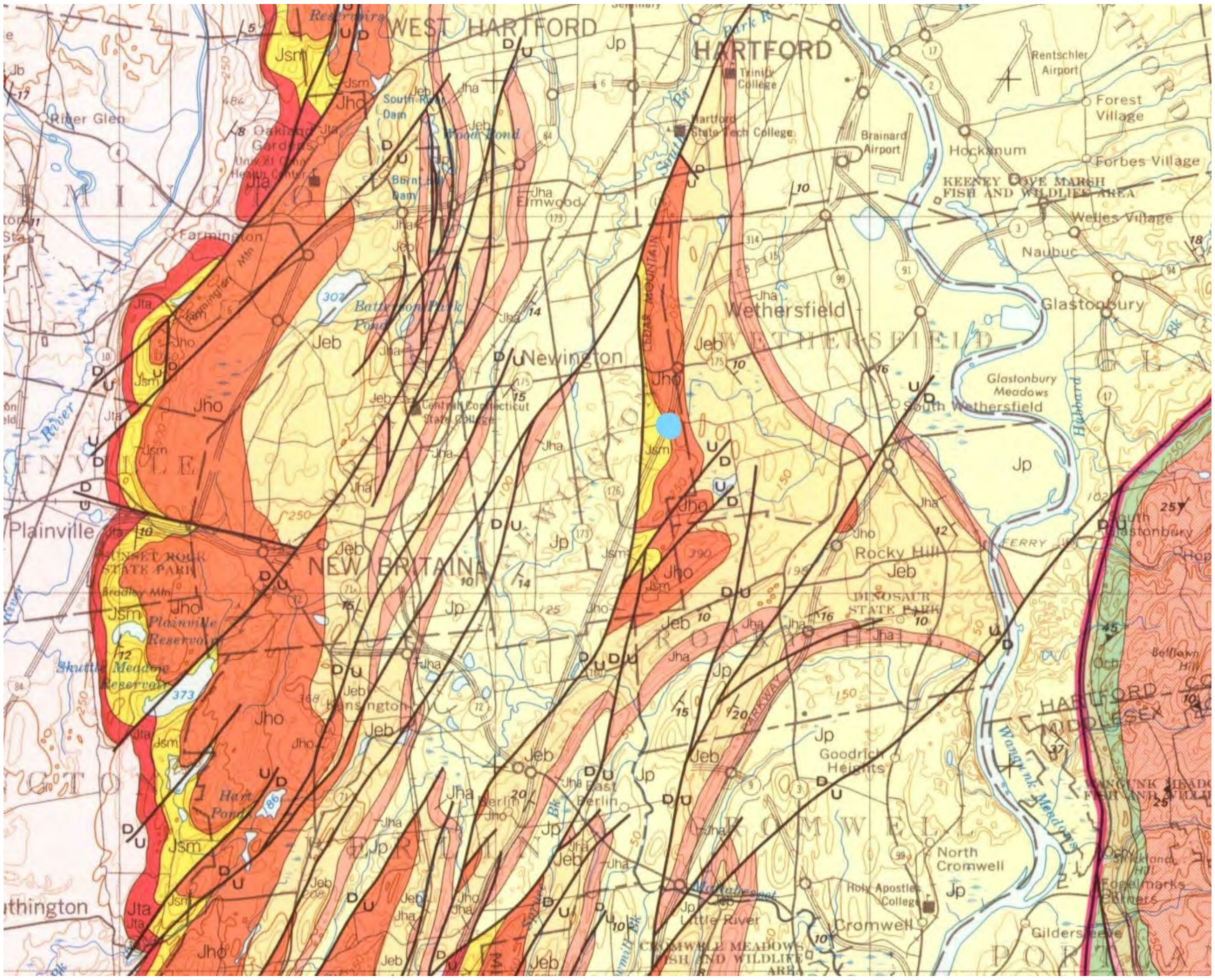

Figure 1: Sample location (blue dot) on Connecticut state geologic map of Rodgers, 1985.

<u>Failed samples from same site:</u> C374,C375, C376, C377

<u>Ages</u>: C378A: 51 ± 10 Ma

Summary: This site is located at the back of the Wendy's parking lot, between the Dunkin Donuts and Wendy's on the east side of the route 5/15 northbound lane. A well-developed sub-vertical vein or fault zone cuts through the Holyoke Basalt striking roughly 168° and dipping 60° W, roughly parallel to a mapped extensional fault immediately to its west (Fig. 1). The same feature can be viewed just north of the Dunkin Donuts drive-through window as it extends northward. The U-Pb data are puzzling and the age may be unreliable because the isochron is scattered and exhibits a very low slope suggesting a low initial $^{207}Pb/^{206}Pb$ ratio of around 0.45. Additional observations of the field site and hand samples are required to develop a robust interpretation here.

Field Descriptions:

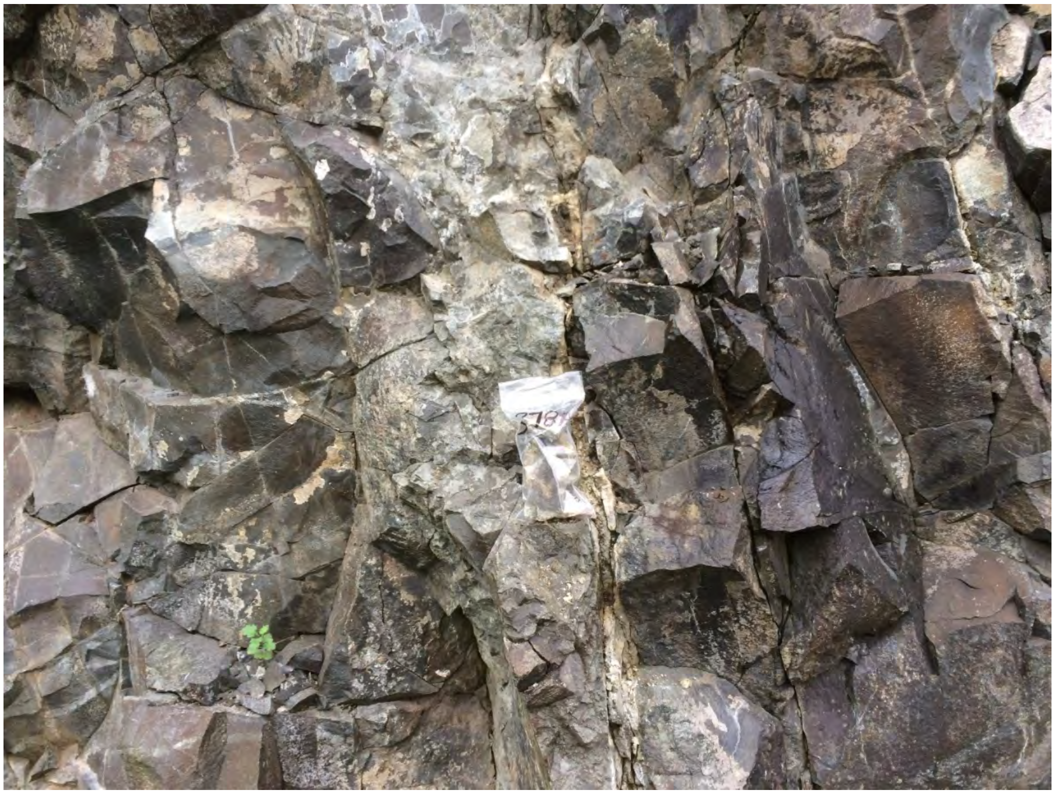

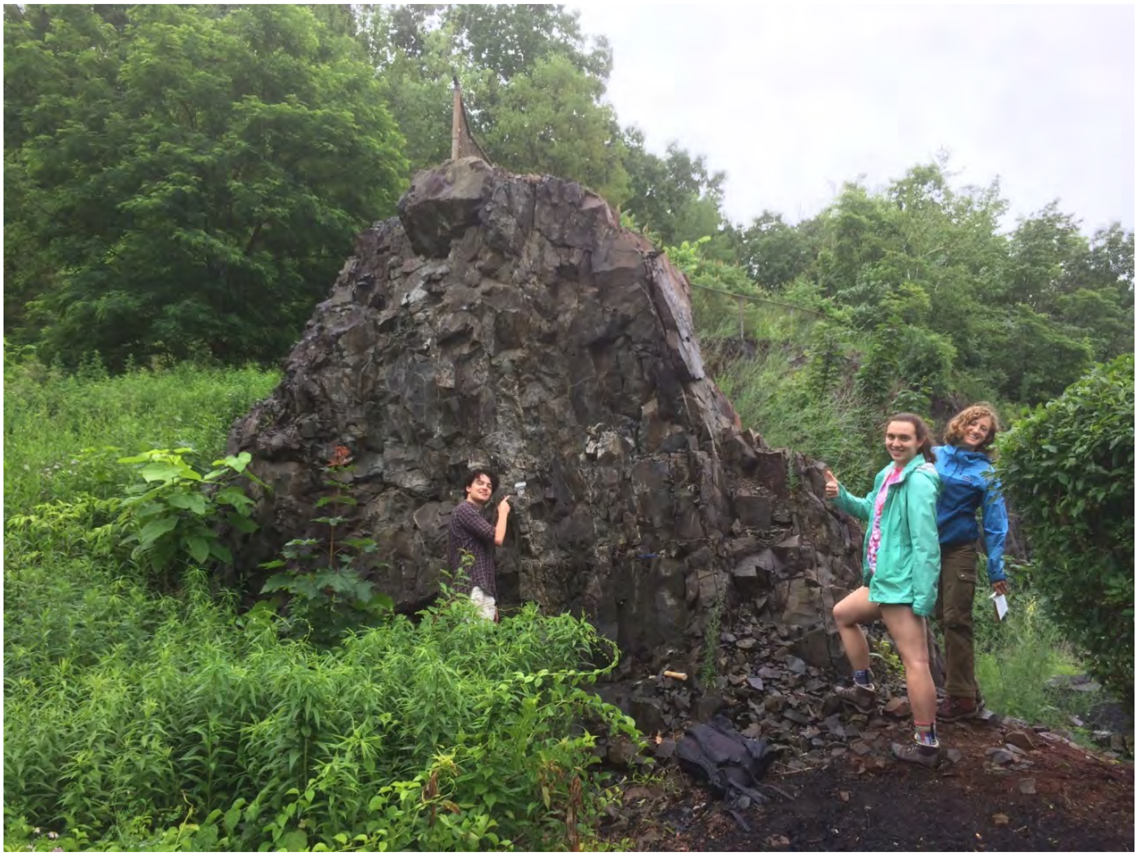

Lab Description:

C378a

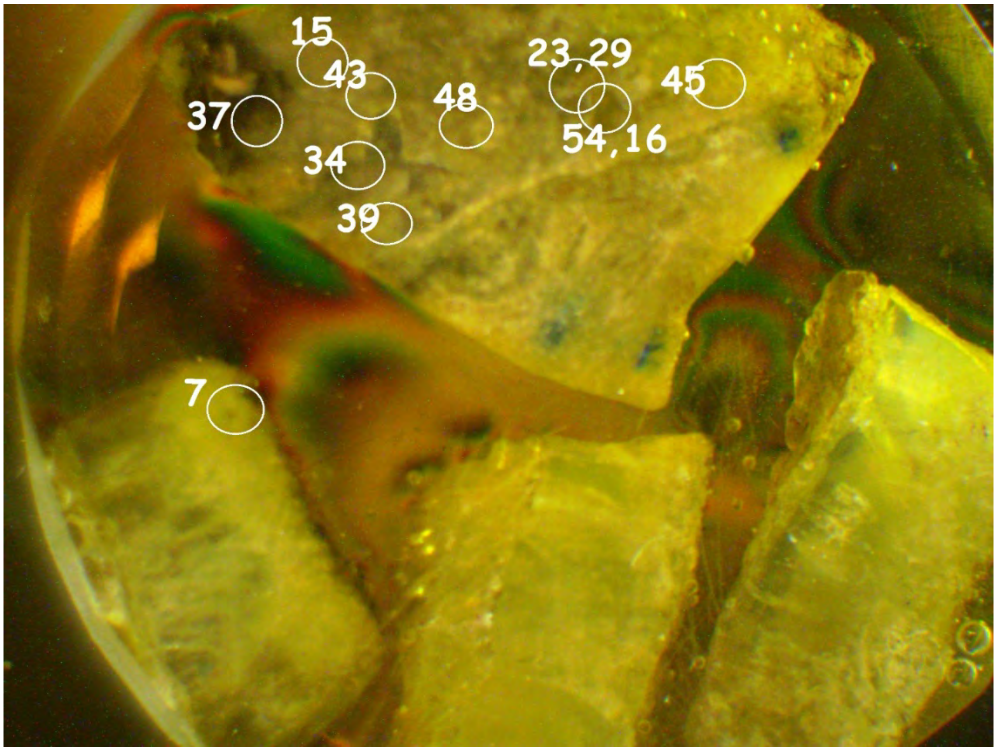
15
43
37
34
39
48
23,29
54,16
45
7

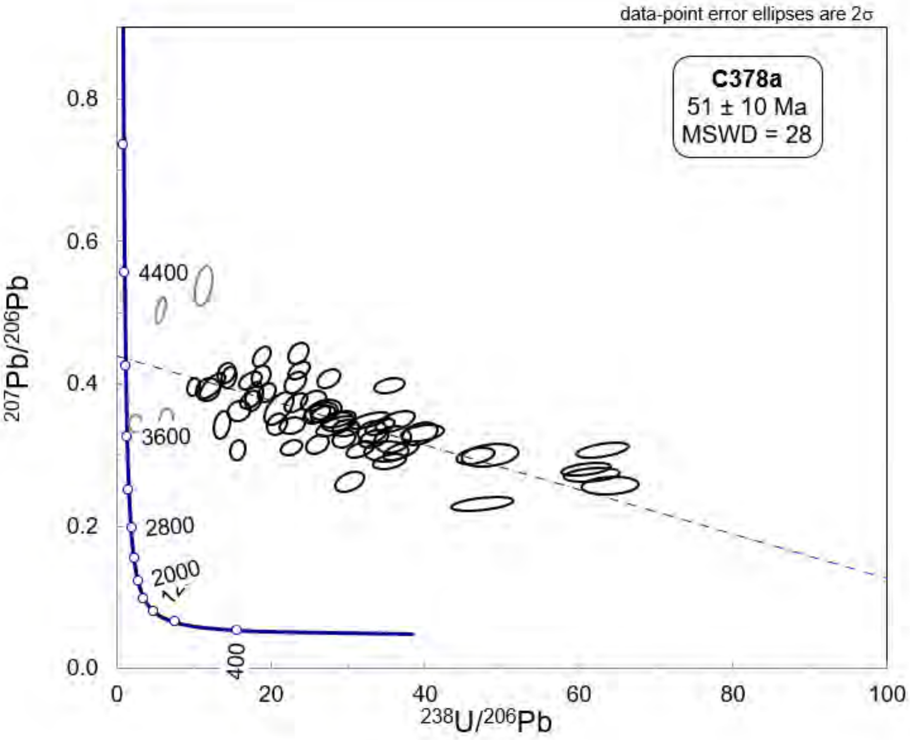
data-point error ellipses are 2σ
C378a
51 ± 10 Ma
MSWD = 28
207Pb/206Pb
238U/206Pb
4400
3600
2800
2000
400
0.0
0.2
0.4
0.6
0.8
0
20
40
60
80
100
