# Supplementary material for: Pace of passive margin tectonism revealed by U-Pb dating of fracture-filling calcite
Source: Nat Commun. 2022 Apr 12;13:1953. doi: 10.1038/s41467-022-29680-z (PMC9005700; doi:10.1038/s41467-022-29680-z)
Supplement: Supplementary file 6 — Source Data [file 41467_2022_29680_MOESM6_ESM.zip › 2.zip!/appendix2b_site_summaries_21to50/41_Hills Point_central_final.pdf]

41: Hills Point Central (44.34599, -73.28486)

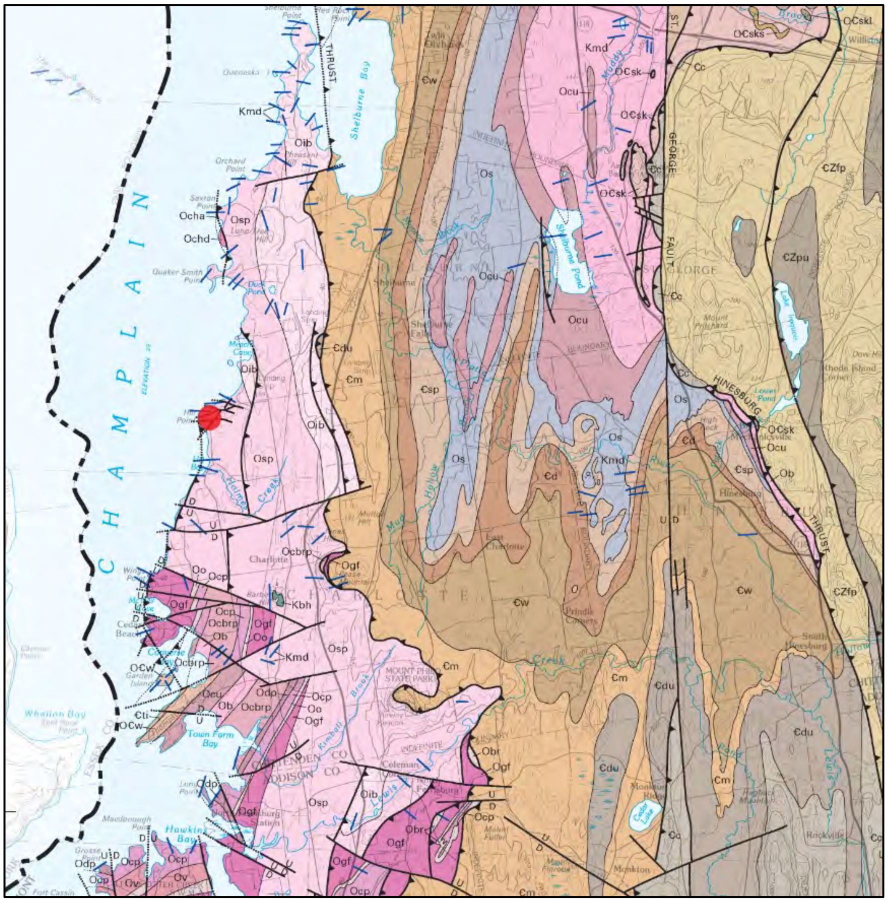

Figure 1: Sample location on state geologic map of Ratcliffe at al. (2011)

<u>Failed samples from same site:</u> None!

<u>Bedrock Unit:</u> Stony Point Formation (Osp)

<u>Ages</u>: **C230A**: 102.0 ± 3.7 **C230B**: 95.5 ± 1.1 **C230C**: 100.1 ± 3.7 **C230D**: 112.9 ± 3.0 **C230E**: 77.3 ± 0.9 **C230F**: 96.1 ± 6.6 **C230H**: 92.8 ± 1.2 **C230I**: 110.0 ± 2.2

Summary: Complex fault zone exposed along Lake Champlain shoreline at Hills Point, Charlotte, VT. Best access by canoe or land owner stairs to beach. Site located about 30 m north of site 13 and about 100 m south of site 14. A very complex fault zone with a similar distribution of ages to what is observed at site 13. Main fault zone strikes 60° and dips 50° SE, splaying upward into a horsetail structure that records multiple episodes of slip. Several sub-vertical veins and cross faults intersect the main fault zone from below, some of which are offset by the main fault zone. Overall calcite ages are high quality and single domain ages suggesting they do record specific episodes of slip within the fault zone, which seems to record four events:

1) The ~113-110 Ma event is recorded by two vertical mode-1 veins oriented roughly E-W (C230D and C230I). C230D is offset by the lower horsetail and sitting just above the fault zone. It is visually distinct from the calcite tabs within the fault zone because of its 2-3 thickness and milky white to orangeish

color. Although this fragment has been significantly translated from its original position, its similar orientation to other nearby veins of the same character gives come confidence in inferring a roughly N-S oriented σ_3 at this time. C230I is a sub-vertical vein sitting south and above the fault zone with only minor offsets by cleavage-plane slip (Fig. 2).

2) The ~100-102 Ma event appears in the lower section of the main fault (C230A) as well as the middle horsetail (C230C). Faint growth striae on the underside of sample C230C suggest N-S extension at this time (Fig. 2).

3) The ~96 Ma event is seen on a lower accessory fault (C230F) and in a calcite tab within the middle horsetail (C230B). Slickensides along both surfaces suggest oblique normal slip with a TTSE sense and a trend of ~126° (Fig. 2).

4) The ~93 Ma event appears to be a N-S compressional event based on thrust-sense slickenfibres on the dated calcite tab. However, we place low confidence in this thrust interpretation.

5) The 77 Ma event (C230E) is recorded by a 1-2 cm thick calcite tab of clear to greenish, sparry, syntaxial calcite in the middle horsetail. No slip indicators were observed although it is inferred to record generally N-S extension based on the void space required to form this tabular, sparry calcite with elongate crystals formed perpendicular to wall rock.

<u>Field Observations</u>:

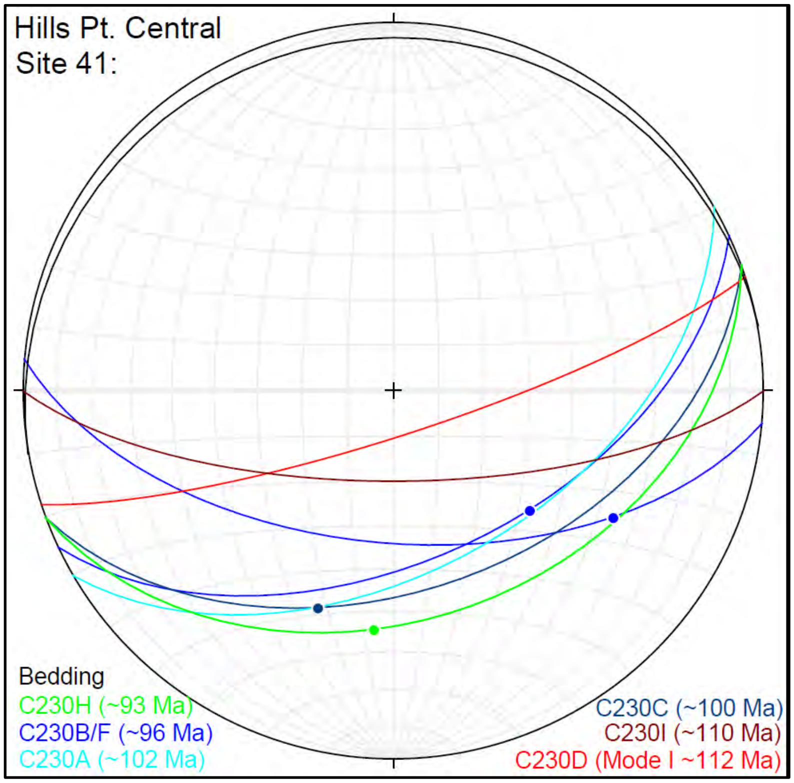

Figure 2: Stereonet showing orientations of features dated at site 41

General photos (All samples have prefix C230):

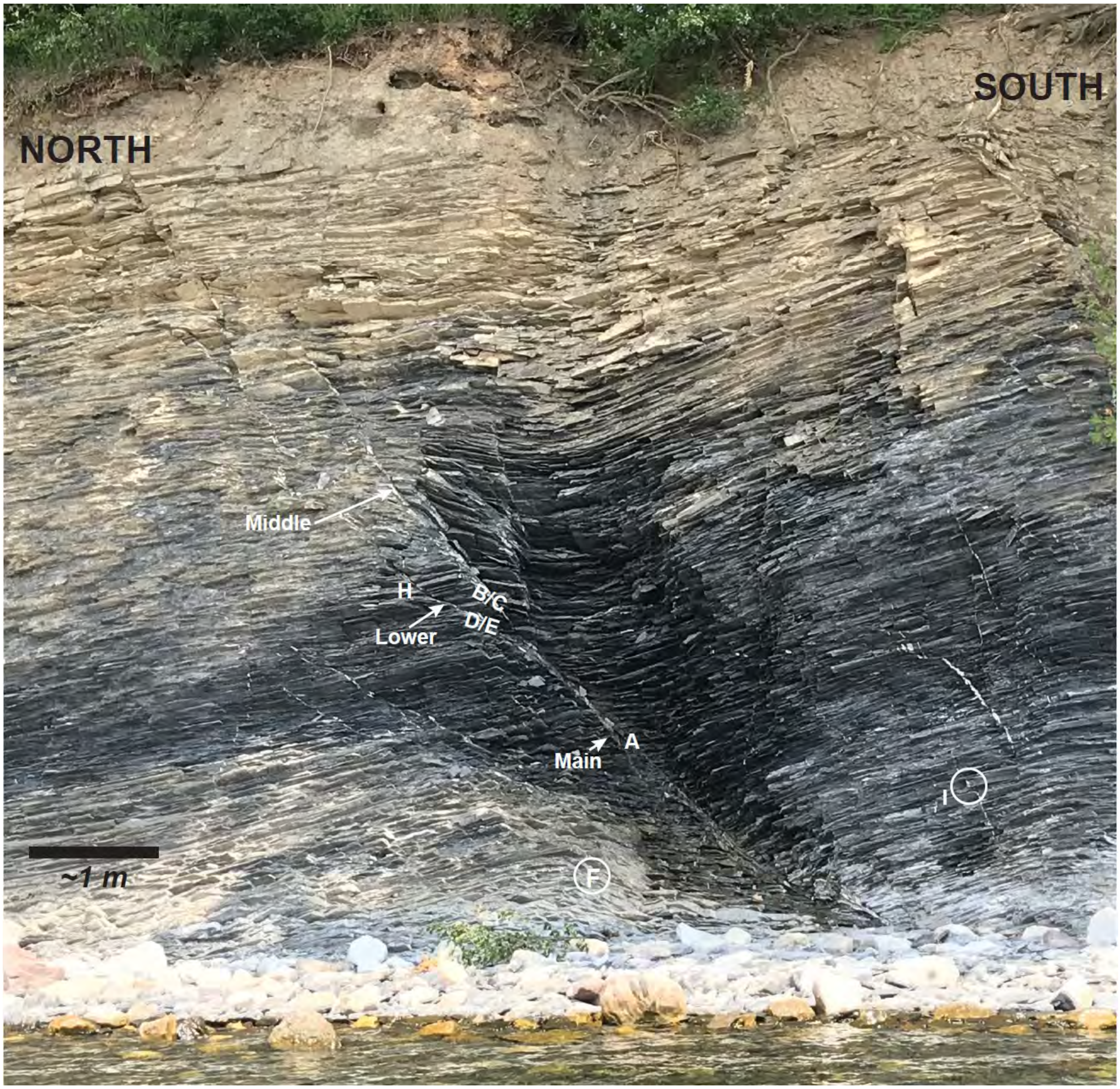

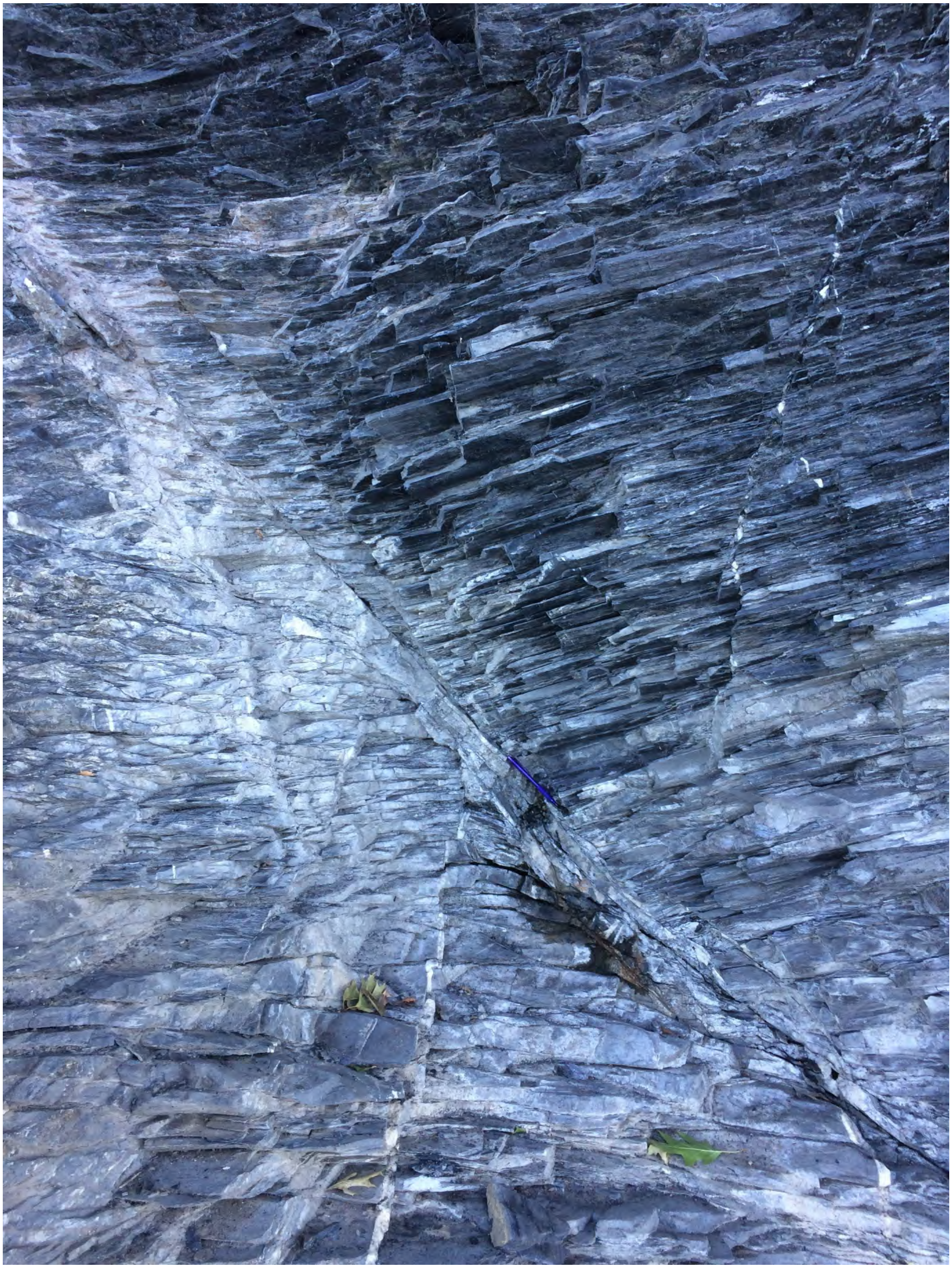

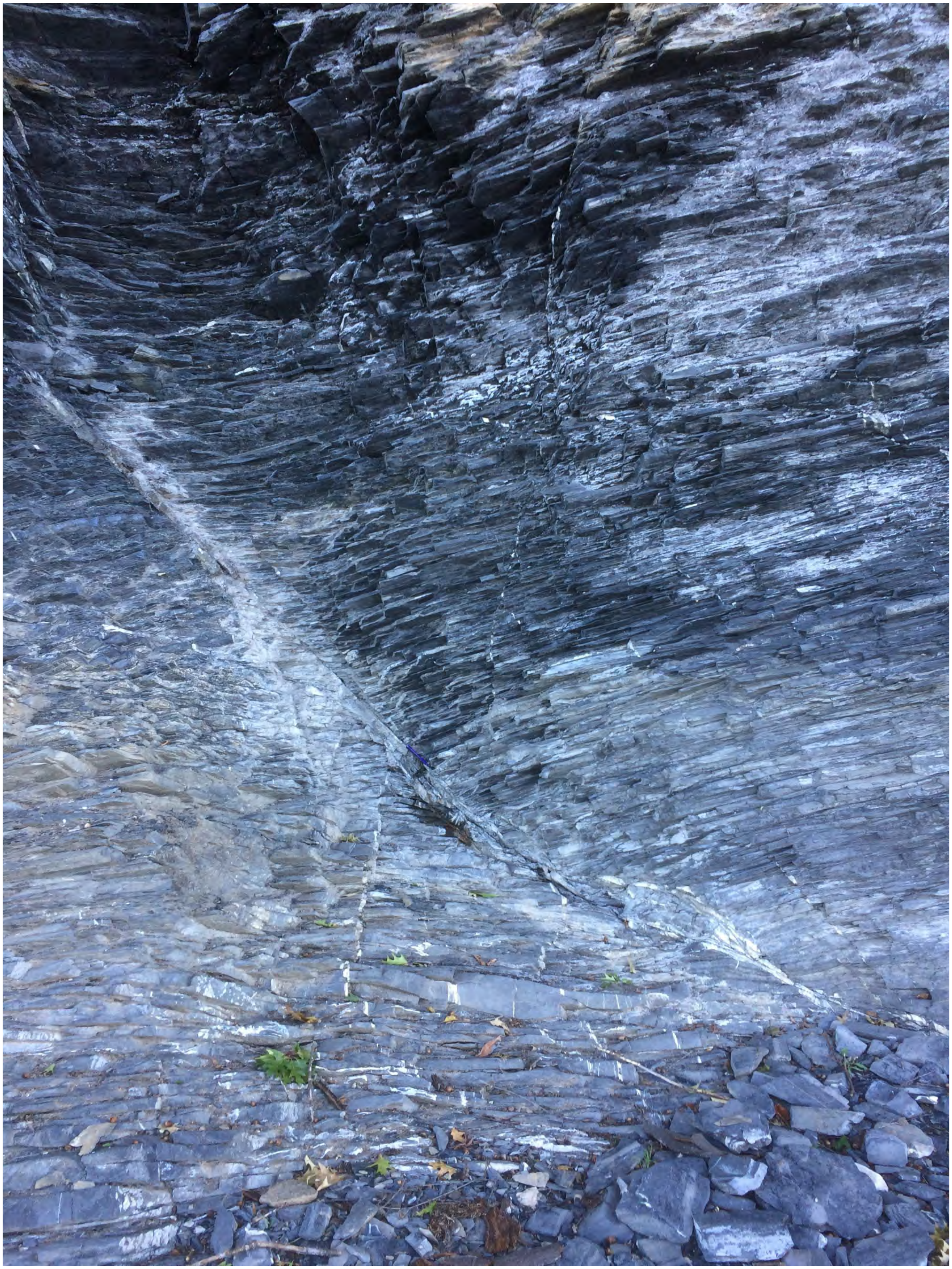

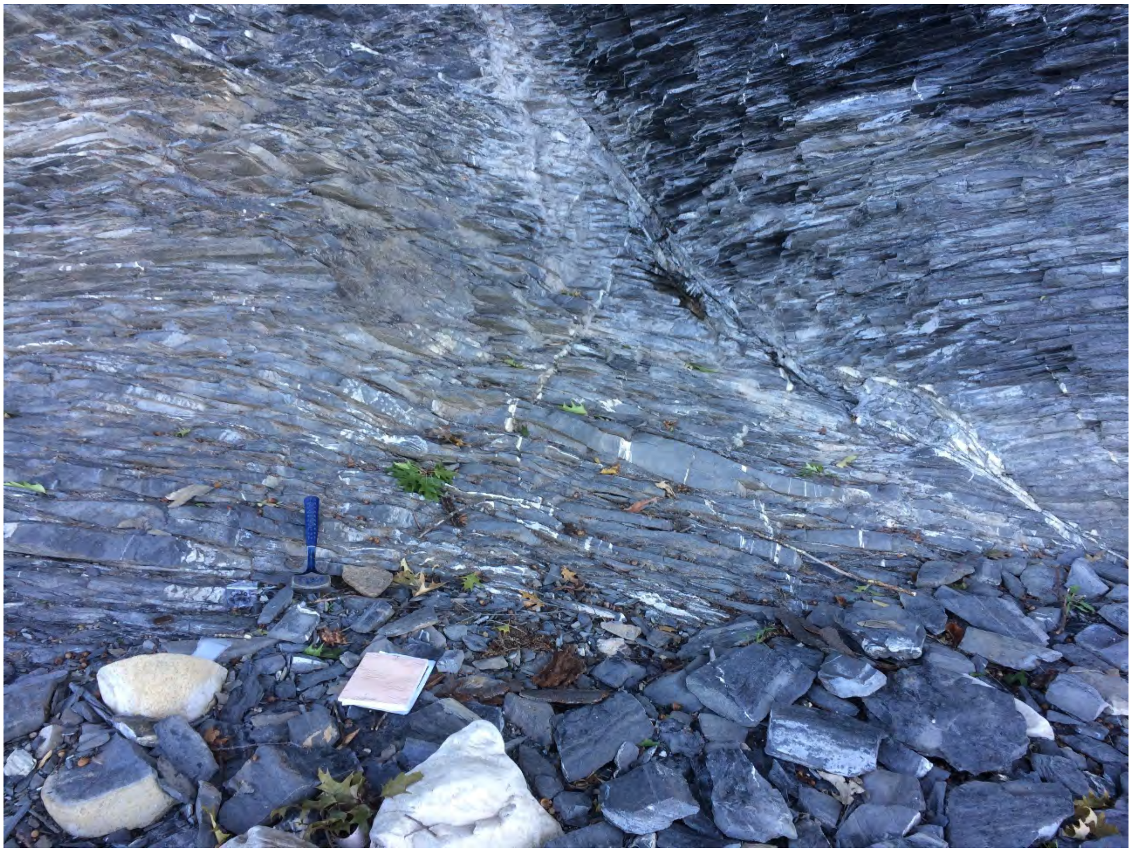

C230A:

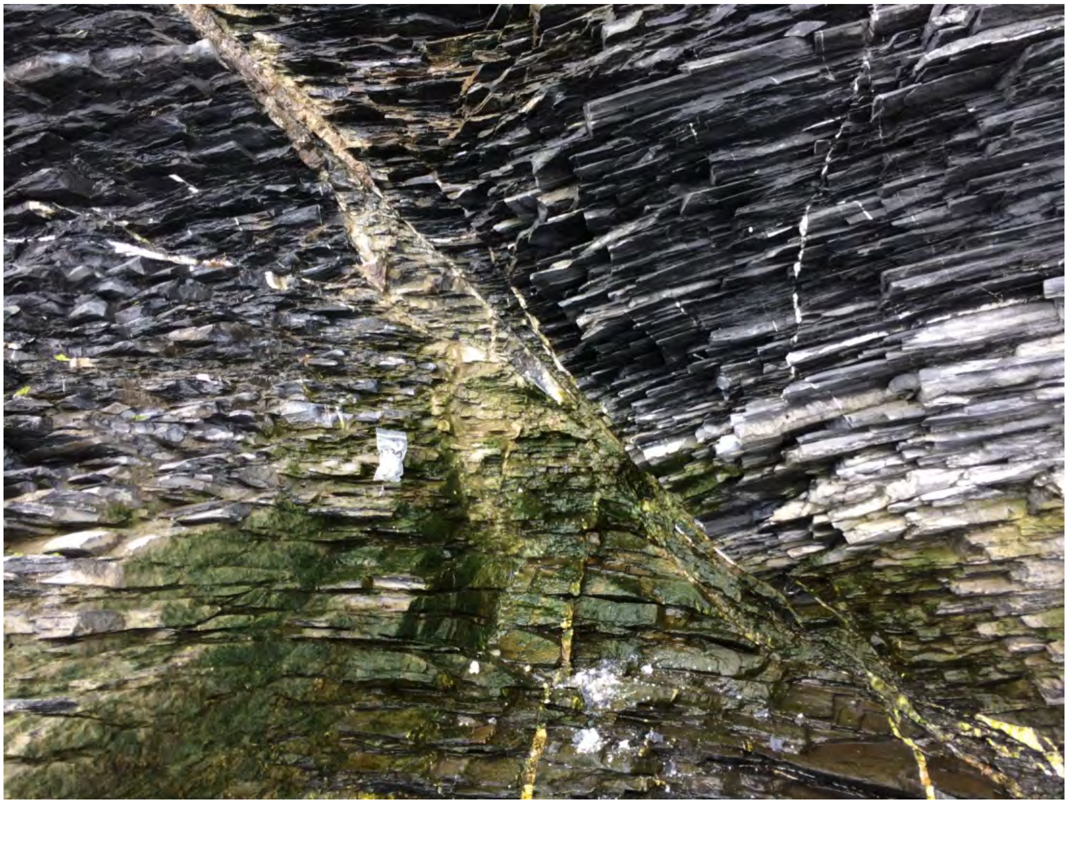

C230B: Middle horsetail

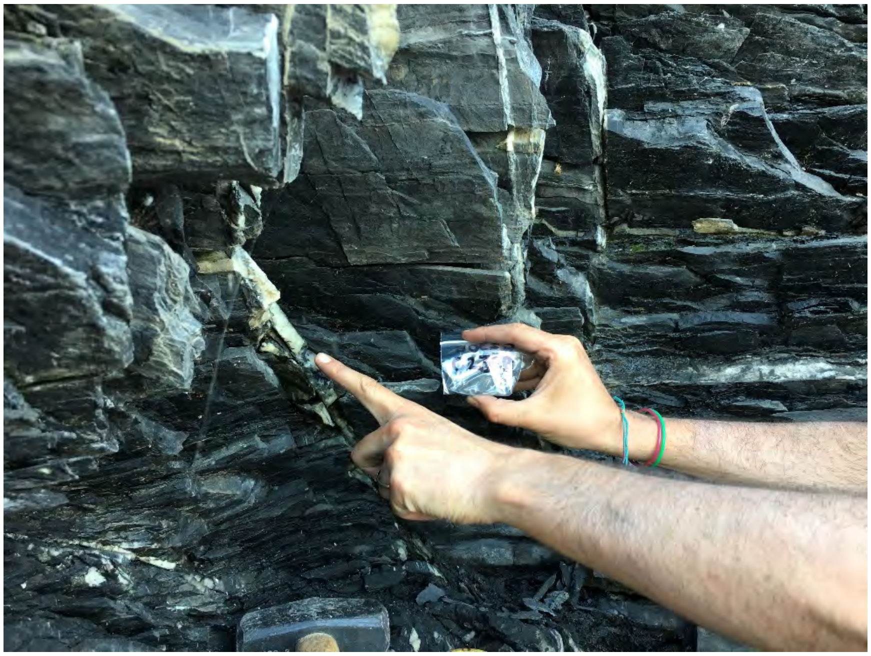

C230C: No photo ???

C230D:

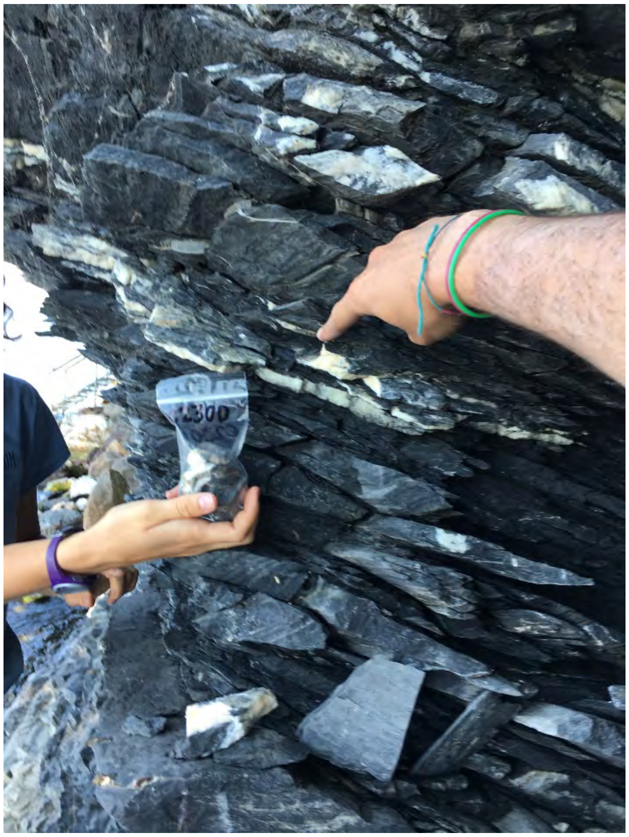

C230E:

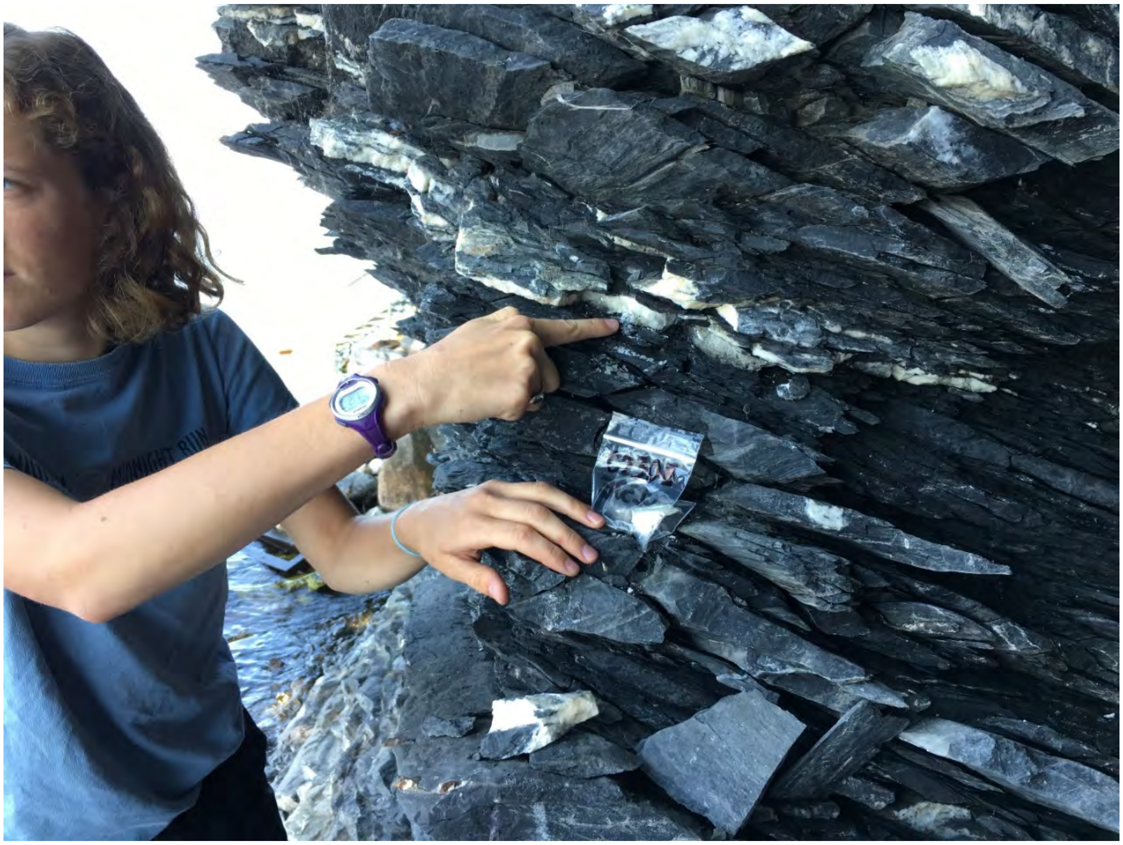

C230F:

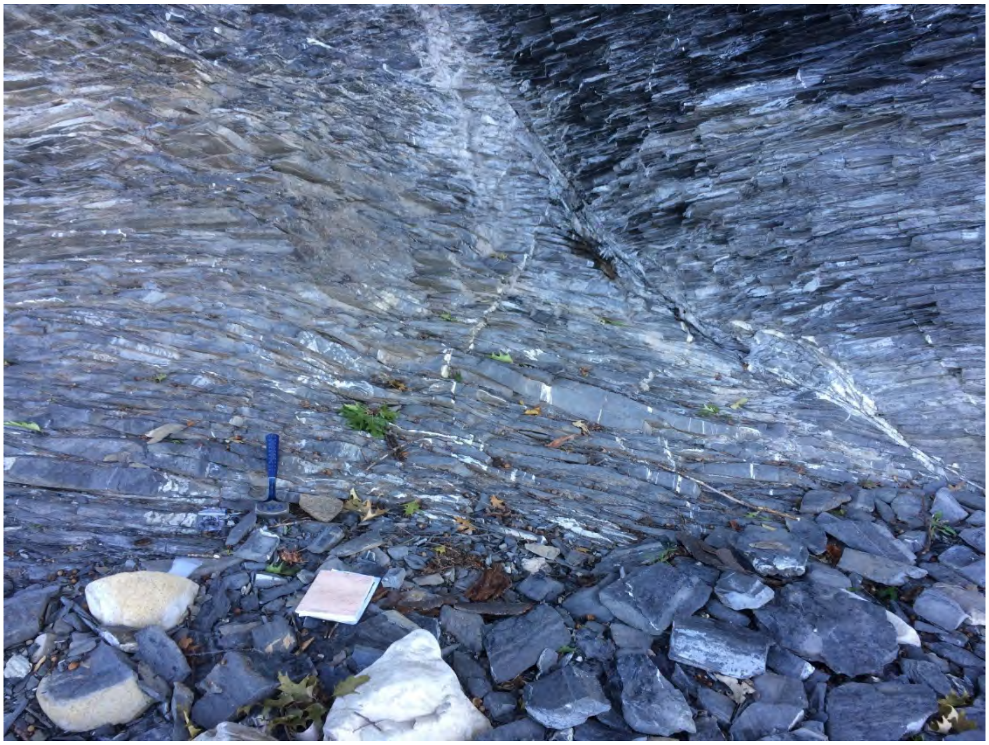

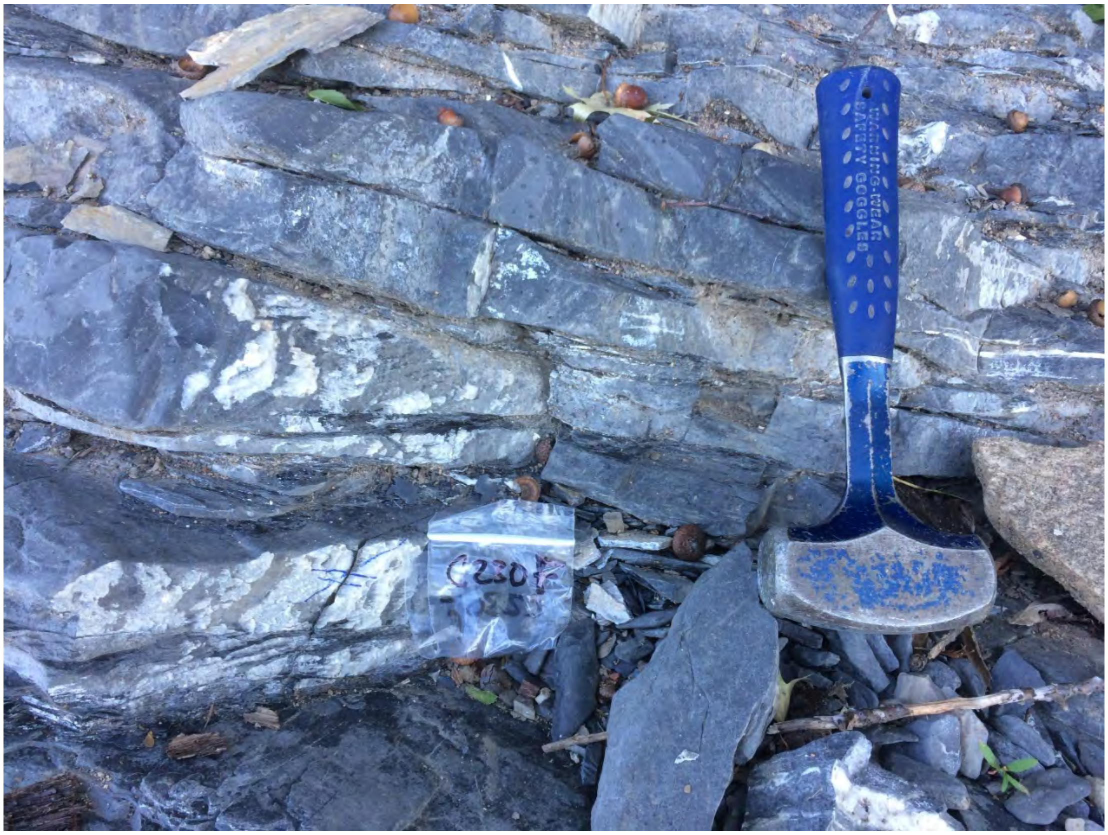

C230H

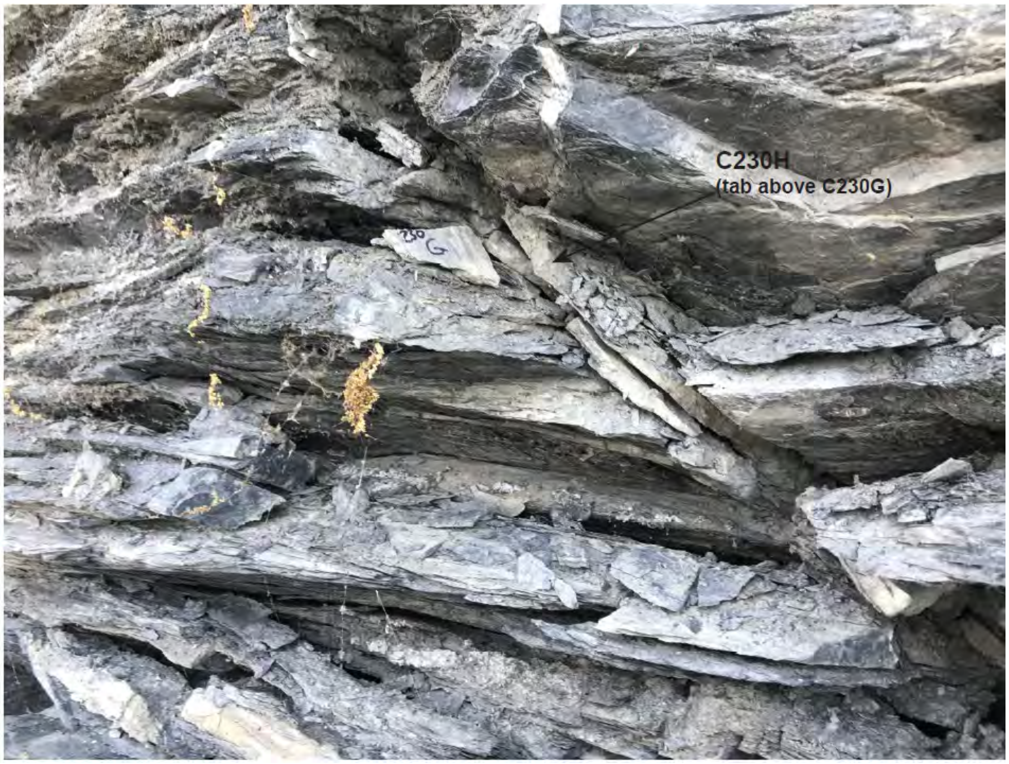

C230I

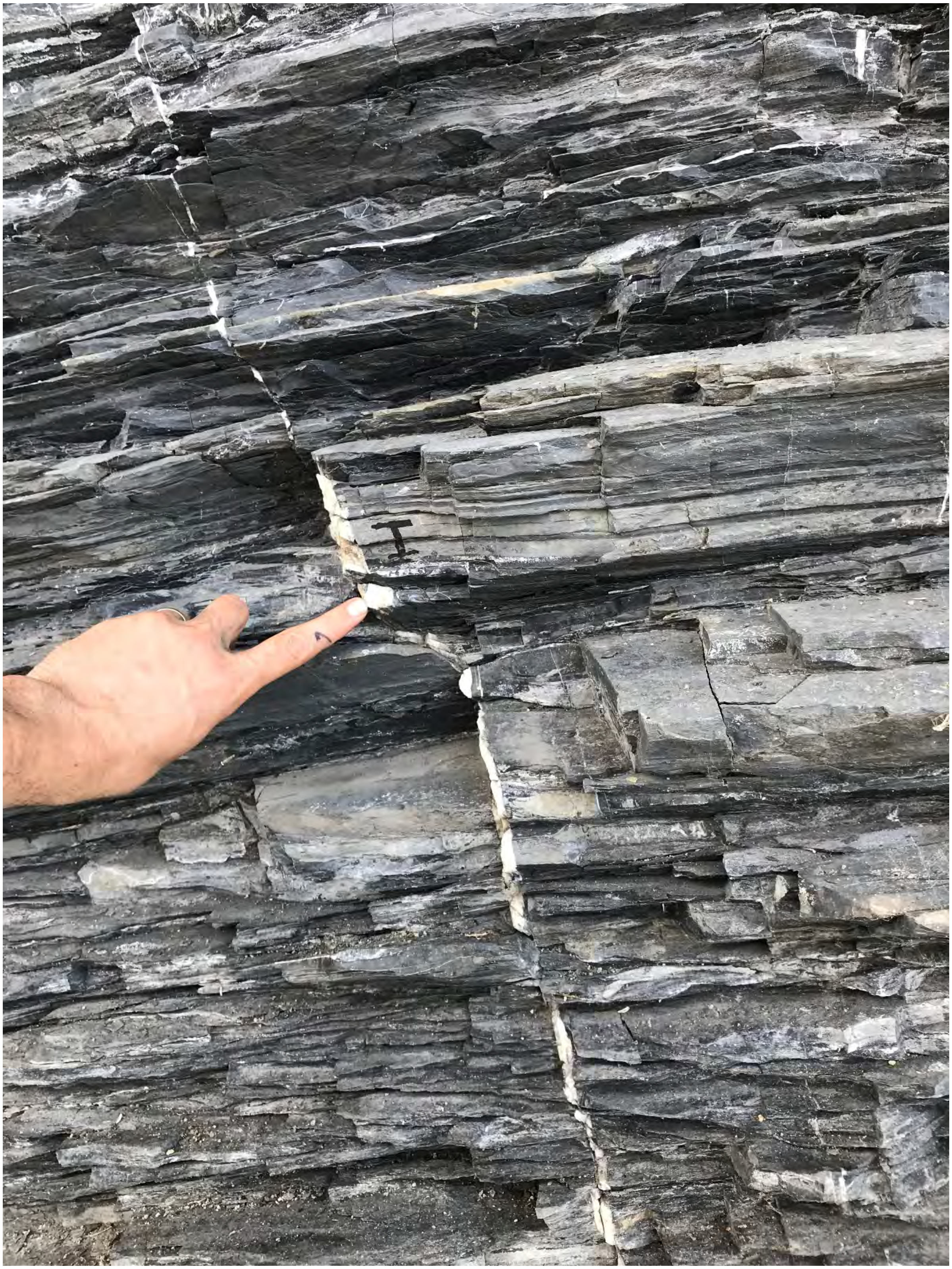

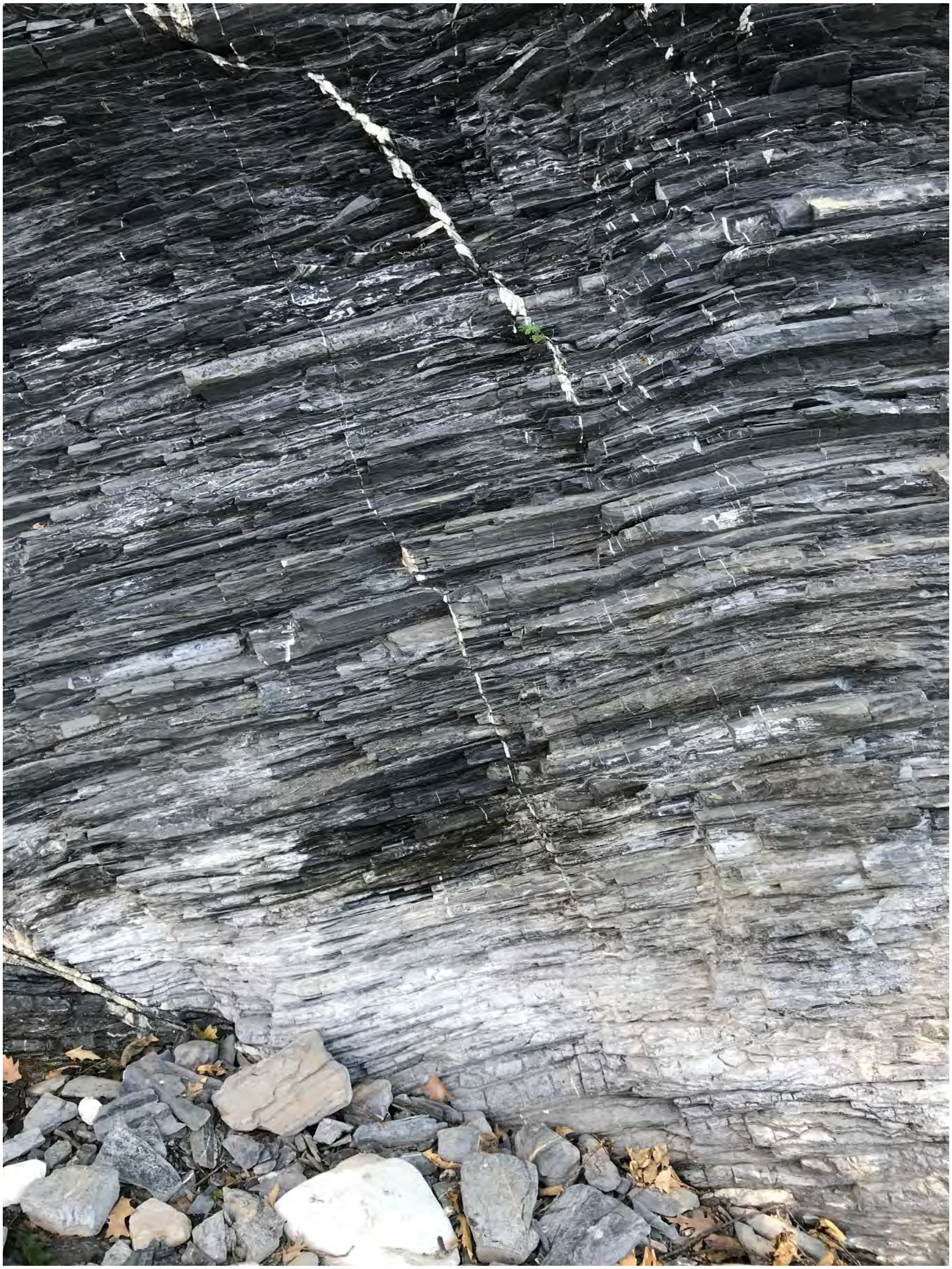

<u>Lab Description</u>:

C230A:

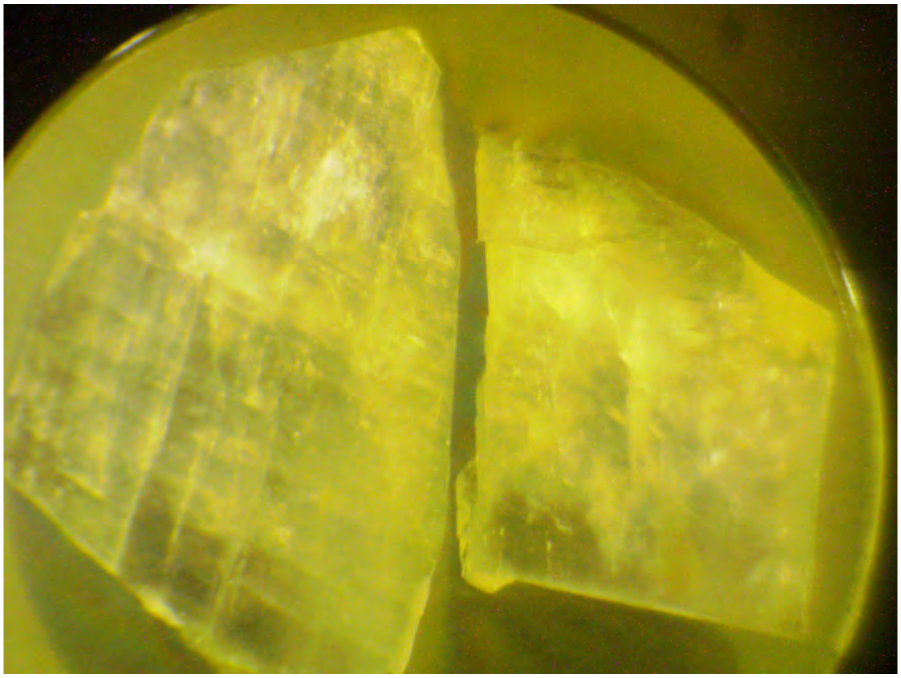

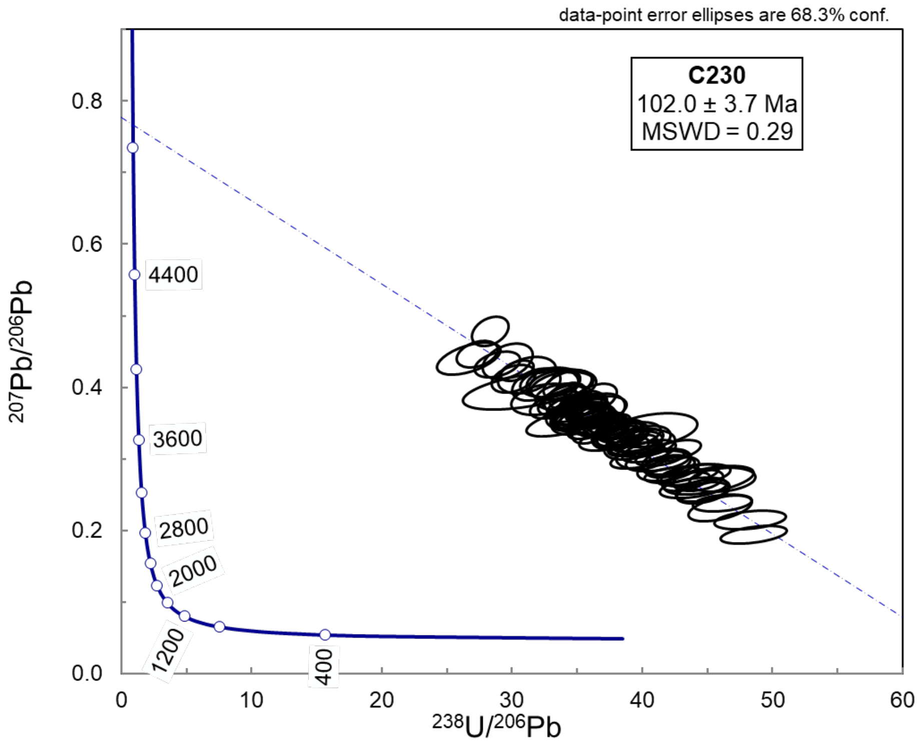

C230B:

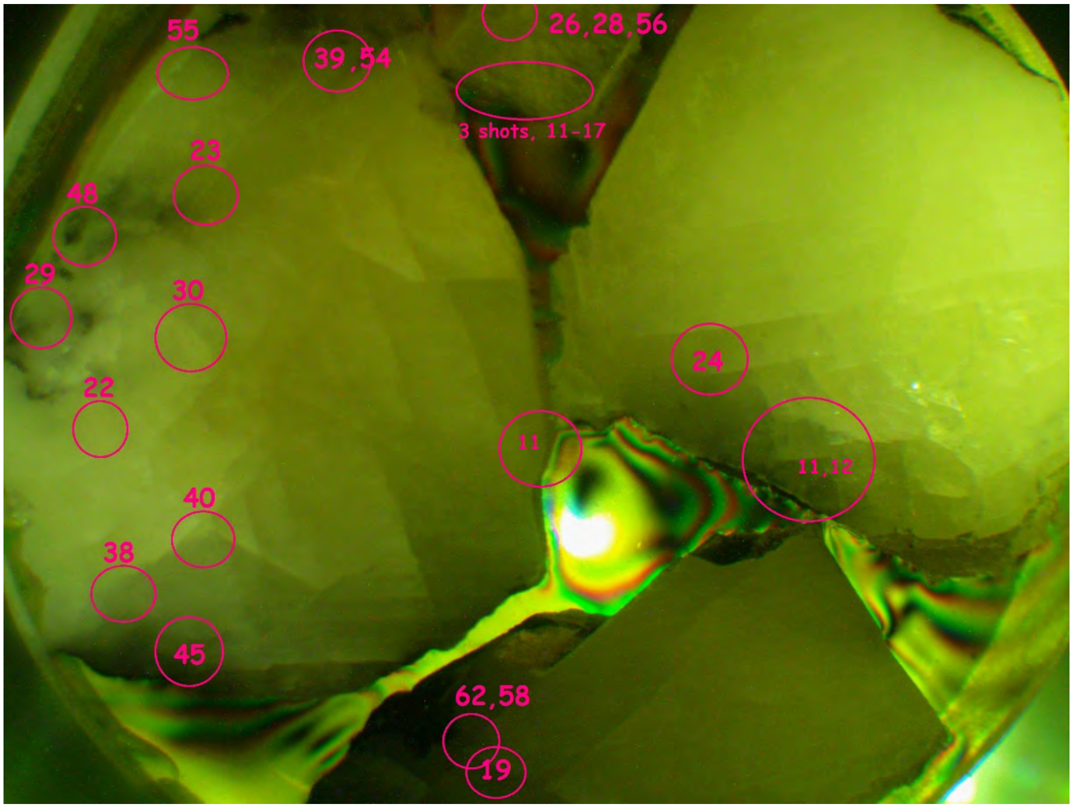
55
39,54
26,28,56
3 shots, 11-17
23
48
29
30
22
24
11
11,12
40
38
45
62,58
19

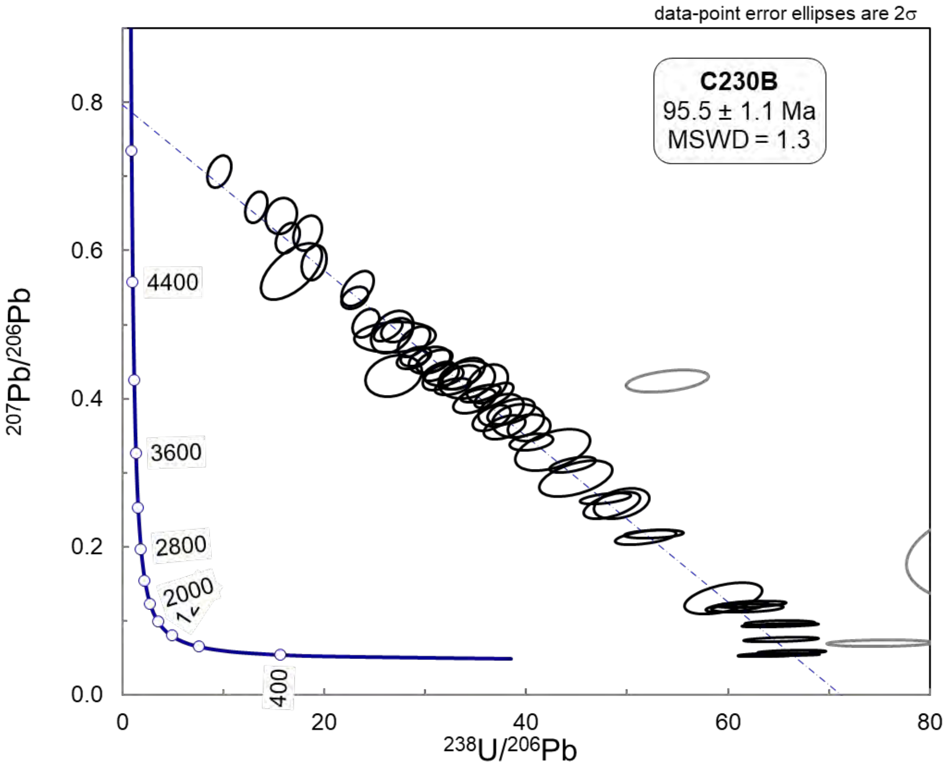
data-point error ellipses are 2σ
C230B
95.5 ± 1.1 Ma
MSWD = 1.3
0.8
0.6
0.4
0.2
0.0
4400
3600
2800
2000
400
0
20
40
60
80
$^{207}Pb/^{206}Pb$
$^{238}U/^{206}Pb$

C230C: Up-dip from C230B in middle Horsetail

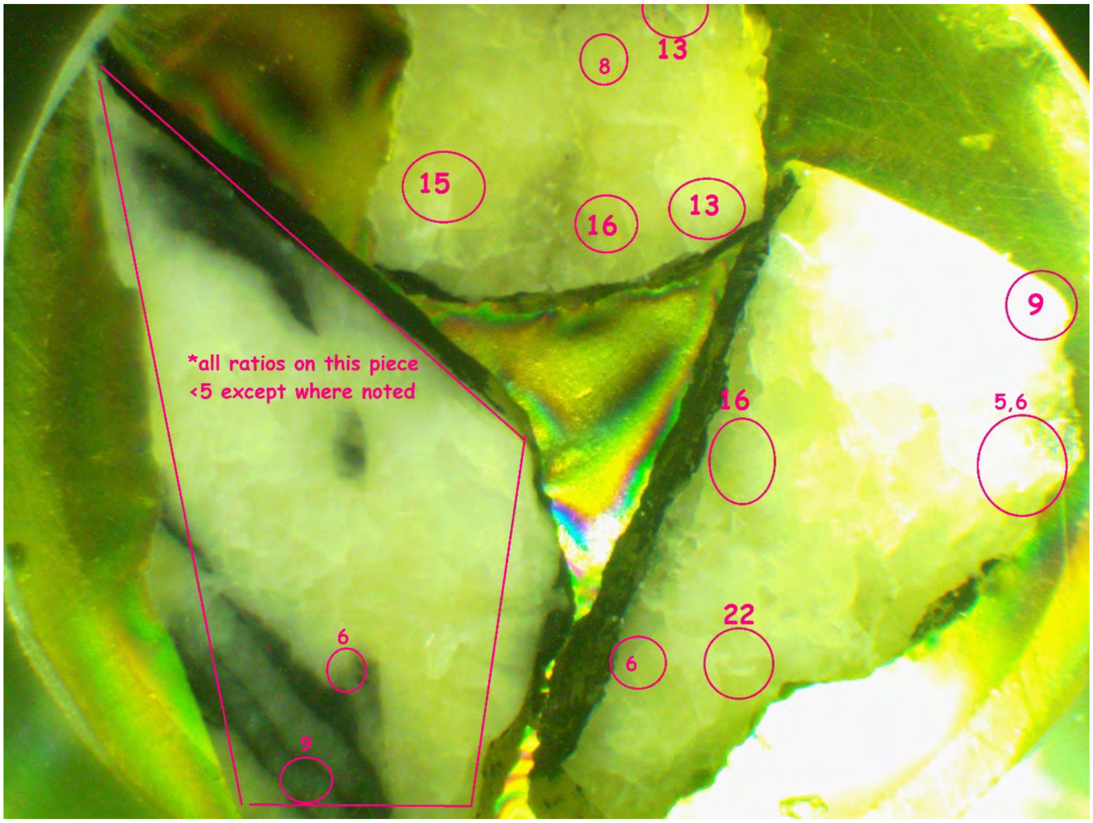

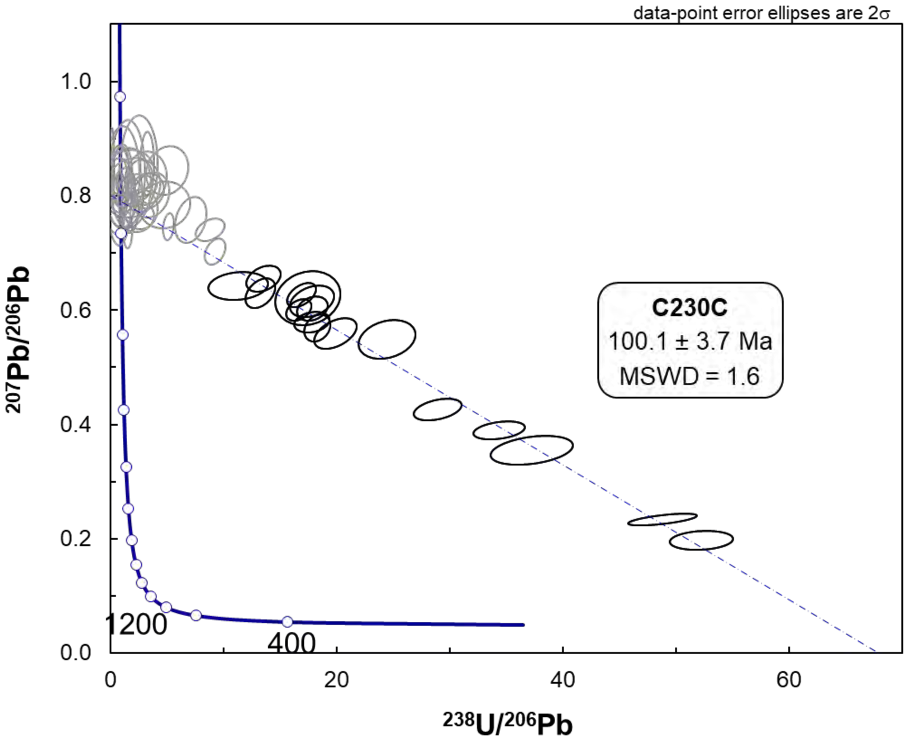

C230D:

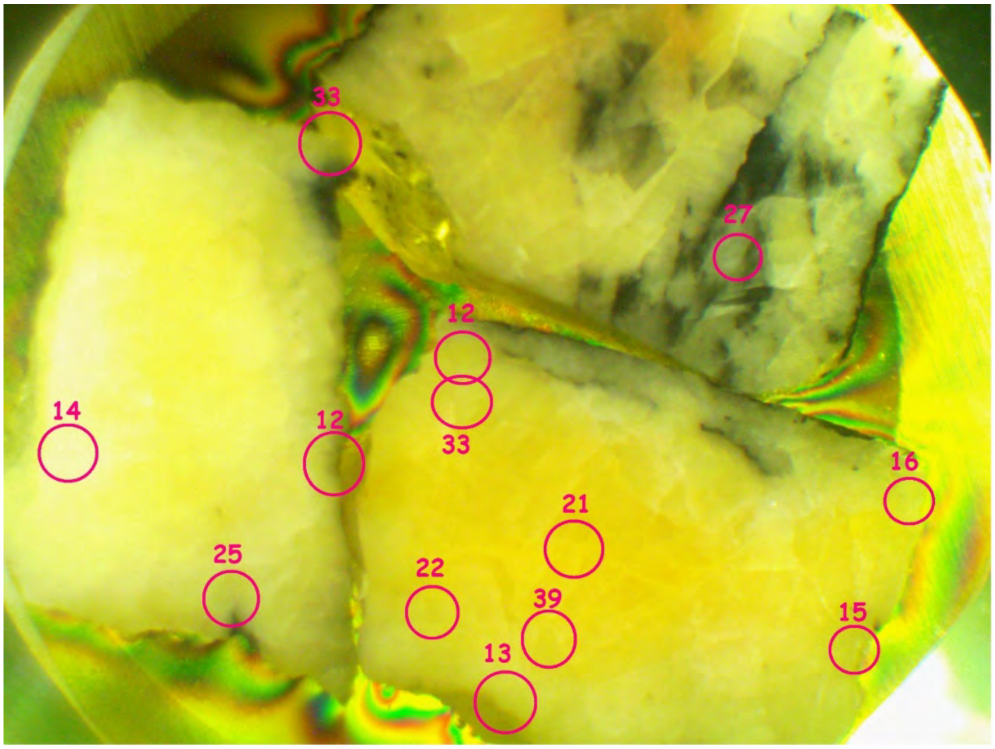
33
27
12
14
12
33
16
21
25
22
39
15
13

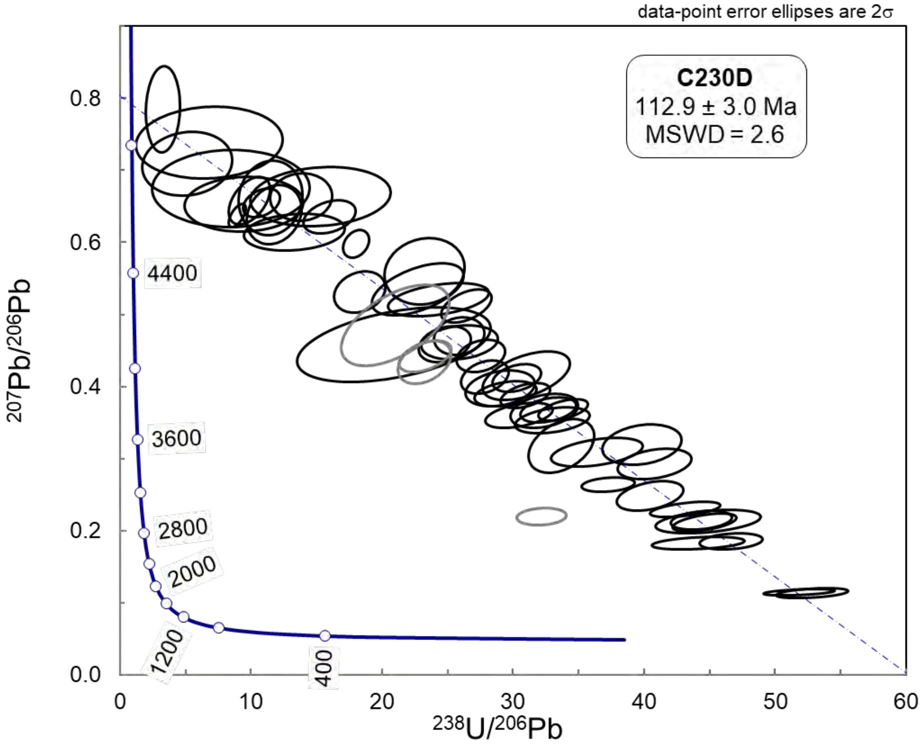
data-point error ellipses are 2σ
C230D
112.9 ± 3.0 Ma
MSWD = 2.6
0.8
0.6
0.4
0.2
0.0
4400
3600
2800
2000
1200
400
0
10
20
30
40
50
60
207Pb/206Pb
238U/206Pb

C230E:

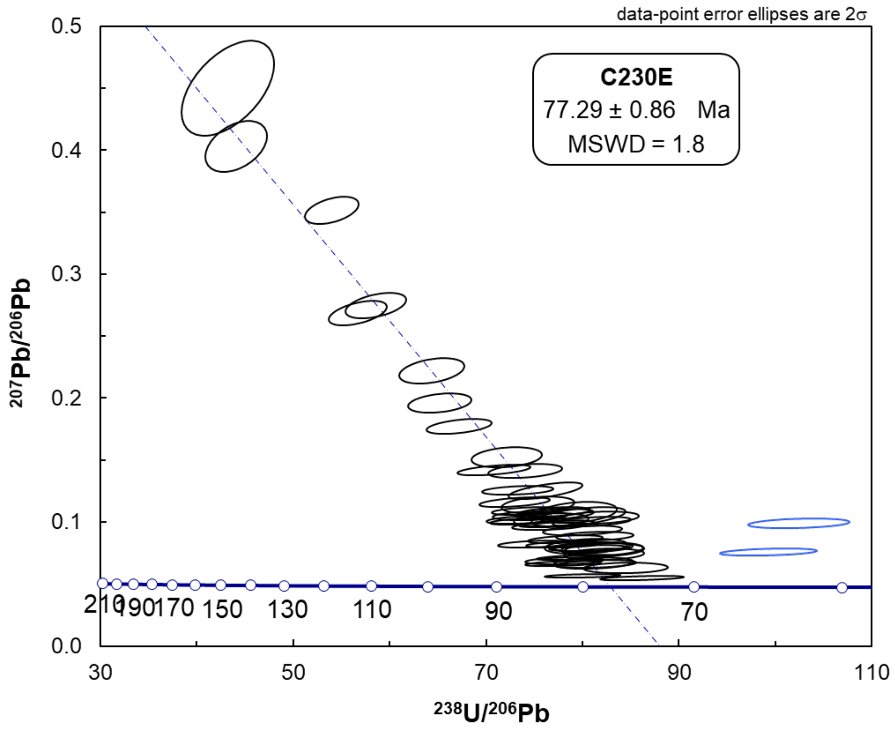
data-point error ellipses are 2σ
C230E
77.29 ± 0.86 Ma
MSWD = 1.8
207Pb/206Pb
238U/206Pb
0.5
0.4
0.3
0.2
0.1
0.0
30
50
70
90
110
210
190
170
150
130
110
90
70

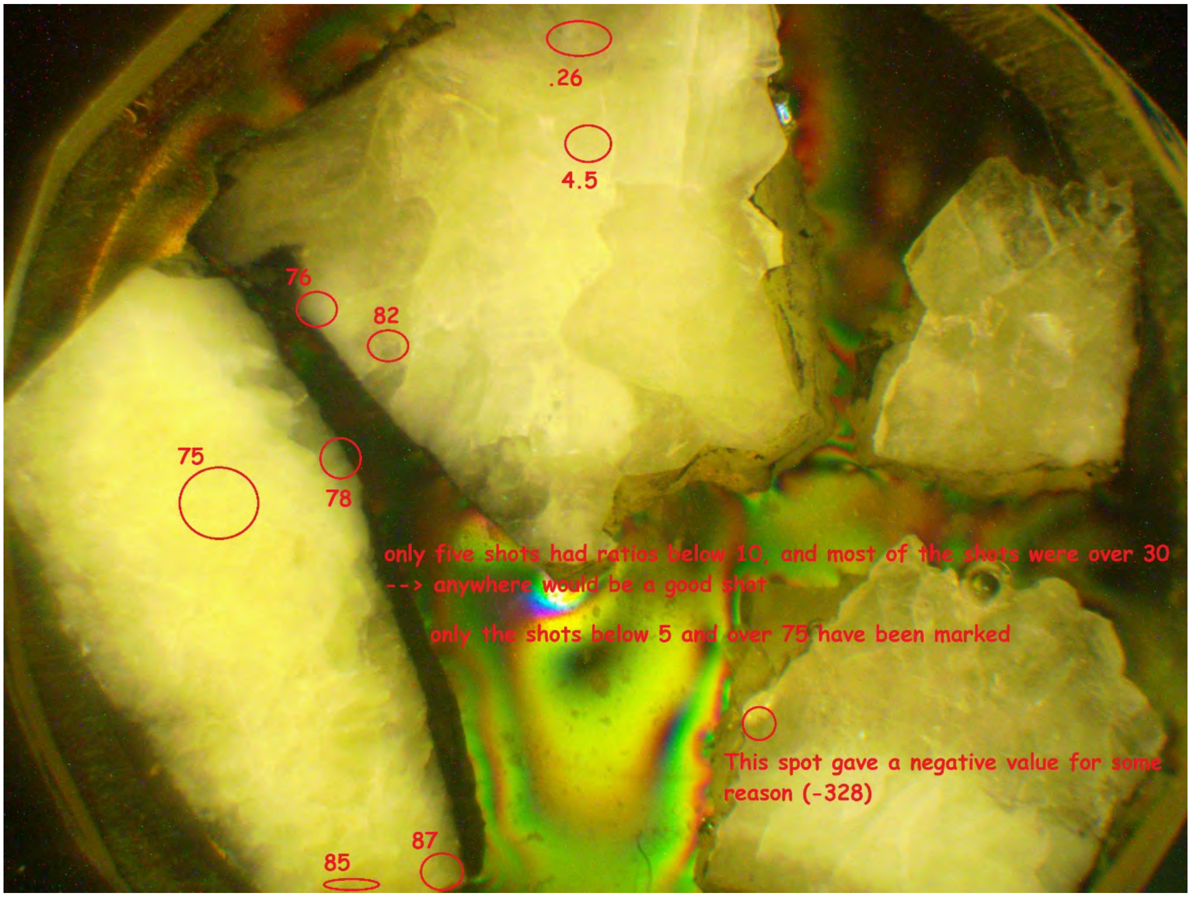
.26
4.5
76
82
75
78
only five shots had ratios below 10, and most of the shots were over 30
--> anywhere would be a good shot
only the shots below 5 and over 75 have been marked
This spot gave a negative value for some reason (-328)
85
87

C230F:

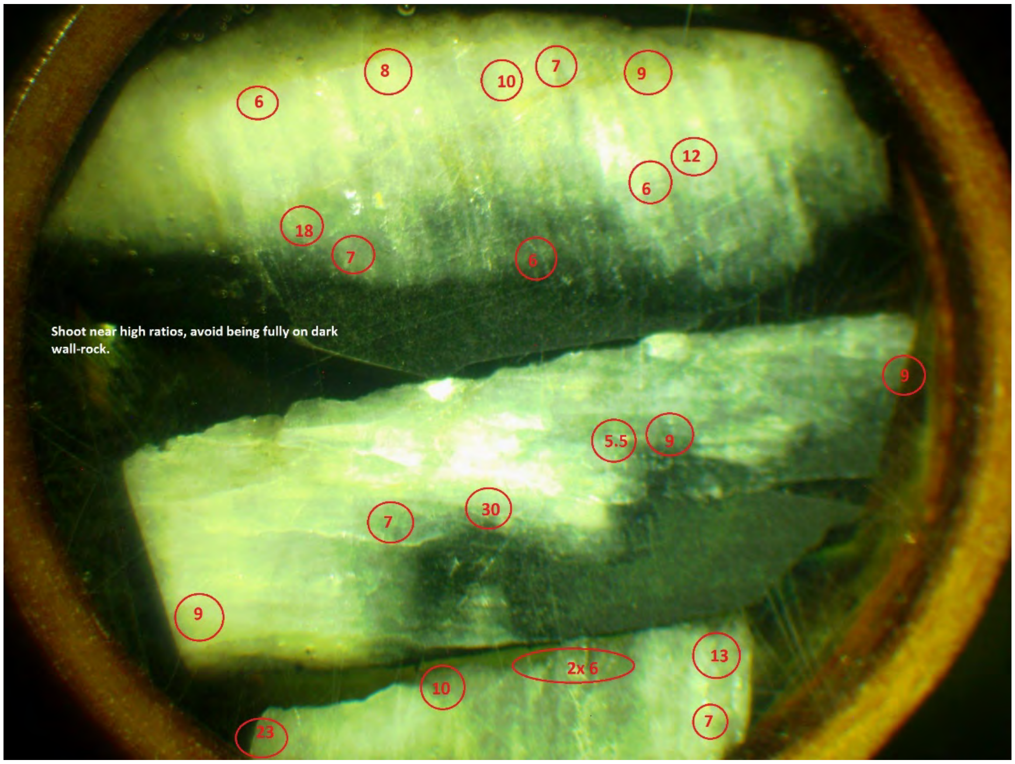
6
8
10
7
9
12
6
18
7
6
Shoot near high ratios, avoid being fully on dark wall-rock.
9
5.5
9
7
30
9
13
2x 6
10
7
23

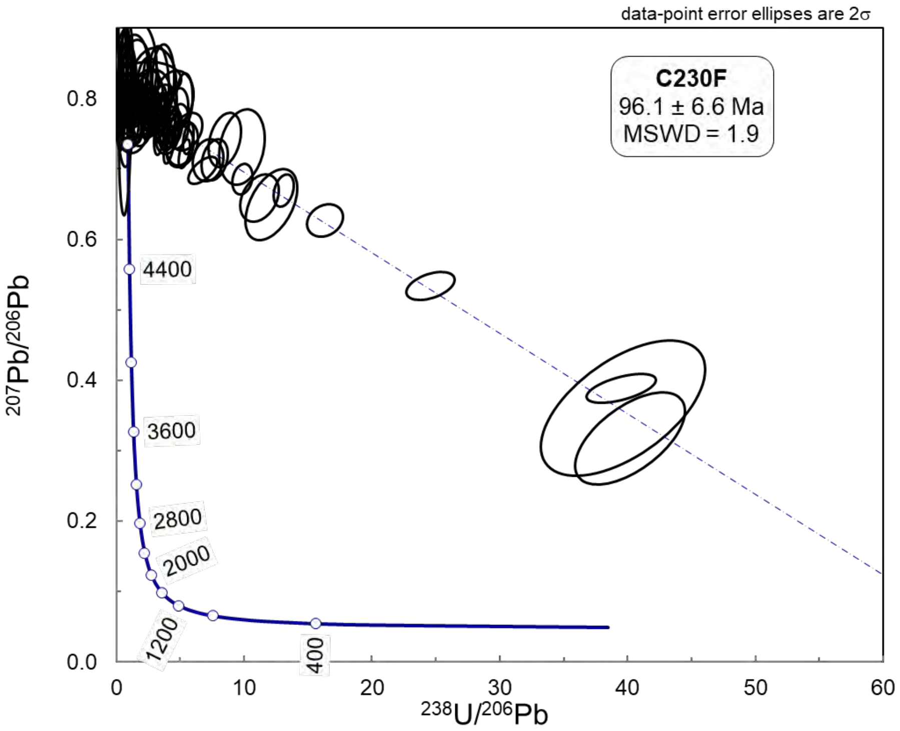
data-point error ellipses are 2σ
C230F
96.1 ± 6.6 Ma
MSWD = 1.9
0.8
0.6
0.4
0.2
0.0
4400
3600
2800
2000
1200
400
0
10
20
30
40
50
60
207Pb/206Pb
238U/206Pb

C230H:

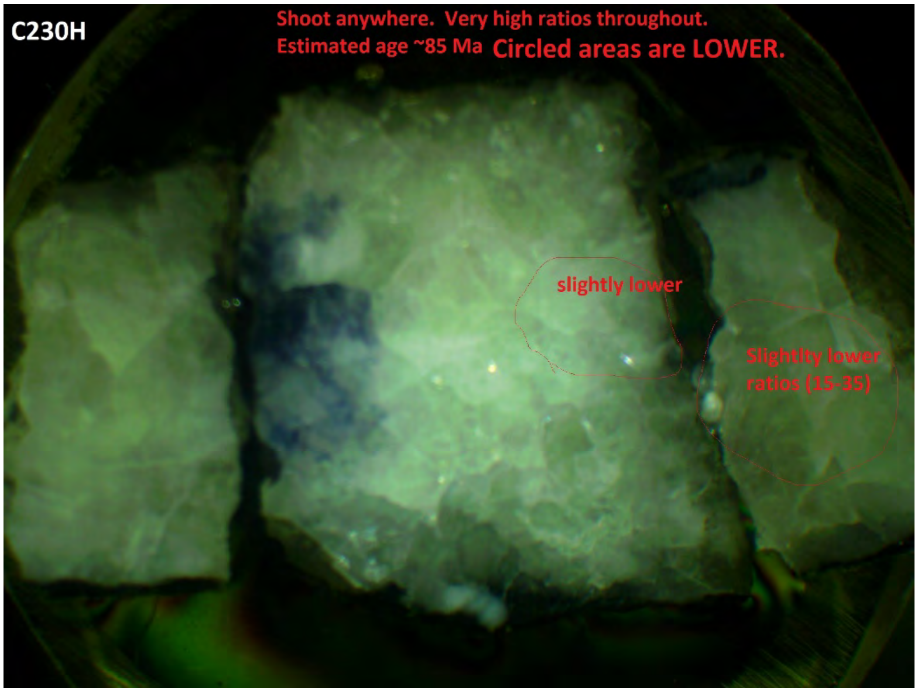
C230H
Shoot anywhere. Very high ratios throughout.
Estimated age ~85 Ma
Circled areas are LOWER.
slightly lower
Slightlty lower
ratios (15-35)

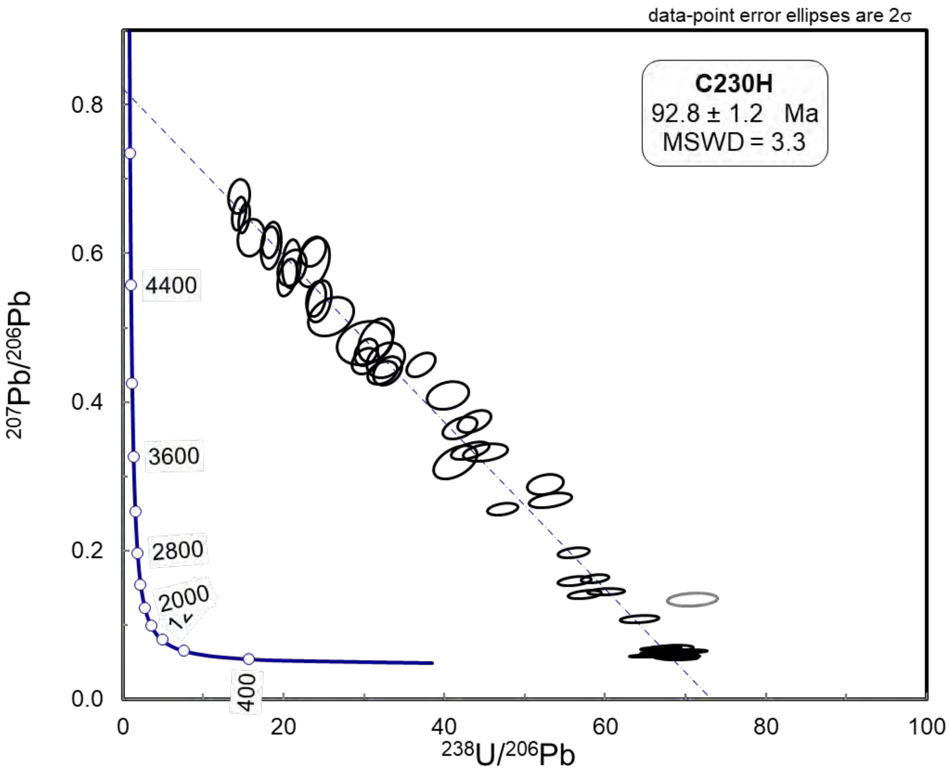
data-point error ellipses are 2σ
C230H
92.8 ± 1.2 Ma
MSWD = 3.3
0.8
0.6
0.4
0.2
0.0
4400
3600
2800
2000
400
0
20
40
60
80
100
238U/206Pb
207Pb/206Pb

C230I:

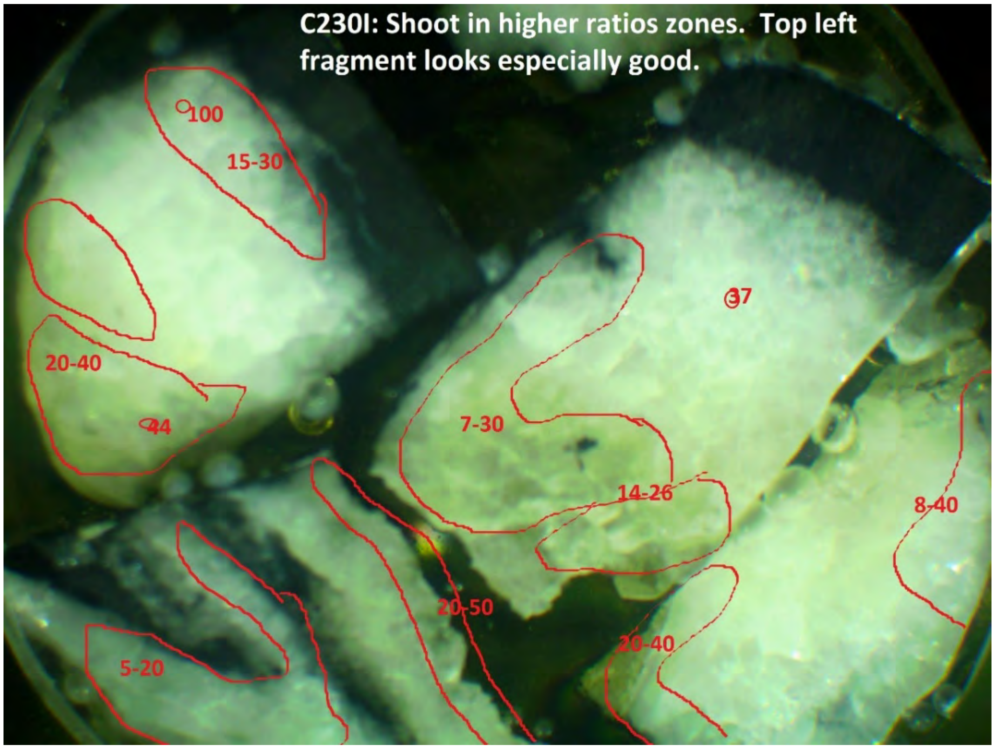
C230I: Shoot in higher ratios zones. Top left fragment looks especially good.
100
15-30
37
20-40
44
7-30
14-26
8-40
20-50
20-40
5-20

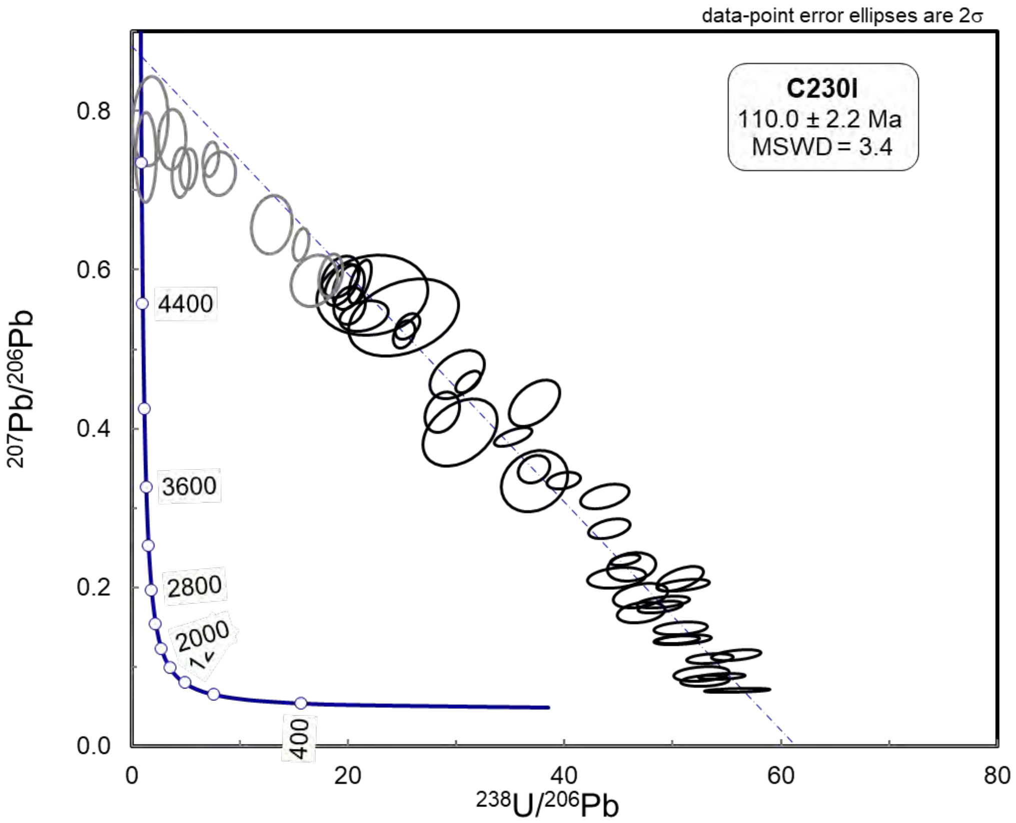
data-point error ellipses are 2σ
C230I
110.0 ± 2.2 Ma
MSWD = 3.4
0.8
0.6
0.4
0.2
0.0
4400
3600
2800
2000
400
0
20
40
60
80
207Pb/206Pb
238U/206Pb
