# Supplementary material for: Pace of passive margin tectonism revealed by U-Pb dating of fracture-filling calcite
Source: Nat Commun. 2022 Apr 12;13:1953. doi: 10.1038/s41467-022-29680-z (PMC9005700; doi:10.1038/s41467-022-29680-z)

43: Rte. 22, Putnam Center North, NY (43.750374, -73.399768)

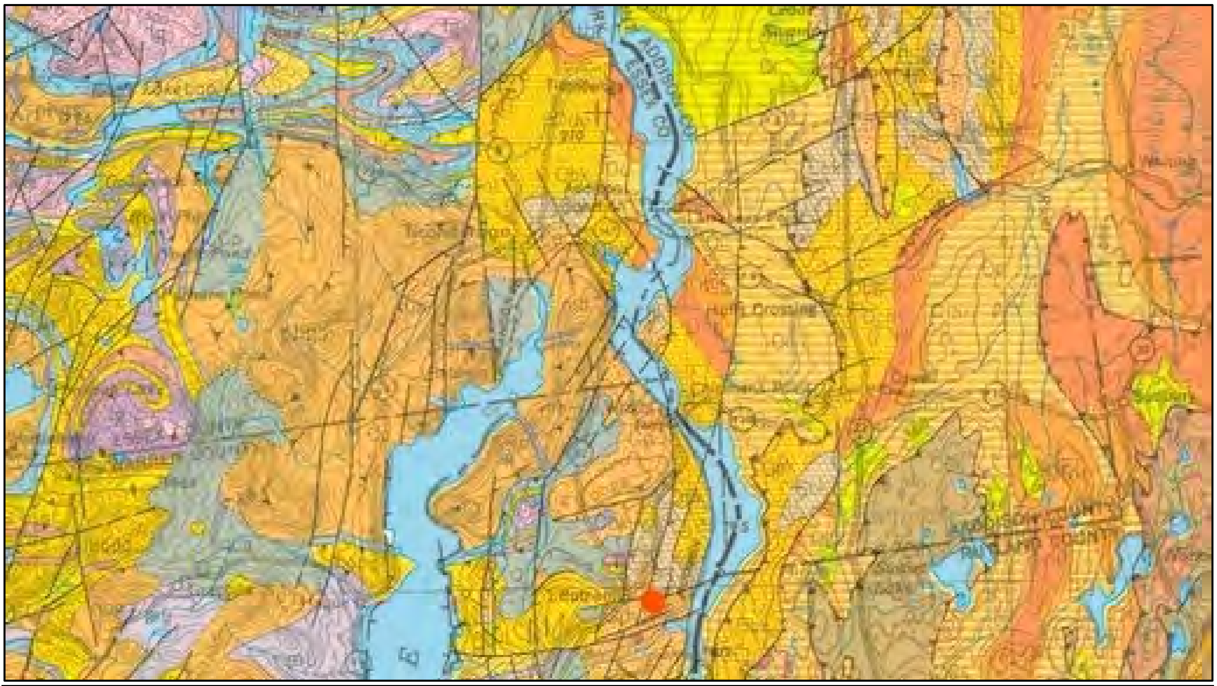

Figure 1: Sample located on Adirondack geologic map of Isachsen and Fisher, 1970.

Failed samples from same site: C437a

Bedrock Unit: Gneiss

Ages: **C437B**: 429 ± 13 Ma

Summary: This site is a large roadcut along the west side of New York State Highway 22 south of Ticonderoga, NY. The sample comes from a 2-4 mm thick calcite vein that is planar, extensive, and continuous with no slip indicators. It has not been revisited since its initial sampling and we thus offer no kinematic interpretations. The age of ~ 429 Ma suggests it may be associated with the Taconic orogeny.

Field Observations:

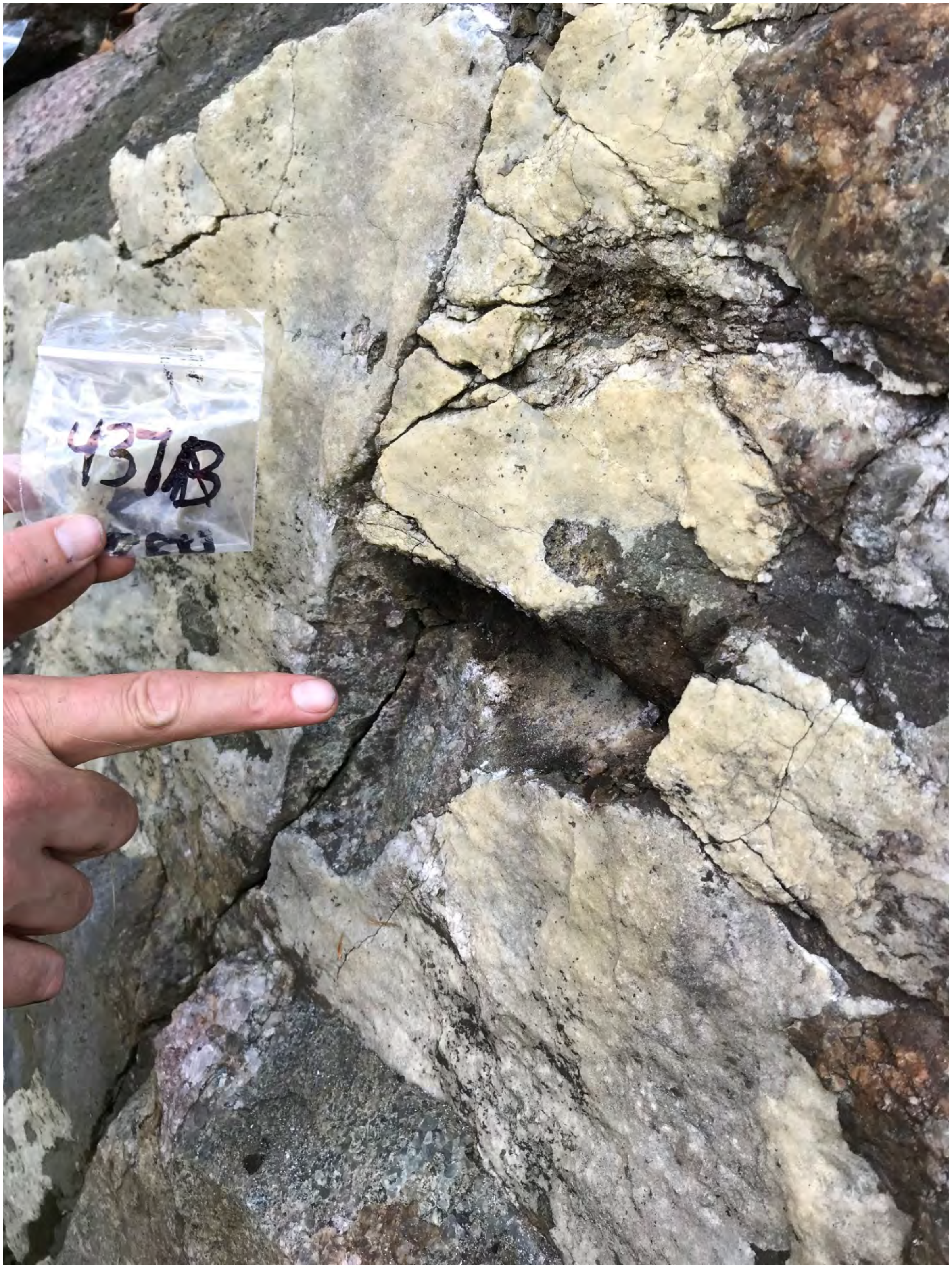

Lab Description:

C437B:

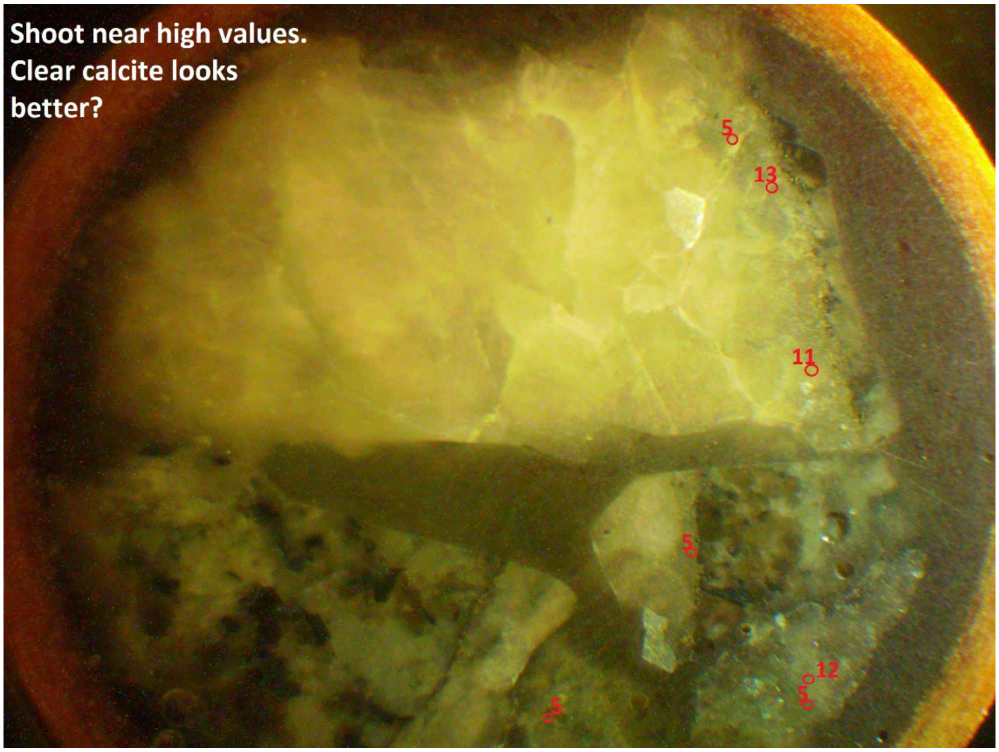

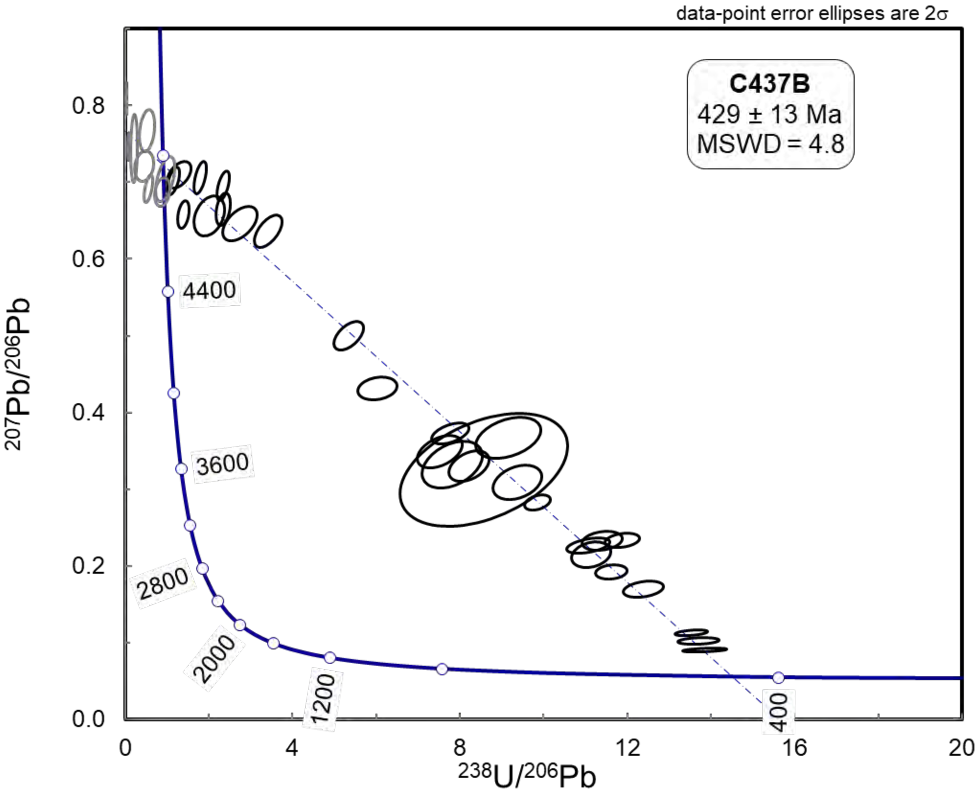

Supplement: Supplementary file 6 — Source Data [file 41467_2022_29680_MOESM6_ESM.zip › 2.zip!/appendix2b_site_summaries_21to50/43_rte22_putnam_center_north_final.pdf]
